# Inhibition of merozoite invasion and transient de-sequestration by sevuparin in humans with *Plasmodium falciparum* malaria

**DOI:** 10.1371/journal.pone.0188754

**Published:** 2017-12-15

**Authors:** Anna M. Leitgeb, Prakaykaew Charunwatthana, Ronnatrai Rueangveerayut, Chirapong Uthaisin, Kamolrat Silamut, Kesinee Chotivanich, Patima Sila, Kirsten Moll, Sue J. Lee, Maria Lindgren, Erik Holmer, Anna Färnert, Mpungu S. Kiwuwa, Jens Kristensen, Christina Herder, Joel Tarning, Mats Wahlgren, Arjen M. Dondorp

**Affiliations:** 1 Modus Therapeutics AB, Stockholm, Sweden; 2 Faculty of Tropical Medicine, Mahidol University, Bangkok, Thailand; 3 Mae Sot Hospital, Mae Sot, Thailand; 4 Mae Ramat Hospital, Mae Ramat, Thailand; 5 Department of Microbiology, Tumor- and Cell Biology, Karolinska Institutet, Stockholm, Sweden; 6 Nuffield Department of Medicine, University of Oxford, Oxford, United Kingdom; 7 Department of Infectious Diseases, Karolinska University Hospital and Department Medicine Solna, Karolinska Institutet, Stockholm, Sweden; 8 Department of Pediatrics, School of Medicine, Makerere University College of Health Sciences, and Department of Biochemistry, School of Biomedical Sciences, Makerere University College of Health Sciences, Kampala, Uganda; George Washington University School of Medicine and Health Sciences, UNITED STATES

## Abstract

**Severe malaria:**

Even with the best available treatment, the mortality from severe *Plasmodium falciparum* malaria remains high. Typical features at death are high parasite loads and obstructed micro- vasculature. Infected erythrocytes (IE) containing mature parasites bind to the host receptor heparan sulfate, which is also an important receptor for merozoite invasion. To block merozoite invasion has not previously been proposed as an adjunctive therapeutic approach but it may preclude the early expansion of an infection that else leads to exacerbated sequestration and death.

**Sevuparin in phase I study:**

The drug sevuparin was developed from heparin because heparan sulfate and heparin are nearly identical, so the rationale was that sevuparin would act as a decoy receptor during malaria infection. A phase I study was performed in healthy male volunteers and sevuparin was found safe and well tolerated.

**Sevuparin in phase I/II clinical study:**

A phase I/II clinical study was performed in which sevuparin was administered via short intravenous infusions to malaria patients with uncomplicated malaria who were also receiving atovaquone/proguanil treatment. This was a Phase I/II, randomized, open label, active control, parallel assignment study.

Sevuparin was safe and well tolerated in the malaria patients. The mean relative numbers of ring-stage IEs decreased after a single sevuparin infusion and mature parasite IEs appeared transiently in the circulation. The effects observed on numbers of merozoites and throphozoites in the circulation, were detected already one hour after the first sevuparin injection. Here we report the development of a candidate drug named sevuparin that both blocks merozoite invasion and transiently de-sequesters IE in humans with *P*. *falciparum* malaria.

**Trial registration:**

ClinicalTrials.gov NCT01442168

## Introduction

Severe malaria due to *P*. *falciparum* claims 430,000 lives annually, despite the use of the best anti-malarial medications currently available [1]. Improvements of supportive care, including novel drugs, could lower the fatality rate and diminish neurological- and cognitive dysfunctions seen in some survivors of the four million or so who develop severe malaria each year. Historically, adjunct therapies have been found to be of benefit to animals in models of severe malaria [2–4]. However, the effects have not been possible to reproduce in humans infected with *P*. *falciparum* likely because the hosts, the parasites and the pathology that bring about severe malaria are only partially alike in animals and man [5–8]. Indeed, no adjunct treatment with acceptable safety profile has been shown to be of benefit to humans with severe malaria [9]. Excessive sequestration of *P*. *falciparum* infected erythrocytes (IE) and inflammation are typical features of severe malaria but it is also known that patients with complicated disease carry higher parasite loads, a larger biomass, than those with mild malaria [10]. We hypothesized that ring stage parasitemia in *P*. *falciparum* infected humans is maintained by an asynchronous rupture of schizonts, the subsequent invasion of erythrocytes by merozoites and the development of the parasites into ring- and adhesive trophozoite stage IE. The time of development of a merozoite into an adhesive IE that may obstruct the micro-vasculature is about 18–20 hours and the majority of deaths in severe malaria occur the first day of hospitalization during an equivalent timespan [11–13]. In historical studies, heparin has been found to reduce the mortality from severe *P*. *falciparum* malaria if it was given as an adjunct to anti-plasmodial drugs, but its use was stopped because of the risk of hemostatic disorders [14–16]. Importantly, the inhibitory effect of heparin on *P*. *falciparum* sequestration and merozoite invasion was found to be independent of its anti-coagulant activity [17–26]. Notably, preclinical investigations have demonstrated that sevuparin, an acidic, negatively charged, antiadhesive polysaccharide drug manufactured from heparin with eliminated antithrombin (AT) binding site, affects both merozoite invasion and sequestration of IE.

Sevuparin blocks *P*. *falciparum* merozoite invasion into fresh erythrocytes *in vitro*, and both disrupts and blocks the binding of IE to uninfected erythrocytes (rosetting) and binding to vascular endothelial cells (cytoadherence) *in vitro*. Furthermore, from i*n vivo* studies sevuparin has been demonstrated to block IE from binding in the micro-vasculature of the rat but also to de-sequester IE, i.e. release already sequestered IE into circulation, both in rats and in a non-human primate [25–27]. In addition, sevuparin binds to the N-terminal extracellular, heparan sulfate binding structure of Plasmodium falciparum erythrocyte membrane protein 1 (PfEMP1), the Duffy-binding like domain 1α (DBL1α), known as a vital contributor to sequestration of IE [28].

Here we report the results of both a phase I study in healthy volunteers and a clinical study with sevuparin in patients with *P*. *falciparum* malaria.

## Methods

### Sevuparin

**Fig 1:**
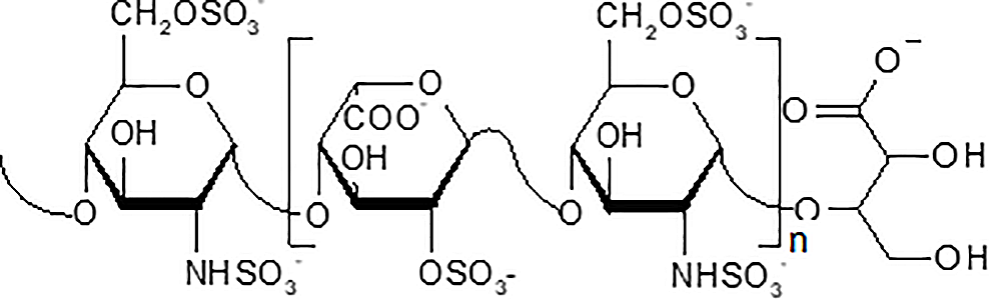
Sevuparin is an acidic, negatively charged, antiadhesive polysaccharide drug. The predominant structure of sevuparin with the main monosaccharide constituents. The weight average molecular weight of sevuparin is 6.5–9.5 kDa.

Sevuparin, like other members of the chemical class of heparins, is polydisperse, encompassing a range of polysaccharide chain lengths. The predominant size is 6–16 disaccharide units of 2-N-sulfo-6-O-sulfo-glucosamine and iduronic-2-O-sulfate acid, which corresponds to molecular weights of 3.6–9.6 kDa. The weight average molecular weight is 6.5–9.5 kDa. The predominant chemical structure of sevuparin is illustrated in Fig 1.

Sevuparin does not contain the specific binding sequence for AT which is the main contributor to prolonged coagulation and as a consequence it has minimal anticoagulant potency compared to other heparinoids [29]. The anticoagulant potency of sevuparin was determined, as part of batch release specifications, by the Pharmacopoeia Europaea (Ph. Eur.) assays designed to measure the ability to potentiate inhibition of the coagulation factors Xa and IIa (thrombin) by anti-thrombin (AT), [30,31]. for low molecular weight heparin (LMWH). As shown in Table 1, sevuparin has only a small effect on normal coagulation parameters.

**Table 1:** Anticoagulant activity of sevuparin.

Compound	MW (Da)	Anticoagulant activity	
		Anti-Xa (IU/mg)	Anti-IIa (IU/mg)
**Sevuparin**	8000	<10	<10
**Dalteparin (LMWH)**	6000	110–210	35–100
**Heparin**	15000	>180	>180

The specific anticoagulant activity of sevuparin measured *in vitro* with standard Ph. Eur. methods. The comparison values are from the monographs Fragmin 1195 (for dalteparin) and Heparin 0333.

### Clinical study—Phase I/II study in patients with uncomplicated *P*. *falciparum* malaria

**Fig 2:**
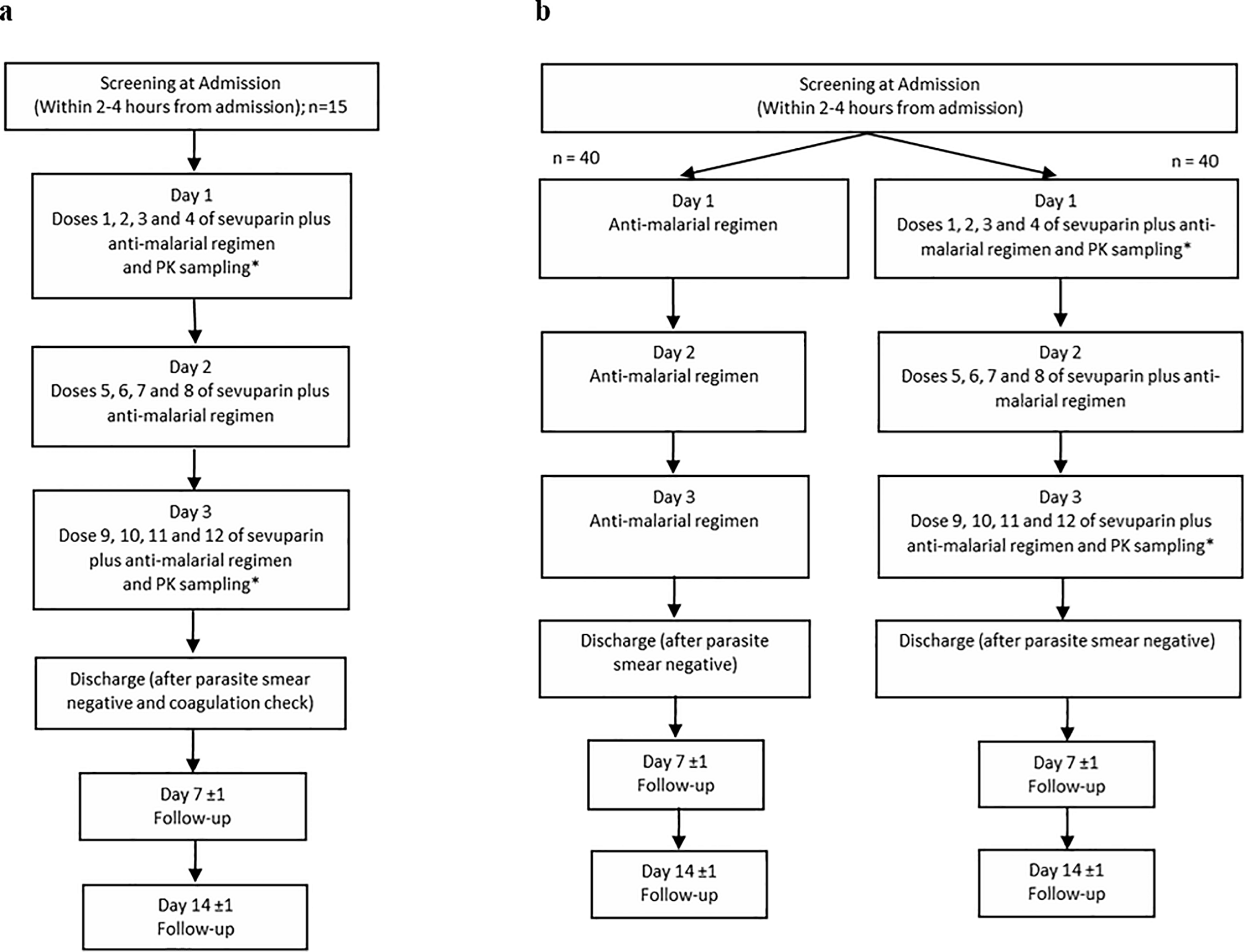
Study design of phase I/II study in patients with uncomplicated *falciparum* malaria-TSM02. a, Overview of study design and dosing regimen for all patients in part 1. b, Overview of study design and dosing regimen for patients in part 2 randomized to the anti-malarial regimen (atovaquone and proguanil) alone or sevuparin plus anti-malarial regimen (atovaquone/proguanil).

The clinical phase I/II study Treatment of Severe Malaria study 02 (TSM02) was conducted in compliance with International Conference on Harmonisation Good Clinical Practice guidelines and the Declaration of Helsinki [32]. In order to investigate sevuparin’s capability to affect malaria parasites *in vivo* in humans an exploratory proof-of–principle study was conducted. It was decided to conduct a study in uncomplicated *falciparum* malaria prior a proof-of- concept study in severe malaria patients after considering ethically limitations to go directly in to severely ill patients after only a phase I study. The phase I/II, randomized, open label, active control, parallel assignment study was conducted at Mae Sot Hospital and Mae Ramat Hospital, Tak Province Thailand between 2011 and 2014 [33]. In the areas of Mae Sot and Mae Ramat, along the Thai-Myanmar border, there were high annual malaria incidence but low and stable disease transmission with two seasonal peaks and forest-related during May-August and November-January of each year. The population has very little naturally acquired immunity against malaria [34]. The study consisted of an open labelled dose escalation phase (part 1) followed by an open labelled, randomized phase (part 2), for study design see Fig 2. Ethical approvals for the present study were obtained from the Ministry of Public Health of the Royal Government of Thailand, Ethics Committee of the Faculty of Tropical Medicine, Mahidol University, Bangkok, Thailand and from the Oxford Tropical Research Ethics Committee (OxTREC) of Oxford University, Oxford, UK.

Consecutive adult patients (18–65 years old) with slide-proven *falciparum* malaria with parasitemia counts of 10 000–100 000/ul at screening and admitted to the hospitals were enrolled in the study, provided that written informed consent was given by the patient. Patients with any sign of severe malaria, determined on the basis of World Health Organization criteria [35,36] were not eligible. Other exclusion criteria were mixed infections; history of significant bleeding; history of convulsion; use of high dose aspirin, dual anti-platelet therapy, heparin, LMWH or warfarin; significant anaemia as defined by Hb <8 g/dL or Hct < 25%; platelet count <50 000/μL; white blood counts <2000 or >12 000 cell/mcl; prothrombin time (PT) and activated partial thromboplastin time (APTT) >1.5 from upper limit normal (ULN) (PT ULN = 13.5 seconds, APTT ULN = 33 seconds); aspartate aminotransferase (AST: ULN = 40 U/L), alanine aminotransferase (ALT: ULN = 40 U/L) and alkaline phosphatase (ALP: ULN = 125 U/L) >2.5 from ULN range, pregnancy, treatment with anti-malarial drug or antibiotics with anti-malarial activity within 7 days, treatment with mefloquine within 30 days prior to current episode, clinically significant drug allergy to one or more of the study drugs, including sensitivity to heparin or LMWHs, or to products with similar chemical structures, conducted splenectomy, immunodeficiency virus infection. Participation in another clinical trial within 30 days was not allowed.

The study was designed to determine the safety, tolerability, pharmacokinetics and *in vivo* efficacy of sevuparin when administered as a short (five minutes) i.v. infusions as adjunctive therapy to standard care i.e. a fixed dose combination of 1000 mg atovaquone and 400 mg proguanil (Malanil®) once daily for three days, in subjects affected with uncomplicated *falciparum* malaria, as compared to sole atovaquone/proguanil (Malanil®) treatment (control). An artemisinin combination therapy was not used, because the broad stage specificity and the rapid onset of action of the artemisinins prevents maturation and subsequent sequestration IEs, thus obscuring the effects of sevuparin. Subjects were treated with sevuparin during three consecutive days (12 doses, 6 hours apart). In part 1 of the study, the pharmacokinetic parameters of sevuparin were studied in cohorts of 3 patients receiving 12 consecutive i.v. infusions of 1.5 mg/kg, 3 mg/kg or 6 mg/kg sevuparin in addition to standard of care. Doses were selected following a safety evaluation of the single dose data from the phase I study. The dose 1.5 mg/kg every six hour for three days corresponds the no observable effect level (NOEL) of sevuparin at 2 hours after treatment and is 4-fold below the dose of the 360 mg, which was well tolerated when given every six hours to healthy volunteers. The selected starting dose is also in the range of the therapeutic dose of heparin and LMWH in adults as well as in children. Taken into account the significant decreased anticoagulant potency of sevuparin compared with heparin, the dose of 1.5 mg/kg is considered to have a sufficient safety margin for the intended patient population. This dose was therefore considered justified as a safe starting dose in 18–65 years old adults suffering from acute uncomplicated malaria disease. Subjects were enrolled in cohorts of three in each, and escalation of dose to the next cohort was determined based on evaluation of DLTs in the previous cohort, following a dose escalation strategy. After all three subjects in one cohort had been observed for a minimum of 14 days an evaluation of the dose limiting toxicity (DLT) was done and reported to the Data Safety Monitoring Board (DSMB). Part 2 of the study was open label and with patients randomized in a 1:1 ratio, in blocks of 10 (sequentially numbered containers), to receive either 12 consecutive i.v. infusions (6 hours apart) of sevuparin 3 mg/kg in addition to standard of care, or standard of care alone. This part of the study could only be initiated following a safety evaluation of part 1 data performed by the DSMB. The dose to be used was recommended by the DSMB due to increased, but not above stopping criteria, APTT, and no obvious improved efficacy with higher dose. Study design and dosing regimen of part 1 and part 2 are shown in Fig 2.

The primary endpoint of part 2 was area under the curve (AUC) of the graph plotting the throphozoite stage peripheral blood parasitemia over time of sevuparin treatment as adjunctive to atovaquone/proguanil in comparison to atovaquone/proguanil treatment alone. Relative numbers of ring-, throphozoite- and schizont-IE were calculated from the number of IE at one time point related to the baseline number of IEs at time point 0 h (immediately prior to the first dose of sevuparin).

The total parasitemia and the number of ring-IE, trophozoite-IE and schizont-IE were estimated from blood collected before treatment of sevuparin was initiated (H0) as well as at specified time-points after the first dose, including H1, H2, H3, H4, H6, H8, H10, and H11 thereafter every 6h until two sequential parasite negative slides were collected. Thin and thick films were prepared from 0.5 mL blood at each timepoint. One hundred parasites were counted. The slides were read for determination of numbers of parasites by two experienced independent microscopists at respective hospital and by one expert at Mahidol Oxford Tropical Medicine Research Unit (MORU), Mahidol University, Bangkok, Thailand for assessment of stages of the parasites in the blood [36,37]. Microscopists were blinded with regard to treatment allocation.

For part 2, a sample size of 40 in the sevuparin treated group and 40 in the control group was estimated to allow the detection of a difference of 0.5 log units in terms of the Area Under the Curve (AUC) of the late-stage peripheral blood parasitemia over time curve from log 10^5^ parasites/μL/h to log 3X10^5^ parasites/μL/h between the sevuparin group and the control group, with an expected SD of 0.8 logs in both groups, significance level of 0.05 and a power of 80%. Due to slow recruitment, the study was prematurely stopped when a total of 44 patients were randomized in part 2 (out of 79 screened). Out of these, 21 patients were randomized to treatment with sevuparin and 23 were controls.

Statistical analyses including tables, figures and data listings were produced using SPSS software (version 15.0), STATA (version 13) and R (versions 3.0.1 and 3.1.3). Data were log transformed to obtain a normal distribution, where necessary. Normally distributed data were compared using Student’s t test. AUC was calculated using the trapezoidal rule, differences between groups were assessed using Mann- Whitney tests. Categorical variables such as gender, blood type etc was analyzed using the Fishers exact test. Differences in change from baseline in parasitemia, abundance of early and late stages of parasites were analyzed using the Mann- Whitney test. The Mann-Whitney U test has been used for non-paired nonparametric data.

### Bioanalytical method for studying levels of sevuparin in human plasma

The sevuparin concentrations in the peripheral blood taken during TSM02 study were measured using a validated solid- phase competitive Enzyme-Linked Immunosorbent Assay (ELISA) kit for plasma samples (Lifespan Technologies, Salt Lake City, Utah, USA) according to the manufacturer’s instructions. This assay measures sevuparin directly using a sevuparin/heparin binding protein, conjugated to horseradish peroxidase (HRP). Calibration range for sevuparin was 500–10000 ng/ml. The range of quantitation for this method was 1000–5000 ng/ml, the calibration curve fitted with the 4PL logistic model spanned a concentration range between 500 and 10000 ng/ml. The Lower Limit of Quantification (LLOQ) for sevuparin was 1000 ng/ml and the Upper Limit of Quantification (ULOQ) was 5000 ng/ml.

## *In vitro* experiments—Invasion inhibition

Trophozoite IE were placed at a haematocrit of 5% in 75 μL of culture medium with 5% human AB-serum in 96 well round-bottom microtiter plates (Falcon) with the addition of 25 μL sevuparin (final dilutions: 1000 to 0.125 μg/mL in triplicates) or 25 μL phosphate buffered saline (control).

Laboratory strains were grown for one cycle while patient isolates were grown for two, after which the cultures were stained with acridine orange and the parasitemia of each well was determined by flow cytometry using a FACSVerse, BD Bioscience with a plate reader. Data were analyzed using FlowJo software. Invasion inhibition was expressed as a percentage of the parasitemia in absence of sevuparin with PBS only. Parasite strains used were 10 lab strains (3D7, 3D7PG12, FCR3S1.2, Dd2, R29, TM180, TM284, F32, 7G8, HB3), 11 isolates from Ugandan patients (six from severe cases, five from uncomplicated cases), two from Ethiopia/Eritrea, three from Kenya, one from Niger all of travellers with uncomplicated *falciparum* malaria diagnosed at Karolinska University Hospital, Sweden and established at Karolinska Institutet; three EBA-knock out strains (ΔEBA140; ΔEBA175, ΔEBA181) where the gift of Dr. Alan Cowman, four Cambodian isolates were from ATCC-MR4, Manassas, VA, USA (IPC-4884, Pursut, artemisinin resistant (RSA 0-3h: 6,5%); IPC 5188 Rattanakiri; IPC 3663 Pailin; IPC 4912 artemisinin resistant (RSA 0–3 h: 49%)). *P*. *falciparum* laboratory strains were cultivated according to standard methods [38]. Patient isolates were collected in Kampala, Uganda [39], or in Stockholm, Sweden, and establishment of cultures was carried out as described before [40]. The Ethics committees of the Faculty of Medicine, Makerere University, the Uganda National Council of Science and Technology and the Karolinska Institutet approved the study.

## Results

### Phase I study in healthy volunteers

In a phase I study conducted in healthy male volunteers we found sevuparin to be safe and well tolerated at the dose levels studied, the data of which are to be found in S2 Supportive Information.

### Sevuparin in humans with *P*. *falciparum* malaria

**Fig 3:**
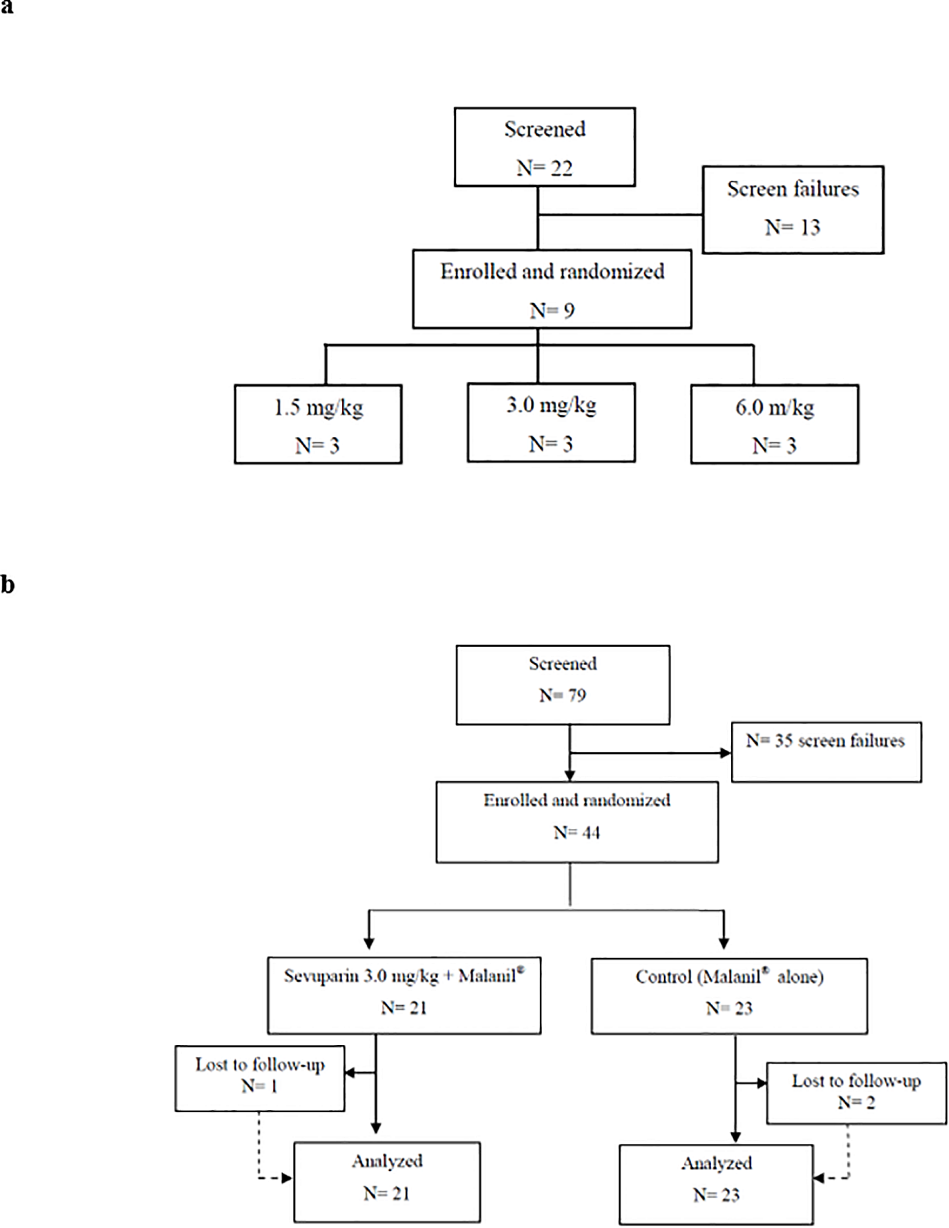
CONSORT flow diagram. **Flow diagram of the progress through the phases of TSM02 randomized trial with two groups for Part and Part 2.** a, Part 1 enrolment, intervention allocation, follow-up, and data analysis. b, Part 2 enrolment, intervention allocation, follow-up, and data analysis.

To investigate the primary pharmacodynamics of sevuparin in patients with uncomplicated *P*. *falciparum* malaria, we conducted an exploratory study, clinical phase I/II open-label trial (TSM02, Fig 2). A total of 53 patients were included in the trial and were treated with oral atovaquone/proguanil (Malanil®), with or without adjunctive treatment in the form of short i.v. infusions of sevuparin (Fig 3). The baseline clinical parameters (from Day 01 Hour 0) of the two groups of patients were similar and are summarized in Table 2.

**Table 2:** Baseline characteristics of patients with uncomplicated *P*. *falciparum* malaria enrolled in the Phase I/II study of sevuparin (TSM02)*.

		Controls	Sevuparin	Total	
		(N = 23)	(N = 21)	(N = 44)	p-value
**Age**	Mean (SD)	30.95 (9.78)	29.19 (8.08)	30.11 (8.95)	0.520
	Median	34.00	27.00	28.88	
	Range	18 to 51	19 to 42	18 to 51	
**Sex, ratio female:male**	Ratio	01:22	04:17	05:39	0.176
**Body temperature (°C)**	Mean (SD)	37.43 (0.99)	37.53 (0.98)	37.48 (0.97)	0.689
	Median	37	37.6	37.25	
	Range	35.8 to 39.9	36 to 39.9	35.8 to 39.9	
Serum lactate dehydrogenase (U/L)**	Mean (SD)	175.74 (52.43)	170.48 (56.33)	173.23 (53.75)	0.681
	Median	188	159	161.5	
	Range	91 to 283	90 to 310	90 to 310	
Serum glucose (mg/dL)***	Mean (SD)	126.50 (35.26)	129.86 (31.45)	128.14 (33.10)	0.552
	Median	119	128	121	
	Range	75 to 215	76 to 195	75 to 215	
**ABO blood group**					0.308
**A**	N (%)	8 (34.78)	9 (42.86)	17 (38.64)	
**AB**	N (%)	2 (8.70)	0 (0.00)	2 (4.55)	
**B**	N (%)	5 (21.74)	8 (38.10)	13 (29.55)	
**O**	N (%)	8 (34.78)	4 (19.05)	12 (27.27)	
**Parasitemia**	Mean (SD)	42107.04 (38719.56)	36305.48 (35464.94)	39338.11 (36886.76)	0.499
	Median	24390	20161	20629.5	
	Range	4956–121240	350–99750	350–121240	
**Parasite stages (%): trophozoites, schizonts**	Mean (SD)	31.96 (35.67)	28.33 (29.17)	30.23 (32.41)	0.981
	Median	18	26	19.0	
	Range	0–100	0–97	0–100	
**Parasite stages (%): rings**	Mean (SD)	68.00 (35.68)	71.57 (29.14)	69.70 (32.39)	0.999
	Median	82	74	80.5	
	Range	0–100	3–100	0–100

*Patients were treated with oral atovaquone/proguanil, without (controls) or with sevuparin (sevuparin) as adjunctive treatment in the form of short i.v. infusions. For details of the study (TSM02) please see Methods.

** (normal range: 114–240 U/L).

*** (normal range: 70–110).

The first part of the trial was a dose-escalation component with the aim to determine the dose for the second part, the efficacy evaluation. The first part included 9 patients who were administered 12 doses of sevuparin at 1.5 mg/kg, 3.0 mg/kg or 6 mg/kg every 6 h for three days (three patients/ dose level). After the dose escalating part (part 1) safety data were reviewed by a data safety monitoring board (DSMB). Pre-defined stopping criteria included individual APTT ≥1.5 x ULN 5 hours after dose 1 or dose 5, mean APTT in one cohort ≥ 3 x ULN 2 hours after dose 1 or dose 5, mean APTT in one cohort close to, or ≥3 x ULN 2 hours after dose 12, or at the Investigator’s discretion, but had not been reached in any dose group. However, as the dose 6.0 mg/kg 6 q6h did give higher APTT values than the lower doses and in one patient APTT was up to 120 seconds 1 hour after the dose (ULN = 33 sec), the DSMB considered the risk of adverse events was potentially increased and recommended that the study should continue to part 2 with 3.0 mg/kg sevuparin q6h. Forty-four patients were enrolled in the second part of this 1:1 randomized and open-label study, 21 were given i.v. sevuparin infusions in addition to the oral atovaquone/proguanil, and 23 patients were only given oral atovaquone/proguanil. All 53 subjects included in the trial completed the study treatment.

#### Safety

There were minimal and clinically non-relevant changes in anti-Xa- and prothrombin-times, and the international normalized ratios (INR) associated to the sevuparin dose (S1 and S2 Tables). Increases in APTT were seen to be dose-dependent and appeared to follow the time-concentration curve for each sevuparin infusion. The 6 hour interval between the infusions allowed for nearly full reversibility of APTT levels after each dose, and no accumulative effects were seen over the course of the 12 consecutive infusions (S3 and S4 Tables). Thrombocytopenia was observed in one subject (3 mg/kg) but it was also present before the initiation of sevuparin treatment (S5 Table). There was no incident of bleeding in any of the subjects.

No severe adverse event (SAE) or adverse event (AE) leading to withdrawal occurred in the study (S5 and S6 Tables). In the first dose-escalating part, no dose-dependent increase in probability for relation between event and sevuparin treatment was seen. In part 2, the AE rate was higher in the sevuparin group (average 2.4 AEs/subject) as compared to the control group (average 1.3 events/subject). A relatively high frequency of abnormal liver transaminases and high bilirubin levels at baseline was observed and was likely due to the malaria infection and the antimalarial treatment with atovaquone/proguanil [41,42]. Increases above the ULN in liver transaminases (ALT and AST) on Day 7 were seen in all dose groups in part 1 and in both treatment groups in part 2. In part 2, the increases were more pronounced in the sevuparin treated subjects. However, it is notable that the increase in liver transaminase seen after sevuparin and atovaquone/proguanil administration in malaria infected patients seemed not to be more pronounced than what was demonstrated from heparin and LMWH administration in healthy volunteers in a study presented by Harril el al, 2012 [41].

There were no apparent changes from baseline or differences between dose groups with regard to physical examination findings and vital signs. There were no apparent changes in ECG or QTc in sevuparin treated subjects. To summarize, sevuparin administered as i.v. infusion for five minutes every six hours for three consecutive days (a total of 12 infusions) at different dose levels was safe and well tolerated in adult patients with uncomplicated *falciparum* malaria.

#### Efficacy

**Fig 4:**
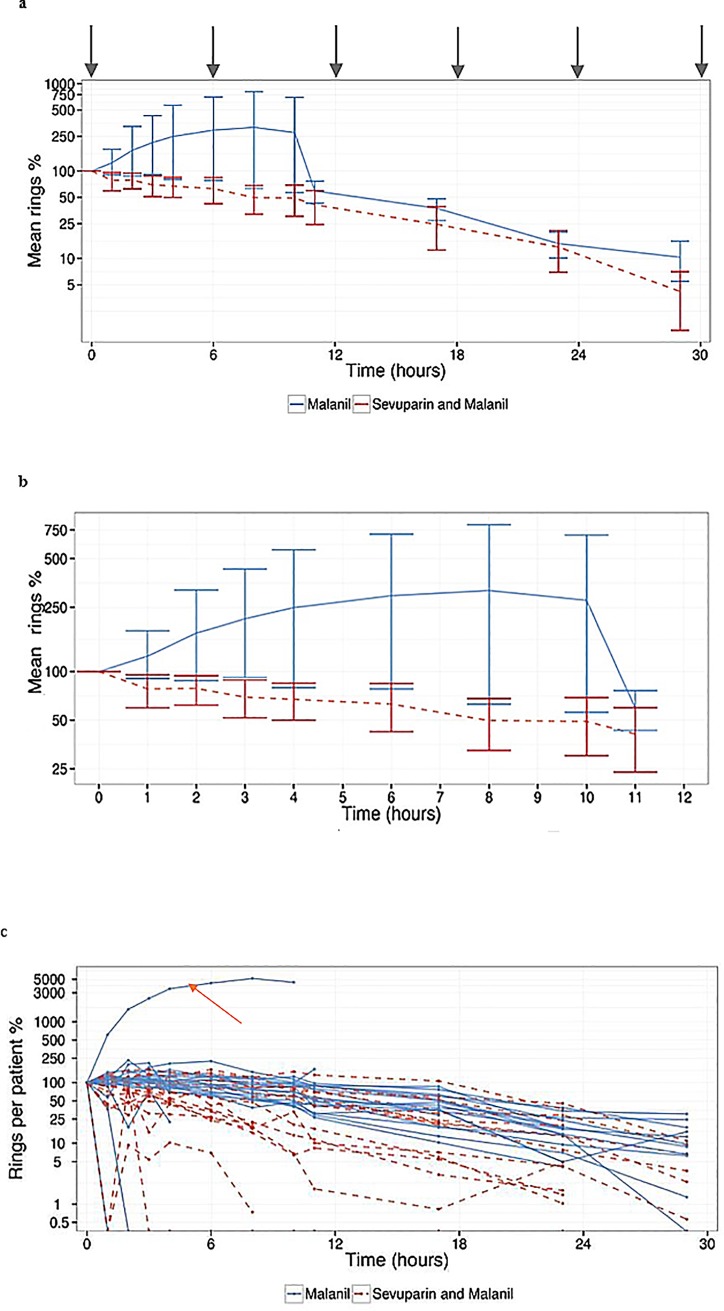
Sevuparin lowers the relative mean number of ring-stage IEs after a single sevuparin infusion in *P*. *falciparum* infected patients. A total of 44 patients were included in the efficacy part of the trial (part 2) and were treated with oral atovaquone/proguanil with or without adjunctive treatment in the form of i.v. infusions of sevuparin. The relative numbers were calculated from the number of ring IEs at one time point related to the baseline value of ring IEs at time point 0 h (immediately prior to the first dose of sevuparin), and the mean was measured based on all subjects in one group. a, The mean relative numbers (mean ± SD) of ring stage parasites in the two study groups from 0 h to 30 h. The numbers of ring-stage IEs were estimated in peripheral blood samples on thin and thick films that were taken at time points 0, 1, 2, 3, 4, 6, 8, 10, and 11 h and thereafter every 6 h until two consecutive blood samples were parasite negative. The red dotted line represents the patients treated with sevuparin (3 mg/kg) and oral atovaquone/proguanil, and the blue line represents the control patients who were given only oral atovaquone/proguanil, a logarithmic y-axis is used. Significantly lower levels of ring stage IEs were found in the sevuparin treated patients at time points 1 h (p = 0.0223), 2 h (p = 0.0246), 3 h (p = 0.0027), 4 h (p = 0.0278), and 6 h (p = 0.0346). (An outlier appears in the data but does not drive the difference as the statistical significant difference between the two groups remains even if data from this patient is excluded from the analysis since the tests used are non-parametric which are thus very robust against divergent.) b, Detailed mean relative changes in the number (mean ± SD) of ring stage parasites during the first 12 hours after the first injection of sevuparin. c, Numbers of ring-stage IEs levels in the individual patients. Oragne arrow indicate an outlier. Grey arrows indicate the short i.v. sevuparin infusions over five minutes.

Efficacy of treatment with sevuparin in *P*. *falciparum* infected patients was determined as change in numbers of mature throphozoit-IE, and number of ring-IE in the peripheral blood, as compared with control patients. The stages of the parasites in the peripheral blood and the total parasitemia were scored by microscopy. Sevuparin treatment was anticipated to both increase the number of circulating trophozoite/schizont IEs in the peripheral bllod, which would mean that sevuparin cause de-sequestration of previously bound/sequestered trophozoite/schizont IEs [18,19,21–26] and to reduce the number of ring-stage IEs because sevuparin blocks the invasion of merozoites into erythrocytes [17,20,25]. In the control patients not treated with sevuparin trophozoite/schizont IEs continue to be sequestered and are not detected in the peripheral blood and merozoites continue to invade erythrocytes leading to an increase in ring-IE. Following the initial 5 min infusion of sevuparin, an immediate effect was observed on the ring-stage IEs: the relative mean numbers decreased consistently for six hours (Fig 4A–4C). In contrast, the parasites continued to expand in the controls who were given only oral atovaquone/proguanil (Fig 4A–4C). The mean percentage of ring-stage IEs decreased by 50% after 8 hours of the combined sevuparin and atovaquone/proguanil treatment, whereas it took more than 13 hours for the patients in the control group to reach this level. Significant differences between the groups, as calculated by the Mann–Whitney U test, were observed at one hour (H1, p = 0.022), two hours (H2, p = 0.025), three hours (H3, p = 0.003), four hours (H4, p = 0.028) and six hours (H6, p = 0.035) (Fig 4A–4C). The largest mean difference occurred at three hours after the first infusion (H3, p<0.005). Furthermore, the relative mean number of ring-stage IEs was also lower during the rest of the study period in the sevuparin-treated group compared to the control group, but the difference was not significant, likely due to the effect of atovaquone/proguanil on the parasites in both groups of patients (Fig 4A–4C). There was one clear outlier in the atovaquone/proguanil control group (Fig 4C), but the significant differences in between the groups were also present when this patient was excluded from the analysis (p<0.05, H1, H2, H3, H4, H6). This because the tests we use are non-parametric. In these tests, the only parameter that matters is an observation rank and not its absolute value. The tests are thus very robust against divergent observations. Taken together, we show that impaired merozoite invasion in humans with *P*. *falciparum* malaria in the presence of sevuparin leads to fewer ring stage parasites in the peripheral blood.

**Fig 5:**
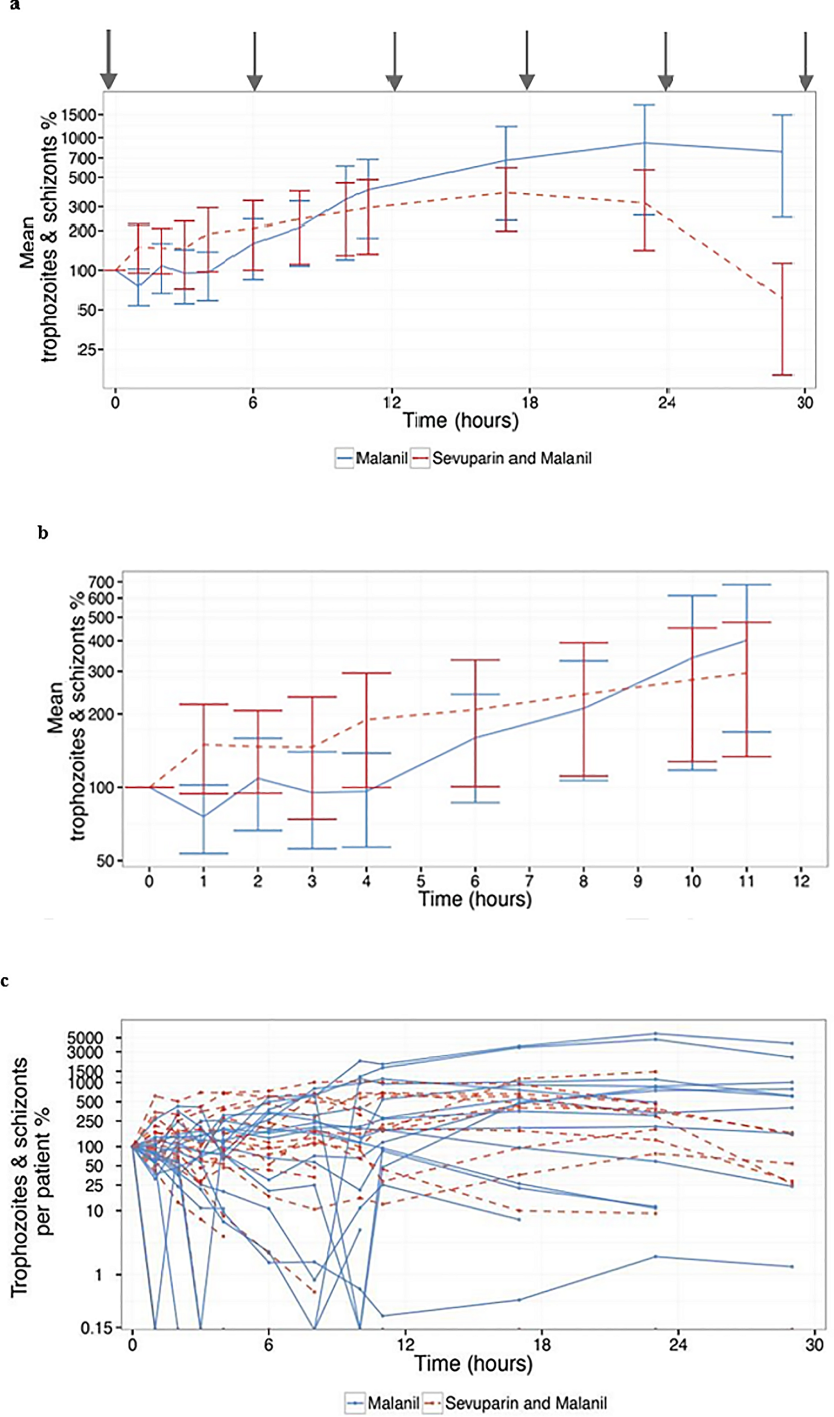
The de-sequestering capacity of sevuparin in *P*. *falciparum* infected patients. A total of 44 patients were included in the efficacy part of the trial and were treated with oral atovaquone/proguanil with or without adjunctive treatment in the form of short i.v. infusions of sevuparin. The numbers of trophozoite and schizont IEs were estimated in the peripheral blood samples on thin and thick films that were taken at time points 0, 1, 2, 3, 4, 6, 8, 10, and 11 h and thereafter every 6 h until two consecutive blood samples were parasite negative. The relative numbers were calculated from the number of trophozoite and schizont IEs at one time point related to the baseline number of trophozoite and schizont IEs at time point 0 h (immediately prior to the first dose of sevuparin), and the mean was measured based on all subjects in one group. The red dotted line represents the sevuparin treated patients, and the blue line represents the control patients. Logarithmic y- axis is used. Significantly (p<0.05) higher numbers of trophozoite and schizont IEs were found in the sevuparin treated patients at time point 1 h (p = 0.0322). In a, the relative numbers (mean ± SD) of trophozoite and schizont parasites in the two study groups up to H30 are presented. In b, the detailed relative changes in the number of trophozoite and schizont parasites (mean ± SD) during the first 12 hours after the first injection of sevuparin are shown. In c, individual effects of sevuparin on the number of trophozoites and schizont parasites per patient is shown. The grey arrows indicate the periodic sevuparin infusions.

The two patient groups in the clinical phase I/II open-label trial were also analysed in terms of de-sequestration, which is the increase of mature parasite IEs in the circulating blood after the initiation of treatment with sevuparin. Notably, more trophozoite and schizont IEs appeared in the circulating peripheral blood one hour after the first dose of sevuparin than in the controls who were not given sevuparin (H1, p = 0.031; Fig 5A–5C). The relative mean number of mature- parasite IEs was also increased at time points H2-H8 in the sevuparin treated group compared to the controls, but the result was not significant (Fig 5A–5C). Mature-parasite IEs continued to an increase in the control group after H9, most likely due to the postponed effect of atovaquone/proguanil and a recruitment of IE that was blocked in the sevuparin treated group. In conclusion, we present results that support that sevuparin de-sequesters mature IEs in humans already present one hour after an infusion.

The primary endpoint was to compare the difference in the number of mature-IEs in circulating blood between the two treatment groups as the cumulative area under the curve (AUC) for the first 11 hours after the initiation of sevuparin. In the sevuparin group, the AUC of late stage parasitemia was expected to increase over time as compared to the control group. No statistically significant difference between treatment groups was detected as regards cumulative AUC of late stage peripheral blood parasitemia at the time point 11 hours after first dose of sevuparin (S7 Table). Other time-points evaluated were 1, 2, 3, 4 and 17 hours post first dose (S7 Table). Similarly, no statistically significant difference was detected at the other time points tested (1, 2, 3, 4 and 17 hours post dose) (S7 Table). The study failed to meet its primary end-point at 11h likely due to the unexpected rapid onset of sevuparin. It is important to stress that the study presented is exploratory and prior to its execution we did not anticipate the important effect on merozoite invasion that by itself leads to fewer mature-IE in the sevuparin treated group, a counter-effect on the clinical endpoint.

The median parasite clearance time was evaluated in part 2 and was 53 hours (min:max, 41:65 hours) in the sevuparin group, versus 59 hours (min:max, 53:65 hours) in the control group (p = 0.720). The median parasite reduction rates (PRRs) at 24 and 48 hours were respectively 87.44% (min:max, -7.69:100) and 99.96% (min:max, 98.61:100%) in the sevuparin group, versus 84.64% (min:max, 18.08:100) and 99.78% (min:max, 94.18:100) in the control group, both not significantly different (p = 0.391 and p = 0.054, respectively). The median time to 50% reduction of peripheral blood parasitemia was 12.66 hours (min:max, 0.77:27.39 hours) in the sevuparin group, versus 12.87 hours (min:max, 1.06:31.45 hours) in the control group (p = 0.404), and the median time to 90% reduction was in the sevuparin group 25.59 hours (min:max, 2.63:37.02 hours) versus 30.71 hours (min:max, 4.39:44.47 hours) in the control group (p = 0.148). (S8 Table).

#### Pharmacokinetics

In patients with uncomplicated malaria treated with sevuparin, systemic exposure in terms of Cmax and AUC increased in a dose-proportional manner. Mean Cmax in part 1 and part 2 were between 56.7–149 μg/mL. In both parts and both after the first and last dose, plasma levels declined mono-compartmentally with an apparent terminal half-life of 0.7–1.3 hours. Systemic exposure in terms of Cmax and AUC did not show any notable differences on Day 3 vs. Day 1 at any dose level. Overall between-patient variability in terms of systemic exposure parameters was moderate and tended to decrease with increasing dose.

### Sevuparin and merozoite invasion *in vitro*

**Fig 6:**
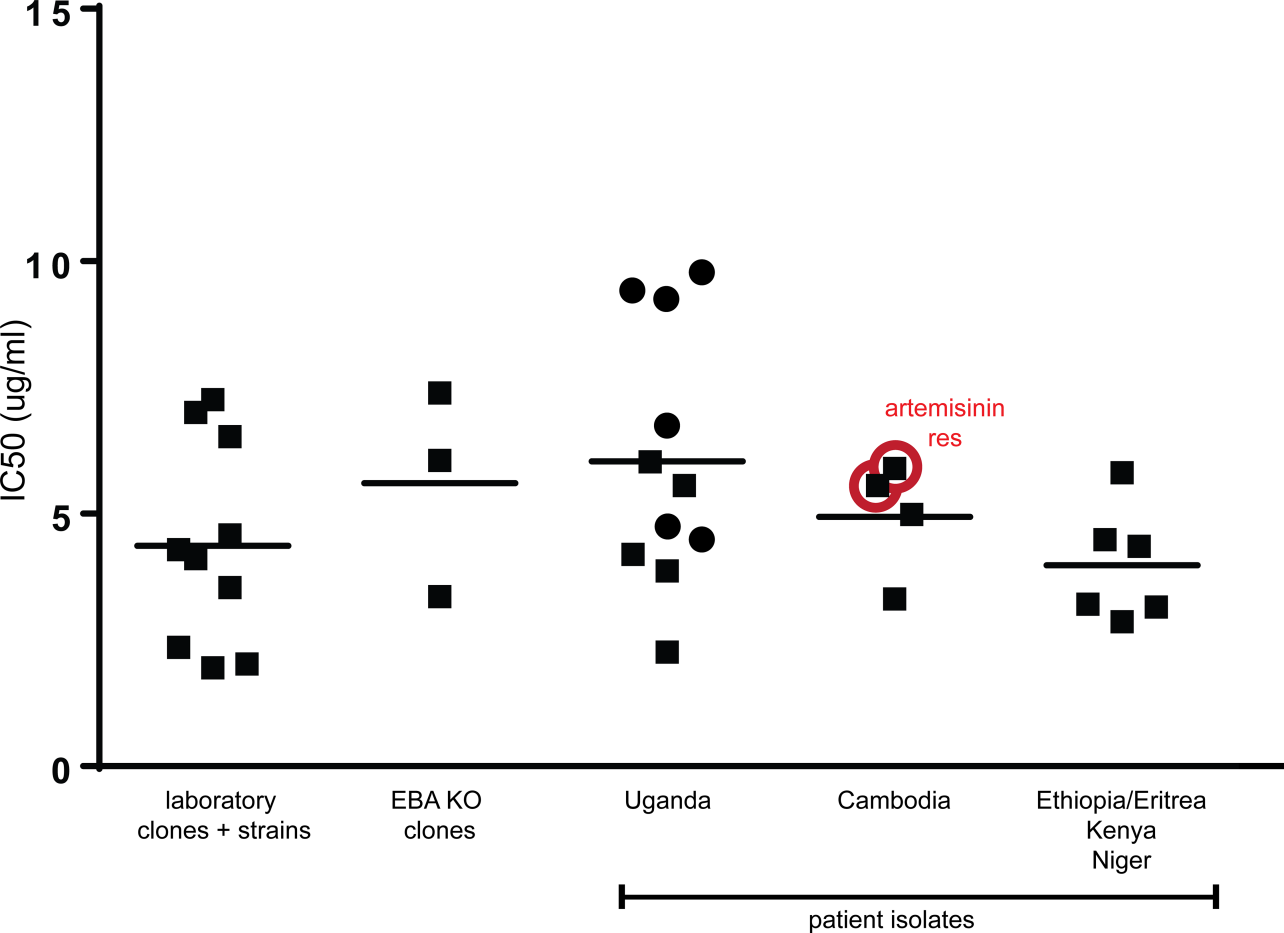
Sevuparin inhibits merozoite invasion of *P*. *falciparum* clones, strains and fresh isolates *in vitro* at low concentrations, independently of parasite origin or phenotype. The invasion blocking capacity of sevuparin in 34 *in vitro* propagated *P*. *falciparum* isolates expressed as IC50. The inhibitory capacity of sevuparin was titrated in double dilution steps between 0.125 μg/mL and 1 mg sevuparin/mL culture. Ten laboratory isolates were either sensitive (3D7, 3D7PG12, Dd2, HB3) or resistant (R29, TM180, TM284, F32, 7G8, FCR3S1.2) to chloroquine. Three parasites of the W2mef background carried disrupted genes for EBA 140, EBA 175 or EBA 181 (EBA-KO). W2mef is a cloned line of parasites derived from the Indochina III-CDC strain. Of the fresh primary isolates 11 were from Ugandan children with either severe (dot) or uncomplicated (square) malaria and six isolates were from adults infected in Ethiopia/Eritrea, Kenya or Niger. Four Cambodian isolates were sensitive or resistant to artemisinin (red-circled square; IPC-4884, Pursut, artemisinin resistant (RSA 0-3h: 6,5%) and IPC 4912 artemisinin resistant (red circled square; RSA 0–3 h: 49%). ICP 5188 Rattanakiri and IPC 3663 Pailin were artemisinin sensitive (square).

The rapid reduction in ring stage-IEs observed in the sevuparin treated subjects is likely a consequence of the capacity of sevuparin to block the earliest steps of merozoite invasion into erythrocytes [20,25,43–48]. The effect of sevuparin was further studied *in vitro* with 34 parasites from various geographical origins, including 17 primary patient isolates from Ethiopia/Eritrea, Kenya, Niger or Uganda, four *in vitro* established parasites from Cambodia that were resistant or sensitive to artemisinin, three parasites that were deficient in invasion ligands (EBA 140, EBA 175 or EBA 181 gene knock-out W2mef parasites) and 10 *in vitro*, long-term propagated parasites that were sensitive (HB3, DD2, 3D7AH1, 3D7P2G12) or resistant to chloroquine and/or mefoloquine (FCR3S1.2, F32, IT-R29, 7G8, TM180, TM284) [19]. Sevuparin completely inhibited merozoite invasion of all isolates at low concentrations, independently of phenotype (IC50, mean = 5,2 μg/mL; Fig 6).

## Discussion

Most deaths in severe malaria occur within the first day of hospitalization when the characteristic features of high parasite loads and obstructed micro-vasculatures are present [12–14]. It is therefore crucial for the physician to be able to stop further merozoite invasion and “unstick” the infected cells, thereby arresting the infection and restoring blood-flow without delay [9,48]. Based on the rapid pharmacodynamic effects of sevuparin seen already at one hour we argue that it could constitute an important adjunctive treatment of severe malaria. Specifically, sevuparin has the potential to hinder parasite invasion outside the red blood cell and thus multiplication and expansion of the infection, including that of artemisinin-, chloroquine, and mefloquine resistant parasites, further it also transiently de-sequesters IE. Although the indication of sevuparin is severe malaria a Phase I/II study was conducted in patients with mild disease before entering patients with severe disease since the drug was novel and had previously not been tried in malaria-infected individuals. Further, atovaquone/ proguanil was chosen as anti-parasite treatment in order to easily visualize the effects of sevuparin given that it is a relatively slow acting drug-combination as compared to artesunate.

Adhesive IE involved in causing severe malaria are unlike those of uncomplicated disease in that they are prone to bind excessively to the vascular endothelium and to form rosettes [48], processes that are particularly sensitive to sevuparin, and the effects could therefore be superior in patients with severe as compared to those with uncomplicated malaria.

In the sevuparin-treated individuals the mean ring-stage parasitemia decreased with 50% by 8 hours while it took another ≈5 hours for the group treated with solely atovaquone/proguanil (Fig 4A). The significant effects of sevuparin were detected during the first six hours after the study commenced but the mean ring-stage parasitemia was also lower in the sevuparin-treated group throughout the study. However, the late differences were not significant, likely due to the anti-parasitocidal effects of atovaquone/proguanil that start to have an influence on the parasites in both groups of patients at this time. The very first effects of artesunate are seen at four hours post-invasion, thus merozoites and very early ring-stage IE are refractory to artesunate during the first hours of hospitalization and it takes longer to obtain the full effect. Even so, the surprisingly rapid effects of sevuparin described are at place already before those of both atovaquone/ proguanil and artesunate [49] and warrant assessment of its potential as an adjunct to current treatment regimens for severe and complicated malaria.

## Supporting information

S1 Supportive Information CONSORT Checklist resub. (DOC)

S2 Supportive Information CSRS_TSM01. (PDF)

S3 Supportive Information CSP_TSM02_V4_20120414. (PDF)

S4 Supportive Information TSM02Part1_Listings_20140501. (PDF)

S5 Supportive Information TSM02Part2_Listings_20140501. (PDF)

S1 Fig Individual total parasitemias and ring-, trophozoite- and schizont- stage IE, over the complete treatment period in *P*. *falciparum* patients treated with oral Malanil® (atovaquone/proguanil) or treated with sevuparin and oral Malanil® (atovaquone/proguanil). (PDF)

S1 Table Shift table of Anti Xa in patients with uncomplicated malaria treated with sevuparin. (DOCX)

S2 Table Summary of PT (seconds) and INR in patient with uncomplicated malaria receiving 1.5, 3, and 6 mg/kg sevuparin in part 1 and 3 mg/kg sevuparin in part 2. (DOCX)

S3 Table APTT (seconds) at 1, 2 and 5 hours following sevuparin dose number 1 and 12, part 1. (DOCX)

S4 Table APTT (seconds) over time following sevuparin treatment in patients with uncomplicated malaria, part 2. (DOCX)

S5 Table Summary of AEs reported in part 1 of study in patients with uncomplicated malaria treated with multiple doses sevuparin. (DOCX)

S6 Table Summary of AEs reported in part 2 of study in patients with uncomplicated malaria, treated with multiple doses of sevuparin. (DOCX)

S7 Table Cumulative AUC of late stage peripheral blood parasitemia (trophozoites and schizonts only) at 11 hours (primary endpoint) and at 1, 2, 3, 4 and 17 hours (secondary endpoints) after first dose of sevuparin. Part 2.
(DOCX)

S8 Table Median (range) parasite clearance outcomes in the two groups in part 2 of study in patients with uncomplicated malaria. (DOCX)
