# Supplementary material for: Inhibition of merozoite invasion and transient de-sequestration by sevuparin in humans with Plasmodium falciparum malaria
Source: PLoS One. 2017 Dec 15;12(12):e0188754. doi: 10.1371/journal.pone.0188754 (PMC5731734; doi:10.1371/journal.pone.0188754)
Supplement: S2 Supportive Information — (PDF) [file pone.0188754.s002.pdf]

Clinical Study Report

1. TITLE PAGE

**CLINICAL STUDY REPORT**	
**Report version**: Final 1.0	**Report date**: 13 January 2010
A Single Centre, Double Blind, Randomised, Placebo Controlled, Phase I Study to Evaluate the Safety, Tolerability, Pharmacokinetics and Pharmacodynamics of the Depolymerised Heparin Compound DF02 Following Single and Multiple Intravenous Doses in Healthy Male Volunteers	
**Sponsor:**	Dilafor AB<br>Nobels väg 3<br>SE 171 77 Stockholm<br>Sweden
**Investigational medicinal product:**	DF02
**Clinical trial protocol number:**	TSM01
**PAREXEL number**	PXL98220
**EudraCT number:**	2009-009616-41
**Drug development phase:**	Phase I
**Indication:**	Malaria
**Principal Investigator:**	Dr John Lambert<br>PAREXEL International Early Phase Clinical Unit<br>Level 7, Northwick Park Hospital<br>Harrow, HA1 3UJ, London<br>United Kingdom<br>Tel: +44 (0) 189 561 4865<br>Fax: +44 (0) 208 422 6070
**Study duration:**	14APR2009 (first subject first visit) to<br>11AUG2009 (last subject last visit)

This study was conducted in compliance with International Conference on Harmonisation Good Clinical Practice guidelines and the Declaration of Helsinki. The essential documentation related to this study has been retained by relevant parties.

Confidentiality Statement

This confidential document is the property of Dilafor AB. No unpublished information contained herein may be disclosed without prior written approval from Dilafor AB. Access to this document must be restricted to relevant parties.

SIGNATURE PAGE

Study Title: A single centre, double-blind, randomised, placebo-controlled, Phase I study to evaluate the safety, tolerability, pharmacokinetics and pharmacodynamics of the depolymerised heparin compound DF02 following single and multiple intravenous doses in healthy male volunteers

PAREXEL STUDY No.: PXL98220

SPONSOR STUDY No.: TSM01

I have read this report and confirm that, to the best of my knowledge, it accurately describes the conduct and results of this study.

Anders Åsell<br>Chief Executive Officer<br>Dilafor AB	Date
Gunvor Ekman Ordeberg<br>Professor in Gynaecology and Obstetrics<br>Karolinska University Hospital	Date
Dr John Lambert<br>Principal Investigator<br>PAREXEL International EPCU	Date

2. SYNOPSIS

Name of Sponsor/Company:	Individual Study Table Referring to Part of the Dossier	(For National Authority Use Only)
Name of Finished Product:	Volume:	
Name of Active Ingredient:	Page:	

**Title of Study:**	A single centre, double-blind, randomised, placebo-controlled, Phase I study to evaluate the safety, tolerability, pharmacokinetics and pharmacodynamics of the depolymerised heparin compound DF02 following single and multiple intravenous doses in healthy male volunteers
**Principal Investigator:**	Dr John Lambert
**Study Centre:**	PAREXEL International Early Phase Clinical (EPCU), Level 7, Northwick Park Hospital, London United Kingdom (UK)
**Publication:**	Not applicable at the time of this report.
**Studied Period:**	First subject first visit to 14APR2009 to<br>Last subject last visit: 11AUG2009
**Development Phase:**	Safety, tolerability, pharmacokinetics and pharmacodynamics

Study Objectives:

Primary Objectives

Part 1:

- To explore the safety and tolerability of DF02 following intravenous (IV) administration of single ascending doses (SAD) and to estimate the maximum tolerated dose (MTD), if within the predefined exposure and dose limits.

Part 2:

- To explore the safety and tolerability of DF02 following IV administration of multiple ascending doses (four times in 24-hour period) (MAD).

Secondary Objectives

Part 1:

- To determine the single dose pharmacokinetics (PK) of DF02 following IV administration of SAD (including assessment of dose proportionality and linearity).
- To determine the single dose pharmacodynamics (PD) of DF02 following IV administration of SAD (by measurement of activated partial thromboplastin time [aPTT], prothrombin time [PT], bleeding time and platelet function).
- To study the time to normalisation of aPTT.

Part 2:

- To determine the multiple dose PK of DF02 following IV administration of MAD (four times in 24-hour period).
- To determine the multiple dose PD of DF02 following IV administration of MAD (four times in 24-hour period) (by measurement of aPTT, PT, bleeding time and platelet function).
- To study possible accumulation of aPTT and plasma concentrations between Dose 1 and Dose 9.

Name of Sponsor/Company:	Individual Study Table Referring to Part of the Dossier	(For National Authority Use Only)
Name of Finished Product:	Volume:	
Name of Active Ingredient:	Page:	

- To study the time to normalisation of aPTT for Dose 1 and Dose 9.

Methodology:

This was a first-in-human study in two parts, conducted at a single study centre in the UK.

Part 1 employed a double-blind, randomised, placebo-controlled, parallel group, SAD design. This part of the study consisted of a Screening Visit, a Treatment Phase comprising three treatment periods (with a wash-out period of at least 12 days between treatments [*within* a cohort]), and a Follow-up Visit. Eligible subjects were included in one of two cohorts and randomised to active treatment (DF02) or placebo. Subjects in Cohort 1 were to receive doses of 10, 90 and 360 mg of DF02/placebo in Treatment Periods 1, 2 and 3, respectively. Subjects in Cohort 2 were to receive doses of 30, 180 and 480 mg of DF02/placebo in Treatment Periods 1, 2 and 3, respectively. All doses of investigational product were to be administered as a single IV infusion in the morning of Day 1 in each treatment period. The decision to escalate to each higher dose was based on a review of blinded interim safety data and PD data up to 24 hours post-dose. Subjects were to attend a Follow-up Visit 7 to 14 days after administration of the last dose of investigational product.

Part 2 of the study employed a double-blind, randomised, placebo-controlled, multiple dose ascending (MAD) design. This part of the study consisted of a Screening Visit, a Treatment Phase comprising a single treatment period, and a Follow-up Visit. Part 2 of the study was only initiated after the MTD dose was determined in Part 1. As in Part 1, subjects were assigned to one of two cohorts. Subjects were to be dosed starting at a dose two steps below the MTD determined in Part 1. Subjects were to receive nine identical doses of the active treatment/placebo, administered 6 hours apart (four doses in 24 hours). Doses 1 to 3 were to be administered on Day 1, Doses 4 to 7 on Day 2, and Doses 8 and 9 on Day 3. The first cohort treated (Cohort 1) were to receive a lower dose level than Cohort 2. The first dose level was decided after review of safety and PD data from study Part 1. The second dose level (Cohort 2) was decided after a review of Part 2, Cohort 1 data. Subjects were to attend a Follow-up Visit 7 to 14 days after administration of the last dose of investigational product.

Study Subjects:

Planned for completion:	It was planned to include 16 subjects in each part of the clinical study; that is, 32 subjects in total.<br>Part 1 and Part 2 of the study were to each comprise two cohorts of 8 subjects randomised to DF02 (n=6) or placebo (n=2) in each cohort. Additional subjects were allowed to be enrolled where replacements were required.
Enrolled and randomised:	Seventeen (17) subjects were enrolled and randomised to treatment in Part 1 of the study, while 16 subjects were enrolled and randomised to treatment in Part 2 of the study.
Excluded:	Six (6) subjects in Part 2 were excluded from the PKS.
Analysed:	Part 1: all 17 randomised subjects were included in the ASTS, PDS and PKS.<br>Note: Subject 113 (Cohort 2, Part 1) was included in the ASTS for 420 mg dose, however this subject received 480 mg in Period 3 of Part 1.<br>Part 2: 16 subjects were included in the ASTS and PDS and 12 subjects were included in the PKS.

Name of Sponsor/Company:	Individual Study Table Referring to Part of the Dossier	(For National Authority Use Only)
Name of Finished Product:	Volume:	
Name of Active Ingredient:	Page:	

Diagnosis and Main Criteria for Inclusion:

Subjects were to provide written informed consent to participate before any study procedures were performed. Subjects were to be male, between the ages of 18 and 55 years (inclusive), with a minimum body weight of 50 kg, a body mass index (BMI) between 19 and 29.9 kg/m^2 (inclusive), and haemostasis values (aPTT, PT and platelet count) within the normal laboratory range. Appropriate methods of contraception were to be used from first dosing until at least 3 months after receiving the last dose of investigational product.

Test Product, Dose and Mode of Administration, Batch Number:

Test product: DF02

Batch number: 2434247 (Expiry date: June 2009)

Mode of administration: Intravenous infusion over 5 minutes (maximum 7 minutes)

Dose: Planned doses in Part 1 were as follows:

Treatment Period	**Cohort 1**	**Cohort 2**
**1**	10 mg DF02 or placebo	30 mg DF02 or placebo
**2**	90 mg DF02 or placebo	180 mg DF02 or placebo
**3**	360 mg DF02 or placebo	480 mg DF02 or placebo*

*One subject each received 480 mg DF02 and placebo as planned, remaining subjects received 420 mg DF02 or placebo.

Doses chosen for Part 2 were based on results observed in Part 1. Doses in Part 2 were as follows:

Cohort 1: 9 identical doses of 180 mg DF02/placebo

Cohort 2: 9 identical doses of 360 mg DF02/placebo

Duration of Treatment:

Part 1: Subjects attended three treatment periods, each separated by a wash-out period of at least 12 days.

Part 2: Subjects received study medication over a period of three days, with a 6-hour period observed between doses.

Reference Product, Dose and Mode of Administration, Batch Number:

Reference product: Placebo

Batch number:

Batch Number	Expiry Date	Batch Number	Expiry Date
09A23E2U	DEC2011	09C30E8Z	FEB2012
09B10E42	JAN2012	09D16E6K	MAR2012
09B20E4U	JAN2012	09E27E2H	APR2012
09B05E4Z	JAN2012		

Name of Sponsor/Company:	Individual Study Table Referring to Part of the Dossier	(For National Authority Use Only)
Name of Finished Product:	Volume:	
Name of Active Ingredient:	Page:	

Mode of administration:	Intravenous infusion over 5 minutes (maximum 7 minutes)
Dose:	As described for active treatment.

Criteria for Evaluation:

Safety:

- Adverse event monitoring, vital signs (blood pressure, heart rate and body temperature), cardiac monitoring (12-lead ECGs and telemetry), clinical laboratory assessments (standard haematology and biochemistry laboratory parameters).

Pharmacokinetics:

- Part 1: maximum observed plasma concentration (C_{max}), time corresponding to occurrence of C_{max} (t_{max}), terminal elimination rate constant (λ_z), apparent elimination half-life in plasma ($t_{½}$), area under the plasma concentration-time curve (AUC) from time zero to the last quantifiable concentration ($AUC_{0\text{-last}}$), AUC from time zero to infinity ($AUC_{0\text{-inf}}$), clearance (CL), and volume of distribution (V_z).
- Part 2: Following doses 1, 5 and 9 (Days 1, 2, 3): C_{max}, t_{max}, λ_z, $t_{½}$, AUC in the dosing interval ($AUC_{0\text{-tau}}$), and $AUC_{0\text{-inf}}$. Additionally, following dose 9: volume of distribution at steady state (V_{ss}), clearance at steady state (CL_{ss}), % fluctuation, average plasma concentration (C_{avg}), and accumulation ratio (AR).

Pharmacodynamics:

aPTT, PT, bleeding time and platelet function.

Statistical Methods:

The two study parts were considered separately for all analyses.

All subjects within a cohort who received placebo were pooled into a single placebo group for the final statistical analysis. The assignment of subjects to the various analysis sets were listed and summarised.

All subjects treated were accounted for with respect to number of received doses and days in the study until completion/withdrawal.

All statistical tests were two-sided and were performed at the 5% level of significance, unless otherwise stated.

Continuous data were summarised by treatment group using descriptive statistics (number, mean, standard deviation [SD], minimum, median and maximum). Categorical data were summarised by treatment group using frequency tables (frequency and percent).

Summary - Conclusions:

Pharmacokinetic Results:

Part 1

- Following single ascending 5 minute IV infusions of DF02, mean C_{max} of 2.7, 8.9, 28.8, 53.0, 97.2 and 114 µg/mL were recorded for doses of 10, 30, 90, 180, 360 and 420 mg, respectively. The increase was dose proportionate.
- Peak concentrations were achieved between 5 and 30 minutes after infusion start.
- The mean $t_{½}$ ranged from 0.66 to 1.0 hours and was dose independent.

Name of Sponsor/Company:	Individual Study Table Referring to Part of the Dossier	(For National Authority Use Only)
Name of Finished Product:	Volume:	
Name of Active Ingredient:	Page:	

Pharmacokinetic Results (continued):

Part 1 (continued)

- Exposure measured in terms of mean $AUC_{0\text{-}last}$ showed a dose proportionate increase from 7.1 h.µg/mL (10 mg dose) up to 140 h.µg/mL (420 mg dose).
- DF02 CL appeared independent of dose administered with means ranging from 2.58 to 2.99 L/h.

Part 2

180 mg 6-hourly

- Following 9 multiple doses of 180 mg 6-hourly, plasma concentration versus time profiles after Injections 1, 5 and 9 were almost superimposable, that is, no drug accumulation was observed. The mean AR was 1.03.
- Mean C_{max} was 47.5, 54.2 and 47.3 µg/mL for Injections 1, 5 and 9, respectively
- Time to peak concentration (t_{max}) showed a similar range to that after single doses.
- Mean $AUC_{0\text{-}6}$ was 63.6, 76.4 and 66.2 h. µg/mL for Injections 1, 5 and 9, respectively.
- Mean $t_{½}$ was similar to that after single doses, ranging from 0.87 to 1.24 hours.
- Clearance at steady state (CL_{ss}) was similar to that after single doses (mean 2.72 L/h).

360 mg 6-hourly

- Following nine multiple doses of 360 mg 6-hourly, plasma concentration versus time profiles after Injections 1, 5 and 9 were similar, only minimal drug accumulation was observed. The mean AR was 1.34.
- Mean C_{max} was 87.6, 80.7 and 104 µg/mL for Injections 1, 5 and 9, respectively.
- Time to peak concentration (t_{max}) showed a similar range to that after single doses and 180 mg 6-hourly.
- Mean $AUC_{0\text{-}6}$ increased slightly after multiple doses: 105, 115 and 141 µg/mL for Injections 1, 5 and 9, respectively, confirming the small amount of drug accumulation.
- Mean $t_{½}$ was similar to that after single doses and 180 mg 6-hourly, ranging from 0.86 to 1.21 hours.
- Clearance at steady state (CL_{ss}) was similar to that after 180 mg 6-hourly (mean 2.55 L/h).

Pharmacodynamic Results:

Part 1

- A linear, dose-dependent increase in aPTT was seen following single ascending DF02 doses. The mean maximal change from baseline was 0.8, 2.0, 13.8, 54.2, 72.7 and 95.4 seconds following doses of 10, 30, 90, 180, 360 and 420 mg, respectively.
- Peak aPTT response was 30 minutes post-dose; thereafter aPTT reduced towards baseline with a median normalisation time ranging from 2 hours post-dose (90 mg dose) to 4 hours post-dose (420 mg dose).
- Prothrombin time showed a small increase from baseline following all DF02 doses. The mean maximal response was between 0.5 and 2 hours post-dose and the magnitude of the response appeared to be dose independent; the maximum increase from baseline (1.2 seconds) occurring after a dose of 180 mg.
- Bleeding time increased marginally relative to placebo at doses up to 90 mg but the increase was not seen consistently at higher doses. All individual subject post-dose bleeding times were within the range of 2 to 10 minutes.
- PFA-100 data showed a small increase from baseline following the lowest dose of 10 mg, but no consistent increase was seen at higher doses.

Name of Sponsor/Company:	Individual Study Table Referring to Part of the Dossier	(For National Authority Use Only)
Name of Finished Product:	Volume:	
Name of Active Ingredient:	Page:	

Pharmacodynamic Results (continued):

Part 2

- A linear, dose-dependent increase in aPTT was seen following multiple 6-hourly IV DF02 doses of 180 mg and 360 mg. The mean maximal change from baseline was 41 and 110 seconds following the ninth injection of DF02 180 and 360 mg, respectively.
- A small build up in aPTT was seen over the 3 days of treatment at both dose levels; that is, slightly larger increases from baseline were seen following Injection 9 compared with Injections 5 and 1. The mean maximal change from baseline was 35 and 76 seconds following the first injection of DF02 180 and 360 mg, respectively.
- Peak aPTT response was 30 minutes post-dose; thereafter the response reduced with a median normalization time of 5 hours after Injection 9 (180 mg 6-hourly) and 6.5 hours after Injection 9 (360 mg 6-hourly).
- Prothrombin time increases were small; mean maximum increases from baseline ranged from 0.7 seconds (180 mg 6-hourly) to 1.5 seconds (360 mg 6-hourly) for all injections. The peak response was between 0.5 and 3 hours post-dose, with PT returning to baseline 6 hours after Injection 9 (180 mg 6-hourly) and >24 hours after Injection 9 (360 mg 6-hourly).
- Bleeding time showed an upward trend at 2 and 24 hours for the 360 mg dose following Injection 9 only. No other bleeding time changes were evident.
- Platelet function tests demonstrated no apparent difference in change from baseline data following active treatment compared to placebo treatment.

Safety Results:

No dose-related trend was observed in the prevalence of TEAEs in Part 1 or Part 2 of the study. However a greater number of TEAEs overall were reported following multiple dosing in Part 2 of the study, than following single dose administration in Part 1 of the study. The majority of TEAEs were considered mild and not related to the investigational product. One TEAE leading to withdrawal was reported in Part 1 of the study; elevated hepatic enzymes results following 10 mg DF02 in Subject 104. No SAEs were reported during the study.

No dose-related or treatment-related trends were observed in the median values of any biochemistry parameters or any haematology parameters other than aPTT. A dose-related increase was observed in the median aPTT values following single-ascending doses of DF02 in Part 1 and multiple doses of DF02 in Part 2 of the study. Activated partial thromboplastin time values above the reference range were observed in several subjects following treatment with DF02 in Parts 1 and 2, but values at the post-dose safety sample time points (2 and 5 hours post dose) were not considered significant in any subjects. No urinalysis values of note were observed in any subjects during the study.

There was no observable dose-related trend in any vital signs or 12-lead ECG parameters, and mean and median absolute values and changes over time were similar for all treatment groups in Parts 1 and 2 of the study. Twelve-lead ECG abnormalities were noted in 21 subjects overall, and abnormal ECG telemetry results in 3 subjects; none of these abnormalities were deemed to be CS by the Investigator.

No abnormal physical examination findings were observed in any subjects at follow-up in Parts 1 and 2 of the study.

Name of Sponsor/Company:	Individual Study Table Referring to Part of the Dossier	(For National Authority Use Only)
Name of Finished Product:	Volume:	
Name of Active Ingredient:	Page:	

**Conclusion:**
The reduced anticoagulant activity of DF02 in humans in comparison to normal heparin and anticoagulant LMWHs coupled with its good safety profile after single doses up to 420 mg and after multiple doses of 180 and 360 mg 6-hourly, support further research into the potential use of DF02 in the treatment of malaria.
**Date of Report:** Final 1.0, 13 January 2010
This study was conducted in compliance with International Conference on Harmonisation Good Clinical Practice guidelines and the Declaration of Helsinki.
