# Supplementary material for: Inhibition of merozoite invasion and transient de-sequestration by sevuparin in humans with Plasmodium falciparum malaria
Source: PLoS One. 2017 Dec 15;12(12):e0188754. doi: 10.1371/journal.pone.0188754 (PMC5731734; doi:10.1371/journal.pone.0188754)
Supplement: S3 Supportive Information — (PDF) [file pone.0188754.s003.pdf]

CONFIDENTIAL

CLINICAL TRIAL PROTOCOL

**PROTOCOL TITLE:**	**A Phase I/II, Randomized, Open Label, Active Control, Parallel Assignment, Safety/Efficacy Study of Sevuparin/DF02, as an Adjunctive Therapy in Subjects Affected with Uncomplicated Falciparum Malaria**
**PROTOCOL NUMBER:**	**Sevuparin/DF02_TSM02, Part 1 and Part 2**
**VERSION:**	**4.0, April 14, 2012 including Amendment 1.0 6th of May2011 and Amendment 2.0; April 14, 2012**
**INVESTIGATIONAL PRODUCT:**	**Sevuparin/DF02**
**SPONSOR NAME:**	**Dilaforette AB**
**SPONSOR ADDRESS:**	**Karolinska Institutet Science Park<br>Retzius väg 8, SE-171 65 Solna, Sweden**
**CONTRACT RESEARCH ORGANIZATION :**	**PAREXEL International (Thailand) Co., LTD.<br>Q House Sathorn Bld., 10 Fl., Zone AB,<br>11 Sathorn Rd., Tungmahamek, Sathorn,<br>Bangkok 10120, Thailand<br>Tel: +66 (0) 2639 3223<br>Mob: +66 (0) 8 7560 8204<br>Fax: +66 (0) 2679 2501**
**RESEARCH FACILITY**	**Mahidol-Oxford Research Unit<br>Faculty of Tropical Medicine<br>Mahidol University<br>420/6 Rajvithi Rd.<br>Bangkok 10400<br>Thailand**
**RESEARCH HOSPITALS**	**Maesot General hospital,<br>Mae Sot, Tak Province<br>Thailand**
	**Mae Ramat Hospital<br>Mae Ramat district, Tak province<br>Thailand**

Hospital for Tropical Diseases, Faculty of Tropical Medicine, Mahidol University, Bangkok
Thailand

SPONSOR MEDICAL EXPERT: **Per Arne Parment, MD., Ph,D**
TELEPHONE NUMBER: **+46-70-644 4771**
FAX NUMBER: **+46-8-32 31 44**

DSMB CONTACT NAME: **Prof. Dr. Sasithon Pukrittayakamee**
Department of Clinical Tropical Medicine
Faculty of Tropical Medicine
Mahidol University
Rajvithi Road 420/6
Bangkok 10400, Thailand

SAE CONTACT DETAILS

SAE REPORTS TO: **Phone: +33 1 44 90 32 90**
Fax : +33 1 44 90 32 75
E-mail: DF02_safety@parexel.com

MONITOR **Supatat Limpakdee , CRA**
+66 2 639 3205, +66 2 679 2501
supatat.limpakdee@parexel.com

CONFIDENTIALITY STATEMENT

Contains confidential/trade secret information subject to 18 USC § 1905 and to which all claims of privilege and confidentiality are asserted in both statutory and common law. Further dissemination may only be made with the express written permission of Dilaforette AB.

The information contained in this document, particularly unpublished data, is the property of Dilaforette AB and is provided to you in confidence as an Investigator, potential Investigator, or Consultant, for review by you, your staff, and an applicable Institutional Review Board or Independent Ethics Committee. The information is only to be used by you in connection with authorized clinical studies of the investigational drug described in the protocol. You will not disclose any of the information to others without prior written authorization from Dilaforette AB except to the extent necessary to obtain informed consent from those persons to whom the drug may be administered.

PROTOCOL AGREEMENT PAGE

Protocol Number: **Sevuparin/DF02_TSM02 (Part 1 and Part 2)**

Protocol Title: **A Phase I/II, Randomized, Open Label, Active Control, Parallel Assignment, Safety/Efficacy Study of Sevuparin/DF02, as an Adjunctive Therapy in Subjects Affected with Uncomplicated Falciparum Malaria**

IN WITNESS WHEREOF THE FOLLOWING HAVE AGREED TO BE BOUND BY THE TERMS OF THE SAID PROTOCOL BY SIGNING BELOW

On behalf of the Sponsor:

Signature: *N.A (Protocol Amendment No. 2 to be signed)* **Date:** *N.A*
Anna Lietgeb, PhD (Project Manager)

Signature: *N.A (Protocol Amendment No. 2 to be signed)* **Date:** *N.A*
Per Arne Parment, MD, PhD (Medical Expert)

On behalf of:

Maesot General Hospital, Maesot, Tak

Site Principal Investigator Signature: *N.A (Protocol Amendment No. 2 to be signed)* **Date:**
Ronnatrai Rueangveerayuth, MD, FM, BSc

Mae Ramat Hospital, Tak

Site Principal Investigator Signature: [signature] **Date:** 21/5/2012
Chirapong Uthaisin, MD

Hospital for Tropical Diseases, Bangkok

Site Principal Investigator Signature: **Date:**
Prakaykaew Charunwatthana, MD, PhD

PROTOCOL AGREEMENT PAGE

Protocol Number: **Sevuparin/DF02_TSM02 (Part 1 and Part 2)**

Protocol Title: **A Phase I/II, Randomized, Open Label, Active Control, Parallel Assignment, Safety/Efficacy Study of Sevuparin/DF02, as an Adjunctive Therapy in Subjects Affected with Uncomplicated Falciparum Malaria**

IN WITNESS WHEREOF THE FOLLOWING HAVE AGREED TO BE BOUND BY THE TERMS OF THE SAID PROTOCOL BY SIGNING BELOW

On behalf of the Sponsor:

Signature: *N.A (Protocol Amendment No. 2 to be signed)* **Date:** *N.A*
Anna Lietgeb, PhD (Project Manager)

Signature: *N.A (Protocol Amendment No. 2 to be signed)* **Date:** *N.A*
Per Arne Parment, MD, PhD (Medical Expert)

On behalf of:

Maesot General Hospital, Maesot, Tak

Site Principal Investigator Signature: *N.A (Protocol Amendment No. 2 to be signed)* **Date:**
Ronnatrai Rueangveerayuth, MD, FM, BSc

Mae Ramat Hospital, Tak

Site Principal Investigator Signature: ____________ **Date:** ________
Chirapong Uthaisin, MD

Hospital for Tropical Diseases, Bangkok

Site Principal Investigator Signature: [signature] **Date:** 24 /5 /12
Prakaykaew Charunwatthana, MD, PhD

On behalf of:

Mahidol-Oxford Tropical Medicine Research Unit
420/6 Rajvithee rd
Bangkok 10400
Thailand

**Principal Investigator Signature:**	***N.A (Protocol Amendment No. 2 to be signed)***	**Date:**	***N.A***
	Arjen Dondorp, MD PhD		

TABLE OF CONTENTS

CONFIDENTIALITY STATEMENT **3**
PROTOCOL AGREEMENT PAGE **4**
TABLE OF CONTENTS **6**
GLOSSARY **11**
PROTOCOL SYNOPSIS **17**
1 BACKGROUND INFORMATION **22**
1.1 The Disease and Current Treatments **22**
1.2 Name and Description of the Investigational Product **23**
1.3 Pre-Clinical Pharmacology Study Results **24**
1.4 Toxicology **26**
1.4.1 Pre Clinical Toxicology 26
1.4.2 Toxicokinetic Studies 28
1.4.3 Clinical Toxicology 29
1.5 Summary of Clinical Data **32**
1.6 Rationale **38**
1.6.1 Rationale for Dosage 39
1.6.2 Conduct of Trial 40
2 TRIAL OBJECTIVES AND PURPOSES **40**
2.1 Objectives **40**
2.1.1 Primary Objective 40
2.1.2 Secondary Objectives 41
3 STUDY DESIGN **41**
3.1 Primary and secondary endpoints **46**
3.1.1 Part 1 46
3.1.2 Part 2 46
3.2 Concomitant Medication and Treatment **47**
3.2.1 Palliative and Supportive Care 47
3.3 Restrictions **47**
3.3.1 Prior Therapy 47
3.3.2 Concomitant medication 47

3.3.3 Fluid and Food Intake 48
3.3.4 Subject Activities and Restrictions 48
3.4 Randomization and Blinding 48
3.5 Number of Subjects in the Study 48
3.6 Centers 48
3.7 Population 48
3.7.1 Subject Inclusion Criteria 49
3.7.2 Subject Exclusion Criteria 49
4 STUDY TREATMENT 50
4.1 Study Drug 50
4.2 Study Therapy Administration –Sevuparin/DF02 50
4.2.1 Study Therapy Reconstitution 51
4.3 Selection and Timing of Dose for Each Subject 51
4.4 Dose Modification 51
4.4.1 Dose Modifications of Sevuparin/DF02 51
4.5 Packaging 51
4.5.1 Atovaquone-proguanil (Malanil®): 51
4.6 Labelling 52
4.7 Storage 53
4.7.1 Destruction of Surplus Medication 53
4.8 Storage and Accountability 53
4.8.1 Sevuparin/DF02 53
4.8.2 Malanil® 54
4.9 Investigational Product Retention at Study Site and Destruction 54
4.10 Duration 54
4.11 Table of study evaluations 55
4.12 Criteria for Stopping Treatment 58
4.13 Rescue medication patients receving Sevuparin/DF02 58
5 STUDY PROCEDURES 58
5.1 Informed Consent 58
5.2 Subject Identification 59
5.2.1 Subject Number 59
5.3 Medical History 59

5.4 Physical Examination ... 59
5.5 Vital Signs and Cardiovascular Assessments ... 59
5.6 Dispensing Study Drug ... 60
5.6.1 Malanil® ... 60
5.7 Clinical Laboratory Tests ... 60
5.7.1 Laboratory Parameters ... 60
5.7.2 Pharmacokinetics (PK) ... 61
6 STUDY ACTIVITIES ... 61
6.1 Screening Procedures for Study Entry part 1 and part 2 ... 61
6.2 Treatment Period ... 62
6.2.1 Criteria of early treatment failure are defined below: ... 62
6.2.2 Part 1 all patients and part 2 patients randomzied to combination treatment ... 63
6.2.3 Part 2 patients randomsied to Malanil® alone ... 66
7 SELECTION AND WITHDRAWAL OF SUBJECTS ... 68
7.1 Pregnancy ... 68
7.2 Subject Withdrawal Criteria ... 68
8 ASSESSMENT OF SAFETY ... 69
8.1 Specification of Safety Parameters ... 69
8.1.1 Adverse Events ... 69
8.1.1.1 Adverse Drug Reaction (ADR) ... 70
8.1.2 Laboratory Test Abnormalities ... 70
8.1.3 Serious Adverse Event ... 70
8.1.4 Follow-up of Adverse Events ... 71
8.1.5 Follow-up of Abnormal Laboratory Values ... 71
8.1.6 Pregnancy ... 71
8.2 Assessing, Recording, and Analyzing Safety Parameters ... 72
8.2.1 ECG Parameters ... 72
8.3 Recording and Reporting Adverse Events/Intercurrent Illnesses ... 72
8.3.1 Relationship of AE to Study Medication ... 73
8.3.2 Unexpected Adverse Event ... 73
8.3.3 Reporting of Serious Adverse Events ... 74
8.4 Warnings and Precautions ... 75
8.4.1 Sevuparin/DF02 Precautions ... 75

8.4.2 Malanil@ Precautions 75
8.4.3 Interaction with Other Medicine Products 76
8.5 Eficacy Assessments 77
8.5.1 Pharmacokinetics (part 1 all patients and part 2, 10 patients) 77
8.5.2 Pharmacodynamics (part 2) 77
9 STATISTICAL CONSIDERATIONS 78
9.1 General Considerations 78
9.1.1 Sample Size and Associated Power 79
9.1.2 Randomization of the Trial 79
9.2 Populations 79
9.2.1 Safety Population 79
9.2.2 Pharmacokinetic Population 79
9.3 Analysis of Safety 79
9.3.1 Incidence of Adverse Events 80
9.3.2 Laboratory Data 80
9.3.2.1 Analysis of paracites 80
9.4 Analysis of Pharmacokinetics 81
9.4.1 Plasma Pharmacokinetic Parameters of Sevuparin/DF02 81
9.5 Subject Accountability and Missing Data 81
9.6 Interim Analysis 82
10 DIRECT ACCESS TO SOURCE DATA / DOCUMENTS AND INVESTIGATOR RESPONSIBILITY 82
10.1 Source Data 83
10.2 Source Documents 83
10.3 Direct Access 83
10.4 Study Monitoring 83
11 QUALITY CONTROL/ QUALITY ASSURANCE 84
11.1 Quality Control 84
11.2 Quality Assurance 84
11.2.1 Inspection 84
11.2.2 Audit 84
12 ETHICS APPROVALS 85
12.1 Independent Ethics Committee (IEC) 85
12.2 Institutional Review (IRB) 85

12.3 Drug Safety Committee (Data Safety Monitoring Board –DSMB)........................ 86

13 DATA HANDLING AND RECORD KEEPING... 87

13.1 Completion of Case Report Forms... 87

13.2 Archiving 87

14 FINANCING AND INSURANCE 87

15 PUBLICATION POLICY 88

15.1 Publication Policy 88

15.2 Confidentiality.. 88

16 MODIFICATION OF PROTOCOL AND PROTOCOL AMENDMENTS.................. 89

17 REFERENCES.. 90

18 APPENDICES 92

APPENDIX 1 World medical association declaration of Helsinki 93
APPENDIX 2 Criteria For Severe Malaria: WHO 2010..98
APPENDIX 3 ICH Guidelines for Clinical Safety Data Management, Definitions and Standards for Expedited Reporting, Topic E2 99
APPENDIX 4 Dispensing Guidelines For Sevuparin/Df02 101
APPENDIX 5 Infusion Reaction Criteria 102
APPENDIX 6 Anti-malarial regimen to be used during the study.....................................103
APPENDIX 7 Cytoadherence assay 128
APPENDIX 8 Rosetting formation assay 129
APPENDIX 9 CTCAE V. 4.0...130
APPENDIX 10 Blood and urine volumes 131

GLOSSARY

a-FXa	anti-Factor Xa
a_FIIa	anti-Factor IIa thrombin)
ADR	Adverse Drug Reaction
AGC	Absolute Granulocyte Count
ALT	Alanine Aminotransferase
ANC	Absolute Neutrophil Count
APTT	Activated Partial Thromboplastin Time
AT	Anti-thrombin III
5-ASA	5- Aminosalicylic Acid
AST	Aspartate Aminotransferase
AUC	Area Under the Curve
BSA	Body Surface Area
BUN	Blood Urea Nitrogen
CBC	Complete Blood Count
CEA	Carcinoembryonic Antigen
CFR	Code of Federal Regulations
CL	Clearance
Cmax	Maximum plasma concentration
CNS	Central Nervous System
CRF	Case Report Forms
CR	Complete response
CRA	Clinical Research Associate
Css	Steady-state plasma concentrations
CT	Computed Tomography
CTCAE	Common Terminology Criteria for Adverse Events v4.0
DBL1α	Duffy-binding-like domain 1α
DCF	Data Clarification Form
DF01	Tafoxiparin
DF02	Sevuparin
DLT	Dose Limiting Toxicities
DOB	Date of birth
DHFR	Dihydrofolate Reductase
DSMB	Data Safety Monitoring Board
EC	Ethics Committee
ECG	Electrocardiogram
ESF	Eligibility Screening Form
ELISA	Enzyme-Linked Immunosorbent Assay
FDA	Food and Drug Administration
GCP	Good Clinical Practice
GI	Gastrointestinal
GlcN	D-glucosamine moiety N-sulfated or N-acetylated.
GLP	Good Laboratory Practice
Hb	Haemoglobin
Hct	Hematocrit
HIPAA	Health Insurance Portability and Accountability Act
HIV	Human Immunodeficiency Virus
HRP	Horseradish peroxidase
HS	Heparin Sulphates
IE	Infected Erythrocytes
i.v.	Intravenous
ICF	Informed Consent Form
ICH	International Conference on Harmonization
IE	Infected erythrocytes

IRB	Institutional Review Board
kDa	Kilo Daltons
LAH	Low Anticoagulant Heparin
LDH	Lactic Acid Dehydrogenase
LLN	Lower Limit of Normal
LMWH	Low Molecular Weight Heparin
LOQ	Limit of Quantification
LLOQ	Lower Limit of Quantification
m2	Meters squared
MedDRA	Medical dictionary for regulatory activities
mg	Milligrams
mL	Milli Litre
MCH	Mean Corpuscular Hemoglobin
MCHC	Mean Corpuscular Hemoglobin Concentration
MCV	Mean Corpuscular Volume
MTD	Maximum Tolerated Dose
µL	Micro Litre
NOAEL	No Observable Adverse Effect Level
NOEL	No Observable Effect Level
p.o.	Per orally
PfEMP1	Plasmodium falciparum erythrocyte membrane protein 1
PhEur	European Pharmacopeia
PI	Principal Investigator
PK	Pharmacokinetic
PT	Prothrombin time
pKa	pH at which an acid is half-dissociated (measure of strength of an acid)
RBC	Red Blood Cell
SAD	Single Ascending Dose
SAE	Serious Adverse Event
SAP	Statistical Analysis Plan
SD	Stable Disease
SEA	South East Asia
SGOT	Serum glutamic-oxaloacetic transaminase/aspartate Aminotransferase (AST)
SGPT	Serum glutamic-pyruvic transaminase/alanine aminotransferase (ALT)
SPSS	SPSS statistical software
STATA	STATA statistical software
TEAES	Treatment Emergent Adverse Event by System
Tmax	Time to Maximum Plasma Concentration
UA	Uronic Acid
US	United States
USP	United Stated Pharmacopeia
ULN	Upper Limit of Normal
ULOQ	Upper Limit of Quantification
Vd	Volume of distribution
WBC	White Blood Cell
WHO	World Health Organisation
WMA	World Medical Association
WNL	Within Normal Limits

List of tables

Table 1.3.T1 *In Vitro* and *In Vivo* Studies……………………………………………………….…24

Table 1.3.T2 Pre-Clinical Pharmacology Study Results…………………………………………....25

Table 1.4.1.T1 Pre-Clinical Toxicology Study Results……………………………………………....26

Table 1.4.2.T1 Systemic Exposure of Sevuparin/DF02 on Day 14 after IV 4, 20 and 60 mg/kg/day to Male and Female Sprague Dawley Rats……………………………………….…....28

Table 1.4.2.T2 Sevuparin/DF02 Toxicokinetic Parameters in Dogs Treated Once Daily with Sevuparin/DF02…………………………………………..…………………....28

Table 1.4.3.T1 Treatment Emergent Adverse Event by System Organ Class – Part 1………………....29

Table 1.4.3.T2 Treatment Emergent Adverse Event by System Organ Class – Part 2…………………31

Table 1.5.T1 Part 1 Dosing Design……………………………………………………………...33

Table 4.11.T1 Study Schedule of Evaluations…………………………………………….…...55

Table 4.11.T2 PK Blood Samples, APTT, PT (coagulation), and antiXa Activity…….……………...57

List of figures

Figure 1.2.F1 Representative Example of Heparin Sequence……………………………………..24

Figure 1.2.F2 Predominant Structure of Sevuparin/DF02……………………………………..…24

Figure 1.5.F1 Part 1 Study Design…………………………………………………………….…..34

Figure 1.5.F2 Part 2 Study Design…………………………………………………………..…35

Figure 3.0.F1 Overview of Study Design and Dosing Regimen for all patients in Part 1………..….43

Figure 3.0.F2 Overview of Study Design and Dosing Regimen for patients in part 2 randomised to the Anti-malarial regimen (Malanil®) alone and Sevuparin/DF02 plus anti-malarial regimen (Malanil®)..…………………………………………………… ….…..44

Figure 3.0.F3 Study Dose Escalation Scheme (Fibonacci Dose Escalation Scheme) in Part 1…..…..45

Figure 8.5.2.F1 Morphological Stages of the *P. Falciparum*…………………….……..…….………...78

PROTOCOL REVISION HISTORY

Revision History	Dec 21, 2010	Original: Version 1.0
	March 4, 2011	Version 2.0
	March 14, 2011	Protocol version 2.1
	May 6, 2011	Protocol version 3.0, including Amendment 1.0
	April 14, 2012	Protocol version 4.0, Including Amendments 1.0 and 2.0

PROTOCOL SUMMARY / SYNOPSIS

**PROTOCOL TITLE:**	**A Phase I/II, Randomized, Open Label, Active Control, Parallel Assignment, Safety/Efficacy Study of Sevuparin/DFO2, as an Adjunctive Therapy in Subjects Affected with Uncomplicated Falciparum Malaria**
**PROTOCOL NUMBER:**	**Sevuparin/DF02_TSM02, Part 1 and Part 2**
**VERSION:**	**4.0, 14th of April 2012**
**INCLUDING AMENDMENTS:**	**1.0, 6th of May 2011 and 2.0, 14th of April 2012**
**INVESTIGATIONAL PRODUCT:**	**Sevuparin/DF02**<br><br>**The names Sevuparin and DF02 are used interchangeably in the context of this protocol**
**SPONSOR NAME:** `	**Dilaforette AB**
**SPONSOR ADDRESS:**	**Karolinska Institutet Science Park**<br>**Retzius väg 8, SE-171 65 Solna, Sweden**
**CONTRACT RESEARCH ORGANIZATIONS:**	**Regulatory contact; ARIANNE Corp.**<br>**9444 Waples Street, Suite 160**<br>**San Diego, California 92121, USA**<br><br>**PAREXEL International (Thailand) Co., LTD.**<br>**Q House Sathorn Bld., 10 Fl., Zone AB,**<br>**11 Sathorn Rd., Tungmahamek, Sathorn,**<br>**Bangkok 10120, Thailand**
**RESEARCH FACILITY**	**Mahidol-Oxford Tropical Medicine Research Unit,**<br>**Faculty of Tropical Medicine**<br>**Mahidol University**<br>**420/6 Rajvithi rd**<br>**Bangkok 10400, Thailand**
**RESEARCH HOSPITALS**	**Mae Sot General hospital,**<br>**Mae Sot, Tak Province**<br>**Thailand**<br><br>**Mae Ramat Hospital**<br>**Mae Ramat district, Tak province**<br>**Thailand**<br><br>**Hospital for Tropical Diseases, Faculty of Tropical Medicine, Mahidol University, Bangkok**<br>**Thailand**
**SPONSOR MEDICAL EXPERT:**	**Per Arne Parment. MD., Ph.D**<br>**Dilaforette AB**<br>**Karolinska Institutet Science Park**<br>**Nobels väg 3, SE-171 65 Solna, Sweden**
**SAE CONTACT DETAILS:**	**Prof. Dr. Sasithon Pukrittayakamee**<br>**Department of Clinical Tropical Medicine**

Faculty of Tropical Medicine
Mahidol University
420/6 Rajvithi rd
Bangkok 10400, Thailand

Tel +66 2 3549100 ext. 1435
Fax. +66 2 3549169

**SAE REPORTS TO:**	**Phone: +33 1 44 90 32 90**<br>**Fax : +33 1 44 90 32 75**<br>**E-mail: DF02_safety@parexel.com**
**MONITOR:**	**Supatat Limpakdee , CRA**<br>**+66 2 639 3205, +66 2 679 2501**<br>**supatat.limpakdee@parexel.com**

PROTOCOL SYNOPSIS

**Protocol Number:**	Sevuparin/DF02_TSM02, Part1 and Part 2
**Protocol Title:**	A Phase I/II, Randomized, Open Label, Active Control, Parallel Assignment, Safety/Efficacy Study of Sevuparin/DF02, as an Adjunctive therapy in Subjects Affected with Uncomplicated Falciparum Malaria
**Study Phase:**	I/II (Part 1 is phase I study in patients and Part 2 is a phase II study)
**Principal Investigators:**	Site Principal Investigator , Mae Sot General Hospital: Dr. Ronnatrai Rueangveerayuth, MD<br><br>Site Principal Investigator Mae Ramat Hospital: Chirapong Uthaisin, MD<br><br>Site Principal Investigator Hospital for Tropical Diseases, Bangkok: Prakaykaew Charunwatthana, MD, PhD<br><br>Principal Investigator: Dr. Arjen Dondorp, Mahidol-Oxford Tropical Medicine Research Unit, Faculty of Tropical Medicine, Mahidol University, Bangkok, Thailand
**Study Centers:**	Maesot General Hospital, Mae Sot, Tak Province, Thailand<br><br>Mae Ramat Hospital, Mae Ramat district, Tak province, Thailand<br><br>Hospital for Tropical Diseases, Faculty of Tropical Medicine, Mahidol University, Bangkok, Thailand
**Number of Subjects:**	Approximately 95 patients with uncomplicated falciparum malaria will be recruited to the study. It is anticipated that a maximum of 15 patients will be required for the MTD study. For the extension study a total of 80 patients, hence the total number of patients for the study is 95.<br>The number of expected patients will be between 83 and 95.
**Study Drug:**	Sevuparin/DF02 as an adjunctive therapy with Malanil® (atovaquone/proquanil)
**Project Duration:**	18 months
**Interim safety report**	There will be a safety report submitted to the ethical committees concerned, after the first two dose levels in part 1 of the study have been administered and the results have been evaluated by the DSMB. Part 2 can only start after safety report has been approved and the DSMB has stated that there are no safety concerns to continue the study.
**Objectives Part 1:**	**Objectives:**<br>*Primary:* To characterize the safety, and dose-limiting toxicities (DLTs) for Sevuparin/DF02 when administered IV, repeatedly, in combination with defined antimalarial treatment in adult patients with uncomplicated falciparum malaria; prevalence, severity, drug-relatedness, seriousness of adverse events of Sevuparin/DF02.<br>3.<br>*Secondary:*<br>• To characterize the pharmacokinetic (PK) profile of Sevuparin/DF02 when administered intra-venous (IV), repeatedly in combination with defined, antimalarial treatment in adult patients with uncomplicated falciparum malaria.<br>• To determine the pharmacokinetic parameters of half-life, Cmax, AUC, Tmax of Sevuparin/DF02.<br>• To determine the area under the curve of late stage peripheral blood parasitemia over time curve.

	• To characterise the difference in sequestration of mature form *P. falciparum* parasites in patients treated with Sevuparin/DF02<br>• To determine *ex-vivo* the ability of Sevuparin/DF02 to reverse and prevent rosette formation of IE to uninfected erythrocytes and to block cytoadherence of IE to endothelial cells.
**Objectives Part 2:**	**<u>Objectives:</u>**<br>*Primary:*<br>• To assess the anti-adhesive properties of Sevuparin/DF02 measured as the ability to reverse and prevent sequestration of mature stage parasitized erythrocytes in adult patients with uncomplicated falciparum malaria.<br><br>*Secondary:*<br>• To characterize the safety, for Sevuparin/DF02 when administered IV, repeatedly, in combination with defined antimalarial treatment in adult patients with uncomplicated falciparum malaria; prevalence, severity, drug-relatedness, seriousness of adverse events of Sevuparin/DF02 will be recorded.<br>• To determine the pharmacokinetic parameters of half-life, Cmax, AUC, Tmax of Sevuparin/DF02, of the 10 first patients randomized to the Sevuparin/DF02 arm.<br>• To determine the area under the curve of late stage peripheral blood parasitemia over time curve.<br>• To characterise the difference in sequestration rate of mature form *P. falciparum* parasites in patients treated with Sevuparin/DF02 compared to control group.<br>• To determine *ex-vivo* the ability of Sevuparin/DF02 to reverse and prevent rosette formation of IE to uninfected erythrocytes and to block cytoadherence of IE to endothelial cells.
**Study Design part 1:**	This study will be an open dose escalation/safety and efficacy study Sevuparin/DF02, to assess the safety, tolerability and dose-limiting toxicities (DLTs) for Sevuparin/DF02 when administered intravenous (IV) as a single agent, in combination with Malanil®as antimalarial therapy in patients with uncomplicated falciparum malaria.<br>When the first two dose levels in part 1 (1.5 and 3 mg/kg/dose) have been evaluated by the DSMB and assessed as safe, part 2 of the study will start recruitment on dose level 3mg/kg/dose. Recruitment to the last cohort in part 1 (6 mg/kg/dose) will continue in parallel with start of part 2.
**Study design part 2:**	Part 2 is an active controlled randomized two arm study further evaluating the safety and ex vivo efficacy of Sevuparin/DF02 when administered with Malanil® as an adjunctive antimalarial therapy versus anti-malarial treatment with Malanil® alone.<br>Patients will be randomized to Sevuparin/DF02 or no adjunctive therapy in a 1:1 ratio.<br>When 10 patients in each treatment group (active/control) have been treated, an interim evaluation of safety and efficacy will be performed, including the data for the last cohort of part 1 (6 mg/kg/dose). Based on this evaluation it will be decided if the dose for the following patients should be increased or the study continued on 3 mg/kg/dose.
**Endpoints Part 1:**	**Primary endpoint**<br>• Safety and tolerability; Prevalence, severity, drug-relatedness, seriousness of adverse events of Sevuparin/DF02 as adjunctive therapy to Malanil®<br>• dose-limiting toxicities (DLTs) for Sevuparin/DF02 as adjunctive therapy to Malanil®<br>**Secondary endpoints**<br>• Pharmacokinetic parameters of half-life, Cmax, AUC, Tmax of Sevuparin/DF02 as adjunctive therapy to Malanil®

**Endpoints Part 2:**	**Primary endpoint**<br>Area under the curve of the graph plotting the late stage peripheral blood parasitemia over time of sevuparin/DF02 as adjunctive to malanil® in comparison to malanil® alone<br>**Secondary endpoints**<br>• Safety and tolerability; Prevalence, severity, drug-relatedness, seriousness of adverse events of Sevuparin/DF02 as adjunctive therapy to Malanil®<br>• Pharmacokinetic parameters of half-life, Cmax, AUC, Tmax of Sevuparin/DF02 as adjunctive therapy to Malanil®<br>• Area under the curve of late stage peripheral blood parasitaemia over time curve Sevuparin/DF02 as adjunctive therapy to Malanil® in comparison to Malanil® group.<br>• Difference in the sequestration rates of the Sevuparin/DF02 as adjunctive therapy to Malanil® group in comparison to the Malanil® group
**Study Populations:**	Male or female subjects, 18 to 65 years of age (inclusive), with acute uncomplicated plasmodium *falciparum* malaria, defined as febrile disease with a positive peripheral blood malaria slide.
**Study Drug Administration Part 1:**	For the Maximum Tolerated Dose (MTD) study, subjects will be dosed according to a dose escalation scheme starting with a dose of 1.5 mg/kg/dose and escalating until MTD is reached (max dose of 6 mg/kg/dose). Subjects will receive Sevuparin/DF02 as an adjunctive to Malanil@. Sevuparin/DF02 is given as iv infusion and Malanil@ is given orally. A written recommendation by the DSMB concerning the dose to be used in part 2 included in the interim safety report will be submitted to the concerned ethical committees before the part 2 of the study will commence.
**Study Drug Administration Part 2:**	• Half of the patients will receive Malanil@ alone and half of the patients will receive 3 mg/kg/dose Sevuparin/DF02 in combination with Malanil@<br>• A maximum total dose of 360 mg can be given per dosing occasion<br>• The dose of Sevuparin/DF02 is based in the results from part 1.<br>• Sevuparin/DF02 is given as iv infusion and Malanil® is given orally.
**Inclusion Criteria:**	1. Male or female, 18 to 65 years old, inclusive<br>2. Presence of acute uncomplicated *P. falciparum* malaria, confirmed by positive blood smear with asexual forms of a single species (*P. falciparum*)<br>3. Counts of asexual forms of *P. falciparum*: 10 000-100 000/ul with or without gametocytaemia<br>4. Presence of fever defined as $\geq$ 38° C tympanic temperature or a history of fever within the last 24 hours<br>5. Written informed consent<br>6. Willingness and ability of the patient to comply with study protocol for the duration of the study
**Exclusion Criteria:**	1. Mixed infection with other Plasmodium species<br>2. History of significant bleeding (e.g. upper GI bleeding, recurrent epistaxis, joint bleeding, melena)<br>3. Patient with any criteria of severe or complicated malaria as defined by the World Health Organization, 2010 (Appendix 2)<br>4. Known history or evidence of clinically significant disorders: neurological, psychiatric (depression, psychosis or schizophrenia), cardiovascular (including arrhythmia), pulmonary, metabolic, gastrointestinal, endocrine diseases or malignancies.<br>5. For females: pregnancy, lactating or intention of becoming pregnant within the expected duration of the study<br>6. Patients with history of convulsions<br>7. Use of high doses aspirin (more than 100mg/day) or dual anti-platelet therapy or use

	of heparin,LMWH or warfarin<br>8. Presence of significant anemia as defined by Hb <8 g/dL or Hct < 25%<br>9. A platelet count < 50,000/µL<br>10. Presence of febrile conditions caused by diseases other than malaria<br>11. Patients with abnormal laboratory result including<br>• Haematology: WBC<2000 or >12 000 cell/mcl<br>• Coagulation: PT and APTT >1.5 from upper normal range<br>12. Liver function tests (AST, ALT and alkaline phosphatase levels) more than 2.5 times the upper limit of normal range<br>13. Treatment with anti-malarial drug or antibiotics with anti-malarial activity within 7 days prior to enrolment or treatment with mefloquine within 30 days prior to current episode<br>14. Have a history of a clinically significant drug allergy to one or more of the study drugs, including sensitivity to heparin or LMWHs. History of hypersensitivity to the investigational medicinal product or any of the excipients or to medicinal products with similar chemical structures<br>15. Patients who have had splenectomy<br>16. Patients with known human immunodeficiency virus (HIV) infection<br>17. Participation in another clinical trial within 30 days is not allowed.
**Efficacy Outcomes:**	The expected efficacy outcomes will be assessed by<br>• Area under the curve of late stage peripheral blood parasitemia over time curve.<br>• Evaluation of the difference in sequestration of mature form *P. falciparum* parasites in patients treated with Sevuparin/DF02 compared to control group.<br>• Detection of the *ex-vivo* the ability of Sevuparin/DF02 to reverse and prevent rosette formation of IE to uninfected erythrocytes and to block cytoadherence of IE to endothelial cells.
**Safety Outcomes:**	Adverse events will be assessed throughout all phases of the study. Periodic physical examinations, including vital signs (blood pressure, heart rate and body temperature), cardiac monitoring (12 lead ECG) and clinical laboratory assessments (heamatology including coagulation and chemistry).
**Pharmacokinetic Outcomes:**	All patients in part 1 and an additional 10 patients of part 2 will be sampled for the PK analysis. The following PK parameters will be calculated from the determination of levels of Sevuparin/DF02 in plasma by an ELISA method: maximum plasma concentration (C_{max}), T_{max}, area under the curve to the final time with a concentration >LOQ [AUC_{∞}] and to infinity [$AUC_{(0\text{-}inf)}$], elimination half-life (t½), clearance (CL) and volume of distribution (V_d).
**Statistical Methods:**	All statistical analysis including all tables, figures and data listings will be performed by SPSS software (version 15.0) and STATA (version 10). Data will be log transformed to obtain a normal distribution, where necessary. Normally distributed data will be compared using Student's t test. The Mann-Whitney U test will be used for nonpaired nonparametric data. Categorical data will be compared by Pearson's chi-squared test or Fisher's exact test, as appropriate. The level of significance will be p<0.05. Summary statistics for continuous variables will include the mean, standard deviation, median, minimum, and maximum value; categorical variables will be presented as counts and percentage.

<table>
<tr>
<td>Study Drugs Description:</td>
<td>
<u>Investigational Product:</u>

Sevuparin/DF02 a 150 mg/mL, sterile, aqueous solution for intravenous administration
Sevuparin/DF02 is in a phosphate buffer 0.015M.
5 mL drug is dispensed in a 10 mL glass vial, sealed with a rubber stopper covered with a tear-off aluminium cap.
Sevuparin/DF02 vial is to be stored refrigerated (2-8°C or 35.6 – 46.4 F°) protected from light and heat until use.

<u>Standard of Care:</u>

Atovaquone-proguanil (Malanil®): Atovaquone and proguanil hydrochloride) is a fixed-dose combination of the antimalarial agents atovaquone and proguanil hydrochloride. The chemical name of atovaquone is trans-2-[4-(4-chlorophenyl)cyclohexyl]-3-hydroxy-1,4-naphthalenedione. The chemical name of proguanil is 1-(4-chlorophenyl)-2-(N'-propan-2-ylcarbamimidoyl) guanidine.
Each coated tablet contains 250 mg atovaquone /100 mg proguanil. They are packaged in PVC aluminium foil blister pack/s containing 12 tablets.
This medicinal product does not require any special storage conditions

</td>
</tr>
</table>

1 BACKGROUND INFORMATION

1.1 The Disease and Current Treatments

In 2008, there were 247 million cases of malaria and nearly one million deaths, mostly among children living in Africa (http://www.who.int/mediacentre/factsheets/fs094/en/index.html). Cases in Africa account for approximately 90% of the malaria cases in the world (WHO, 1996). During 2009, the number of estimated malaria cases in the South-East Asia Region (SEA) was 26-36 million (http://www.searo.who.int/en/Section10/Section21/Section340_4018.htm), where 10 out of 11 countries are malaria endemic. Around 70% of the population in the SEA region is at risk of malaria. In Thailand the risk of contracting malaria is mainly confined to the border areas with Myanmar, Cambodia and Malaysia. The estimated annual direct and indirect costs of malaria were $800 millions in 1987 and were expected to exceed $1.8 billion by 1995 (Anderson *et al*, 1996).

Malaria in humans is caused by four species of the genus plasmodium including *P*. falciparum, *P*. vivax, *P*. malariae, and *P*. ovale. The species responsible for the vast majority of fatal infections is *P*. falciparum causing severe malaria. Important manifestations of severe malaria include cerebral malaria, metabolic acidosis, severe anaemia and other vital organ failure.

Pivotal in the pathogenesis of severe falciparum malaria is the sequestration of parasitized red blood cells containing the mature form of the parasite in the microcirculation of vital organs including the brain. Sequestration is the results of binding of *P*. falciparum-infected erythrocytes (IE) to the vascular endothelium (cytoadherance). In addition, IE bind to infected (auto-agglutination) and non-infected erythrocytes (rosetting, giant-rosetting). As a result, microcirculatory flow of vital organs can be severely impaired directly contributing to the acute patholophysiology of severe human malaria.

The parasite employs heparin sulphates (HS) during the process of adherence both to the endothelium and to erythrocytes. Binding to the HS receptor is mediated by the N-terminal Duffy-binding-like domain 1α (DBL1α) of PfEMP1 in which high-affinity binding requires 12-mers of HS as well as the presence of N-2, and 6-O-sulfate. HS and heparin inhibit and reverse cytoadherence and rosetting of laboratory strains and wild isolates *in vitro*. Furthermore, the capacity of IE to adhere to HS is more frequent in isolates of children with severe malaria, suggesting HS to be one of the sequestration receptors participating in the causation of severe malaria (Vogt, A.M *et al* 2003; Vogt. A.M *et al* 2004; Carlson J. *et al* 1990; Heddini A. *et al*, 2001; Chen, Q. *et al*, 1998).
Heparins have been shown to disrupt cytoadherence and rosette formation *in vitro* and have been previously used in the treatment of severe malaria (Jaroonvesama, N. 1972; .Sheehy, T. W. & Reba, R. C. 1967; Smitskamp, H. & Wolthuis, F. H. 1971; Munir, M., *et al* 1980). Their use was discontinued because of their anticoagulant effect, causing an increased risk for bleeding, including intracranial haemorrhages. Heparins are composed of the same building blocks (glucosamine and glucoronic- or iduronuic acid) as the chemically closely related HS deployed by the parasite and negatively charged (sulfate groups). The anticoagulant effect of heparins depends on a single pentameric sequence which has a strong affinity to anti-thrombin III (AT). Modification of this pentameric sequence deprives heparins from their strong anticoagulant

activity, whereas their inhibitory effect on cytoadherence and rosette formation of *P. falciparum* is maintained. The proposed investigational drug Sevuparin/DF02 is based on this principle.

There is an acute need to develop new therapies for severe cases of malaria, since even with optimal antimalarial drug treatment mortality remains high. One of the approaches would be based on a well-designed antagonist that would significantly reduce sequestration of IE in the microvasculature of vital organs by restoring microcirculatory flow. Dilaforette has developed a HS receptor antagonist mimicking heparane sulfate, Sevuparin/DF02, which holds promise as an adjunctive therapy for the treatment of severe falciparum malaria. The antagonist is a low anticoagulant heparin (LAH). Based on preliminary preclinical studies conducted by Dilaforette, it is expected that Sevuparin/DF02 will affect the sequestration of IE by releasing already bound IE and block further binding of IE. Sevuparin/DF02 is expected to release late stage parasites into pheriperal blood, Sevuparin/DF02 also inhibits rosette formation *in vitro* and has also been found to inhibit the entrance of the merozoite into the red cell, being yet another possible way by which Sevuparin/DF02 may affect parasite growth and sequestration.

1.2 Name and Description of the Investigational Product

Name of investigational product: Sevuparin/DF02

DF02 has recently been assigned an INN name: *sevuparin sodium* (9041-08-1), DF02 is the same as sevuparin throughout the text.

Chemical Name:

Sevuparin/DF02 is a depolymerized form of heparin with a projected average molecular weight of 6.5 – 9.5 kDa and is prepared from porcine heparin of PhEur/USP quality. This preparation involves selective oxidation of non-sulfated uronic acid residues in heparin by periodate, including the glucuronic acid moiety in the pentasaccharide sequence that binds AT. Disruption of the structure of this residue annihilates the high-affinity interaction with AT and, consequently, the anticoagulant effect (measured as a-FXa or a-FIIa). Subsequent treatments by acid results in cleavage of the polymer at the sites that has been oxidized by periodate. Together, these manipulations lead to a loss of anticoagulant activity along with adequate depolymerization of the heparin chain.

Both heparin and LMWH are built up of repeating disaccharide units containing one uronic acid residue (D-glucuronic or L-iduronic acid, UA) and one D-glucosamine moiety (GlcN) that is either N-sulfated or N-acetylated. These sugar residues may be further O-sulfated, at the C-6 and C-3 positions in the case of glucosamine and the C-2 position of the uronic acid. The structure of heparin is variable regarding distribution of UA and sulfate residues; a representative partial sequence is shown in Figure 1.2.F1 (which also illustrates the mode of numbering of carbon atoms in a monosaccharide residue). Figure 1.2F2 shows the predominant structure of Sevuparin.

Figure 1.2.F1. Representative Example of Heparin Sequence

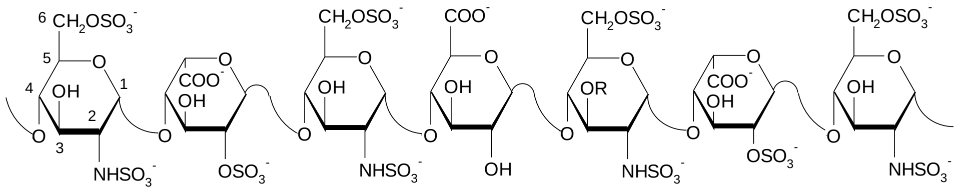

Figure 1.2.F2. Predominant Structure of Sevuparin/DF02

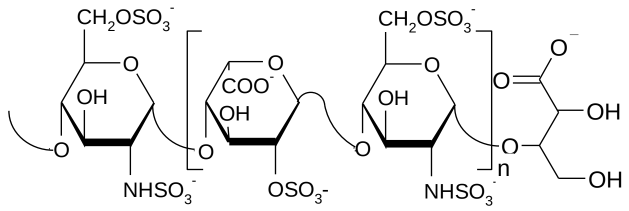

1.3 Pre-Clinical Pharmacology Study Results

Table 1.3.T1. *In Vitro* and *In Vivo* Studies

Release of sequestered malaria parasites upon injection of a glycosaminoglycan	Vogt et al. (2006)<br>PLoS Pathog 2(9):e100. DOI: 10.1371/journal.ppat.0020100	LAH (and Sevuparin/DF02) blocked sequestration, rosetting and adherence to endothelial cells, as well as inhibited merozoite invasion in a dose dependent manner and the inhibition was more than 80%. The inhibitory effects of the Sevuparin/DF02 were found to be equal to those of standard heparin. In the rat model, an injection of the substance together with the infected erythrocytes (IE) blocked up to 80% of IE from binding in the lung of the rat. When instead IE were injected and allowed to bind and after which the animals were treated with LAH up to 60% of the previously sequestrated IE was found to be released by the treatment. Similar results were obtained with Sevuparin/DF02
Whole body imaging of sequestration of Plasmodium falciparum in the rat	Pettersson F, Vogt AM, Jonsson C, Mok BW, Shamaei-Tousi A, et al<br>Infect Immun 73: 7736–7746	The paper describes the animal model (rat model) for in vivo evaluation of LAH

Table 1.3.T2. Pre-Clinical Pharmacology Study Results

Study/Species/GLP Status	Dose (mg/kg) and/or Concentration	Report Number (Ref No)	Summary
Efficacy of DF01 and Sevuparin/DF02 in a Sprague-Dawley template bleeding model<br>Male Sprague-Dawley rats<br>Final report	DF01: 7.0; 35;105; 350; and 700 mg/kg<br>Sevuparin/DF02: 7.0; 35;105; 350; and 700 mg/kg<br>Heparin 0.7; 1.5; 3.5 and 7.0 mg/kg	166-003	The results of the bleeding time analysis, the APTT and the PT analysis demonstrate that DF01 and Sevuparin/DF02 have less prominent anti-coagulating properties than heparin and Fragmin. Hence DF01 and Sevuparin/DF02 have lower anti-coagulating impact on natural haemostasis than the two classic anti-coagulating.
Anti-haemostatic and anti-coagulation efficacy of Sevuparin/DF02combination toxicology studies with Quinine and artemisinin.<br>8 male rats	Quinine 30 mg/kg<br>Sevuparin/DF02 35 mg/kg<br>Sevuparin/DF02 105 mg/kg<br>Artemisinin 70mg/kg	166-005	The objective of the study was to assess the anti-haemostatic and anti-coagulation efficacy of Sevuparin/DF02 when dosed in combination with either 30 mg/kg Quinine or 70 mg/kg Artemisinin.
Anti-haemostatic and anti-coagulation effects of Sevuparin/DF02 in juvenile rats.<br>Male Sprague-Dawley rats<br>14 days ± 1 day	Sevuparin/DF02 7, 35, 70, 105 mg/kg	166-006	A dose-related increase in the average bleeding time and APTT was observed from 35 and 7mg/kg of Sevuparin/DF02 respectively. The NOEL for an effect on bleeding time was therefore 7mg/kg and the NOEL for an effect on APTT was <7mg/kg.
Effect on Cardiovascular Parameters after intravenous administration to the beagle dog<br>Sevuparin/DF02 in adult dogs.<br>Female Beagle Dog, Marshall Europe	Sevuparin/DF02 0, 4, 20, 60 mg/kg	Accelera Report No 0469-2007-R (20)	Arterial blood pressure was moderately but transiently increased at the dose of 60 mg/kg and was recovered by 1.5-2 hours after treatment. The no-observed-effect level (NOEL) of DF02 on ECG intervals, heart rate and body temperature in the beagle dog is 60 mg/kg. A moderate effect on blood pressure was observed at 60 mg/kg, with a NOEL at 20 mg/kg.

1.4 Toxicology

1.4.1 Pre Clinical Toxicology

Table 1.4.1.T1. Pre-Clinical Toxicology Study Results

Study/Species/GLP Status	Route	Dose (mg/kg) and/or Concentration	Report Number (Ref No)	Summary
7-Day Intravenous Toxicity Study in the Rat	IV bolus	4, 20, 60 mg/kg	Nerviano Medical Sciences 0441/2007	Sevuparin/DF02, given intravenously for seven consecutive days to Sprague Dawley rats as a daily intravenous bolus at the doses of 0, 4, 20 and 60 mg/kg/day, was well tolerated. Treatment related effects on coagulation were recorded in that activated partial thromboplastin time was prolonged with dose-relationship at 20 and 60 mg/kg/day. The dose of 60 mg/kg/day can be considered as the maximum tolerated dose (MTD), and the dose of 4 mg/kg/day as the no adverse effect level (NOAEL).
14.Day study / rat / GLP	IV bolus	4, 20, 60 mg/kg	Accelera Report No 0022-2008-R (29)	Intravenous bolus at the doses of 0, 4, 20 and 60 mg/kg/day, caused a dose-dependent elevation of APTT two hours after last dosing in animals of both sexes at 20 and 60 mg/kg/day. In addition, histopathological changes were seen at the end of the treatment period in the femur of a few animals of both sexes treated with 20 and 60 mg/kg/day, in mandibular and mesenteric lymph nodes of animals of both sexes at all doses tested with increased frequency and severity at 20 and 60 mg/kg/day, and in the injection sites of animals of both sexes at all doses without a clear-cut dose relationship. At the end of the observation period the APTT values were normal while minimal histopathological changes persisted in the femur and lymph nodes of few high dose animals. The systemic exposure was dose-dependent with no gender deviation on Day 14 of study. Under the experimental condition applied in the present study the dose of 4 mg/kg/day was considered the no adverse effect level (NOAEL).
7-Day study /dog/ nonGLP	IV bolus	4, 20, 60 mg/kg	Accelera Report No 0480-2008-R (30)	Sevuparin/DF02, given for seven consecutive days to beagle dogs as a daily intravenous bolus injection at the doses of 4, 20 and 60 mg/kg/day, caused a dose-dependent elevation of APTT at 2 hours after dosing starting from 20 mg/kg. No other treatment-related effects were

Study/Species/GLP Status	Route	Dose (mg/kg) and/or Concentration	Report Number (Ref No)	Summary
				observed. In this study the dose of 4 mg/kg/day (corresponding to AUC0-t (last) after 7 Days of treatment of 3.35 or 6.75µg·Hours /mL in male and female animals, respectively) was considered the no adverse effect level (NOAEL) and 60 mg/kg/day can be considered the maximum tolerated dose (MTD).
14-Day study / dog / GLP	IV bolus	4, 20, 60 mg/kg	Accelera Report No 0025-2008-R (31)	No mortality occurred in any group. No meaningful clinical signs were seen up to 20 mg/kg/day. At the dose of 60 mg/kg/day episodes of soft stool or diarrhoea and reduced food consumption were seen in two males and two females. No treatment-related effects on body weight or changes in ophthalmoscopy, electrocardiographic evaluation, hematology, clinical chemistry or urine parameters were seen during the study. A dose-dependent, slight to moderate elevation of activated partial thromboplastin time (APTT) was recorded at 2 hours after the fourteenth treatment in animals of both sexes at 20 and 60 mg/kg/day. The mean elevation of APTT was 1.6-fold and 3.3-fold at 20 and 60 mg/kg/day, respectively. Values were normal on Day 28 of study.<br>Under the experimental condition applied in this study, the dose of 4 mg/kg/day in the absence of any clear treatment-related effect, was considered the no adverse effect level<br>(NOAEL).

1.4.2 Toxicokinetic Studies

Table 1.4.2.T1. Systemic Exposure of Sevuparin/DF02 on Day 14 after IV 4, 20 and 60 mg/kg/day to Male and Female Sprague Dawley Rats

Gender	Study Day	Dose mg/kg/day	C_{max} (µg/mL)		AUC0-t(last) (µg·Hours/mL)	
			mean	SD	mean	SD
**Male**	14	4	47.9	20.8	50.7	50.0
		20	112	7.00	173	54.6
		60	691	46.2	865	276
**Gender**	**Study Day**	**Dose mg/kg/day**	C_{max} (µg/mL)		AUC0-t(last) (µg·Hours/mL)	
			mean	SD	mean	SD
**Female**	14	4	28.4	24.2	22.7	16.0
		20	173	63.8	164	83.1
		60	1020	496	904	220

Table 1.4.2.T2. Sevuparin/DF02 Toxicokinetic Parameters in Dogs Treated Once Daily with Sevuparin/DF02

Gender	Study Day	Dose mg/kg/day	Cmax (µg/mL)	Tlast (Hours)	AUC0-t(last) (µg·Hours/mL)
			**Mean**	**Median**	**Mean**
Male	1	4	18.1	0.333	5.71
		20	105	1	77.2
		60	490	6	433
	14	4	76.6	0.333	15.2
		20	98.3	1	63.6
		60	757	6	541
Gender	**Study Day**	**Dose mg/kg/day**	**Cmax (µg/mL)**	**Tlast (Hours)**	**AUC0-t(last) (µg·Hours/mL))**
			**Mean**	**Median**	**Mean**
Female	1	4	25.0	1	10.7
		20	249	3	131
		60	652	6	408
	14	4	20.1	1	9.01
		20	156	1	96.0
		60	1010	6	613

1.4.3 Clinical Toxicology

Dilafor has evaluated Sevuparin/DF02 in a phase I trial in healthy volunteers. The study: "A single centre, double-blind, randomized, placebo-controlled, phase I study to evaluate the safety, tolerability, pharmacokinetics and pharmacodynamics of depolymerized heparin compound Sevuparin/DF02 following a single and multiple intravenous doses in healthy male volunteers" was a first-in-man study.

A total of 15 TEAEs were reported for a total of 8 subjects in part 1 of the study (table 1.4.3.T1), while a total of 18 TEAEs were reported for a total of 12 subjects in part 2 of the study (Table 1.4.3.T2).

Table 1.4.3.T1. Treatment Emergent Adverse Event by System Organ Class – Part 1

	**Cohort 1 n (%)**			**Cohort 2 n (%)**			**Cohort 1 & 2 n (%)**
	**10mg**	**90mg**	**360mg**	**30mg**	**180mg**	**420mg**	**Pooled placebo**
Subject treated	5	6	6	6	6	6	11
Subjects with TEAEs	2	2	2	1	2	2	2
**Gastrointestinal disorders**		**1(16.7)1**	**1(16.7)1**				
Diarrhea		1(16.7)1					
Toothache			1(16.7)1				
**General disorders and administration site conditions**		**1(16.7)2**				**1(16.7)1**	
Chills		1(16.7)1					
Feeling hot		1(16.7)1					
Thirst						1(16.7)1	
**Infections and infestations**				**1(16.7)1**		**1(16.7)1**	**1(9.1)1**
Nasopharyngitis				1(16.7)1		1(16.7)1	1(9.1)1
**Investigations**	**1(20)1**						
Hepatic enzymes increased	1(20)1						
**Nervous system disorders**	**1(20)1**	**1(16.7)1**			**1(16.7)1**		**1(9.1)1**
Dizziness							1(9.1)1
Dizziness postural					1(16.7)1		
Headache		1(16.7)1					
Hypoesthesia	1(20)1						
**Skin and subcutaneous tissue disorders**			**1(16.7)1**		**1(16.7)1**		
Blister			1(16.7)1				
Dry skin					1(16.7)1		

The most common TEAE reported in part 1 was nasopharyngitis, 3 events reported for 3 individual subjects. The most common TEAEs reported in part 2 were somnolence, 4 events reported by 3 subjects and increased in hepatic enzyme, 2 events reported in 2 subjects.

The majority of the TEAEs reported during the study were mild. In part 1, two moderate TEAEs were reported, “feeling hot” and “chills” on Day 6 in Period 2 for one subject. In part 2, one moderate TEAE was reported, “increased hepatic enzymes” were reported in one subject on Day 4, following multiple dosing with 180mg of Sevuparin/DF02 (Day 1 to 3). This event was considered probably related to Sevuparin/DF02 and resolved without the need for concomitant medication.

No subjects in parts 1 and 2 had any clinically relevant bleeding during the study period. Thus no protamine sulphate was administered as rescue medication.

The majority of the TEAEs reported during the study were not considered related to Sevuparin/DF02. In part 1, 4 TEAEs considered possibly/probably related to Sevuparin/DF02 were reported in 4 subjects as follows:

- One subject from cohort 1: “increased hepatic enzyme’ on Day 13 in Period 1, following administration of 10mg Sevuparin/DF02. This event was considered mild and possibly related to Sevuparin/DF02 and resolved with sequelae. Hepatic enzyme levels were still elevated on Day 1 in Period 2. Values of ALT, AST and GGT were considered clinically significant and the subject was withdrawn from the study.
- A second subject in cohort 1: diarrhoea on Day 1 in Period 2, following administration of 90mg Sevuparin/DF02. This event was considered mild and possibly related to Sevuparin/DF02. This event resolved without the need for concomitant medication/therapy.
- A third subject in cohort 1: hypoaesthesia on Day 1 Period 1, following administration of 10mg Sevuparin/DF02. This event was considered mild and possibly related to Sevuparin/DF02. This event resolved without the need for concomitant medication/therapy.
- One subject in cohort 2: dizziness (postural) on Day 1 Period 2, following administration of 180mg Sevuparin/DF02. This event was considered mild and possibly related to Sevuparin/DF02. This event resolved without the need for concomitant medication/therapy.

Table 1.4.3.T2. Treatment Emergent Adverse Event by System Organ Class – Part 2

	Cohort 1 n (%)	Cohort 2 n (%)	Cohort 1 & 2 n (%)
	**180mg**	**360mg**	**Pooled placebo**
Subject treated	6	6	4
Subjects with TEAEs	3	4	4
**Gastrointestinal disorders**			**2(50)2**
Diarrhea			1(25)1
Food poisoning			1(25)1
**General disorders and administration site conditions**			**1(25)1**
Application site pain			1(25)1
**Investigations**	**2(33.3)2**		
Hepatic enzymes increased	2(33.3)2		
**Musculoskeletal and connective tissue disorders**	**1(16.7)1**	**1(16.7)1**	
Pain in extremity	1(16.7)1	1(16.7)1	
**Nervous system disorders**	**1(16.7)1**	**3(50)4**	**1(25)1**
Dizziness	1(16.7)1		
Dysgeusia		1(16.7)1	
Somnolence		2(33.3)3	1(25)1
**Respiratory, thoracic and mediastinal disorders**		**1(16.7)1**	
Oropharangeal pain		1(16.7)1	
**Skin and subcutaneous tissue disorders**		**1(16.7)1**	**2(50)2**
Blister			1(25)1
Skin irritation		1(16.7)1	
Skin edema			1(25)1
**Vascular disorders**		**1(16.7)1**	
Hematoma		1(16.7)1	

E: number of events, n: number of subjects, SAE: serious adverse event, TEAE: treatment emergent adverse event

In part 2, 10 TEAEs were considered related to Sevuparin/DF02 and reported in 8 subjects as follows:

- First subject in cohort 1: increased hepatic enzyme on Day 4 in Period 1 following the administration of 180mg Sevuparin/DF02. This event was considered mild and probably related to Sevuparin/DF02.
- Second subject in cohort 1: increased hepatic enzyme on Day 4 in Period 1, following administration of 180mg Sevuparin/DF02. This event was considered moderate and probably related to Sevuparin/DF02.

- First subject in cohort 2: Two reports of somnolence in the morning and evening of Day 1 in Period 1, following the administration of 360 mg Sevuparin/DF02. Both events were considered mild and possibly related to the investigational product.
- Second subject in cohort 2: pain in extremity on Day 1 in Period 1, following administration of 360 mg Sevuparin/DF02. This event was considered mild and possibly related to Sevuparin/DF02.
- Third subject in cohort 2: somnolence on Day 1 in Period 1, and hematoma on Day 6 in Period 1, following administration of 360mg of Sevuparin/DF02. Both events were considered mild and possibly related to Sevuparin/DF02.
- Fourth subject in cohort 2: dysgeusia on Day 3 in Period 1, following the administration of 360mg of Sevuparin/DF02. This event was considered mild and possibly related to Sevuparin/DF02.
- Fifth subject in cohort 2: somnolence on Day 1 Period 1, following the administration of placebo. This event was considered mild and possibly related to the investigational product.
- Sixth subject in cohort 2: diarrhoea on Day 3 in Period 1, following administration of placebo. This event was considered mild and possibly related to the investigational product.

All events resolved without the need for concomitant medication/therapy.

One subject was withdrawn from the study. The subject was withdrawn in Period 2 of part 1 of the study due to elevated hepatic enzyme results on Day 13 in Period 1. Clinically significant elevated ALT, AST and GGT values were noted on Day 1 in Period 2.

No SAEs were reported during the study.

No deaths were reported during the study.

1.5 Summary of Clinical Data

The study was conducted in two parts, in a single study centre in the United Kingdom. Part 1 employed a double-blind, randomized, placebo-controlled, parallel group, of a single ascending dose (SAD). The primary objective of part 1 was to determine the safety and tolerability of Sevuparin/DF02 following i.v. administration of a single ascending dose and to estimate the maximum tolerated dose. The secondary objective was to determine the pharmacokinetics and the pharmacodynamics of Sevuparin/DF02 by measurements of activated partial thromboplastin time (APTT), prothrombin time (PT), bleeding time and platelet function and time to normalization of APTT after a single iv administration of Sevuparin/DF02.

Part 2 of the study employed a double-blind, randomized, placebo-controlled, multi-dose ascending (MAD) design. The primary objectives of part 2 were similar to part 1 with the addition of possible accumulation of APTT and plasma concentration before first and last dose evaluated. A total of 9 doses were administered in part 2 of the study.

In each part of the study, subjects were randomized to two cohorts, in each cohort, subjects were given various doses of Sevuparin/DF02 and randomly assigned to treatment with either Sevuparin/DF02 (n=6) or placebo (n=2).

In part 1, after screening, subjects underwent 3 treatment periods with a washout period of 12 days between treatments (within each cohort) and a final follow-up visits (see figure 1.5.F1).

The ascending doses administered to the subjects in part 1 are outlined in Table 1.5.T1. All doses were administered via a 5 minutes iv infusion in the morning of Day 1 (one subject was treated with Sevuparin/DF02 while a second was administered the placebo). Remaining subjects were only dosed after 24 hours post-dose assessments of these two subjects and the tolerability profile was determined as acceptable. Sequential dosing of subjects within a cohort was staggered such that there was at least 15 minutes between dosing of individual subjects. At least 6 subjects were dosed at each dose level before progressing to the next higher dose.

Table 1.5.T1. Part 1 Dosing Design

	Cohort 1	Cohort 2
Treatment period 1	10mg of Sevuparin/DF02 or placebo	30mg of Sevuparin/DF02 or placebo
Treatment period 2	90mg of Sevuparin/DF02 or placebo	180mg of Sevuparin/DF02 or placebo
Treatment period 3	360 mg of Sevuparin/DF02 or placebo	480*mg of Sevuparin/DF02 or placebo

*One subject each received 480 mg SevuparinDF02 and placebo as planned; remaining subjects received 420 mg DF02 or placebo.

Figure 1.5.F1. Part 1 Study Design

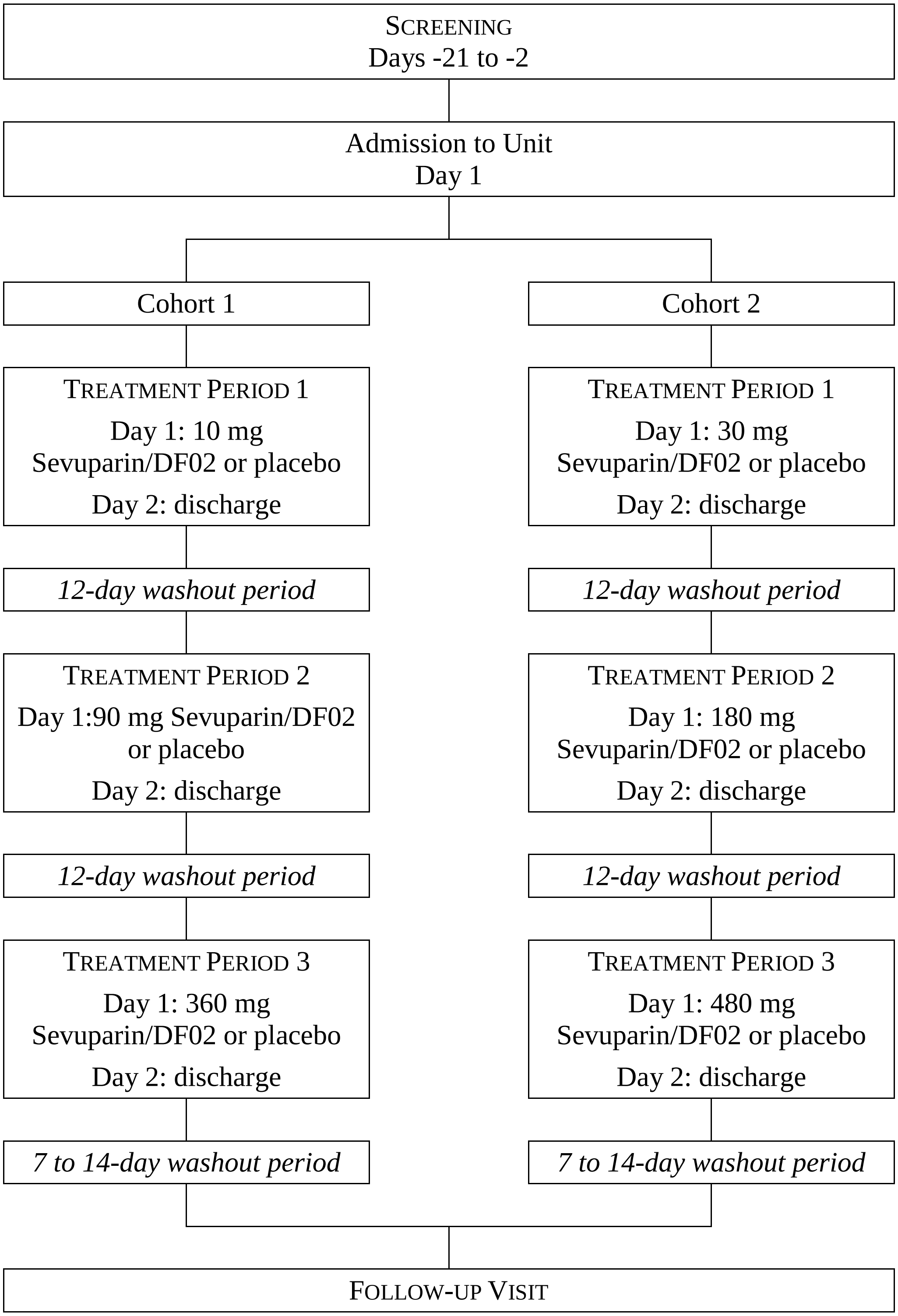

In part 2, after screening, subjects had only one treatment period and a final follow-up visit at completion of the study. Subjects in part 2 received repeated iv administration of Sevuparin/DF02 or placebo (figure 1.5.F2). The starting dose in part 2 was two steps below the MTD determined in part 1. Subjects received nine identical doses of Sevuparin/DF02 administered 6 hours apart over 5 minute infusions. Doses 1 to 3 were administered on Day 1, Doses 4 to 7 on Day 2, and Doses 8 and 9 on Day 3. The first cohort treated (cohort 1, dose 180mg) received a lower dose than cohort 2. The first dose was decided after review of safety and PK data from study part 1. The second dose level (cohort 2, dose 360mg) was decided after a review of part 2, cohort 1 data.

Sequential dosing of subjects within a cohort was staggered so that there was at least 15 minutes between dosing of individual subjects. Six subjects were dosed at each dose level before dose escalation was allowed.

Figure 1.5.F2. Part 2 Study Design

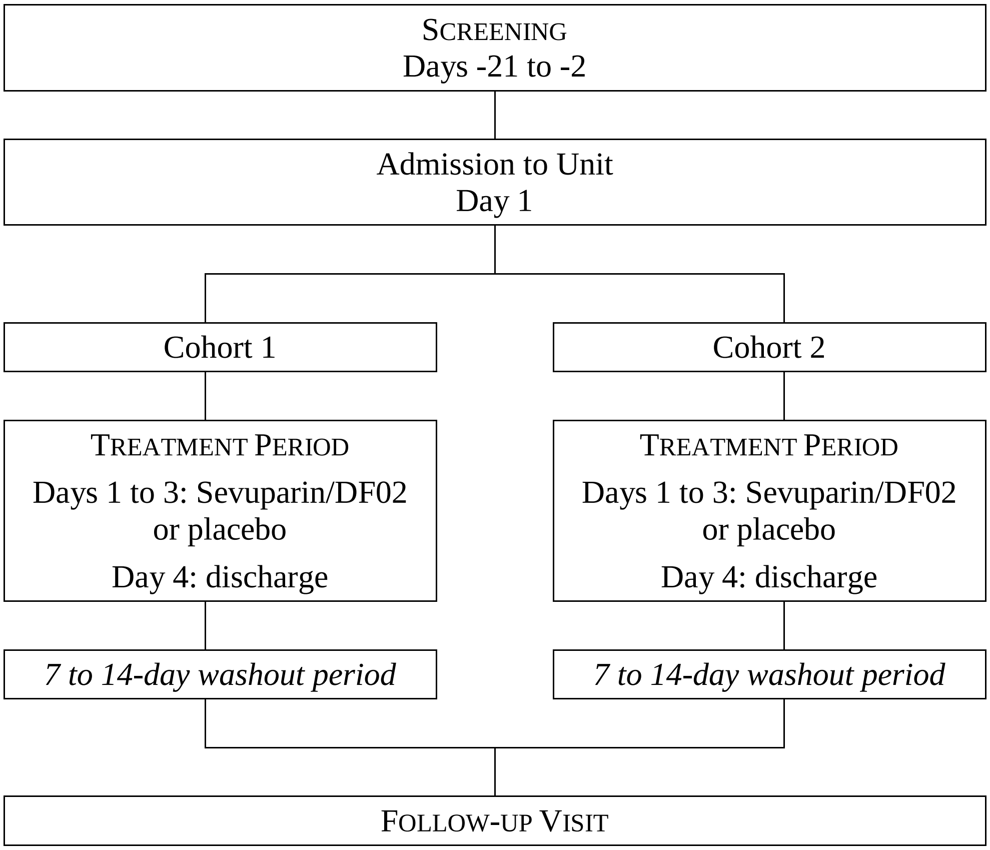

Subjects that participated in part 1 were not allowed to participate in part 2.

In part 1, subjects were in the study for 10 weeks while subject participating in part 2 were in the study for 6 weeks.

In part 1, a total of 17 (a subject in cohort 1 was replaced due to high liver enzymes on Day 1 of period treatment 2) subjects aged between 20 and 46 years old were enrolled, the mean age in cohort 1 was 26.1 and 28.9 in cohort 2 respectively.

In part 2, sixteen subjects were enrolled and randomized, their age varied between 22 and 50 with a mean of 28.7 in cohort 1 and 27.5 in cohort 2, respectively.

The pharmacokinetics profile of Sevuparin/DF02 was evaluated by measuring in:

Part 1: maximum observed plasma concentration (C_{max}), time corresponding to occurrence of C_{max} (t_{max}), terminal elimination rate constant (λ_z), apparent elimination half-life in plasma ($t_{1/2}$), area under the plasma concentration-time curve (AUC) from time zero to the last quantifiable concentration ($AUC_{0\text{-last}}$), AUC from time zero to infinity ($AUC_{0\text{-inf}}$) clearance (CL), and volume of distribution (V_z).

Part 2: Following doses 1, 5 and 9 (Days 1,2 and 3): C_{max}, t_{max}, λ_z, $t_{1/2}$, AUC in the dosing interval ($AUC_{0\text{-tau}}$), and $AUC_{0\text{-inf}}$. Additionally, following dose 9, volume of distribution at steady state (V_{ss}), clearance at steady state (CL_{ss}), % fluctuation, average plasma concentration (C_{avg}), and accumulation ratio (AR).

The pharmacodynamics effects of Sevuparin/DF02 were evaluated by measuring APTT, PT, bleeding time and platelet function.

The safety of Sevuparin/DF02 in this study was evaluated by monitoring adverse events, vital signs (blood pressure, heart rate and body temperature), cardiac monitoring (12-lead ECGs and telemetry) and clinical laboratories assessment (standard hematology and biochemistry laboratory parameters).

The Pharmacokinetics results are presented below for:

Part 1:

- Following single ascending 5 minute iv infusions of Sevuparin/DF02, mean C_{max} of 2.7, 8.9, 28.8, 53.0, 97.2 and 114µg/mL were recorded for doses of 10, 30, 90, 180, 360 and 420 mg, respectively. The increase was dose proportionate.
- The peak concentrations were achieved between 5 and 30 minutes after infusion start.
- The mean $t_{1/2}$ ranged from 0.66 to 1.0 hours and was dose independent.
- Exposure measured in terms of mean $AUC_{0\text{-last}}$ showed a dose proportionate increase from 7.1h.µg/mL (10mg dose) up to 140.h.µg/mL (420 mg dose).
- Sevuparin/DF02 clearance appeared independent of dose administered with means ranging for 2.58 to 2.99L/h.

Part 2:

At 180 mg 6-hourly

- Following nine multiple doses of 180 mg 6-hourly, plasma concentration versus time profiles after Infusions 1, 5 and 9 were almost super imposable, that is, no drug accumulation was observed. The mean AR was 1.03.
- Mean C_{max} was 47.5, 54.2, and 47.3µg/mL for Infusions 1, 5 and 9, respectively.
- Time to peak concentration (t_{max}) showed a similar range to that after single doses.
- Mean $AUC_{0\text{-}6}$ was 63.6, 76.4 and 66.2h. µg/mL for Infusions 1, 5 and 9.
- Means $t_{1/2}$ was similar to that after single doses, ranging from 0.87 to 1.24 hours.

- Clearance at steady state (CL_{ss}) was similar to that after single doses (mean 2.72/h).

At 360 mg 6-hourly

- Following nine multiple doses of 360mg 6-hourly, plasma concentration versus time profiles after Infusions 1, 5 and 9 were similar, only minimal drug accumulation was observed. The mean AR was 1.34.
- Mean C_{max} was 87.6, 80.7 and 104µg/mL for Infusions 1, 5 and 9.
- Time to peak concentration (t_{max}) showed a similar range to that after single doses and 180mg 6-hourly.
- Mean $AUC_{0\text{-}6}$ increased slightly after multiple doses: 105, 115, 141µg/mL for Infusions 1, 5 and 9, respectively confirming the small amount of Sevuparin/DF02 accumulation.
- Mean $t_{1/2}$ was similar to that after single doses and 180 mg 6-hourly, ranging from 0.86 to 1.21 hours.
- Clearance at steady state (CL_{ss}) was similar to that after 180mg 6-hourly (mean 2.55L/h).

The Pharmacodynamic results are presented below for:

Part 1.

- A linear dose-dependent increase in APTT was seen following single ascending Sevuparin/DF02 doses. The mean maximal change from baseline was 0.8, 2.0, 13.8, 54.2, 72.7 and 95.4 seconds following doses of 10, 30, 90, 180, 360 and 420 mg, respectively.
- Peak APTT response was 30 minutes post-dose, thereafter APTT reduced towards baseline with a median normalization time ranging from 2 hours post-dose (90mg dose) to 4 hours post-dose (420 mg dose).
- Prothrombin time showed a small increase from baseline following all Sevuparin/DF02 doses. The mean maximal response was between 0.5 and 2 hours post-dose and the magnitude of the response appeared to be dose independent, the maximum increase from baseline (1.2 seconds) occurring after a dose of 180 mg.
- Bleeding time increase marginally relative to placebo at doses up to 90mg but the increase was not seen consistently at higher doses. All individual subject post-dose bleeding times were within the range of 2 to 10 minutes.
- PFA-100 data showed a small increase from baseline following the lowest dose of 10mg, but not consistent increase was seen at higher doses.

Part 2.

- A linear, dose-dependent increase in APTT was seen following multiple 6-hourly iv of Sevuparin/DF02 doses of 180 mg and 360 mg. The mean maximal change from baseline was 41 and 110 seconds following the ninth infusion of Sevuparin/DF02 180 mg and 360 mg, respectively.
- A small build up in APTT was seen over the 3 days of treatment in both dose levels, that is, slightly larger increases from baseline were seen following Infusion 9 compared with Infusions 5 and 1. The mean maximal changed from baseline was 35 and 76 seconds following the first infusion of Sevuparin/DF02 180 and 360 mg, respectively.
- Peak APTT response was 30 minutes post-dose; thereafter the response reduced with a median normalization time of 5 hours after Infusion 9 (180 mg 6-hourly) and 6.5 hours after Infusion 9 (360 mg 6-hourly).

- Prothrombin time increases were small; mean maximum increases from baseline ranged from 0.7 seconds (180 mg 6-hourly) to 1.5 seconds (360 mg 6-hourly) for all infusions. The peak response was between 0.5 and 3 hours post-dose, with PT returning to baseline 6 hours after Infusion 9 (180 mg 6-hourly) and >24 hours after Infusion 9 (360 mg 6-hourly).
- Bleeding time showed an upward trend of 2 and 24 hours for the 360 mg dose following Infusion 9 only. No other bleeding time changes were evident.
- Platelet function tests demonstrated no apparent difference in change from baseline data following active treatment compared to placebo treatment.

The safety results are presented below:

- No dose-related trend was observed in the prevalence of treatment emergent adverse events (TEAEs) in part 1 and 2 of the study. However, a greater number of TEAEs overall were reported following multiple dosing in part 2 of the study, than following single dose administration in part 1 of the study. The majority of the TEAEs were considered mild and not related to Sevuparin/DF02. One TEAE leading to withdrawal was reported in part 1 of the study, elevated hepatic enzymes results following 10mg Sevuparin/DF02 in one subject. No SAEs were reported during the study TEAEs are summarized in tables 9.2.T2 and 9.2.T3.
- No dose-related or treatment related trends were observed in the median values of any biochemistry parameters or any hematology parameters other than APTT. A dose-related increase was observed in the median APTT values following single-ascending doses for Sevuparin/DF02 in part 1 and multiple doses of Sevuparin/DF02 in part 2 of the study. Activated partial thromboplastin time values above the reference range were observed in several subjects following treatment with Sevuparin/DF02 in parts 1 and 2, but values at the post-dose safety sample time points (2 and 5 hours post-dose) were not considered significant in any subjects. No urinalysis values of note were observed in any subjects during the study.
- There was no observable dose-related trend in any vital signs or 12-lead ECG parameters, and mean and median absolute values and change over time were similar for all treatment groups in parts 1 and 2 of the study. Twelve-lead ECG abnormalities were noted in 21 subjects overall, and abnormal ECG telemetry results in 3 subjects; none of these abnormalities were deemed to be clinically significant.
- No abnormal physical examination findings were observed in any subjects at follow-up in parts 1 and 2 of the study.

1.6 Rationale

Sevuparin/DF02 has been developed by Dilaforette for intravenous use as an adjunctive treatment in severe falciparum malaria. Its inhibitory effects on cytoadherence and rosette formation as shown *in vitro* and in animal models have the potential to interfere with a pivotal part of the pathophysiology of severe falciparum malaria, i.e. sequestration and impairment of microcirculatory flow in the microcirculation of vital organs. In contrast with other heparins, Sevuparin/DF02 is a low anticoagulant heparin derivate (LAH). Its low anticoagulant potency

has been confirmed in both preclinical studies and a phase I study in adult healthy volunteers. Based on the preclinical and clinical data, Sevuparin/DF02 has a good safety profile.

Before Sevuparin/DF02 can be tested in patients with severe falciparum malaria, the safety and proof of principle of the anti-adhesive effects of the drug should be tested in adult patients with uncomplicated falciparum malaria.

The current study is designed to determine the tolerability and pharmacokinetics of Sevuparin/DF02 when administered as an intravenous infusion in subjects affected with uncomplicated malaria in combination with Malanil® (atovaquone/proguanil) as anti-malarial treatment. The study will also assess the potential of Sevupatin/DF02 to reduce IE sequestration in patients and rosette formation.

If we can show that Sevuparin/DF02 is safe also for patients and that there is an effect on the *ex vivo* cytoadherance and rosetting in patients with uncomplicated falciparum malaria. The next step will be to try it out in patients with severe falciparum malaria. Sevuparin/ DF02 have then the potential to lower the number of patients dying from severe malaria.

The patients in this study may not receive additional benefit by participating in the study. However, the beneficial results will improve our understanding of malaria, and hope to reduce the mortality of severe malaria in the future.

1.6.1 Rationale for Dosage

The dose rational for the discussed study with Sevuparin/DF02, as an adjunctive therapy in subjects affected with uncomplicated falciparum malaria is based on generated non-clinical and clinical data.

Non- clinical data

Animal data shows that sevuparin has a low anticoagulant potency compared with heparin derived anticoagulant compounds. The dose-limiting toxicity is related to the remaining low anticoagulant potency of Sevuparin. There was no other serious toxicity observed in any of the safety pharmacology-, toxicity- or mutagenicity studies. A study assessing repeated-dose toxicity using very high doses of 20 and 60 mg/kg/day showed a prolongation in APTT prolongation of 1.5-fold and 3-fold respectively, in both rats and dogs, indicating dose-dependency with little species-dependency.

Clinical data

Since the anticoagulatory effects have shown to be the dose-limiting toxicity in the pre-clinical investigations, the effects on coagulation (APPT, PT, bleeding time) have been a main endpoint in the phase I studies in healthy volunteers. These studies are summarised in the protocol. A 6-hourly intravenous dose of 180 mg/kg studied in 8 healthy volunteers prolonged the APTT with a mean maximal change from baseline of 41 seconds The APTT was normalised at 5h post dose. With a dose of 360 mg the maximum change was 110.seconds and was returned to normal at 6.5 h. Mean changes from baseline peaked at 0.5 hours post-dose, declining thereafter to approach baseline again. Because of the prolongation in APTT the maximum dose in part 1 of the current study will be 6 mg/kg/dose.

Dose escalation

The dose escalation will be done by modified Fibbonaci scale, which allows for careful monitoring of side effects before exposing the next patient: Starting dose is 1.5 mg/kg/dose next dose is 3 and the maximum dosage will be 6 mg/kg/dose.

During dose escalation, side effects will be closely monitored, and the dose will not be increased further when any of the strictly defined stopping criteria are met.

Taken into account the >10-fold less anticoagulant potency (anti-FXa and anti-IIa) of Sevuparin/DF02 compared with heparin and LMWH, the dose of 1.5 mg/kg/dose is considered to have a sufficient safety margin for the intended patient population. This dose is therefore considered justified as a safe starting dose in adults 18 to 65 years old without a history of a bleeding tendency suffering from uncomplicated falciparum malaria disease.

The first dose administered will be 1.5 mg/kg/dose, then, doses will be escalated to 3 and 6 mg/kg/dose, respectively. A maximum total dose of 360 mg can be administered per dosing occasion.

1.6.2 Conduct of Trial

This clinical trial will be conducted according to the current revision which has its origin in the Declaration of Helsinki (Revised 59th WMA General Assembly, Seoul, October 2008 - Appendix 1). It will be conducted in compliance with this protocol, CFR parts 50, 56, and 312, Good Clinical Practice (CPMP/ICH/135/95), and designated Standard Operating Procedures.

2 TRIAL OBJECTIVES AND PURPOSES

2.1 Objectives

2.1.1 Primary Objective

Part 1:

- To characterize the safety, and dose-limiting toxicities (DLTs) for Sevuparin/DF02 when administered IV, repeatedly, in combination with defined antimalarial treatment in adult patients with uncomplicated falciparum malaria; prevalence, severity, drug-relatedness, seriousness of adverse events of Sevuparin/DF02.

Part 2:

- To assess the anti-adhesive properties of Sevuparin/DF02 measured as the ability to reverse and prevent sequestration of mature stage parasitized erythrocytes in adult patients with uncomplicated falciparum malaria.

2.1.2 Secondary Objectives

Part 1:

- To characterize the pharmacokinetic (PK) profile of Sevuparin/DF02 when administered intra-venous (IV), daily, repeatedly in combination with defined antimalarial treatment in adult patients with uncomplicated falciparum malaria.
- To determine the pharmacokinetic parameters of half-life, Cmax, AUC, Tmax of Sevuparin/DF02.
- To determine the area under the curve of late stage peripheral blood parasitaemia over time curve.
- To characterise the difference in sequestration of mature form P. falciparum parasites in patients treated with Sevuparin/DF02
- To determine *ex-vivo* the ability of Sevuparin/DF02 to reverse and prevent rosette formation of IE to uninfected erythrocytes and to block cytoadhernce of IE to endothelial cells.

Part 2:

- To characterize the safety, for Sevuparin/DF02 when administered IV, repeatedly, in combination with defined antimalarial treatment in adult patients with uncomplicated falciparum malaria; prevalence, severity, drug-relatedness, seriousness of adverse events of Sevuparin/DF02 will be recorded.
- To determine the pharmacokinetic parameters of half-life, Cmax, AUC, Tmax of Sevuparin/DF02, of the 10 first patients randomized to the Sevuparin/DF02 arm.
- To determine the area under the curve of late stage peripheral blood parasitaemia over time.
- To characterise the difference in sequestration of mature form P. falciparum parasites in patients treated with Sevuparin/DF02 compared to control group
- To determine *ex-vivo* the ability of Sevuparin/DF02 to reverse and prevent rosette formation of IE to uninfected erythrocytes and to block cytoadhernce of IE to endothelial cells.
- To characterize the safety, and dose-limiting toxicities (DLTs) for Sevuparin/DF02, when administered IV, repeatedly, in combination with defined standard treatment in adult patients with uncomplicated falciparum malaria. And the prevalence, severity, drug-relatedness, seriousness of adverse events of Sevuparin/DF02 will be recorded.

3 STUDY DESIGN

The study aim to assess safety, tolerability, and the anti-adhesive properties of Sevuparin/DF02, as an adjunctive therapy, measured as the ability to reverse and prevent sequestration of mature stage parasitized erythrocytes in adult patients with uncomplicated falciparum malaria.

The study is a two part study; part 1 is an open labelled dose escalation study and part 2 is an open labelled, active controlled, randomized study.

Part 1:

In this part a dose escalation will be made to reach an MTD.

When the first two dose levels in part 1 (1,5 and 3 mg/kg/dose) have been evaluated by the DSMB and assessed as safe, part 2 of the study will start recruitment on dose 3 mg/kg/dose. Recruitment to the last cohort in part 1 (6 mg/kg/dose) will continue in parallel with start of part 2.

There will be a safety report submitted to the ethical committees concerned based on results for the first two dose levels in part 1.

Part 2:

This part will start on recommendation by the DSMB, based on the evaluation of the two first dose levels administered in part 1 of the study.

After screening, the patients will be randomized to either receive 3 mg/kg/dose Sevuparin/DF02 as an adjunctive therapy in combination with defined anti-malarial medication (Malanil®: a fixed combination of atovaquone-proguinil) or to Malanil® alone. The dose of Sevuparin/DF02 will be based on the results from part 1.

When 10 patients in each treatment group (active/control) have been treated, an interim evaluation of safety and efficacy will be performed, including the data for the last cohort of part 1 (6 mg/kg/dose). Based on this evaluation it will be decided if the dose for the following patients should be increased or the study continued on 3 mg/kg/dose.

The antimalarial treatment in the study will be with oral Malanil® rather than an artemisinin combination therapy, as the artemisinin component could potentially severely obscure the pharmacodynamic effects of Sevuparin/DF02. The proposed pharmacodynamic effect of Sevuparin/DF02 is reversal and prevention of cytoadherence of mature form parasitized red blood cells to vascular endothelium causing their sequestration into the microcirculation. Artemisinins have indirectly an important effect on cytoadherence, since they kill young ring stage parasites, preventing their further maturation and subsequent sequestration into the microcirculation. Because of this, the sensitivity to observe an effect of Sevuparin/DF02 on sequestration in patients with uncomplicated malaria would be importantly reduced by combining the drug with artemisinin treatment. In contrast Malanil® only acts on parasite in the second half of their extra-erythrocytic development, and therefore does not prevent sequestration.

Figure 3.0.F1. Overview of Study Design and Dosing Regimen for all patients in Part 1

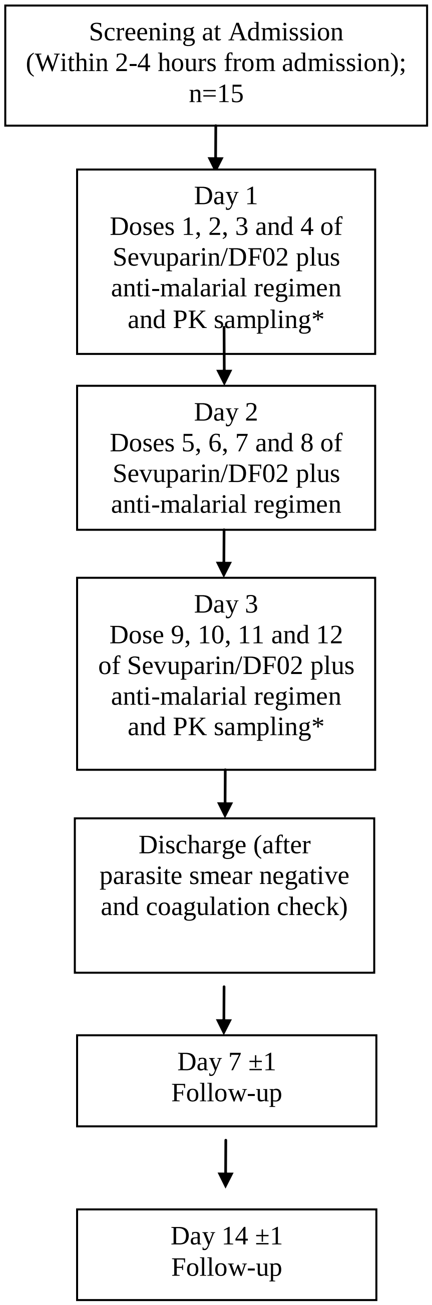

Figure 3.0.F2. Overview of Study Design and Dosing Regimen for patients in part 2 randomized to the Anti-malarial regimen (Malanil®) alone and Sevuparin/DF02 plus anti-malarial regimen (Malanil®)

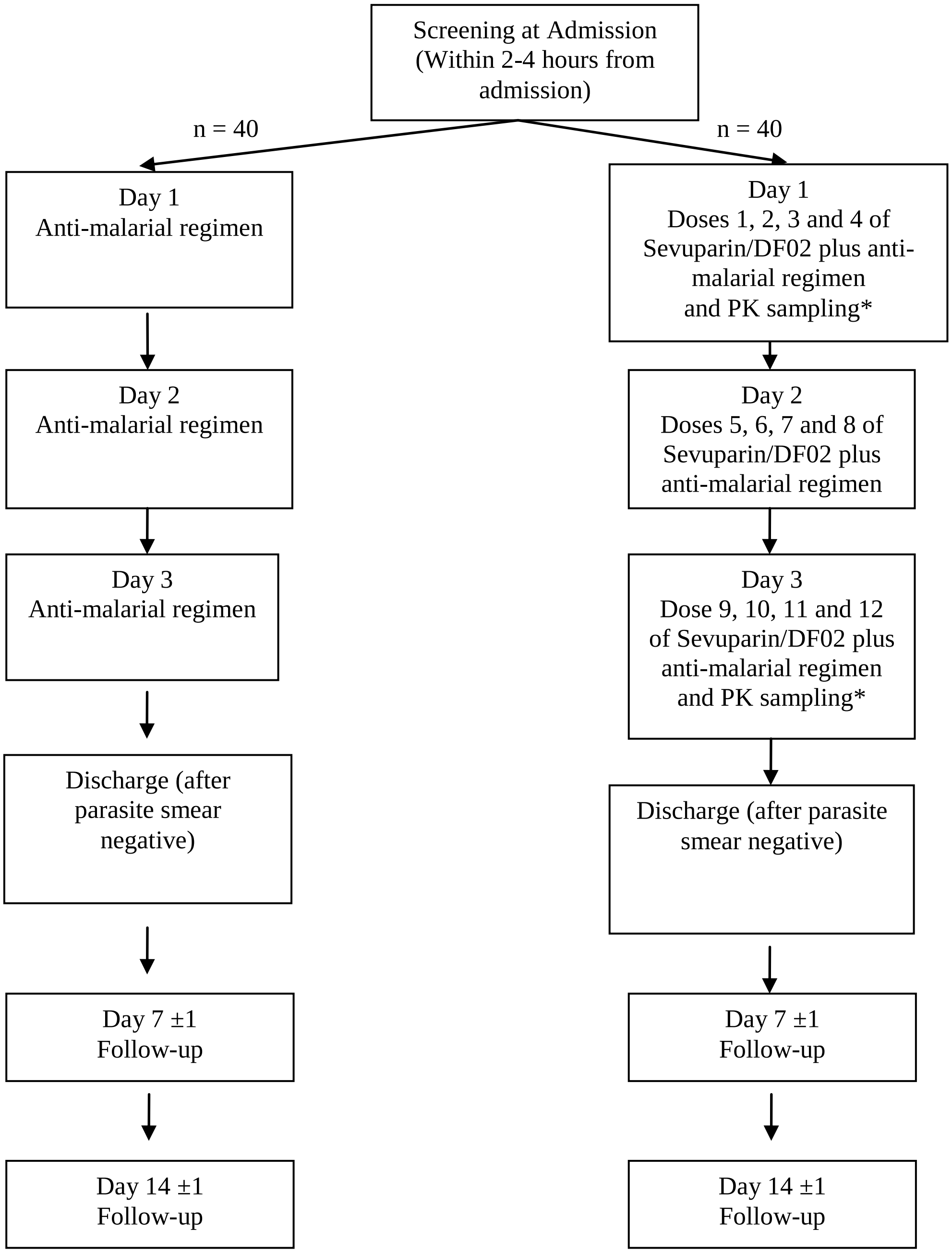

*Remark: *PK sampling of first 10 patients who are ramdomised to receive Sevuparin/DF02 plus anti-malarial regimen*

Figure 3.0.F3. Study Dose Escalation Scheme (Fibonacci Dose Escalation Scheme) in part 1

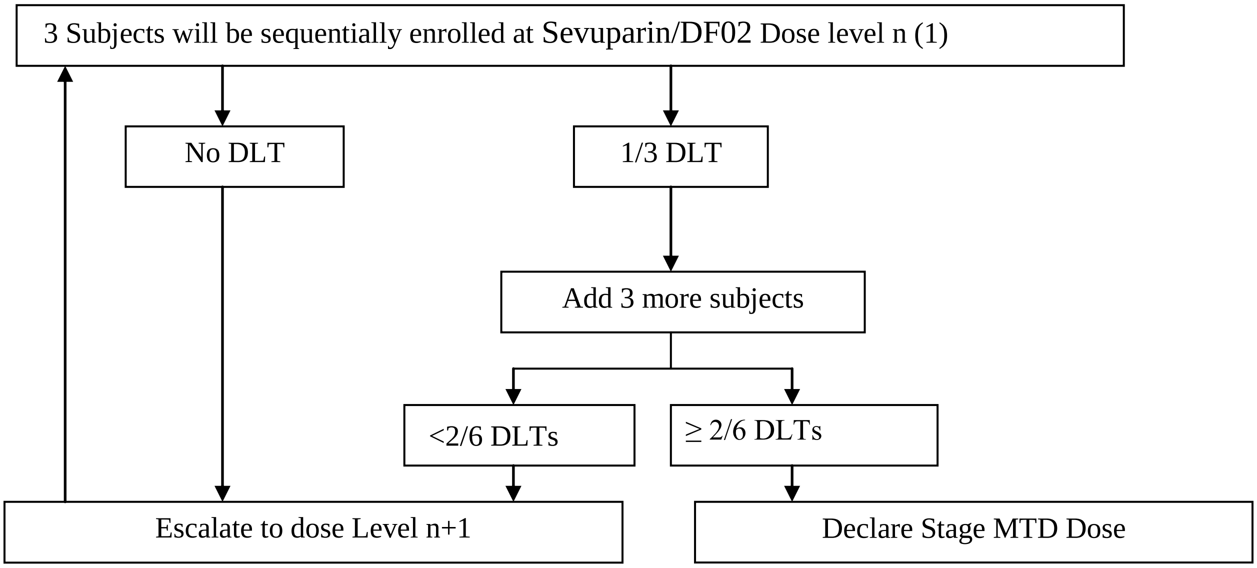

Incidence of Toxicity	Dose Escalation
DLT in 1 of 3 subjects	Add 3 subjects to the current dose
DLT in 1 of 6 subjects	Proceed to next dose level
DLT in a similar body system ≥ 2 subjects	Dose escalation not permitted
DLT in dissimilar body systems in ≥ 2 subjects	Hold and consult with MedicalMonitor/ Dilaforette AB and DSMB review.

Subjects will be enrolled in cohorts of three subjects each, and escalation of dose to the next cohort will be determined based on dose-limiting toxicity (DLT) in the previous cohort.

A minimum of 3 evaluable subjects will be entered at each dose level. For each dose cohort, a single patient will be enrolled and evaluated for SAEs for at least one week before enrolment of the next two subjects in the dose cohort. For the second and third subjects in the dose cohort, simultaneous enrolment is permitted. All subjects in a cohort must have been observed for a minimum of 14 days post treatment before escalation to the next dose level can occur. This means that the first subject is dosed and observed until day 7, and then another two patients may be admitted in paralelle. After all three patients have been observed for a minimum of 14 days an evaluation of the DLTs will be done by the Investigator and reported to the DSMB.

If 0 of 3 subjects experience DLT (see definition below) then the next dose level will be evaluated for the subsequent group of subjects. If 1 of 3 subjects experiences a DLT then 3 more subjects will be treated at the same dose level.

Dose limiting toxicity is defined as any adverse event (AE) of severity grade 3 or 4 (CTCAE v4.0; Grade 3 Severe or medically significant but not immediately life-threatening; hospitalization or prolongation of hospitalization indicated; disabling; limiting self care activities of daily life. Grade 4 Life-threatening consequences; urgent intervention indicated including serious or life-threatening, see Appendix 9.) considered possibly, probably or definitely related to Sevuparin/DF02 treatment, excluding the following:

- Events that occur and are completely resolved within 4-6 hours of the first dose of (infusion-related reactions)
- Grade 3 constitutional symptoms (fatigue, anorexia, diarrhoea) that are reversible to grade 2 or less with supportive care

3.1 Primary and secondary endpoints

3.1.1 Part 1

Primary endpoint

- Safety and tolerability; Prevalence, severity, drug-relatedness, seriousness of adverse events of Sevuparin/DF02 as adjunctive therapy to Malanil®
- Dose-limiting toxicities (DLTs) for Sevuparin/DF02 as adjunctive therapy to Malanil®

Secondary endpoints

- Pharmacokinetic parameters of half-life, Cmax, AUC, Tmax of Sevuparin/DF02 as adjunctive therapy to Malanil®

3.1.2 Part 2

Primary endpoint

- Area under the curve of the graph plotting the late stage peripheral blood parasitemia over time of sevuparin/DF02 as adjunctive to malanil in comparison to malanil® alone

Secondary endpoints

- Safety and tolerability; Prevalence, severity, drug-relatedness, seriousness of adverse events of Sevuparin/DF02 as adjunctive therapy to Malanil®
- Pharmacokinetic parameters of half-life, Cmax, AUC, Tmax of Sevuparin/DF02 as adjunctive therapy to Malanil®
- Area under the curve of late stage peripheral blood parasitaemia over time curve Sevuparin/DF02 as adjunctive therapy to Malanil® in comparison to Malanil group.

- Difference in the sequestration rates of the Sevuparin/DF02 as adjunctive therapy to Malanil® group in comparison to the Malanil® group

3.2 Concomitant Medication and Treatment

At study initiation, subjects should continue with their concomitant medications, as directed by their physicians. Note that medication affecting the coagulation must not be taken during the study.

Any medication or therapy received by the patient within 4 weeks of baseline, during the study, including over-the-counter, herbal preparations, supplements, prescription and non-prescription drugs, vaccinations or discontinued medications must be reported on the appropriate concomitant medication page(s) of the case report form (CRF). The information on the concomitant medication/therapy CRFs will include the name of the medication/therapy, dose, frequency, route of administration, dates of use (start and finish date), and indication for use. Subjects should be instructed not to take any medications including over-the-counter products, without first consulting with the Investigator. Any adverse event that results from taking a concomitant medication/therapy should be recorded on the CRF adverse event page. Additionally, any diagnostic, therapeutic or surgical procedure performed during the study period, should be recorded including date, indication, description of the procedure and any clinical findings.

3.2.1 Palliative and Supportive Care

Palliative and supportive care for disease-related symptoms will be offered as needed to all subjects in this study per the caring physician's judgment. These may be anti-emetics, anti-diarrhea agents, anti-abdominal cramping agents and blood products and blood transfusions as appropriate.

3.3 Restrictions

3.3.1 Prior Therapy

Participation in another clinical trial within 30 days is not allowed, and also patients who received any anti-malarial treatment within 7 days prior to enrolment including substance with anti-malaria activity, e.g., antibiotics or treatment with mefloquine within 30 days of enrolment.

3.3.2 Concomitant medication

Even though the anti coagulative activity of Sevuparin/DF02 is low, other medications that will affect the blood coagulation should be avoided.

Heparin, LMWH or Warfarin must not be used during the study drug medication.

Intake of 5-ASA (5- Aminosalicylic Acid) should be avoided preferably for four weeks prior to treatment.Treatment with acetaminophen (paracetamol) is recommended.

3.3.3 Fluid and Food Intake

There are no protocol-mandated restrictions with respect to ingested fluids and food.

3.3.4 Subject Activities and Restrictions

There are no specific restrictions in physical activities.

3.4 Randomization and Blinding

While the first part of the study (Part 1) is a dose escalation study, the second part (Part 2) is an open-label randomized study. Subjects will be randomized in the in 1:1 ratio. No blinding will be done as it is an open-label actively controlled study.

The additional PK sampling on 10 patients in part 2 will be done on the first 10 patients randomized to the Sevuparin/DF02 treatment arm at the Mae Sot General Hospital.

For the analysis of the *ex vivo* cytoadherance and rosetting samples, the researcher will be blinded to which treatment the patient have received. Assessment of peripheral blood slides will be performed by 2 independent microscopists, who are blinded to the study drug allocation.

3.5 Number of Subjects in the Study

It is assumed that the first stage, the MTD study (part 1) will require a recruitment of maximum 15 patients. For the extension study (part 2) an additional 80 patients will be recruited and randomized 1:1 to either arm reaching a total number of 95 subjects.

A sample size of 40 in the Sevuparin/DF02 treated group and 40 in the non Sevuparin/DF02 treated group allows detection of a difference of 0.5 logs on the AUC of the late-stage peripheral blood parasitaemia over time curve from log 10^5 parasites/µL/h to log $3X10^5$ parasites/µL/h between the treated and the control group, with an expected SD of 0.8 logs in both groups, significance level of 0.05 and a power of 80%.

3.6 Centers

Part 1 of the study is performed at Maesot General Hospital only. The second part of the study will be conducted at three study sites in Thailand;

- Maesot General Hospital,Tak province
- Mae Ramat Hospital,Tak province
- Hospital for Tropical diseases, Faculty of Tropical Medicine, Mahidol University, Bangkok

3.7 Population

Male or female subjects, 18 to 65 years of age (inclusive), presenting with acute uncomplicated *Plasmodium falciparum* malaria will be considered for enrolment. Uncomplicated malaria is

defined as febrile disease with a positive peripheral blood falciparum malaria slide and the absence of any severe malaria criteria (Appendix 2).

3.7.1 Subject Inclusion Criteria

Patients must meet all the following to be eligible for the study:

1. Male or female, 18 to 65 years old, inclusive
2. Presence of acute uncomplicated *P. falciparum* malaria, confirmed by positive blood smear with asexual forms of a single species (*P. falciparum*)
3. Counts of asexual forms of *P. falciparum*: 10 000- 100 000/ul with or without gametocytaemia
4. Presence of fever defined as $\geq$ 38°C tympanic temperature or a history of fever within the last 24 hours
5. Written informed consent
6. Willingness and ability of the patient to comply with study protocol for the duration of the study

3.7.2 Subject Exclusion Criteria

Subjects may not meet any of the following criteria to be eligible for the study:

1. Mixed infection with other Plasmodium species
2. History of significant bleeding (e.g. upper GI bleeding, recurrent epistaxis, joint bleeding, melena)
3. Patient with any criteria of severe or complicated malaria as defined by the World Health Organization, 2010 (Appendix 2)
4. Known history or evidence of clinically significant disorders: neurological, psychiatric (depression, psychosis or schizophrenia), cardiovascular (including arrhythmia), pulmonary, metabolic, gastrointestinal, endocrine diseases or malignancies.
5. For females: pregnancy, lactating or intention of becoming pregnant within the expected duration of the study
6. Patients with history of convulsions
7. Use of high doses aspirin (more than 100 mg/day) or dual anti-platelet therapy or use of heparin,LMWH or warfarin
8. Presence of significant anemia as defined by Hb <8 g/dL or Hct < 25%
9. A platelet count < 50,000/µL
10. Presence of febrile conditions caused by diseases other than malaria
11. Patients with abnormal laboratory result including:
    - Haematology: WBC<2000 or >12 000 cell/mcl

- Coagulation: PT and APTT>1.5 from upper normal range

12. Liver function tests (AST, ALT and alkaline phosphatase levels) more than 2.5 times the upper limit of normal range
13. Treatment with anti-malarial drug or antibiotics with anti-malarial activity within 7 days prior to enrolment or treatment with mefloquine within 30 days prior to current episode
14. Have a history of a clinically significant drug allergy to one or more of the study drugs, including sensitivity to heparin or LMWHs, history of hypersensitivity to the investigational medicinal product or any of the excipients or to medicinal products with similar chemical structures
15. Patients who have had splenectomy
16. Patients with known human immunodeficiency virus (HIV) infection
17. Participation in another clinical trial within 30 days is not allowed.

4 STUDY TREATMENT

4.1 Study Drug

Sevuparin/DF02 is a 5 mL overfill solution in a 10 mL vial at a 150 mg/ml concentration in a phosphate buffer 0.015M. The solution is in flint glass vial with a rubber stopper covered with a tear-off aluminium cap. Sevuparin/DF02 is to be administered intravenously, the vials can be stored refrigerated (2-8°C or 35.6 – 46.4°F). Once a vial is opened, the solution remaining in the vial must be used within 48 hours. The solution for administration prepared in each syringe must be kept refridgerated and used within 24 hours.

4.2 Study Therapy Administration –Sevuparin/DF02

The study therapy will be administered intravenously via an intravenous catheter in the forearm. This access vein will not be used for blood sampling. Note the cannula may **not** be flushed with heparin. Replacement of the intravenous catheter will be done according to local practice and the discretion of Investigator or designee, e.g., in the event of phlebitis. If a catheter is replaced it will be flushed with saline prior to removal unless there are signs of phlebitis.

In case of an immediate adverse reaction to the IV administration such as an anaphylactic reaction, a protocol is in place which will be followed strictly (Appendix 5: Infusion Reaction Criteria).

Note:
Epinephrine (1:1000) for subcutaneous injection, diphenhydramine (12.5 mg to 50 mg) for IV injection, and any other medications and resuscitation equipment for the emergency management of anaphylactic reactions will be available in the room where the infusions are being performed.

The study drug will be administered every 6 hours for 3 consecutive days. The subjects will receive a total of 12 doses of Sevuparin/DF02 in combination with their anti-malarial treatment.

During first 24 hours (day 1), subjects will receive 4 infusions of Sevuparin/DF02, on the second 24 hours (day 2) they will receive 4 doses of Sevuparin/DF02 and on third 24 hours (day 3) they will receive 4 additional short infusions of Sevuparin/DF02 .

After study therapy discontinuation, all subjects will be evaluated at two follow-up visits from the final study therapy administration.

4.2.1 Study Therapy Reconstitution

Sevuparin/DF02 should be prepared by a healthcare professional using aseptic technique. Please refer to Appendix 4 for the directions on how to calculate for the different doses to be administered to the subjects.

Dosing will be calculated on a mg/kg body weight basis and rounded to the closest 1 mg. A certified and study specific scales will be used. A maximum total dose of 360 mg can be administered per dosing occasion.

4.3 Selection and Timing of Dose for Each Subject

Sevuparin/DF02 will be administered to enrolled subjects as defined in the protocol ± 30 minutes.

4.4 Dose Modification

4.4.1 Dose Modifications of Sevuparin/DF02

There will be no dose reductions for Sevuparin/DF02 when administered in combination with anti-malarial care, for three consecutive days.

4.5 Packaging

Sevuparin/DF02 is supplied in 10 mL vials each containing 5 mL 150 mg/mL of Sevuparin/DF02 in a phosphate buffer 0.015M and to be stored at 2-8°C/ 35.6 – 46.4°F (refrigerated) until use. Sevuparin/DF02 should be protected from light and heat until use. Dosing is calculated to the nearest 1 mg for administration. Administration of Sevuparin/DF02 should be done within 24 hours from preparation.

Sevuparin/DF02 is supplied as individually boxed10 vials in each package.

4.5.1 Atovaquone-proguanil (Malanil®):

Malanil® is a fixed-dose combination of the antimalarials atovaquone and proguanil hydrochloride. The chemical name of atovaquone is trans-2-[4-(4-chlorophenyl) cyclohexyl]-3-hydroxy-1,4-naphthalenedione. The chemical name of proguanil is 1-(4-chlorophenyl)-2-(N'-propan-2-ylcarbamimidoyl) guanidine. Each coated tablet contains 250 mg atovaquone /100 mg proguanil. They are packaged in PVC aluminium foil blister pack/s containing 12 tablets. This medicinal product does not require any special storage conditions.

The mechanism of action of atovaquone is by inhibition of parasite mitochondrial electron transport at the level of the cytochrome bc_1 complex and collapse of mitochondrial membrane potential. Proguanil inhibits plasmodial dihydrofolate reductase (DHFR) primarily through its metabolite cycloguanil, which results in disruption of parasite DNA synthesis. The mechanism of synergy of proguanil with atovaquone is postulated to be by its biguanide mode of action rather than through its cycloguanil metabolite.

Subjects recieveing the combination therapy will be administered the Malanil® before the initial infusion of Sevuparin/DF02. On the following days; day 2 and 3, the dosing will be once per day in the morning. Subjects randomized to treatment with antimalarian medication only will recevive the Malanil® in the morning of day 1, 2 and 3.

4.6 Labelling

The labels will contain the information listed below.

The outer packaging of Sevuparin/DF02 will contain the following information:

150 mg/mL Sevuparin/DF02 SOLUTION
VIALS FOR RECONSTITUTION
Protocol # Sevuparin/DF02 _TSM02

Contents: 10 x 5 mL 150mg/mL Sevuparin/DF02 vials

Coordinating Investigator: Prof. Arjen M. Dondorp
Batch No #___________

Keep at temperature 2-8°C (35.6 – 46.4°F) refrigerated, protect from light
"CAUTION: ใช้เพื่อการวิจัยเท่านั้น For Clinical Research Use Only"
Dilaforette AB
Karolinska Institutet Science Park
Rezius väg 8, SE-171 65 Solna, Sweden
Telephone: +46 706444771

Each vial of Sevuparin/DF02 will have a label attached containing the following information:

150 mg/mL DF02 solution (5 mL) for IV administration
Dilute prior to administration as directed by protocol number Sevuparin/DF02_TSM02

Store at 2-8°C (refrigerated), protect from light

Batch No. ____________

Re-test date: __________

Patient No.____________

"CAUTION: ใช้เพื่อการวิจัยเท่านั้น For Clinical Research Use Only"

Dilaforette AB, Karolinska Institutet Science Park, Retzius väg 8, SE-171 65 Solna, Sweden.
Telephone +46-706-444771 Manufacturer: Apotek Produktion & Laboratorier AB, Formvägen 5B, SE-90621 Umeå, Sweden

4.7 Storage

Sevuparin/DF02 should be stored at temperature between 2-8°C. The reconstituted drug should be protected from light and heat until use. Malanil® does not require any specific storage conditions.

4.7.1 Destruction of Surplus Medication

At the conclusion of the study, all unused and used vials of Sevuparin/DF02 will be returned to the sponsor or designee. Alternately, drug may be destroyed on site as dictated by the appropriate standard operating procedure at the participating institution (if appropriate). Vials should only be destroyed or returned after drug accountability has been performed by the CRA and all vials were accounted for. If there is an error or perceived error, please return the vials to Dilaforette AB.

4.8 Storage and Accountability

4.8.1 Sevuparin/DF02

The study medication will be supplied by Dilaforette AB directly to the study hospitals and retrieved (unused medication and returned, used vials/packaging) at the end of the study. All transfers of study medication between the above mentioned pharmaceutical companies and the study hospitals will be documented.

All treatment doses will be observed, therefore, all unused medication will be collected by a member of the study team.

All study therapy must be kept in a secure place under adequate storage conditions.

The Investigator has overall responsibility for ensuring that study therapy is stored in a safe limited access location under the specified appropriate storage conditions. Limited responsibility may be delegated to a nominated pharmacy representative, but this delegation must be documented.

Study therapy will be dispensed by the hospital pharmacy or nominated member of the study team. The Investigator or designee will record dispensing of the study medication on a study medication accountability record. These records should include dates, quantities, batch/serial numbers, expiration dates (if applicable), and the unique code number assigned to the investigational product and trial subject. This record will be made available to clinical monitoring personnel for the purpose of accounting for the clinical study medication supply. A study medication supply inspection for inventory purposes and assurance of proper storage will be conducted as necessary. Any significant discrepancy will be recorded and reported to the sponsor and a plan for resolution will be documented.

4.8.2 Malanil®

The Malanil® will be purchased from GSK, Thailand.

4.9 Investigational Product Retention at Study Site and Destruction

The study drug Sevuparin/DF02 required for completion of this study will be provided by Dilaforette AB. The recipient will acknowledge receipt of the drug indicating shipment content and condition. Damaged supplies will be replaced. Accurate records of all study drug dispensed, used and returned will be maintained. At the conclusion of the trial, all unused and used vials will be returned to the sponsor or designee. Alternately, drug may be destroyed on site as dictated by the standard operating procedures at the participating institution (if appropriate). Vials should only be destroyed or returned after drug accountability has been performed by the CRA and all vials were accounted for. If there is an error or perceived error, please return the vials to Dilaforette AB.

4.10 Duration

Start Date: June 2011

End of Recruitment: November 2012

End of Treatment: November 2012

4.11 Table of study evaluations

Table 4.11.T1. Study Schedule of Evaluations

Evaluation	Screening	Day 1	Day 2	Day 3	Post dosing follow-up		Day 30
					Day 7 (±1 day)	Day 14 (±1 day)	
Consent[1]	X						
I/E Criteria	X						
Medical History[2]	X				X[6]	X[6]	
Physical Examination[3,4]	X	X[4]	X[4]	X[4]	X[4]	X[3]	
Tympanic temperature	X	X[5]	X[5]	X[5]	X	X	
Vital signs :HR, BP, resp rate	X	X	X	X	X	X	
Weight/Height	X						
12 Lead ECG[7]	X	X	X	X			
Parasitemia[8] (Malaria Blood Film)	X	X	X	X	X	X	
Cytoadherance, Rosette assay, and PfEMP1 activity		X[9]					
Pregnancy test (Urine)	X					X	
Coagulation: APTT,PT and antiXa	X	X[11]	X[11]	X[11]	X[11]	X[11]	
Haematology & Clinical Biochemistry	X[10]		X	X	X	X	
Sevuparin/DF02 Administration and/or Malanil® Standard of Care		X	X	X			
PK samples[12]		X		X			
Urine Sample for analysis	X						

**Evaluation**	**Screening**	**Day 1**	**Day 2**	**Day 3**	**Post dosing follow-up**		**Day 30**
AEs Assessment		X	X	X	X	X	
Concomitant Medications	X	X	X	X	X	X	
Telephone call to assess the pregnancy risk (only female)							X

[1]: To be obtained before any study-related activities are performed
[2]: Include a review of previous/ongoing medications for 30 days prior to enrolment, includes sex, age and race
[3]: Complete examination, including assessments of the skin, head, eyes, ears, nose, throat, neck, thyroid, lungs, heart, abdomen, lymph nodes, extremities and body weight
[4]: Partial examinations to update findings from the examination performed at screening
[5]: Pre dosing and every 6h post dose until parasite or fever negative
[6]: Updated medical history
[7]: ECG to be taken at pre-dose and then 1 hour after sevuparin doses 1 and 12.
[8]: Blood will be taken at pre dosing and post initial dosing at 30, 60, 90 min, 2, 3, 4, 6, 8, 10, 12 hrs and then every 6 hrs until parasite negative on the peripheral blood for the evaluation of haematocrit levels.
[9]-Rosette assay and PfEMP1 activity will be analyzed on blood samples from time pre dose.
[10] Blood group typing
[11] Only patients receiving Sevuparin/DF02
[12] Only patients part 1 and 10 first patients part 2 receiving Sevuparin/DF02 at the Mae Sot General Hospital. Note that the number of samples may be reduced in part 2, if indicated by the part 1 PK results.

Table 4.11.T2. PK Blood Samples, APTT, PT (coagulation), and antiXa Activity

Only for patients receiving treatment with Sevuparin/DF02

Time points [3] (Hr:Min)	Dose	PK blood sample[1]	APTT, PT and anti Xa[4]
Pre-dose 1		X	X
Dose 1(0:00)	X	X[2]	
0:06		X	
0:15		X	
0:30		X	
0:45		X	
1:00		X	
1:30		X	
2.00		X	X
3.00		X	
4.00		X	
5.00		X	X
Dose 2(6:00)	X		
11:00			X
Dose 3(12:00)	X		
17:00			X
Dose 4(18:00)	X		
23:00			X
Dose 5(24:00)	X		
29:00			X
Dose 6(30:00)	X		
32:00			X
35:00			X
Dose 7(36:00)	X		
41:00			X
Dose 8(42:00)	X		
47:00			X
Dose 9(48:00)	X		
53:00			X
Dose 10(54:00)	X		
59:00			X
Dose 11(60:00)	X		
65:00		X	X
Dose 12(66:00)	X	X[2]	
66:15		X	
66:30		X	
66:45		X	
67:30		X	
68:00		X	X
69:00		X	
70:00		X	
71:00		X	X

[1] All patients in part 1, and first 10 patients allocated Sevuparin/DF02 part 2 at Mae Sot General Hospital. Note that the number of samples may be reduced in part 2, if indicated by the part 1 PK results.

[2] Samples to be taken immediately after the end of the infusion

[3] ± 2 minutes for time points up to15 min post dose, ± 10 minutes for all time points above 15 min post dose

[4] Note that for the patients at the 6 mg/kg/dose cohort in part 1 coagulation samples should also be collected and analyzed at 1, 31 and 67 hours post first dose, according to the part 1 CRF.

4.12 Criteria for Stopping Treatment

(for further information on patient withdrawal see section 7, and DLT above)

- If on-site APTT is ≥1.5 times the upper limit of normal value (ULN) for samples taken 5 hours after administration of dose 1 or dose 5, the subject will be withdrawn from study treatment. Withdrawn subjects will be closely followed up.
- If the mean APTT across subjects in Cohort 1 (calculated after all subjects in that cohort had been dosed) is >3 times ULN on samples taken 2 hours after administration of dose 1 or dose 5, dose progression will stop.
- If the mean APTT value is >3 times ULN on samples taken 2 hours after administration of dose 12, or the mean value is close to 3 times ULN, the Sevuparin dose may only be adjusted after confirmation from the Chairman of the DSMB
- At the Investigator's discretion, the treatment may also be stopped for one or all subjects or the dose levels reduced based on the results of the APTT, antiXa and PTcoagulation analyses or any adverse event judged to be causally related to Sevuparin/DF02. Safety and pharmacodynamic assessments will continue until such time as indicated by the Investigator.

4.13 Rescue medication patients receving Sevuparin/DF02

If the APTT value was >3.5 times the ULN and clinically relevant abnormal bleeding is observed, protamine sulphate is allowed to be administered as rescue medication, at the discretion of the Principal Investigator. The dose of protamine sulphate administered has to reflect the dose of DF02, with 1 mg neutralising 1 mg of circulating DF02, up to a maximum dose of 50 mg by IV infusion at a rate of <5 mg/minute.

5 STUDY PROCEDURES

5.1 Informed Consent

All subjects are to give informed consent in accordance with the origins of the Declaration of Helsinki (Appendix 1) and CFR part 50 and ICH GCP guidelines.

The subject will sign the Informed Consent Form for part 1 or 2 depending on which part of the study the patient will participate in before she/he enters the study, i.e., before any study-related activities are performed. The subject will be given sufficient time to consider the study's implications before deciding whether to participate. If the person is unable to write, thumb print together with an impartial witnessed consent is permitted. Should the Investigator choose to produce his own information sheet then the detail provided by Dilaforette AB information sheet should be regarded as the minimum written explanation required. The format and content of the informed consent form must be agreed upon by Dilaforette AB, the Institutional Review Board /Independent Ethics Committee and regulatory authorities.

Should there be any amendments to the final protocol the subject or witness must agree to sign the amended ICF indicating that they re-consent to participate in the trial.

Written and verbal versions of the patient information and consent form will be presented to the patients in their language detailing no less than: the exact nature of the study; the implications and constraints of the protocol; the known side effects and any risks involved in taking part. It will be clearly stated that the patient is free to withdraw from the study at any time for any reason without prejudice to future care, and with no obligation to give the reason for withdrawal.

5.2 Subject Identification

5.2.1 Subject Number

When the subject has completed the screening process and enters into the trial, he/she will be assigned a unique subject number composed of a site identification number and unique subject identification sequence number. The subject number and the subject's initials will be used as identification for the whole duration of the study.

5.3 Medical History

Medical history will include age, sex, race, and subject medical and disease history.

5.4 Physical Examination

A complete physical examination will be conducted at screening/study entry. Partial physical examinations will be performed per schedule of events. Physical examination will be performed by site personnel who are experienced and routinely conduct physical examinations.

5.5 Vital Signs and Cardiovascular Assessments

Vital signs are defined as body temperature (via tympanic measurement), pulse, blood pressure (systolic and diastolic blood pressure) and respiratory rate.

The **body temperature**, measured by a tympanic thermometer, will be recorded at pre dosing and then every 6 hrs post dose until parasite negative on the peripheral blood or until no fever (temperature can influence the cyto-adherent properties of parasite infected RBCs).

Subjects **pulse and respiratory rates** are to be recorded prior the initiation of Sevuparin/DF02 infusion or Malanil®. They will also be recorded during follow-up visits on Day 7 and Day 14.

Blood pressure should be determined by cuff (manual or automated is acceptable although the same method should be used throughout the study).

Blood pressure (systolic and diastolic) and **pulse** will be recorded at screening (defined as baseline) and prior to, Sevuparin/DF02 IV infusion and every 6 hours thereafter until last Sevuparin/DF02 infusions. The time of the measurements will be noted. All measurements will be made with the subject in the supine position.

A standard 12 lead **ECG** will be recorded at screening (defined as baseline) and then 1 hour after sevuparin doses 1 and 12. The Investigator will provide an overall judgment of the ECG.

5.6 Dispensing Study Drug

Study drugs must be used only as directed in the protocol. The Investigator will be provided with forms to enable accurate, written records of all medication received from Dilaforette AB. These records will be kept by the Investigator or Pharmacist/nominated person. The Investigator or pharmacist/nominated person will also keep accurate records of the quantities dispensed and used of the investigational product.

At the end of the study all unused trial medication (i.e., vials) will be returned by the pharmacist/ nominated person to a nominated contractor on behalf of Dilaforette AB. All certificates of delivery and returns must be signed by the pharmacist/nominated person. Based on entries in the drug accountability forms, at the end of the trial, it must be possible to reconcile delivery records with those of usage and returned stocks. All study medication must be accounted for and all discrepancies documented appropriately.

5.6.1 Malanil®

The Malanil® will be purchased. The pharmacy will keep records on batch numbers and medication accountablility. The Investigator or designee will record dispensing of Malanil®on a medication accountability record. These records should include dates, quantities, batch/serial numbers, expiration dates (if applicable), and trial subject. This record will be made available to clinical monitoring personnel.

5.7 Clinical Laboratory Tests

5.7.1 Laboratory Parameters

Baseline Investigations

1. Haematology/ Complete blood count (CBC): Hct, Hb, MCH, MCHC, MCV, platelet count, red blood cell count, white blood cell count and blood group (3 ml)
2. Parasite count/ Malaria blood film: thick and thin film for assessing parasitaemia and staging (0.5 ml)
3. Blood for biochemistry : glucose, BUN, creatinine, creatine-kinase, total bilirubin, alkaline phosphatase, alanine aminotransferase, aspartate aminotransferase, electrolytes, calcium, lactate dehydrogenase (5 ml)
4. Coagulation: prothrombin time (PT), activated partial thromboplastin time (APTT), anti-Xa activity (1.5 ml)
5. Pharmacokinetic sample(predose) (3 ml)
6. Rosette formation (see in Appendix 8), and plasma for immunity activity (PfEMP1) (5 ml)
7. Urine pregnancy test: for females of childbearing potential (1 ml)
8. Urinanalysis: appearance, colour,specific gravity, glucose, ketones, protein, nitrite, bilirubin, blood, pH (5 ml)
9. Other blood tests depending on clinical condition and judgment of the treating doctor

Total blood volume for the baseline investigations is 18 ml and 6 ml for urine

5.7.2 Pharmacokinetics (PK)

Blood samples will be collected for pharmacokinetic assessments for subjects enrolled in Part 1: the MTD study and additionally 10 (ten) first patients randomized to Sevuparin from Part 2 at Mae sot General Hospital: the extension study will be included in the PK study.

Pharmacokinetic samples (non-heparinised CTAD Vacutainer tubes) (3 ml)

Blood samples for Sevuparin/DF02 PK Profile and APTT, PT, and antiXa will be measured according to time points listed in table 4.11.T2. The number of PK samples may be reduced in part 2, if indicated by the part 1 PK results.

Note: These samples must be collected from a different vein than the one used for drug infusion. The samples must be collected in non-heparinised CTAD Vacutainer tubes. A special laboratory manual is provided.

A sampling kit for determination of Sevuparin will be supplied by the Sponsor/Dilaforette AB. The sampling kit will contain a complete set up for the PK sample handling.

The pharmacokinetic profile of Sevuparin/DF02 administered as a 5 minutes short infusion will be characterized and the steady-state plasma concentrations (Css) estimated for individual subjects from the observed concentrations measured during the infusion. In addition, Css will be estimated by fitting a 2 or 3-compartment model to the plasma concentrations using a Bayesian algorithm as implemented in the software program WinNonlin. Relationships between Css, clinical outcomes (e.g., toxicity effect and anti-coagulation effects) will be explored.

Concentrations of Sevuparin/DF02 will be measured using a LMWH ELISA kit for plasma samples (Lifespan Technologies, Salt Lake City, Utah, USA) according to the manufacturer's instructions. The heparin - ELISA is a quantitative enzyme-linked assay designed for the in vitro measurement of LMWH levels in plasma. This assay measures heparin directly using a heparin binding protein which has been conjugated to horseradish peroxidase (HRP). Sevuparin/DF02 will be determined in human plasma using a validated solid-phase competitive ELISA method (0162-2009-P). Calibration Range: Sevuparin/DF02: 500 – 10000 ng/mL. The range of quantitation for this method was 1000-5000 ng/mL, the calibration curve fitted with the 4PL logistic model spanned a concentration range between 500 and 10000 ng/mL. Inclusion of a calibration standard outside the range of quantitation did not extend the validated range. The LLOQ for Sevuparin/DF02 was 1000 ng/mL and the ULOQ was 5000 ng/mL.

6 STUDY ACTIVITIES

Study visits should occur on the indicated visit day or within specified allowed window (see schedule of events below).

6.1 Screening Procedures for Study Entry part 1 and part 2

An Eligibility Screening Form (ESF) documenting the subject's fulfilment of the entry criteria for all subjects considered for the study is to be completed by the Investigator. Subjects who are considered for study entry, but who fail to meet the eligibility requirements, should also have an ESF completed with the reason for lack of eligibility. These subjects will not be entered into the

trial database. The ESFs for subjects who fail to meet the eligibility criteria requirements should be kept in the files at the sites.

Eligible subjects meeting the inclusion criteria for this study will be asked to read, comprehend and sign an informed consent form. The subject will sign the Informed Consent Form before she/he enters the study, i.e., before screening bloods, screening assessments or any other study-related activity.

The following procedures/evaluations must be completed **within 2-4 hours** prior to start of the study:

The sequence of events:

- Patient seek medical care
- Vital signs will be measured
- Routine screening for malaria and additional sampling as requested
- Informed consent
- Review of eligibility criteria (inclusion and exclusion criteria) ie additional, study specific lab tests
- Women of childbearing potential will undergo a urine pregnancy test.
- Laboratory tests will be performed see list under 5.7.1 for complete information of tests.
- The complete subject medical history and concomitant medications will be recorded
- A complete physical examination will be performed assessing the following: skin, eyes, ear, nose, throat, neck, thyroid, lungs, heart, abdomen, lymph nodes, extremities, and body weight and height
- 12-Lead ECG will be performed
- If the subject is found to be eligible, a subject number will be assigned and baseline data transcribed to CRF.

Patients in part 2 found eligible will be randomized with eqaual probabilities to treatment with Malanil® alone or the combination of Sevuparin/DF02 and Malanil®.

6.2 Treatment Period

Every patient will be treated with atovaquone-proguanil (Malanil®) as antimalarial drugs (four tablets once per day in three days).

The patients with early treatment failure (see below) will be treated with rescue treatment; A dose of artesunate 4 mg/kg/ day given once a day for 3 days and mefloquine 25 mg /kg/ dose split over 2 days.

6.2.1 Criteria of early treatment failure are defined below:

- Danger signs (according to Modified WHO criteria for severe malaria (see Appendix 2) or severe malaria in presence of parasitemia on any day
- An increase or a decrease less than 50 % of the concentration of parasites on the second day of treatment, compared with the concentration at admission

The initial antimalarial treatment in the study will be with oral atovaquone-proguanil (Malanil®) rather than an artemisinin combination therapy as the artemisinin component could potentially severely obscure the pharmacodynamic effects of Sevuparin/DF02. This proposed pharmacodynamic effect of Sevuparin/DF02 is reversal and prevention of cytoadherence of mature form parasitized red blood cells to vascular endothelium causing their sequestration into the microcirculation. Artemisinins have indirectly an important effect on cytoadherence, since they kill young ring stage parasites, preventing their further maturation and subsequent sequestration into the microcirculation. Because of this, the sensitivity to observe an effect of Sevuparin/DF02 on sequestration in patients with uncomplicated malaria would be importantly reduced by combining the drug with artemisinin treatment. In contrast atovaquone-proguanil (Malanil®) only acts on parasite in the second half of their extra-erythrocytic development, and therefore does not prevent sequestration.

Part 1. During the dose escalation stage:
Patients in each cohort will be given adjunctive therapy with Sevuparin/DF02 in accordance with the following dose escalation design: 1.5, 3 and 6 mg/kg/dose on days 1, 2, and 3 as illustrated below.

Sevuparin/DF02 will be administered every 6 hours for 3 consecutive days. The subjects will receive a total of 12 doses of Sevuparin/DF02 in combination with their anti-malarial treatment. During day 1, subjects will receive 4 short infusions of Sevuparin/DF02, on Day 2 they will receive 4 doses of Sevuparin/DF02 and on Day 3 they will receive 4 additional short infusions of Sevuparin/DF02.

The dose of Sevuparin/DF02 to be used in part 2: the extension stage of the study (randomized active controlled stage of the study) will be determined using the Fibonacci dose escalation scheme. The part 2 will start after completed safety report review, to the DSMB and the ethical committees concerned.

6.2.2 Part 1 all patients and part 2 patients randomzied to combination treatment

The patients will receive Sevuparin/DF02 administration as an adjunctive therapy toanti-malarial treatment, each 6 hours during 3 consecutive days, followed by follow-up visits at Day 7 and Day 14 from the first dose of Sevuparin/DF02 (Day 1).

Day 1:
The subject will undergo the following steps:

Prior to initial Sevuparin/DF02 dosing:
- A partial physical examination to update findings from the examination performed at screening
- Laboratory tests (haematology,chemistry,coagulation and urinalysis) see laboratory parameters at baseline investigations (above)if not performed at screening
- Women of childbearing potential will undergo a urine pregnancy test.
- Blood sample as pre-dose PK sample will be collected
- Blood sample for smear and parasite count will be taken

- A 12-lead pre-dose ECG as baseline (pre-dose) will be recorded, if not performed at screening
- Vital signs will be taken (pre-dose), if not performed at screening
- Concomitant medications recorded
- An updated medical history (when applicable)

Subjects will be administered antimalarial medication

Administration of first Sevuparin/DF02 dose: Sevuparin/DF02 will be administered via a short i.v. infusion over 5 minutes. The following procedures will be performed after initiation of dosing:

- Vital signs will be taken every 6 hrs post-infusion
- Any immediate AEs recorded post initial infusion
- A 12-lead pre-dose ECG and at 1 hour post initial infusion
- PK samples will be collected according to table **4.11.T2**
- Blood samples will be collected at various time points according to table **4.11.T2** to measure APTT, PT and antiXa see laboratory parameters at baseline investigations (above)
- Parasitaemia will be evaluated at 30, 60, 90 min, 2, 3 and 4 hours

Administration of anti-malarial medication and Sevuparin/DF02 doses 2, 3 and 4:

- If subject has well tolerated first initial dose of Sevuparin/DF02, the subject will be administered dose 2, 3 and 4 of Sevuparin/DF02, 6 and 12 and 18 hours after the first dose, respectively.
- Vital signs will be recorded every 6 hours.
- Chemistry, hematology at 24 hours post infusion will be performed see laboratory parameters at baseline investigations (above)Parasitemia will continue to be evaluated at 6, 8, 10, 12 hrs and then every 6 hrs until parasite negative on the peripheral blood
- AE evaluation
- An concomitant medications provided to the subject during this period will be recorded

Day 2

Prior to subject receiving Sevuparin/DF02 doses 5, 6, 7, and 8 the following will be assessed:

- A partial physical examination
- AE assessment and vital signs
- Laboratory tests (hematology, chemistry and coagulation) see laboratory parameters at baseline investigations (above)
  If the subject had tolerated the first day of therapy, and the investigator has determined that the patient can continue on study therapy, the patient will be administered:

- Subjects will be continue on study therapy (Sevuparin/DF02 iv doses 5, 6, 7 and 8 and their antimalarial regimen)
- Blood samples for APTT, PT and antiXa will be collected after dosing according to table **4.11.T2**. , see laboratory parameters at baseline investigations (above)
- Vital signs will be recorded every 6 hours (continued of schedule from Day 1)
- Parasitaemia will be measured every 6 hours
- Concomitant medications will be recorded

Day 3

Prior to subject receiving Sevuparin/DF02 doses 9, 10, 11 and 12, the following will be assessed:

- A partial physical examination
- AE assessment
- Vital signs (every 6 hours)
- Laboratory tests (haematology, chemistry and coagulation)see laboratory parameters at baseline investigations (above)
- Sevuparin/DF02 infusions (9, 10, 11 and 12) over 5 minutes plus antimalarial medication
- Concomitant medications will be recorded
- Parasitemia will be assessed every 6 hours
- Blood samples for PK, APTT, PT and antiXa will be collected after dosing according to table **4.11.T2**
- A 12-lead ECG will be recorded 1 hour after sevuparin dose 12

Day 4 -6

Prior to releasing the subject, the following procedures will be performed:

- A complete physical examination
- Vital signs and weight
- AE assessment
- Laboratory tests (haematology and biochemistry), if not back to baseline
- Coagulation , if not back to baseline
- Concomitant medications will be recorded
- Parasitemia will be assessed

Day 7 (First follow-up ± 1 day)

During this visit, the following procedures will be performed:

- A complete physical examination
- Updated medical history
- Vital signs and weight
- AE assessment
- Laboratory tests (haematology and biochemistry), see laboratory parameters at baseline investigations (above)
- Parasitemia
- Coagulation
- Concomitant medications will be recorded
- Evaluation of their disease status

Day 14 (Second follow-up ± 1 day)

During the final follow-up visit, the following procedures will be performed:

- A complete physical examination
- Updated medical history
- Vital signs and weight
- AE assessment

- Laboratory tests (haematology and biochemistry), see laboratory parameters at baseline investigations (above)
- Parasitemia
- Coagulation
- Concomitant medications will be recorded
- Evaluation of their disease status
- Women of childbearing potential will undergo a urine pregnancy test, to be followed on Day 30 with a phone call.

6.2.3 Part 2 patients randomsied to Malanil® alone

The patients will receive 3 consecutive days of anti-malarial treatment (Malanil®), followed by follow-up visits at Day 7 and Day 14 from the first dose of Malanil® (Day 1).

Day 1:
The subject will undergo the following steps:

Prior to initial Malanil®dosing:

- A partial physical examination to update findings from the examination performed at screening
- Weight measurement to be taken
- Laboratory tests (haematology and biochemistry and urinalysis) see laboratory parameters at baseline investigations (above)
- Women of childbearing potential will undergo a urine pregnancy test. Blood sample for smear and parasite count will be taken
- A 12-lead pre-dose ECG as baseline (pre-dose) will be recorded
- Vital signs will be taken (pre-dose)
- Concomitant medications will be recorded
- An updated medical history (when applicable)

Administration of first dose of Malanil®

The following procedures will be performed after initiation of dosing:

- Vital signs will be taken every 6 hours
- Any immediate AEs recorded
- Laboratory tests (haematology and biochemistry) see laboratory parameters at baseline investigations (above)
- A 12-lead pre-dose ECG 1 hour after first malanil administration.
- Adverse event (AE) evauation
- Parasitaemia will be evaluated at 30, 60, 90 min, 2, 3 and 6, 8, 10, 12 hrs and then every 6 hrs until parasite negative on the peripheral blood
- Concomitant medications will be recorded

Day 2
Prior to subject receiving Malanil® the following will be assessed:

- A partial physical examination

- AE assessment
- Laboratory tests (haematology, chemistry,) , see laboratory parameters at baseline investigations (above)
- Vital signs will be recorded every 6 hours (continued of schedule from Day 1)
- Parasitaemia will be measured every 6 hours
- Concomitant medications will be recorded

Day 3

Prior to subject receiving Malanil® the following will be assessed:

- A partial physical examination
- AE assessment
- Vital signs every 6 hours
- Laboratory tests (haematology and bio chemistry), see laboratory parameters at baseline investigations (above)
- Parasitemia will be assessed every 6 hours
- Concomitant medications will be recorded
- A 12-lead ECG will be recorded at timepoint 67:00 hours (from first dose of Malanil)

Day 4 -6

Prior to releasing the subject, the following procedures will be performed:

- A complete physical examination
- Vital signs
- AE assessment
- Laboratory tests (haematology and biochemistry), see laboratory parameters at baseline investigations (above), if not back to baseline
- Concomitant medications will be recorded
- Parasitemia will be assessed

Day 7 (First follow-up ± 1 day)

During this visit, the following procedures will be performed:

- A complete physical examination
- Updated medical history
- Vital signs
- AE assessment
- Laboratory tests (hematology and biochemistry) , see laboratory parameters at baseline investigations (above)
- Parasitemia
- Concomitant medications will be recorded
- Evaluation of their disease status

Day 14 (Second follow-up ± 1 day)

During the final follow-up visit, the following procedures will be performed:

- A complete physical examination
- Updated medical history

- Vital signs
- AE assessment
- Laboratory tests (hematology and biochemistry) , see laboratory parameters at baseline investigations (above)
- Parasitemia
- Concomitant medications will be recorded
- Women of childbearing potential will undergo a urine pregnancy test, to be followed on Day 30 with a phone call.
- Evaluation of their disease status

7 SELECTION AND WITHDRAWAL OF SUBJECTS

7.1 Pregnancy

Pregnancy, in and of itself, is not regarded as an adverse event, unless there is suspicion that study medication may have interfered with the effectiveness of a contraceptive medication or method. The procedures that will be followed based on whether a pregnancy is confirmed by a positive serum or urine test result are listed below. Pregnancy test will be performed at screeing, day 14 and an additional follow up call on day 30,

If pregnancy Confirmed by a Positive Urine Test Result:

- Investigator and subject must notify each other immediately
- Investigator must notify the sponsor immediately
- Study medication must be discontinued immediately
- A serum pregnancy test must be performed to confirm the urine test result. (The serum test should be performed at the investigative site to ensure the test will be performed promptly and the result available immediately for review.)

If a positive serum test confirms the urine test result, then:

- Withdraw the subject from the study
- Perform the required Early Termination visit study evaluations
- Investigator must complete and submit the required initial and follow-up report to the sponsor
- If a negative serum test does not confirm the urine test result, then:
- The Investigator will use his/her expert judgment, based on an assessment of the potential benefit/risk to the subject, to determine if it is in the subject’s best interest to resume study medication and continue participation in the study.

7.2 Subject Withdrawal Criteria

A subject may voluntarily discontinue study participation at any time. At any time, the Investigator may also at his/her discretion discontinue a subject’s study participation. Subjects may be withdrawn from this study for the following reasons:

- At the Investigator’s discretion in situations where the subject complies poorly with the protocol.
- Major protocol violation.
- If Sevuparin/DF02 treatment is delayed due to toxicity for more than 24 hours
- Intercurrent event preventing administration of study medication for more than 24 hours
- Subject withdraws consent.
- Subject becomes pregnant.
- Subject starts any other agent other than study therapy for other disease.
- Death

In the event of premature discontinuation, every effort should be made to perform the study follow-up procedures (physical examination including performance status, evaluation of toxicities).

Subjects who are withdrawn from this study due to toxicity must be followed with appropriate medical management until resolution or stabilization, regardless of evidence of disease status.

The subject will be advised in the Informed Consent Forms that he/she has the right to withdraw from the study at any time without prejudice, and may be withdrawn at the discretion of the Investigator/CRO/Dilaforette AB at any time. In the event that the subject drops out of the study or is withdrawn from the study, the withdrawal CRF should be completed. On the withdrawal page the Investigator should record the date of the withdrawal, the person who initiated withdrawal and the reason for withdrawal.

If the patient withdraws consent, the data and samples from the subject will not be excluded unless specifically requested.

Reasonable effort should be made to contact any subject lost to follow-up during the course of the study in order to complete assessments and retrieve any outstanding data.

8 ASSESSMENT OF SAFETY

8.1 Specification of Safety Parameters

8.1.1 Adverse Events

An Adverse Event (AE) is any untoward medical occurrence in a patient or clinical investigational subject administered a pharmaceutical product and which does not necessarily have a casual relationship with this treatment. An AE can be any unfavourable and unintended sign (including an abnormal laboratory finding), symptom, or disease temporally associated with the use of an Investigational product, whether or not related to the Investigational product. Pre-existing conditions which worsen during a study are to be reported as Adverse Events.

All adverse events, regardless of causal relationship, encountered during the clinical study will be reported on the AE page of the CRF (please see section 9.3). Intensity of AEs will be graded using the National Cancer Institute Common Terminology Criteria for Adverse Events (CTCAE,

version 4.0) and reported in detail as indicated on the CRF. If an adverse event occurs which is not contained in the NCI-CTCAE, the five-point scale (mild, moderate, severe, life threatening, death) below will be used.

Mild	discomfort noticed but no disruption of normal daily activity
Moderate	discomfort sufficient to reduce or affect daily activity
Severe	severe discomfort, inability to work or perform normal daily activity
Life threatening	represents an immediate threat to life
Death	death related to AE

8.1.1.1 Adverse Drug Reaction (ADR)

All untoward and unintended responses to a medicinal product related to any dose. The phrase "responses to a medicinal product" means that a causal relationship between a study medication and an AE is at least a reasonable possibility, i.e., the relationship cannot be ruled out. All cases judged by either the reporting medically qualified professional or the sponsor as having a reasonable suspected causal relationship to the study medication qualify as adverse reactions.

8.1.2 Laboratory Test Abnormalities

Laboratory test results will be recorded on the laboratory results pages of the CRF. Laboratory test value abnormalities as such should not be reported on the AE page of the CRF as adverse events, unless there is an associated clinical condition for which the subject is given treatment or concomitant treatment is altered, study treatment is interrupted/delayed or the dose of study drug is modified, or the subject is permanently discontinued from the study drug because of the abnormal test value.

8.1.3 Serious Adverse Event

A serious adverse event (SAE) is any untoward medical occurrence that at any dose:

- Results in death
- Is life-threatening (subject is at immediate risk of death from the event as it occurred),
- Requires subject hospitalization or prolongation of existing hospitalization
- Results in persistent or significant disability/incapacity
- Is a congenital anomaly/birth defect
- Other medically important condition

Hospitalisation longer than 3 days for the uncomplicated malaria, prolonged treatment may be needed and will not be regarded as a SAE.

All of the above criteria apply to the case as a whole and should not be confused with the outcomes of individual reactions/events. More than one of the above criteria can be applicable to the one event.

Important medical events that may not be immediately life-threatening or result in death or hospitalization may be considered an SAE when, based upon appropriate medical judgment, they may jeopardize the subject or require medical or surgical intervention to prevent one of the outcomes listed in the definition above. Examples of such medical events include allergic

bronchospasm requiring intensive treatment in an emergency room or at home, blood dyscrasias or convulsions that do not result in hospitalization, or development of drug dependency or drug abuse.

The definition and reporting requirements of the ICH Guideline for Clinical Safety Data management,

All SAEs, whether or not deemed drug-related or expected, must be reported to the pharmacovigelance team of the CRO, Dilaforette AB and to the trial clinical monitor.

8.1.4 Follow-up of Adverse Events

All adverse events (and treatments for them) must be documented throughout the Study Treatment Phase starting after the administration of the first dose of study medication and for 30 days after the last intake of study medication, and followed up until the event is either resolved or adequately explained, even after the subject has completed his/her study treatment. Unrelated, mild or moderate events must be followed for 30 days after the last study drug administration. Serious, life threatening or related events must be followed until resolution.

8.1.5 Follow-up of Abnormal Laboratory Values

In the events of unexplained abnormal laboratory test values, the tests should be repeated and followed up until they have returned to normal range or to the baseline value, and/or an adequate explanation of the abnormality is found. If a clear explanation is established it should be recorded on the CRF.

8.1.6 Pregnancy

Pregnancy, in and of itself, is not regarded as an adverse event, unless there is suspicion that study medication may have interfered with the effectiveness of a contraceptive medication or method. The procedures that will be followed based on whether a pregnancy is confirmed by a positive serum or urine test result are listed below:

- Pregnancy Confirmed by a Positive Serum Test Result
- Investigator and subject must notify each other immediately
- Investigator must notify the sponsor immediately
- Discontinue study medication immediately
- Withdraw the subject from the study
- Perform the required Early Termination visit study evaluations
- Investigator must complete and submit the initial and follow-up report to the sponsor

If a negative serum test does not confirm the urine test result, then:

- The Investigator will use his/her expert judgment, based on an assessment of the potential benefit/risk to the subject, to determine if it is in the subject's best interest to resume study medication and continue participation in the study.

Any pregnancy diagnosed during the study, or that occurs within 30 days after stopping study medication, must be reported immediately to the Investigator. The Investigator will notify the

sponsor or designee. The outcome of all such pregnancies (i.e., spontaneous miscarriage, elective termination, normal birth, or congenital abnormality) must be documented and followed-up on a form that will be provided by the sponsor. The pregnancy will be followed to term and the outcome, including any premature termination, must be reported to the sponsor. All live births must be followed for a minimum of 30 days or to the first well-baby visit. All reports of congenital abnormalities/birth defects and spontaneous abortions/miscarriages should be reported as an SAE for this study. Elective abortion procedures, without complications, should not be considered as adverse events.

8.2 Assessing, Recording, and Analyzing Safety Parameters

Safety lab assessments will be performed by the local investigative site and will be collected and processed according to standard investigative site procedures. Normal value ranges and laboratory certification will be collected from all laboratories prior to study initiation.

Safety labs will include:

- Haematology/Complete Blood Count with differential, including cell counts, haemoglobin, hematocrit, RBC, platelet count.
- Coagulation: APTT, PT and anti-Xa (only applicable for patients receiving Sevuparin/DF02)
- Clinical Chemistry panel including, total bilirubin, alkaline phosphatase, AST, ALT, LDH, calcium, BUN, creatinine,CPK, electrolytes

8.2.1 ECG Parameters

ECG parameters will be analyzed including ECG abnormalities and Investigator’s assessment of clinical significance analyzing above parameters, rhythm abnormalities, QTc interval and conduction abnormalities. Any abnormalities will be reported on the CRF with a copy of the ECG recording.

8.3 Recording and Reporting Adverse Events/Intercurrent Illnesses

The Investigator will probe, via discussion with the subject, for the occurrence of AEs during each subject visit and record the information in the site’s source documents. For each AE, the Investigator should note the start and resolution dates, the severity, whether it meets the definition of an SAE, the relationship of the event to the study drug, the action taken regarding study drug, and the outcome of the event. Data should be transcribed from the source documents to the case report forms as per the case report form instructions, so that all changes in intensity and seriousness will be captured on the AE case report form.

The Investigator must report in detail all adverse signs and symptoms which are either volunteered by subjects or observed during or following the course of Investigational product administration on the appropriate CRF page.

All AEs will be monitored until resolution or, if the AE is determined to be chronic, a cause is identified. If an AE is considered possibly related to study treatment and remains unresolved at the conclusion of the study, this event will be followed until resolution, stabilization, or initiation of treatment that confounds the ability to assess the event.

8.3.1 Relationship of AE to Study Medication

The relationship of an adverse event to study medication is to be assessed according to the following definitions

**Definitely Related:**	Strong evidence exists that the study drug caused the adverse event. There is a temporal relationship between the event onset and administration of the study drug. There is strong therapeutic and pharmacologic evidence that the event was caused by the study drug. The subject's clinical state or concomitant therapies have been ruled out as a cause. In the case of cessation or reduction of the dose, the event abates or resolves, and reappears upon re-challenge.
**Probably Related:**	A temporal relationship exists between the event onset and the administration of study drug, and appears with some degree of certainty to be related based on the known therapeutic and pharmacologic actions of the study drug. It cannot be readily explained by the subject's clinical state or concomitant therapies. In the case of cessation or reduction of the dose, the event abates or resolves.
**Possibly Related:**	A temporal relationship exists between the event onset and the administration of study drug. Although the adverse event may appear unlikely to be related to the study drug, it cannot be ruled out with certainty; and/or the event cannot be readily explained by the subject's clinical state or concomitant therapies.
**Not Related:**	Evidence exists that the adverse event definitely has an aetiology other than the study drug (e.g., pre-existing condition or underlying disease, intercurrent illness, or concomitant medication) and does not meet any criteria listed above.

Included in the description should be the nature of the sign or symptom; the date of onset; date of resolution (duration); the severity; the relationship to study treatment or other therapy; the action taken (if any), and the outcome.

8.3.2 Unexpected Adverse Event

An unexpected adverse event is one not previously reported (in nature, severity or incidence) in the current Investigator's Brochure, in the clinical plan, or elsewhere. Unexpected, as used in this definition, refers to an adverse drug experience that has not been previously observed (e.g.,

included in the Investigator Brochure) rather than from the perspective of such experience not being anticipated from the pharmacological properties of the pharmaceutical product.

8.3.3 Reporting of Serious Adverse Events

Adverse events classified as serious require expeditious handling and reporting to the pharmacovigelance team of the CRO to comply with regulatory requirements. For any serious adverse event (SAE) that occurs while a subject is on-study; within 30 days of the last study drug administration, regardless of any opinion as to the relationship of the SAE to the study drug; or if any SAE that the Investigator feels is related to the study drug occurs later than 30 days after the last study drug administration, the CRO must be notified by telephone within 24 hours of becoming aware of the event. During both business and non-business hours, the telephone number listed below should be used to notify the CRO. A recorded message will provide the caller with the pager number of the on-call monitor.

All SAEs require that, in addition to telephone notification, a Serious Adverse Event Report Form be completed and forwarded via facsimile to the pharmacovigelance team of the CRO at the number listed below within 24 hours of becoming aware of the event.

All SAEs (not clearly related to disease progression) whether considered to be drug-related or not should be considered unexpected in this study, and must rapidly be communicated to the institutional review board(s) and all other investigators.

SAEs will be reported to:

Phone: +33 1 44 90 32 90
Fax : +33 1 44 90 32 75
E-mail: **DF02_safety@parexel.com**

All adverse events must be recorded in the case report form (CRF). To avoid colloquial expressions, the adverse event should be reported in standard medical terminology and will be coded in MedDRA. Whenever possible, the adverse event should be evaluated and reported as a diagnosis rather than as individual signs or symptoms. If a definitive diagnosis is not possible, the individual symptoms and signs should be recorded. Any laboratory abnormalities deemed clinically significant by the Investigator should be reported on the adverse event CRF. Whenever possible, the aetiology of the abnormal findings will be documented on the CRF. Any additional relevant laboratory results obtained by the Investigator during the course of this study will be supplied to the sponsor and recorded on the CRF.

The required SAE information should also be completed on the CRFs and the data reported within 24 hours from the time that the event was identified. This will ensure that the written documentation is transmitted to the appropriate pharmacovigilance contact person within the required reporting time period.

The IRB/regulatory authority must be informed if the SAE or AE, in the opinion of the CRO, Dilaforette AB or the Investigator, is likely to affect the safety of the subjects or the conduct of the study.

All Investigators participating in the trial will also be notified of any unexpected SAEs determined to be related to study treatment.

For any questions about adverse events or serious adverse events contact:

Sponsor Medical Monitor:
Per Arne Parment M.D, PhD
Dilaforette AB
Karolinska Institutet Science Park
Nobels väg 3
SE-171 65 Solna
Sweden
Tel: +46-70-6444771
Fax: +46-8-32 31 44

8.4 Warnings and Precautions

Any medication, other than the study medication taken during the study will be recorded in the CRF.

Subjects receiving study therapy (study therapy is define as Sevuparin/DF02 or in combination with anti-malarial medication (atovaquone-proguanil (Malanil®) or Malanil® alone should be monitored by a physician experienced in the use of anti-malarial agents.

8.4.1 Sevuparin/DF02 Precautions

Infusion reactions with the first dose of Sevuparin/DF02 may be observed. Sevuparin/DF02 administration should be interrupted in all subjects with severe infusion reactions and appropriate medical therapy administered.

Statural dizziness, elevated liver enzymes, somnolence, and pharyngitis were the most frequently reported adverse reactions in the first study in man with Sevuparin/DF02.

Due to the anticoagulant properties of Sevuparin in high doses bleeding may occur.

8.4.2 Malanil@ Precautions

Malanil@ has not been evaluated for the treatment of cerebral malaria or other severe manifestation of complicated malaria including hyperparasitaemia, pulmonary oedema or renal failure.

The concomitant administration of Malanil@ and rifampicin or rifabutin is not recommended.

The patients with severe renal impairment (creatinine clearance less than 30 mL/min) alternatives to Malanil@ should be recommended for treatment of acute P. falciparum malaria whenever possible.

The following adverse events have been reported;

Cardiovascular: anginal symptoms, conduction disturbances, ventricular tachycardia, severe hypotension, arrhythmia and acute circulatory failure.

CNS: Vertigo, headache, fever, apprehension, restlessness, confusion, syncope, excitement, delirium, hypothermia, convulsions and dizziness.

GI: Nausea, vomiting, epigastric pain, hepatitis and GI disturbance.

Hematologic: Acute hemolysis, haemolytic anaemia, thrombocytopenic purpura, agranulocytosis and hypoprothrombinaemia.

Hypersensitivity: Cutaneous rashes (urticarial, popular and scarlatinal), pruritus, flushing, sweating, facial oedema and asthmatic symptoms.

Ophthalmic: Visual disturbances including disturbed colour vision and perception, photophobia, blurred vision with scotomata, night blindness, amblyopia, diplopia, diminished visual fields, mydriasis and optic atrophy.

Others: Vasculitis, hypoglycemia, lichenoid photosensitivity, granulomatous hepatitis, hepatocellular cholestatic hepatotoxicity, renal failure associated with coagulopathy.

8.4.3 Interaction with Other Medicine Products

Proguanil may potential the anticoagulant effect of warfarin and other coumarin based anticoagulants. The mechanism of this potential drug interaction has not been established. Caution is advised when initiating or withdrawing malaria prophylaxis a treatment with atovaquone-proguanil in patients on continuous treatment with coumarin base anticoagulants.

Concomitant treatments with tetracycline, metoclopramide, rifampicin and rifabutin have been associated with significant decreases in plasma concentrations of atovaquone.

Frequently reported adverse events: The most frequently reported adverse events are nausea, vomiting, loose stools or diarrhoea, abdominal pain, dizziness or vertigo, and neuropsychiatric events such as headaches. These are usually mild and may decrease despite continued use.

Infrequent adverse events include:

Cardiovascular: hypotension, hypertension, flushing and syncope. Chest pain, tachycardia or palpitation, bradycardia, irregular pulse, extrasystoles, A-V block and other transient cardiac conduction alterations.

Skin disorders: Rash, exanthema, urticaria, pruritus, oedema, hair loss, erythaema multiforme and Stevens-Johnson syndrome.

Musculoskeletal disorders: muscle weakness, muscle cramps, myalgia and arthralgia.

Other symptoms: visual disturbances, vestibular disorders including tinnitus and hearing impairment, dyspnea, asthenia, malaise, fatigue, fever, sweating, chills, dyspepsia and loss of appetite.

Laboratory abnormalities: Most frequently decreased hematocrit, transient elevation of transaminases, leucopoenia and thrombocytopenia. These alterations were observed in subjects with acute malaria who received treatment doses of the drug and were attributed to the disease itself.

8.5 Eficacy Assessments

8.5.1 Pharmacokinetics (part 1 all patients and part 2, 10 patients)

Plasma samples for establishing the pharmacokinetic and biomarker profiles will be obtained at times specified in the protocol. The results of the exploratory assessments will not be recorded in the CRF, but will be reported separately in an addendum.

8.5.2 Pharmacodynamics (part 2)

Parasite clearance curves and sequential peripheral blood parasite staging of Sevuparin/DF02 treated patients will be compared with the control group. If cytoadherence and thus sequestration of IRBCs containing the more mature forms of the parasite is indeed affected by Sevuparin/DF02, a temporary rise in parasitemia and appearance of more mature stages in the peripheral blood can be expected. The clearance curves in relation to the peripheral blood staging will be modeled using stage distribution, proportion of stage specific sequestration and stage specific parasite clearance as parameters. A similar approach has been trialed in the evaluation of levamisole as anti-adhesive adjunctive therapy in falciparum malaria (Dondorp et al. J Infect Dis 2007; 196:460–6). Differences in sequestration between Sevuparin/DF02 treated patients and the control group will be evaluated by comparing the integrated numbers (in parasites per microliter) and parasitemia (in percentages) of trophozoite- and schizont-stage parasites seen in the peripheral blood over time up to 72 h, determined as the area under the time-parasitemia curve. This will be the primary end point. Moreover, secondary end points include the differences in sequestration assessed through calculation of stage-specific sequestration ratios (SQRs) at various sample times, defined as follows: SQR=observed number of parasites / (expected number of parasites xC_f) , where the observed number of parasites is the peripheral blood parasitemia (in parasites per microliter) of a well-defined developmental stage; the expected number of parasites of that developmental stage is the number expected to be present in the peripheral blood if the matching cohort of circulating younger-form parasites on admission had developed unrestricted, without either sequestration in the microvasculature or splenic clearance; and C_f is the factor by which parasites are cleared by the spleen and through antimalarial drug action. Matching developmental-stage cohorts at different times after admission were derived from the parasite developmental ages bordering the morphological stages [Desakorn V, et al. Trans R Soc Trop Med Hyg 2005; 99:517–24.8]. The well-defined morphological stages of the parasite consist of the following: tiny rings, small rings, large rings, early trophozoites, midtrophozoites, late trophozoites, and schizonts [Silamut K, et al. Am J Pathol 1999; 155:395–410]. The parasite asexual-stage ages (from merozoite invasion) bordering the morphological stages, as assessed by in vitro culture, are, respectively, 12, 17, 22, 28, 37, and 42 h. Assuming similar development rates in vivo, a cohort of large-ring forms on admission will evolve to the early trophozoite stage 6 h later (figure 8.5.2.F1). Other matching cohorts include tiny rings on admission and small and large rings combined after 12 h, small rings on admission and large rings after 6 h, early trophozoites after 12 h and midtrophozoites after 18 h, and large rings on admission and either midtrophozoites after 12 h or late trophozoites after 18 h. A decrease in SQR, thus, represents an increase in sequestration, if Cf is constant. Parasite clearance time is defined as the interval between the start of treatment and the time of the first of 2 sequential negative thick films. Parasite reduction ratios (PRRs) at 24 and 48 h are defined as the ratio of the parasite count at admission to that at 24 and 48 h, respectively [White NJ. Antimicrob Agents

Chemother 1997; 41:1413–22]. Assessment of peripheral blood slides will be performed by 2 independent microscopists, who are blinded to the study drug allocation.

Figure 8.5.2.F1. Morphological Stages of the *P. Falciparum*

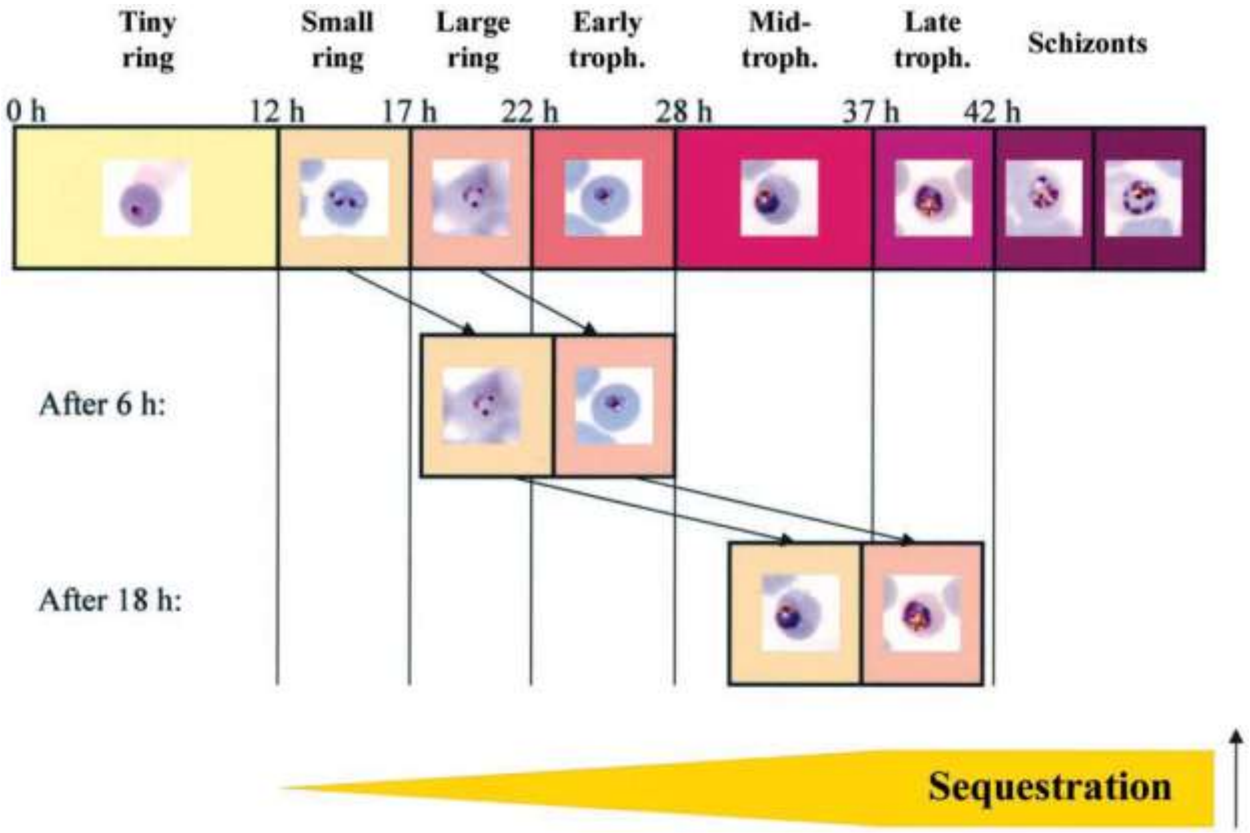

9 STATISTICAL CONSIDERATIONS

9.1 General Considerations

All statistical analysis including all tables, figures and data listings will be performed by SPSS software (version 15.0) and STATA (version 10) (validated statistical program). Data will be log transformed to obtain a normal distribution, where necessary. Normally distributed data will be compared using Student's t test. The Mann-Whitney U test will be used for non-paired nonparametric data. Categorical data will be compared by Pearson's chi-squared test or Fisher's exact test, as appropriate. The level of significance will be $p<0.05$. Summary statistics for continuous variables will include the mean, standard deviation, median, minimum, and maximum value; categorical variables will be presented as counts and percentage.

9.1.1 Sample Size and Associated Power

Part 1

The primary objective of the MTD study is to evaluate the safety and tolerability of administered intravenously to subjects with uncomplicated malaria. This will entail the determination of the maximum tolerated dose (MTD) of Sevuparin/DF02. The MTD is defined as the dose level below the dose level that results in unacceptable toxicity. The sample size is based on previous experiences of similar studies rather than on statistical calculations. This trial is not powered to demonstrate statistical significance.

Cohorts of 3 to 6 subjects will be accrued sequentially to each of the planned dosage levels for the MTC study. If 1 of 3 subjects experience a dose limiting toxicity as defined in the protocol then an additional 3 subjects will be accrued to that dose level. If 2 or more subjects in a cohort experience dose-limiting toxicity, then the MTD has been surpassed and a total of 6 subjects must be treated at the previous level to ensure its tolerability. Dose escalation will proceed according to the scheme as outlined.

Part 2

For the extension study, a sample size of 40 in the Sevuparin/DF02 treated group and 40 in the placebo group allows detection of a difference of 0.5 logs on the AUC of the late-stage peripheral blood parasitemia over time curve from log 10^5 parasites/µL/h to log $3X10^5$ parasites/µL/h between the treated and the control group, with an expected SD of 0.8 logs in both groups, significance level of 0.05 and a power of 80%.

9.1.2 Randomization of the Trial

There will be no randomization of subjects in the Part 1, MTD study. For part 2, the extension study, subjects will be randomized 1:1 in blocks of ten. The randomization codes will be distributed to each site, as needed, in blocks of 10 until all 80 patients are recruited.

9.2 Populations

9.2.1 Safety Population

The safety population will consist of all subjects who receive at least one dose of Sevuparin/DF02.

9.2.2 Pharmacokinetic Population

Subjects that received at least one infusion of Sevuparin/DF02 will be included in the pharmacokinetic study. All the patients in Part 1 (MTD) and in Part 2 the first 10 patients randomized to the Sevuparin/DF02 arm.

9.3 Analysis of Safety

All subjects in the study will be evaluated for safety at the completion of schedule events.

Vital signs (systolic and diastolic blood pressure, and pulse) and physical examination will be summarized by treatment group using appropriate descriptive statistics. Continuous variables will be summarized using a number of observations, mean, standard deviation, minimum, median and maximum values. Categorical values will be summarized using number of observations and percentages.

Withdrawals from the study will be summarized by dose group.

9.3.1 Incidence of Adverse Events

Adverse events will be classified and graded according to the NCI Common Terminology Criteria for Adverse Events (CTCAE), version 4.0.

The primary safety analysis using descriptive statistics will include summaries of incidence rates, severity, and type of adverse events; summary of changes in subjects' clinical laboratory results; and summaries of the number of toxicity grades for laboratory and non-laboratory data.

Adverse events will be coded using the MedDRA adverse event dictionary. Frequency of treatment emergent adverse events will be calculated for each body system, by preferred term, by treatment group, for number of subjects and proportion reporting the event. The severity of the adverse events and the relationship to study medication will be summarized for each body system and preferred term by treatment group. Withdrawals due to adverse events will be summarized for each body system and preferred term by treatment group.

9.3.2 Laboratory Data

The number and percentage of subjects with laboratory abnormalities at the screening visit and the final visit (or early withdrawal) will be tabulated.

Descriptive statistics (number of observations, mean, standard deviation, minimum, median and maximum values) will be calculated for clinical laboratory tests (hematology, coagulation, clinical chemistry, and urinalysis) at applicable visits.

9.3.2.1 Analysis of paracites

1) Pheripheral blood parasetemia will be assessed in thin films frequently. This will be done both to assess numbers of parasites and to assess the stages of the parasites in the blood at different timepoints, until clearance.
2) Infected red blood sell cytoadherence will be evaluated. See further Appendix 7.
3) Additionally, malaria infected red blood cells adhere to other blood cells, this will be evauated in a resetting assay see Appendix 8.
4) Differencies in sequestration between Sevuparin/DF02 and the control group will be evaluated by comparing the integrated numbers (in parasites per microliter) and parasetemia (in percentages) of trophozite- and schizont-stage parasites seen in the peripheral blood over time, determined as the area under the time- parasetemia curve. Se pharmacodynamics

9.4 Analysis of Pharmacokinetics

9.4.1 Plasma Pharmacokinetic Parameters of Sevuparin/DF02

The following parameters will be measured for the subjects included in the pharmacokinetic analysis.

- Maximum concentration (maximum C)
- Minimum concentration (minimum C)
- Area under the concentration-time curve from time zero to the last measurable concentration [AUC_{0-t}]
- Area under the concentration-time curve from time zero to infinity [AUC_{0-inf}]
- Half life (t½)
- Terminal elimination constant (kel)
- Clearance (CL)
- Time of maximum concentration (T_{max})
- Volume of distribution (V_d)

The mean primary pharmacokinetic estimates (Cmax, Cmin, AUC0-t, AUC0-inf, t1/2, kel, CL, Tmax, and Vd) for each treatment group, of Sevuparin/DF02 obtained from the model dependent analysis and from the population kinetic analysis will be compared.

The pharmacokinetic analysis will generate individual plasma concentration versus time curves. The aim is to describe the relationship between Sevuparin/DF02 concentration and clinically relevant adverse effects considering their time-course. Because this is an exploratory analysis, the methods cannot be specified in advance.

A population kinetic analysis will be performed with the primary aim to evaluate the population distribution of pharmacokinetic parameters in patients from the MTD study and additionally 10 patients from the extension study. Estimates of the individual kinetic parameters for all subjects will be obtained through Bayesian feedback using the results of the population kinetic analysis and the individual data for dosing and plasma concentrations of Sevuparin/DF02.

Model-dependent individual kinetic modelling will be performed in each subject from whom more than four blood samples have been obtained. It is foreseen that a one compartment open model describes the data best according to previous results with Sevuparin/DF02. However, other models will also be tested. Different weighting schemes for the plasma concentration data will be tested and the scheme that gives the lowest coefficient of variation of the estimates will be applied. The WinNonlin software system will be used for the individual kinetic modeling.

9.5 Subject Accountability and Missing Data

Data from subjects lost to follow-up will be tabulated and any difference between those subjects and subjects not lost to follow-up will be noted.

The data collected prior to a subject withdrawal will, unless specifically requested, be included.

9.6 Interim Analysis

There will be an interim analysis between part 1 and part 2 of the study, for safety evaluation. An interim safety report will be submitted to the DSMB and ethical committees concerned. Additionally, safety data will be evaluated prior to each dose escalation.

10 DIRECT ACCESS TO SOURCE DATA / DOCUMENTS AND INVESTIGATOR RESPONSIBILITY

It is understood that the term "investigator" as used in this protocol and on case report forms refers to the principal investigator or a member of the staff that the principal investigator designates to perform a certain duty. However, on specific CRFs the investigator him/herself is required to sign where indicated. The principal investigator is ultimately responsible for the conduct of all aspects of the study.

The investigator will ensure that this study is conducted in full conformance with the principles of the "Declaration of Helsinki" (as amended in Tokyo, Venice, Hong Kong, Somerset West, Edinburgh and Seoul) or with the laws and regulations of the country in which the research is conducted, whichever affords the greater protection to the individual.

It is the responsibility of the investigator to obtain written informed consent from each individual participating in this study, after adequate explanation of the aims, methods, objectives and potential hazards of the study. The investigator must also explain to the subjects that they are completely free to refuse to enter the study or to withdraw from it at any time. The document must contain all the elements described in 21 CFR, Part 50.

The investigator shall make accurate and adequate progress reports to Dilaforette AB and/or its representative and to the IRB on the progress of the study at appropriate intervals. Additionally, the investigator shall make accurate and adequate final status reports, through completed CRFs, to the monitors and to the IRB within three weeks after the completion, termination, or discontinuation of the study. The final status report must include all case report forms that were not previously provided to the monitors.

Qualified representatives of the sponsor or sponsor designees ("study monitors") will monitor the study according to a predetermined monitoring plan. Monitoring visits provide the sponsor with the opportunity to:

- Evaluate the progress of the study
- Verify the accuracy and completeness of CRFs
- Assure that all protocol requirements, applicable laws and/or regulations, and investigator's obligations are being fulfilled
- Resolve any inconsistencies in the study records.

The investigator must allow the study monitors to periodically review, at mutually convenient times during the study and after the study has been completed, all CRFs and office, hospital, and laboratory records supporting the participation of each subject in the study. The CRFs and other documentation supporting the study must be kept up-to-date by the investigator and the research

staff at the investigative site. These study materials must be available for review by the study monitor, and/or other qualified representatives of the sponsor, at each monitoring visit.

The study monitor will regularly inspect the various records of the study (CRFs, subject medical and laboratory records, and other pertinent data) provided that patient confidentiality is maintained in accordance with local institution, state, country, and federal requirements. The study monitor will verify the data against other source documentation in order to verify its accuracy and completeness. The study monitor will identify data discrepancies and collaborate with the investigator and research staff to resolve the discrepancies in the CRF as in a timely manner. The study monitor will generate queries as required using the CRF system. Queries will be tracked via a central reporting tool until satisfactory resolution is achieved. Protocol deviations will also be identified and recorded on a “Protocol Deviation Log”. The study monitor will follow an “Issue Escalation” plan in order to ensure that each issue identified during a monitoring visit is appropriately documented, reported, and resolved in a timely manner in accordance with the plan’s requirements.

10.1 Source Data

Source data is defined as all information in original records and certified copies of original records of clinical findings, observations or other activities in a clinical trial necessary for the reconstruction and evaluation of the trial. Source data are contained in source documents (original records or certified copies).

10.2 Source Documents

Source documents are defined as original documents, data and records (e.g. hospital records, clinical and office charts, laboratory notes, memoranda, subject diaries or evaluation check lists, pharmacy dispensing records, recorded data from automated instruments, copies or manuscripts certified after verification as being accurate copies, microfiches, photographic negatives, microfilm or magnetic media, x-rays, subject files, records kept at pharmacy, at the laboratories and at medico technical departments involved in clinical trial).

10.3 Direct Access

Direct access is defined as the permission to examine, analyze, verify and reproduce any records and reports that are important to evaluation of a clinical trial. Any party (e.g. domestic and foreign regulatory authorities, Dilaforette AB / CRO monitors and auditors) with direct access should take all reasonable precautions within the constraints of the applicable regulatory requirements to maintain the confidentiality of subject identities and sponsor proprietary information.

10.4 Study Monitoring

In accordance with applicable regulations, Good Clinical Practice, and Dilaforette AB or its representative’s procedures, monitors will periodically contact the site, including conducting on-site visits. During these contacts, the monitor will check and assess the progress of the study,

review the data collected, conduct source document verification, and identify any issues and address their resolution. These activities are performed in order to verify that the data are authentic, accurate, and complete; the safety and rights of the subjects are being protected; and the study is conducted in accordance with the currently approved protocol (and any amendments), GCP, and all applicable regulatory requirements.

11 QUALITY CONTROL/ QUALITY ASSURANCE

An independent audit at the study site may take place at any time during or after the trial. The independent audit can be carried by the QA Department at the CRO, the QA department of Dilaforette ABs or a regulatory authority.

The study will be conducted in accordance with the current approved protocol, ICH GCP, relevant regulations and the Thailand/Laos Major Overseas Programme standard operating procedures.

11.1 Quality Control

Quality Control is defined as the operational techniques and activities undertaken within the quality assurance system to verify that the requirements for quality of the trial related activities have been fulfilled.

Regular monitoring will be performed according to ICH GCP. Data will be evaluated for compliance with the protocol and accuracy in relation to source documents. Following written standard operating procedures, the monitors will verify that the clinical trial is conducted and data are generated, documented and reported in compliance with the protocol, GCP and the applicable regulatory requirements.

11.2 Quality Assurance

Quality Assurance is defined as the planned and systematic actions that are established to ensure that the trial is performed and the data are generated, documented (recorded) and reported in compliance with Good Clinical Practice (GCP) and the applicable regulatory requirements.

11.2.1 Inspection

An Inspection is defined as the act by a regulatory authority of conducting an official review of documents, facilities, records and any other resources that are deemed by the authorities to be related to the clinical trial and that may be located at the site of the trial, or at the sponsors and or clinical research organization facilities or at any other establishments deemed appropriate by the regulatory authorities.

11.2.2 Audit

An Audit is a systematic and independent review of trial related activities and documents to determine whether the validate trial related activities were conducted and the data were recorded,

analyzed and accurately reported according to the protocol, designated Standard Operating Procedure (SOPs), Good Clinical Practice (GCP) and the applicable regulatory requirements.

12 ETHICS APPROVALS

Before initiating a trial, the Investigator should have written and dated approval/favourable opinion from the relevant IRB/IEC for the trial protocol (and any amendments), written informed consent form, consent form updates, subject recruitment procedures (e.g. advertisements), and any other written information to be provided to subjects. Approval will be indicated in writing with reference to the final protocol number and date. Details of the IRB/IEC's constitution including names of its members and what function they perform on the committee (e.g. chairman, specialist, and lay-member) should be made available.

During the trial the Investigator should provide to the IRB/IEC all documents that are subject to review.

The Investigator will ensure that this study is conducted in compliance with the current revision of the Declaration of Helsinki. The study will be conducted in accordance with the principles of ICH GCP.

The study and its associated documents will be submitted to the appropriate ECs; the Oxford Tropical Research Ethics Committee (OXTREC), the Mahidol University Faculty of Tropical Medicine Ethics Committee (FTMEC), and the Thailand Ministry of Public Health Ethics Committee (MOPHEC) for written approval. The Investigator will submit and, where necessary, obtain approval from the above parties for all substantial amendments to the original approved documents.

12.1 Independent Ethics Committee (IEC)

This is an independent body a review board or a committee, institutional, regional, national or internationally constituted of medical / scientific professional and non-medical/ non scientific members whose responsibility it is to ensure that the protection of the rights, safety and well being of human subjects involved in the trial to provide public assurance of that protection, by reviewing and providing a favourable opinion on the trial protocol, suitability of the investigator, facilities and the methods and material to be used in obtaining and documenting informed consent from trial subjects

The legal status, composition, function, operations and regulatory requirements pertaining to Independent Ethics Committee may differ among countries, but should allow the Ethics Committee to act in agreement with GCP.

12.2 Institutional Review (IRB)

This is an independent body constituted of medical scientific and non-scientific members, whose responsibilities is to ensure the protection of the rights, safety and well being of human subjects involved in a trial by among other things reviewing approving and providing continued review of trial protocol and amendments and of the methods and material used in obtaining and documenting informed consent of the trial subjects.

12.3 Drug Safety Committee (Data Safety Monitoring Board –DSMB)

To ensure the safety of the patients all available demographic and safety data (including, but not limited to, cumulative listings of AE and SAE reports, laboratory data including coagulation and bleeding time, ECGs, vital signs, and physical examinations) will be reviewed on an ongoing basis by a Safety Monitor/ Chairman of DSMB. The safety monitor will inform the DSMB in case of any findings. Based on these ongoing reviews, the DSMB may make recommendations and Dilaforette will from this decide on the enrolment, modify dose level, add or modify safety procedures, or discontinue the study and moving forward with the dose escalation steps.

The committee will be responsible for monitoring for each dose escalation during the Part 1 and on an approximately semi-monthly basis, or at necessary time-points, the safety data of the clinical trial and the safety of the patients. This review will continue until the last patient has been recruited. This group will consist of independent experts not involved in the trial, including at least 2 practicing infectious disease MDs and expert in clinical research ethics, one statistician, and one safety person. Furthermore, representatives from Dilaforette e.g., medical expert, one drug expert and pre-clinical manager will also participate in the meetings as non-voting members.

The drug safety committee will provide recommendations concerning the progress of the trial and the safety of the patients after each meeting.

The drug safety committee will be provided with summary tables on demographic data, adverse events, serious adverse events, laboratory abnormalities, shift in laboratory values, withdrawals from study medication and death treatment group. Further information may be given on request; however, the efficacy information may be provided to the drug safety committee.

13 DATA HANDLING AND RECORD KEEPING

13.1 Completion of Case Report Forms

All study data will be recorded on standard paper Case Report Forms. Data will be entered on a secure database in accordance with standard operating procedures. Data may be used alone or in combination with data from related studies in secondary analyzes.

The patients will be identified by a study specific patients number and/or code in any database. The name and any other identifying details will NOT be included in any study data electronic file.

Data reported on the CRF that are derived from source documents should be consistent with the source documents or the discrepancies should be explained. Any data to be recorded directly on the CRFs (to be considered as source data) will be identified at the start of the trial. Data may be used alone or in combination with data from related studies in secondary analyses.

13.2 Archiving

The investigator sites should retain essential documents until at least 2 years after the last approval of all outstanding marketing application(s) in an ICH region or at least 2 years have elapsed since the formal discontinuation of clinical development of the Investigational product. These documents should be retained for a longer period however, if required by the applicable regulatory requirements or by an agreement with Dilaforette AB. It is the responsibility of Dilaforette AB to inform the Investigator as to when these documents no longer need to be retained.

14 FINANCING AND INSURANCE

The costs necessary to perform the study will be agreed with each Investigator and will be documented in a separate financial agreement that will be signed by the Investigator and Dilaforette AB prior to the trial commencing.

Subjects will be reimbursed for reasonable study-related travel expenses and loss of income.

Dilaforette AB has appropriate insurance cover and will provide compensation for injury caused by taking part in this study. Insurance policies aim at remaining in full force and effect for least 12months beyond the termination of the Clinical Trial.

15 PUBLICATION POLICY

15.1 Publication Policy

It is intended that the results of the study will be published as scientific literature. Results may also be used in submissions to regulatory authorities. The following conditions are to protect commercial confidential materials (patents, etc), not to restrict publication. However, any proposed oral or written use of such results must be submitted to Dilaforette AB, for review and written approval at least 60 days prior submission for publication, presentation, or use.

All information concerning Sevuparin/DF02 (such as patent applications, formulae, manufacturing processes, basic scientific data, or formulation information supplied to the Investigator by Dilaforette AB and not previously published) is considered confidential by Dilaforette AB and shall remain the sole property of Dilaforette AB. The Investigator agrees not to use it for other purposes without Dilaforette AB's written consent.

It is understood by the Investigator that Dilaforette AB will use the information developed in this clinical study in connection with the development of Sevuparin/DF02 and therefore may be disclosed as required to other Dilaforette AB Investigators or any appropriate international Regulatory Authorities. In order to allow for the use of information derived from this clinical study, the Investigator understands that he/she has an obligation to provide Dilaforette AB with complete test results and all data developed during this study. In accordance with generally recognized principles of scientific collaboration, co-authorship with any Dilaforette AB personnel will be discussed and mutually agreed upon before submission of a manuscript to a publisher.

15.2 Confidentiality

The principal investigator and any other study personnel involved in this study shall not disclose, or use for any purposes (other than for the performance of this study), any data, records, or other information (hereinafter collectively "Information") disclosed to the principal investigator or other study personnel. Such information shall remain the confidential and proprietary property of Dilaforette AB and shall be disclosed only to the principal investigator or other designated personnel. The obligation of non-disclosure shall not apply to the following:

- Information after such time that it is or becomes publicly available through no fault of the principal investigator or other study personnel
- Information after such time that it is disclosed to the principal investigator by a third party entitled to disclose such information.

The investigator must assure that patient anonymity will be maintained and that identities are protected from unauthorized parties. Patients should not be identified by their names but by their initials and an identification code on CRFs or other documents submitted to the sponsor. Documents that will not be submitted to Dilaforette AB (e.g., written informed consent form), should be maintained by the investigator in strict confidence.

16 MODIFICATION OF PROTOCOL AND PROTOCOL AMENDMENTS

The Investigator should not implement any deviation from, or changes of, the protocol without agreement by Dilaforette AB and prior review and documented approval/favourable opinion from the IRB/IEC of an amendment. The only exceptions are where necessary to eliminate an immediate hazard(s) to trial subjects, or when the change(s) involves only logistical or administrative aspects of the trial [e.g. change in monitor(s), change of telephone number(s)].

As soon as possible, the implemented deviation or change, the reasons for it, and, if appropriate, the proposed protocol amendment(s) should be submitted:

- to the IRB/IEC for review and approval/favourable opinion,
- to the sponsor for agreement and, if required,
- To the regulatory authority (ies), if applicable.

The party initiating an amendment must confirm it clearly in writing and it must be signed and dated by Dilaforette AB and the Principal Investigator. Dilaforette AB or designee will ensure that the Investigators submit necessary protocol amendments to the appropriate IRB/IECs.

All agreed protocol amendments must be clearly documented using standard procedures as defined by Dilaforette AB, and must be signed and dated by Dilafor AB and the Investigator.

17 REFERENCES

Accelera 0022-2008-R; Sevuparin/DF02: 14-day intravenous toxicity study in the rat

Accelera 0025-2008-P; Sevuparin/DF02: 14-day intravenous toxicity study in the beagle dog

Accelera 0050-2008-R; Sevuparin/DF02: Bacterial reverse mutation assay

Accelera 0032-2008-R; Sevuparin/DF02: Single dose intravenous toxicity study in the rat

Accelera 0048-2008-R; Sevuparin/DF02: Single dose intravenous toxicity study in the mouse

Accelera 0441-2007-R; Sevuparin/DF02: 7-day intravenous toxicity study in the rat

Accelera 0480-2007-R; Sevuparin/DF02: Preliminary intravenous toxicity study in the beagle dog

Accelera 0469-2007-R; Sevuparin/DF02: Effect on Cardiovascular Parameters after intravenous administration to the beagle dog

Anderson J, MacLean M, Davies C. Malaria research: an audit of international activity. Prism Report No. 7, The Wellcome Trust; (1996)

Carlson, J. et al. Human cerebral malaria: association with erythrocyte rosetting and lack of anti-rosetting antibodies. Lancet 336, 1457-1460 (1990).

Chen, Q. et al. Identification of Plasmodium falciparum erythrocyte membrane protein 1 (PfEMP1) as the rosetting ligand of the malaria parasite P.falciparum. J. Exp. Med. **187**, 15-23 (1998).

Chotivanich KT, Dondorp AM, White NJ, Peters K, Vreeken J, Kager PA,Udomsangpetch R. The resistance to physiological shear stresses of the erythrocytic rosettes formed by cells infected with Plasmodium falciparum. Ann Trop Med Parasitol. 2000 Apr; 94 (3):219-26.

Desakorn, V, Dondorp A, Silamut K et al. Stage-dependent production and release of histidine-rich protein 2 by plasmodium falciparum. Transaction of the Royal Society of tropical Medicine and Hygiene 2005. 99: 517 – 524.

Dondorp, A, Ince C, Charunwatthana P, et al. Direct *in vivo* assessment of microcirculatory dysfunction in severe *falciparum* malaria. JID. 2008:197 (1 January), 79 – 84.

Heddini, A. et al. Fresh isolates from children with severe Plasmodium falciparum malaria bind tomultiple receptors. Infect Immun 69, 5849-56. (2001).

Jaroonvesama, N. Intravascular coagulation in falciparum malaria. Lancet **i**, 221-223 (1972).

Le Tourneau, C. et al. Dose Escalation Methods in Phase I Cancer Clinical Trials. J Natl Cancer Inst (2009);101: 708 – 720

Munir, M., Tjandra H, Rampengan TH, Mustadjab I, Wulur FH. Heparin in the treatment of cerebral malaria. Paediatr Indones 20 **(**1-2**)**, 47-50. (1980).

Sheehy, T. W. & Reba, R. C. Complications of falciparum malaria and their treatment. Ann Int Med **66**, 807-809 (1967).

Silamut K, Phu N, Whitty C et al. A quantitative analysis of the microvascular sequestration of malaria parasites in the human brain. 1999. American Journal of Pathology, Vol 155, No 2: 395 – 410.

Sivaraman CA, Rai Chowduri AN (1983) Effect of heparin sodium on in vitro development of Plasmodium falciparum. Ind J Exp Biol 21: 247–250.

Smitskamp, H. & Wolthuis, F. H. New concepts in treatment of malignant tertian malaria with cerebral involvement. Br. Med. J. **1**, 714-716 (1971).

Trager W, Jensen JB. Human malaria parasites in continuous culture. Science.1976 Aug 20;193 (4254):673-5.

Udomsangpetch R, Pipitaporn B, Krishna S, Angus B, Pukrittayakamee S, Bates I,Suputtamongkol Y, Kyle DE, White NJ. Antimalarial drugs reduce cytoadherence and rosetting Plasmodium falciparum. J Infect Dis. 1996 Mar;173(3):691-8.

Vogt et al. Release of sequestered malaria parasites upon injection of a glycosaminoglycan. PLoS Pathog 2(9):e100. 2006. DOI: 10.1371/journal.ppat.0020100.

Vogt, A. M. et al. Heparan sulfate on endothelial cells mediates the binding of Plasmodium falciparum-infected erythrocytes via the DBL1alpha domain of PfEMP1. 2003. Blood 101, 2405-11. Epub 2002 Nov 14.

Vogt, A. M., Winter, G., Wahlgren, M. & Spillmann, D. Heparan sulfate identified on human erythrocytes: a P. falciparum receptor. 2004. Biochem J 21.

White, N. J. Assessment of the pharmacodynamic properties of antimalarial drugs *in vivo.* 1997. Antimicrobial Agents and Chemotherapy; July, Vol 41, No 7: 1413 – 1422.

Yipp BG, Anand S, Schollaardt T, Patel KD, Looareesuwan S, Ho M. Synergism of multiple adhesion molecules in mediating cytoadherence of Plasmodium falciparum-infected erythrocytes to microvascular endothelial cells under flow. Blood. 2000 Sep 15; 96 (6):2292-8.

18 APPENDICES

1. World Medical Association Declaration of Helsinki
2. Criteria for Severe Malaria
3. ICH Guidelines for Clinical Data Management, Definitions and Standards for Expedited Reporting, Topic E2.
4. Dispensing Guidelines for Sevuparin/DF02
5. Infusion Reaction Criteria
6. Antimalarial Regimen
7. Cytoadherence Assay
8. Rosetting Assay
9. CTCAE ver 4.0
10. Table of Blood and Urine volumes.

APPENDIX 1 World medical association declaration of Helsinki

Ethical Principles for Medical Research Involving Human Subjects

Adopted by the 18th WMA General Assembly, Helsinki, Finland, June 1964, and amended by the:
29th WMA General Assembly, Tokyo, Japan, October 1975
35th WMA General Assembly, Venice, Italy, October 1983
41st WMA General Assembly, Hong Kong, September 1989
48th WMA General Assembly, Somerset West, Republic of South Africa, October 1996
52nd WMA General Assembly, Edinburgh, Scotland, October 2000
53th WMA General Assembly, Washington 2002 (Note of Clarification on paragraph 29 added)
55th WMA General Assembly, Tokyo 2004 (Note of Clarification on Paragraph 30 added)
59th WMA General Assembly, Seoul, October 2008

A. INTRODUCTION

1. The World Medical Association (WMA) has developed the Declaration of Helsinki as a statement of ethical principles for medical research involving human subjects**,** including research on identifiable human material and data. The Declaration is intended to be read as a whole and each of its constituent paragraphs should not be applied without consideration of all other relevant paragraphs.

2. Although the Declaration is addressed primarily to physicians, the WMA encourages other participants in medical research involving human subjects to adopt these principles.

3. It is the duty of the physician to promote and safeguard the health of patients, including those who are involved in medical research. The physician's knowledge and conscience are dedicated to the fulfilment of this duty.

4. The Declaration of Geneva of the WMA binds the physician with the words, “The health of my patient will be my first consideration,” and the International Code of Medical Ethics declares that, “A physician shall act in the patient's best interest when providing medical care.”

5. Medical progress is based on research that ultimately must include studies involving human subjects. Populations that are underrepresented in medical research should be provided appropriate access to participation in research.

6. In medical research involving human subjects, the well-being of the individual research subject must take precedence over all other interests.

7. The primary purpose of medical research involving human subjects is to understand the causes, development and effects of diseases and improve preventive, diagnostic and therapeutic interventions (methods, procedures and treatments). Even the best current interventions must be evaluated continually through research for their safety, effectiveness, efficiency, accessibility and quality.

8. In medical practice and in medical research, most interventions involve risks and burdens.

9. Medical research is subject to ethical standards that promote respect for all human subjects and protect their health and rights. Some research populations are particularly vulnerable and need special protection. These include those who cannot give or refuse consent for themselves and those who may be vulnerable to coercion or undue influence.

10. Physicians should consider the ethical, legal and regulatory norms and standards for research involving human subjects in their own countries as well as applicable international norms and standards. No national or international ethical, legal or regulatory requirement should reduce or eliminate any of the protections for research subjects set forth in this Declaration.

B. PRINCIPLES FOR ALL MEDICAL RESEARCH

11. It is the duty of physicians who participate in medical research to protect the life, health, dignity, integrity, right to self-determination, privacy, and confidentiality of personal information of research subjects.

12. Medical research involving human subjects must conform to generally accepted scientific principles, be based on a thorough knowledge of the scientific literature, other relevant sources of information, and adequate laboratory and, as appropriate, animal experimentation. The welfare of animals used for research must be respected.

13. Appropriate caution must be exercised in the conduct of medical research that may harm the environment.

14. The design and performance of each research study involving human subjects must be clearly described in a research protocol. The protocol should contain a statement of the ethical considerations involved and should indicate how the principles in this Declaration have been addressed. The protocol should include information regarding funding, sponsors, institutional affiliations other potential conflicts of interest, incentives for subjects and provisions for treating and/or compensating subjects who are harmed as a consequence of participation in the research study. The protocol should describe arrangements for post-study access by study subjects to interventions identified as beneficial in the study or access to other appropriate care or benefits.

15. The research protocol must be submitted for consideration, comment, guidance and approval to a research ethics committee before the study begins. This committee must be independent of the researcher, the sponsor and any other undue influence. It must take into consideration the laws and regulations of the country or countries in which the research is to be performed as well as applicable international norms and standards but these must not be allowed to reduce or eliminate any of the protections for research subjects set forth in this Declaration. The committee must have the right to monitor ongoing studies. The researcher must provide monitoring information to the committee, especially information about any serious adverse events. No change to the protocol may be made without consideration and approval by the committee.

16. Medical research involving human subjects must be conducted only by individuals with the appropriate scientific training and qualifications. Research on patients or healthy volunteers requires the supervision of a competent and appropriately qualified physician or other health care professional. The responsibility for the protection of research subjects must always rest with the physician or other health care professional and never the research subjects, even though they have given consent.

17. Medical research involving a disadvantaged or vulnerable population or community is only justified if the research is responsive to the health needs and priorities of this population or community and if there is a reasonable likelihood that this population or community stands to benefit from the results of the research.

18. Every medical research study involving human subjects must be preceded by careful assessment of predictable risks and burdens to the individuals and communities involved in the research in comparison with foreseeable benefits to them and to other individuals or communities affected by the condition under investigation.

19. Every clinical trial must be registered in a publicly accessible database before recruitment of the first subject.

20. Physicians may not participate in a research study involving human subjects unless they are confident that the risks involved have been adequately assessed and can be satisfactorily managed. Physicians must immediately stop a study when the risks are found to outweigh the potential benefits or when there is conclusive proof of positive and beneficial results.

21. Medical research involving human subjects may only be conducted if the importance of the objective outweighs the inherent risks and burdens to the research subjects.

22. Participation by competent individuals as subjects in medical research must be voluntary. Although it may be appropriate to consult family members or community leaders, no competent individual may be enrolled in a research study unless he or she freely agrees.

23. Every precaution must be taken to protect the privacy of research subjects and the confidentiality of their personal information and to minimize the impact of the study on their physical, mental and social integrity.

24. In medical research involving competent human subjects, each potential subject must be adequately informed of the aims, methods, sources of funding, any possible conflicts of interest, institutional affiliations of the researcher, the anticipated benefits and potential risks of the study and the discomfort it may entail, and any other relevant aspects of the study. The potential subject must be informed of the right to refuse to participate in the study or to withdraw consent to participate at any time without reprisal. Special attention should be given to the specific information needs of individual potential subjects as well as to the methods used to deliver the information. After ensuring that the potential subject has understood the information, the physician or another appropriately qualified individual must then seek the potential subject’s freely-given informed consent, preferably in writing. If the consent cannot be expressed in writing, the non-written consent must be formally documented and witnessed.

25. For medical research using identifiable human material or data, physicians must normally seek consent for the collection, analysis, storage and/or reuse. There may be situations where consent would be impossible or impractical to obtain for such research or would pose a threat to the validity of the research. In such situations the research may be done only after consideration and approval of a research ethics committee.

26. When seeking informed consent for participation in a research study the physician should be particularly cautious if the potential subject is in a dependent relationship with the physician or may consent under duress. In such situations the informed consent should be sought by an appropriately qualified individual who is completely independent of this relationship.

27. For a potential research subject who is incompetent, the physician must seek informed consent from the legally authorized representative. These individuals must not be included in a research study that has no likelihood of benefit for them unless it is intended to promote the health of the population represented by the potential subject, the research cannot instead be performed with competent persons, and the research entails only minimal risk and minimal burden.

28. When a potential research subject who is deemed incompetent is able to give assent to decisions about participation in research, the physician must seek that assent in addition to the consent of the legally authorized representative. The potential subject's dissent should be respected.

29. Research involving subjects who are physically or mentally incapable of giving consent, for example, unconscious patients, may be done only if the physical or mental condition that prevents giving informed consent is a necessary characteristic of the research population. In such circumstances the physician should seek informed consent from the legally authorized representative. If no such representative is available and if the research cannot be delayed, the study may proceed without informed consent provided that the specific reasons for involving subjects with a condition that renders them unable to give informed consent have been stated in the research protocol and the study has been approved by a research ethics committee. Consent to remain in the research should be obtained as soon as possible from the subject or a legally authorized representative.

30. Authors, editors and publishers all have ethical obligations with regard to the publication of the results of research. Authors have a duty to make publicly available the results of their research on human subjects and are accountable for the completeness and accuracy of their reports. They should adhere to accepted guidelines for ethical reporting. Negative and inconclusive as well as positive results should be published or otherwise made publicly available. Sources of funding, institutional affiliations and conflicts of interest should be declared in the publication. Reports of research not in accordance with the principles of this Declaration should not be accepted for publication.

C. ADDITIONAL PRINCIPLES FOR MEDICAL RESEARCH COMBINED WITH MEDICAL CARE

31. The physician may combine medical research with medical care only to the extent that the research is justified by its potential preventive, diagnostic or therapeutic value and if

the physician has good reason to believe that participation in the research study will not adversely affect the health of the patients who serve as research subjects.

32. The benefits, risks, burdens and effectiveness of a new intervention must be tested against those of the best current proven intervention, except in the following circumstances:

    a. The use of placebo, or no treatment, is acceptable in studies where no current proven intervention exists; or

    b. Where for compelling and scientifically sound methodological reasons the use of placebo is necessary to determine the efficacy or safety of an intervention and the patients who receive placebo or no treatment will not be subject to any risk of serious or irreversible harm. Extreme care must be taken to avoid abuse of this option.

33. At the conclusion of the study, patients entered into the study are entitled to be informed about the outcome of the study and to share any benefits that result from it, for example, access to interventions identified as beneficial in the study or to other appropriate care or benefits.

34. The physician must fully inform the patient which aspects of the care are related to the research. The refusal of a patient to participate in a study or the patient's decision to withdraw from the study must never interfere with the patient-physician relationship.

35. In the treatment of a patient, where proven interventions do not exist or have been ineffective, the physician, after seeking expert advice, with informed consent from the patient or a legally authorized representative, may use an unproven intervention if in the physician's judgment it offers hope of saving life, re-establishing health or alleviating suffering. Where possible, this intervention should be made the object of research, designed to evaluate its safety and efficacy. In all cases, new information should be recorded and, where appropriate, made publicly available.

APPENDIX 2 Criteria For Severe Malaria: WHO 2010

WHO Criteria for Severe Malaria:
Clinical features:<br>– impaired consciousness or unrousable coma<br>– prostration, i.e. generalized weakness so that the patient is unable walk or sit up without assistance<br>– failure to feed<br>– multiple convulsions – more than two episodes in 24 h<br>– deep breathing, respiratory distress (acidotic breathing)<br>– circulatory collapse or shock, systolic blood pressure < 70 mm Hg in adults and < 50 mm Hg in children<br>– clinical jaundice plus evidence of other vital organ dysfunction<br>– haemoglobinuria<br>– abnormal spontaneous bleeding<br>– pulmonary oedema (radiological)<br>*Laboratory findings:*<br>– hypoglycaemia (blood glucose < 2.2 mmol/l or < 40 mg/dl)<br>– metabolic acidosis (plasma bicarbonate < 15 mmol/l)<br>– severe normocytic anaemia (Hb < 5 g/dl, packed cell volume < 15%)<br>– haemoglobinuria<br>– hyperparasitaemia (> 2%/100 000/μl in low intensity transmission areas or > 5% or 250 000/μl in areas of high stable malaria transmission intensity)<br>– hyperlactataemia (lactate > 5 mmol/l)<br>– renal impairment (serum creatinine > 265 μmol/l).

APPENDIX 3 ICH Guidelines for Clinical Safety Data Management, Definitions and Standards for Expedited Reporting, Topic E2

A Serious Adverse Event (SAE) is any experience that suggests a significant hazard, contraindications, side effect or precaution. It is any Adverse Event that at any dose fulfils at least one of the following criteria:

- Is fatal (results in death)

(Note: death is an outcome, not an event).

- Is life threatening

(Note: the term “Life threatening” refers to an event in which the patient was at immediate risk of death at the time of the event, it does not refer to an event which could hypothetically have caused a death it been more severe).

- Required in patient hospitalization or prolongation of existing hospitalization
- Results in persistent or significant disability/incapacity
- Is a congenital anomaly/birth defect
- Is medically significant or requires intervention to prevent one or other of the outcomes listed above.

Medical and scientific judgment should be exercised in deciding whether expedited reporting to the sponsor is appropriate on other situations, such as important medical events that may not be immediately life-threatening or result in death or hospitalization but may jeopardize the patient or may require intervention to prevent one of the outcomes listed in the definitions above. These situations should also usually be considered serious.

Examples of such events are intensive treatment in an emergency room or at home for allergic bronchospasm, blood dyscrasias or convulsions that do not result in hospitalization, or development of drug dependency or drug abuse.

An unexpected Adverse Event is one, the nature or severity of which is not consistent with the applicable product information.

Causality is initially assessed by the investigator. For SAEs, causality can be one of the two possibilities:

- No (unrelated, equals not drug related)
- Yes (possibly, probably or definitely drug related; or relationship not provided).

The term severe is a measure of intensity, thus a severe adverse event is not necessarily serious. For example, nausea of several hours duration may be rated as severe, but may not be clinically serious.

A serious Adverse Event occurring during the study or which comes to the attention of the investigator within 28 days after stopping the treatment or during the protocol defined follow-up period, if this is longer, whether considered treatment related or not, must be reported.

Additionally, a SAE event that occurs after this time, if considered related to test “drug”, should be reported.

ICH Guidelines for Clinical Safety Data Management, Definitions and Standards for Expedited Reporting, Topic E2 (cont’d.)

Progression of neoplasia should not be reported as an AE or SAE. Findings that are clearly consistent with the expected progression of the underlying cancer should not be reported as an AE, and hospitalizations due to the progression of cancer do not necessarily qualify for a SAE. If there is any uncertainty about a finding being due solely to progression of neoplasia, the finding should be reported as an AE or SAE as appropriate.

Such preliminary reports will be followed by detailed descriptions later, which will include copies of hospital case reports, autopsy reports and other documents when requested and applicable.

For SAEs, the following must be assessed and recorded on the adverse event page of the CRF: intensity, relationship to test substance, action taken, and outcome.

The investigator must notify the Ethics Review Committee/Institutional Review Board of a SAE in writing as soon as is practical and in accordance with international and local laws and regulations.

APPENDIX 4 Dispensing Guidelines For Sevuparin/Df02

Calculations; Calculations must always be checked by a pharmacist or experienced study personnel allowed to prepare study drug.

Step A: Calculate the amount of Sevuparin/DF02 150mg/ml needed

1. Indicate Dose to patient
2. Indicate Body Weight
3. Calculate amount needed per dose
4.

Please fill in

ID	Dose (D)	Body weight (BW)	Amount Sevuparin/DF02 /dose (A)
	D mg/kg	BW kg	A=BW*D mg
	D=_____mg/kg	BW= ______kg	A=____ mg

Step B: Method for preparation of final dilution. The four doses for one patient will be prepared once daily and kept refridgerated.

1. Calculate the Volume Sevuparin/DF02 needed by:
$V_{Sevuparin/DF02}$ = A mg/ 150 mg/ml= _____ ml
2. Calculate the Volume of 0.9% NaCl (USP for injection) needed by
V_{NaCl} =21 - $V_{Sevuparin/DF02}$ =_____ ml
3. Label the vial(s) with patient number
4. Swab the rubber stopper of the top of the Sevuparin/DF02 vial
5. Swab the port of a sodium chloride 0.9% bottle (USP for injection)
6. Using a 2, 5 or 15 ml Syringe withdraw $V_{Sevuparin/DF02}$ ml of Sevuparin/DF02 solution from the vial
7. Fill Syringe 1 with $V_{Sevuparin/DF02}$ calculated ml.
8. Fill Syringe 2 (Luer-Lok™ Syringe 30 ml, BD Plastipack, Ref 301229) with V_{NaCl}.
9. Connect the two syringes and mix 1 minute but carefully pushing the solutions back and forth.
10. End with all solution in Syringe 2. Discharge Syringe 2 and close it.
11. Prepare 4 items of 30 ml syringes
12. Keep the syringes in +2-8°C
13. For use within 24 hours
14. Let one syringe adjust to room temperature before infusion
15. Infusion of 20 ml (4 ml/min for 5 minutes)
16. After injection, weight the syringe and note the results.

APPENDIX 5 Infusion Reaction Criteria

For grading of AEs see appendix 9.

Epinephrine (1:1000) for subcutaneous injection, diphenhydramine (12.5 mg to 50 mg) for IV injection, and any other medications and resuscitation equipment for the emergency management of anaphylactic reactions must be available in the room where the infusions are being performed.

Section 1
The subject's study treatment must be discontinued *immediately* if a Grade 4 AE occurs during the infusion of Sevuparin/DF02 and the subject must be withdrawn from the study. The subject's study treatment must be discontinued *immediately* if a Grade 3 AE occurs during the short infusion of Sevuparin/DF02 and should not be completed even if the subject recovers. Subsequent administration of Sevuparin/DF02 should be preceded by prophylactic premedication as outlined in Section 2. If another Grade 3 infusion reaction occurs during a subsequent treatment day, treatment will again be discontinued for the rest of that infusion. **If the investigator wishes to continue treatment in this subject, there should be discussion with the medical monitor about the possibility of reduced infusion rates for subsequent infusions.**

If Grade 1 or 2 AEs occur during infusion of the subject may complete the treatment at half the previous infusion rate. Subsequent administration of should be preceded with prophylactic treatment as outlined in Section 2. To assess the events, please refer to the NCI CTCAE in the Study Reference Manual.

Treatment Recommendations Prior to, During, and Immediately After Infusion
Section 2 Prophylactic Treatment Prior to Infusion
Prophylactic measures prior to the administration of Sevuparin/DF02 should not be initiated unless a previous infusion reaction has occurred. Subjects who have previously experienced a reaction to Sevuparin/DF02 must receive prophylactic treatment prior to subsequent infusions. Prophylactic treatment with acetaminophen (paracetamol) (1 g) and diphenhydramine HCl (12.5 to 50 mg; or equivalent dose of a similar agent) by mouth 30 to 60 minutes prior to the start of the infusion is recommended. **Steroids should not be used as pre-medication in this study**.

Section 3 Treatment of Infusion Reactions
The use of acetaminophen (paracetamol) plus an antihistamine, such as diphenhydramine (or similar agent), is recommended for the treatment of an infusion reaction. For adjustment to the subject's infusion see Section 1. **Modification of the Subject's Treatment During Therapy.** Acetaminophen and diphenhydramine should be repeated as clinically indicated for the infusion reaction. Vital sign measurements will be recorded on the appropriate CRF. Any infusion reaction will be recorded as an AE on the AE CRF. Management of Grade 3 or Grade 4 infusion reaction is outlined in Section 1. These recommendations do not address life-threatening events, including anaphylaxis, for which study treatment should be discontinued and all appropriate standard measures, including full resuscitation medicine and equipment, must be available and should be used as clinically indicated.

APPENDIX 6 Anti-malarial regimen to be used during the study

5. Every patient will be treated with Atovaquone-proguanil (Malanil®/Malanil®) as antimalarial drugs

MALANIL - atovaquone and proguanil hydrochloride tablet, film coated
SmithKline Beecham Corporation

MALANIL®
(atovaquone and proguanil hydrochloride)
Tablets
MALANIL®
(atovaquone and proguanil hydrochloride)
Pediatric Tablets

DESCRIPTION

MALANIL (atovaquone and proguanil hydrochloride) is a fixed-dose combination of the antimalarial agents atovaquone and proguanil hydrochloride. The chemical name of atovaquone is trans-2-[4-(4-chlorophenyl)cyclohexyl]-3-hydroxy-1,4-naphthalenedione. Atovaquone is a yellow crystalline solid that is practically insoluble in water. It has a molecular weight of 366.84 and the molecular formula C22H19ClO3. The compound has the following structural formula:

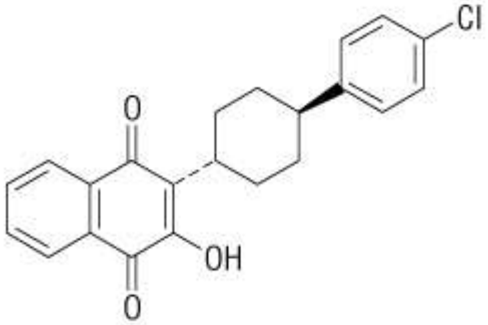

The chemical name of proguanil hydrochloride is 1-(4-chlorophenyl)-5-isopropyl-biguanide hydrochloride. Proguanil hydrochloride is a white crystalline solid that is sparingly soluble in water. It has a molecular weight of 290.22 and the molecular formula C11H16ClN5•HCl. The compound has the following structural formula:

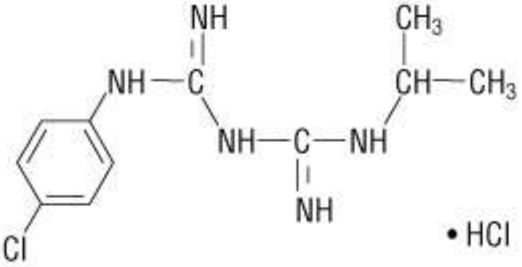

MALANIL Tablets and MALANIL Pediatric Tablets are for oral administration. Each MALANIL Tablet contains 250 mg of atovaquone and 100 mg of proguanil hydrochloride and each MALANIL Pediatric Tablet contains 62.5 mg of atovaquone and 25 mg of proguanil hydrochloride. The inactive ingredients in both tablets are low-substituted hydroxypropyl cellulose, magnesium stearate, microcrystalline cellulose, poloxamer 188, povidone K30, and sodium starch glycolate. The tablet coating contains hypromellose, polyethylene glycol 400, polyethylene glycol 8000, red iron oxide, and titanium dioxide.

CLINICAL PHARMACOLOGY

Microbiology

Mechanism of Action

The constituents of MALANIL, atovaquone and proguanil hydrochloride, interfere with 2 different pathways involved in the biosynthesis of pyrimidines required for nucleic acid replication. Atovaquone is a selective inhibitor of parasite mitochondrial electron transport. Proguanil hydrochloride primarily exerts its effect by means of the metabolite cycloguanil, a dihydrofolate reductase inhibitor. Inhibition of dihydrofolate reductase in the malaria parasite disrupts deoxythymidylate synthesis.

Activity *In Vitro* and In Vivo

Atovaquone and cycloguanil (an active metabolite of proguanil) are active against the erythrocytic and exoerythrocytic stages of Plasmodium spp. Enhanced efficacy of the combination compared to either atovaquone or proguanil hydrochloride alone was demonstrated in clinical studies in both immune and non-immune patients (see CLINICAL STUDIES).

Drug Resistance

Strains of P. falciparum with decreased susceptibility to atovaquone or proguanil/cycloguanil alone can be selected in vitro or in vivo. The combination of atovaquone and proguanil hydrochloride may not be effective for treatment of recrudescent malaria that develops after prior therapy with the combination.

Pharmacokinetics

Absorption

Atovaquone is a highly lipophilic compound with low aqueous solubility. The bioavailability of atovaquone shows considerable inter-individual variability.

Dietary fat taken with atovaquone increases the rate and extent of absorption, increasing AUC 2 to 3 times and Cmax 5 times over fasting. The absolute bioavailability of the tablet formulation of atovaquone when taken with food is 23%. MALANIL Tablets should be taken with food or a milky drink.

Proguanil hydrochloride is extensively absorbed regardless of food intake.

Distribution

Atovaquone is highly protein bound (>99%) over the concentration range of 1 to 90 mcg/mL. A population pharmacokinetic analysis demonstrated that the apparent volume of distribution of atovaquone (V/F) in adult and pediatric patients after oral administration is approximately 8.8 L/kg.

Proguanil is 75% protein bound. A population pharmacokinetic analysis demonstrated that the apparent V/F of proguanil in adult and pediatric patients >15 years of age with body weights from 31 to 110 kg ranged from 1,617 to 2,502 L. In pediatric patients ≤15 years of age with body weights from 11 to 56 kg, the V/F of proguanil ranged from 462 to 966 L.

In human plasma, the binding of atovaquone and proguanil was unaffected by the presence of the other.

Metabolism

In a study where 14C-labeled atovaquone was administered to healthy volunteers, greater than 94% of the dose was recovered as unchanged atovaquone in the feces over 21 days. There was little or no excretion of atovaquone in the urine (less than 0.6%). There is indirect evidence that atovaquone may undergo limited metabolism; however, a specific metabolite has not been identified. Between 40% to 60% of proguanil is excreted by the kidneys. Proguanil is metabolized to cycloguanil (primarily via CYP2C19) and 4-chlorophenylbiguanide. The main routes of elimination are hepatic biotransformation and renal excretion.

Elimination

The elimination half-life of atovaquone is about 2 to 3 days in adult patients.

The elimination half-life of proguanil is 12 to 21 hours in both adult patients and pediatric patients, but may be longer in individuals who are slow metabolizers.

A population pharmacokinetic analysis in adult and pediatric patients showed that the apparent clearance (CL/F) of both atovaquone and proguanil are related to the body weight. The values CL/F for both atovaquone and proguanil in subjects with body weight ≥11 kg are shown in Table 1.

Table 1. Apparent Clearance for Atovaquone and Proguanil in Patients as a Function of Body Weight

		Atovaquone		Proguanil
Body Weight	N	CL/F (L/hr) Mean ± SD* (range)	N	CL/F (L/hr) Mean ± SD* (range)
11-20 kg	159	1.34 ± 0.63 (0.52-4.26)	146	29.5 ± 6.5 (10.3-48.3)
21-30 kg	117	1.87 ± 0.81 (0.52-5.38)	113	40.0 ± 7.5 (15.9-62.7)
31-40 kg	95	2.76 ± 2.07 (0.97-12.5)	91	49.5 ± 8.30 (25.8-71.5)
>40 kg	368	6.61 ± 3.92 (1.32-20.3)	282	67.9 ± 19.9 (14.0-145)

* SD = standard deviation.

The pharmacokinetics of atovaquone and proguanil in patients with body weight below 11 kg have not been adequately characterized.

Special Populations

Pediatrics
The pharmacokinetics of proguanil and cycloguanil are similar in adult patients and pediatric patients. However, the elimination half-life of atovaquone is shorter in pediatric patients (1 to 2 days) than in adult patients (2 to 3 days). In clinical trials, plasma trough levels of atovaquone and proguanil in pediatric patients weighing 5 to 40 kg were within the range observed in adults after dosing by body weight.

Geriatrics
In a single-dose study, the pharmacokinetics of atovaquone, proguanil, and cycloguanil were compared in 13 elderly subjects (age 65 to 79 years) to 13 younger subjects (age 30 to 45 years). In the elderly subjects, the extent of systemic exposure (AUC) of cycloguanil was increased (point estimate = 2.36, CI = 1.70, 3.28). Tmax was longer in elderly subjects (median 8 hours) compared with younger subjects (median 4 hours) and average elimination half-life was longer in elderly subjects (mean 14.9 hours) compared with younger subjects (mean 8.3 hours).

Hepatic Impairment
In a single-dose study, the pharmacokinetics of atovaquone, proguanil, and cycloguanil were compared in 13 subjects with hepatic impairment (9 mild, 4 moderate, as indicated by the Child-Pugh method) to 13 subjects with normal hepatic function. In subjects with mild or moderate hepatic impairment as compared to healthy subjects, there were no marked differences (<50%) in the rate or extent of systemic exposure of atovaquone. However, in subjects with moderate hepatic impairment, the elimination half-life of atovaquone was increased (point estimate = 1.28, 90% CI = 1.00 to 1.63). Proguanil AUC, Cmax, and its t1/2 increased in subjects with mild hepatic impairment when compared to healthy subjects (Table 2). Also, the proguanil AUC and its t1/2 increased in subjects with moderate hepatic impairment when compared to healthy subjects. Consistent with the increase in proguanil AUC, there were marked decreases in the systemic exposure of cycloguanil (Cmax and AUC) and an increase in its elimination half-life in subjects with mild hepatic impairment when compared to healthy volunteers (Table 2). There were few measurable cycloguanil concentrations in subjects with moderate hepatic impairment (see DOSAGE AND ADMINISTRATION). The pharmacokinetics of atovaquone, proguanil, and cycloguanil after administration of MALANIL have not been studied in patients with severe hepatic impairment.

Table 2. Point Estimates (90% CI) for Proguanil and Cycloguanil Parameters in Subjects With Mild and Moderate Hepatic Impairment Compared to Healthy Volunteers

Parameter	Comparison	Proguanil	Cycloguanil

AUC(0-inf)*	mild:healthy	1.96 (1.51, 2.54)	0.32 (0.22, 0.45)
Cmax*	mild:healthy	1.41 (1.16, 1.71)	0.35 (0.24, 0.50)
t1/2†	mild:healthy	1.21 (0.92, 1.60)	0.86 (0.49, 1.48)
AUC(0-inf)*	moderate:healthy	1.64 (1.14, 2.34)	ND
Cmax*	moderate:healthy	0.97 (0.69, 1.36)	ND
t1/2†	moderate:healthy	1.46 (1.05, 2.05)	ND

ND = not determined due to lack of quantifiable data.

* Ratio of geometric means.

† Mean difference.

Renal Impairment

In patients with mild renal impairment (creatinine clearance 50 to 80 mL/min), oral clearance and/or AUC data for atovaquone, proguanil, and cycloguanil are within the range of values observed in patients with normal renal function (creatinine clearance >80 mL/min). In patients with moderate renal impairment (creatinine clearance 30 to 50 mL/min), mean oral clearance for proguanil was reduced by approximately 35% compared with patients with normal renal function (creatinine clearance >80 mL/min) and the oral clearance of atovaquone was comparable between patients with normal renal function and mild renal impairment. No data exist on the use of MALANIL for long-term prophylaxis (over 2 months) in individuals with moderate renal failure. In patients with severe renal impairment (creatinine clearance <30 mL/min), atovaquone Cmax and AUC are reduced but the elimination half-lives for proguanil and cycloguanil are prolonged, with corresponding increases in AUC, resulting in the potential of drug accumulation and toxicity with repeated dosing (see CONTRAINDICATIONS).

Drug Interactions

There are no pharmacokinetic interactions between atovaquone and proguanil at the recommended dose.

Concomitant treatment with tetracycline has been associated with approximately a 40% reduction in plasma concentrations of atovaquone.

Concomitant treatment with metoclopramide has also been associated with decreased bioavailability of atovaquone.

Concomitant administration of rifampin or rifabutin is known to reduce atovaquone levels by approximately 50% and 34%, respectively (see PRECAUTIONS: Drug Interactions). The mechanisms of these interactions are unknown.

Concomitant administration of atovaquone (750 mg BID with food for 14 days) and indinavir (800 mg TID without food for 14 days) did not result in any change in the steady-state AUC and Cmax of indinavir but resulted in a decrease in the Ctrough of indinavir (23% decrease [90% CI

8%, 35%]). Caution should be exercised when prescribing atovaquone with indinavir due to the decrease in trough levels of indinavir.

Atovaquone is highly protein bound (>99%) but does not displace other highly protein-bound drugs in vitro, indicating significant drug interactions arising from displacement are unlikely (see PRECAUTIONS: Drug Interactions). Proguanil is metabolized primarily by CYP2C19. Potential pharmacokinetic interactions with other substrates or inhibitors of this pathway are unknown.

INDICATIONS AND USAGE

Prevention of Malaria

MALANIL is indicated for the prophylaxis of P. falciparum malaria, including in areas where chloroquine resistance has been reported (see CLINICAL STUDIES).

Treatment of Malaria

MALANIL is indicated for the treatment of acute, uncomplicated P. falciparum malaria. MALANIL has been shown to be effective in regions where the drugs chloroquine, halofantrine, mefloquine, and amodiaquine may have unacceptable failure rates, presumably due to drug resistance.

CONTRAINDICATIONS

MALANIL is contraindicated in individuals with known hypersensitivity to atovaquone or proguanil hydrochloride or any component of the formulation. Rare cases of anaphylaxis following treatment with atovaquone/proguanil have been reported.

MALANIL is contraindicated for prophylaxis of P. falciparum malaria in patients with severe renal impairment (creatinine clearance <30 mL/min) (see CLINICAL PHARMACOLOGY: Special Populations: Renal Impairment).

PRECAUTIONS

General

MALANIL has not been evaluated for the treatment of cerebral malaria or other severe manifestations of complicated malaria, including hyperparasitemia, pulmonary edema, or renal failure. Patients with severe malaria are not candidates for oral therapy.

Elevated liver function tests and rare cases of hepatitis have been reported with prophylactic use of MALANIL. A single case of hepatic failure requiring liver transplantation has also been reported with prophylactic use.

Absorption of atovaquone may be reduced in patients with diarrhea or vomiting. If MALANIL is used in patients who are vomiting (see DOSAGE AND ADMINISTRATION), parasitemia should be closely monitored and the use of an antiemetic considered. Vomiting occurred in up to 19% of pediatric patients given treatment doses of MALANIL. In the controlled clinical trials of MALANIL, 15.3% of adults who were treated with atovaquone/proguanil received an antiemetic drug during that part of the trial when they received atovaquone/proguanil. Of these patients,

98.3% were successfully treated. In patients with severe or persistent diarrhea or vomiting, alternative antimalarial therapy may be required.

Parasite relapse occurred commonly when P. vivax malaria was treated with MALANIL alone.

In the event of recrudescent P. falciparum infections after treatment with MALANIL or failure of chemoprophylaxis with MALANIL, patients should be treated with a different blood schizonticide.

Information for Patients

Patients should be instructed:

- to take MALANIL tablets at the same time each day with food or a milky drink.
- to take a repeat dose of MALANIL if vomiting occurs within 1 hour after dosing.
- to take a dose as soon as possible if a dose is missed, then return to their normal dosing schedule. However, if a dose is skipped, the patient should not double the next dose.
- that rare serious adverse events such as hepatitis, severe skin reactions, neurological, and hematological events have been reported when MALANIL was used for the prophylaxis or treatment of malaria.
- to consult a healthcare professional regarding alternative forms of prophylaxis if prophylaxis with MALANIL is prematurely discontinued for any reason.
- that protective clothing, insect repellents, and bednets are important components of malaria prophylaxis.
- that no chemoprophylactic regimen is 100% effective; therefore, patients should seek medical attention for any febrile illness that occurs during or after return from a malaria-endemic area and inform their healthcare professional that they may have been exposed to malaria.
- that falciparum malaria carries a higher risk of death and serious complications in pregnant women than in the general population. Pregnant women anticipating travel to malarious areas should discuss the risks and benefits of such travel with their physicians (see Pregnancy section).

Drug Interactions

Concomitant treatment with tetracycline has been associated with approximately a 40% reduction in plasma concentrations of atovaquone. Parasitemia should be closely monitored in patients receiving tetracycline. While antiemetics may be indicated for patients receiving MALANIL, metoclopramide may reduce the bioavailability of atovaquone and should be used only if other antiemetics are not available.

Concomitant administration of rifampin or rifabutin is known to reduce atovaquone levels by approximately 50% and 34%, respectively. The concomitant administration of MALANIL and rifampin or rifabutin is not recommended.

Proguanil may potentiate the anticoagulant effect of warfarin and other coumarin-based anticoagulants. The mechanism of this potential drug interaction has not been established. Caution is advised when initiating or withdrawing malaria prophylaxis or treatment with

MALANIL in patients on continuous treatment with coumarin-based anticoagulants. When these products are administered concomitantly, suitable coagulation tests should be closely monitored.

Atovaquone is highly protein bound (>99%) but does not displace other highly protein-bound drugs in vitro, indicating significant drug interactions arising from displacement are unlikely.

Potential interactions between proguanil or cycloguanil and other drugs that are CYP2C19 substrates or inhibitors are unknown.

Carcinogenesis, Mutagenesis, Impairment of Fertility

Atovaquone

Carcinogenicity studies in rats were negative; 24-month studies in mice showed treatment-related increases in incidence of hepatocellular adenoma and hepatocellular carcinoma at all doses tested which ranged from approximately 5 to 8 times the average steady-state plasma concentrations in humans during prophylaxis of malaria. Atovaquone was negative with or without metabolic activation in the Ames Salmonella mutagenicity assay, the Mouse Lymphoma mutagenesis assay, and the Cultured Human Lymphocyte cytogenetic assay. No evidence of genotoxicity was observed in the in vivo Mouse Micronucleus assay.

Proguanil

No evidence of a carcinogenic effect was observed in 24-month studies conducted in CD-1 mice (doses up to 1.5 times the average systemic human exposure based on AUC) and in Wistar Hannover rats (doses up to 1.1 times the average systemic human exposure).

Proguanil was negative with or without metabolic activation in the Ames Salmonella mutagenicity assay and the Mouse Lymphoma mutagenesis assay. No evidence of genotoxicity was observed in the in vivo Mouse Micronucleus assay.

Cycloguanil, the active metabolite of proguanil, was also negative in the Ames test, but was positive in the Mouse Lymphoma assay and the Mouse Micronucleus assay. These positive effects with cycloguanil, a dihydrofolate reductase inhibitor, were significantly reduced or abolished with folinic acid supplementation.

Genotoxicity studies have not been performed with atovaquone in combination with proguanil. Effects of MALANIL on male and female reproductive performance are unknown.

Pregnancy

Pregnancy Category C. Falciparum malaria carries a higher risk of morbidity and mortality in pregnant women than in the general population. Maternal death and fetal loss are both known complications of falciparum malaria in pregnancy. In pregnant women who must travel to malaria-endemic areas, personal protection against mosquito bites should always be employed (see Information for Patients) in addition to antimalarials.

Atovaquone was not teratogenic and did not cause reproductive toxicity in rats at maternal plasma concentrations up to 5 to 6.5 times the estimated human exposure during treatment of malaria. Following single-dose administration of 14C-labeled atovaquone to pregnant rats, concentrations of radiolabel in rat fetuses were 18% (mid-gestation) and 60% (late gestation) of concurrent maternal plasma concentrations. In rabbits, atovaquone caused maternal toxicity at plasma concentrations that were approximately 0.6 to 1.3 times the estimated human exposure during treatment of malaria. Adverse fetal effects in rabbits, including decreased fetal body

lengths and increased early resorptions and post-implantation losses, were observed only in the presence of maternal toxicity. Concentrations of atovaquone in rabbit fetuses averaged 30% of the concurrent maternal plasma concentrations.

The combination of atovaquone and proguanil hydrochloride was not teratogenic in rats at plasma concentrations up to 1.7 and 0.10 times, respectively, the estimated human exposure during treatment of malaria. In rabbits, the combination of atovaquone and proguanil hydrochloride was not teratogenic or embryotoxic to rabbit fetuses at plasma concentrations up to 0.34 and 0.82 times, respectively, the estimated human exposure during treatment of malaria.

While there are no adequate and well-controlled studies of atovaquone and/or proguanil hydrochloride in pregnant women, MALANIL may be used if the potential benefit justifies the potential risk to the fetus. The proguanil component of MALANIL acts by inhibiting the parasitic dihydrofolate reductase (see CLINICAL PHARMACOLOGY: Microbiology: Mechanism of Action). However, there are no clinical data indicating that folate supplementation diminishes drug efficacy, and for women of childbearing age receiving folate supplements to prevent neural tube birth defects, such supplements may be continued while taking MALANIL.

Nursing Mothers

It is not known whether atovaquone is excreted into human milk. In a rat study, atovaquone concentrations in the milk were 30% of the concurrent atovaquone concentrations in the maternal plasma.

Proguanil is excreted into human milk in small quantities.

Caution should be exercised when MALANIL is administered to a nursing woman.

Pediatric Use

Treatment of Malaria

The efficacy and safety of MALANIL for the treatment of malaria have been established in controlled studies involving pediatric patients weighing 5 kg or more (see CLINICAL STUDIES). Safety and effectiveness have not been established in pediatric patients who weigh less than 5 kg.

Prophylaxis of Malaria

The efficacy and safety of MALANIL have been established for the prophylaxis of malaria in controlled studies involving pediatric patients weighing 11 kg or more (see CLINICAL STUDIES). Safety and effectiveness have not been established in pediatric patients who weigh less than 11 kg.

Geriatric Use

Clinical studies of MALANIL did not include sufficient numbers of subjects aged 65 and over to determine whether they respond differently from younger subjects. In general, dose selection for an elderly patient should be cautious, reflecting the greater frequency of decreased hepatic, renal, or cardiac function, the higher systemic exposure to cycloguanil (see CLINICAL

PHARMACOLOGY: Special Populations: Geriatrics), and the greater frequency of concomitant disease or other drug therapy.

ADVERSE REACTIONS

Because MALANIL contains atovaquone and proguanil hydrochloride, the type and severity of adverse reactions associated with each of the compounds may be expected. The higher treatment doses of MALANIL were less well tolerated than the lower prophylactic doses.

Among adults who received MALANIL for treatment of malaria, attributable adverse experiences that occurred in ≥5% of patients were abdominal pain (17%), nausea (12%), vomiting (12%), headache (10%), diarrhea (8%), asthenia (8%), anorexia (5%), and dizziness (5%). Treatment was discontinued prematurely due to an adverse experience in 4 of 436 adults treated with MALANIL.

Among pediatric patients (weighing 11 to 40 kg) who received MALANIL for the treatment of malaria, attributable adverse experiences that occurred in ≥5% of patients were vomiting (10%) and pruritus (6%). Vomiting occurred in 43 of 319 (13%) pediatric patients who did not have symptomatic malaria but were given treatment doses of MALANIL for 3 days in a clinical trial. The design of this clinical trial required that any patient who vomited be withdrawn from the trial. Among pediatric patients with symptomatic malaria treated with MALANIL, treatment was discontinued prematurely due to an adverse experience in 1 of 116 (0.9%).

In a study of 100 pediatric patients (5 to <11 kg body weight) who received MALANIL for the treatment of uncomplicated P. falciparum malaria, only diarrhea (6%) occurred in ≥5% of patients as an adverse experience attributable to MALANIL. In 3 patients (3%), treatment was discontinued prematurely due to an adverse experience.

Abnormalities in laboratory tests reported in clinical trials were limited to elevations of transaminases in malaria patients being treated with MALANIL. The frequency of these abnormalities varied substantially across studies of treatment and were not observed in the randomized portions of the prophylaxis trials.

In one phase III trial of malaria treatment in Thai adults, early elevations of ALT and AST were observed to occur more frequently in patients treated with MALANIL compared to patients treated with an active control drug. Rates for patients who had normal baseline levels of these clinical laboratory parameters were: Day 7: ALT 26.7% vs. 15.6%; AST 16.9% vs. 8.6%. By day 14 of this 28-day study, the frequency of transaminase elevations equalized across the 2 groups.

In this and other studies in which transaminase elevations occurred, they were noted to persist for up to 4 weeks following treatment with MALANIL for malaria. None were associated with untoward clinical events.

Among subjects who received MALANIL for prophylaxis of malaria in placebo-controlled trials, adverse experiences occurred in similar proportions of subjects receiving MALANIL or placebo (Table 3). The most commonly reported adverse experiences possibly attributable to MALANIL or placebo were headache and abdominal pain. Prophylaxis with MALANIL was discontinued prematurely due to a treatment-related adverse experience in 3 of 381 adults and 0 of 125 pediatric patients.

Table 3. Adverse Experiences in Placebo-Controlled Clinical Trials of MALANIL for Prophylaxis of Malaria

	Percent of Subjects With Adverse Experiences (Percent of Subjects With Adverse Experiences Attributable to Therapy)									
	Adults						Children and Adolescents			
	Placebo		MALANIL*		MALANIL†		Placebo		MALANIL	
Adverse Experience	n = 206		n = 206		n = 381		n = 140		n = 125	
Headache	27	(7)	22	(3)	17	(5)	21	(14)	19	(14)
Fever	13	(1)	5	(0)	3	(0)	11	(<1)	6	(0)
Myalgia	11	(0)	12	(0)	7	(0)	0	(0)	0	(0)
Abdominal pain	10	(5)	9	(4)	6	(3)	29	(29)	33	(31)
Cough	8	(<1)	6	(<1)	4	(1)	9	(0)	9	(0)
Diarrhea	8	(3)	6	(2)	4	(1)	3	(1)	2	(0)
Upper respiratory infection	7	(0)	8	(0)	5	(0)	0	(0)	<1	(0)
Dyspepsia	5	(4)	3	(2)	2	(1)	0	(0)	0	(0)
Back pain	4	(0)	8	(0)	4	(0)	0	(0)	0	(0)
Gastritis	3	(2)	3	(3)	2	(2)	0	(0)	0	(0)
Vomiting	2	(<1)	1	(<1)	<1	(<1)	6	(6)	7	(7)
Flu syndrome	1	(0)	2	(0)	4	(0)	6	(0)	9	(0)
Any adverse experience	65	(32)	54	(17)	49	(17)	62	(41)	60	(42)

* Subjects receiving the recommended dose of atovaquone and proguanil hydrochloride in placebo-controlled trials.

† Subjects receiving the recommended dose of atovaquone and proguanil hydrochloride in any trial.

In an additional placebo-controlled study of malaria prophylaxis with MALANIL involving 330 pediatric patients in a malaria-endemic area (see CLINICAL STUDIES), the safety profile of MALANIL was consistent with that described above. The most common treatment-emergent adverse events with MALANIL were abdominal pain (13%), headache (13%), and cough (10%). Abdominal pain (13% vs. 8%) and vomiting (5% vs. 3%) were reported more often with MALANIL than with placebo, while fever (5% vs. 12%) and diarrhea (1% vs. 5%) were more common with placebo. No patient withdrew from the study due to an adverse experience with MALANIL. No routine laboratory data were obtained during this study.

Among subjects who received MALANIL for prophylaxis of malaria in clinical trials with an active comparator, adverse experiences occurred in a similar or lower proportion of subjects receiving MALANIL than an active comparator (Table 4). The mean durations of dosing and the periods for which the adverse experiences are summarized in Table 4, were 28 days (Study 1) and 26 days (Study 2) for MALANIL, 53 days for mefloquine, and 49 days for chloroquine plus proguanil (reflecting the different recommended dosing regimens). Fewer neuropsychiatric adverse experiences occurred in subjects who received MALANIL than mefloquine. Fewer gastrointestinal adverse experiences occurred in subjects receiving MALANIL than chloroquine/proguanil. Compared with active comparator drugs, subjects receiving MALANIL had fewer adverse experiences overall that were attributed to prophylactic therapy (Table 4). Prophylaxis with MALANIL was discontinued prematurely due to a treatment-related adverse experience in 7 of 1,004 travelers.

Table 4. Adverse Experiences in Active-Controlled Clinical Trials of MALANIL for Prophylaxis of Malaria

	Percent of Subjects With Adverse Experiences* (Percent of Subjects With Adverse Experiences Attributable to Therapy)							
	Study 1				Study 2			
Adverse Experience	MALANIL n = 493		Mefloquine n = 483		MALANIL n = 511		Chloroquine plus Proguanil n = 511	
Diarrhea	38	(8)	36	(7)	34	(5)	39	(7)
Nausea	14	(3)	20	(8)	11	(2)	18	(7)
Abdominal pain	17	(5)	16	(5)	14	(3)	22	(6)
Headache	12	(4)	17	(7)	12	(4)	14	(4)
Dreams	7	(7)	16	(14)	6	(4)	7	(3)
Insomnia	5	(3)	16	(13)	4	(2)	5	(2)
Fever	9	(<1)	11	(1)	8	(<1)	8	(<1)
Dizziness	5	(2)	14	(9)	7	(3)	8	(4)
Vomiting	8	(1)	10	(2)	8	(0)	14	(2)
Oral ulcers	9	(6)	6	(4)	5	(4)	7	(5)
Pruritus	4	(2)	5	(2)	3	(1)	2	(<1)
Visual difficulties	2	(2)	5	(3)	3	(2)	3	(2)
Depression	<1	(<1)	5	(4)	<1	(<1)	1	(<1)
Anxiety	1	(<1)	5	(4)	<1	(<1)	1	(<1)
Any adverse experience	64	(30)	69	(42)	58	(22)	66	(28)
Any neuropsychiatric event	20	(14)	37	(29)	16	(10)	20	(10)
Any GI event	49	(16)	50	(19)	43	(12)	54	(20)

* Adverse experiences that started while receiving active study drug.

In a third active-controlled study, MALANIL (n = 110) was compared with chloroquine/proguanil (n = 111) for the prophylaxis of malaria in 221 non-immune pediatric patients (see CLINICAL STUDIES). The mean duration of exposure was 23 days for MALANIL, 46 days for chloroquine, and 43 days for proguanil, reflecting the different recommended dosage regimens for these products. Fewer patients treated with MALANIL reported abdominal pain (2% vs. 7%) or nausea (<1% vs. 7%) than children who received chloroquine/proguanil. Oral ulceration (2% vs. 2%), vivid dreams (2% vs. <1%), and blurred vision (0% vs. 2%) occurred in similar proportions of patients receiving either MALANIL or chloroquine/proguanil, respectively. Two patients discontinued prophylaxis with chloroquine/proguanil due to adverse events, while none of those receiving MALANIL discontinued due to adverse events.

Post-Marketing Adverse Reactions

In addition to adverse events reported from clinical trials, the following events have been identified during world-wide post-approval use of MALANIL. Because they are reported voluntarily from a population of unknown size, estimates of frequency cannot be made. These events have been chosen for inclusion due to a combination of their seriousness, frequency of reporting, or potential causal connection to MALANIL.

Blood and Lymphatic System Disorders

Neutropenia and rarely anemia. Pancytopenia in patients with severe renal impairment treated with proguanil.

Immune System Disorders

Allergic reactions including angioedema, urticaria, and rare cases of anaphylaxis and vasculitis.

Nervous System Disorders

Rare cases of seizures and psychotic events (such as hallucinations); however, a causal relationship has not been established.

Gastrointestinal Disorders

Stomatitis.

Hepatobiliary Disorders

Elevated liver function tests and rare cases of hepatitis, cholestasis; a single case of hepatic failure requiring transplant has been reported.

Skin and Subcutaneous Tissue Disorders

Photosensitivity, rash, and rare cases of erythema multiforme and Stevens-Johnson syndrome.

OVERDOSAGE

There is no information on overdoses of MALANIL substantially higher than the doses recommended for treatment.

There is no known antidote for atovaquone, and it is currently unknown if atovaquone is dialyzable. The median lethal dose is higher than the maximum oral dose tested in mice and rats (1,825 mg/kg/day). Overdoses up to 31,500 mg of atovaquone have been reported. In one such patient who also took an unspecified dose of dapsone, methemoglobinemia occurred. Rash has also been reported after overdose.

Overdoses of proguanil hydrochloride as large as 1,500 mg have been followed by complete recovery, and doses as high as 700 mg twice daily have been taken for over 2 weeks without serious toxicity. Adverse experiences occasionally associated with proguanil hydrochloride doses of 100 to 200 mg/day, such as epigastric discomfort and vomiting, would be likely to occur with overdose. There are also reports of reversible hair loss and scaling of the skin on the palms and/or soles, reversible aphthous ulceration, and hematologic side effects.

DOSAGE AND ADMINISTRATION

The daily dose should be taken at the same time each day with food or a milky drink. In the event of vomiting within 1 hour after dosing, a repeat dose should be taken.

Prevention of Malaria

Prophylactic treatment with MALANIL should be started 1 or 2 days before entering a malaria-endemic area and continued daily during the stay and for 7 days after return.

Adults

One MALANIL Tablet (adult strength = 250 mg atovaquone/100 mg proguanil hydrochloride) per day.

Pediatric Patients

The dosage for prevention of malaria in pediatric patients is based upon body weight (Table 5).

Table 5. Dosage for Prevention of Malaria in Pediatric Patients

Weight (kg)	Atovaquone/ Proguanil HCl Total Daily Dose	Dosage Regimen
11-20	62.5 mg/25 mg	1 MALANIL Pediatric Tablet daily
21-30	125 mg/50 mg	2 MALANIL Pediatric Tablets as a single dose daily

31-40	187.5 mg/75 mg	3 MALANIL Pediatric Tablets as a single dose daily
>40	250 mg/100 mg	1 MALANIL Tablet (adult strength) as a single dose daily

Treatment of Acute Malaria

Adults

Four MALANIL Tablets (adult strength; total daily dose 1 g atovaquone/400 mg proguanil hydrochloride) as a single dose daily for 3 consecutive days.

Pediatric Patients

The dosage for treatment of acute malaria in pediatric patients is based upon body weight (Table 6).

Table 6. Dosage for Treatment of Acute Malaria in Pediatric Patients

Weight (kg)	Atovaquone/ Proguanil HCl Total Daily Dose	Dosage Regimen
5-8	125 mg/50 mg	2 MALANIL Pediatric Tablets daily for 3 consecutive days
9-10	187.5 mg/75 mg	3 MALANIL Pediatric Tablets daily for 3 consecutive days
11-20	250 mg/100 mg	1 MALANIL Tablet (adult strength) daily for 3 consecutive days
21-30	500 mg/200 mg	2 MALANIL Tablets (adult strength) as a single dose daily for 3 consecutive days
31-40	750 mg/300 mg	3 MALANIL Tablets (adult strength) as a single dose daily for 3 consecutive days
>40	1 g/400 mg	4 MALANIL Tablets (adult strength) as a single dose daily for 3 consecutive days

MALANIL Tablets may be crushed and mixed with condensed milk just prior to administration for children who may have difficulty swallowing tablets.

Patients With Renal Impairment

MALANIL should not be used for malaria prophylaxis in patients with severe renal impairment (creatinine clearance <30 mL/min). MALANIL may be used with caution for the treatment of malaria in patients with severe renal impairment (creatinine clearance <30 mL/min), only if the

benefits of the 3-day treatment regimen outweigh the potential risks associated with increased drug exposure (see CLINICAL PHARMACOLOGY: Special Populations: Renal Impairment). No dosage adjustments are needed in patients with mild (creatinine clearance 50 to 80 mL/min) and moderate (creatinine clearance 30 to 50 mL/min) renal impairment (see CLINICAL PHARMACOLOGY: Special Populations).

Patients With Hepatic Impairment

No dosage adjustments are needed in patients with mild to moderate hepatic impairment. No studies have been conducted in patients with severe hepatic impairment (see CLINICAL PHARMACOLOGY: Special Populations: Hepatic Impairment).

HOW SUPPLIED

MALANIL Tablets, containing 250 mg atovaquone and 100 mg proguanil hydrochloride, are pink, film-coated, round, biconvex tablets engraved with “GX CM3” on one side.

Bottle of 100 tablets with child-resistant closure (NDC 0173-0675-01).

Unit Dose Pack of 24 (NDC 0173-0675-02).

MALANIL Pediatric Tablets, containing 62.5 mg atovaquone and 25 mg proguanil hydrochloride, are pink, film-coated, round, biconvex tablets engraved with “GX CG7” on one side.

Bottle of 100 tablets with child-resistant closure (NDC 0173-0676-01).

Store at 25°C (77°F); excursions permitted to 15° to 30°C (59° to 86°F) (see USP Controlled Room Temperature).

ANIMAL TOXICOLOGY

Fibrovascular proliferation in the right atrium, pyelonephritis, bone marrow hypocellularity, lymphoid atrophy, and gastritis/enteritis were observed in dogs treated with proguanil hydrochloride for 6 months at a dose of 12 mg/kg/day (approximately 3.9 times the recommended daily human dose for malaria prophylaxis on a mg/m2 basis). Bile duct hyperplasia, gall bladder mucosal atrophy, and interstitial pneumonia were observed in dogs treated with proguanil hydrochloride for 6 months at a dose of 4 mg/kg/day (approximately 1.3 times the recommended daily human dose for malaria prophylaxis on a mg/m2 basis). Mucosal hyperplasia of the cecum and renal tubular basophilia were observed in rats treated with proguanil hydrochloride for 6 months at a dose of 20 mg/kg/day (approximately 1.6 times the recommended daily human dose for malaria prophylaxis on a mg/m2 basis). Adverse heart, lung, liver, and gall bladder effects observed in dogs and kidney effects observed in rats were not shown to be reversible.

CLINICAL STUDIES

Treatment of Acute Malarial Infections

In 3 phase II clinical trials, atovaquone alone, proguanil hydrochloride alone, and the combination of atovaquone and proguanil hydrochloride were evaluated for the treatment of acute, uncomplicated malaria caused by P. falciparum. Among 156 evaluable patients, the

parasitological cure rate was 59/89 (66%) with atovaquone alone, 1/17 (6%) with proguanil hydrochloride alone, and 50/50 (100%) with the combination of atovaquone and proguanil hydrochloride.

MALANIL was evaluated for treatment of acute, uncomplicated malaria caused by P. falciparum in 8 phase III controlled clinical trials. Among 471 evaluable patients treated with the equivalent of 4 MALANIL Tablets once daily for 3 days, 464 had a sensitive response (elimination of parasitemia with no recurrent parasitemia during follow-up for 28 days) (see Table 7). Seven patients had a response of RI resistance (elimination of parasitemia but with recurrent parasitemia between 7 and 28 days after starting treatment). In these trials, the response to treatment with MALANIL was similar to treatment with the comparator drug in 4 trials, and better than the response to treatment with the comparator drug in the other 4 trials.

The overall efficacy in 521 evaluable patients was 98.7% (Table 7).

Table 7. Parasitological Response in Clinical Trials of MALANIL for Treatment of P. falciparum Malaria

Study Site	MALANIL*		Comparator		
	Evaluable Patients (n)	% Sensitive Response†	Drug(s)	Evaluable Patients (n)	% Sensitive Response†
Brazil	74	98.6%	Quinine and tetracycline	76	100.0%
Thailand	79	100.0%	Mefloquine	79	86.1%
France‡	21	100.0%	Halofantrine	18	100.0%
Kenya‡,§	81	93.8%	Halofantrine	83	90.4%
Zambia	80	100.0%	Pyrimethamine/ sulfadoxine (P/S)	80	98.8%
Gabon‡	63	98.4%	Amodiaquine	63	81.0%
Philippines	54	100.0%	Chloroquine (Cq)	23	30.4%
			Cq and P/S	32	87.5%
Peru	19	100.0%	Chloroquine	13	7.7%
			P/S	7	100.0%

* MALANIL = 1,000 mg atovaquone and 400 mg proguanil hydrochloride (or equivalent based on body weight for patients weighing ≤40 kg) once daily for 3 days.

† Elimination of parasitemia with no recurrent parasitemia during follow-up for 28 days.

‡ Patients hospitalized only for acute care. Follow-up conducted in outpatients.

§ Study in pediatric patients 3 to 12 years of age.

Eighteen of 521 (3.5%) evaluable patients with acute falciparum malaria presented with a pretreatment serum creatinine greater than 2.0 mg/dL (range 2.1 to 4.3 mg/dL). All were successfully treated with MALANIL and 17 of 18 (94.4%) had normal serum creatinine levels by day 7.

Data from a phase II trial of atovaquone conducted in Zambia suggested that approximately 40% of the study population in this country were HIV-infected patients. The enrollment criteria were similar for the phase III trial of MALANIL conducted in Zambia and the results are presented in Table 7. Efficacy rates for MALANIL in this study population were high and comparable to other populations studied.

The efficacy of MALANIL in the treatment of the erythrocytic phase of nonfalciparum malaria was assessed in a small number of patients. Of the 23 patients in Thailand infected with P. vivax and treated with atovaquone/proguanil hydrochloride 1,000 mg/400 mg daily for 3 days, parasitemia cleared in 21 (91.3%) at 7 days. Parasite relapse occurred commonly when P. vivax malaria was treated with MALANIL alone. Seven patients in Gabon with malaria due to P. ovale or P. malariae were treated with atovaquone/proguanil hydrochloride 1,000 mg/400 mg daily for 3 days. All 6 evaluable patients (3 with P. malariae, 2 with P. ovale, and 1 with mixed P. falciparum and P. ovale) were cured at 28 days. Relapsing malarias including P. vivax and P. ovale require additional treatment to prevent relapse.

The efficacy of MALANIL in treating acute uncomplicated P. falciparum malaria in children weighing ≥5 and <11 kg was examined in an open-label, randomized trial conducted in Gabon. Patients received either MALANIL (2 or 3 MALANIL Pediatric Tablets once daily depending upon body weight) for 3 days (n = 100) or amodiaquine (10 mg/kg/day) for 3 days (n = 100). In this study, the MALANIL Tablets were crushed and mixed with condensed milk just prior to administration. In the per-protocol population, adequate clinical response was obtained in 95% (87/92) of the pediatric patients who received MALANIL and in 53% (41/78) of those who received amodiaquine. A response of RI resistance (elimination of parasitemia but with recurrent parasitemia between 7 and 28 days after starting treatment) was noted in 3% and 40% of the patients, respectively. Two cases of RIII resistance (rising parasite count despite therapy) were reported in the patients receiving MALANIL. There were 4 cases of RIII in the amodiaquine arm.

Prevention of Malaria

MALANIL was evaluated for prophylaxis of malaria in 5 clinical trials in malaria-endemic areas and in 3 active-controlled trials in non-immune travelers to malaria-endemic areas.

Three placebo-controlled studies of 10 to 12 weeks' duration were conducted among residents of malaria-endemic areas in Kenya, Zambia, and Gabon. Of a total of 669 randomized patients (including 264 pediatric patients 5 to 16 years of age), 103 were withdrawn for reasons other than falciparum malaria or drug-related adverse events. (Fifty-five percent of these were lost to follow-up and 45% were withdrawn for protocol violations.) The results are listed in Table 8.

Table 8. Prevention of Parasitemia in Placebo-Controlled Clinical Trials of MALANIL for Prophylaxis of P. falciparum Malaria in Residents of Malaria-Endemic Areas

Total number of patients randomized	MALANIL	Placebo
	326	341
Failed to complete study	57	44
Developed parasitemia (P. falciparum)	2	92

In another study, 330 Gabonese pediatric patients (weighing 13 to 40 kg, and aged 4 to 14 years) who had received successful open-label radical cure treatment with artesunate, were randomized to receive either MALANIL (dosage based on body weight) or placebo in a double-blind fashion for 12 weeks. Blood smears were obtained weekly and any time malaria was suspected. Nineteen of the 165 children given MALANIL and 18 of 165 patients given placebo withdrew from the study for reasons other than parasitemia (primary reason was lost to follow-up). In the per-protocol population, 1 out of 150 patients (<1%) who received MALANIL developed P. falciparum parasitemia while receiving prophylaxis with MALANIL compared with 31 (22%) of the 144 placebo recipients.

In a 10-week study in 175 South African subjects who moved into malaria-endemic areas and were given prophylaxis with 1 MALANIL Tablet daily, parasitemia developed in 1 subject who missed several doses of medication. Since no placebo control was included, the incidence of malaria in this study was not known.

Two active-controlled studies were conducted in non-immune travelers who visited a malaria-endemic area. The mean duration of travel was 18 days (range 2 to 38 days). Of a total of 1,998 randomized patients who received MALANIL or controlled drug, 24 discontinued from the study before follow-up evaluation 60 days after leaving the endemic area. Nine of these were lost to follow-up, 2 withdrew because of an adverse experience, and 13 were discontinued for other reasons. These studies were not large enough to allow for statements of comparative efficacy. In addition, the true exposure rate to P. falciparum malaria in both studies is unknown. The results are listed in Table 9.

Table 9. Prevention of Parasitemia in Active-Controlled Clinical Trials of MALANIL for Prophylaxis of P. falciparum Malaria in Non-Immune Travelers

Total number of randomized patients who received study drug	MALANIL	Mefloquine	Chloroquine plus Proguanil
	1,004	483	511
Failed to complete study	14	6	4
Developed parasitemia (P. falciparum)	0	0	3

A third randomized, open-label study was conducted which included 221 otherwise healthy pediatric patients (weighing ≥11 kg and 2 to 17 years of age) who were at risk of contracting malaria by traveling to an endemic area. The mean duration of travel was 15 days (range 1 to 30 days). Prophylaxis with MALANIL (n = 110, dosage based on body weight) began 1 or 2 days before entering the endemic area and lasted until 7 days after leaving the area. A control group (n = 111) received prophylaxis with chloroquine/proguanil dosed according to WHO guidelines. No cases of malaria occurred in either group of children. However, the study was not large enough to allow for statements of comparative efficacy. In addition, the true exposure rate to P.falciparum malaria in this study is unknown.

In a malaria challenge study conducted in healthy US volunteers, atovaquone alone prevented malaria in 6 of 6 individuals, whereas 4 of 4 placebo-treated volunteers developed malaria.

Causal Prophylaxis

In separate studies with small numbers of volunteers, atovaquone and proguanil hydrochloride were independently shown to have causal prophylactic activity directed against liver-stage parasites of P. falciparum. Six patients given a single dose of atovaquone 250 mg 24 hours prior to malaria challenge were protected from developing malaria, whereas all 4 placebo-treated patients developed malaria.

During the 4 weeks following cessation of prophylaxis in clinical trial participants who remained in malaria-endemic areas and were available for evaluation, malaria developed in 24 of 211 (11.4%) subjects who took placebo and 9 of 328 (2.7%) who took MALANIL. While new infections could not be distinguished from recrudescent infections, all but 1 of the infections in patients treated with MALANIL occurred more than 15 days after stopping therapy, probably representing new infections. The single case occurring on day 8 following cessation of therapy with MALANIL probably represents a failure of prophylaxis with MALANIL.

The possibility that delayed cases of P. falciparum malaria may occur some time after stopping prophylaxis with MALANIL cannot be ruled out. Hence, returning travelers developing febrile illnesses should be investigated for malaria.

GlaxoSmithKline

Research Triangle Park, NC 27709

©2008, GlaxoSmithKline. All rights reserved.

June 2008 MLR:3PI

Principal Display Panel

NDC 0173-0675-01

MALANIL®

(atovaquone and proguanil HCl)

Tablets

Each tablet contains 250 mg atovaquone and 100 mg proguanil HCl.

Rx only

100 Tablets

See package insert for Dosage and Administration.

Store at 25oC (77oF); excursions permitted to 15o to 30oC (59oto 86oF) (see USP Controlled Room Temperature).

Do not use if printed safety seal under cap is broken or missing.

GlaxoSmithKline

Research Triangle Park, NC 27709

Made in Canada

4156005

A001970 Rev. 8/03

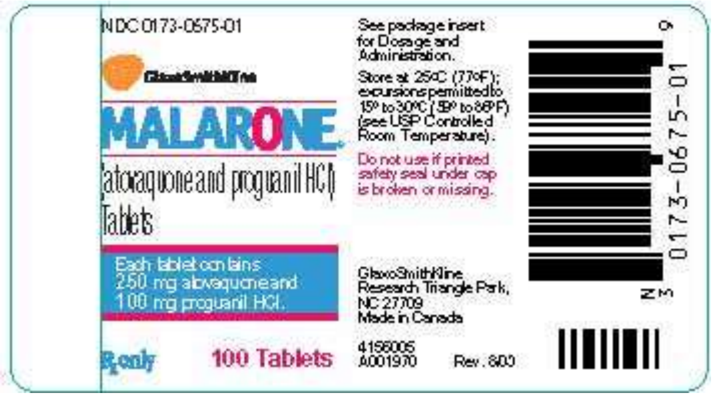

NDC 0173-0676-01

MALANIL®

(atovaquone and proguanil HCl)

Pediatric Tablets

Each tablet contains 62.5 mg atovaquone and 25 mg proguanil HCl.

Rx only

100 Tablets

See prescribing information for Dosage and Administration.

Store at 25oC (77oF); excursions permitted to 15o to 30oC (59oto 86oF) (see USP Controlled Room Temperature).

Do not use if printed safety seal under cap is broken or missing.

GlaxoSmithKline

RTP, NC 27709

Made in Canada

4159187

A002538 Rev. 1/04

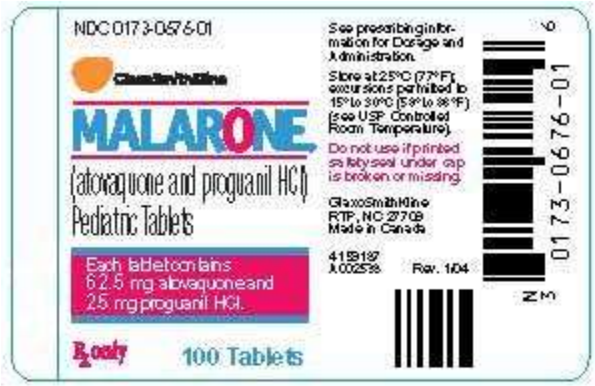

MALANIL

atovaquone and proguanil hydrochloride   tablet, film coated

Product Information			
Product Type	HUMAN PRESCRIPTION DRUG	NDC Product Code (Source)	0173-0675
Route of Administration	ORAL	DEA Schedule	

Active Ingredient/Active Moiety

Ingredient Name	Basis of Strength	Strength
**ATOVAQUONE** (ATOVAQUONE)	ATOVAQUONE	250 mg
**PROGUANIL HYDROCHLORIDE** (PROGUANIL)	PROGUANIL HYDROCHLORIDE	100 mg

Inactive Ingredients

Ingredient Name	Strength
**HYDROXYPROPYL CELLULOSE, LOW SUBSTITUTED**	
**MAGNESIUM STEARATE**	
**CELLULOSE, MICROCRYSTALLINE**	
**POLOXAMER 188**	
**POVIDONE K30**	
**SODIUM STARCH GLYCOLATE TYPE A POTATO**	
**HYPROMELLOSE**	
**POLYETHYLENE GLYCOL 400**	
**POLYETHYLENE GLYCOL 8000**	
**FERRIC OXIDE RED**	
**TITANIUM DIOXIDE**	

Product Characteristics

Color	PINK	Score	no score
Shape	ROUND	Size	11mm

Flavor		Imprint Code	GX;CM3
Contains			

Packaging

#	NDC	Package Description	Multilevel Packaging
**1**	0173-0675-02	24 TABLET In 1 DOSE PACK	None
**2**	0173-0675-01	100 TABLET In 1 BOTTLE	None

Marketing Information

Marketing Category	Application Number or Monograph Citation	Marketing Start Date	Marketing End Date
NDA	NDA021078	07/26/2000	

MALANIL
atovaquone and proguanil hydrochloride tablet, film coated

Product Information

Product Type	HUMAN PRESCRIPTION DRUG	NDC Product Code (Source)	0173-0676
Route of Administration	ORAL	DEA Schedule	

Active Ingredient/Active Moiety

Ingredient Name	Basis of Strength	Strength
**ATOVAQUONE** (ATOVAQUONE)	ATOVAQUONE	62.5 mg
**PROGUANIL HYDROCHLORIDE** (PROGUANIL)	PROGUANIL HYDROCHLORIDE	25 mg

Inactive Ingredients

Ingredient Name	Strength
**HYDROXYPROPYL CELLULOSE, LOW SUBSTITUTED**	
**MAGNESIUM STEARATE**	
**CELLULOSE, MICROCRYSTALLINE**	
**POLOXAMER 188**	
**POVIDONE K30**	
**SODIUM STARCH GLYCOLATE TYPE A POTATO**	
**HYPROMELLOSE**	
**POLYETHYLENE GLYCOL 400**	
**POLYETHYLENE GLYCOL 8000**	
**FERRIC OXIDE RED**	
**TITANIUM DIOXIDE**	

Product Characteristics

Color	PINK	Score	no score
Shape	ROUND	Size	7mm
Flavor		Imprint Code	GX;CG7
Contains			

Packaging

#	NDC	Package Description	Multilevel Packaging
**1**	0173-0676-01	100 TABLET In 1 BOTTLE	None

Marketing Information

Marketing Category	Application Number or Monograph Citation	Marketing Start Date	Marketing End Date
NDA	NDA021078	07/26/2000	

Labeler - SmithKline Beecham Corporation (167380711)
Revised: 06/2008SmithKline Beecham Corporation

APPENDIX 7 Cytoadherence assay

P. falciparum-infected blood samples will be cultured *in vitro* (Trager W and Jensen JB, 1976) until develop to trophozoite stage and then use for the adhesion assay. The Sevuparin/DF02 will be added to the red cell suspension form parasite culture at various concentrations, then incubated for 1, 2, 4, 6 hours. After incubation period, cell suspension will be harvested and centrifuged at 2,500 rpm for 5 minutes. Only the pellet will be used for the adhesion assay. Adhesion assay will performed static and flow based condition as described previously (Udomsangpetch R et al, 1996; Yipp BG *et a*l., 2000). Briefly, IRBC - endothelial cell interactions at static and fluid shear stresses will be studied by using a parallel plate flow chamber. A suspension of IRBCs (1% hematocrit, 5% parasitemia in RPMI 1640, pH 7.2) was drawn through the flow chamber at varying rates with an infusion pump attached to the outlet. At this concentration of IRBCs, attached IRBCs and their motion could be clearly observed with phase contrast objectives and quantitated by analysis of videotaped images. Tethering referred to the initial contact between IRBCs and the endothelial monolayer. An IRBC was considered adherent if it remained stationary for more than 10 seconds, and the results will be expressed as the number of adherent IRBCs per square millimetre of surface area.

Trager W, Jensen JB. Human malaria parasites in continuous culture. Science.1976 Aug 20; 193(4254):673-5.

Udomsangpetch R, Pipitaporn B, Krishna S, Angus B, Pukrittayakamee S, Bates I,Suputtamongkol Y, Kyle DE, White NJ. Antimalarial drugs reduce cytoadherence and rosetting Plasmodium falciparum. J Infect Dis. 1996 Mar; 173(3):691-8.

Yipp BG, Anand S, Schollaardt T, Patel KD, Looareesuwan S, Ho M. Synergism of multiple adhesion molecules in mediating cytoadherence of Plasmodium falciparum-infected erythrocytes to microvascular endothelial cells under flow. Blood. 2000 Sep 15; 96(6):2292-8.

APPENDIX 8 Rosetting formation assay

Blood sample will be collected in defibrinated non-heparinised tubes and centrifuged at 800 g for 5 min. Buffy coat and plasma will be removed, and packed RBCs are washed 3 times them resuspended in a standard malaria culture medium of RPMI-1640 supplemented with 25 mmol/L HEPES, 10 % human AB serum, and 40% µg/ml gentamicin, to achieve a final concentration of 5% hematocrit. After up to 30 hrs of cultivation at 37°C in 5% CO_2, thin films will be prepared, and the stage of parasite maturation will be examined by microscopy. Ten microlitres of culture will be dropped on a glass slide. Mix with a drop of ethidium bromide (0.2 mg/mL). Put on a coverslip. Analyze slide immediately under microscope (100 x objective lens). Count 100 infected red cells to estimate the rosetting rate. The number of IRBCs forming rosettes ($\geq$ 2 uninfected RBCs adherent to one IRBCs) are expressed as the number of IRBCs that formed rosettes in a total of 100 IRBCs.

Chotivanich KT, Dondorp AM, White NJ, Peters K, Vreeken J, Kager PA,Udomsangpetch R. The resistance to physiological shear stresses of the erythrocytic rosettes formed by cells infected with Plasmodium falciparum. *Ann Trop Med Parasitol*. 2000 Apr; 94(3):219-26.

Comments

- Infected erythrocytes (except ring forms) within a rosette and infected erythrocytes not connected to a rosette are scored separately.
- Infected erythrocytes that have bound two or more non-infected cells are scored as rosettes. If the rosette contains more than one infected erythrocyte, the cells are scored separately.
- Ring forms within a rosette are not counted.
- Late-stage (trophozoite and schizont, but not ring forms) infected erythrocytes are scored and added to the total number of late infected erythrocytes.
- Since it can be hard to tell the difference between a ring form and an early trophozoite, only parasites with a diameter that covers at least one-third of the diameter of the erythrocyte are counted.
- The number of infected erythrocytes in large rosettes (where it can be difficult to determine the exact number of infected cells) are counted as 3.
- If it is difficult to determine the number of cells (usually 2 or 3) bound to an infected erythrocyte, the cover slip can be pressed lightly (using a pencil) to induce a slight movement. This is important, because quite often it is found that only 1 erythrocyte is actually bound to the infected cell, when others that may appear to be are not.

Reference: MR4 / ATCC Manassas, Virginia 2008 METHODS IN MALARIA RESEARCH Fifth Edition; Assay III: A. Acridine orange (AO) vital stain of cultures, and VI: M. Reversion of rosettes.

APPENDIX 9 CTCAE V. 4.0

Common Terminology Criteria for Adverse Events v4.0 (CTCAE)

Publish Date: May 28, 2009

Quick Reference

The NCI Common Terminology Criteria for Adverse Events is a descriptive terminology which can be utilized for Adverse Event (AE) reporting. A grading (severity) scale is provided for each AE term.

Components and Organization

SOC

System Organ Class, the highest level of the MedDRA hierarchy, is identified by anatomical or physiological system, etiology, or purpose (e.g., SOC Investigations for laboratory test results). CTCAE terms are grouped by MedDRA Primary SOCs. Within each SOC, AEs are listed and accompanied by descriptions of severity (Grade).

CTCAE Terms

An Adverse Event (AE) is any unfavorable and unintended sign (including an abnormal laboratory finding), symptom, or disease temporally associated with the use of a medical treatment or procedure that may or may *not* be considered related to the medical treatment or procedure. An AE is a term that is a unique representation of a specific event used for medical documentation and scientific analyses. Each CTCAE v4.0 term is a MedDRA LLT (Lowest Level Term).

Definitions

A brief definition is provided to clarify the meaning of each AE term.

Grades

Grade refers to the severity of the AE. The CTCAE displays Grades 1 through 5 with unique clinical descriptions of severity for each AE based on this general guideline:

Grade 1	Mild; asymptomatic or mild symptoms; clinical or diagnostic observations only; intervention not indicated.
Grade 2	Moderate; minimal, local or noninvasive intervention indicated; limiting age-appropriate instrumental ADL*.
Grade 3	Severe or medically significant but not immediately life-threatening; hospitalization or prolongation of hospitalization indicated; disabling; limiting self care ADL**.
Grade 4	Life-threatening consequences; urgent intervention indicated.
Grade 5	Death related to AE.

A Semi-colon indicates 'or' within the description of the grade.

A single dash (-) indicates a grade is not available.

Not all Grades are appropriate for all AEs. Therefore, some AEs are listed with fewer than five options for Grade selection.

Grade 5

Grade 5 (Death) is not appropriate for some AEs and therefore is not an option.

Activities of Daily Living (ADL)

*Instrumental ADL refer to preparing meals, shopping for groceries or clothes, using the telephone, managing money, etc.

**Self care ADL refer to bathing, dressing and undressing, feeding self, using the toilet, taking medications, and not bedridden.

† CTCAE v4.0 incorporates certain elements of the MedDRA terminology. For further details on MedDRA refer to the MedDRA MSSO Web site (http://www.meddramsso.com).

APPENDIX 10 Blood and urine volumes

1. Blood and urine volumes for part 1 study

Day	Investigations	Volume (ml)
**Screening investigation**	• Blood for Haematology/ Complete blood count and blood group	2 ml
	• Parasite count (thick and thin blood films)	0.5 ml
	• Blood for Biochemistry (Glucose, BUN, Creatinine, AST,ALT,ALP,total bilirubin, electrolyte (Na,K,tCO_2),calcium, LDH,CPK	7 ml
	• aPTT, PT and antiXa	1 ml
	• Urine pregnancy test and urine analysis	6 ml
	**Total blood volume**	**10.5 ml**<br>**urine 6 ml**
**<u>Day 1</u>**<br>First treament day	• Blood for Haematology/ Complete blood count **(use the result from screening)**	-
	• Blood for Biochemistry (Glucose, BUN, Creatinine, AST,ALT,ALP,total bilirubin, electrolyte (Na,K,tCO_2),calcium, LDH,CPK **(use the result from screening)**	-
	• Cytoadherance and Rosette assay (pre-dose)	7 ml.
	• Parasite count (Thick and thin blood film) and heamatocrite at 0, 30, 60, 90 min, 2, 3, 4, 6, 8, 10, 12, 18, 24 hrs	0.5 ml.x13<br>=6.5 ml
	• aPTT, PT and antiXa at 1,2,5,11,17,23 hrs	1 ml*6<br>=6 ml
	• PK blood sample at 0, 6, 15, 30, 45min, 1, 1.5, 2, 3, 4, 5 hrs. Acceptable time windows:	3.5 ml.x11<br>=38.5 ml.
	**Total blood volume**	**58 ml**
**<u>Day 2</u>**<br>24 hrs after treatment	• Parasite count(Thick and thin blood film) and heamatocrite every 6 hrs until parasite negative 2 times on the peripheral blood	0.5 ml.x4<br>= 2 ml.
	• Blood for Haematology/ Complete blood count	2 ml
	• Blood for Biochemistry (Glucose, BUN, Creatinine, AST,ALT,ALP,total bilirubin, electrolyte (Na,K,tCO_2),calcium, LDH,CPK	7 ml
	• aPTT, PT and antiXa at 30,31,32,35,41,47 hrs.	1 ml*6<br>=6 ml
	**Total blood volume**	**17 ml**
**<u>Day 3</u>**<br>48 hrs after treatment	• Parasite count(Thick and thin blood film) and heamatocrite every 6 hrs until parasite negative 2 times on the peripheral blood	0.5 ml.x4<br>= 2 ml.
	• Blood for Haematology/ Complete blood count	2 ml
	• Blood for Biochemistry (Glucose, BUN, Creatinine, AST,ALT,ALP,total bilirubin, electrolyte (Na,K,tCO_2),calcium, LDH,CPK	7 ml
	• aPTT , PT and antiXa at 53,56,59,65,65:45,67,68,71 hrs (± 5 minutes for all time points).	1 ml*8<br>=8 ml
	• PK blood sample at 65:45, 66,66:15,66:30,66:45,67,67:30,68,69,70,71 hrs	3.5 ml.x11<br>=38.5 ml.
	**Total blood volume**	**57.5 ml**
**<u>Day 4</u>**<br>72 hrs after treatment	• Parasite count(Thick and thin blood film) and heamatocrite every 6 hrs until parasite negative 2 times on the peripheral blood	0.5 ml.x4<br>= 2 ml.
	**Total blood volume on day 4**	**2 ml.**
**Day 7 <u>+</u>1**	• Blood for Haematology/ Complete blood count, Parasite count(Thick and thin blood film) and heamatocrite	2 ml.
	• Blood for Biochemistry (Glucose, BUN, Creatinine, AST,ALT,ALP,total bilirubin, electrolyte (Na,K,tCO_2),calcium and aPTT	7 ml.
	**Total blood volume on follow up day(Day 7<u>+</u>1)**	**9 ml**
**Day 14 <u>+</u>1**	• Blood for Complete blood count, Parasite count(Thick and thin blood film) and haematocrite	2 ml.
	• Blood for Biochemistry (Glucose, BUN, Creatinine, AST,ALT, ALP,total bilirubin, electrolyte (Na,K,tCO_2),calcium and aPTT	7 ml.
	• Urine pregnancy test	1 ml
	**Total blood volume on follow up day(Day 14<u>+</u>1)**	**9ml**<br>**Urine 1 ml**

Total blood volume is 163.0 ml (11 table spoonfuls) and 7 ml urine

2. Blood and urine volume for part 2 study who randomized to receive Sevuparin/ DF02 (10 first patients for PK analysis)

Day	Investigations	Volume (ml)
**Screening investigation**	• Blood for Haematology/ Complete blood count and blood group	3 ml
	• Parasite count (thick and thin blood films)	0.5 ml
	• Blood for Biochemistry (Glucose, BUN, Creatinine, AST,ALT,ALP,total bilirubin, electrolyte (Na,K,tCO_2),calcium, LDH,CPK	5 ml
	• aPTT, PT and antiXa (± 2 minutes)	1.5 ml
	• Urine pregnancy test and urine analysis	6 ml
	**Total blood volume**	**10 ml**<br>**urine 6 ml**
**Day 1**<br>First treament day	• Blood for Haematology/ Complete blood count (use the result from screening)	-
	• Blood for Biochemistry (Glucose, BUN, Creatinine, AST,ALT,ALP,total bilirubin, electrolyte (Na,K,tCO2),calcium, LDH,CPK (use the result from screening)	-
	• Cytoadherance and Rosette assay (pre-dose)	5 ml
	• Parasite count (Thick and thin blood film) and heamatocrite at 0, 30, 60, 90 min, 2, 3, 4, 6, 8, 10, 12, 18, 24 hrs	0.5 ml.x13<br>=6.5 ml
	• aPTT, PT and antiXa at 2, 5 ,11, 17, 23 hrs (± 10 minutes)	1.5 ml x 5<br>= 7.5 ml
	• PK blood sample at pre-dose, 0 ,6, 15, 30, 45 min, 1, 1.5, 2, 3, 4, 5 hrs (± 2 minutes for time points up to15 min post dose, ± 10 minutes for all time points above 15 min post dose)	3 ml.x 12<br>= 36.0 ml.
	**Total blood volume**	**55 ml**
**Day 2**<br>24 hrs after treatment	• Parasite count(Thick and thin blood film) and heamatocrite every 6 hrs until parasite negative 2 times on the peripheral blood	0.5 ml.x4<br>= 2 ml
	• Blood for Haematology/ Complete blood count	3 ml
	• Blood for Biochemistry (Glucose, BUN, Creatinine, AST,ALT,ALP,total bilirubin, electrolyte (Na,K,tCO_2),calcium, LDH,CPK	5 ml
	• aPTT, PT and antiXa at 29, 32,35,41,47hrs.	1.5 ml x 5<br>=7.5 ml
	**Total blood volume**	**17.5 ml**
**Day 3**<br>48 hrs after treatment	• Parasite count(Thick and thin blood film) and heamatocrite every 6 hrs until parasite negative 2 times on the peripheral blood	0.5 ml.x4<br>= 2 ml
	• Blood for Haematology/ Complete blood count	3 ml
	• Blood for Biochemistry (Glucose, BUN, Creatinine, AST,ALT,ALP,total bilirubin, electrolyte (Na,K,tCO_2),calcium, LDH,CPK	5 ml
	• aPTT , PT and antiXa at 53 ,59 ,65, 68, 71 hrs (± 10 minutes)	1.5 ml x 5<br>=7.5 ml
	• PK blood sample at 65, 66, 66:15, 66:30, 66:45, 67:30, 68 ,69, 70, 71 hrs (± 10 minutes)	3 ml x 10<br>=30 ml
	**Total blood volume**	**47.5 ml**
**Day 4**<br>72 hrs after treatment	• Parasite count(Thick and thin blood film) and heamatocrite every 6 hrs until parasite negative 2 times on the peripheral blood	0.5 ml.x4<br>= 2 ml.
	**Total blood volume on day 4**	**2 ml**
**Day 7 +1**	• Blood for Haematology/ Complete blood count, Parasite count(Thick and thin blood film) and heamatocrite	3 ml
	• Blood for Biochemistry (Glucose, BUN, Creatinine, AST,ALT,ALP,total bilirubin, electrolyte (Na,K,tCO_2),calcium and aPTT	5 m.
	**Total blood volume on follow up day(Day 7+1)**	**8 ml**
**Day 14 +1**	• Blood for Complete blood count, Parasite count(Thick and thin blood film) and haematocrite	3 ml.
	• Blood for Biochemistry (Glucose, BUN, Creatinine, AST,ALT, ALP,total bilirubin, electrolyte (Na,K,tCO_2),calcium and aPTT	5 ml.
	• Urine pregnancy test	1 ml
	**Total blood volume on follow up day(Day 14+1)**	**8 ml**<br>**Urine 1 ml**

Total blood volume is 148 ml (9.9 table spoonfuls) and 7 ml urine

3. Blood and urine volume for part 2 study who randomized to receive Sevuparin/ DF02 with antimalarial drug: Malanil® without PK analysis)

Day	Investigations	Volume (ml)
**Screening investigation**	• Blood for Haematology/ Complete blood count and blood group	3 ml
	• Parasite count (thick and thin blood films)	0.5 ml
	• Blood for Biochemistry (Glucose, BUN, Creatinine, AST,ALT,ALP,total bilirubin, electrolyte (Na,K,tCO_2),calcium, LDH,CPK	5 ml
	• aPTT, PT and antiXa (± 2 minutes)	1.5 ml
	• Urine pregnancy test and urine analysis	6 ml
	**Total blood volume**	**10 ml**<br>**urine 6 ml**
**Day 1**<br>First treament day	• Blood for Haematology/ Complete blood count **(use the result from screening)**	-
	• Blood for Biochemistry (Glucose, BUN, Creatinine, AST,ALT,ALP,total bilirubin, electrolyte (Na,K,tCO_2),calcium, LDH,CPK **(use the result from screening)**	-
	• Cytoadherance and Rosette assay (pre-dose)	5 ml
	• Parasite count (Thick and thin blood film) and heamatocrite at 0, 30, 60, 90 min, 2, 3, 4, 6, 8, 10, 12, 18, 24 hrs	0.5 ml.x13<br>=6.5 ml
	• aPTT, PT and antiXa at 2, 5, 11, 17, 23 hrs (± 10 minutes)	1.5 ml x 5<br>=7.5ml
	**Total blood volume**	**19 ml**
**Day 2**<br>24 hrs after treatment	• Parasite count(Thick and thin blood film) and heamatocrite every 6 hrs until parasite negative 2 times ong the peripheral blood	0.5 ml x 4<br>= 2 ml
	• Blood for Haematology/ Complete blood count	3 ml
	• Blood for Biochemistry (Glucose, BUN, Creatinine, AST,ALT,ALP,total bilirubin, electrolyte (Na,K,tCO2),calcium, LDH,CPK	5 ml
	• aPTT, PT and antiXa at 29, 32,35,41,47 hrs (± 10 minutes)	1.5 ml x 5<br>=7.5 ml
	**Total blood volume**	**17.5 ml**
**Day 3**<br>48 hrs after treatment	• Parasite count(Thick and thin blood film) and heamatocrite every 6 hrs until parasite negative 2 times on the peripheral blood	0.5 ml x 4<br>= 2 ml
	• Blood for Haematology/ Complete blood count	3 ml
	• Blood for Biochemistry (Glucose, BUN, Creatinine, AST,ALT,ALP,total bilirubin, electrolyte (Na,K,tCO2),calcium, LDH,CPK	5 ml
	• aPTT , PT and antiXa at 53,59,65, 68,71 hrs (± 10 minutes)	1.5 ml x 5<br>=7.5 ml
	**Total blood volume**	**17.5ml**
**Day 4**<br>72 hrs after treatment	• Parasite count (Thick and thin blood film) and heamatocrite every 6 hrs until parasite negative 2 times on the peripheral blood	0.5 ml.x4<br>= 2 ml
	**Total blood volume on day 4**	**2 ml**
**Day 7 ±1**	• Blood for Haematology/ Complete blood count, Parasite count(Thick and thin blood film) and heamatocrite	3 ml
	• Blood for Biochemistry (Glucose, BUN, Creatinine, AST,ALT,ALP,total bilirubin, electrolyte (Na,K,tCO_2),calcium and aPTT	5 ml
	**Total blood volume on follow up day(Day 7±1)**	**8 ml**
**Day 14 ±1**	• Blood for Complete blood count, Parasite count(Thick and thin blood film) and haematocrite	3 ml
	• Blood for Biochemistry (Glucose, BUN, Creatinine, AST,ALT, ALP,total bilirubin, electrolyte (Na,K,tCO_2),calcium and aPTT	5 ml
	• Urine pregnancy test	1 ml
	**Total blood volume on follow up day(Day 14±1)**	**8 ml**<br>**Urine 1 ml**

The total blood volume is 82 ml (5.4 table spoonfuls and 7 ml of urine).

4. Blood and urine volume for part 2 study who randomized to receive antimalarial drug: malanil®alone

Day	Investigations	Volume (ml)
**Screening investigation**	• Blood for Haematology/ Complete blood count and blood group	3 ml
	• Parasite count (thick and thin blood films)	0.5 ml
	• Blood for Biochemistry (Glucose, BUN, Creatinine, AST,ALT,ALP,total bilirubin, electrolyte (Na,K,tCO_2),calcium, LDH,CPK	5 ml
	• aPTT, PT and antiXa (± 2 minutes)	1.5 ml
	• Urine pregnancy test and urine analysis	6 ml
	**Total blood volume**	**10 ml**<br>**urine 6 ml**
**Day 1**<br>First treament day	• Blood for Haematology/ Complete blood count **(use the result from screening)**	-
	• Blood for Biochemistry (Glucose, BUN, Creatinine, AST,ALT,ALP,total bilirubin, electrolyte (Na,K,tCO_2),calcium, LDH,CPK **(use the result from screening)**	-
	• Cytoadherance and Rosette assay (pre-dose)	5 ml
	• Parasite count (Thick and thin blood film) and heamatocrite at 0, 30, 60, 90 min, 2, 3, 4, 6, 8, 10, 12, 18, 24 hrs	0.5 ml x 13<br>= 6.5 ml
	**Total blood volume**	**11.5 ml**
**Day 2**<br>24 hrs after treatment	• Parasite count (Thick and thin blood film) and heamatocrite every 6 hrs until parasite negative 2 times ong the peripheral blood	0.5 ml x 4<br>= 2 ml
	• Blood for Haematology/ Complete blood count	3 ml
	• Blood for Biochemistry (Glucose, BUN, Creatinine, AST,ALT,ALP,total bilirubin, electrolyte (Na,K,tCO_2),calcium, LDH,CPK	5 ml
	**Total blood volume**	**10 ml**
**Day 3**<br>48 hrs after treatment	• Parasite count(Thick and thin blood film) and heamatocrite every 6 hrs until parasite negative 2 times on the peripheral blood	0.5 ml x 4<br>= 2 ml
	• Blood for Haematology/ Complete blood count	3 ml
	• Blood for Biochemistry (Glucose, BUN, Creatinine, AST,ALT,ALP,total bilirubin, electrolyte (Na,K,tCO_2),calcium, LDH,CPK	5 ml
	**Total blood volume**	**10 ml**
**Day 4**<br>72 hrs after treatment	• Parasite count(Thick and thin blood film) and heamatocrite every 6 hrs until parasite negative 2 times on the peripheral blood	0.5 ml x 4<br>= 2 ml
	**Total blood volume on day 4**	**2 ml**
**Day 7 ±1**	• Blood for Haematology/ Complete blood count, Parasite count (Thick and thin blood film) and heamatocrite	3 ml
	• Blood for Biochemistry (Glucose, BUN, Creatinine, AST,ALT,ALP,total bilirubin, electrolyte (Na,K,tCO_2),calcium	5 ml
	**Total blood volume on follow up day(Day 7±1)**	**8 ml**
**Day 14 ±1**	• Blood for Complete blood count, Parasite count (Thick and thin blood film) and haematocrite	3 ml
	• Blood for Biochemistry (Glucose, BUN, Creatinine, AST,ALT, ALP,total bilirubin, electrolyte (Na,K,tCO_2),calcium	5 ml
	• Urine pregnancy test	1 ml
	**Total blood volume on follow up day(Day 14±1)**	**8 ml**<br>**Urine 1 ml**

The total blood volume is 59.5 ml (3.9 table spoonfuls), 7 ml of urine
