# Supplementary material for: Inhibition of merozoite invasion and transient de-sequestration by sevuparin in humans with Plasmodium falciparum malaria
Source: PLoS One. 2017 Dec 15;12(12):e0188754. doi: 10.1371/journal.pone.0188754 (PMC5731734; doi:10.1371/journal.pone.0188754)
Supplement: S4 Supportive Information — (PDF) [file pone.0188754.s004.pdf]

16 APPENDICES

16.2 Subject data listings (fully anonymized)

16.2.1 Discontinued subjects

None

16.2.2 Protocol deviations

16.2.3 Subjects excluded from the efficacy analysis (if applicable)

Not applicable.

16.2.4 Demographic data and other baseline characteristics

16.2.4.1 Demographics

SUBJID	INITIAL	ARM	SEX	ETHNIC	BIRTHDATE
		1.5 mg/kg	Female	BURMESE	
		1.5 mg/kg	Female	BURMESE	
		1.5 mg/kg	Male	BURMESE	
		3 mg/kg	Male	BURMESE	
		3 mg/kg	Male	BURMESE	
		3 mg/kg	Male	BURMESE	
		6 mg/kg	Male	BURMESE	
		6 mg/kg	Male	BURMESE	
		6 mg/kg	Female	BURMESE	

TSM02 Individual subject listings part 1

CSR Appendix 16.2

16.2.4.2 Baseline symptoms

SUBJID	INITIAL	ARM	FEVER	DUR (Days)	DIZZI	DUR (Days)	NAUSEA	DUR (Days)	ANOREXIA	DUR (Days) *99=UNKNOWN*	VOMIT	DUR (Days)	TIMES in 24HOURS
		1.5 mg/kg	Yes	3	No		Yes	2	No		Yes	2	10
		1.5 mg/kg	Yes	3	No		Yes	2	Yes	2	Yes	2	3
		1.5 mg/kg	Yes	3	No		Yes	3	Yes	3	No		
		3 mg/kg	Yes	2	No		No		No		No		
		3 mg/kg	Yes	5	No		No		Yes	3	No		
		3 mg/kg	Yes	3	No		No		Yes	3	No		
		6 mg/kg	Yes	2	Yes	1	No		No		No		
		6 mg/kg	Yes	2	No		No		No		No		
		6 mg/kg	Yes	4	No		No		Yes	99	No		

16.2.4.2 Baseline symptoms (-continued)

		ARM	DIARRHOEA	ABD PAIN	DUR (Days)	PAIN AREA	ITCHING	SKIN RASH	JOINT PAIN	MUSCLE PAIN	DUR (Days)	PALPITATIONS
		1.5 mg/kg	No	No			No	No		Yes	1	No
		1.5 mg/kg	No	No			No	No		No		No
		1.5 mg/kg	No	No			No	No		No		No
		3 mg/kg	No	No			No	No		No		No
		3 mg/kg	No	Yes	3	EPI	No	No		Yes	2	No
		3 mg/kg	No	No			No	No		Yes	3	No
		6 mg/kg	No	No			No	No		No		No
		6 mg/kg	No	No			No	No		Yes	1	No
		6 mg/kg	No	No			No	No		Yes	2	No

16.2.4.2 Baseline symptoms (-continued)

SUBJID	INITIAL	ARM	DIFFICULTY BREATHING	DIFFICULTY HEARING	CONFUSION	BLURRED VISION	TIREDNESS	HEADACHE	DUR (Days)
		1.5 mg/kg	No	No	No	No	No	No	
		1.5 mg/kg	No	No	No	No	No	Yes	3
		1.5 mg/kg	No	No	No	No	No	Yes	3
		3 mg/kg	No	No	No	No	No	No	
		3 mg/kg	No	No	No	No	No	Yes	5
		3 mg/kg	No	No	No	No	No	Yes	3
		6 mg/kg	No	No	No	No	No	Yes	2
		6 mg/kg	No	No	No	No	No	Yes	2
		6 mg/kg	No	No	No	No	No	Yes	4

16.2.4.3 Significant past medical history

None

16.2.4.4 Drug history

None

16.2.5 Compliance and drug concentration data

16.2.5.1 Listing of drug administration data (Malanil administration)

SUBJID	INITIAL	ARM	DOSE	DATE	TIME	TABs	VOMIT	VOMIT TIME	RETREAT	RETREAT TIME	TABs
		1.5 mg/kg	1		17:55	4	Yes	19:45	Yes	20:20	2
		1.5 mg/kg	2		16:00	4	No		No		
		1.5 mg/kg	3		15:45	4	No		No		
		1.5 mg/kg	1		15:35	4	No		No		
		1.5 mg/kg	2		15:00	4	No		No		
		1.5 mg/kg	3		15:00	4	No		No		
		1.5 mg/kg	1		15:00	4	No		No		
		1.5 mg/kg	2		14:45	4	No		No		
		1.5 mg/kg	3		14:45	4	No		No		
		3 mg/kg	1		17:32	4	No		No		
		3 mg/kg	2		17:00	4	No		No		
		3 mg/kg	3		17:05	4	No		No		
		3 mg/kg	1		18:52	4	No		No		
		3 mg/kg	2		18:00	4	No		No		
		3 mg/kg	3		18:10	4	No		No		
		3 mg/kg	1		17:55	4	No		No		
		3 mg/kg	2		18:00	4	No		No		
		3 mg/kg	3		18:00	4	No		No		
		6 mg/kg	1		15:45	4	No		No		
		6 mg/kg	2		15:40	4	No		No		
		6 mg/kg	3		15:45	4	No		No		
		6 mg/kg	1		16:25	4	No		No		
		6 mg/kg	2		16:00	4	No		No		
		6 mg/kg	3		16:00	4	No		No		
		6 mg/kg	1		16:30	4	No		No		
		6 mg/kg	2		16:45	4	No		No		
		6 mg/kg	3		16:35	4	No		No		

16.2.5.2 Listing of drug administration data (Sevuparin administration)

SUBJID	INITIAL	ARM	DOSE	DATE	TIME	DRUG AGMIN (ml)
		1.5 mg/kg	1		18:00	20
		1.5 mg/kg	2		23:55	20
		1.5 mg/kg	3		05:58	20
		1.5 mg/kg	4		11:55	20
		1.5 mg/kg	5		18:05	20
		1.5 mg/kg	6		23:55	20
		1.5 mg/kg	7		06:00	20
		1.5 mg/kg	8		11:53	20
		1.5 mg/kg	9		17:55	20
		1.5 mg/kg	10		23:57	20
		1.5 mg/kg	11		06:00	20
		1.5 mg/kg	12		11:55	20
		1.5 mg/kg	1		15:46	20
		1.5 mg/kg	2		21:45	20
		1.5 mg/kg	3		03:50	20
		1.5 mg/kg	4		09:46	20
		1.5 mg/kg	5		15:55	20
		1.5 mg/kg	6		21:40	20
		1.5 mg/kg	7		03:43	20
		1.5 mg/kg	8		09:46	20
		1.5 mg/kg	9		15:46	20
		1.5 mg/kg	10		21:40	20
		1.5 mg/kg	11		03:42	20
		1.5 mg/kg	12		10:00	20
		1.5 mg/kg	1		14:55	20
		1.5 mg/kg	2		20:57	20
		1.5 mg/kg	3		02:53	20
		1.5 mg/kg	4		08:58	20
		1.5 mg/kg	5		14:55	20
		1.5 mg/kg	6		20:55	20
		1.5 mg/kg	7		02:57	20
		1.5 mg/kg	8		08:55	20
		1.5 mg/kg	9		15:00	20
		1.5 mg/kg	10		20:55	20

SUBJID	INITIAL	ARM	DOSE	DATE	TIME	DRUG AGMIN (ml)
		1.5 mg/kg	11		02:55	20
		1.5 mg/kg	12		09:00	20
		3 mg/kg	1		18:00	20
		3 mg/kg	2		23:58	20
		3 mg/kg	3		05:58	20
		3 mg/kg	4		11:57	20
		3 mg/kg	5		17:50	20
		3 mg/kg	6		00:00	20
		3 mg/kg	7		05:57	20
		3 mg/kg	8		11:54	20
		3 mg/kg	9		17:50	20
		3 mg/kg	10		23:52	23.5
		3 mg/kg	11		05:58	20
		3 mg/kg	12		11:57	20
		3 mg/kg	1		18:55	20
		3 mg/kg	2		01:00	20
		3 mg/kg	3		07:00	20
		3 mg/kg	4		13:00	20
		3 mg/kg	5		19:00	20
		3 mg/kg	6		00:55	20
		3 mg/kg	7		07:00	20
		3 mg/kg	8		13:00	20
		3 mg/kg	9		19:00	20
		3 mg/kg	10		01:00	20
		3 mg/kg	11		06:55	20
		3 mg/kg	12		13:00	20
		3 mg/kg	1		18:00	20
		3 mg/kg	2		23:58	20
		3 mg/kg	3		06:00	20
		3 mg/kg	4		11:55	20
		3 mg/kg	5		17:59	20
		3 mg/kg	6		00:00	20
		3 mg/kg	7		06:05	20
		3 mg/kg	8		12:00	20
		3 mg/kg	9		18:00	20
		3 mg/kg	10		00:00	20
		3 mg/kg	11		06:00	20

SUBJID	INITIAL	ARM	DOSE	DATE	TIME	DRUG AGMIN (ml)
		3 mg/kg	12		12:00	20
		6 mg/kg	1		15:55	20
		6 mg/kg	2		22:00	20
		6 mg/kg	3		04:03	20
		6 mg/kg	4		10:00	20
		6 mg/kg	5		16:00	20
		6 mg/kg	6		21:57	20
		6 mg/kg	7		04:02	20
		6 mg/kg	8		10:00	20
		6 mg/kg	9		16:00	20
		6 mg/kg	10		22:00	20
		6 mg/kg	11		04:00	20
		6 mg/kg	12		10:00	20
		6 mg/kg	1		16:30	20
		6 mg/kg	2		22:26	20
		6 mg/kg	3		04:35	20
		6 mg/kg	4		10:28	20
		6 mg/kg	5		16:30	20
		6 mg/kg	6		22:30	20
		6 mg/kg	7		04:30	20
		6 mg/kg	8		10:30	20
		6 mg/kg	9		16:30	20
		6 mg/kg	10		22:30	20
		6 mg/kg	11		04:30	20
		6 mg/kg	12		10:30	20
		6 mg/kg	1		16:55	20
		6 mg/kg	2		23:00	20
		6 mg/kg	3		05:00	20
		6 mg/kg	4		11:00	20
		6 mg/kg	5		17:00	20
		6 mg/kg	6		22:55	20
		6 mg/kg	7		05:00	20
		6 mg/kg	8		11:00	20
		6 mg/kg	9		17:00	20
		6 mg/kg	10		22:50	20
		6 mg/kg	11		05:00	20
		6 mg/kg	12		11:00	20

16.2.6 Individual efficacy response data

16.2.6.1 Malaria Blood smear

(ND = Not Done)

SUBJID	INITIAL	ARM	TIMEPOINT	SPECIES	PARASITAEMIA (/µL)	Tiny Rings (%)	Small Rings (%)	Large Rings (%)	Early Troph (%)	Mid Troph (%)	Late Troph (%)	Gamet (%)	Schiz (%)
		1.5 mg/kg	SCREENING	Pf	Not available	79	15	6	0	0	0	0	0
		1.5 mg/kg	D1H0	Pf	Not available	43	45	10	2	0	0	0	0
		1.5 mg/kg	D1H0:30	Pf	Not available	48	41	8	3	0	0	0	0
		1.5 mg/kg	D1H1	Pf	Not available	33	40	17	9	1	0	0	0
		1.5 mg/kg	D1H1:30	Pf	Not available	35	48	11	6	0	0	0	0
		1.5 mg/kg	D2H2	Pf	Not available	11	45	27	17	0	0	0	0
		1.5 mg/kg	D1H3	Pf	Not available	17	31	22	25	5	0	0	0
		1.5 mg/kg	D1H4	Pf	Not available	82	15	0	2	1	0	0	0
		1.5 mg/kg	D1H6	Pf	Not available	83	12	3	2	0	0	0	0
		1.5 mg/kg	D1H8	Pf	Not available	68	12	4	15	1	0	0	0
		1.5 mg/kg	D1H10	Pf	Not available	57	15	3	25	0	0	0	0
		1.5 mg/kg	D1H12	Pf	Not available	38	40	9	13	0	0	0	0
		1.5 mg/kg	D1H18	Pf	Not available	15	33	22	27	3	0	0	0
		1.5 mg/kg	D2H24	Pf	Not available	10	32	8	44	6	0	0	0
		1.5 mg/kg	D2H30	Pf	Not available	ND	ND	ND	ND	ND	ND	ND	ND
		1.5 mg/kg	D2H36	Pf	Not available	ND	ND	ND	ND	ND	ND	ND	ND
		1.5 mg/kg	D2H42	Pf	Not available	ND	ND	ND	ND	ND	ND	ND	ND
		1.5 mg/kg	D3H48	Pf	Not available	ND	ND	ND	ND	ND	ND	ND	ND

SUBJID	INITIAL	ARM	TIMEPOINT	SPECIES	PARASITAEMIA (/µL)	Tiny Rings (%)	Small Rings (%)	Large Rings (%)	Early Troph (%)	Mid Troph (%)	Late Troph (%)	Gamet (%)	Schiz (%)
		1.5 mg/kg	D3H54	Pf	Not available	ND	ND	ND	ND	ND	ND	ND	ND
		1.5 mg/kg	D3H60	Pf	Not available	ND	ND	ND	ND	ND	ND	ND	ND
		1.5 mg/kg	D3H66	Pf	Not available	ND	ND	ND	ND	ND	ND	ND	ND
		1.5 mg/kg	D7	NEGATIVE	Not available	ND	ND	ND	ND	ND	ND	ND	ND
		1.5 mg/kg	D14	NEGATIVE	Not available	ND	ND	ND	ND	ND	ND	ND	ND
		1.5 mg/kg	SCREENING	Pf	97290	ND	ND	ND	ND	ND	ND	ND	ND
		1.5 mg/kg	D1H0	Pf	197400	71	25	4	0	0	0	0	0
		1.5 mg/kg	D1H0:30	Pf	193200	79	18	3	0	0	0	0	0
		1.5 mg/kg	D1H1	Pf	180600	71	25	2	1	0	0	0	1
		1.5 mg/kg	D1H1:30	Pf	193200	70	18	6	6	0	0	0	0
		1.5 mg/kg	D2H2	Pf	176400	72	25	3	0	0	0	0	0
		1.5 mg/kg	D1H3	Pf	184800	49	39	11	1	0	0	0	0
		1.5 mg/kg	D1H4	Pf	184800	17	43	26	14	0	0	0	0
		1.5 mg/kg	D1H6	Pf	168000	55	30	8	7	0	0	0	0
		1.5 mg/kg	D1H8	Pf	151200	32	52	15	1	0	0	0	0
		1.5 mg/kg	D1H10	Pf	168000	57	26	10	7	0	0	0	0
		1.5 mg/kg	D1H12	Pf	155400	12	40	33	15	0	0	0	0
		1.5 mg/kg	D1H18	Pf	155400	8	35	26	31	0	0	0	0
		1.5 mg/kg	D2H24	Pf	70000	3	22	28	47	0	0	0	0
		1.5 mg/kg	D2H30	Pf	56000	ND	ND	ND	ND	ND	ND	ND	ND
		1.5 mg/kg	D2H36	Pf	13170.5	ND	ND	ND	ND	ND	ND	ND	ND
		1.5 mg/kg	D2H42	Pf	9990.5	ND	ND	ND	ND	ND	ND	ND	ND

SUBJID	INITIAL	ARM	TIMEPOINT	SPECIES	PARASITAEMIA (/µL)	Tiny Rings (%)	Small Rings (%)	Large Rings (%)	Early Troph (%)	Mid Troph (%)	Late Troph (%)	Gamet (%)	Schiz (%)
		1.5 mg/kg	D3H48	Pf	1827	ND	ND	ND	ND	ND	ND	ND	ND
		1.5 mg/kg	D3H54	Pf	1596	ND	ND	ND	ND	ND	ND	ND	ND
		1.5 mg/kg	D3H60	Pf	525	ND	ND	ND	ND	ND	ND	ND	ND
		1.5 mg/kg	D3H66	Pf	105	ND	ND	ND	ND	ND	ND	ND	ND
		1.5 mg/kg	D4H72	Pf	60	ND	ND	ND	ND	ND	ND	ND	ND
		1.5 mg/kg	D7	NEGATIVE	Not available	ND	ND	ND	ND	ND	ND	ND	ND
		1.5 mg/kg	D14	NEGATIVE	Not available	ND	ND	ND	ND	ND	ND	ND	ND
		1.5 mg/kg	SCREENING	Pf	95000	90	8	2	0	0	0	0	0
		1.5 mg/kg	D1H0	Pf	152000	84	14	2	0	0	0	0	0
		1.5 mg/kg	D1H0:30	Pf	133000	65	30	4	1	0	0	0	0
		1.5 mg/kg	D1H1	Pf	161500	57	36	5	2	0	0	0	0
		1.5 mg/kg	D1H1:30	Pf	171000	44	38	8	10	0	0	0	0
		1.5 mg/kg	D2H2	Pf	137750	47	41	8	4	0	0	0	0
		1.5 mg/kg	D1H3	Pf	128250	42	39	12	7	0	0	0	0
		1.5 mg/kg	D1H4	Pf	104500	64	22	5	9	0	0	0	0
		1.5 mg/kg	D1H6	Pf	109250	ND	ND	ND	ND	ND	ND	ND	ND
		1.5 mg/kg	D1H8	Pf	114000	63	17	9	11	0	0	0	0
		1.5 mg/kg	D1H10	Pf	114000	57	29	6	8	0	0	0	0
		1.5 mg/kg	D1H12	Pf	114000	34	23	16	27	0	0	0	0
		1.5 mg/kg	D1H18	Pf	76000	17	33	8	42	0	0	0	0
		1.5 mg/kg	D2H24	Pf	60000	15	17	22	46	0	0	0	0
		1.5 mg/kg	D2H30	Pf	36000	ND	ND	ND	ND	ND	ND	ND	ND

SUBJID	INITIAL	ARM	TIMEPOINT	SPECIES	PARASITAEMIA (/µL)	Tiny Rings (%)	Small Rings (%)	Large Rings (%)	Early Troph (%)	Mid Troph (%)	Late Troph (%)	Gamet (%)	Schiz (%)
		1.5 mg/kg	D2H36	Pf	10464	ND	ND	ND	ND	ND	ND	ND	ND
		1.5 mg/kg	D2H42	Pf	8128	ND	ND	ND	ND	ND	ND	ND	ND
		1.5 mg/kg	D3H48	Pf	4050	ND	ND	ND	ND	ND	ND	ND	ND
		1.5 mg/kg	D3H54	Pf	2760	ND	ND	ND	ND	ND	ND	ND	ND
		1.5 mg/kg	D3H60	Pf	960	ND	ND	ND	ND	ND	ND	ND	ND
		1.5 mg/kg	D3H66	Pf	720	ND	ND	ND	ND	ND	ND	ND	ND
		1.5 mg/kg	D4H72	Pf	60	ND	ND	ND	ND	ND	ND	ND	ND
		1.5 mg/kg	D7	NEGATIVE	Not available	ND	ND	ND	ND	ND	ND	ND	ND
		1.5 mg/kg	D14	NEGATIVE	Not available	ND	ND	ND	ND	ND	ND	ND	ND
		3 mg/kg	SCREENING	Pf	71400	ND	ND	ND	ND	ND	ND	ND	ND
		3 mg/kg	D1H0	Pf	65450	60	39	1	0	0	0	0	0
		3 mg/kg	D1H0:30	Pf	59500	56	43	1	0	0	0	0	0
		3 mg/kg	D1H1	Pf	83300	52	43	5	0	0	0	0	0
		3 mg/kg	D1H1:30	Pf	95200	45	47	8	0	0	0	0	0
		3 mg/kg	D2H2	Pf	89250	18	43	26	13	0	0	0	0
		3 mg/kg	D1H3	Pf	77350	63	34	2	1	0	0	0	0
		3 mg/kg	D1H4	Pf	47600	36	46	10	8	0	0	0	0
		3 mg/kg	D1H6	Pf	64900	13	40	16	31	0	0	0	0
		3 mg/kg	D1H8	Pf	41650	24	29	22	25	0	0	0	0
		3 mg/kg	D1H10	Pf	29750	6	25	22	47	0	0	0	0
		3 mg/kg	D1H12	Pf	35400	4	17	22	56	1	0	0	0
		3 mg/kg	D1H18	Pf	53550	0	12	28	57	3	0	0	0

SUBJID	INITIAL	ARM	TIMEPOINT	SPECIES	PARASITAEMIA (/µL)	Tiny Rings (%)	Small Rings (%)	Large Rings (%)	Early Troph (%)	Mid Troph (%)	Late Troph (%)	Gamet (%)	Schiz (%)
		3 mg/kg	D2H24	Pf	8169	0	8	22	61	9	0	0	0
		3 mg/kg	D2H30	Pf	2434	ND	ND	ND	ND	ND	ND	ND	ND
		3 mg/kg	D2H36	Pf	1116	ND	ND	ND	ND	ND	ND	ND	ND
		3 mg/kg	D2H42	Pf	620	ND	ND	ND	ND	ND	ND	ND	ND
		3 mg/kg	D3H48	Pf	216	ND	ND	ND	ND	ND	ND	ND	ND
		3 mg/kg	D3H54	Pf	108	ND	ND	ND	ND	ND	ND	ND	ND
		3 mg/kg	D3H60	Pf	72	ND	ND	ND	ND	ND	ND	ND	ND
		3 mg/kg	D3H66	Pf	36	ND	ND	ND	ND	ND	ND	ND	ND
		3 mg/kg	D4H72	NEGATIVE	Not available	ND	ND	ND	ND	ND	ND	ND	ND
		3 mg/kg	D7	NEGATIVE	Not available	ND	ND	ND	ND	ND	ND	ND	ND
		3 mg/kg	D14	NEGATIVE	Not available	ND	ND	ND	ND	ND	ND	ND	ND
		3 mg/kg	SCREENING	Pf	38000	ND	ND	ND	ND	ND	ND	ND	ND
		3 mg/kg	D1H0	Pf	34047	67	23	7	2	0	0	1	0
		3 mg/kg	D1H0:30	Pf	20547	45	42	8	5	0	0	0	0
		3 mg/kg	D1H1	Pf	30996	46	34	13	7	0	0	0	0
		3 mg/kg	D1H1:30	Pf	25326	37	44	10	8	0	0	1	0
		3 mg/kg	D2H2	Pf	28863	45	31	12	12	0	0	0	0
		3 mg/kg	D1H3	Pf	44631	14	49	25	12	0	0	0	0
		3 mg/kg	D1H4	Pf	52731	12	50	20	18	0	0	0	0
		3 mg/kg	D1H6	Pf	37422	14	33	16	37	0	0	0	0
		3 mg/kg	D1H8	Pf	29727	12	32	14	42	0	0	0	0
		3 mg/kg	D1H10	Pf	29889	18	35	22	24	0	0	1	0

SUBJID	INITIAL	ARM	TIMEPOINT	SPECIES	PARASITAEMIA (/µL)	Tiny Rings (%)	Small Rings (%)	Large Rings (%)	Early Troph (%)	Mid Troph (%)	Late Troph (%)	Gamet (%)	Schiz (%)
		3 mg/kg	D1H12	Pf	37530	1	23	34	41	0	0	1	0
		3 mg/kg	D1H18	Pf	19764	0	9	22	67	1	0	1	0
		3 mg/kg	D2H24	Pf	16816	0	2	17	76	5	0	0	0
		3 mg/kg	D2H30	Pf	6064	ND	ND	ND	ND	ND	ND	ND	ND
		3 mg/kg	D2H36	Pf	832	ND	ND	ND	ND	ND	ND	ND	ND
		3 mg/kg	D2H42	Pf	176	ND	ND	ND	ND	ND	ND	ND	ND
		3 mg/kg	D3H48	Pf	34	ND	ND	ND	ND	ND	ND	ND	ND
		3 mg/kg	D3H54	Pf	51	ND	ND	ND	ND	ND	ND	ND	ND
		3 mg/kg	D3H60	Pf	17	ND	ND	ND	ND	ND	ND	ND	ND
		3 mg/kg	D3H66	NEGATIVE	Not available	ND	ND	ND	ND	ND	ND	ND	ND
		3 mg/kg	D4H72	NEGATIVE	Not available	ND	ND	ND	ND	ND	ND	ND	ND
		3 mg/kg	D7	NEGATIVE	Not available	ND	ND	ND	ND	ND	ND	ND	ND
		3 mg/kg	D14	NEGATIVE	Not available	ND	ND	ND	ND	ND	ND	ND	ND
		3 mg/kg	SCREENING	Pf	10200	0	7	13	60	20	0	0	0
		3 mg/kg	D1H0	Pf	882	0	14	36	32	18	0	0	0
		3 mg/kg	D1H0:30	Pf	924	18	32	20	22	6	2	0	0
		3 mg/kg	D1H1	Pf	462	6	40	20	30	4	0	0	0
		3 mg/kg	D1H1:30	Pf	420	12	36	20	20	12	0	0	0
		3 mg/kg	D2H2	Pf	420	6	32	26	28	8	0	0	0
		3 mg/kg	D1H3	Pf	210	2	28	34	30	6	0	0	0
		3 mg/kg	D1H4	Pf	126	16	26	26	32	0	0	0	0
		3 mg/kg	D1H6	Pf	462	ND	ND	ND	ND	ND	ND	ND	ND

SUBJID	INITIAL	ARM	TIMEPOINT	SPECIES	PARASITAEMIA (/µL)	Tiny Rings (%)	Small Rings (%)	Large Rings (%)	Early Troph (%)	Mid Troph (%)	Late Troph (%)	Gamet (%)	Schiz (%)
		3 mg/kg	D1H8	Pf	378	ND	ND	ND	ND	ND	ND	ND	ND
		3 mg/kg	D1H10	Pf	210	ND	ND	ND	ND	ND	ND	ND	ND
		3 mg/kg	D1H12	Pf	84	ND	ND	ND	ND	ND	ND	ND	ND
		3 mg/kg	D1H18	NEGATIVE	Not available	ND	ND	ND	ND	ND	ND	ND	ND
		3 mg/kg	D2H24	Pf	38	ND	ND	ND	ND	ND	ND	ND	ND
		3 mg/kg	D2H30	Pf	152	ND	ND	ND	ND	ND	ND	ND	ND
		3 mg/kg	D2H36	Pf	76	ND	ND	ND	ND	ND	ND	ND	ND
		3 mg/kg	D2H42	NEGATIVE	Not available	ND	ND	ND	ND	ND	ND	ND	ND
		3 mg/kg	D3H48	NEGATIVE	Not available	ND	ND	ND	ND	ND	ND	ND	ND
		3 mg/kg	D3H54	NEGATIVE	Not available	ND	ND	ND	ND	ND	ND	ND	ND
		3 mg/kg	D3H60	NEGATIVE	Not available	ND	ND	ND	ND	ND	ND	ND	ND
		3 mg/kg	D3H66	NEGATIVE	Not available	ND	ND	ND	ND	ND	ND	ND	ND
		3 mg/kg	D7	NEGATIVE	Not available	ND	ND	ND	ND	ND	ND	ND	ND
		3 mg/kg	D14	NEGATIVE	Not available	ND	ND	ND	ND	ND	ND	ND	ND
		6 mg/kg	SCREENING	Pf	86700	1	18	34	47	0	0	0	0
		6 mg/kg	D1H0	Pf	51000	8	24	20	46	2	0	0	0
		6 mg/kg	D1H0:30	Pf	61200	20	26	9	36	8	1	0	0
		6 mg/kg	D1H1	Pf	51000	22	17	11	35	11	4	0	0
		6 mg/kg	D1H1:30	Pf	45900	ND	ND	ND	ND	ND	ND	ND	ND
		6 mg/kg	D2H2	Pf	35700	40	21	8	26	5	0	0	0
		6 mg/kg	D1H3	Pf	35700	40	31	7	16	6	0	0	0
		6 mg/kg	D1H4	Pf	30600	50	34	6	10	0	0	0	0

SUBJID	INITIAL	ARM	TIMEPOINT	SPECIES	PARASITAEMIA (/µL)	Tiny Rings (%)	Small Rings (%)	Large Rings (%)	Early Troph (%)	Mid Troph (%)	Late Troph (%)	Gamet (%)	Schiz (%)
		6 mg/kg	D1H6	Pf	25500	5	36	42	15	2	0	0	0
		6 mg/kg	D1H8	Pf	15300	19	38	20	22	1	0	0	0
		6 mg/kg	D1H10	Pf	10200	1	31	32	36	0	0	0	0
		6 mg/kg	D1H12	Pf	9590	1	20	36	42	0	1	0	0
		6 mg/kg	D1H18	Pf	5845	4	22	28	46	0	0	0	0
		6 mg/kg	D2H24	Pf	1230	0	8	36	52	0	4	0	0
		6 mg/kg	D2H30	Pf	1650	ND	ND	ND	ND	ND	ND	ND	ND
		6 mg/kg	D2H36	Pf	360	ND	ND	ND	ND	ND	ND	ND	ND
		6 mg/kg	D2H42	Pf	60	ND	ND	ND	ND	ND	ND	ND	ND
		6 mg/kg	D3H48	Pf	12	ND	ND	ND	ND	ND	ND	ND	ND
		6 mg/kg	D3H54	NEGATIVE	0	ND	ND	ND	ND	ND	ND	ND	ND
		6 mg/kg	D3H60	NEGATIVE	0	ND	ND	ND	ND	ND	ND	ND	ND
		6 mg/kg	D3H66	NEGATIVE	0	ND	ND	ND	ND	ND	ND	ND	ND
		6 mg/kg	D7	NEGATIVE	Not available	ND	ND	ND	ND	ND	ND	ND	ND
		6 mg/kg	D14	NEGATIVE	Not available	ND	ND	ND	ND	ND	ND	ND	ND
		6 mg/kg	SCREENING	Pf	33360	ND	ND	ND	ND	ND	ND	ND	ND
		6 mg/kg	D1H0	Pf	68200	23	43	27	7	0	0	0	0
		6 mg/kg	D1H0:30	Pf	62000	17	54	20	9	0	0	0	0
		6 mg/kg	D1H1	Pf	55800	20	45	28	7	0	0	0	0
		6 mg/kg	D1H1:30	Pf	19600	6	59	25	10	0	0	0	0
		6 mg/kg	D2H2	Pf	49600	6	49	34	11	0	0	0	0
		6 mg/kg	D1H3	Pf	43400	32	29	17	21	1	0	0	0

SUBJID	INITIAL	ARM	TIMEPOINT	SPECIES	PARASITAEMIA (/µL)	Tiny Rings (%)	Small Rings (%)	Large Rings (%)	Early Troph (%)	Mid Troph (%)	Late Troph (%)	Gamet (%)	Schiz (%)
		6 mg/kg	D1H4	Pf	37200	10	58	12	20	0	0	0	0
		6 mg/kg	D1H6	Pf	24800	9	49	27	15	0	0	0	0
		6 mg/kg	D1H8	Pf	Not available	3	25	36	36	0	0	0	0
		6 mg/kg	D1H10	Pf	18600	2	23	29	46	0	0	0	0
		6 mg/kg	D1H12	Pf	40320	4	19	20	57	0	0	0	0
		6 mg/kg	D1H18	Pf	38352	1	2	23	71	3	0	0	0
		6 mg/kg	D2H24	Pf	16470	1	2	23	73	1	0	0	0
		6 mg/kg	D2H30	Pf	9058	ND	ND	ND	ND	ND	ND	ND	ND
		6 mg/kg	D2H36	Pf	2836	ND	ND	ND	ND	ND	ND	ND	ND
		6 mg/kg	D2H42	Pf	3355	ND	ND	ND	ND	ND	ND	ND	ND
		6 mg/kg	D3H48	Pf	130	ND	ND	ND	ND	ND	ND	ND	ND
		6 mg/kg	D3H54	Pf	13	ND	ND	ND	ND	ND	ND	ND	ND
		6 mg/kg	D3H60	NEGATIVE	Not available	ND	ND	ND	ND	ND	ND	ND	ND
		6 mg/kg	D3H66	NEGATIVE	Not available	ND	ND	ND	ND	ND	ND	ND	ND
		6 mg/kg	D7	NEGATIVE	Not available	ND	ND	ND	ND	ND	ND	ND	ND
		6 mg/kg	D14	NEGATIVE	Not available	ND	ND	ND	ND	ND	ND	ND	ND
		6 mg/kg	SCREENING	Pf	20020	ND	ND	ND	ND	ND	ND	ND	ND
		6 mg/kg	D1H0	Pf	20817	59	32	5	4	0	0	0	0
		6 mg/kg	D1H0:30	Pf	16417	63	26	5	5	1	0	0	0
		6 mg/kg	D1H1	Pf	17187	49	40	8	2	1	0	0	0
		6 mg/kg	D1H1:30	Pf	17407	49	37	10	4	0	0	0	0
		6 mg/kg	D2H2	Pf	17710	40	42	14	4	0	0	0	0

SUBJID	INITIAL	ARM	TIMEPOINT	SPECIES	PARASITAEMIA (/µL)	Tiny Rings (%)	Small Rings (%)	Large Rings (%)	Early Troph (%)	Mid Troph (%)	Late Troph (%)	Gamet (%)	Schiz (%)
		6 mg/kg	D1H3	Pf	23127	7	47	26	19	1	0	0	0
		6 mg/kg	D1H4	Pf	24365	10	39	31	20	0	0	0	0
		6 mg/kg	D1H6	Pf	26922	9	53	24	12	1	0	1	0
		6 mg/kg	D1H8	Pf	13997	13	40	37	10	0	0	0	0
		6 mg/kg	D1H10	Pf	21862	0	42	36	21	0	0	1	0
		6 mg/kg	D1H12	Pf	19057	10	5	35	45	5	0	0	0
		6 mg/kg	D1H18	Pf	16637	1	10	19	59	9	2	0	0
		6 mg/kg	D2H24	Pf	8500	4	4	18	72	2	0	0	0
		6 mg/kg	D2H30	Pf	7900	ND	ND	ND	ND	ND	ND	ND	ND
		6 mg/kg	D2H36	Pf	1020	ND	ND	ND	ND	ND	ND	ND	ND
		6 mg/kg	D2H42	Pf	160	ND	ND	ND	ND	ND	ND	ND	ND
		6 mg/kg	D3H48	Pf	9	ND	ND	ND	ND	ND	ND	ND	ND
		6 mg/kg	D3H54	NEGATIVE	0	ND	ND	ND	ND	ND	ND	ND	ND
		6 mg/kg	D3H60	NEGATIVE	0	ND	ND	ND	ND	ND	ND	ND	ND
		6 mg/kg	D7	NEGATIVE	0	ND	ND	ND	ND	ND	ND	ND	ND
		6 mg/kg	D14	NEGATIVE	0	ND	ND	ND	ND	ND	ND	ND	ND

16.2.6.2 Cytoadherance, rosette assay

To be reported later.

16.2.7 Individual safety data

16.2.7.1 Adverse event listings

SUBJ ID	INITIAL	ARM	AE SEQ	AETERM	AEDECOD	AESOC	DESCRIPTION	ONSET DATE TIME *99:99=UNK*	END DATE	OUTCOME	INTENS	RELAT. DF02	RELAT. MALANIL	ALT RELAT	ALTRELOTHER	ACTION
		1.5 mg/kg	1	VOMITING	Vomiting	Gastrointestinal disorders	SHE HAD FEVER AND VOMITING WITH MILD DEHYDRATION. THE INTRAVENOUS FLUID AND METOCLOPLAMIDE (PLASIL) WERE GIVEN.			Recovered	Mild	Not related	Not related	Concurrent illness	UNCOMPLICATED PF MALARIA	Treatmen[illegible] Medicatio[illegible]
		1.5 mg/kg	2	HYPOKALEMIA	Hypokalaemia	Metabolism and nutrition disorders	HER SERUM K+=3.1mmol/L AND SERUM Ca2+ WAS 6.9mmol/L BUT NO CLINICAL SIGNIFICANT. SHE GOT 10% CALCIUM GLUCONATE 10ml IV SLOWLY PUSH AND 30ml KCl ELIXIR.			Recovered	Mild	Not related	Not related	Concurrent illness	UNCOMPLICATED PF MALARIA	Treatmen[illegible] Medicatio[illegible]
		1.5	3	HYPOCALCEMI	Hypocalca	Metabolis	HER SERUM			Recovered	Mild	Not	Not	Concurrent	UNCOMPLI	Treatmen[illegible]

SUBJ ID	INITIAL	ARM	AE SEQ	AETERM	AEDECOD	AESOC	DESCRIPTION	ONSET DATE TIME *99:99=UNK*	END DATE	OUTCOME	INTENS	RELAT. DF02	RELAT. MALANIL	ALT RELAT	ALTRELOTHER	ACTIO
		mg/kg		A	emia	m and nutrition disorders	K+=3.1mmol/L AND SERUM Ca2+ WAS 6.9 mmol/L BUT NO CLINICAL SIGNIFICANT. SHE GOT 10% CALCIUM GLUCONATE 10 ML IV SLOWLY PUSH AND 30ML KCl ELIXIR					related	related	illness	CATED PF MALARIA	Medicatio
		1.5 mg/kg	1	NAUSEA	Nausea	Gastrointestinal disorders	IN DAY 2, PATIENT HAS NAUSEA AND VOMITING SYMPTOM. HE DID NOT HAVE THE SYMPTOM BEFORE. I THINK THESE SYMPTOM ARE RELATED WITH PF MALARIAL INFECTION, TYPICALLY.			Recovered	Mild	Not related	Not related	Concurrent illness	MALARIA INFECTION	Treatment Medicatio
		1.5 mg/kg	2	VOMITING	Vomiting	Gastrointestinal disorders	IN DAY 2, PATIENT HAS NAUSEA AND			Recovered	Mild	Not related	Not related	Concurrent illness	MALARIA INFECTION	Treatment Medicatio

SUBJ ID	INITIAL	ARM	AE SEQ	AETERM	AEDECOD	AESOC	DESCRIPTION	ONSET DATE TIME *99:99=UNK*	END DATE	OUTCOME	INTENS	RELAT. DF02	RELAT. MALANIL	ALT RELAT	ALTRELOTHER	ACTION
							VOMITING SYMPTOM. HE DID NOT HAVE THE SYMPTOM BEFORE. I THINK THESE SYMPTOMS ARE RELATED WITH PF MALARIA INFECTION, TYPICALLY.									
		3 mg/kg	1	HYPOCALCEMIA	Hypocalcaemia	Metabolism and nutrition disorders	LAB RESULT ON 19-DEC-2011, SERUM Ca2+ 6.7 mmol/L, NO TETANY, NO QTC PROLONGATION. HE GOT 10% CALCIUM GLUCONATE 10 ML IV SLOWLY PUSH AT 22.30 PM.			Recovered	Mild	Possibly related	Possibly related	Concurrent illness	MALARIA PF INFECTION	Treatment Medicatio
		3 mg/kg	2	APTT PROLONGATION	Activated partial thromboplastin time prolonged	Investigations	THIS PATIENT HAD HYPOCALCEMIA SO HE GOT 10% CALCIUM			Recovered	Mild	Possibly related	Possibly related	Concomit medication	10% CALCIUM GLUCONATE	None

SUBJ ID	INITIAL	ARM	AE SEQ	AETERM	AEDECOD	AESOC	DESCRIPTION	ONSET DATE TIME *99:99=UNK*	END DATE	OUTCOME	INTENS	RELAT. DF02	RELAT. MALANIL	ALT RELAT	ALTRELOTHER	ACTION
							GLUCONATE AT 22:30. WE ALSO INVESTIGATED THE COAGULATION TEST AT 30H, THE RESULT WAS INVALID DUE TO PROLONG APTT. HIS CLINICAL WAS FINE (NO ANY AE), THE TEST WAS DONE FOLLOW THE PROTOCOL.									
		3 mg/kg	3	ALT INCREASED	Alanine aminotransferase increased	Investigations	THE FOLLOW LABORATORY AT DAY 7 IS ABNORMAL. AST=53, ALT=88, NO CLINICAL OF HEPATITIS, DENIED ALCOHOL DRINKING OR HERBAL USE.			Not recovered	Mild	Possibly related	Possibly related	Concurrent illness	MALARIA PF INFECTION	Other treatmen
		3	4	ALT	Alanine	Investigati	THE			Not	Mild	Possibly	Possibly	Concurrent	MALARIA	Other

SUBJ ID	INITIAL	ARM	AE SEQ	AETERM	AEDECOD	AESOC	DESCRIPTION	ONSET DATE TIME *99:99=UNK*	END DATE	OUTCOME	INTENS	RELAT. DF02	RELAT. MALANIL	ALT RELAT	ALTRELOTHER	ACTION
		mg/kg		INCREASED	aminotransferase increased	ons	LABORATORY AT DAY 14 IS ABNORMAL. AST=46, ALT=75 NO CLINICAL OF HEPATITIS, DENIED ALCOHOL DRINKING OR HERBAL USE.			recovered		related	related	illness	PF INFECTION	treatmen
		3 mg/kg	1	ANEMIA (HEMOGLOBINOPATHY)	Haemoglobinopathy	Congenital, familial and genetic disorders	ON D2, HCT 19.7%, HB 6.6G/DL.PERIPHERAL BLOOD SMEAR SHOWS AN ISOPOIKILOCYTOSIS +3, POSSIBLE HEMOGLOBINOPATHY. NO CLINICAL ANEMIC SYMPTOM OR HEART FAILURE. HE DENIED TO RECEIVE BLOOD TRANSFUSION.			Recovered	Severe	Not related	Not related	Concurrent illness	MALARIA PF INFECTION	Treatment Medicatio

SUBJ ID	INITIAL	ARM	AE SEQ	AETERM	AEDECOD	AESOC	DESCRIPTION	ONSET DATE TIME 99:99=UNK	END DATE	OUTCOME	INTENS	RELAT. DF02	RELAT. MALANIL	ALT RELAT	ALTRELOTHER	ACTION
		3 mg/kg	2	MILD HYPOCALCEMIA	Hypocalcaemia	Metabolism and nutrition disorders	SERUM Ca2+ =7.5 mmol/L, BUT NO CLINICAL SIGNIFICANT. HE GOT CALCIUM CARBONATE ORALLY.			Recovered	Moderate	Possibly related	Possibly related	Concurrent illness	MALARIA PF INFECTION	Treatment Medicatio
		3 mg/kg	1	VOMITING	Vomiting	Gastrointestinal disorders	HE HAD A FEVER WITH 1 TIME OF VOMITING IN THE EVENING, SO DOMPERIDON WAS GIVEN			Recovered	Mild	Not related	Possibly related	Concurrent illness	UNCOMPLICATED PF MALARIA	Treatment Medicatio
		3 mg/kg	2	THROMBOCYTOPENIA	Thrombocytopenia	Blood and lymphatic system disorders	LAB RESULT: PLATELET 66,000 NO CLINICAL BLEEDING, NO PETECHIAE			Recovered	Moderate	Not related	Not related	Concurrent illness	MALARIA PF INFECTION	None
		3 mg/kg	3	ALT INCREASED	Alanine aminotransferase increased	Investigations	HE HAS ABNORMAL LABORATORY RESULT IN DAY 7 FOLLOW UP. AST = 87, ACT = 104 NO CLINICAL OF			Not recovered	Mild	Possibly related	Possibly related	Concurrent illness	MALARIA PF INFECTION	None

SUBJ ID	INITIAL	ARM	AE SEQ	AETERM	AEDECOD	AESOC	DESCRIPTION	ONSET DATE TIME *99:99=UNK*	END DATE	OUTCOME	INTENS	RELAT. DF02	RELAT. MALANIL	ALT RELAT	ALTRELOTHER	ACTION
							HEPATITIS, DENIED ALCOHOL DRINKING OR HERBAL USE									
		3 mg/kg	4	ALT INCREASED	Alanine aminotransferase increased	Investigations	DAY 14 FOLLOW UP AST = 24, ACT = 54. (DECREASE FROM DAY 7 RESULT), NO CLINICAL HEPATITIS, FROM DATA OF 5 PATIENT, SUGGEST TRANSIENT ELEVATE OF ACT			Recovered	Mild	Possibly related	Possibly related	Concurrent illness	MALARIA PF INFECTION	None
		6 mg/kg	1	INCREASED SGOT	Aspartate aminotransferase increased	Investigations	THE BLOOD RESULT FOR SGOT, SGPT WERE INCREASED BUT THEY WERE WITHIN 2 FOLD OF NORMAL RANGE. HE DENIED FOR HISTORY OF			Recovered	Mild	Possibly related	Possibly related	Other	UNKNOWN	None

SUBJ ID	INITIAL	ARM	AE SEQ	AETERM	AEDECOD	AESOC	DESCRIPTION	ONSET DATE TIME *99:99=UNK*	END DATE	OUTCOME	INTENS	RELAT. DF02	RELAT. MALANIL	ALT RELAT	ALTRELOTHER	ACTION
							ALCOHOL DRINKING OR HERBAL MEDICINE. DAY 14 OF STUDY, THE SGOT AND SGPT RESULT WERE NORMAL.									
		6 mg/kg	2	INCREASED SGPT	Alanine aminotransferase increased	Investigations	THE BLOOD RESULT FOR SGOT, SGPT WERE INCREASED BUT THEY WERE WITHIN 2 FOLD OF NORMAL RANGE. HE DENIED FOR HISTORY OF ALCOHOL DRINKING OR HERBAL MEDICINE. DAY14 OF STUDY, THE SGOT AND SGPT RESULT WERE NORMAL.			Recovered	Mild	Possibly related	Possibly related	Other	UNKNOWN	None

SUBJ ID	INITIAL	ARM	AE SEQ	AETERM	AEDECOD	AESOC	DESCRIPTION	ONSET DATE TIME *99:99=UNK*	END DATE	OUTCOME	INTENS	RELAT. DF02	RELAT. MALANIL	ALT RELAT	ALTRELOTHER	ACTION
		6 mg/kg	1	HYPER K+	Hyperkalaemia	Metabolism and nutrition disorders	THE BLOOD SAMPLE WAS STORED OVERNIGHT, SO THE RESULT OF E'LYTE WAS ERROR. IT HAD AN EFFECT ON POTASSIUM, SO K+ LEVEL WAS HIGHT (6.2) WITHOUT ANY SPECIFIC SIGN AND SYMPTOMS. THE BLOOD WAS INVESTIGATED AGAIN IN THE AFTERNOON, K+ LEVEL WAS NORMAL RANGE			Recovered	Mild	Not related	Not related	Concurrent illness	UNCOMPLICATED MALARIA	Other treatmen
		6 mg/kg	2	INCREASED SGOT	Aspartate aminotransferase increased	Investigations	SGOT AND SGPT RESULTS WERE INCREASED DURING THE FOLLOW UP ON DAY 7. HE			Recovered	Moderate	Possibly related	Possibly related	Concurrent illness	UNCOMPLICATED PF MALARIA	None

SUBJ ID	INITIAL	ARM	AE SEQ	AETERM	AEDECOD	AESOC	DESCRIPTION	ONSET DATE TIME *99:99=UNK*	END DATE	OUTCOME	INTENS	RELAT. DF02	RELAT. MALANIL	ALT RELAT	ALTRELOTHER	ACTION
							HAD NO SIGN OF JAUNDICE AND DENIED FOR HISTORY OF ALCOHOL DRINKING OR HERBAL MEDICINE. ON 30 APR 12, THE RESULT WAS DECREASED TO NORMAL RANGE									
		6 mg/kg	3	INCREASED SGPT	Alanine aminotransferase increased	Investigations	SGOT AND SGPT RESULTS WERE INCREASED DURING THE FOLLOW UP ON DAY 7. HE HAD NO SIGN OF JAUNDICE AND DENIED FOR HISTORY OF ALCOHOL DRINKING OR HERBAL MEDICINE. ON 30APR12, THE RESULT WAS DECREASED TO NORMAL			Recovered	Moderate	Possibly related	Possibly related	Concurrent illness	UNCOMPLICATED PF MALARIA	None

SUBJ ID	INITIAL	ARM	AE SEQ	AETERM	AEDECOD	AESOC	DESCRIPTION	ONSET DATE TIME *99:99=UNK*	END DATE	OUTCOME	INTENS	RELAT. DF02	RELAT. MALANIL	ALT RELAT	ALTRELOTHER	ACTION
							RANGE.									
		6 mg/kg	1	DIZZINESS	Dizziness	Nervous system disorders	SHE HAS DIZZINESS IN THE MORNING ON 20 APR 2012. SHE HAD NO NAUSEA OR VOMITING			Recovered	Mild	Not related	Possibly related	Concurrent illness	UNCOMPLICATED MALARIA	None
		6 mg/kg	2	ANEMIA	Anaemia	Blood and lymphatic system disorders	SHE HAD ANEMIA. HEMOGLOBIN DROP FROM SCREENING DAY. NO ANEMIC SYMPTOM			Recovered	Severe	Not related	Not related	Concurrent illness	UNCOMPLICATED PF MALARIA	Treatmen Medicatic
		6 mg/kg	3	INCREASED SGOT	Aspartate aminotransferase increased	Investigations	THE RESULT OF SGOT& SGPT WERE INCREASED BUT LESS THAN 2.5 FOLD. SHE HAD NO HISTORY OF ALCOHOLIC DRINKING OR USING HERBAL MEDICINE. SHE HAD NO JAUNDICE			Recovered	Mild	Not related	Not related	Concurrent illness	UNCOMPLICATED PF MALARIA	None

SUBJ ID	INITIAL	ARM	AE SEQ	AETERM	AEDECOD	AESOC	DESCRIPTION	ONSET DATE TIME *99:99=UNK*	END DATE	OUTCOME	INTENS	RELAT. DF02	RELAT. MALANIL	ALT RELAT	ALTRELOTHER	ACTION
							TOO.									
		6 mg/kg	4	INCREASED SGPT	Alanine aminotransferase increased	Investigations	THE RESULT OF SGOT & SGPT WERE INCREASED BUT LESS THAN 2.5 FOLD. SHE HAD NO HISTORY OF ALCOHOL DRINKING OR USING HERBAL MEDICINE. SHE HAD NO JAUNDICE TOO.			Recovered	Mild	Not related	Not related	Concurrent illness	UNCOMPLICATED PF MALARIA	None

16.2.7.2 Serious adverse event listing

Not applicable.

16.2.7.3 Physical examination (including weight and height)

SUBJID	INITIAL	ARM	DAY	HGT cm	WGT kg	GCSEYE	GCSVERBAL	GCSMOTOR	GCSTOTAL
		1.5 mg/kg	1	168	67.9	4	5	6	15
		1.5 mg/kg	2	NA	NA	4	5	6	
		1.5 mg/kg	3	NA	NA	4	5	6	
		1.5 mg/kg	4	NA	68.5	4	5	6	
		1.5 mg/kg	5	NA	69	4	5	6	
		1.5 mg/kg	7	NA	66	4	5	6	
		1.5 mg/kg	14	NA	65.7	4	5	6	
		1.5 mg/kg	1	160	53.5	4	5	6	15
		1.5 mg/kg	2	NA	NA	4	5	6	
		1.5 mg/kg	3	NA	NA	4	5	6	
		1.5 mg/kg	4	NA	55.7	4	5	6	
		1.5 mg/kg	5	NA	56.4	4	5	6	
		1.5 mg/kg	7	NA	55.7	4	5	6	
		1.5 mg/kg	14	NA	54.3	4	5	6	
		1.5 mg/kg	1	162	52.1	4	5	6	15
		1.5 mg/kg	2	NA	NA	4	5	6	
		1.5 mg/kg	3	NA	NA	4	5	6	
		1.5 mg/kg	4	NA	51.7	4	5	6	
		1.5 mg/kg	7	NA	50	4	5	6	
		1.5 mg/kg	14	NA	50	4	5	6	
		3 mg/kg	1	163	50	4	5	6	15

SUBJID	INITIAL	ARM	DAY	HGT cm	WGT kg	GCSEYE	GCSVERBAL	GCSMOTOR	GCSTOTAL
		3 mg/kg	2	NA	NA	4	5	6	
		3 mg/kg	3	NA	NA	4	5	6	
		3 mg/kg	4	NA	49	4	5	6	
		3 mg/kg	7	NA	49	4	5	6	
		3 mg/kg	14	NA	49	4	5	6	
		3 mg/kg	1	165	47.2	4	5	6	15
		3 mg/kg	2	NA	NA	4	5	6	
		3 mg/kg	3	NA	NA	4	5	6	
		3 mg/kg	4	NA	45.2	4	5	6	
		3 mg/kg	7	NA	45.5	4	5	6	
		3 mg/kg	14	NA	47	4	5	6	
		3 mg/kg	1	165	49.3	4	5	6	15
		3 mg/kg	2	NA	NA	4	5	6	
		3 mg/kg	3	NA	NA	4	5	6	
		3 mg/kg	4	NA	48.5	4	5	6	
		3 mg/kg	7	NA	49	4	5	6	
		3 mg/kg	14	NA	49	4	5	6	
		6 mg/kg	1	175	62.9	4	5	6	15
		6 mg/kg	2	NA	NA	4	5	6	
		6 mg/kg	3	NA	NA	4	5	6	
		6 mg/kg	4	NA	62.2	4	5	6	
		6 mg/kg	7	NA	62.9	4	5	6	
		6 mg/kg	14	NA	62.9	4	5	6	

SUBJID	INITIAL	ARM	DAY	HGT cm	WGT kg	GCSEYE	GCSVERBAL	GCSMOTOR	GCSTOTAL
		6 mg/kg	1	157	51	4	5	6	15
		6 mg/kg	2	NA	NA	4	5	6	
		6 mg/kg	3	NA	NA	4	5	6	
		6 mg/kg	4	NA	50.8	4	5	6	
		6 mg/kg	7	NA	51	4	5	6	
		6 mg/kg	14	NA	51	4	5	6	
		6 mg/kg	1	155	51.1	4	5	6	15
		6 mg/kg	2	NA	NA	4	5	6	
		6 mg/kg	3	NA	NA	4	5	6	
		6 mg/kg	4	NA	52.1	4	5	6	
		6 mg/kg	7	NA	51	4	5	6	
		6 mg/kg	14	NA	52	4	5	6	

16.2.7.3 Physical examination (including weight and height) (-continued)

SUBJID	INITIAL	ARM	DAY	EARS	EYES	NOSE	MOUTH	THROAT	HEENT
		1.5 mg/kg	1	Normal	Normal	Normal	Normal	Normal	NA
		1.5 mg/kg	2	NA	NA	NA	NA	NA	No
		1.5 mg/kg	3	NA	NA	NA	NA	NA	No
		1.5 mg/kg	4	NA	NA	NA	NA	NA	No
		1.5 mg/kg	5	NA	NA	NA	NA	NA	No
		1.5 mg/kg	7	NA	NA	NA	NA	NA	No
		1.5 mg/kg	14	NA	NA	NA	NA	NA	No
		1.5 mg/kg	1	Normal	Normal	Normal	Normal	Normal	NA
		1.5 mg/kg	2	NA	NA	NA	NA	NA	No
		1.5 mg/kg	3	NA	NA	NA	NA	NA	No
		1.5 mg/kg	4	NA	NA	NA	NA	NA	No
		1.5 mg/kg	5	NA	NA	NA	NA	NA	No
		1.5 mg/kg	7	NA	NA	NA	NA	NA	No
		1.5 mg/kg	14	NA	NA	NA	NA	NA	No
		1.5 mg/kg	1	Normal	Normal	Normal	Normal	Normal	NA
		1.5 mg/kg	2	NA	NA	NA	NA	NA	No
		1.5 mg/kg	3	NA	NA	NA	NA	NA	No
		1.5 mg/kg	4	NA	NA	NA	NA	NA	No
		1.5 mg/kg	7	NA	NA	NA	NA	NA	No
		1.5 mg/kg	14	NA	NA	NA	NA	NA	No
		3 mg/kg	1	Normal	Normal	Normal	Normal	Normal	NA
		3 mg/kg	2	NA	NA	NA	NA	NA	No

SUBJID	INITIAL	ARM	DAY	EARS	EYES	NOSE	MOUTH	THROAT	HEENT
		3 mg/kg	3	NA	NA	NA	NA	NA	No
		3 mg/kg	4	NA	NA	NA	NA	NA	No
		3 mg/kg	7	NA	NA	NA	NA	NA	No
		3 mg/kg	14	NA	NA	NA	NA	NA	No
		3 mg/kg	1	Normal	Normal	Normal	Normal	Normal	NA
		3 mg/kg	2	NA	NA	NA	NA	NA	No
		3 mg/kg	3	NA	NA	NA	NA	NA	No
		3 mg/kg	4	NA	NA	NA	NA	NA	No
		3 mg/kg	7	NA	NA	NA	NA	NA	No
		3 mg/kg	14	NA	NA	NA	NA	NA	No
		3 mg/kg	1	Normal	Normal	Normal	Normal	Normal	NA
		3 mg/kg	2	NA	NA	NA	NA	NA	No
		3 mg/kg	3	NA	NA	NA	NA	NA	No
		3 mg/kg	4	NA	NA	NA	NA	NA	No
		3 mg/kg	7	NA	NA	NA	NA	NA	No
		3 mg/kg	14	NA	NA	NA	NA	NA	No
		6 mg/kg	1	Normal	Normal	Normal	Normal	Normal	NA
		6 mg/kg	2	NA	NA	NA	NA	NA	No
		6 mg/kg	3	NA	NA	NA	NA	NA	No
		6 mg/kg	4	NA	NA	NA	NA	NA	No
		6 mg/kg	7	NA	NA	NA	NA	NA	No
		6 mg/kg	14	NA	NA	NA	NA	NA	No
		6 mg/kg	1	Normal	Normal	Normal	Normal	Normal	NA

SUBJID	INITIAL	ARM	DAY	EARS	EYES	NOSE	MOUTH	THROAT	HEENT
		6 mg/kg	2	NA	NA	NA	NA	NA	No
		6 mg/kg	3	NA	NA	NA	NA	NA	No
		6 mg/kg	4	NA	NA	NA	NA	NA	No
		6 mg/kg	7	NA	NA	NA	NA	NA	No
		6 mg/kg	14	NA	NA	NA	NA	NA	No
		6 mg/kg	1	Normal	Normal	Normal	Normal	Normal	NA
		6 mg/kg	2	NA	NA	NA	NA	NA	No
		6 mg/kg	3	NA	NA	NA	NA	NA	No
		6 mg/kg	4	NA	NA	NA	NA	NA	No
		6 mg/kg	7	NA	NA	NA	NA	NA	No
		6 mg/kg	14	NA	NA	NA	NA	NA	No

16.2.7.3 Physical examination (including weight and height) (-continued)

SUBJID	INITIAL	ARM	DAY	HEPATOMEGALY	SPLENOMEGALY	CARDIO	RESPIRATORY	GASTRO	SKIN	JOINTS	CNS
		1.5 mg/kg	1	No	No	Normal	Normal	Normal	Normal	Normal	Normal
		1.5 mg/kg	2	No	No	No	No	No	No	No	No
		1.5 mg/kg	3	No	No	No	No	No	No	No	No
		1.5 mg/kg	4	No	No	No	No	No	No	No	No
		1.5 mg/kg	5	No	No	No	No	No	No	No	No
		1.5 mg/kg	7	No	No	No	No	No	No	No	No
		1.5 mg/kg	14	No	No	No	No	No	No	No	No
		1.5 mg/kg	1	No	No	Normal	Normal	Normal	Normal	Normal	Normal
		1.5 mg/kg	2	No	No	No	No	No	No	No	No
		1.5 mg/kg	3	No	No	No	No	No	No	No	No
		1.5 mg/kg	4	No	No	No	No	No	No	No	No
		1.5 mg/kg	5	No	No	No	No	No	No	No	No
		1.5 mg/kg	7	No	No	No	No	No	No	No	No
		1.5 mg/kg	14	No	No	No	No	No	No	No	No
		1.5 mg/kg	1	No	No	Normal	Normal	Normal	Normal	Normal	Normal
		1.5 mg/kg	2	No	No	No	No	No	No	No	No
		1.5 mg/kg	3	No	No	No	No	No	No	No	No
		1.5 mg/kg	4	No	No	No	No	No	No	No	No
		1.5 mg/kg	7	No	No	No	No	No	No	No	No
		1.5 mg/kg	14	No	No	No	No	No	No	No	No
		3 mg/kg	1	No	No	Normal	Normal	Normal	Normal	Normal	Normal
		3 mg/kg	2	No	No	No	No	No	No	No	No

SUBJID	INITIAL	ARM	DAY	HEPATOMEGALY	SPLENOMEGALY	CARDIO	RESPIRATORY	GASTRO	SKIN	JOINTS	CNS
		3 mg/kg	3	No	No	No	No	No	No	No	No
		3 mg/kg	4	No	No	No	No	No	No	No	No
		3 mg/kg	7	No	No	No	No	No	No	No	No
		3 mg/kg	14	No	No	No	No	No	No	No	No
		3 mg/kg	1	No	No	Normal	Normal	Normal	Normal	Normal	Normal
		3 mg/kg	2	No	No	No	No	No	No	No	No
		3 mg/kg	3	No	No	No	No	No	No	No	No
		3 mg/kg	4	No	No	No	No	No	No	No	No
		3 mg/kg	7	No	No	No	No	No	No	No	No
		3 mg/kg	14	No	No	No	No	No	No	No	No
		3 mg/kg	1	No	No	Normal	Normal	Normal	Normal	Normal	Normal
		3 mg/kg	2	No	No	No	No	No	No	No	No
		3 mg/kg	3	No	No	No	No	No	No	No	No
		3 mg/kg	4	No	No	No	No	No	No	No	No
		3 mg/kg	7	No	No	No	No	No	No	No	No
		3 mg/kg	14	No	No	No	No	No	No	No	No
		6 mg/kg	1	No	No	Normal	Normal	Normal	Normal	Normal	Normal
		6 mg/kg	2	No	No	No	No	No	No	No	No
		6 mg/kg	3	No	No	No	No	No	No	No	No
		6 mg/kg	4	No	No	No	No	No	No	No	No
		6 mg/kg	7	No	No	No	No	No	No	No	No
		6 mg/kg	14	No	No	No	No	No	No	No	No
		6 mg/kg	1	No	No	Normal	Normal	Normal	Not available	Not available	Normal

SUBJID	INITIAL	ARM	DAY	HEPATOMEGALY	SPLENOMEGALY	CARDIO	RESPIRATORY	GASTRO	SKIN	JOINTS	CNS
		6 mg/kg	2	No	No	No	No	No	No	No	No
		6 mg/kg	3	No	No	No	No	No	No	No	No
		6 mg/kg	4	No	No	No	No	No	No	No	No
		6 mg/kg	7	No	No	No	No	No	No	No	No
		6 mg/kg	14	No	No	No	No	No	No	No	No
		6 mg/kg	1	No	No	Normal	Normal	Normal	Normal	Normal	Normal
		6 mg/kg	2	No	No	No	No	No	No	No	No
		6 mg/kg	3	No	No	No	No	No	No	No	No
		6 mg/kg	4	No	No	No	No	No	No	No	No
		6 mg/kg	7	No	No	No	No	No	No	No	No
		6 mg/kg	14	No	No	No	No	No	No	No	No

16.2.7.4 Vital signs

SUBJID	INITIAL	ARM	TIMEPOINT	TEMP (Celsius) *99=Not available*	HR (bpm) *999=Not available*	RR (bpm) *99=Not available*	BP (mmHg) *999/999= Not available*
		1.5 mg/kg	screening	37.8	94	24	116/079
		1.5 mg/kg	D1H0	38.8	98	24	113/083
		1.5 mg/kg	D1H0:30	38.7	103	24	109/071
		1.5 mg/kg	D1H1	38.3	94	24	110/069
		1.5 mg/kg	D1H1:30	38.1	96	24	107/068
		1.5 mg/kg	D1H2	37.8	99	22	113/075
		1.5 mg/kg	D1H3	37.7	999	99	999/999
		1.5 mg/kg	D1H4	37.8	999	99	999/999
		1.5 mg/kg	D1H6	37.1	93	22	112/076
		1.5 mg/kg	D1H8	38.7	98	20	107/067
		1.5 mg/kg	D1H10	39.2	999	99	999/999
		1.5 mg/kg	D1H12	38	98	22	106/073
		1.5 mg/kg	D1H14	37.6	92	20	110/072
		1.5 mg/kg	D1H18	38.9	101	24	109/067
		1.5 mg/kg	D1H20	38.4	97	24	108/073
		1.5 mg/kg	D2H24	39	108	24	113/071
		1.5 mg/kg	D2H30	39.4	108	24	114/075
		1.5 mg/kg	D2H36	39.2	108	24	120/080
		1.5 mg/kg	D2H42	38.8	96	24	107/073
		1.5 mg/kg	D3H48	39.5	110	24	101/070
		1.5 mg/kg	D3H54	38.5	95	22	109/070
		1.5 mg/kg	D3H60	38.2	88	20	099/056
		1.5 mg/kg	D3H66	36.9	79	20	107/068
		1.5 mg/kg	D4H72	37.2	81	20	112/072
		1.5 mg/kg	D5	36.8	90	18	100/060
		1.5 mg/kg	D7	36.5	87	16	105/062
		1.5 mg/kg	D14	36.7	96	18	123/086
		1.5 mg/kg	screening	39.6	114	22	092/059
		1.5 mg/kg	D1H0	37.1	82	20	096/058
		1.5 mg/kg	D1H0:30	37.3	90	20	097/060
		1.5 mg/kg	D1H1	37.5	95	20	104/068
		1.5 mg/kg	D1H1:30	36.9	90	20	102/069
		1.5 mg/kg	D1H2	37.8	88	20	095/060
		1.5 mg/kg	D1H3	37.8	999	99	999/999
		1.5 mg/kg	D1H4	37.7	999	99	999/999

SUBJID	INITIAL	ARM	TIMEPOINT	TEMP (Celsius) *99=Not available*	HR (bpm) *999=Not available*	RR (bpm) *99=Not available*	BP (mmHg) *999/999= Not available*
		1.5 mg/kg	D1H6	38	87	20	104/066
		1.5 mg/kg	D1H8	36.1	77	18	082/056
		1.5 mg/kg	D1H10	36	999	99	999/999
		1.5 mg/kg	D1H12	36.2	72	20	085/058
		1.5 mg/kg	D1H14	36.8	84	20	094/066
		1.5 mg/kg	D1H18	36	76	20	095/059
		1.5 mg/kg	D1H20	36.7	999	99	999/999
		1.5 mg/kg	D2H24	36.7	77	22	092/063
		1.5 mg/kg	D2H30	38.9	96	20	102/062
		1.5 mg/kg	D2H36	37.9	88	20	097/065
		1.5 mg/kg	D2H42	37.1	84	18	099/069
		1.5 mg/kg	D3H48	37	80	20	106/066
		1.5 mg/kg	D3H54	36.9	80	20	104/073
		1.5 mg/kg	D3H60	37.2	74	20	095/056
		1.5 mg/kg	D3H66	36.7	77	20	101/067
		1.5 mg/kg	D4H72	37.4	81	20	103/068
		1.5 mg/kg	D7	36.4	66	18	121/073
		1.5 mg/kg	D14	36.6	89	18	108/076
		1.5 mg/kg	screening	39	79	18	117/064
		1.5 mg/kg	D1H0	38.4	84	18	093/057
		1.5 mg/kg	D1H0:30	38.5	84	18	117/067
		1.5 mg/kg	D1H1	38.7	84	20	119/067
		1.5 mg/kg	D1H1:30	38.8	81	20	118/070
		1.5 mg/kg	D1H2	38.6	76	20	115/062
		1.5 mg/kg	D1H3	37.7	999	99	999/999
		1.5 mg/kg	D1H4	37.3	999	99	999/999
		1.5 mg/kg	D1H6	37	63	18	109/071
		1.5 mg/kg	D1H8	37.1	72	20	120/080
		1.5 mg/kg	D1H10	36.2	999	99	999/999
		1.5 mg/kg	D1H12	36	54	18	121/083
		1.5 mg/kg	D1H14	36	60	18	114/071
		1.5 mg/kg	D1H18	36.4	56	18	119/075
		1.5 mg/kg	D1H20	37.4	63	18	124/082
		1.5 mg/kg	D2H24	37.5	63	18	120/067
		1.5 mg/kg	D2H30	37.3	69	18	117/074
		1.5 mg/kg	D2H36	37.2	61	18	114/066

SUBJID	INITIAL	ARM	TIMEPOINT	TEMP (Celsius) *99=Not available*	HR (bpm) *999=Not available*	RR (bpm) *99=Not available*	BP (mmHg) *999/999= Not available*
		1.5 mg/kg	D2H42	36.4	65	18	117/089
		1.5 mg/kg	D3H48	37.5	69	18	136/082
		1.5 mg/kg	D3H54	36.9	61	18	106/069
		1.5 mg/kg	D3H60	37	53	18	123/075
		1.5 mg/kg	D3H66	36.2	58	18	124/069
		1.5 mg/kg	D4H72	36.7	54	18	131/083
		1.5 mg/kg	D7	36.2	73	16	105/062
		1.5 mg/kg	D14	36.4	72	16	119/075
		3 mg/kg	screening	36.7	82	16	123/084
		3 mg/kg	D1H0	37.6	78	18	110/065
		3 mg/kg	D1H0:30	36.9	82	20	108/070
		3 mg/kg	D1H1	36.5	92	20	110/083
		3 mg/kg	D1H1:30	36.8	92	20	111/072
		3 mg/kg	D1H2	36.6	83	18	109/070
		3 mg/kg	D1H3	36.7	999	99	999/999
		3 mg/kg	D1H4	36.9	90	18	103/074
		3 mg/kg	D1H6	37.2	89	18	107/062
		3 mg/kg	D1H8	36.6	999	99	999/999
		3 mg/kg	D1H10	37.1	999	99	999/999
		3 mg/kg	D1H12	38.5	93	20	111/057
		3 mg/kg	D1H14	36.4	73	20	110/065
		3 mg/kg	D1H18	36.8	75	18	108/062
		3 mg/kg	D1H20	37.4	80	20	106/062
		3 mg/kg	D2H24	38.1	94	20	110/070
		3 mg/kg	D2H30	36.1	66	18	128/080
		3 mg/kg	D2H36	36.3	71	18	111/067
		3 mg/kg	D2H42	36.3	70	18	119/070
		3 mg/kg	D3H48	36.8	68	18	120/071
		3 mg/kg	D3H54	36	62	18	101/066
		3 mg/kg	D3H60	36.2	65	18	107/072
		3 mg/kg	D3H66	36.5	63	18	111/068
		3 mg/kg	D4H72	36.2	72	18	115/067
		3 mg/kg	D7	36.4	75	16	147/089
		3 mg/kg	D14	36.6	80	16	135/098
		3 mg/kg	screening	39.2	84	16	098/061
		3 mg/kg	D1H0	37.4	90	18	090/050

SUBJID	INITIAL	ARM	TIMEPOINT	TEMP (Celsius) *99=Not available*	HR (bpm) *999=Not available*	RR (bpm) *99=Not available*	BP (mmHg) *999/999= Not available*
		3 mg/kg	D1H0:30	37.1	76	18	102/050
		3 mg/kg	D1H1	37.1	78	18	098/051
		3 mg/kg	D1H1:30	37.3	75	18	101/054
		3 mg/kg	D1H2	37.4	76	18	096/050
		3 mg/kg	D1H3	37.6	999	99	999/999
		3 mg/kg	D1H4	38.9	87	16	089/050
		3 mg/kg	D1H6	38.8	96	18	093/051
		3 mg/kg	D1H8	39.1	999	99	999/999
		3 mg/kg	D1H10	37.7	999	99	999/999
		3 mg/kg	D1H12	35.9	57	18	090/056
		3 mg/kg	D1H14	36.5	76	18	096/055
		3 mg/kg	D1H18	36.1	62	18	085/046
		3 mg/kg	D1H20	37	74	16	089/050
		3 mg/kg	D2H24	36.6	70	18	093/051
		3 mg/kg	D2H30	37.1	83	18	090/053
		3 mg/kg	D2H36	36.1	61	18	089/054
		3 mg/kg	D2H42	36.8	78	18	094/051
		3 mg/kg	D3H48	37.3	84	18	093/051
		3 mg/kg	D3H54	36.5	73	18	089/050
		3 mg/kg	D3H60	36.3	82	18	089/049
		3 mg/kg	D3H66	36.1	81	18	090/050
		3 mg/kg	D4H72	36.2	78	18	090/045
		3 mg/kg	D7	36.2	91	18	096/063
		3 mg/kg	D14	36.7	93	16	106/063
		3 mg/kg	screening	37.2	58	16	104/060
		3 mg/kg	D1H0	37.3	82	18	092/055
		3 mg/kg	D1H0:30	38	82	18	108/066
		3 mg/kg	D1H1	38.4	86	18	116/067
		3 mg/kg	D1H1:30	38.3	84	18	112/052
		3 mg/kg	D1H2	39.3	84	18	112/062
		3 mg/kg	D1H3	38.8	86	18	113/065
		3 mg/kg	D1H4	38.3	85	18	100/051
		3 mg/kg	D1H6	36.5	80	18	120/060
		3 mg/kg	D1H8	36.3	82	18	101/063
		3 mg/kg	D1H10	36.7	70	18	098/056
		3 mg/kg	D1H12	37.3	72	18	110/054

SUBJID	INITIAL	ARM	TIMEPOINT	TEMP (Celsius)<br>*99=Not available*	HR (bpm)<br>*999=Not available*	RR (bpm)<br>*99=Not available*	BP (mmHg)<br>*999/999= Not available*
		3 mg/kg	D1H14	38.8	85	18	100/052
		3 mg/kg	D1H18	37.2	77	18	107/063
		3 mg/kg	D1H20	37.3	90	18	111/067
		3 mg/kg	D2H24	38.7	91	20	110/063
		3 mg/kg	D2H30	36.6	77	18	104/066
		3 mg/kg	D2H36	37.3	77	18	104/061
		3 mg/kg	D2H42	36.4	70	18	091/062
		3 mg/kg	D3H48	36.7	80	18	102/072
		3 mg/kg	D3H54	36.4	71	18	099/058
		3 mg/kg	D3H60	36.3	76	18	102/064
		3 mg/kg	D3H66	36.2	65	18	098/059
		3 mg/kg	D4H72	36.7	76	18	105/065
		3 mg/kg	D7	36.7	76	16	108/067
		3 mg/kg	D14	36.6	74	16	114/071
		6 mg/kg	screening	36.6	53	20	111/065
		6 mg/kg	D1H0	36.6	53	20	111/065
		6 mg/kg	D1H0:30	37	59	20	108/061
		6 mg/kg	D1H1	37	69	20	110/061
		6 mg/kg	D1H1:30	37.3	80	18	105/065
		6 mg/kg	D1H2	38.1	75	20	106/053
		6 mg/kg	D1H3	37.8	75	18	105/060
		6 mg/kg	D1H4	37.6	70	18	107/063
		6 mg/kg	D1H6	36.9	56	18	107/055
		6 mg/kg	D1H8	37	60	18	110/080
		6 mg/kg	D1H10	36.9	72	18	106/076
		6 mg/kg	D1H12	36.2	52	18	096/056
		6 mg/kg	D1H14	36.8	61	18	101/068
		6 mg/kg	D1H18	38.4	71	20	121/063
		6 mg/kg	D1H20	37.3	65	20	111/071
		6 mg/kg	D2H24	37.5	61	20	101/064
		6 mg/kg	D2H30	37.1	52	18	102/061
		6 mg/kg	D2H36	36.6	56	18	101/057
		6 mg/kg	D2H42	36.7	55	18	102/058
		6 mg/kg	D3H48	36.6	54	18	105/061
		6 mg/kg	D3H54	37	53	18	106/067
		6 mg/kg	D3H60	35.8	45	18	098/059

SUBJID	INITIAL	ARM	TIMEPOINT	TEMP (Celsius) *99=Not available*	HR (bpm) *999=Not available*	RR (bpm) *99=Not available*	BP (mmHg) *999/999= Not available*
		6 mg/kg	D3H66	36.3	56	18	089/052
		6 mg/kg	D4H72	36.3	61	18	092/060
		6 mg/kg	D7	36.3	67	16	102/062
		6 mg/kg	D14	36.1	64	16	121/073
		6 mg/kg	screening	37.4	67	16	103/059
		6 mg/kg	D1H0	37.4	93	18	094/053
		6 mg/kg	D1H0:30	37.2	89	18	093/056
		6 mg/kg	D1H1	37.3	89	18	092/053
		6 mg/kg	D1H1:30	37.3	89	20	091/058
		6 mg/kg	D1H2	37.4	89	20	088/059
		6 mg/kg	D1H3	37.3	85	18	086/055
		6 mg/kg	D1H4	37	82	18	086/057
		6 mg/kg	D1H6	36.7	91	18	099/054
		6 mg/kg	D1H8	37.1	84	18	096/064
		6 mg/kg	D1H10	37.2	81	18	089/050
		6 mg/kg	D1H12	37	71	18	092/056
		6 mg/kg	D1H14	37.1	80	18	087/055
		6 mg/kg	D1H18	36.3	67	18	089/053
		6 mg/kg	D1H20	36.7	71	18	091/061
		6 mg/kg	D2H24	36.9	78	18	097/055
		6 mg/kg	D2H30	36.9	73	18	112/056
		6 mg/kg	D2H36	36.6	71	18	096/054
		6 mg/kg	D2H42	36.9	68	18	096/052
		6 mg/kg	D3H48	36.8	75	18	101/056
		6 mg/kg	D3H54	36.4	71	18	095/054
		6 mg/kg	D3H60	36.6	78	18	098/050
		6 mg/kg	D3H66	35.6	61	18	083/050
		6 mg/kg	D4H72	36.7	63	18	108/066
		6 mg/kg	D7	36.2	74	16	116/067
		6 mg/kg	D14	36.5	75	18	139/073
		6 mg/kg	screening	39.1	98	16	102/061
		6 mg/kg	D1H0	36.7	87	18	099/063
		6 mg/kg	D1H0:30	37.1	95	18	090/052
		6 mg/kg	D1H1	36.9	92	18	096/046
		6 mg/kg	D1H1:30	36.8	91	18	097/051
		6 mg/kg	D1H2	37.5	93	18	095/049

SUBJID	INITIAL	ARM	TIMEPOINT	TEMP (Celsius) *99=Not available*	HR (bpm) *999=Not available*	RR (bpm) *99=Not available*	BP (mmHg) *999/999= Not available*
		6 mg/kg	D1H3	38.2	90	18	089/050
		6 mg/kg	D1H4	39.5	106	20	088/040
		6 mg/kg	D1H6	37.5	86	18	084/044
		6 mg/kg	D1H8	37.1	81	18	080/045
		6 mg/kg	D1H10	36.9	86	18	092/051
		6 mg/kg	D1H12	36.7	80	18	104/055
		6 mg/kg	D1H14	36.6	79	18	098/059
		6 mg/kg	D1H18	36.6	86	18	108/070
		6 mg/kg	D1H20	36.9	77	18	099/070
		6 mg/kg	D2H24	36.7	100	18	115/078
		6 mg/kg	D2H30	36.8	92	18	101/069
		6 mg/kg	D2H36	36.3	87	18	114/075
		6 mg/kg	D2H42	36.7	90	18	107/073
		6 mg/kg	D3H48	36.8	82	18	101/065
		6 mg/kg	D3H54	36.2	74	18	119/069
		6 mg/kg	D3H60	36.1	88	18	120/073
		6 mg/kg	D3H66	37	80	18	115/071
		6 mg/kg	D4H72	37.1	88	18	099/068
		6 mg/kg	D5	36.6	78	18	096/054
		6 mg/kg	D7	36.6	67	16	110/065
		6 mg/kg	D14	36.5	98	16	137/073

16.2.7.5 ECG

SUBJID	INITIAL	ARM	TIMEPOINT	QTCmsec	RESULT	RESOTHER
		1.5 mg/kg	SCREENING	378	Abnormal	no clinically significant
		1.5 mg/kg	D1H1	371	Abnormal	no clinically significant
		1.5 mg/kg	D1H4	393	Abnormal	no clinically significant
		1.5 mg/kg	D1H12	389	Abnormal	no clinically significant
		1.5 mg/kg	D2	371	Abnormal	no clinically significant
		1.5 mg/kg	D3	360	Abnormal	no clinically significant
		1.5 mg/kg	SCREENING	378	Normal	
		1.5 mg/kg	D1H1	411	Normal	
		1.5 mg/kg	D1H4	405	Normal	
		1.5 mg/kg	D1H12	421	Normal	
		1.5 mg/kg	D2	394	Normal	
		1.5 mg/kg	D3	403	Normal	
		1.5 mg/kg	SCREENING	380	Normal	
		1.5 mg/kg	D1H1	390	Normal	
		1.5 mg/kg	D1H4	410	Normal	
		1.5 mg/kg	D1H12	376	Normal	
		1.5 mg/kg	D2	394	Normal	
		1.5 mg/kg	D3	417	Normal	
		3 mg/kg	SCREENING	415	Normal	
		3 mg/kg	D1H1	412	Normal	
		3 mg/kg	D1H4	416	Normal	
		3 mg/kg	D1H12	402	Normal	
		3 mg/kg	D2	408	Normal	
		3 mg/kg	D3	418	Normal	
		3 mg/kg	SCREENING	408	Normal	
		3 mg/kg	D1H1	437	Normal	
		3 mg/kg	D1H4	418	Normal	
		3 mg/kg	D1H12	433	Normal	
		3 mg/kg	D2	438	Normal	
		3 mg/kg	D3	411	Normal	
		3 mg/kg	SCREENING	420	Normal	
		3 mg/kg	D1H1	404	Normal	
		3 mg/kg	D1H4	407	Normal	
		3 mg/kg	D1H12	410	Normal	

TSM02 Individual subject listings part 1

CSR Appendix 16.2

SUBJID	INITIAL	ARM	TIMEPOINT	QTCmsec	RESULT	RESOTHER
		3 mg/kg	D2	397	Normal	
		3 mg/kg	D3	423	Normal	
		6 mg/kg	SCREENING	429	Normal	
		6 mg/kg	D1H1	407	Normal	
		6 mg/kg	D1H4	402	Normal	
		6 mg/kg	D1H12	426	Normal	
		6 mg/kg	D2	413	Normal	
		6 mg/kg	D3	405	Normal	
		6 mg/kg	SCREENING	390	Normal	
		6 mg/kg	D1H1	405	Normal	
		6 mg/kg	D1H4	402	Normal	
		6 mg/kg	D1H12	417	Normal	
		6 mg/kg	D2	405	Normal	
		6 mg/kg	D3	381	Normal	
		6 mg/kg	SCREENING	368	Normal	
		6 mg/kg	D1H1	389	Normal	
		6 mg/kg	D1H4	368	Normal	
		6 mg/kg	D1H12	404	Normal	
		6 mg/kg	D2	385	Normal	
		6 mg/kg	D3	385	Normal	

16.2.8 Listing of individual laboratory measurements by subject

16.2.8.1 Haematology

SUBJID	INITIAL	ARM	DAY	RBC ($10^6/\mu L$) *99=Not avalible*	HCT (%) *99=Not avalible*	HB (g/dL) *99=Not avalible*	MCV (fL) *999=Not avalible*	MCH (pg/cell) *999=Not avalible*	MCHC (pg/cell) *999=Not avalible*	WBC ($10^3/ \mu L$) *99=Not avalible*	NEU (%) *999=Not avalible*	LYM (%) *999=Not avalible*	MON (%) *999=Not avalible*	EOS (%) *999=Not avalible*	PLT ($10^3/ \mu L$) *999=Not avalible*	OTHER
		1.5 mg/kg	1	4.96	44.8	13.5	90.3	27.3	30.2	7.3	83.6	7.1	6.7	1.7	84	
		1.5 mg/kg	2	4.19	38	9.3	90.7	22.2	24.5	5.3	55	15	10	20	48	
		1.5 mg/kg	3	4.07	37.6	10.7	92.4	26.2	28.4	6.9	55	33	0	12	60	
		1.5 mg/kg	4	99	39	11.7	88.9	26.7	30.1	7.2	34	21	1	44	89	
		1.5 mg/kg	5	99	36.7	12.2	89.5	29.7	33.2	8.6	43	22	10	25	119	
		1.5 mg/kg	7	99	39.7	13.4	89.3	30.2	33.8	9.3	31.8	38.1	10.7	19.2	300	
		1.5 mg/kg	14	99	35.8	12.2	89.3	30.4	34	8.6	34.6	48.3	7.2	9.5	360	
		1.5 mg/kg	1	4.2	33.2	11.4	78.7	27	34.3	8.8	87.9	7	3.4	1	89	
		1.5 mg/kg	2	3.5	26.9	8.8	78	25.6	32.8	5.3	68.3	19.6	10.2	1.2	64	
		1.5 mg/kg	3	3.09	24	7.9	77.4	25.6	33	4.2	62	27	10	1	71	
		1.5 mg/kg	4	3.03	23	7.6	76	24.9	32.8	4	51.2	36.1	11.5	0.6	99	
		1.5 mg/kg	7	3.2	24.9	8.3	76.4	25.5	33.4	5.9	55.3	31.4	9.8	2.9	240	
		1.5 mg/kg	14	3.5	29.1	9.9	83.4	28.5	34.1	8	58	34	1	7	408	
		1.5 mg/kg	1	4.7	36.3	11.7	76.5	24.6	32.2	8.4	88.2	7.8	3.5	0.3	96	
		1.5 mg/kg	2	4.1	31.1	10.1	75.9	24.6	32.4	6.4	71.3	23.4	3.8	1.3	81	
		1.5 mg/kg	3	4.9	29.9	9.8	75.1	24.6	32.8	6	58	34.6	5.6	1	72	
		1.5 mg/kg	4	3.9	29.7	9.8	74.8	24.8	33.2	6.2	51	39	10	0	100	
		1.5 mg/kg	7	4.5	33.1	10.8	74.3	24.1	32.5	10.9	43	48.3	7	1.7	349	

SUBJID	INITIAL	ARM	DAY	RBC (10^6/µL) *99=Not avalible*	HCT (%) *99=Not avalible*	HB (g/dL) *99=Not avalible*	MCV (fL) *999=Not avalible*	MCH (pg/cell) *999=Not avalible*	MCHC (pg/cell) *999=Not avalible*	WBC (10^3/ µL) *99=Not avalible*	NEU (%) *999=Not avalible*	LYM (%) *999=Not avalible*	MON (%) *999=Not avalible*	EOS (%) *999=Not avalible*	PLT (10^3/ µL) *999=Not avalible*	OTHER
		1.5 mg/kg	14	4.1	30.7	10.2	74.8	24.8	33.2	9.2	47.3	41.8	8.3	2.4	335	
		3 mg/kg	1	5.9	42.4	13.4	71.2	22.5	31.5	4.9	63	27	0	10	88	
		3 mg/kg	2	4.7	33.1	10.5	70.4	22.4	31.7	3.1	46	35	10	9	82	
		3 mg/kg	3	4.9	34.8	11	69.9	22.1	31.6	3.6	29	52	3	16	114	
		3 mg/kg	4	5.47	38.1	12.4	69.7	22.7	32.5	5.8	38	36	10	16	138	
		3 mg/kg	7	5.4	38.5	12.2	71.6	22.8	31.8	5.8	39.5	40.2	5.1	13.3	242	
		3 mg/kg	14	5.4	38.4	12.3	71.5	22.9	32	6.8	43.9	38.3	5.5	12.3	291	
		3 mg/kg	1	3.8	32	8.2	63.4	21.1	33.3	5.4	31	65	4	0	202	
		3 mg/kg	2	3.1	19.7	6.6	63.4	21.1	33.3	3.2	42	49	1	6	184	
		3 mg/kg	3	3.1	20.1	6.7	64.4	21.6	33.5	3.4	60	38	1	1	127	
		3 mg/kg	4	3.5	22.8	7.7	64.7	21.8	33.7	4.4	33	65	0	2	202	
		3 mg/kg	7	4.5	30.5	10	68.1	22.3	32.7	6.3	32	58	5	5	386	
		3 mg/kg	14	4.5	30.8	10.2	69.1	22.8	33.1	8.8	48.4	39.7	6.7	4.9	207	
		3 mg/kg	1	3.4	35.2	15.4	102.5	44.9	43.9	8.4	72.6	16.6	7.9	2.9	120	
		3 mg/kg	2	2.5	27.7	12.6	108	49.1	45.5	7.6	70	18	10	2	66	HCT 39% repeat
		3 mg/kg	3	3.4	34.3	13.2	102.4	39.3	38.3	7.8	62	23	10	5	64	
		3 mg/kg	4	2.1	38	12.9	112.2	61.9	55.2	15	75.9	10.5	6.6	6.4	85	
		3 mg/kg	7	1.4	34	11.7	112.1	83	74	7.7	44.4	35.9	8.4	11.1	162	
		3 mg/kg	14	2.1	36	12.6	104.5	60.5	57.8	10.1	61	34	0	5	306	
		6 mg/kg	1	5.1	40.1	13.2	78.7	25.8	32.8	7	49	34	10	7	179	
		6 mg/kg	2	4.3	33.9	11	78.5	25.6	32.6	6	57	26	10	7	131	

**SUBJID**	**INITIAL**	**ARM**	**DAY**	**RBC** ($10^6/\mu L$) *99=Not avalible*	**HCT** (%) *99=Not avalible*	**HB** (g/dL) *99=Not avalible*	**MCV** (fL) *999=Not avalible*	**MCH** (pg/cell) *999=Not avalible*	**MCHC** (pg/cell) *999=Not avalible*	**WBC** ($10^3/ \mu L$) *99=Not avalible*	**NEU** (%) *999=Not avalible*	**LYM** (%) *999=Not avalible*	**MON** (%) *999=Not avalible*	**EOS** (%) *999=Not avalible*	**PLT** ($10^3/ \mu L$) *999=Not avalible*	**OTHER**
		6 mg/kg	3	4.4	33.9	11.1	77.4	25.4	32.8	5.6	47	50	0	3	125	
		6 mg/kg	7	4.9	37.5	12.8	76.4	26.1	34.1	8.5	33	45.1	7	14.6	187	
		6 mg/kg	14	4.8	37.7	12.5	77.1	25.6	33.2	10.4	40.4	33.9	7.7	17.3	185	
		6 mg/kg	1	6.2	33	10.5	53.1	16.9	31.8	9.6	76	14	0	1	169	
		6 mg/kg	2	5.6	29.2	9.6	52.3	17.1	32.7	6.1	44	52	0	4	133	
		6 mg/kg	3	5.2	26.4	8.7	51.4	16.9	32.9	6.5	62	33	4	1	301	
		6 mg/kg	7	5.02	26.2	8.4	52.1	16.7	31.9	10.5	49	36	2	13	299	
		6 mg/kg	14	5.41	28.9	9.1	53.4	26.8	31.5	11.3	56	38	0	6	562	
		6 mg/kg	1	4.1	27.3	8.9	66.5	21.6	32.5	5.5	48	35	10	7	112	
		6 mg/kg	2	3.6	24.9	7.9	69.5	22.1	31.8	4	41	43	10	6	130	
		6 mg/kg	3	3.4	22.7	7.4	66	21.6	32.7	4.3	43	48	0	9	70	
		6 mg/kg	4	3.2	21.4	7	66.3	21.6	32.5	5.6	37	50	4	9	149	
		6 mg/kg	7	3.7	25.2	8.2	67.7	21.9	32.3	5.7	47.1	33.3	9.9	9.4	303	
		6 mg/kg	14	3.1	24.4	8.6	77.7	27.2	35	5.4	40.7	40.6	11	7.5	262	

16.2.8.1 Biochemistry

**SUBJID**	**INITIAL**	**ARM**	**DAY**	**GLUC** (mg/dL) *999= Not available*	**BUN** (mg/dL) *999= Not available*	**CREAT** (mg/dL) *99= Not available*	**BILI** (mg/dL) *99= Not available*	**AST** (U/L) *9999= Not available*	**ALT** (U/L) *9999= Not available*	**ALP** (U/L) *9999= Not available*	**SODIUM** (mmol/L) *999= Not available*	**K** (mmol/L) *99= Not available*	**CL** (mmol/L) *999= Not available*	**CO2** (mmol/L) *999= Not available*	**CA** (mmol/ L) *99= Not available*	**CK** (U/L) *9999= Not available*	**LDH** (U/L) *9999= Not available*
		1.5 mg/kg	1	182	12.9	0.9	1.3	39	44	80	132	3.6	97.9	23	7.8	99	326
		1.5 mg/kg	2	154	7.3	0.7	1.3	23	35	53	133.8	3	105.5	21	6.7	72	276
		1.5 mg/kg	3	154	6.4	0.6	0.8	18	29	58	132.5	2.7	102.4	23	7	46	225
		1.5 mg/kg	4	999	4.6	0.5	0.6	28	34	52	136.5	3.3	103.4	22	7.2	30	287
		1.5 mg/kg	7	118	5.4	0.7	0.4	23	49	66	137.6	3.9	102.7	25	8.1	60	250
		1.5 mg/kg	14	92	5	0.7	0.45	22	37	72	142.4	4.1	105.8	29	8.4	54	280
		1.5 mg/kg	1	121	13.4	0.8	1.3	30	25	87	134.9	3.9	99.6	25	8	47	256
		1.5 mg/kg	2	115	8.6	0.6	0.5	23	18	66	136	3.1	105.3	22	7.8	36	250
		1.5 mg/kg	3	103	5.1	0.6	0.8	18	15	60	132.9	3	102.3	26	7.4	34	307
		1.5 mg/kg	4	999	999	99	99	9999	9999	9999	139.5	3.02	107.5	24	6.9	9999	9999
		1.5 mg/kg	7	102	3.6	0.6	0.3	93	90	145	144.6	3.8	108	30	7.8	42	325
		1.5 mg/kg	14	107	8	0.7	0.4	26	36	125	143.8	4.6	106	26	9.2	50	288
		1.5 mg/kg	1	141	9.8	1.1	0.9	25	26	59	132.7	4	97.9	27	8	143	235
		1.5 mg/kg	2	128	7	0.9	0.4	16	18	63	136.3	4	104.6	25	7.1	57	167
		1.5 mg/kg	3	131	5.8	0.9	0.5	14	16	66	136.6	3.7	105.4	30	7.5	23	158
		1.5 mg/kg	4	105	5.2	0.9	0.3	20	18	74	138.6	3.6	103.8	25	8.2	15	159
		1.5 mg/kg	7	121	9.1	0.9	0.4	34	54	73	141	4.7	101.8	32	8.3	32	177
		1.5 mg/kg	14	106	11.6	1	0.4	22	27	67	139.5	4.7	103.2	29	8.6	102	181

SUBJID	INITIAL	ARM	DAY	GLUC (mg/dL) 999= Not available	BUN (mg/dL) 999= Not available	CREAT (mg/dL) 99= Not available	BILI (mg/dL) 99= Not available	AST (U/L) 9999= Not available	ALT (U/L) 9999= Not available	ALP (U/L) 9999= Not available	SODIUM (mmol/L) 999= Not available	K (mmol/L) 99= Not available	CL (mmol/L) 999= Not available	CO2 (mmol/L) 999= Not available	CA (mmol/ L) 99= Not available	CK (U/L) 9999= Not available	LDH (U/L) 9999= Not available
		3 mg/kg	1	118	19.4	0.9	0.7	31	31	97	131.5	4.4	97.3	29	8	67	219
		3 mg/kg	2	131	13.8	0.7	0.5	19	20	69	136	3.6	105.9	27	6.7	44	173
		3 mg/kg	3	121	7.8	0.7	99	18	23	71	136.9	3.9	104.7	30	8	35	178
		3 mg/kg	4	100	10.9	0.8	0.2	37	35	81	140.4	4.3	103.9	27	9.2	46	190
		3 mg/kg	7	91	11.2	0.7	0.3	53	88	80	139.2	4.7	101.4	32	9.4	48	247
		3 mg/kg	14	93	9.6	0.7	0.3	46	75	90	140.8	5.1	104.2	29	9	83	213
		3 mg/kg	1	125	7.4	1	0.6	25	16	52	130.3	4.8	101.1	22	7.5	41	495
		3 mg/kg	2	98	6.3	0.7	0.4	20	8	44	136.9	4	109.1	23	7.5	30	430
		3 mg/kg	3	115	4.8	0.7	0.3	27	17	52	136.5	3.8	107.9	23	7.6	27	408
		3 mg/kg	4	110	4.9	0.7	0.4	39	20	18	142.5	4.4	111.8	23	8.1	29	475
		3 mg/kg	7	70	6.3	0.8	0.4	38	41	73	140.4	4.6	103.2	30	9.4	44	377
		3 mg/kg	14	133	8.2	0.8	0.4	27	23	82	140	4.3	102.7	30	9.1	51	296
		3 mg/kg	1	142	33.1	0.9	0.5	20	34	86	139	4	104.4	23	8.4	29	194
		3 mg/kg	2	104	21.4	0.9	0.5	30	40	76	137.5	3.2	107.7	20	7.7	24	165
		3 mg/kg	3	76	18.7	0.7	0.5	23	38	81	137.5	3.3	106.6	20	7.8	18	197
		3 mg/kg	4	73	20.9	0.7	0.4	26	35	103	137.7	3.5	108.5	20	7.9	16	217
		3 mg/kg	7	113	14.5	0.7	0.3	87	104	104	142.2	3.5	104.9	30	8.2	40	245
		3 mg/kg	14	124	11.1	0.8	0.5	24	54	83	143.4	5.7	107.2	29	8.4	49	260
		6 mg/kg	1	113	13.9	0.9	0.8	20	19	70	143	4.3	105.5	26	9	69	183
		6 mg/kg	2	106	6.9	0.9	1.1	21	12	53	138.7	3.2	107.6	24	8.4	56	245
		6 mg/kg	3	96	7.3	0.9	0.9	18	14	51	138.3	3.8	106	24.1	8.4	45	255

**SUBJID**	**INITIAL**	**ARM**	**DAY**	**GLUC** (mg/dL) *999= Not available*	**BUN** (mg/dL) *999= Not available*	**CREAT** (mg/dL) *99= Not available*	**BILI** (mg/dL) *99= Not available*	**AST** (U/L) *9999= Not available*	**ALT** (U/L) *9999= Not available*	**ALP** (U/L) *9999= Not available*	**SODIUM** (mmol/L) *999= Not available*	**K** (mmol/L) *99= Not available*	**CL** (mmol/L) *999= Not available*	**CO2** (mmol/L) *999= Not available*	**CA** (mmol/ L) *99= Not available*	**CK** (U/L) *9999= Not available*	**LDH** (U/L) *9999= Not available*
		6 mg/kg	7	82	13	0.9	0.9	75	73	62	139.8	4.6	103.4	28.6	9.2	49	248
		6 mg/kg	14	100	9.1	0.9	0.7	28	26	56	140.9	4.7	105.1	31.5	8.9	80	234
		6 mg/kg	1	119	19.3	1.3	1.7	43	53	92	132	3.6	101.1	19.4	8.6	45	186
		6 mg/kg	2	93	18.1	1.1	0.9	29	34	94	134.6	6.3	105.1	21.3	8.1	27	178
		6 mg/kg	3	116	16	1	0.6	27	32	95	137.9	3.7	108.9	20.2	8.4	22	158
		6 mg/kg	7	130	11.9	1.1	0.47	136	157	113	134.9	4.12	104.7	24.4	8.4	38	215
		6 mg/kg	14	85	13.1	0.9	0.56	25	47	81	140.1	4.26	107.1	25	8.8	67	188
		6 mg/kg	1	116	19	0.8	1.1	17	15	83	134.2	3.5	101.7	24.8	8.4	64	164
		6 mg/kg	2	112	9.8	0.7	0.7	16	13	9999	138.5	3.9	108.3	24.4	7.8	55	223
		6 mg/kg	3	115	7.3	0.6	0.6	14	11	65	137.8	3.6	108.9	20.7	7.9	40	216
		6 mg/kg	7	127	12.9	0.7	0.5	72	71	83	136.9	4.7	102.5	27.2	9	32	190
		6 mg/kg	14	98	9.2	0.7	0.6	25	28	97	141.2	4.5	106.7	26.4	9	76	216

16.2.8.2 Coagulation tests

SUBJID	INITIAL	ARM	TIMEPOINT	APTT	PT	INR
		1.5 mg/kg	SCREENING	27.5	12.9	1.08
		1.5 mg/kg	D1H1	39.2	12.4	1.03
		1.5 mg/kg	D1H2	33.8	10.9	0.91
		1.5 mg/kg	D1H5	27.8	11.7	0.98
		1.5 mg/kg	D1H11	26.2	11.5	0.96
		1.5 mg/kg	D1H17	26	11	0.92
		1.5 mg/kg	D1H23	28	12.1	1.01
		1.5 mg/kg	D2H26	32.7	12.5	1.04
		1.5 mg/kg	D2H29	29	11.8	0.98
		1.5 mg/kg	D2predose6	26.2	11.2	0.93
		1.5 mg/kg	D2H31	37.9	12.8	1.07
		1.5 mg/kg	D2H32	34	12	1
		1.5 mg/kg	D2H35	30.7	12.1	1.01
		1.5 mg/kg	D2H41	31.4	12.1	1.01
		1.5 mg/kg	D2H47	23.8	10.6	0.88
		1.5 mg/kg	D3H53	27.3	11.3	0.94
		1.5 mg/kg	D3H59	26.5	10.4	0.87
		1.5 mg/kg	D3H65	27.4	10.2	0.95
		1.5 mg/kg	D3predose12	26.7	10.1	0.84
		1.5 mg/kg	D3H67	40.7	11.5	0.96
		1.5 mg/kg	D3H68	29.7	12.5	1.04
		1.5 mg/kg	D3H71	25.3	15.1	1.25
		1.5 mg/kg	D7	22.9	11.2	0.93
		1.5 mg/kg	D14	26.2	9.8	0.82
		1.5 mg/kg	SCREENING	29.1	12.4	1.03
		1.5 mg/kg	D1H1	46.9	13	1.08
		1.5 mg/kg	D1H2	34.5	12	1
		1.5 mg/kg	D1H5	31.3	12.3	1.02
		1.5 mg/kg	D1H11	30.6	12.7	1.06
		1.5 mg/kg	D1H17	31.1	12.4	1.03
		1.5 mg/kg	D1H23	31.2	12.2	1.02
		1.5 mg/kg	D2H26	36.6	11.3	0.94
		1.5 mg/kg	D2H29	31.4	11.7	0.98
		1.5 mg/kg	D2predose6	31.4	11.8	0.98
		1.5 mg/kg	D2H31	49	12	1

SUBJID	INITIAL	ARM	TIMEPOINT	APTT	PT	INR
		1.5 mg/kg	D2H32	46.9	11.6	0.97
		1.5 mg/kg	D2H35	31.4	11.6	0.97
		1.5 mg/kg	D2H41	31.6	12.4	1.03
		1.5 mg/kg	D2H47	33.1	12.5	1.04
		1.5 mg/kg	D3H53	31.9	12.5	1.04
		1.5 mg/kg	D3H59	29.7	12.6	1.05
		1.5 mg/kg	D3H65	31.1	12.3	1.02
		1.5 mg/kg	D3predose12	29.5	12.8	1.07
		1.5 mg/kg	D3H67	54.6	11.9	0.99
		1.5 mg/kg	D3H68	41.7	12.3	1.02
		1.5 mg/kg	D3H71	32.6	12.4	1.03
		1.5 mg/kg	D7	25	12.3	1.02
		1.5 mg/kg	D14	25.2	11.2	0.93
		1.5 mg/kg	SCREENING	29.2	12.3	1.02
		1.5 mg/kg	D1H1	41.3	12.8	1.07
		1.5 mg/kg	D1H2	36.6	13.8	1.15
		1.5 mg/kg	D1H5	32.1	13.9	1.16
		1.5 mg/kg	D1H11	30.8	13.6	1.13
		1.5 mg/kg	D1H17	31.6	12.9	1.08
		1.5 mg/kg	D1H23	28.6	12.3	1.02
		1.5 mg/kg	D2H26	33	12	1
		1.5 mg/kg	D2H29	28.7	12.2	1.02
		1.5 mg/kg	D2predose6	28.8	11.9	0.99
		1.5 mg/kg	D2H31	41	12.5	1.04
		1.5 mg/kg	D2H32	32.6	12.2	1.02
		1.5 mg/kg	D2H35	28.4	11.9	0.99
		1.5 mg/kg	D2H41	26.8	12	1
		1.5 mg/kg	D2H47	27.3	12.3	1.02
		1.5 mg/kg	D3H53	27.7	11.9	0.99
		1.5 mg/kg	D3H59	25.3	12	1
		1.5 mg/kg	D3H65	27	12.4	1.03
		1.5 mg/kg	D3predose12	27.3	12.7	1.06
		1.5 mg/kg	D3H67	37.7	11.8	0.98
		1.5 mg/kg	D3H68	30.7	12	1
		1.5 mg/kg	D3H71	27.8	12.2	1.02
		1.5 mg/kg	D7	25.2	12.3	1.02
		1.5 mg/kg	D14	26.9	12.6	1.05

SUBJID	INITIAL	ARM	TIMEPOINT	APTT	PT	INR
		3 mg/kg	SCREENING	30	13.3	1.11
		3 mg/kg	D1H1	54.1	12.7	1.06
		3 mg/kg	D1H2	43.7	13.5	1.13
		3 mg/kg	D1H5	34	14.1	1.18
		3 mg/kg	D1H11	34.9	14.7	1.22
		3 mg/kg	D1H17	35.7	14.8	1.22
		3 mg/kg	D1H23	35.5	13.9	1.16
		3 mg/kg	D2H26	43.7	13.7	1.14
		3 mg/kg	D2H29	41.7	14	1.17
		3 mg/kg	D2predose6	error	17.1	1.42
		3 mg/kg	D2H31	error	23.9	1.96
		3 mg/kg	D2H32	81.8	14.5	1.21
		3 mg/kg	D2H35	111.4	15	1.24
		3 mg/kg	D2H41	40.4	13.1	1.09
		3 mg/kg	D2H47	32.3	12.3	1.02
		3 mg/kg	D3H53	32.5	13.2	1.1
		3 mg/kg	D3H59	33.8	12.7	1.06
		3 mg/kg	D3H65	32.8	12	1
		3 mg/kg	D3predose12	31.1	12.4	1.03
		3 mg/kg	D3H67	57.9	11.9	0.99
		3 mg/kg	D3H68	45.6	12.4	1.03
		3 mg/kg	D3H71	33.1	12.1	1.01
		3 mg/kg	D7	23.1	11.2	0.93
		3 mg/kg	D14	26.1	11.4	0.95
		3 mg/kg	SCREENING	26.2	14.8	1.22
		3 mg/kg	D1H1	53.1	16.9	1.4
		3 mg/kg	D1H2	41.6	16.4	1.36
		3 mg/kg	D1H5	42.5	17.5	1.45
		3 mg/kg	D1H11	34.5	18.8	1.56
		3 mg/kg	D1H17	34.7	16.4	1.36
		3 mg/kg	D1H23	38	16.8	1.39
		3 mg/kg	D2H26	51.5	15.2	1.26
		3 mg/kg	D2H29	38.7	15.9	1.32
		3 mg/kg	D2predose6	35.7	16.1	1.33
		3 mg/kg	D2H31	61.3	15.6	1.29
		3 mg/kg	D2H32	45.8	15.9	1.32
		3 mg/kg	D2H35	44.2	15.4	1.27

SUBJID	INITIAL	ARM	TIMEPOINT	APTT	PT	INR
		3 mg/kg	D2H41	33.3	14.8	1.22
		3 mg/kg	D2H47	37.5	14.9	1.23
		3 mg/kg	D3H53	37	15.7	1.3
		3 mg/kg	D3H59	36.5	15.7	1.3
		3 mg/kg	D3H65	39.1	14.8	1.22
		3 mg/kg	D3predose12	37.6	14.5	1.21
		3 mg/kg	D3H67	68.9	14.5	1.21
		3 mg/kg	D3H68	53.2	15	1.24
		3 mg/kg	D3H71	38.9	14.9	1.23
		3 mg/kg	D7	28.3	14.4	1.2
		3 mg/kg	D14	23.4	11.2	1.1
		3 mg/kg	SCREENING	30.7	11.5	0.96
		3 mg/kg	D1H1	48	11.9	0.99
		3 mg/kg	D1H2	36.9	11.7	0.98
		3 mg/kg	D1H5	33.9	12.6	1.05
		3 mg/kg	D1H11	33.3	12.7	1.06
		3 mg/kg	D1H17	37.5	13.2	1.1
		3 mg/kg	D1H23	39	13.9	1.16
		3 mg/kg	D2H26	50.4	14.6	1.22
		3 mg/kg	D2H29	42	14.5	1.21
		3 mg/kg	D2predose6	40.9	14.3	1.19
		3 mg/kg	D2H31	79.4	15.4	1.27
		3 mg/kg	D2H32	47.5	13.9	1.16
		3 mg/kg	D2H35	46	14	1.17
		3 mg/kg	D2H41	48.8	14.1	1.18
		3 mg/kg	D2H47	45.1	13.3	1.11
		3 mg/kg	D3H53	46.3	13	1.08
		3 mg/kg	D3H59	41.4	12.8	1.07
		3 mg/kg	D3H65	40	13.1	1.09
		3 mg/kg	D3predose12	36.1	12.3	1.02
		3 mg/kg	D3H67	55.5	12.7	1.06
		3 mg/kg	D3H68	49.6	13	1.08
		3 mg/kg	D3H71	38	12.5	1.04
		3 mg/kg	D7	29.3	11.9	0.99
		3 mg/kg	D14	28.7	11.9	1.16
		6 mg/kg	SCREENING	26	12.2	1.02
		6 mg/kg	D1H1	55.3	13.1	1.09

SUBJID	INITIAL	ARM	TIMEPOINT	APTT	PT	INR
		6 mg/kg	D1H2	35.7	13.1	1.08
		6 mg/kg	D1H5	28	12.8	1.07
		6 mg/kg	D1H11	30.2	13.1	1.09
		6 mg/kg	D1H17	32.1	14	1.17
		6 mg/kg	D1H23	34	14.1	1.18
		6 mg/kg	D2H26	53.6	14.3	1.19
		6 mg/kg	D2H29	37.1	14.4	1.02
		6 mg/kg	D2predose6	36.6	14.5	1.21
		6 mg/kg	D2H31	80.7	15.5	1.28
		6 mg/kg	D2H32	56.6	14.1	1.18
		6 mg/kg	D2H35	39	14.8	1.22
		6 mg/kg	D2H41	36.3	13.7	1.14
		6 mg/kg	D2H47	37.6	13.2	1.1
		6 mg/kg	D3H53	34.5	13.3	1.11
		6 mg/kg	D3H59	35.8	13	1.08
		6 mg/kg	D3H65	34.4	12.8	1.07
		6 mg/kg	D3predose12	31.4	12	1
		6 mg/kg	D3H67	68.6	12.7	1.06
		6 mg/kg	D3H68	53.9	13.4	1.12
		6 mg/kg	D3H71	36.1	13.2	1.1
		6 mg/kg	D4	27	12.9	1.08
		6 mg/kg	D7	25.1	12.6	1.05
		6 mg/kg	D14	24	10.8	1.06
		6 mg/kg	SCREENING	30.6	13.4	1.12
		6 mg/kg	D1H1	117.4	16.4	1.36
		6 mg/kg	D1H2	66.8	16	1.32
		6 mg/kg	D1H5	41.7	14.9	1.23
		6 mg/kg	D1H11	43.2	14.6	1.22
		6 mg/kg	D1H17	42	14.1	1.18
		6 mg/kg	D1H23	40.8	13.5	1.13
		6 mg/kg	D2H26	68.6	14.1	1.18
		6 mg/kg	D2H29	42	13.8	1.15
		6 mg/kg	D2predose6	39	13.4	1.12
		6 mg/kg	D2H31	117.1	15	1.24
		6 mg/kg	D2H32	76.6	13.1	1.09
		6 mg/kg	D2H35	48.7	13.6	1.13
		6 mg/kg	D2H41	39.4	13.1	1.09

SUBJID	INITIAL	ARM	TIMEPOINT	APTT	PT	INR
		6 mg/kg	D2H47	42.9	13.1	1.09
		6 mg/kg	D3H53	43.2	14	1.17
		6 mg/kg	D3H59	48.9	14.6	1.22
		6 mg/kg	D3H65	44.2	13.1	1.09
		6 mg/kg	D3predose12	43.2	13.9	1.16
		6 mg/kg	D3H67	109.4	13.9	1.16
		6 mg/kg	D3H68	73.4	13.9	1.16
		6 mg/kg	D3H71	41.5	13.6	1.13
		6 mg/kg	D4	27.5	12.7	1
		6 mg/kg	D7	26.9	12.6	1.05
		6 mg/kg	D14	28.4	11.4	0.95
		6 mg/kg	SCREENING	22.6	12.1	1.01
		6 mg/kg	D1H1	60	12.1	1.01
		6 mg/kg	D1H2	40.2	12.3	1.02
		6 mg/kg	D1H5	31	13.7	1.14
		6 mg/kg	D1H11	32.6	13.4	1.12
		6 mg/kg	D1H17	32.9	13.1	1.09
		6 mg/kg	D1H23	34.5	12.9	1.08
		6 mg/kg	D2H26	47.3	13.3	1.11
		6 mg/kg	D2H29	34.7	13	1.08
		6 mg/kg	D2predose6	33.5	12.6	1.05
		6 mg/kg	D2H31	72.4	12.5	1.04
		6 mg/kg	D2H32	58.2	14.3	1.19
		6 mg/kg	D2H35	34.9	12.8	1.07
		6 mg/kg	D2H41	37.3	12.1	1.07
		6 mg/kg	D2H47	44.4	14.4	1.2
		6 mg/kg	D3H53	39.8	13.1	1.09
		6 mg/kg	D3H59	45.8	13.5	1.13
		6 mg/kg	D3H65	46.8	14.1	1.18
		6 mg/kg	D3predose12	30.4	11.5	0.96
		6 mg/kg	D3H67	81.3	12.6	1.05
		6 mg/kg	D3H68	59.1	13.3	1.11
		6 mg/kg	D3H71	40.4	12.9	1.08
		6 mg/kg	D4	26.6	12.1	1.01
		6 mg/kg	D7	25.9	12.3	1.02
		6 mg/kg	D14	24.8	11.9	0.99

16.2.9 Concomitant medications

SUBJID	INITIAL	ARM	CM SEQ	DRUGNAME	ATC TERM	ATC CODE	START DATE	DUR (days)	DOSE	UNIT	ROUTE	FREQ	INDICATION
		1.5 mg/kg	1	METOCLOPAMIDE	metoclopramide	A03FA01		2	10	MG	IV	PRN	NAUSEA, VOMITIN
		1.5 mg/kg	2	PARACETAMOL	paracetamol	N02BE01		ONGOING	500	MG	PO	PRN	FEVER
		1.5 mg/kg	3	ELIXIR KCL	potassium chloride	A12BA01		1	30	ML	PO	TDS	HYPOKALEMIA
		1.5 mg/kg	4	10%CA2+ GLUCONATE	calcium gluconate	A12AA03		1	10	ML	IV	OTHER	HYPOCALCEMIA
		1.5 mg/kg	5	KCL 40mEq IN IV FLUID 1000ML	potassium chloride	B05XA01		1	20	ML	IV	OTHER	HYPOKALEMIA
		1.5 mg/kg	6	KCL 40mEq IN IV FLUID 1000ML	potassium chloride	B05XA01		1	20	ML	IV	OTHER	HYPOKALEMIA
		1.5 mg/kg	7	METOCLOPAMIDE	metoclopramide	A03FA01		1	10	MG	IV	PRN	NAUSEA, VOMITIN
		1.5 mg/kg	8	METOCLOPAMIDE	metoclopramide	A03FA01		ONGOING	10	MG	PO	TDS	NAUSEA, VOMITIN
		1.5 mg/kg	9	SODIUM PHOSPHATE	sodium phosphate	A06AD17		1	30	ML	PO	BD	HYPONATREMIA, HYPOPHOSPHATE
		1.5 mg/kg	10	CALCIUM	Calcium	A12AA		ONGOING	1250	MG	PO	TDS	HYPOCALCEMIA
		1.5 mg/kg	11	K2PO4 40 mEq IN IV FLUID 1000ML	potassium phosphate	B05XA06		2	20	ML	IV	OTHER	HYPOKALEMIA,HY OPHOSPHATE
		1.5 mg/kg	1	Paracetamol	paracetamol	N02BE01		ONGOING	500	MG	PO	PRN	FEVER
		1.5 mg/kg	2	Metoclopramide	metoclopramide	A03FA01		2	10	MG	IV	OTHER	NAUSEA, VOMITIN
		1.5 mg/kg	3	10% Calcium gluconate	calcium gluconate	A12AA03		1	10	ML	IV	OTHER	HYPOCALCEMIA
		1.5 mg/kg	4	E. KCl 30 ml	potassium chloride	A12BA01		1	30	ML	PO	OTHER	HYPOKALEMIA
		1.5 mg/kg	5	ORS	Oral rehydration salt formulations	A07CA		ONGOING	1	OTHER	PO	PRN	ELECTROLYTE IMBALANCE
		1.5 mg/kg	1	PARA	paracetamol	N02BE01		ONGOING	1000	MG	PO	PRN	FEVER
		1.5 mg/kg	2	PLA	metoclopramide	A03FA01		ONGOING	10	MG	IV	OTHER	NAUSEA, VOMITIN

SUBJID	INITIAL	ARM	CM SEQ	DRUGNAME	ATC TERM	ATC CODE	START DATE	DUR (days)	DOSE	UNIT	ROUTE	FREQ	INDICATION
		1.5 mg/kg	3	10%CA2+ GLUCONATE	calcium gluconate	A12AA03		1	10	ML	IV	OTHER	HYPOCALCEMIA
		3 mg/kg	1	Paracetamol	paracetamol	N02BE01		ONGOING	1000	MG	PO	PRN	FEVER
		3 mg/kg	2	10% Calcium gluconate	calcium gluconate	A12AA03		1	10	ML	IV	OTHER	HYPOCALCEMIA
		3 mg/kg	3	Calcium carbonate	calcium carbonate	A12AA04		3	1250	MG	PO	BD	HYPOCALCEMIA
		3 mg/kg	5	0.9% NaCl	sodium chloride	B05XA03		3	40	OTHER	IV	Not available	HYDRATION
		3 mg/kg	1	PARACETAMOL	paracetamol	N02BE01		ONGOING	1000	MG	PO	PRN	FEVER
		3 mg/kg	2	Calcium Carbonate	calcium carbonate	A12AA04		3	1250	MG	PO	TDS	HYPOCALCEMIA
		3 mg/kg	3	Folic acid	folic acid	B03BB01		13	5	MG	PO	OD	ANEMIA
		3 mg/kg	1	PARACETAMOL	paracetamol	N02BE01		ONGOING	500	MG	PO	PRN	FEVER
		3 mg/kg	2	DOMPERIDONE	domperidone	A03FA03		1	10	MG	PO	PRN	VOMITING
		6 mg/kg	1	PARA	paracetamol	N02BE01		2	1000	MG	PO	PRN	FEVER
		6 mg/kg	2	0.9% NSS	sodium chloride	B05XA03		2	1000	ML	IV	Not available	HYDRATION
		6 mg/kg	3	Alum-milk	ANTACIDS	A02A		1	30	ML	PO	OTHER	GASTRITIS
		6 mg/kg	1	PARA	paracetamol	N02BE01		1	1000	MG	PO	PRN	FEVER
		6 mg/kg	1	PLA	metoclopramide	A03FA01		1	1	VIAL	IV	PRN	VOMITING
		6 mg/kg	2	PARA	paracetamol	N02BE01		3	1000	MG	PO	PRN	FEVER
		6 mg/kg	3	0.9% NSS	sodium chloride	B05XA03		3	1000	ML	IV	Not available	DEHYDRATION
		6 mg/kg	4	FBC	iron, multivitamins and minerals	B03AE04		30	1	TABS	PO	TDS	ANEMIA
		6 mg/kg	5	folic acid	folic acid	B03BB01		30	5	MG	PO	OD	ANEMIA
		6 mg/kg	6	Albendazole	albendazole	P02CA03		3	200	MG	PO	OD	PARASITIC INFECTION
