# Supplementary material for: Inhibition of merozoite invasion and transient de-sequestration by sevuparin in humans with Plasmodium falciparum malaria
Source: PLoS One. 2017 Dec 15;12(12):e0188754. doi: 10.1371/journal.pone.0188754 (PMC5731734; doi:10.1371/journal.pone.0188754)
Supplement: S5 Supportive Information — (PDF) [file pone.0188754.s005.pdf]

16 APPENDICES

16.2 Subject data listings (fully anonymized)

16.2.1 Discontinued subjects

SUBJID	INITIAL	ARM	ENROL DATE	D2DATE	D3DATE	D4DATE	D7DATE	STATUS	LAST FOLLOW UP DATE	DISCONTINUE REASON
		Malanil								Subject withdraws consent
		Malanil								Subject withdraws consent
		Sevuparin/DF02+ Malanil								Subject withdraws consent

16.2.2 Protocol deviations

16.2.3 Subjects excluded from the efficacy analysis (if applicable)

Not applicable.

16.2.4 Demographic data and other baseline characteristics

16.2.4.1 Demographics

SUBJID	INITIAL	ARM	SEX	ETHNIC	BIRTHDATE	BLOODTYPE
		Malanil	Male	KAREN		B
		Sevuparin/DF02+ Malanil	Male	KAREN		A
		Sevuparin/DF02+ Malanil	Male	KAREN		A
		Malanil	Male	THAI		O
		Malanil	Male	THAI		A
		Sevuparin/DF02+ Malanil	Male	KAREN		B
		Malanil	Male	KAREN		B
		Sevuparin/DF02+ Malanil	Male	KAREN		B
		Sevuparin/DF02+ Malanil	Male	KAREN		A
		Malanil	Male	KAREN		B
		Malanil	Male	KAREN		O
		Sevuparin/DF02+ Malanil	Male	KAREN		B
		Sevuparin/DF02+ Malanil	Male	KAREN		A
		Malanil	Male	KAREN		O
		Malanil	Male	KAREN		O
		Malanil	Male	KAREN		O
		Sevuparin/DF02+ Malanil	Female	KAREN		O
		Sevuparin/DF02+ Malanil	Female	KAREN		O
		Sevuparin/DF02+ Malanil	Female	KAREN		A
		Malanil	Male	KAREN		O
		Sevuparin/DF02+ Malanil	Male	KAREN		B
		Sevuparin/DF02+ Malanil	Male	KAREN		A
		Sevuparin/DF02+ Malanil	Female	KAREN		B
		Sevuparin/DF02+ Malanil	Male	KAREN		B
		Malanil	Female	KAREN		A
		Malanil	Male	KAREN		O
		Malanil	Male	KAREN		A
		Sevuparin/DF02+ Malanil	Male	KAREN		A
		Malanil	Male	KAREN		AB
		Malanil	Male	KAREN		A
		Malanil	Male	KAREN		AB
		Malanil	Male	KAREN		O
		Sevuparin/DF02+ Malanil	Male	KAREN		A
		Malanil	Male	KAREN		A

SUBJID	INITIAL	ARM	SEX	ETHNIC	BIRTHDATE	BLOODTYPE
		Malanil	Male	KAREN		B
		Malanil	Male	KAREN		A
		Sevuparin/DF02+ Malanil	Male	KAREN		A
		Sevuparin/DF02+ Malanil	Male	KAREN		B
		Sevuparin/DF02+ Malanil	Male	KAREN		O
		Sevuparin/DF02+ Malanil	Male	KAREN		B
		Sevuparin/DF02+ Malanil	Male	KAREN		O
		Malanil	Male	KAREN		B
		Malanil	Male	KAREN		A
		Malanil	Male	KAREN		A

16.2.4.2 Baseline symptoms

SUBJID	INITIAL	ARM	FEVER	DUR (Days)	DIZZI	DUR (Days)	HEADACHE	DUR (Days)	NAUSEA	DUR (Days)	ANOREXIA	DUR (Days)	VOMIT	DUR (Days)	TIMES in 24HOURS
		Malanil	Yes	1	Yes	1	No		No		No		No		
		Sevuparin/DF02+ Malanil	Yes	2	No		Yes	2	No		No		No		
		Sevuparin/DF02+ Malanil	Yes	2	No		Yes	2	No		No		No		
		Malanil	Yes	1	No		Yes	1	No		No		No		
		Malanil	Yes	1	No		Yes	1	No		No		No		
		Sevuparin/DF02+ Malanil	Yes	3	No		Yes	3	Yes	3	Yes	3	No		
		Malanil	Yes	3	No		No		No		No		No		
		Sevuparin/DF02+ Malanil	Yes	4	No		No		No		Yes	4	No		
		Sevuparin/DF02+ Malanil	Yes	1	No		Yes	1	Yes	1	Yes	1	No		
		Malanil	Yes	4	No		Yes	4	No		Yes	4	No		
		Malanil	Yes	5	No		Yes	5	No		No		No		
		Sevuparin/DF02+ Malanil	Yes	3	No		Yes	3	No		No		No		
		Sevuparin/DF02+ Malanil	Yes	2	No		Yes	2	No		No		No		
		Malanil	Yes	2	No		Yes	2	No		No		No		
		Malanil	Yes	3	No		Yes	3	No		No		No		
		Malanil	Yes	1	No		Yes	1	No		No		No		
		Sevuparin/DF02+ Malanil	Yes	4	No		Yes	4	Yes	4	Yes	4	No		

SUBJID	INITIAL	ARM	FEVER	DUR (Days)	DIZZI	DUR (Days)	HEADACHE	DUR (Days)	NAUSEA	DUR (Days)	ANOREXIA	DUR (Days)	VOMIT	DUR (Days)	TIMES in 24HOURS
		Sevuparin/DF02+ Malanil	Yes	2	Yes	2	Yes	2	Yes	2	No		Yes	2	1
		Sevuparin/DF02+ Malanil	Yes	3	Yes	3	Yes	3	No		Yes	3	No		
		Malanil	Yes	5	No		Yes	5	No		No		No		
		Sevuparin/DF02+ Malanil	Yes	5	No		Yes	5	Yes	5	Yes	5	No		
		Sevuparin/DF02+ Malanil	Yes	3	No		Yes	3	Yes	3	Yes	3	No		
		Sevuparin/DF02+ Malanil	Yes	2	No		Yes	2	Yes	2	Yes	2	No		
		Sevuparin/DF02+ Malanil	Yes	3	No		Yes	3	Yes	3	Yes	3	No		
		Malanil	Yes	3	No		Yes	3	Yes	3	Yes	3	No		
		Malanil	Yes	2	No		Yes	2	No		No		No		
		Malanil	Yes	2	No		Yes	2	No		Yes	2	No		
		Sevuparin/DF02+ Malanil	Yes	4	No		Yes	4	Yes	4	Yes	4	No		
		Malanil	Yes	3	No		Yes	3	No		No		No		
		Malanil	Yes	2	No		Yes	2	No		No		No		
		Malanil	Yes	3	No		Yes	3	No		No		No		
		Malanil	Yes	1	No		Yes	1	No		No		No		
		Sevuparin/DF02+ Malanil	Yes	2	No		Yes	2	No		No		No		
		Malanil	Yes	3	No		Yes	3	Yes	3	No		No		
		Malanil	Yes	2	No		Yes	2	No		Yes	2	No		

SUBJID	INITIAL	ARM	FEVER	DUR (Days)	DIZZI	DUR (Days)	HEADACHE	DUR (Days)	NAUSEA	DUR (Days)	ANOREXIA	DUR (Days)	VOMIT	DUR (Days)	TIMES in 24HOURS
		Malanil	Yes	2	No		Yes	2	Yes	1	No		Yes	1	1
		Sevuparin/DF02+ Malanil	Yes	4	No		Yes	4	No		No		No		
		Sevuparin/DF02+ Malanil	Yes	2	No		Yes	2	No		No		No		
		Sevuparin/DF02+ Malanil	Yes	3	No		Yes	3	No		No		No		
		Sevuparin/DF02+ Malanil	Yes	4	No		Yes	4	Yes	4	Yes	4	No		
		Sevuparin/DF02+ Malanil	Yes	5	No		Yes	5	Yes	5	No		No		
		Malanil	Yes	4	No		Yes	4	Yes	4	Yes	4	No		
		Malanil	Yes	4	No		Yes	4	Yes	4	Yes	4	No		
		Malanil	Yes	3	No		Yes	3	Yes	3	Yes	3	No		

16.2.4.2 Baseline symptoms (-continued)

SUBJID	INITIAL	ARM	DIARRHOEA	ABD PAIN	DUR (Days)	PAIN AREA	ITCHING	SKIN RASH	JOINT PAIN	DUR (Days)	MUSCLE PAIN	DUR (Days)
		Malanil	No	No			No	No	No		Yes	1
		Sevuparin/DF02+Malanil	No	No			No	No	No		Yes	2
		Sevuparin/DF02+Malanil	No	No			No	No	No		Yes	2
		Malanil	No	No			No	No	No		No	
		Malanil	No	No			No	No	No		No	
		Sevuparin/DF02+Malanil	No	No			No	No	No		Yes	3
		Malanil	No	No			No	No	Yes	5	Yes	5
		Sevuparin/DF02+Malanil	No	No			No	No	No		Yes	4
		Sevuparin/DF02+Malanil	No	Yes	1	EPI	No	No	No		No	
		Malanil	No	No			No	No	No		Yes	4
		Malanil	No	No			No	No	No		No	
		Sevuparin/DF02+Malanil	No	No			No	No	Yes	3	Yes	3
		Sevuparin/DF02+Malanil	No	No			No	No	No		No	
		Malanil	No	No			No	No	Yes	2	Yes	2
		Malanil	No	No			No	No	No		No	
		Malanil	No	No			No	No	Yes	1	Yes	1
		Sevuparin/DF02+Malanil	No	Yes	4	EPI	No	No	Yes	4	Yes	4
		Sevuparin/DF02+Malanil	No	No			No	No	Yes	2	Yes	2
		Sevuparin/DF02+Malanil	No	Yes	3	EPI	No	No	No		Yes	3
		Malanil	No	No			No	No	No		Yes	5
		Sevuparin/DF02+Malanil	No	No			No	No	Yes	5	Yes	5
		Sevuparin/DF02+Malanil	No	No			No	No	Yes	3	Yes	3

SUBJID	INITIAL	ARM	DIARRHOEA	ABD PAIN	DUR (Days)	PAIN AREA	ITCHING	SKIN RASH	JOINT PAIN	DUR (Days)	MUSCLE PAIN	DUR (Days)
		Sevuparin/DF02+Malanil	No	No			No	No	Yes	2	Yes	2
		Sevuparin/DF02+Malanil	No	No			No	No	Yes	3	Yes	3
		Malanil	No	No			No	No	Yes	3	Yes	3
		Malanil	No	No			No	No	Yes	2	Yes	2
		Malanil	No	No			No	No	Yes	2	Yes	2
		Sevuparin/DF02+Malanil	No	No			No	No	Yes	4	Yes	4
		Malanil	No	No			No	No	No		Yes	3
		Malanil	No	No			No	No	No		Yes	2
		Malanil	No	No			No	No	Yes	3	Yes	3
		Malanil	No	No			No	No	Yes	1	Yes	1
		Sevuparin/DF02+Malanil	No	No			No	No	Yes	2	Yes	2
		Malanil	No	No			No	No	Yes	3	Yes	3
		Malanil	No	No			No	No	No		Yes	2
		Malanil	No	No			No	No	Yes	2	Yes	2
		Sevuparin/DF02+Malanil	No	No			No	No	No		No	
		Sevuparin/DF02+Malanil	No	No			No	No	No		No	
		Sevuparin/DF02+Malanil	No	No			No	No	Yes	3	Yes	3
		Sevuparin/DF02+Malanil	No	No			No	No	Yes	4	Yes	4
		Sevuparin/DF02+Malanil	No	No			No	No	Yes	5	Yes	5
		Malanil	No	No			No	No	No		Yes	4
		Malanil	No	No			No	No	No		Yes	4
		Malanil	No	No			No	No	Yes	3	Yes	3

16.2.4.2 Baseline symptoms (-continued)

SUBJID	INITIAL	ARM	PALPITATIONS	DIFFICULTY BREATHING	DIFFICULTY HEARING	CONFUSION	BLURRED VISION	TIREDNESS	DUR (Days)	OTHER	DUR (Days)
		Malanil	No	No	No	No	No	No			
		Sevuparin/DF02+ Malanil	No	No	No	No	No	No			
		Sevuparin/DF02+ Malanil	No	No	No	No	No	Yes	2		
		Malanil	No	No	No	No	No	No			
		Malanil	No	No	No	No	No	No		SORE THROAT	1
		Sevuparin/DF02+ Malanil	No	No	No	No	No	No			
		Malanil	No	No	No	No	No	No			
		Sevuparin/DF02+ Malanil	No	No	No	No	No	No			
		Sevuparin/DF02+ Malanil	No	No	No	No	No	No			
		Malanil	No	No	No	No	No	No			
		Malanil	No	No	No	No	No	No			
		Sevuparin/DF02+ Malanil	No	No	No	No	No	No			
		Sevuparin/DF02+ Malanil	No	No	No	No	No	No			
		Malanil	No	No	No	No	No	No			
		Malanil	No	No	No	No	No	No			
		Malanil	No	No	No	No	No	No			
		Sevuparin/DF02+ Malanil	No	No	No	No	No	No			

SUBJID	INITIAL	ARM	PALPITATIONS	DIFFICULTY BREATHING	DIFFICULTY HEARING	CONFUSION	BLURRED VISION	TIREDNESS	DUR (Days)	OTHER	DUR (Days)
		Sevuparin/DF02+ Malanil	No	No	No	No	No	No			
		Sevuparin/DF02+ Malanil	No	No	No	No	No	No			
		Malanil	No	No	No	No	No	No			
		Sevuparin/DF02+ Malanil	No	No	No	No	No	No			
		Sevuparin/DF02+ Malanil	No	No	No	No	No	No			
		Sevuparin/DF02+ Malanil	No	No	No	No	No	No			
		Sevuparin/DF02+ Malanil	No	No	No	No	No	No			
		Malanil	No	No	No	No	No	No			
		Malanil	No	No	No	No	No	No			
		Malanil	No	No	No	No	No	No			
		Sevuparin/DF02+ Malanil	No	No	No	No	No	No			
		Malanil	No	No	No	No	No	No			
		Malanil	No	No	No	No	No	No			
		Malanil	No	No	No	No	No	No			
		Malanil	No	No	No	No	No	No			
		Sevuparin/DF02+ Malanil	No	No	No	No	No	No			
		Malanil	No	No	No	No	No	No			
		Malanil	No	No	No	No	No	No			

SUBJID	INITIAL	ARM	PALPITATIONS	DIFFICULTY BREATHING	DIFFICULTY HEARING	CONFUSION	BLURRED VISION	TIREDNESS	DUR (Days)	OTHER	DUR (Days)
		Malanil	No	No	No	No	No	No			
		Sevuparin/DF02+ Malanil	No	No	No	No	No	No			
		Sevuparin/DF02+ Malanil	No	No	No	No	No	No			
		Sevuparin/DF02+ Malanil	No	No	No	No	No	No			
		Sevuparin/DF02+ Malanil	No	No	No	No	No	Yes	4		
		Sevuparin/DF02+ Malanil	No	No	No	No	No	No			
		Malanil	No	No	No	No	No	No			
		Malanil	No	No	No	No	No	Yes	4		
		Malanil	No	No	No	No	No	No			

16.2.4.3 Significant past medical history

SUBJID	INITIAL	ARM	MH SEQ	DISEASE	START DATE	END DATE	MedDRA PT	MedDRA PT CODE	MedDRA SOC	MedDRA SOC CODE
		Sevuparin/DF02+ Malanil	1	HYPONATREMIA			Hyponatraemia	10021036	Metabolism and nutrition disorders	10027433
		Sevuparin/DF02+ Malanil	2	HYPOKALEMIA			Hypokalaemia	10021015	Metabolism and nutrition disorders	10027433
		Sevuparin/DF02+ Malanil	3	ANEMIA			Anaemia	10002034	Blood and lymphatic system disorders	10005329
		Malanil	1	HYPERTHYROIDISM			Hyperthyroidism	10020850	Endocrine disorders	10014698
		Malanil	1	PLATELET DECREASE			Platelet count decreased	10035528	Investigations	10022891
		Sevuparin/DF02+ Malanil	1	HYPOKALEMIA			Hypokalaemia	10021015	Metabolism and nutrition disorders	10027433

16.2.4.4 Drug history

SUBJID	INITIAL	ARM	DH SEQ	DRUGNAME	ATC TERM	ATC CODE	REASON	START DATE	END DATE
		Malanil	1	THYROXINE SODIUM (100MCG)	levothyroxine sodium	H03AA01	HYPERTHYROIDISM		ONGOING
		Sevuparin/DF02 +Malanil	1	PARACETAMOL	paracetamol	N02BE01	FEVER		16-Sep-2012
		Sevuparin/DF02 +Malanil	1	PARACETAMOL	paracetamol	N02BE01	FEVER		ONGOING
		Malanil	1	PARA	paracetamol	N02BE01	HEADACHE AND FEVER		ONGOING
		Sevuparin/DF02 +Malanil	1	PARACETAMOL	paracetamol	N02BE01	FEVER		ONGOING
		Sevuparin/DF02 +Malanil	2	R-DEN ORAL CONTRACEPTIVE	levonorgestrel and ethinylestradiol	G03AA07	PREVENT PREGNANCY		ONGOING
		Sevuparin/DF02 +Malanil	1	R-DEN ORAL CONTRACEPTIVE	levonorgestrel and ethinylestradiol	G03AA07	PREVENT PREGNANCY		ONGOING
		Sevuparin/DF02 +Malanil	1	R-DEN	levonorgestrel and ethinylestradiol	G03AA07	PREVENT PREGNANCY		ONGOING
		Sevuparin/DF02 +Malanil	1	PARACETAMOL	paracetamol	N02BE01	FEVER		ONGOING
		Sevuparin/DF02 +Malanil	1	PARACETAMOL	paracetamol	N02BE01	FEVER		ONGOING
		Sevuparin/DF02 +Malanil	1	PARACETAMOL	paracetamol	N02BE01	FEVER		ONGOING
		Malanil	1	PARACETAMOL	paracetamol	N02BE01	FEVER		ONGOING
		Malanil	2	DEPO PROGESTA	medroxyprogesterone	G03AC06	PREVENT PREGNANCY		ONGOING

SUBJID	INITIAL	ARM	DH SEQ	DRUGNAME	ATC TERM	ATC CODE	REASON	START DATE	END DATE
		Malanil	1	PARACETAMOL	paracetamol	N02BE01	FEVER		ONGOING
		Malanil	1	PARACETAMOL	paracetamol	N02BE01	FEVER		ONGOING
		Sevuparin/DF02 +Malanil	1	PARACETAMOL	paracetamol	N02BE01	FEVER		ONGOING
		Malanil	1	PARACETAMOL	paracetamol	N02BE01	FEVER		
		Malanil	1	PARACETAMOL	paracetamol	N02BE01	FEVER		ONGOING
		Malanil	1	PARACETAMOL	paracetamol	N02BE01	FEVER		ONGOING
		Sevuparin/DF02 +Malanil	1	PARACETAMOL	paracetamol	N02BE01	FEVER		ONGOING
		Malanil	1	PARACETAMOL	paracetamol	N02BE01	FEVER		
		Malanil	1	PARACETAMOL	paracetamol	N02BE01	FEVER		
		Sevuparin/DF02 +Malanil	1	PARACETAMOL	paracetamol	N02BE01	FEVER		ONGOING
		Sevuparin/DF02 +Malanil	1	PARACETAMOL	paracetamol	N02BE01	FEVER		ONGOING
		Malanil	1	PARACETAMOL	paracetamol	N02BE01	FEVER		ONGOING
		Malanil	1	PARACETAMOL	paracetamol	N02BE01	FEVER		ONGOING
		Malanil	1	PARACETAMOL	paracetamol	N02BE01	FEVER		ONGOING

16.2.5 Compliance and drug concentration data

16.2.5.1 Listing of drug administration data (Malanil administration)

SUBJID	INITIAL	ARM	DOSE	DATE	TIME	TABLETS	VOMIT
		Malanil	1		13:30	4	No
		Malanil	2		13:30	4	No
		Malanil	3		13:30	4	No
		Sevuparin/DF02+Malanil	1		13:45	4	No
		Sevuparin/DF02+Malanil	2		13:45	4	No
		Sevuparin/DF02+Malanil	3		13:45	4	No
		Sevuparin/DF02+Malanil	1		17:35	4	No
		Sevuparin/DF02+Malanil	2		17:35	4	No
		Sevuparin/DF02+Malanil	3		17:35	4	No
		Malanil	1		12:15	4	No
		Malanil	2		12:15	4	No
		Malanil	3		12:15	4	No
		Malanil	1		14:10	4	No
		Malanil	2		14:10	4	No
		Malanil	3		14:10	4	No
		Sevuparin/DF02+Malanil	1		14:10	4	No
		Sevuparin/DF02+Malanil	2		14:10	4	No
		Sevuparin/DF02+Malanil	3		14:10	4	No
		Malanil	1		14:40	4	No
		Malanil	2		14:40	4	No
		Malanil	3		14:40	4	No
		Sevuparin/DF02+Malanil	1		14:40	4	No
		Sevuparin/DF02+Malanil	2		14:40	4	No
		Sevuparin/DF02+Malanil	3		14:40	4	No
		Sevuparin/DF02+Malanil	1		12:40	4	No
		Sevuparin/DF02+Malanil	2		12:40	4	No
		Sevuparin/DF02+Malanil	3		12:40	4	No
		Malanil	1		13:10	4	No
		Malanil	2		13:10	4	No
		Malanil	3		13:10	4	No
		Malanil	1		14:10	4	No
		Malanil	2		14:10	4	No
		Malanil	3		14:10	4	No

SUBJID	INITIAL	ARM	DOSE	DATE	TIME	TABLETS	VOMIT
		Sevuparin/DF02+Malanil	1		11:40	4	No
		Sevuparin/DF02+Malanil	2		11:40	4	No
		Sevuparin/DF02+Malanil	3		11:40	4	No
		Sevuparin/DF02+Malanil	1		16:05	4	No
		Sevuparin/DF02+Malanil	2		16:05	4	No
		Sevuparin/DF02+Malanil	3		16:05	4	No
		Malanil	1		14:40	4	No
		Malanil	2		14:40	4	No
		Malanil	3		14:40	4	No
		Malanil	1		11:15	4	No
		Malanil	2		11:15	4	No
		Malanil	3		11:15	4	No
		Malanil	1		14:35	4	No
		Malanil	2		14:35	4	No
		Malanil	3		14:35	4	No
		Sevuparin/DF02+Malanil	1		14:25	4	No
		Sevuparin/DF02+Malanil	2		14:25	4	No
		Sevuparin/DF02+Malanil	3		14:25	4	No
		Sevuparin/DF02+Malanil	1		15:55	4	No
		Sevuparin/DF02+Malanil	2		15:55	4	No
		Sevuparin/DF02+Malanil	3		15:55	4	No
		Sevuparin/DF02+Malanil	1		14:35	4	No
		Sevuparin/DF02+Malanil	2		14:35	4	No
		Sevuparin/DF02+Malanil	3		14:35	4	No
		Malanil	1		14:15	4	No
		Malanil	2		14:15	4	No
		Malanil	3		14:15	4	No
		Sevuparin/DF02+Malanil	1		16:20	4	No
		Sevuparin/DF02+Malanil	2		16:20	4	No
		Sevuparin/DF02+Malanil	3		16:20	4	No
		Sevuparin/DF02+Malanil	1		14:50	4	No
		Sevuparin/DF02+Malanil	2		14:50	4	No
		Sevuparin/DF02+Malanil	3		14:50	4	No
		Sevuparin/DF02+Malanil	1		14:00	4	No
		Sevuparin/DF02+Malanil	2		14:00	4	No
		Sevuparin/DF02+Malanil	3		14:00	4	No
		Sevuparin/DF02+Malanil	1		15:20	4	No

SUBJID	INITIAL	ARM	DOSE	DATE	TIME	TABLETS	VOMIT
		Sevuparin/DF02+Malanil	2		15:20	4	No
		Sevuparin/DF02+Malanil	3		15:20	4	No
		Malanil	1		12:30	4	No
		Malanil	2		12:30	4	No
		Malanil	3		12:30	4	No
		Malanil	1		14:25	4	No
		Malanil	2		14:25	4	No
		Malanil	3		14:25	4	No
		Malanil	1		16:20	4	No
		Malanil	2		16:20	4	No
		Malanil	3		16:20	4	No
		Sevuparin/DF02+Malanil	1		13:10	4	No
		Sevuparin/DF02+Malanil	2		13:10	4	No
		Sevuparin/DF02+Malanil	3		13:10	4	No
		Malanil	1		14:40	4	No
		Malanil	2		14:40	4	No
		Malanil	3		14:40	4	No
		Malanil	1		12:20	4	No
		Malanil	2		12:20	4	No
		Malanil	3		12:20	4	No
		Malanil	1		16:10	4	No
		Malanil	2		16:10	4	No
		Malanil	3		16:10	4	No
		Malanil	1		16:15	4	No
		Malanil	2		16:15	4	No
		Malanil	3		16:15	4	No
		Sevuparin/DF02+Malanil	1		16:20	4	No
		Sevuparin/DF02+Malanil	2		16:20	4	No
		Sevuparin/DF02+Malanil	3		16:20	4	No
		Malanil	1		15:20	4	No
		Malanil	2		15:20	4	No
		Malanil	3		15:20	4	No
		Malanil	1		16:15	4	No
		Malanil	2		16:15	4	No
		Malanil	3		16:15	4	No
		Malanil	1		15:05	4	No
		Malanil	2		15:05	4	No

SUBJID	INITIAL	ARM	DOSE	DATE	TIME	TABLETS	VOMIT
		Malanil	3		15:05	4	No
		Sevuparin/DF02+Malanil	1		16:10	4	No
		Sevuparin/DF02+Malanil	2		16:10	4	No
		Sevuparin/DF02+Malanil	3		16:10	4	No
		Sevuparin/DF02+Malanil	1		15:00	4	No
		Sevuparin/DF02+Malanil	2		15:00	4	No
		Sevuparin/DF02+Malanil	3		15:00	4	No
		Sevuparin/DF02+Malanil	1		18:40	4	No
		Sevuparin/DF02+Malanil	2		18:40	4	No
		Sevuparin/DF02+Malanil	3		18:40	4	No
		Sevuparin/DF02+Malanil	1		13:20	4	No
		Sevuparin/DF02+Malanil	2		13:20	4	No
		Sevuparin/DF02+Malanil	3		13:20	4	No
		Sevuparin/DF02+Malanil	1		17:50	4	No
		Sevuparin/DF02+Malanil	2		17:50	4	No
		Sevuparin/DF02+Malanil	3		17:50	4	No
		Malanil	1		15:30	4	No
		Malanil	2		15:30	4	No
		Malanil	3		15:30	4	No
		Malanil	1		15:20	4	No
		Malanil	2		15:20	4	No
		Malanil	3		15:20	4	No
		Malanil	1		12:10	4	No
		Malanil	2		12:10	4	No
		Malanil	3		12:10	4	No

16.2.5.2 Listing of drug administration data (Sevuparin administration)

SUBJID	INITIAL	ARM	DOSE	DATE	TIME	DOSE (ml)
		Sevuparin/DF02+Malanil	1		14:20	1.1
		Sevuparin/DF02+Malanil	2		20:20	1.1
		Sevuparin/DF02+Malanil	3		02:20	1.1
		Sevuparin/DF02+Malanil	4		08:20	1.1
		Sevuparin/DF02+Malanil	5		14:20	1.1
		Sevuparin/DF02+Malanil	6		20:20	1.1
		Sevuparin/DF02+Malanil	7		02:20	1.1
		Sevuparin/DF02+Malanil	8		08:20	1.1
		Sevuparin/DF02+Malanil	9		14:20	1.1
		Sevuparin/DF02+Malanil	10		20:20	1.1
		Sevuparin/DF02+Malanil	11		02:20	1.1
		Sevuparin/DF02+Malanil	12		08:20	1.1
		Sevuparin/DF02+Malanil	1		18:10	0.96
		Sevuparin/DF02+Malanil	2		00:10	0.96
		Sevuparin/DF02+Malanil	3		06:10	0.96
		Sevuparin/DF02+Malanil	4		12:10	0.96
		Sevuparin/DF02+Malanil	5		18:10	0.96
		Sevuparin/DF02+Malanil	6		00:10	0.96
		Sevuparin/DF02+Malanil	7		06:10	0.96
		Sevuparin/DF02+Malanil	8		12:10	0.96
		Sevuparin/DF02+Malanil	9		18:10	0.96
		Sevuparin/DF02+Malanil	10		00:10	0.96
		Sevuparin/DF02+Malanil	11		06:10	0.96
		Sevuparin/DF02+Malanil	12		12:10	0.96
		Sevuparin/DF02+Malanil	1		14:40	1.28
		Sevuparin/DF02+Malanil	2		20:40	1.28
		Sevuparin/DF02+Malanil	3		02:40	1.28
		Sevuparin/DF02+Malanil	4		08:40	1.28
		Sevuparin/DF02+Malanil	5		14:40	1.28
		Sevuparin/DF02+Malanil	6		20:40	1.28
		Sevuparin/DF02+Malanil	7		02:40	1.28
		Sevuparin/DF02+Malanil	8		08:40	1.28
		Sevuparin/DF02+Malanil	9		14:40	1.28
		Sevuparin/DF02+Malanil	10		20:40	1.28
		Sevuparin/DF02+Malanil	11		02:40	1.28
		Sevuparin/DF02+Malanil	12		08:40	1.28

SUBJID	INITIAL	ARM	DOSE	DATE	TIME	DOSE (ml)
		Sevuparin/DF02+Malanil	1		15:10	1.16
		Sevuparin/DF02+Malanil	2		21:10	1.16
		Sevuparin/DF02+Malanil	3		03:10	1.16
		Sevuparin/DF02+Malanil	4		09:10	1.16
		Sevuparin/DF02+Malanil	5		15:10	1.16
		Sevuparin/DF02+Malanil	6		21:10	1.16
		Sevuparin/DF02+Malanil	7		03:10	1.16
		Sevuparin/DF02+Malanil	8		09:10	1.16
		Sevuparin/DF02+Malanil	9		15:10	1.16
		Sevuparin/DF02+Malanil	10		21:10	1.16
		Sevuparin/DF02+Malanil	11		03:10	1.16
		Sevuparin/DF02+Malanil	12		09:10	1.16
		Sevuparin/DF02+Malanil	1		13:10	0.98
		Sevuparin/DF02+Malanil	2		19:10	0.98
		Sevuparin/DF02+Malanil	3		01:10	0.98
		Sevuparin/DF02+Malanil	4		07:10	0.98
		Sevuparin/DF02+Malanil	5		13:10	0.98
		Sevuparin/DF02+Malanil	6		19:10	0.98
		Sevuparin/DF02+Malanil	7		01:10	0.98
		Sevuparin/DF02+Malanil	8		07:10	0.98
		Sevuparin/DF02+Malanil	9		13:10	0.98
		Sevuparin/DF02+Malanil	10		19:10	0.98
		Sevuparin/DF02+Malanil	11		01:10	0.98
		Sevuparin/DF02+Malanil	12		07:10	0.98
		Sevuparin/DF02+Malanil	1		12:10	1.1
		Sevuparin/DF02+Malanil	2		18:10	1.1
		Sevuparin/DF02+Malanil	3		00:10	1.1
		Sevuparin/DF02+Malanil	4		06:10	1.1
		Sevuparin/DF02+Malanil	5		12:10	1.1
		Sevuparin/DF02+Malanil	6		18:10	1.1
		Sevuparin/DF02+Malanil	7		00:10	1.1
		Sevuparin/DF02+Malanil	8		06:10	1.1
		Sevuparin/DF02+Malanil	9		12:10	1.1
		Sevuparin/DF02+Malanil	10		18:10	1.1
		Sevuparin/DF02+Malanil	11		00:10	1.1
		Sevuparin/DF02+Malanil	12		06:10	1.1
		Sevuparin/DF02+Malanil	1		16:35	1.08

SUBJID	INITIAL	ARM	DOSE	DATE	TIME	DOSE (ml)
		Sevuparin/DF02+Malanil	2		22:35	1.08
		Sevuparin/DF02+Malanil	3		04:35	1.08
		Sevuparin/DF02+Malanil	4		10:35	1.08
		Sevuparin/DF02+Malanil	5		16:35	1.08
		Sevuparin/DF02+Malanil	6		22:35	1.08
		Sevuparin/DF02+Malanil	7		04:35	1.08
		Sevuparin/DF02+Malanil	8		10:35	1.08
		Sevuparin/DF02+Malanil	9		16:35	1.08
		Sevuparin/DF02+Malanil	10		22:35	1.08
		Sevuparin/DF02+Malanil	11		04:35	1.08
		Sevuparin/DF02+Malanil	12		10:35	1.08
		Sevuparin/DF02+Malanil	1		14:55	1.14
		Sevuparin/DF02+Malanil	2		20:55	1.14
		Sevuparin/DF02+Malanil	3		02:55	1.14
		Sevuparin/DF02+Malanil	4		08:55	1.14
		Sevuparin/DF02+Malanil	5		14:55	1.14
		Sevuparin/DF02+Malanil	6		20:55	1.14
		Sevuparin/DF02+Malanil	7		02:55	1.14
		Sevuparin/DF02+Malanil	8		08:55	1.14
		Sevuparin/DF02+Malanil	9		14:55	1.14
		Sevuparin/DF02+Malanil	10		20:55	1.14
		Sevuparin/DF02+Malanil	11		02:55	1.14
		Sevuparin/DF02+Malanil	12		08:55	1.14
		Sevuparin/DF02+Malanil	1		16:25	1.34
		Sevuparin/DF02+Malanil	2		22:25	1.34
		Sevuparin/DF02+Malanil	3		04:25	1.34
		Sevuparin/DF02+Malanil	4		10:25	1.34
		Sevuparin/DF02+Malanil	5		16:25	1.34
		Sevuparin/DF02+Malanil	6		22:25	1.34
		Sevuparin/DF02+Malanil	7		04:25	1.34
		Sevuparin/DF02+Malanil	8		10:25	1.34
		Sevuparin/DF02+Malanil	9		16:25	1.34
		Sevuparin/DF02+Malanil	10		22:25	1.34
		Sevuparin/DF02+Malanil	11		04:25	1.34
		Sevuparin/DF02+Malanil	12		10:25	1.34
		Sevuparin/DF02+Malanil	1		15:05	1
		Sevuparin/DF02+Malanil	2		21:05	1

SUBJID	INITIAL	ARM	DOSE	DATE	TIME	DOSE (ml)
		Sevuparin/DF02+Malanil	3		03:05	1
		Sevuparin/DF02+Malanil	4		09:05	1
		Sevuparin/DF02+Malanil	5		15:05	1
		Sevuparin/DF02+Malanil	6		21:05	1
		Sevuparin/DF02+Malanil	7		03:05	1
		Sevuparin/DF02+Malanil	8		09:05	1
		Sevuparin/DF02+Malanil	9		15:05	1
		Sevuparin/DF02+Malanil	10		21:05	1
		Sevuparin/DF02+Malanil	11		03:05	1
		Sevuparin/DF02+Malanil	12		09:05	1
		Sevuparin/DF02+Malanil	1		16:45	1.12
		Sevuparin/DF02+Malanil	2		22:45	1.12
		Sevuparin/DF02+Malanil	3		04:45	1.12
		Sevuparin/DF02+Malanil	4		10:45	1.12
		Sevuparin/DF02+Malanil	5		16:45	1.12
		Sevuparin/DF02+Malanil	6		22:45	1.12
		Sevuparin/DF02+Malanil	7		04:45	1.12
		Sevuparin/DF02+Malanil	8		10:45	1.12
		Sevuparin/DF02+Malanil	9		16:45	1.12
		Sevuparin/DF02+Malanil	10		22:45	1.12
		Sevuparin/DF02+Malanil	11		04:45	1.12
		Sevuparin/DF02+Malanil	12		10:45	1.12
		Sevuparin/DF02+Malanil	1		15:20	0.98
		Sevuparin/DF02+Malanil	2		21:20	0.98
		Sevuparin/DF02+Malanil	3		03:20	0.98
		Sevuparin/DF02+Malanil	4		09:20	0.98
		Sevuparin/DF02+Malanil	5		15:20	0.98
		Sevuparin/DF02+Malanil	6		21:20	0.98
		Sevuparin/DF02+Malanil	7		03:20	0.98
		Sevuparin/DF02+Malanil	8		09:20	0.98
		Sevuparin/DF02+Malanil	9		15:20	0.98
		Sevuparin/DF02+Malanil	10		21:20	0.98
		Sevuparin/DF02+Malanil	11		03:20	0.98
		Sevuparin/DF02+Malanil	12		09:20	0.98
		Sevuparin/DF02+Malanil	1		14:30	1.34
		Sevuparin/DF02+Malanil	2		20:30	1.34
		Sevuparin/DF02+Malanil	3		02:30	1.34

SUBJID	INITIAL	ARM	DOSE	DATE	TIME	DOSE (ml)
		Sevuparin/DF02+Malanil	4		08:30	1.34
		Sevuparin/DF02+Malanil	5		14:30	1.34
		Sevuparin/DF02+Malanil	6		20:30	1.34
		Sevuparin/DF02+Malanil	7		02:30	1.34
		Sevuparin/DF02+Malanil	8		08:30	1.34
		Sevuparin/DF02+Malanil	9		14:30	1.34
		Sevuparin/DF02+Malanil	10		20:30	1.34
		Sevuparin/DF02+Malanil	11		02:30	1.34
		Sevuparin/DF02+Malanil	12		08:30	1.34
		Sevuparin/DF02+Malanil	1		15:45	0.9
		Sevuparin/DF02+Malanil	2		21:45	0.9
		Sevuparin/DF02+Malanil	3		03:45	0.9
		Sevuparin/DF02+Malanil	4		09:45	0.9
		Sevuparin/DF02+Malanil	5		15:45	0.9
		Sevuparin/DF02+Malanil	6		21:45	0.9
		Sevuparin/DF02+Malanil	7		03:45	0.9
		Sevuparin/DF02+Malanil	8		09:45	0.9
		Sevuparin/DF02+Malanil	9		15:45	0.9
		Sevuparin/DF02+Malanil	10		21:45	0.9
		Sevuparin/DF02+Malanil	11		03:45	0.9
		Sevuparin/DF02+Malanil	12		09:45	0.9
		Sevuparin/DF02+Malanil	1		13:40	1.1
		Sevuparin/DF02+Malanil	2		19:40	1.1
		Sevuparin/DF02+Malanil	3		01:40	1.1
		Sevuparin/DF02+Malanil	4		07:40	1.1
		Sevuparin/DF02+Malanil	5		13:40	1.1
		Sevuparin/DF02+Malanil	6		19:40	1.1
		Sevuparin/DF02+Malanil	7		01:40	1.1
		Sevuparin/DF02+Malanil	8		07:40	1.1
		Sevuparin/DF02+Malanil	9		13:40	1.1
		Sevuparin/DF02+Malanil	10		19:40	1.1
		Sevuparin/DF02+Malanil	11		01:40	1.1
		Sevuparin/DF02+Malanil	12		07:40	1.1
		Sevuparin/DF02+Malanil	1		16:40	0.9
		Sevuparin/DF02+Malanil	2		22:40	0.9
		Sevuparin/DF02+Malanil	3		04:40	0.9
		Sevuparin/DF02+Malanil	4		10:40	0.9

SUBJID	INITIAL	ARM	DOSE	DATE	TIME	DOSE (ml)
		Sevuparin/DF02+Malanil	5		16:40	0.9
		Sevuparin/DF02+Malanil	6		22:40	0.9
		Sevuparin/DF02+Malanil	7		04:40	0.9
		Sevuparin/DF02+Malanil	8		10:40	0.9
		Sevuparin/DF02+Malanil	9		16:40	0.9
		Sevuparin/DF02+Malanil	10		22:40	0.9
		Sevuparin/DF02+Malanil	11		04:40	0.9
		Sevuparin/DF02+Malanil	12		10:40	0.9
		Sevuparin/DF02+Malanil	1		16:30	1.14
		Sevuparin/DF02+Malanil	2		22:30	1.14
		Sevuparin/DF02+Malanil	3		04:30	1.14
		Sevuparin/DF02+Malanil	4		10:30	1.14
		Sevuparin/DF02+Malanil	5		16:30	1.14
		Sevuparin/DF02+Malanil	6		22:30	1.14
		Sevuparin/DF02+Malanil	7		04:30	1.14
		Sevuparin/DF02+Malanil	8		10:30	1.14
		Sevuparin/DF02+Malanil	9		16:30	1.14
		Sevuparin/DF02+Malanil	10		22:30	1.14
		Sevuparin/DF02+Malanil	11		04:30	1.14
		Sevuparin/DF02+Malanil	12		10:30	1.14
		Sevuparin/DF02+Malanil	1		15:20	1.02
		Sevuparin/DF02+Malanil	2		21:20	1.02
		Sevuparin/DF02+Malanil	3		03:20	1.02
		Sevuparin/DF02+Malanil	4		09:20	1.02
		Sevuparin/DF02+Malanil	5		15:20	1.02
		Sevuparin/DF02+Malanil	6		21:20	1.02
		Sevuparin/DF02+Malanil	7		03:20	1.02
		Sevuparin/DF02+Malanil	8		09:20	1.02
		Sevuparin/DF02+Malanil	9		15:20	1.02
		Sevuparin/DF02+Malanil	10		21:20	1.02
		Sevuparin/DF02+Malanil	11		03:20	1.02
		Sevuparin/DF02+Malanil	12		09:20	1.02
		Sevuparin/DF02+Malanil	1		19:20	1.12
		Sevuparin/DF02+Malanil	2		01:20	1.12
		Sevuparin/DF02+Malanil	3		07:20	1.12
		Sevuparin/DF02+Malanil	4		13:20	1.12
		Sevuparin/DF02+Malanil	5		19:20	1.12

SUBJID	INITIAL	ARM	DOSE	DATE	TIME	DOSE (ml)
		Sevuparin/DF02+Malanil	6		01:20	1.12
		Sevuparin/DF02+Malanil	7		07:20	1.12
		Sevuparin/DF02+Malanil	8		13:20	1.12
		Sevuparin/DF02+Malanil	9		19:20	1.12
		Sevuparin/DF02+Malanil	10		01:20	1.12
		Sevuparin/DF02+Malanil	11		07:20	1.12
		Sevuparin/DF02+Malanil	12		13:20	1.12
		Sevuparin/DF02+Malanil	1		13:45	0.88
		Sevuparin/DF02+Malanil	2		19:45	0.88
		Sevuparin/DF02+Malanil	3		01:45	0.88
		Sevuparin/DF02+Malanil	4		07:45	0.88
		Sevuparin/DF02+Malanil	5		13:45	0.88
		Sevuparin/DF02+Malanil	6		19:45	0.88
		Sevuparin/DF02+Malanil	7		01:45	0.88
		Sevuparin/DF02+Malanil	8		07:45	0.88
		Sevuparin/DF02+Malanil	9		13:45	0.88
		Sevuparin/DF02+Malanil	10		19:45	0.88
		Sevuparin/DF02+Malanil	11		01:45	0.88
		Sevuparin/DF02+Malanil	12		07:45	0.88
		Sevuparin/DF02+Malanil	1		18:05	0.88
		Sevuparin/DF02+Malanil	2		00:05	0.88
		Sevuparin/DF02+Malanil	3		06:05	0.88
		Sevuparin/DF02+Malanil	4		12:05	0.88
		Sevuparin/DF02+Malanil	5		18:05	0.88
		Sevuparin/DF02+Malanil	6		00:05	0.88
		Sevuparin/DF02+Malanil	7		06:05	0.88
		Sevuparin/DF02+Malanil	8		12:05	0.88
		Sevuparin/DF02+Malanil	9		18:05	0.88
		Sevuparin/DF02+Malanil	10		00:05	0.88
		Sevuparin/DF02+Malanil	11		06:05	0.88
		Sevuparin/DF02+Malanil	12		12:05	0.88

16.2.6 Individual efficacy response data

16.2.6.1 Malaria Blood smear

(ND = Not Done)

SUBJID	INITIAL	ARM	TIMEPOINT	SPECIES	PARASITAEMIA (/µL)	Tiny Rings (%)	Small Rings (%)	Large Rings (%)	Early Troph (%)	Mid Troph (%)	Late Troph (%)	Gamet (%)	Schiz (%)
		Malanil	SCREENING	Pf	10034	0	0	11	68	21	0	0	0
		Malanil	D1H0	Pf	5670	1	6	8	72	13	0	0	0
		Malanil	D1H1	Pf	3544	2	8	11	71	8	0	0	0
		Malanil	D1H2	Pf	1306	0	6	6	70	18	0	0	0
		Malanil	D1H3	Pf	1156	10	28	16	42	4	0	0	0
		Malanil	D1H4	Pf	709	9	0	18	64	9	0	0	0
		Malanil	D1H6	Pf	224	ND	ND	ND	ND	ND	ND	ND	ND
		Malanil	D1H8	Pf	149	ND	ND	ND	ND	ND	ND	ND	ND
		Malanil	D1H10	Pf	75	ND	ND	ND	ND	ND	ND	ND	ND
		Malanil	D1H11	Pf	75	ND	ND	ND	ND	ND	ND	ND	ND
		Malanil	D1H17	Pf	37	ND	ND	ND	ND	ND	ND	ND	ND
		Malanil	D1H23	NEGATIVE	0	ND	ND	ND	ND	ND	ND	ND	ND
		Malanil	D1H29	NEGATIVE	0	ND	ND	ND	ND	ND	ND	ND	ND
		Malanil	D2H35	NEGATIVE	0	ND	ND	ND	ND	ND	ND	ND	ND
		Malanil	D2H41	NEGATIVE	0	ND	ND	ND	ND	ND	ND	ND	ND
		Malanil	D3H47	NEGATIVE	0	ND	ND	ND	ND	ND	ND	ND	ND
		Sevuparin/DF02+ Malanil	SCREENING	Pf	10468	0	0	10	81	9	0	0	0
		Sevuparin/DF02+	D1H0	Pf	5975	0	0	3	76	19	2	0	0

SUBJID	INITIAL	ARM	TIMEPOINT	SPECIES	PARASITAEMIA (/µL)	Tiny Rings (%)	Small Rings (%)	Large Rings (%)	Early Troph (%)	Mid Troph (%)	Late Troph (%)	Gamet (%)	Schiz (%)
		Malanil											
		Sevuparin/DF02+ Malanil	D1H1	Pf	2438	0	0	0	57	38	5	0	0
		Sevuparin/DF02+ Malanil	D1H2	Pf	956	0	5	13	63	17	2	0	0
		Sevuparin/DF02+ Malanil	D1H3	Pf	454	0	0	6	64	23	7	0	0
		Sevuparin/DF02+ Malanil	D1H4	Pf	311	0	5	21	53	16	5	0	0
		Sevuparin/DF02+ Malanil	D1H6	Pf	239	ND	ND	ND	ND	ND	ND	ND	ND
		Sevuparin/DF02+ Malanil	D1H8	Pf	120	ND	ND	ND	ND	ND	ND	ND	ND
		Sevuparin/DF02+ Malanil	D1H10	Pf	72	ND	ND	ND	ND	ND	ND	ND	ND
		Sevuparin/DF02+ Malanil	D1H11	Pf	48	ND	ND	ND	ND	ND	ND	ND	ND
		Sevuparin/DF02+ Malanil	D1H17	Pf	48	ND	ND	ND	ND	ND	ND	ND	ND
		Sevuparin/DF02+ Malanil	D1H23	Pf	24	ND	ND	ND	ND	ND	ND	ND	ND
		Sevuparin/DF02+ Malanil	D1H29	NEGATIVE	0	ND	ND	ND	ND	ND	ND	ND	ND
		Sevuparin/DF02+ Malanil	D2H35	NEGATIVE	0	ND	ND	ND	ND	ND	ND	ND	ND
		Sevuparin/DF02+ Malanil	D2H41	NEGATIVE	0	ND	ND	ND	ND	ND	ND	ND	ND

SUBJID	INITIAL	ARM	TIMEPOINT	SPECIES	PARASITAEMIA (/µL)	Tiny Rings (%)	Small Rings (%)	Large Rings (%)	Early Troph (%)	Mid Troph (%)	Late Troph (%)	Gamet (%)	Schiz (%)
		Sevuparin/DF02+ Malanil	D3H47	NEGATIVE	0	ND	ND	ND	ND	ND	ND	ND	ND
		Sevuparin/DF02+ Malanil	D7	NEGATIVE	0	ND	ND	ND	ND	ND	ND	ND	ND
		Sevuparin/DF02+ Malanil	D14	NEGATIVE	0	ND	ND	ND	ND	ND	ND	ND	ND
		Sevuparin/DF02+ Malanil	SCREENING	Pf	97440	ND	ND	ND	ND	ND	ND	ND	ND
		Sevuparin/DF02+ Malanil	D1H0	Pf	77140	7	40	45	8	0	0	0	0
		Sevuparin/DF02+ Malanil	D1H1	Pf	60900	1	31	39	29	0	0	0	0
		Sevuparin/DF02+ Malanil	D1H2	Pf	56840	1	41	41	17	0	0	0	0
		Sevuparin/DF02+ Malanil	D1H3	Pf	64960	5	38	34	23	0	0	0	0
		Sevuparin/DF02+ Malanil	D1H4	Pf	60900	0	30	32	38	0	0	0	0
		Sevuparin/DF02+ Malanil	D1H6	Pf	85260	1	23	42	34	0	0	0	0
		Sevuparin/DF02+ Malanil	D1H8	Pf	77140	6	40	31	23	0	0	0	0
		Sevuparin/DF02+ Malanil	D1H10	Pf	77140	2	14	30	54	0	0	0	0
		Sevuparin/DF02+ Malanil	D1H11	Pf	73080	0	18	24	58	0	0	0	0
		Sevuparin/DF02+	D1H17	Pf	44333	0	12	22	58	8	0	0	0

SUBJID	INITIAL	ARM	TIMEPOINT	SPECIES	PARASITAEMIA (/µL)	Tiny Rings (%)	Small Rings (%)	Large Rings (%)	Early Troph (%)	Mid Troph (%)	Late Troph (%)	Gamet (%)	Schiz (%)
		Malanil											
		Sevuparin/DF02+ Malanil	D1H23	Pf	31109	0	12	18	55	14	1	0	0
		Sevuparin/DF02+ Malanil	D1H29	Pf	14935	0	14	18	54	8	6	0	0
		Sevuparin/DF02+ Malanil	D2H35	Pf	5825	ND	ND	ND	ND	ND	ND	ND	ND
		Sevuparin/DF02+ Malanil	D2H41	Pf	1468	ND	ND	ND	ND	ND	ND	ND	ND
		Sevuparin/DF02+ Malanil	D3H47	Pf	868	ND	ND	ND	ND	ND	ND	ND	ND
		Sevuparin/DF02+ Malanil	D3H53	Pf	690	ND	ND	ND	ND	ND	ND	ND	ND
		Sevuparin/DF02+ Malanil	D3H59	Pf	334	ND	ND	ND	ND	ND	ND	ND	ND
		Sevuparin/DF02+ Malanil	D3H65	Pf	89	ND	ND	ND	ND	ND	ND	ND	ND
		Sevuparin/DF02+ Malanil	D3H71	Pf	22	ND	ND	ND	ND	ND	ND	ND	ND
		Sevuparin/DF02+ Malanil	D4	NEGATIVE	0	ND	ND	ND	ND	ND	ND	ND	ND
		Sevuparin/DF02+ Malanil	D5	NEGATIVE	0	ND	ND	ND	ND	ND	ND	ND	ND
		Sevuparin/DF02+ Malanil	D7	NEGATIVE	0	ND	ND	ND	ND	ND	ND	ND	ND
		Sevuparin/DF02+ Malanil	D14	NEGATIVE	0	ND	ND	ND	ND	ND	ND	ND	ND

SUBJID	INITIAL	ARM	TIMEPOINT	SPECIES	PARASITAEMIA (/µL)	Tiny Rings (%)	Small Rings (%)	Large Rings (%)	Early Troph (%)	Mid Troph (%)	Late Troph (%)	Gamet (%)	Schiz (%)
		Malanil	SCREENING	Pf	25031	16	29	23	28	4	0	0	0
		Malanil	D1H0	Pf	14740	5	20	37	32	5	0	1	0
		Malanil	D1H1	Pf	15517	22	46	19	13	0	0	0	0
		Malanil	D1H2	Pf	17072	23	41	21	15	0	0	0	0
		Malanil	D1H3	Pf	14526	14	41	19	26	0	0	0	0
		Malanil	D1H4	Pf	13829	22	21	18	39	0	0	0	0
		Malanil	D1H6	Pf	13909	25	30	19	22	4	0	0	0
		Malanil	D1H8	Pf	11497	17	33	23	24	3	0	0	0
		Malanil	D1H10	Pf	8790	47	31	9	13	0	0	0	0
		Malanil	D1H11	Pf	8362	6	20	18	52	4	0	0	0
		Malanil	D1H17	Pf	5574	25	37	16	18	4	0	0	0
		Malanil	D1H23	Pf	2265	37	27	8	26	2	0	0	0
		Malanil	D1H29	Pf	1251	ND	ND	ND	ND	ND	ND	ND	ND
		Malanil	D2H35	Pf	794	ND	ND	ND	ND	ND	ND	ND	ND
		Malanil	D2H41	Pf	473	ND	ND	ND	ND	ND	ND	ND	ND
		Malanil	D3H47	Pf	265	ND	ND	ND	ND	ND	ND	ND	ND
		Malanil	D3H53	Pf	38	ND	ND	ND	ND	ND	ND	ND	ND
		Malanil	D3H59	NEGATIVE	0	ND	ND	ND	ND	ND	ND	ND	ND
		Malanil	D3H65	NEGATIVE	0	ND	ND	ND	ND	ND	ND	ND	ND
		Malanil	D7	NEGATIVE	0	ND	ND	ND	ND	ND	ND	ND	ND
		Malanil	D14	NEGATIVE	0	ND	ND	ND	ND	ND	ND	ND	ND
		Malanil	SCREENING	Pf	11428	4	16	19	61	0	0	0	0

SUBJID	INITIAL	ARM	TIMEPOINT	SPECIES	PARASITAEMIA (/µL)	Tiny Rings (%)	Small Rings (%)	Large Rings (%)	Early Troph (%)	Mid Troph (%)	Late Troph (%)	Gamet (%)	Schiz (%)
		Malanil	D1H0	Pf	10815	4	21	25	50	0	0	0	0
		Malanil	D1H1	Pf	11543	25	22	17	33	3	0	0	0
		Malanil	D1H2	Pf	9204	29	33	4	32	2	0	0	0
		Malanil	D1H3	Pf	8590	57	20	7	16	0	0	0	0
		Malanil	D1H4	Pf	8284	57	25	5	10	3	0	0	0
		Malanil	D1H6	Pf	8322	41	37	15	7	0	0	0	0
		Malanil	D1H8	Pf	4449	69	26	4	1	0	0	0	0
		Malanil	D1H10	Pf	5407	55	28	6	11	0	0	0	0
		Malanil	D1H11	Pf	5484	37	22	16	25	0	0	0	0
		Malanil	D1H17	Pf	1956	30	40	10	20	0	0	0	0
		Malanil	D1H23	Pf	1060	ND	ND	ND	ND	ND	ND	ND	ND
		Malanil	D1H29	Pf	1855	ND	ND	ND	ND	ND	ND	ND	ND
		Malanil	D2H35	Pf	477	ND	ND	ND	ND	ND	ND	ND	ND
		Malanil	D2H41	Pf	239	ND	ND	ND	ND	ND	ND	ND	ND
		Malanil	D3H47	Pf	24	ND	ND	ND	ND	ND	ND	ND	ND
		Malanil	D3H53	NEGATIVE	0	ND	ND	ND	ND	ND	ND	ND	ND
		Malanil	D3H59	NEGATIVE	0	ND	ND	ND	ND	ND	ND	ND	ND
		Malanil	D7	NEGATIVE	0	ND	ND	ND	ND	ND	ND	ND	ND
		Malanil	D14	NEGATIVE	0	ND	ND	ND	ND	ND	ND	ND	ND
		Sevuparin/DF02+ Malanil	SCREENING	Pf	93960	ND	ND	ND	ND	ND	ND	ND	ND
		Sevuparin/DF02+ Malanil	D1H0	Pf	93960	51	37	12	0	0	0	0	0

SUBJID	INITIAL	ARM	TIMEPOINT	SPECIES	PARASITAEMIA (/µL)	Tiny Rings (%)	Small Rings (%)	Large Rings (%)	Early Troph (%)	Mid Troph (%)	Late Troph (%)	Gamet (%)	Schiz (%)
		Sevuparin/DF02+ Malanil	D1H1	Pf	88740	58	32	10	0	0	0	0	0
		Sevuparin/DF02+ Malanil	D1H2	Pf	83520	57	31	8	4	0	0	0	0
		Sevuparin/DF02+ Malanil	D1H3	Pf	78300	74	25	1	0	0	0	0	0
		Sevuparin/DF02+ Malanil	D1H4	Pf	52200	80	20	0	0	0	0	0	0
		Sevuparin/DF02+ Malanil	D1H6	Pf	41760	40	26	14	18	2	0	0	0
		Sevuparin/DF02+ Malanil	D1H8	Pf	31320	21	26	20	31	1	1	0	0
		Sevuparin/DF02+ Malanil	D1H10	Pf	26100	11	19	19	51	0	0	0	0
		Sevuparin/DF02+ Malanil	D1H11	Pf	20880	4	21	27	48	0	0	0	0
		Sevuparin/DF02+ Malanil	D1H17	Pf	10622	0	7	20	69	4	0	0	0
		Sevuparin/DF02+ Malanil	D1H23	Pf	9308	0	1	16	73	8	2	0	0
		Sevuparin/DF02+ Malanil	D1H29	Pf	7581	ND	ND	ND	ND	ND	ND	ND	ND
		Sevuparin/DF02+ Malanil	D2H35	Pf	4910	ND	ND	ND	ND	ND	ND	ND	ND
		Sevuparin/DF02+ Malanil	D2H41	Pf	758	ND	ND	ND	ND	ND	ND	ND	ND
		Sevuparin/DF02+	D3H47	Pf	199	ND	ND	ND	ND	ND	ND	ND	ND

SUBJID	INITIAL	ARM	TIMEPOINT	SPECIES	PARASITAEMIA (/µL)	Tiny Rings (%)	Small Rings (%)	Large Rings (%)	Early Troph (%)	Mid Troph (%)	Late Troph (%)	Gamet (%)	Schiz (%)
		Malanil											
		Sevuparin/DF02+ Malanil	D3H53	Pf	53	ND	ND	ND	ND	ND	ND	ND	ND
		Sevuparin/DF02+ Malanil	D3H59	Pf	18	ND	ND	ND	ND	ND	ND	ND	ND
		Sevuparin/DF02+ Malanil	D3H65	NEGATIVE	0	ND	ND	ND	ND	ND	ND	ND	ND
		Sevuparin/DF02+ Malanil	D3H71	NEGATIVE	0	ND	ND	ND	ND	ND	ND	ND	ND
		Sevuparin/DF02+ Malanil	D7	NEGATIVE	0	ND	ND	ND	ND	ND	ND	ND	ND
		Sevuparin/DF02+ Malanil	D14	NEGATIVE	0	ND	ND	ND	ND	ND	ND	ND	ND
		Malanil	SCREENING	Pf	11890	42	34	17	7	0	0	0	0
		Malanil	D1H0	Pf	16856	26	44	18	12	0	0	0	0
		Malanil	D1H1	Pf	19909	44	23	6	27	0	0	0	0
		Malanil	D1H2	Pf	24532	29	28	8	34	1	0	0	0
		Malanil	D1H3	Pf	34787	31	32	13	24	0	0	0	0
		Malanil	D1H4	Pf	31605	47	41	8	3	0	1	0	0
		Malanil	D1H6	Pf	40076	23	47	13	16	1	0	0	0
		Malanil	D1H8	Pf	28380	36	26	16	20	2	0	0	0
		Malanil	D1H10	Pf	24897	13	32	22	29	4	0	0	0
		Malanil	D1H11	Pf	18813	23	35	11	28	2	0	1	0
		Malanil	D1H17	Pf	19952	9	18	17	50	6	0	0	0

SUBJID	INITIAL	ARM	TIMEPOINT	SPECIES	PARASITAEMIA (/µL)	Tiny Rings (%)	Small Rings (%)	Large Rings (%)	Early Troph (%)	Mid Troph (%)	Late Troph (%)	Gamet (%)	Schiz (%)
		Malanil	D1H23	Pf	12460	8	18	20	50	2	2	0	0
		Malanil	D1H29	Pf	10858	2	12	11	55	17	3	0	0
		Malanil	D2H35	Pf	5838	ND	ND	ND	ND	ND	ND	ND	ND
		Malanil	D2H41	Pf	1673	ND	ND	ND	ND	ND	ND	ND	ND
		Malanil	D3H47	Pf	480	ND	ND	ND	ND	ND	ND	ND	ND
		Malanil	D3H53	Pf	218	ND	ND	ND	ND	ND	ND	ND	ND
		Malanil	D3H59	Pf	87	ND	ND	ND	ND	ND	ND	ND	ND
		Malanil	D3H65	Pf	0	ND	ND	ND	ND	ND	ND	ND	ND
		Malanil	D3H71	Pf	0	ND	ND	ND	ND	ND	ND	ND	ND
		Malanil	D7	NEGATIVE	0	ND	ND	ND	ND	ND	ND	ND	ND
		Sevuparin/DF02+ Malanil	SCREENING	Pf	11578	7	14	13	52	12	2	0	0
		Sevuparin/DF02+ Malanil	D1H0	Pf	7482	24	20	14	40	2	0	0	0
		Sevuparin/DF02+ Malanil	D1H1	Pf	6708	16	26	13	34	11	0	0	0
		Sevuparin/DF02+ Malanil	D1H2	Pf	5999	4	38	21	29	4	4	0	0
		Sevuparin/DF02+ Malanil	D1H3	Pf	4483	ND	ND	ND	ND	ND	ND	ND	ND
		Sevuparin/DF02+ Malanil	D1H4	Pf	5547	ND	ND	ND	ND	ND	ND	ND	ND
		Sevuparin/DF02+ Malanil	D1H6	Pf	2483	ND	ND	ND	ND	ND	ND	ND	ND
		Sevuparin/DF02+	D1H8	Pf	1774	ND	ND	ND	ND	ND	ND	ND	ND

SUBJID	INITIAL	ARM	TIMEPOINT	SPECIES	PARASITAEMIA (/µL)	Tiny Rings (%)	Small Rings (%)	Large Rings (%)	Early Troph (%)	Mid Troph (%)	Late Troph (%)	Gamet (%)	Schiz (%)
		Malanil											
		Sevuparin/DF02+ Malanil	D1H10	Pf	2258	ND	ND	ND	ND	ND	ND	ND	ND
		Sevuparin/DF02+ Malanil	D1H11	Pf	1387	ND	ND	ND	ND	ND	ND	ND	ND
		Sevuparin/DF02+ Malanil	D1H17	Pf	774	ND	ND	ND	ND	ND	ND	ND	ND
		Sevuparin/DF02+ Malanil	D1H23	Pf	355	ND	ND	ND	ND	ND	ND	ND	ND
		Sevuparin/DF02+ Malanil	D1H29	Pf	180	ND	ND	ND	ND	ND	ND	ND	ND
		Sevuparin/DF02+ Malanil	D2H35	Pf	77	ND	ND	ND	ND	ND	ND	ND	ND
		Sevuparin/DF02+ Malanil	D2H41	NEGATIVE	0	ND	ND	ND	ND	ND	ND	ND	ND
		Sevuparin/DF02+ Malanil	D3H47	NEGATIVE	0	ND	ND	ND	ND	ND	ND	ND	ND
		Sevuparin/DF02+ Malanil	D3H53	NEGATIVE	0	ND	ND	ND	ND	ND	ND	ND	ND
		Sevuparin/DF02+ Malanil	D3H59	NEGATIVE	0	ND	ND	ND	ND	ND	ND	ND	ND
		Sevuparin/DF02+ Malanil	D3H65	NEGATIVE	0	ND	ND	ND	ND	ND	ND	ND	ND
		Sevuparin/DF02+ Malanil	D3H71	NEGATIVE	0	ND	ND	ND	ND	ND	ND	ND	ND
		Sevuparin/DF02+ Malanil	D7	NEGATIVE	0	ND	ND	ND	ND	ND	ND	ND	ND

SUBJID	INITIAL	ARM	TIMEPOINT	SPECIES	PARASITAEMIA (/µL)	Tiny Rings (%)	Small Rings (%)	Large Rings (%)	Early Troph (%)	Mid Troph (%)	Late Troph (%)	Gamet (%)	Schiz (%)
		Sevuparin/DF02+ Malanil	D14	NEGATIVE	0	ND	ND	ND	ND	ND	ND	ND	ND
		Sevuparin/DF02+ Malanil	SCREENING	Pf	82980	12	38	28	22	0	0	0	0
		Sevuparin/DF02+ Malanil	D1H0	Pf	96810	2	43	26	27	2	0	0	0
		Sevuparin/DF02+ Malanil	D1H1	Pf	106030	24	46	17	13	0	0	0	0
		Sevuparin/DF02+ Malanil	D1H2	Pf	92200	20	34	19	26	1	0	0	0
		Sevuparin/DF02+ Malanil	D1H3	Pf	87590	13	40	28	19	0	0	0	0
		Sevuparin/DF02+ Malanil	D1H4	Pf	82980	23	48	11	17	1	0	0	0
		Sevuparin/DF02+ Malanil	D1H6	Pf	78370	39	45	10	4	2	0	0	0
		Sevuparin/DF02+ Malanil	D1H8	Pf	73760	42	46	8	4	0	0	0	0
		Sevuparin/DF02+ Malanil	D1H10	Pf	55320	37	42	13	8	0	0	0	0
		Sevuparin/DF02+ Malanil	D1H11	Pf	50710	35	45	13	7	0	0	0	0
		Sevuparin/DF02+ Malanil	D1H17	Pf	50710	12	41	27	20	0	0	0	0
		Sevuparin/DF02+ Malanil	D1H23	Pf	53071	8	32	19	41	0	0	0	0
		Sevuparin/DF02+	D1H29	Pf	22695	1	15	17	65	2	0	0	0

SUBJID	INITIAL	ARM	TIMEPOINT	SPECIES	PARASITAEMIA (/µL)	Tiny Rings (%)	Small Rings (%)	Large Rings (%)	Early Troph (%)	Mid Troph (%)	Late Troph (%)	Gamet (%)	Schiz (%)
		Malanil											
		Sevuparin/DF02+ Malanil	D2H35	Pf	5085	ND	ND	ND	ND	ND	ND	ND	ND
		Sevuparin/DF02+ Malanil	D2H41	Pf	1503	ND	ND	ND	ND	ND	ND	ND	ND
		Sevuparin/DF02+ Malanil	D3H47	Pf	701	ND	ND	ND	ND	ND	ND	ND	ND
		Sevuparin/DF02+ Malanil	D3H53	Pf	348	ND	ND	ND	ND	ND	ND	ND	ND
		Sevuparin/DF02+ Malanil	D3H59	Pf	196	ND	ND	ND	ND	ND	ND	ND	ND
		Sevuparin/DF02+ Malanil	D3H65	Pf	0	ND	ND	ND	ND	ND	ND	ND	ND
		Sevuparin/DF02+ Malanil	D3H71	Pf	0	ND	ND	ND	ND	ND	ND	ND	ND
		Sevuparin/DF02+ Malanil	D7	Pf	0	ND	ND	ND	ND	ND	ND	ND	ND
		Sevuparin/DF02+ Malanil	D14	Pf	0	ND	ND	ND	ND	ND	ND	ND	ND
		Malanil	SCREENING	Pf	18216	3	10	20	39	16	12	0	0
		Malanil	D1H0	Pf	20134	7	24	14	32	14	9	0	0
		Malanil	D1H1	Pf	20671	17	23	17	30	8	5	0	0
		Malanil	D1H2	Pf	19942	14	21	10	32	14	9	0	0
		Malanil	D1H3	Pf	19098	11	25	15	22	9	18	0	0
		Malanil	D1H4	Pf	17641	15	31	7	19	11	17	0	0

SUBJID	INITIAL	ARM	TIMEPOINT	SPECIES	PARASITAEMIA (/µL)	Tiny Rings (%)	Small Rings (%)	Large Rings (%)	Early Troph (%)	Mid Troph (%)	Late Troph (%)	Gamet (%)	Schiz (%)
		Malanil	D1H6	Pf	14074	37	37	10	9	5	2	0	0
		Malanil	D1H8	Pf	11083	25	34	16	19	3	3	0	0
		Malanil	D1H10	Pf	8936	58	42	0	0	0	0	0	0
		Malanil	D1H11	Pf	25004	30	26	4	22	12	6	0	0
		Malanil	D1H17	Pf	5561	ND	ND	ND	ND	ND	ND	ND	ND
		Malanil	D1H23	Pf	2968	ND	ND	ND	ND	ND	ND	ND	ND
		Malanil	D1H29	Pf	2767	ND	ND	ND	ND	ND	ND	ND	ND
		Malanil	D2H35	Pf	905	ND	ND	ND	ND	ND	ND	ND	ND
		Malanil	D2H41	Pf	302	ND	ND	ND	ND	ND	ND	ND	ND
		Malanil	D3H47	Pf	75	ND	ND	ND	ND	ND	ND	ND	ND
		Malanil	D3H53	Pf	25	ND	ND	ND	ND	ND	ND	ND	ND
		Malanil	D3H59	NEGATIVE	0	ND	ND	ND	ND	ND	ND	ND	ND
		Malanil	D3H65	NEGATIVE	0	ND	ND	ND	ND	ND	ND	ND	ND
		Malanil	D7	NEGATIVE	0	ND	ND	ND	ND	ND	ND	ND	ND
		Malanil	D14	NEGATIVE	0	ND	ND	ND	ND	ND	ND	ND	ND
		Malanil	SCREENING	Pf	11494	60	34	0	6	0	0	0	0
		Malanil	D1H0	Pf	24397	74	16	6	4	0	0	0	0
		Malanil	D1H1	Pf	27896	75	19	4	1	0	1	0	0
		Malanil	D1H2	Pf	57640	50	36	8	5	1	0	0	0
		Malanil	D1H3	Pf	32999	50	38	6	6	0	0	0	0
		Malanil	D1H4	Pf	38686	58	35	5	2	0	0	0	0

SUBJID	INITIAL	ARM	TIMEPOINT	SPECIES	PARASITAEMIA (/µL)	Tiny Rings (%)	Small Rings (%)	Large Rings (%)	Early Troph (%)	Mid Troph (%)	Late Troph (%)	Gamet (%)	Schiz (%)
		Malanil	D1H6	Pf	33291	42	36	11	11	0	0	0	0
		Malanil	D1H8	Pf	32975	20	38	22	19	1	0	0	0
		Malanil	D1H10	Pf	29354	52	42	6	0	0	0	0	0
		Malanil	D1H11	Pf	23182	46	41	11	2	0	0	0	0
		Malanil	D1H17	Pf	22672	25	33	21	19	2	0	0	0
		Malanil	D1H23	Pf	15233	6	23	23	36	12	0	0	0
		Malanil	D1H29	Pf	14830	5	25	18	47	5	0	0	0
		Malanil	D2H35	Pf	6307	2	19	13	44	19	3	0	0
		Malanil	D2H41	Pf	2257	ND	ND	ND	ND	ND	ND	ND	ND
		Malanil	D3H47	Pf	546	ND	ND	ND	ND	ND	ND	ND	ND
		Malanil	D3H53	Pf	52	ND	ND	ND	ND	ND	ND	ND	ND
		Malanil	D3H59	NEGATIVE	0	ND	ND	ND	ND	ND	ND	ND	ND
		Malanil	D3H65	NEGATIVE	0	ND	ND	ND	ND	ND	ND	ND	ND
		Malanil	D7	Pf	0	ND	ND	ND	ND	ND	ND	ND	ND
		Malanil	D14	NEGATIVE	0	ND	ND	ND	ND	ND	ND	ND	ND
		Sevuparin/DF02+ Malanil	SCREENING	Pf	26062	1	0	4	60	23	12	0	0
		Sevuparin/DF02+ Malanil	D1H0	Pf	20161	0	1	8	58	24	9	0	0
		Sevuparin/DF02+ Malanil	D1H1	Pf	15089	0	0	0	14	25	61	0	0
		Sevuparin/DF02+ Malanil	D1H2	Pf	8485	0	1	1	2	33	63	0	0
		Sevuparin/DF02+	D1H3	Pf	4913	0	0	2	22	27	49	0	0

SUBJID	INITIAL	ARM	TIMEPOINT	SPECIES	PARASITAEMIA (/µL)	Tiny Rings (%)	Small Rings (%)	Large Rings (%)	Early Troph (%)	Mid Troph (%)	Late Troph (%)	Gamet (%)	Schiz (%)
		Malanil											
		Sevuparin/DF02+ Malanil	D1H4	Pf	1691	0	4	7	31	29	29	0	0
		Sevuparin/DF02+ Malanil	D1H6	Pf	526	0	12	12	28	12	36	0	0
		Sevuparin/DF02+ Malanil	D1H8	Pf	112	0	4	8	10	26	52	0	0
		Sevuparin/DF02+ Malanil	D1H10	Pf	64	ND	ND	ND	ND	ND	ND	ND	ND
		Sevuparin/DF02+ Malanil	D1H11	Pf	48	ND	ND	ND	ND	ND	ND	ND	ND
		Sevuparin/DF02+ Malanil	D1H17	Pf	32	ND	ND	ND	ND	ND	ND	ND	ND
		Sevuparin/DF02+ Malanil	D1H23	Pf	16	ND	ND	ND	ND	ND	ND	ND	ND
		Sevuparin/DF02+ Malanil	D1H29	NEGATIVE	0	ND	ND	ND	ND	ND	ND	ND	ND
		Sevuparin/DF02+ Malanil	D2H35	NEGATIVE	0	ND	ND	ND	ND	ND	ND	ND	ND
		Sevuparin/DF02+ Malanil	D2H41	NEGATIVE	0	ND	ND	ND	ND	ND	ND	ND	ND
		Sevuparin/DF02+ Malanil	D3H47	NEGATIVE	0	ND	ND	ND	ND	ND	ND	ND	ND
		Sevuparin/DF02+ Malanil	D3H53	NEGATIVE	0	ND	ND	ND	ND	ND	ND	ND	ND
		Sevuparin/DF02+ Malanil	D3H59	NEGATIVE	0	ND	ND	ND	ND	ND	ND	ND	ND

SUBJID	INITIAL	ARM	TIMEPOINT	SPECIES	PARASITAEMIA (/µL)	Tiny Rings (%)	Small Rings (%)	Large Rings (%)	Early Troph (%)	Mid Troph (%)	Late Troph (%)	Gamet (%)	Schiz (%)
		Sevuparin/DF02+ Malanil	D3H65	NEGATIVE	0	ND	ND	ND	ND	ND	ND	ND	ND
		Sevuparin/DF02+ Malanil	D3H71	NEGATIVE	0	ND	ND	ND	ND	ND	ND	ND	ND
		Sevuparin/DF02+ Malanil	D7	NEGATIVE	0	ND	ND	ND	ND	ND	ND	ND	ND
		Sevuparin/DF02+ Malanil	SCREENING	Pf	27492	11	20	23	34	11	1	0	0
		Sevuparin/DF02+ Malanil	D1H0	Pf	19434	23	24	14	27	10	2	0	0
		Sevuparin/DF02+ Malanil	D1H1	Pf	13438	30	22	11	24	11	2	0	0
		Sevuparin/DF02+ Malanil	D1H2	Pf	11708	25	13	9	44	9	0	0	0
		Sevuparin/DF02+ Malanil	D1H3	Pf	10073	44	25	9	19	0	3	0	0
		Sevuparin/DF02+ Malanil	D1H4	Pf	8864	20	20	20	28	6	6	0	0
		Sevuparin/DF02+ Malanil	D1H6	Pf	7229	17	21	17	23	17	5	0	0
		Sevuparin/DF02+ Malanil	D1H8	Pf	4740	11	18	18	37	8	8	0	0
		Sevuparin/DF02+ Malanil	D1H10	Pf	3200	ND	ND	ND	ND	ND	ND	ND	ND
		Sevuparin/DF02+ Malanil	D1H11	Pf	2963	ND	ND	ND	ND	ND	ND	ND	ND
		Sevuparin/DF02+	D1H17	Pf	1351	ND	ND	ND	ND	ND	ND	ND	ND

SUBJID	INITIAL	ARM	TIMEPOINT	SPECIES	PARASITAEMIA (/µL)	Tiny Rings (%)	Small Rings (%)	Large Rings (%)	Early Troph (%)	Mid Troph (%)	Late Troph (%)	Gamet (%)	Schiz (%)
		Malanil											
		Sevuparin/DF02+ Malanil	D1H23	Pf	640	ND	ND	ND	ND	ND	ND	ND	ND
		Sevuparin/DF02+ Malanil	D1H29	Pf	162	ND	ND	ND	ND	ND	ND	ND	ND
		Sevuparin/DF02+ Malanil	D2H35	Pf	22	ND	ND	ND	ND	ND	ND	ND	ND
		Sevuparin/DF02+ Malanil	D2H41	NEGATIVE	0	ND	ND	ND	ND	ND	ND	ND	ND
		Sevuparin/DF02+ Malanil	D3H47	NEGATIVE	0	ND	ND	ND	ND	ND	ND	ND	ND
		Sevuparin/DF02+ Malanil	D3H53	NEGATIVE	0	ND	ND	ND	ND	ND	ND	ND	ND
		Sevuparin/DF02+ Malanil	D3H59	NEGATIVE	0	ND	ND	ND	ND	ND	ND	ND	ND
		Sevuparin/DF02+ Malanil	D3H65	NEGATIVE	0	ND	ND	ND	ND	ND	ND	ND	ND
		Sevuparin/DF02+ Malanil	D3H71	NEGATIVE	0	ND	ND	ND	ND	ND	ND	ND	ND
		Sevuparin/DF02+ Malanil	D7	NEGATIVE	0	ND	ND	ND	ND	ND	ND	ND	ND
		Sevuparin/DF02+ Malanil	D14	NEGATIVE	0	ND	ND	ND	ND	ND	ND	ND	ND
		Malanil	SCREENING	Pf	10886	20	25	25	25	0	5	0	0
		Malanil	D1H0	Pf	4956	14	21	29	36	0	0	0	0
		Malanil	D1H1	Pf	4632	ND	ND	ND	ND	ND	ND	ND	ND

SUBJID	INITIAL	ARM	TIMEPOINT	SPECIES	PARASITAEMIA (/µL)	Tiny Rings (%)	Small Rings (%)	Large Rings (%)	Early Troph (%)	Mid Troph (%)	Late Troph (%)	Gamet (%)	Schiz (%)
		Malanil	D1H2	Pf	4307	ND	ND	ND	ND	ND	ND	ND	ND
		Malanil	D1H3	Pf	3717	ND	ND	ND	ND	ND	ND	ND	ND
		Malanil	D1H4	Pf	2626	ND	ND	ND	ND	ND	ND	ND	ND
		Malanil	D1H6	Pf	1859	ND	ND	ND	ND	ND	ND	ND	ND
		Malanil	D1H8	Pf	1416	ND	ND	ND	ND	ND	ND	ND	ND
		Malanil	D1H10	Pf	1121	ND	ND	ND	ND	ND	ND	ND	ND
		Malanil	D1H11	Pf	915	ND	ND	ND	ND	ND	ND	ND	ND
		Malanil	D1H17	Pf	649	ND	ND	ND	ND	ND	ND	ND	ND
		Malanil	D1H23	Pf	157	ND	ND	ND	ND	ND	ND	ND	ND
		Malanil	D1H29	Pf	59	ND	ND	ND	ND	ND	ND	ND	ND
		Malanil	D2H35	Pf	20	ND	ND	ND	ND	ND	ND	ND	ND
		Malanil	D2H41	NEGATIVE	0	ND	ND	ND	ND	ND	ND	ND	ND
		Malanil	D3H47	NEGATIVE	0	ND	ND	ND	ND	ND	ND	ND	ND
		Malanil	D3H53	NEGATIVE	0	ND	ND	ND	ND	ND	ND	ND	ND
		Malanil	D3H59	NEGATIVE	0	ND	ND	ND	ND	ND	ND	ND	ND
		Malanil	D3H65	NEGATIVE	0	ND	ND	ND	ND	ND	ND	ND	ND
		Malanil	D3H71	NEGATIVE	0	ND	ND	ND	ND	ND	ND	ND	ND
		Malanil	D7	NEGATIVE	0	ND	ND	ND	ND	ND	ND	ND	ND
		Malanil	D14	NEGATIVE	0	ND	ND	ND	ND	ND	ND	ND	ND
		Malanil	SCREENING	Pf	73500	75	23	1	1	0	0	0	0
		Malanil	D1H0	Pf	78400	72	22	5	1	0	0	0	0
		Malanil	D1H1	Pf	83300	57	40	3	0	0	0	0	0

SUBJID	INITIAL	ARM	TIMEPOINT	SPECIES	PARASITAEMIA (/µL)	Tiny Rings (%)	Small Rings (%)	Large Rings (%)	Early Troph (%)	Mid Troph (%)	Late Troph (%)	Gamet (%)	Schiz (%)
		Malanil	D1H2	Pf	112700	70	28	2	0	0	0	0	0
		Malanil	D1H3	Pf	122500	70	25	5	0	0	0	0	0
		Malanil	D1H4	Pf	112700	70	26	4	0	0	0	0	0
		Malanil	D1H6	Pf	98000	72	24	4	0	0	0	0	0
		Malanil	D1H8	Pf	98000	46	43	11	0	0	0	0	0
		Malanil	D1H10	Pf	88200	34	40	15	11	0	0	0	0
		Malanil	D1H11	Pf	83300	31	39	14	16	0	0	0	0
		Malanil	D1H17	Pf	68600	8	30	22	38	2	0	0	0
		Malanil	D1H23	Pf	47850	2	7	14	74	3	0	0	0
		Malanil	D1H29	Pf	24552	0	8	13	66	13	0	0	0
		Malanil	D2H35	Pf	9179	ND	ND	ND	ND	ND	ND	ND	ND
		Malanil	D2H41	Pf	2065	ND	ND	ND	ND	ND	ND	ND	ND
		Malanil	D3H47	Pf	817	ND	ND	ND	ND	ND	ND	ND	ND
		Malanil	D3H53	Pf	374	ND	ND	ND	ND	ND	ND	ND	ND
		Malanil	D3H59	Pf	93	ND	ND	ND	ND	ND	ND	ND	ND
		Malanil	D3H65	NEGATIVE	0	ND	ND	ND	ND	ND	ND	ND	ND
		Malanil	D3H71	NEGATIVE	0	ND	ND	ND	ND	ND	ND	ND	ND
		Malanil	D7	NEGATIVE	0	ND	ND	ND	ND	ND	ND	ND	ND
		Malanil	D14	NEGATIVE	0	ND	ND	ND	ND	ND	ND	ND	ND
		Malanil	SCREENING	Pf	15691	74	22	4	0	0	0	0	0
		Malanil	D1H0	Pf	21412	60	22	11	7	0	0	0	0
		Malanil	D1H1	Pf	23350	52	33	13	2	0	0	0	0

SUBJID	INITIAL	ARM	TIMEPOINT	SPECIES	PARASITAEMIA (/µL)	Tiny Rings (%)	Small Rings (%)	Large Rings (%)	Early Troph (%)	Mid Troph (%)	Late Troph (%)	Gamet (%)	Schiz (%)
		Malanil	D1H2	Pf	24004	38	43	12	7	0	0	0	0
		Malanil	D1H3	Pf	24051	40	50	5	5	0	0	0	0
		Malanil	D1H4	Pf	22790	23	51	21	5	0	0	0	0
		Malanil	D1H6	Pf	22650	52	35	11	2	0	0	0	0
		Malanil	D1H8	Pf	21435	34	47	14	5	0	0	0	0
		Malanil	D1H10	Pf	19731	42	36	17	5	0	0	0	0
		Malanil	D1H11	Pf	17466	35	37	18	10	0	0	0	0
		Malanil	D1H17	Pf	17092	5	25	29	36	5	0	0	0
		Malanil	D1H23	Pf	16324	0	11	12	60	12	5	0	0
		Malanil	D1H29	Pf	10946	0	4	12	55	23	6	0	0
		Malanil	D2H35	Pf	9632	0	9	11	54	15	11	0	0
		Malanil	D2H41	Pf	4493	ND	ND	ND	ND	ND	ND	ND	ND
		Malanil	D3H47	Pf	1245	ND	ND	ND	ND	ND	ND	ND	ND
		Malanil	D3H53	Pf	599	ND	ND	ND	ND	ND	ND	ND	ND
		Malanil	D3H59	Pf	263	ND	ND	ND	ND	ND	ND	ND	ND
		Malanil	D3H65	NEGATIVE	0	ND	ND	ND	ND	ND	ND	ND	ND
		Malanil	D3H71	NEGATIVE	0	ND	ND	ND	ND	ND	ND	ND	ND
		Malanil	D7	NEGATIVE	0	ND	ND	ND	ND	ND	ND	ND	ND
		Malanil	D14	NEGATIVE	0	ND	ND	ND	ND	ND	ND	ND	ND
		Sevuparin/DF02+ Malanil	SCREENING	Pf	10679	14	40	39	7	0	0	0	0
		Sevuparin/DF02+ Malanil	D1H0	Pf	11826	2	36	36	23	2	1	0	0

SUBJID	INITIAL	ARM	TIMEPOINT	SPECIES	PARASITAEMIA (/µL)	Tiny Rings (%)	Small Rings (%)	Large Rings (%)	Early Troph (%)	Mid Troph (%)	Late Troph (%)	Gamet (%)	Schiz (%)
		Sevuparin/DF02+ Malanil	D1H1	Pf	10953	30	30	15	18	7	0	0	0
		Sevuparin/DF02+ Malanil	D1H2	Pf	12899	38	31	16	8	7	0	0	0
		Sevuparin/DF02+ Malanil	D1H3	Pf	12899	31	40	18	8	3	0	0	0
		Sevuparin/DF02+ Malanil	D1H4	Pf	13024	26	42	16	14	2	0	0	0
		Sevuparin/DF02+ Malanil	D1H6	Pf	16517	18	48	20	14	0	0	0	0
		Sevuparin/DF02+ Malanil	D1H8	Pf	14172	16	40	19	25	0	0	0	0
		Sevuparin/DF02+ Malanil	D1H10	Pf	15918	22	36	25	17	0	0	0	0
		Sevuparin/DF02+ Malanil	D1H11	Pf	12550	46	35	12	7	0	0	0	0
		Sevuparin/DF02+ Malanil	D1H17	Pf	12226	7	24	45	24	0	0	0	0
		Sevuparin/DF02+ Malanil	D1H23	Pf	8932	3	15	18	55	6	3	0	0
		Sevuparin/DF02+ Malanil	D1H29	Pf	5083	ND	ND	ND	ND	ND	ND	ND	ND
		Sevuparin/DF02+ Malanil	D2H35	Pf	1276	ND	ND	ND	ND	ND	ND	ND	ND
		Sevuparin/DF02+ Malanil	D2H41	Pf	203	ND	ND	ND	ND	ND	ND	ND	ND
		Sevuparin/DF02+	D3H47	Pf	61	ND	ND	ND	ND	ND	ND	ND	ND

SUBJID	INITIAL	ARM	TIMEPOINT	SPECIES	PARASITAEMIA (/µL)	Tiny Rings (%)	Small Rings (%)	Large Rings (%)	Early Troph (%)	Mid Troph (%)	Late Troph (%)	Gamet (%)	Schiz (%)
		Malanil											
		Sevuparin/DF02+ Malanil	D3H53	Pf	48	ND	ND	ND	ND	ND	ND	ND	ND
		Sevuparin/DF02+ Malanil	D3H59	NEGATIVE	0	ND	ND	ND	ND	ND	ND	ND	ND
		Sevuparin/DF02+ Malanil	D3H65	NEGATIVE	0	ND	ND	ND	ND	ND	ND	ND	ND
		Sevuparin/DF02+ Malanil	D7	Pf	0	ND	ND	ND	ND	ND	ND	ND	ND
		Sevuparin/DF02+ Malanil	D14	NEGATIVE	0	ND	ND	ND	ND	ND	ND	ND	ND
		Sevuparin/DF02+ Malanil	SCREENING	Pf	42206	7	53	19	20	1	0	0	0
		Sevuparin/DF02+ Malanil	D1H0	Pf	33366	4	58	28	10	0	0	0	0
		Sevuparin/DF02+ Malanil	D1H1	Pf	29590	29	53	11	7	0	0	0	0
		Sevuparin/DF02+ Malanil	D1H2	Pf	23780	26	49	17	7	1	0	0	0
		Sevuparin/DF02+ Malanil	D1H3	Pf	23033	59	31	6	4	0	0	0	0
		Sevuparin/DF02+ Malanil	D1H4	Pf	28573	48	38	4	6	4	0	0	0
		Sevuparin/DF02+ Malanil	D1H6	Pf	28680	26	46	17	11	0	0	0	0
		Sevuparin/DF02+ Malanil	D1H8	Pf	23883	21	37	27	15	0	0	0	0

SUBJID	INITIAL	ARM	TIMEPOINT	SPECIES	PARASITAEMIA (/µL)	Tiny Rings (%)	Small Rings (%)	Large Rings (%)	Early Troph (%)	Mid Troph (%)	Late Troph (%)	Gamet (%)	Schiz (%)
		Sevuparin/DF02+ Malanil	D1H10	Pf	24444	9	35	30	26	0	0	0	0
		Sevuparin/DF02+ Malanil	D1H11	Pf	23448	12	44	19	22	3	0	0	0
		Sevuparin/DF02+ Malanil	D1H17	Pf	19837	3	10	20	49	17	1	0	0
		Sevuparin/DF02+ Malanil	D1H23	Pf	17140	0	6	18	58	12	6	0	0
		Sevuparin/DF02+ Malanil	D1H29	Pf	5850	0	4	8	63	13	12	0	0
		Sevuparin/DF02+ Malanil	D2H35	Pf	943	ND	ND	ND	ND	ND	ND	ND	ND
		Sevuparin/DF02+ Malanil	D2H41	Pf	244	ND	ND	ND	ND	ND	ND	ND	ND
		Sevuparin/DF02+ Malanil	D3H47	Pf	114	ND	ND	ND	ND	ND	ND	ND	ND
		Sevuparin/DF02+ Malanil	D3H53	Pf	19	ND	ND	ND	ND	ND	ND	ND	ND
		Sevuparin/DF02+ Malanil	D3H59	NEGATIVE	0	ND	ND	ND	ND	ND	ND	ND	ND
		Sevuparin/DF02+ Malanil	D3H65	NEGATIVE	0	ND	ND	ND	ND	ND	ND	ND	ND
		Sevuparin/DF02+ Malanil	D3H71	NEGATIVE	0	ND	ND	ND	ND	ND	ND	ND	ND
		Sevuparin/DF02+ Malanil	D7	NEGATIVE	0	ND	ND	ND	ND	ND	ND	ND	ND
		Sevuparin/DF02+	D14	NEGATIVE	0	ND	ND	ND	ND	ND	ND	ND	ND

SUBJID	INITIAL	ARM	TIMEPOINT	SPECIES	PARASITAEMIA (/µL)	Tiny Rings (%)	Small Rings (%)	Large Rings (%)	Early Troph (%)	Mid Troph (%)	Late Troph (%)	Gamet (%)	Schiz (%)
		Malanil											
		Sevuparin/DF02+ Malanil	SCREENING	Pf	68250	64	32	2	2	0	0	0	0
		Sevuparin/DF02+ Malanil	D1H0	Pf	99750	83	15	0	2	0	0	0	0
		Sevuparin/DF02+ Malanil	D1H1	Pf	105000	79	17	2	2	0	0	0	0
		Sevuparin/DF02+ Malanil	D1H2	Pf	94500	67	29	2	2	0	0	0	0
		Sevuparin/DF02+ Malanil	D1H3	Pf	99750	59	30	9	2	0	0	0	0
		Sevuparin/DF02+ Malanil	D1H4	Pf	94500	77	15	2	6	0	0	0	0
		Sevuparin/DF02+ Malanil	D1H6	Pf	94500	76	18	2	4	0	0	0	0
		Sevuparin/DF02+ Malanil	D1H8	Pf	94500	61	24	2	13	0	0	0	0
		Sevuparin/DF02+ Malanil	D1H10	Pf	89250	68	23	2	7	0	0	0	0
		Sevuparin/DF02+ Malanil	D1H11	Pf	89250	54	33	9	4	0	0	0	0
		Sevuparin/DF02+ Malanil	D1H17	Pf	73500	22	28	19	26	5	0	0	0
		Sevuparin/DF02+ Malanil	D1H23	Pf	52500	6	18	20	50	6	0	0	0
		Sevuparin/DF02+ Malanil	D1H29	Pf	15208	ND	ND	ND	ND	ND	ND	ND	ND

SUBJID	INITIAL	ARM	TIMEPOINT	SPECIES	PARASITAEMIA (/µL)	Tiny Rings (%)	Small Rings (%)	Large Rings (%)	Early Troph (%)	Mid Troph (%)	Late Troph (%)	Gamet (%)	Schiz (%)
		Sevuparin/DF02+ Malanil	D2H35	Pf	3282	ND	ND	ND	ND	ND	ND	ND	ND
		Sevuparin/DF02+ Malanil	D2H41	Pf	1780	ND	ND	ND	ND	ND	ND	ND	ND
		Sevuparin/DF02+ Malanil	D3H47	Pf	858	ND	ND	ND	ND	ND	ND	ND	ND
		Sevuparin/DF02+ Malanil	D3H53	Pf	601	ND	ND	ND	ND	ND	ND	ND	ND
		Sevuparin/DF02+ Malanil	D3H59	Pf	65	ND	ND	ND	ND	ND	ND	ND	ND
		Sevuparin/DF02+ Malanil	D3H65	Pf	22	ND	ND	ND	ND	ND	ND	ND	ND
		Sevuparin/DF02+ Malanil	D3H71	NEGATIVE	0	ND	ND	ND	ND	ND	ND	ND	ND
		Sevuparin/DF02+ Malanil	D4	NEGATIVE	0	ND	ND	ND	ND	ND	ND	ND	ND
		Sevuparin/DF02+ Malanil	D7	NEGATIVE	0	ND	ND	ND	ND	ND	ND	ND	ND
		Sevuparin/DF02+ Malanil	D14	NEGATIVE	0	ND	ND	ND	ND	ND	ND	ND	ND
		Malanil	SCREENING	Pf	37216	26	30	24	19	1	0	0	0
		Malanil	D1H0	Pf	36089	19	33	28	20	0	0	0	0
		Malanil	D1H1	Pf	41973	9	39	30	21	1	0	0	0
		Malanil	D1H2	Pf	31425	19	36	21	23	1	0	0	0
		Malanil	D1H3	Pf	33428	9	35	24	24	6	2	0	0
		Malanil	D1H4	Pf	38436	11	31	24	26	7	1	0	0

SUBJID	INITIAL	ARM	TIMEPOINT	SPECIES	PARASITAEMIA (/µL)	Tiny Rings (%)	Small Rings (%)	Large Rings (%)	Early Troph (%)	Mid Troph (%)	Late Troph (%)	Gamet (%)	Schiz (%)
		Malanil	D1H6	Pf	33929	19	33	19	23	5	1	0	0
		Malanil	D1H8	Pf	28733	9	26	18	30	11	6	0	0
		Malanil	D1H10	Pf	20408	12	30	17	27	10	4	0	0
		Malanil	D1H11	Pf	14523	12	23	18	25	13	9	0	0
		Malanil	D1H17	Pf	4883	17	28	16	21	14	4	0	0
		Malanil	D1H23	Pf	1967	12	36	12	32	4	4	0	0
		Malanil	D1H29	Pf	1650	ND	ND	ND	ND	ND	ND	ND	ND
		Malanil	D2H35	Pf	698	ND	ND	ND	ND	ND	ND	ND	ND
		Malanil	D2H41	Pf	338	ND	ND	ND	ND	ND	ND	ND	ND
		Malanil	D3H47	Pf	64	ND	ND	ND	ND	ND	ND	ND	ND
		Malanil	D3H53	Pf	21	ND	ND	ND	ND	ND	ND	ND	ND
		Malanil	D3H59	NEGATIVE	0	ND	ND	ND	ND	ND	ND	ND	ND
		Malanil	D3H65	NEGATIVE	0	ND	ND	ND	ND	ND	ND	ND	ND
		Malanil	D7	NEGATIVE	0	ND	ND	ND	ND	ND	ND	ND	ND
		Malanil	D14	NEGATIVE	0	ND	ND	ND	ND	ND	ND	ND	ND
		Sevuparin/DF02+ Malanil	SCREENING	Pf	71540	72	26	2	0	0	0	0	0
		Sevuparin/DF02+ Malanil	D1H0	Pf	91980	72	24	4	0	0	0	0	0
		Sevuparin/DF02+ Malanil	D1H1	Pf	132860	45	37	12	5	1	0	0	0
		Sevuparin/DF02+ Malanil	D1H2	Pf	102200	65	27	6	2	0	0	0	0
		Sevuparin/DF02+	D1H3	Pf	76650	61	25	9	5	0	0	0	0

SUBJID	INITIAL	ARM	TIMEPOINT	SPECIES	PARASITAEMIA (/µL)	Tiny Rings (%)	Small Rings (%)	Large Rings (%)	Early Troph (%)	Mid Troph (%)	Late Troph (%)	Gamet (%)	Schiz (%)
		Malanil											
		Sevuparin/DF02+ Malanil	D1H4	Pf	91980	52	28	13	7	0	0	0	0
		Sevuparin/DF02+ Malanil	D1H6	Pf	86870	40	44	12	4	0	0	0	0
		Sevuparin/DF02+ Malanil	D1H8	Pf	71540	26	35	24	15	0	0	0	0
		Sevuparin/DF02+ Malanil	D1H10	Pf	61320	24	46	16	14	0	0	0	0
		Sevuparin/DF02+ Malanil	D1H11	Pf	56210	14	53	21	12	0	0	0	0
		Sevuparin/DF02+ Malanil	D1H17	Pf	27239	4	37	27	32	0	0	0	0
		Sevuparin/DF02+ Malanil	D1H23	Pf	11554	8	21	33	38	0	0	0	0
		Sevuparin/DF02+ Malanil	D1H29	Pf	10154	0	15	17	60	8	0	0	0
		Sevuparin/DF02+ Malanil	D2H35	Pf	2532	4	10	29	43	14	0	0	0
		Sevuparin/DF02+ Malanil	D2H41	Pf	666	ND	ND	ND	ND	ND	ND	ND	ND
		Sevuparin/DF02+ Malanil	D3H47	Pf	133	ND	ND	ND	ND	ND	ND	ND	ND
		Sevuparin/DF02+ Malanil	D3H53	Pf	111	ND	ND	ND	ND	ND	ND	ND	ND
		Sevuparin/DF02+ Malanil	D3H59	Pf	28	ND	ND	ND	ND	ND	ND	ND	ND

SUBJID	INITIAL	ARM	TIMEPOINT	SPECIES	PARASITAEMIA (/µL)	Tiny Rings (%)	Small Rings (%)	Large Rings (%)	Early Troph (%)	Mid Troph (%)	Late Troph (%)	Gamet (%)	Schiz (%)
		Sevuparin/DF02+ Malanil	D3H65	NEGATIVE	0	ND	ND	ND	ND	ND	ND	ND	ND
		Sevuparin/DF02+ Malanil	D3H71	NEGATIVE	0	ND	ND	ND	ND	ND	ND	ND	ND
		Sevuparin/DF02+ Malanil	D7	NEGATIVE	0	ND	ND	ND	ND	ND	ND	ND	ND
		Sevuparin/DF02+ Malanil	D14	NEGATIVE	0	ND	ND	ND	ND	ND	ND	ND	ND
		Sevuparin/DF02+ Malanil	SCREENING	Pf	34019	18	20	7	36	11	8	0	0
		Sevuparin/DF02+ Malanil	D1H0	Pf	17827	20	32	13	16	11	8	0	0
		Sevuparin/DF02+ Malanil	D1H1	Pf	15280	3	14	14	29	18	22	0	0
		Sevuparin/DF02+ Malanil	D1H2	Pf	13790	2	17	20	24	19	18	0	0
		Sevuparin/DF02+ Malanil	D1H3	Pf	12781	8	18	19	26	14	15	0	0
		Sevuparin/DF02+ Malanil	D1H4	Pf	12733	10	19	11	27	16	17	0	0
		Sevuparin/DF02+ Malanil	D1H6	Pf	9178	6	21	6	40	12	15	0	0
		Sevuparin/DF02+ Malanil	D1H8	Pf	5334	10	10	25	40	15	0	0	0
		Sevuparin/DF02+ Malanil	D1H10	Pf	5189	ND	ND	ND	ND	ND	ND	ND	ND
		Sevuparin/DF02+	D1H11	Pf	4805	ND	ND	ND	ND	ND	ND	ND	ND

SUBJID	INITIAL	ARM	TIMEPOINT	SPECIES	PARASITAEMIA (/µL)	Tiny Rings (%)	Small Rings (%)	Large Rings (%)	Early Troph (%)	Mid Troph (%)	Late Troph (%)	Gamet (%)	Schiz (%)
		Malanil											
		Sevuparin/DF02+ Malanil	D1H17	Pf	2114	ND	ND	ND	ND	ND	ND	ND	ND
		Sevuparin/DF02+ Malanil	D1H23	Pf	1057	ND	ND	ND	ND	ND	ND	ND	ND
		Sevuparin/DF02+ Malanil	D1H29	Pf	400	ND	ND	ND	ND	ND	ND	ND	ND
		Sevuparin/DF02+ Malanil	D2H35	Pf	86	ND	ND	ND	ND	ND	ND	ND	ND
		Sevuparin/DF02+ Malanil	D2H41	Pf	57	ND	ND	ND	ND	ND	ND	ND	ND
		Sevuparin/DF02+ Malanil	D3H47	Pf	29	ND	ND	ND	ND	ND	ND	ND	ND
		Sevuparin/DF02+ Malanil	D3H53	NEGATIVE	0	ND	ND	ND	ND	ND	ND	ND	ND
		Sevuparin/DF02+ Malanil	D3H59	NEGATIVE	0	ND	ND	ND	ND	ND	ND	ND	ND
		Sevuparin/DF02+ Malanil	D3H65	NEGATIVE	0	ND	ND	ND	ND	ND	ND	ND	ND
		Sevuparin/DF02+ Malanil	D3H71	NEGATIVE	0	ND	ND	ND	ND	ND	ND	ND	ND
		Sevuparin/DF02+ Malanil	D7	Pf	0	ND	ND	ND	ND	ND	ND	ND	ND
		Sevuparin/DF02+ Malanil	D14	Pf	0	ND	ND	ND	ND	ND	ND	ND	ND
		Sevuparin/DF02+ Malanil	SCREENING	Pf	23629	47	41	9	3	0	0	0	0

SUBJID	INITIAL	ARM	TIMEPOINT	SPECIES	PARASITAEMIA (/µL)	Tiny Rings (%)	Small Rings (%)	Large Rings (%)	Early Troph (%)	Mid Troph (%)	Late Troph (%)	Gamet (%)	Schiz (%)
		Sevuparin/DF02+ Malanil	D1H0	Pf	22630	49	31	13	6	1	0	0	0
		Sevuparin/DF02+ Malanil	D1H1	Pf	23130	45	39	9	7	0	0	0	0
		Sevuparin/DF02+ Malanil	D1H2	Pf	31158	40	23	21	16	0	0	0	0
		Sevuparin/DF02+ Malanil	D1H3	Pf	30451	57	32	7	3	1	0	0	0
		Sevuparin/DF02+ Malanil	D1H4	Pf	30534	31	35	20	13	1	0	0	0
		Sevuparin/DF02+ Malanil	D1H6	Pf	27498	40	36	21	3	0	0	0	0
		Sevuparin/DF02+ Malanil	D1H8	Pf	20134	38	32	16	12	2	0	0	0
		Sevuparin/DF02+ Malanil	D1H10	Pf	19552	17	47	28	8	0	0	0	0
		Sevuparin/DF02+ Malanil	D1H11	Pf	18179	30	28	23	17	1	1	0	0
		Sevuparin/DF02+ Malanil	D1H17	Pf	14310	30	13	9	44	4	0	0	0
		Sevuparin/DF02+ Malanil	D1H23	Pf	5824	ND	ND	ND	ND	ND	ND	ND	ND
		Sevuparin/DF02+ Malanil	D1H29	Pf	1155	ND	ND	ND	ND	ND	ND	ND	ND
		Sevuparin/DF02+ Malanil	D2H35	Pf	350	ND	ND	ND	ND	ND	ND	ND	ND
		Sevuparin/DF02+	D2H41	Pf	105	ND	ND	ND	ND	ND	ND	ND	ND

SUBJID	INITIAL	ARM	TIMEPOINT	SPECIES	PARASITAEMIA (/µL)	Tiny Rings (%)	Small Rings (%)	Large Rings (%)	Early Troph (%)	Mid Troph (%)	Late Troph (%)	Gamet (%)	Schiz (%)
		Malanil											
		Sevuparin/DF02+ Malanil	D3H47	NEGATIVE	0	ND	ND	ND	ND	ND	ND	ND	ND
		Sevuparin/DF02+ Malanil	D3H53	NEGATIVE	0	ND	ND	ND	ND	ND	ND	ND	ND
		Sevuparin/DF02+ Malanil	D3H59	NEGATIVE	0	ND	ND	ND	ND	ND	ND	ND	ND
		Sevuparin/DF02+ Malanil	D3H65	NEGATIVE	0	ND	ND	ND	ND	ND	ND	ND	ND
		Sevuparin/DF02+ Malanil	D7	NEGATIVE	0	ND	ND	ND	ND	ND	ND	ND	ND
		Sevuparin/DF02+ Malanil	D14	NEGATIVE	0	ND	ND	ND	ND	ND	ND	ND	ND
		Sevuparin/DF02+ Malanil	SCREENING	Pf	22200	6	15	29	28	15	7	0	0
		Sevuparin/DF02+ Malanil	D1H0	Pf	10212	23	33	10	27	7	0	0	0
		Sevuparin/DF02+ Malanil	D1H1	Pf	7326	12	17	12	27	12	20	0	0
		Sevuparin/DF02+ Malanil	D1H2	Pf	5957	6	17	10	30	10	27	0	0
		Sevuparin/DF02+ Malanil	D1H3	Pf	6327	7	28	30	21	9	5	0	0
		Sevuparin/DF02+ Malanil	D1H4	Pf	5513	14	27	18	31	2	8	0	0
		Sevuparin/DF02+ Malanil	D1H6	Pf	4995	ND	ND	ND	ND	ND	ND	ND	ND

TSM02 Individual subject listings part 2

CSR Appendix 16.2

SUBJID	INITIAL	ARM	TIMEPOINT	SPECIES	PARASITAEMIA (/µL)	Tiny Rings (%)	Small Rings (%)	Large Rings (%)	Early Troph (%)	Mid Troph (%)	Late Troph (%)	Gamet (%)	Schiz (%)
		Sevuparin/DF02+ Malanil	D1H8	Pf	3256	ND	ND	ND	ND	ND	ND	ND	ND
		Sevuparin/DF02+ Malanil	D1H10	Pf	2701	ND	ND	ND	ND	ND	ND	ND	ND
		Sevuparin/DF02+ Malanil	D1H11	Pf	2590	ND	ND	ND	ND	ND	ND	ND	ND
		Sevuparin/DF02+ Malanil	D1H17	Pf	1221	ND	ND	ND	ND	ND	ND	ND	ND
		Sevuparin/DF02+ Malanil	D1H23	Pf	925	ND	ND	ND	ND	ND	ND	ND	ND
		Sevuparin/DF02+ Malanil	D1H29	Pf	210	ND	ND	ND	ND	ND	ND	ND	ND
		Sevuparin/DF02+ Malanil	D2H35	Pf	70	ND	ND	ND	ND	ND	ND	ND	ND
		Sevuparin/DF02+ Malanil	D2H41	NEGATIVE	0	ND	ND	ND	ND	ND	ND	ND	ND
		Sevuparin/DF02+ Malanil	D3H47	NEGATIVE	0	ND	ND	ND	ND	ND	ND	ND	ND
		Sevuparin/DF02+ Malanil	D3H53	NEGATIVE	0	ND	ND	ND	ND	ND	ND	ND	ND
		Sevuparin/DF02+ Malanil	D3H59	NEGATIVE	0	ND	ND	ND	ND	ND	ND	ND	ND
		Sevuparin/DF02+ Malanil	D3H65	NEGATIVE	0	ND	ND	ND	ND	ND	ND	ND	ND
		Sevuparin/DF02+ Malanil	D3H71	NEGATIVE	0	ND	ND	ND	ND	ND	ND	ND	ND
		Sevuparin/DF02+	D7	NEGATIVE	0	ND	ND	ND	ND	ND	ND	ND	ND

SUBJID	INITIAL	ARM	TIMEPOINT	SPECIES	PARASITAEMIA (/µL)	Tiny Rings (%)	Small Rings (%)	Large Rings (%)	Early Troph (%)	Mid Troph (%)	Late Troph (%)	Gamet (%)	Schiz (%)
		Malanil											
		Sevuparin/DF02+ Malanil	D14	NEGATIVE	0	ND	ND	ND	ND	ND	ND	ND	ND
		Malanil	SCREENING	Pf	69300	24	32	25	18	1	0	0	0
		Malanil	D1H0	Pf	69300	36	33	21	10	0	0	0	0
		Malanil	D1H1	Pf	64680	42	28	19	11	0	0	0	0
		Malanil	D1H2	Pf	73920	18	33	26	23	0	0	0	0
		Malanil	D1H3	Pf	78540	33	38	19	10	0	0	0	0
		Malanil	D1H4	Pf	64680	12	35	20	27	6	0	0	0
		Malanil	D1H6	Pf	60060	16	31	15	30	8	0	0	0
		Malanil	D1H8	Pf	50820	13	32	20	28	7	0	0	0
		Malanil	D1H10	Pf	41580	11	32	34	21	2	0	0	0
		Malanil	D1H11	Pf	30845	4	25	30	36	5	0	0	0
		Malanil	D1H17	Pf	14851	7	16	32	45	0	0	0	0
		Malanil	D1H23	Pf	8472	10	12	30	42	6	0	0	0
		Malanil	D1H29	Pf	2472	0	11	22	33	30	4	0	0
		Malanil	D2H35	Pf	603	ND	ND	ND	ND	ND	ND	ND	ND
		Malanil	D2H41	Pf	181	ND	ND	ND	ND	ND	ND	ND	ND
		Malanil	D3H47	NEGATIVE	0	ND	ND	ND	ND	ND	ND	ND	ND
		Malanil	D3H53	NEGATIVE	0	ND	ND	ND	ND	ND	ND	ND	ND
		Malanil	D3H59	NEGATIVE	0	ND	ND	ND	ND	ND	ND	ND	ND
		Malanil	D3H65	NEGATIVE	0	ND	ND	ND	ND	ND	ND	ND	ND
		Malanil	D3H71	NEGATIVE	0	ND	ND	ND	ND	ND	ND	ND	ND

SUBJID	INITIAL	ARM	TIMEPOINT	SPECIES	PARASITAEMIA (/µL)	Tiny Rings (%)	Small Rings (%)	Large Rings (%)	Early Troph (%)	Mid Troph (%)	Late Troph (%)	Gamet (%)	Schiz (%)
		Malanil	D7	NEGATIVE	0	ND	ND	ND	ND	ND	ND	ND	ND
		Malanil	D14	NEGATIVE	0	ND	ND	ND	ND	ND	ND	ND	ND
		Malanil	SCREENING	Pf	96200	0	0	4	95	1	0	0	0
		Malanil	D1H0	Pf	96200	0	1	4	80	15	0	0	0
		Malanil	D1H1	Pf	91390	0	2	1	63	33	1	0	0
		Malanil	D1H2	Pf	86580	3	7	1	39	41	9	0	0
		Malanil	D1H3	Pf	62530	5	8	3	24	45	15	0	0
		Malanil	D1H4	Pf	11932	13	13	2	20	34	18	0	0
		Malanil	D1H6	Pf	4563	22	29	17	20	11	0	1	0
		Malanil	D1H8	Pf	3904	21	25	17	19	11	7	0	0
		Malanil	D1H10	Pf	2733	40	29	11	8	6	6	0	0
		Malanil	D1H11	Pf	2318	37	27	27	7	0	2	0	0
		Malanil	D1H17	Pf	2050	18	41	23	18	0	0	0	0
		Malanil	D1H23	Pf	1996	0	12	0	18	70	0	0	0
		Malanil	D1H29	Pf	1971	15	15	8	46	8	8	0	0
		Malanil	D2H35	Pf	1322	ND	ND	ND	ND	ND	ND	ND	ND
		Malanil	D2H41	Pf	175	ND	ND	ND	ND	ND	ND	ND	ND
		Malanil	D3H47	Pf	50	ND	ND	ND	ND	ND	ND	ND	ND
		Malanil	D3H53	NEGATIVE	0	ND	ND	ND	ND	ND	ND	ND	ND
		Malanil	D3H59	NEGATIVE	0	ND	ND	ND	ND	ND	ND	ND	ND
		Malanil	D3H65	NEGATIVE	0	ND	ND	ND	ND	ND	ND	ND	ND
		Malanil	D3H71	NEGATIVE	0	ND	ND	ND	ND	ND	ND	ND	ND

SUBJID	INITIAL	ARM	TIMEPOINT	SPECIES	PARASITAEMIA (/µL)	Tiny Rings (%)	Small Rings (%)	Large Rings (%)	Early Troph (%)	Mid Troph (%)	Late Troph (%)	Gamet (%)	Schiz (%)
		Malanil	D7	NEGATIVE	0	ND	ND	ND	ND	ND	ND	ND	ND
		Malanil	D14	NEGATIVE	0	ND	ND	ND	ND	ND	ND	ND	ND
		Malanil	SCREENING	Pf	17913	47	36	7	7	3	0	0	0
		Malanil	D1H0	Pf	20458	77	18	2	2	1	0	0	0
		Malanil	D1H1	Pf	22408	77	18	3	2	0	0	0	0
		Malanil	D1H2	Pf	22011	64	31	4	0	1	0	0	0
		Malanil	D1H3	Pf	22805	44	39	15	2	0	0	0	0
		Malanil	D1H4	Pf	25944	43	35	16	6	0	0	0	0
		Malanil	D1H6	Pf	19400	49	34	11	6	0	0	0	0
		Malanil	D1H8	Pf	19632	35	50	10	5	0	0	0	0
		Malanil	D1H10	Pf	17517	29	54	13	3	1	0	0	0
		Malanil	D1H11	Pf	20921	22	39	23	15	1	0	0	0
		Malanil	D1H17	Pf	22639	13	24	38	25	0	0	0	0
		Malanil	D1H23	Pf	8780	0	4	35	59	2	0	0	0
		Malanil	D1H29	Pf	8736	0	14	15	57	14	0	0	0
		Malanil	D2H35	Pf	5992	ND	ND	ND	ND	ND	ND	ND	ND
		Malanil	D2H41	Pf	3578	ND	ND	ND	ND	ND	ND	ND	ND
		Malanil	D3H47	Pf	758	ND	ND	ND	ND	ND	ND	ND	ND
		Malanil	D3H53	Pf	308	ND	ND	ND	ND	ND	ND	ND	ND
		Malanil	D3H59	Pf	71	ND	ND	ND	ND	ND	ND	ND	ND
		Malanil	D3H65	NEGATIVE	0	ND	ND	ND	ND	ND	ND	ND	ND
		Malanil	D3H71	NEGATIVE	0	ND	ND	ND	ND	ND	ND	ND	ND

SUBJID	INITIAL	ARM	TIMEPOINT	SPECIES	PARASITAEMIA (/µL)	Tiny Rings (%)	Small Rings (%)	Large Rings (%)	Early Troph (%)	Mid Troph (%)	Late Troph (%)	Gamet (%)	Schiz (%)
		Malanil	D7	NEGATIVE	0	ND	ND	ND	ND	ND	ND	ND	ND
		Malanil	D14	NEGATIVE	0	ND	ND	ND	ND	ND	ND	ND	ND
		Sevuparin/DF02+ Malanil	SCREENING	Pf	13431	21	25	19	28	5	1	1	0
		Sevuparin/DF02+ Malanil	D1H0	Pf	7407	41	24	16	15	1	1	2	0
		Sevuparin/DF02+ Malanil	D1H1	Pf	12678	19	30	17	25	8	1	0	0
		Sevuparin/DF02+ Malanil	D1H2	Pf	13777	28	25	15	21	5	3	3	0
		Sevuparin/DF02+ Malanil	D1H3	Pf	15507	18	26	20	24	8	2	2	0
		Sevuparin/DF02+ Malanil	D1H4	Pf	17298	12	22	17	35	8	6	0	0
		Sevuparin/DF02+ Malanil	D1H6	Pf	16382	4	28	20	27	15	4	2	0
		Sevuparin/DF02+ Malanil	D1H8	Pf	13431	11	22	20	30	5	10	2	0
		Sevuparin/DF02+ Malanil	D1H10	Pf	12373	3	21	15	35	13	10	3	0
		Sevuparin/DF02+ Malanil	D1H11	Pf	11966	0	6	20	46	19	6	2	1
		Sevuparin/DF02+ Malanil	D1H17	Pf	10094	0	7	7	49	17	17	3	0
		Sevuparin/DF02+ Malanil	D1H23	Pf	7977	4	8	3	34	31	10	10	0

SUBJID	INITIAL	ARM	TIMEPOINT	SPECIES	PARASITAEMIA (/µL)	Tiny Rings (%)	Small Rings (%)	Large Rings (%)	Early Troph (%)	Mid Troph (%)	Late Troph (%)	Gamet (%)	Schiz (%)
		Sevuparin/DF02+ Malanil	D1H29	Pf	2207	ND	ND	ND	ND	ND	ND	ND	ND
		Sevuparin/DF02+ Malanil	D2H35	Pf	1095	ND	ND	ND	ND	ND	ND	ND	ND
		Sevuparin/DF02+ Malanil	D2H41	Pf	343	ND	ND	ND	ND	ND	ND	ND	ND
		Sevuparin/DF02+ Malanil	D3H47	Pf	82	ND	ND	ND	ND	ND	ND	ND	ND
		Sevuparin/DF02+ Malanil	D3H53	Pf	0	ND	ND	ND	ND	ND	ND	ND	ND
		Sevuparin/DF02+ Malanil	D3H59	Pf	0	ND	ND	ND	ND	ND	ND	ND	ND
		Sevuparin/DF02+ Malanil	D3H65	Pf	0	ND	ND	ND	ND	ND	ND	ND	ND
		Sevuparin/DF02+ Malanil	D3H71	Pf	0	ND	ND	ND	ND	ND	ND	ND	ND
		Sevuparin/DF02+ Malanil	D7	Pf	0	ND	ND	ND	ND	ND	ND	ND	ND
		Sevuparin/DF02+ Malanil	D14	Pf	0	ND	ND	ND	ND	ND	ND	ND	ND
		Malanil	SCREENING	Pf	96600	33	34	19	14	0	0	0	0
		Malanil	D1H0	Pf	96600	27	36	17	17	3	0	0	0
		Malanil	D1H1	Pf	106260	27	40	23	10	0	0	0	0
		Malanil	D1H2	Pf	111090	9	36	30	25	0	0	0	0
		Malanil	D1H3	Pf	111090	7	46	25	19	3	0	0	0
		Malanil	D1H4	Pf	106260	18	44	15	22	1	0	0	0

SUBJID	INITIAL	ARM	TIMEPOINT	SPECIES	PARASITAEMIA (/µL)	Tiny Rings (%)	Small Rings (%)	Large Rings (%)	Early Troph (%)	Mid Troph (%)	Late Troph (%)	Gamet (%)	Schiz (%)
		Malanil	D1H6	Pf	106260	14	39	15	30	2	0	0	0
		Malanil	D1H8	Pf	96600	3	30	20	41	4	2	0	0
		Malanil	D1H10	Pf	82110	5	35	15	36	7	2	0	0
		Malanil	D1H11	Pf	77280	3	33	18	32	12	2	0	0
		Malanil	D1H17	Pf	67620	1	18	25	41	11	4	0	0
		Malanil	D1H23	Pf	60000	3	13	18	41	13	12	0	0
		Malanil	D1H29	Pf	50000	0	21	17	38	15	9	0	0
		Malanil	D2H35	Pf	15369	3	20	31	31	6	9	0	0
		Malanil	D2H41	Pf	8162	ND	ND	ND	ND	ND	ND	ND	ND
		Malanil	D3H47	Pf	2767	ND	ND	ND	ND	ND	ND	ND	ND
		Malanil	D3H53	Pf	2172	ND	ND	ND	ND	ND	ND	ND	ND
		Malanil	D3H59	Pf	1577	ND	ND	ND	ND	ND	ND	ND	ND
		Malanil	D3H65	Pf	446	ND	ND	ND	ND	ND	ND	ND	ND
		Malanil	D3H71	Pf	179	ND	ND	ND	ND	ND	ND	ND	ND
		Malanil	D4	Pf	30	ND	ND	ND	ND	ND	ND	ND	ND
		Malanil	D5	NEGATIVE	0	ND	ND	ND	ND	ND	ND	ND	ND
		Malanil	D6	NEGATIVE	0	ND	ND	ND	ND	ND	ND	ND	ND
		Malanil	D7	NEGATIVE	0	ND	ND	ND	ND	ND	ND	ND	ND
		Malanil	D14	NEGATIVE	0	ND	ND	ND	ND	ND	ND	ND	ND
		Malanil	SCREENING	Pf	10709	4	11	6	43	26	10	0	0
		Malanil	D1H0	Pf	7381	6	13	5	37	26	13	0	0
		Malanil	D1H1	Pf	3812	4	12	2	46	22	14	0	0

SUBJID	INITIAL	ARM	TIMEPOINT	SPECIES	PARASITAEMIA (/µL)	Tiny Rings (%)	Small Rings (%)	Large Rings (%)	Early Troph (%)	Mid Troph (%)	Late Troph (%)	Gamet (%)	Schiz (%)
		Malanil	D1H2	Pf	2148	ND	ND	ND	ND	ND	ND	ND	ND
		Malanil	D1H3	Pf	1664	ND	ND	ND	ND	ND	ND	ND	ND
		Malanil	D1H4	Pf	1240	ND	ND	ND	ND	ND	ND	ND	ND
		Malanil	D1H6	Pf	908	ND	ND	ND	ND	ND	ND	ND	ND
		Malanil	D1H8	Pf	666	ND	ND	ND	ND	ND	ND	ND	ND
		Malanil	D1H10	Pf	424	ND	ND	ND	ND	ND	ND	ND	ND
		Malanil	D1H11	Pf	272	ND	ND	ND	ND	ND	ND	ND	ND
		Malanil	D1H17	Pf	121	ND	ND	ND	ND	ND	ND	ND	ND
		Malanil	D1H23	Pf	51	ND	ND	ND	ND	ND	ND	ND	ND
		Malanil	D1H29	Pf	34	ND	ND	ND	ND	ND	ND	ND	ND
		Malanil	D2H35	Pf	17	ND	ND	ND	ND	ND	ND	ND	ND
		Malanil	D2H41	Pf	17	ND	ND	ND	ND	ND	ND	ND	ND
		Malanil	D3H47	NEGATIVE	0	ND	ND	ND	ND	ND	ND	ND	ND
		Malanil	D3H53	NEGATIVE	0	ND	ND	ND	ND	ND	ND	ND	ND
		Malanil	D7	NEGATIVE	0	ND	ND	ND	ND	ND	ND	ND	ND
		Malanil	D14	NEGATIVE	0	ND	ND	ND	ND	ND	ND	ND	ND
		Malanil	SCREENING	Pf	10108	0	1	14	42	18	25	0	0
		Malanil	D1H0	Pf	10840	0	1	0	16	34	49	0	0
		Malanil	D1H1	Pf	9476	0	3	4	12	30	51	0	0
		Malanil	D1H2	Pf	5985	6	17	6	7	20	44	0	0
		Malanil	D1H3	Pf	4954	25	19	9	6	16	25	0	0
		Malanil	D1H4	Pf	4489	45	39	0	3	7	6	0	0

SUBJID	INITIAL	ARM	TIMEPOINT	SPECIES	PARASITAEMIA (/µL)	Tiny Rings (%)	Small Rings (%)	Large Rings (%)	Early Troph (%)	Mid Troph (%)	Late Troph (%)	Gamet (%)	Schiz (%)
		Malanil	D1H6	Pf	4921	63	29	3	0	5	0	0	0
		Malanil	D1H8	Pf	5586	48	44	8	0	0	0	0	0
		Malanil	D1H10	Pf	5353	30	50	10	10	0	0	0	0
		Malanil	D1H11	Pf	3325	ND	ND	ND	ND	ND	ND	ND	ND
		Malanil	D1H17	Pf	2627	ND	ND	ND	ND	ND	ND	ND	ND
		Malanil	D1H23	Pf	1136	ND	ND	ND	ND	ND	ND	ND	ND
		Malanil	D1H29	Pf	814	ND	ND	ND	ND	ND	ND	ND	ND
		Malanil	D2H35	Pf	476	ND	ND	ND	ND	ND	ND	ND	ND
		Malanil	D2H41	Pf	230	ND	ND	ND	ND	ND	ND	ND	ND
		Malanil	D3H47	Pf	19	ND	ND	ND	ND	ND	ND	ND	ND
		Malanil	D3H53	NEGATIVE	0	ND	ND	ND	ND	ND	ND	ND	ND
		Malanil	D3H59	NEGATIVE	0	ND	ND	ND	ND	ND	ND	ND	ND
		Malanil	D7	NEGATIVE	0	ND	ND	ND	ND	ND	ND	ND	ND
		Malanil	D14	NEGATIVE	0	ND	ND	ND	ND	ND	ND	ND	ND
		Malanil	SCREENING	Pf	29388	71	26	1	1	1	0	0	0
		Malanil	D1H0	Pf	40409	56	33	9	1	1	0	0	0
		Malanil	D1H1	Pf	49895	61	35	3	1	0	0	0	0
		Malanil	D1H2	Pf	47849	61	29	9	1	0	0	0	0
		Malanil	D1H3	Pf	43106	53	43	4	0	0	0	0	0
		Malanil	D1H4	Pf	41618	60	27	10	3	0	0	0	0
		Malanil	D1H6	Pf	30039	27	40	26	7	0	0	0	0
		Malanil	D1H8	Pf	29528	12	46	20	22	0	0	0	0

SUBJID	INITIAL	ARM	TIMEPOINT	SPECIES	PARASITAEMIA (/µL)	Tiny Rings (%)	Small Rings (%)	Large Rings (%)	Early Troph (%)	Mid Troph (%)	Late Troph (%)	Gamet (%)	Schiz (%)
		Malanil	D1H10	Pf	23715	4	35	28	31	2	0	0	0
		Malanil	D1H11	Pf	21437	10	27	20	36	6	1	0	0
		Malanil	D1H17	Pf	20600	10	24	36	24	6	0	0	0
		Malanil	D1H23	Pf	5914	3	16	14	40	14	13	0	0
		Malanil	D1H29	Pf	3149	ND	ND	ND	ND	ND	ND	ND	ND
		Malanil	D2H35	Pf	435	ND	ND	ND	ND	ND	ND	ND	ND
		Malanil	D2H41	Pf	77	ND	ND	ND	ND	ND	ND	ND	ND
		Malanil	D3H47	Pf	54	ND	ND	ND	ND	ND	ND	ND	ND
		Malanil	D3H53	NEGATIVE	0	ND	ND	ND	ND	ND	ND	ND	ND
		Malanil	D3H59	NEGATIVE	0	ND	ND	ND	ND	ND	ND	ND	ND
		Malanil	D7	NEGATIVE	0	ND	ND	ND	ND	ND	ND	ND	ND
		Malanil	D14	NEGATIVE	0	ND	ND	ND	ND	ND	ND	ND	ND
		Sevuparin/DF02+ Malanil	SCREENING	Pf	11151	12	37	17	21	10	3	0	0
		Sevuparin/DF02+ Malanil	D1H0	Pf	12184	6	38	20	24	10	2	0	0
		Sevuparin/DF02+ Malanil	D1H1	Pf	16638	3	38	16	31	8	4	0	0
		Sevuparin/DF02+ Malanil	D1H2	Pf	15517	4	40	11	24	16	5	0	0
		Sevuparin/DF02+ Malanil	D1H3	Pf	13954	3	24	14	47	10	2	0	0
		Sevuparin/DF02+ Malanil	D1H4	Pf	13747	13	35	23	23	5	1	0	0

SUBJID	INITIAL	ARM	TIMEPOINT	SPECIES	PARASITAEMIA (/µL)	Tiny Rings (%)	Small Rings (%)	Large Rings (%)	Early Troph (%)	Mid Troph (%)	Late Troph (%)	Gamet (%)	Schiz (%)
		Sevuparin/DF02+ Malanil	D1H6	Pf	13629	2	13	9	39	25	12	0	0
		Sevuparin/DF02+ Malanil	D1H8	Pf	11918	5	21	12	40	17	5	0	0
		Sevuparin/DF02+ Malanil	D1H10	Pf	11771	6	15	20	37	13	9	0	0
		Sevuparin/DF02+ Malanil	D1H11	Pf	11535	6	13	9	40	21	11	0	0
		Sevuparin/DF02+ Malanil	D1H17	Pf	10207	3	9	12	39	20	17	0	0
		Sevuparin/DF02+ Malanil	D1H23	Pf	7818	0	15	14	36	21	14	0	0
		Sevuparin/DF02+ Malanil	D1H29	Pf	1993	0	31	6	44	6	13	0	0
		Sevuparin/DF02+ Malanil	D2H35	Pf	1072	ND	ND	ND	ND	ND	ND	ND	ND
		Sevuparin/DF02+ Malanil	D2H41	Pf	244	ND	ND	ND	ND	ND	ND	ND	ND
		Sevuparin/DF02+ Malanil	D3H47	Pf	169	ND	ND	ND	ND	ND	ND	ND	ND
		Sevuparin/DF02+ Malanil	D3H53	Pf	83	ND	ND	ND	ND	ND	ND	ND	ND
		Sevuparin/DF02+ Malanil	D3H59	Pf	41	ND	ND	ND	ND	ND	ND	ND	ND
		Sevuparin/DF02+ Malanil	D3H65	NEGATIVE	0	ND	ND	ND	ND	ND	ND	ND	ND
		Sevuparin/DF02+	D3H71	NEGATIVE	0	ND	ND	ND	ND	ND	ND	ND	ND

SUBJID	INITIAL	ARM	TIMEPOINT	SPECIES	PARASITAEMIA (/µL)	Tiny Rings (%)	Small Rings (%)	Large Rings (%)	Early Troph (%)	Mid Troph (%)	Late Troph (%)	Gamet (%)	Schiz (%)
		Malanil											
		Sevuparin/DF02+ Malanil	D7	NEGATIVE	0	ND	ND	ND	ND	ND	ND	ND	ND
		Sevuparin/DF02+ Malanil	D14	NEGATIVE	0	ND	ND	ND	ND	ND	ND	ND	ND
		Malanil	SCREENING	Pf	25275	73	26	1	0	0	0	0	0
		Malanil	D1H0	Pf	24390	67	29	4	0	0	0	0	0
		Malanil	D1H1	Pf	27990	71	23	5	1	0	0	0	0
		Malanil	D1H2	Pf	26235	65	26	8	1	0	0	0	0
		Malanil	D1H3	Pf	22260	56	30	9	5	0	0	0	0
		Malanil	D1H4	Pf	21990	46	33	15	6	0	0	0	0
		Malanil	D1H6	Pf	15660	51	30	15	4	0	0	0	0
		Malanil	D1H8	Pf	14985	8	24	31	35	2	0	0	0
		Malanil	D1H10	Pf	13980	19	42	23	16	0	0	0	0
		Malanil	D1H11	Pf	11595	5	31	30	34	0	0	0	0
		Malanil	D1H17	Pf	10335	3	21	21	48	7	0	0	0
		Malanil	D1H23	Pf	11063	0	7	14	51	21	7	0	0
		Malanil	D1H29	Pf	7659	0	8	12	41	29	10	0	0
		Malanil	D2H35	Pf	1472	ND	ND	ND	ND	ND	ND	ND	ND
		Malanil	D2H41	Pf	345	ND	ND	ND	ND	ND	ND	ND	ND
		Malanil	D3H47	Pf	33	ND	ND	ND	ND	ND	ND	ND	ND
		Malanil	D3H53	Pf	17	ND	ND	ND	ND	ND	ND	ND	ND
		Malanil	D3H59	NEGATIVE	0	ND	ND	ND	ND	ND	ND	ND	ND

SUBJID	INITIAL	ARM	TIMEPOINT	SPECIES	PARASITAEMIA (/µL)	Tiny Rings (%)	Small Rings (%)	Large Rings (%)	Early Troph (%)	Mid Troph (%)	Late Troph (%)	Gamet (%)	Schiz (%)
		Malanil	D3H65	NEGATIVE	0	ND	ND	ND	ND	ND	ND	ND	ND
		Malanil	D7	NEGATIVE	0	ND	ND	ND	ND	ND	ND	ND	ND
		Malanil	D14	NEGATIVE	0	ND	ND	ND	ND	ND	ND	ND	ND
		Malanil	SCREENING	Pf	10514	ND	ND	ND	ND	ND	ND	ND	ND
		Malanil	D1H0	Pf	6955	0	0	0	8	31	61	0	0
		Malanil	D1H1	Pf	5532	0	0	0	6	37	57	0	0
		Malanil	D1H2	Pf	3332	0	2	0	9	31	58	0	0
		Malanil	D1H3	Pf	1229	ND	ND	ND	ND	ND	ND	ND	ND
		Malanil	D1H4	Pf	938	ND	ND	ND	ND	ND	ND	ND	ND
		Malanil	D1H6	Pf	226	ND	ND	ND	ND	ND	ND	ND	ND
		Malanil	D1H8	Pf	97	ND	ND	ND	ND	ND	ND	ND	ND
		Malanil	D1H10	Pf	65	ND	ND	ND	ND	ND	ND	ND	ND
		Malanil	D1H11	Pf	32	ND	ND	ND	ND	ND	ND	ND	ND
		Malanil	D1H17	NEGATIVE	0	ND	ND	ND	ND	ND	ND	ND	ND
		Malanil	D1H23	NEGATIVE	0	ND	ND	ND	ND	ND	ND	ND	ND
		Malanil	D1H29	NEGATIVE	0	ND	ND	ND	ND	ND	ND	ND	ND
		Malanil	D2H35	NEGATIVE	0	ND	ND	ND	ND	ND	ND	ND	ND
		Malanil	D2H41	NEGATIVE	0	ND	ND	ND	ND	ND	ND	ND	ND
		Malanil	D3H47	NEGATIVE	0	ND	ND	ND	ND	ND	ND	ND	ND
		Malanil	D7	NEGATIVE	0	ND	ND	ND	ND	ND	ND	ND	ND
		Malanil	D14	NEGATIVE	0	ND	ND	ND	ND	ND	ND	ND	ND
		Malanil	SCREENING	Pf	32019	45	39	10	5	1	0	0	0

SUBJID	INITIAL	ARM	TIMEPOINT	SPECIES	PARASITAEMIA (/µL)	Tiny Rings (%)	Small Rings (%)	Large Rings (%)	Early Troph (%)	Mid Troph (%)	Late Troph (%)	Gamet (%)	Schiz (%)
		Malanil	D1H0	Pf	29910	59	32	5	4	0	0	0	0
		Malanil	D1H1	Pf	33472	58	27	11	4	0	0	0	0
		Malanil	D1H2	Pf	31124	47	37	10	5	1	0	0	0
		Malanil	D1H3	Pf	29651	47	33	13	7	0	0	0	0
		Malanil	D1H4	Pf	26905	34	40	21	5	0	0	0	0
		Malanil	D1H6	Pf	26427	13	36	24	25	2	0	0	0
		Malanil	D1H8	Pf	26069	20	33	18	24	4	1	0	0
		Malanil	D1H10	Pf	25790	4	20	22	47	7	0	0	0
		Malanil	D1H11	Pf	25353	6	25	24	38	5	2	0	0
		Malanil	D1H17	Pf	24477	10	21	19	40	7	3	0	0
		Malanil	D1H23	Pf	17458	4	9	10	27	31	19	0	0
		Malanil	D1H29	Pf	7363	0	0	0	48	35	17	0	0
		Malanil	D2H35	Pf	926	ND	ND	ND	ND	ND	ND	ND	ND
		Malanil	D2H41	Pf	204	ND	ND	ND	ND	ND	ND	ND	ND
		Malanil	D3H47	Pf	66	ND	ND	ND	ND	ND	ND	ND	ND
		Malanil	D3H53	NEGATIVE	0	ND	ND	ND	ND	ND	ND	ND	ND
		Malanil	D3H59	NEGATIVE	0	ND	ND	ND	ND	ND	ND	ND	ND
		Malanil	D3H65	NEGATIVE	0	ND	ND	ND	ND	ND	ND	ND	ND
		Malanil	D7	Pf	0	ND	ND	ND	ND	ND	ND	ND	ND
		Malanil	D14	Pf	0	ND	ND	ND	ND	ND	ND	ND	ND
		Sevuparin/DF02+ Malanil	SCREENING	Pf	14823	55	26	14	5	0	0	0	0
		Sevuparin/DF02+	D1H0	Pf	15972	41	27	24	8	0	0	0	0

SUBJID	INITIAL	ARM	TIMEPOINT	SPECIES	PARASITAEMIA (/µL)	Tiny Rings (%)	Small Rings (%)	Large Rings (%)	Early Troph (%)	Mid Troph (%)	Late Troph (%)	Gamet (%)	Schiz (%)
		Malanil											
		Sevuparin/DF02+ Malanil	D1H1	Pf	20661	15	27	20	29	6	3	0	0
		Sevuparin/DF02+ Malanil	D1H2	Pf	17999	12	27	25	28	6	2	0	0
		Sevuparin/DF02+ Malanil	D1H3	Pf	18967	7	16	30	39	6	2	0	0
		Sevuparin/DF02+ Malanil	D1H4	Pf	18906	8	22	23	33	12	2	0	0
		Sevuparin/DF02+ Malanil	D1H6	Pf	17787	0	16	31	44	6	3	0	0
		Sevuparin/DF02+ Malanil	D1H8	Pf	15579	0	4	13	45	19	19	0	0
		Sevuparin/DF02+ Malanil	D1H10	Pf	14339	0	4	3	45	34	14	0	0
		Sevuparin/DF02+ Malanil	D1H11	Pf	13098	2	0	0	9	32	57	0	0
		Sevuparin/DF02+ Malanil	D1H17	Pf	12221	1	0	0	9	26	64	0	0
		Sevuparin/DF02+ Malanil	D1H23	Pf	6534	0	11	0	28	22	39	0	0
		Sevuparin/DF02+ Malanil	D1H29	Pf	1898	ND	ND	ND	ND	ND	ND	ND	ND
		Sevuparin/DF02+ Malanil	D2H35	Pf	146	ND	ND	ND	ND	ND	ND	ND	ND
		Sevuparin/DF02+ Malanil	D2H41	NEGATIVE	0	ND	ND	ND	ND	ND	ND	ND	ND

SUBJID	INITIAL	ARM	TIMEPOINT	SPECIES	PARASITAEMIA (/µL)	Tiny Rings (%)	Small Rings (%)	Large Rings (%)	Early Troph (%)	Mid Troph (%)	Late Troph (%)	Gamet (%)	Schiz (%)
		Sevuparin/DF02+ Malanil	D3H47	NEGATIVE	0	ND	ND	ND	ND	ND	ND	ND	ND
		Sevuparin/DF02+ Malanil	D3H53	NEGATIVE	0	ND	ND	ND	ND	ND	ND	ND	ND
		Sevuparin/DF02+ Malanil	D7	Pf	0	ND	ND	ND	ND	ND	ND	ND	ND
		Sevuparin/DF02+ Malanil	D14	NEGATIVE	0	ND	ND	ND	ND	ND	ND	ND	ND
		Sevuparin/DF02+ Malanil	SCREENING	Pf	11475	0	0	2	54	35	9	0	0
		Sevuparin/DF02+ Malanil	D1H0	Pf	350	10	10	0	30	40	10	0	0
		Sevuparin/DF02+ Malanil	D1H1	Pf	375	ND	ND	ND	ND	ND	ND	ND	ND
		Sevuparin/DF02+ Malanil	D1H2	Pf	300	ND	ND	ND	ND	ND	ND	ND	ND
		Sevuparin/DF02+ Malanil	D1H3	Pf	300	ND	ND	ND	ND	ND	ND	ND	ND
		Sevuparin/DF02+ Malanil	D1H4	Pf	250	ND	ND	ND	ND	ND	ND	ND	ND
		Sevuparin/DF02+ Malanil	D1H6	Pf	225	ND	ND	ND	ND	ND	ND	ND	ND
		Sevuparin/DF02+ Malanil	D1H8	Pf	150	ND	ND	ND	ND	ND	ND	ND	ND
		Sevuparin/DF02+ Malanil	D1H10	Pf	75	ND	ND	ND	ND	ND	ND	ND	ND
		Sevuparin/DF02+	D1H11	Pf	50	ND	ND	ND	ND	ND	ND	ND	ND

SUBJID	INITIAL	ARM	TIMEPOINT	SPECIES	PARASITAEMIA (/µL)	Tiny Rings (%)	Small Rings (%)	Large Rings (%)	Early Troph (%)	Mid Troph (%)	Late Troph (%)	Gamet (%)	Schiz (%)
		Malanil											
		Sevuparin/DF02+ Malanil	D1H17	Pf	25	ND	ND	ND	ND	ND	ND	ND	ND
		Sevuparin/DF02+ Malanil	D1H23	NEGATIVE	0	ND	ND	ND	ND	ND	ND	ND	ND
		Sevuparin/DF02+ Malanil	D1H29	NEGATIVE	0	ND	ND	ND	ND	ND	ND	ND	ND
		Sevuparin/DF02+ Malanil	D7	NEGATIVE	0	ND	ND	ND	ND	ND	ND	ND	ND
		Sevuparin/DF02+ Malanil	D14	NEGATIVE	0	ND	ND	ND	ND	ND	ND	ND	ND
		Sevuparin/DF02+ Malanil	SCREENING	Pf	76860	66	26	7	1	0	0	0	0
		Sevuparin/DF02+ Malanil	D1H0	Pf	76860	71	27	2	0	0	0	0	0
		Sevuparin/DF02+ Malanil	D1H1	Pf	81130	51	34	10	5	0	0	0	0
		Sevuparin/DF02+ Malanil	D1H2	Pf	81130	48	38	10	4	0	0	0	0
		Sevuparin/DF02+ Malanil	D1H3	Pf	68320	24	54	17	5	0	0	0	0
		Sevuparin/DF02+ Malanil	D1H4	Pf	59780	29	41	21	9	0	0	0	0
		Sevuparin/DF02+ Malanil	D1H6	Pf	51240	37	37	10	15	1	0	0	0
		Sevuparin/DF02+ Malanil	D1H8	Pf	42700	43	34	8	15	0	0	0	0

SUBJID	INITIAL	ARM	TIMEPOINT	SPECIES	PARASITAEMIA (/µL)	Tiny Rings (%)	Small Rings (%)	Large Rings (%)	Early Troph (%)	Mid Troph (%)	Late Troph (%)	Gamet (%)	Schiz (%)
		Sevuparin/DF02+ Malanil	D1H10	Pf	28967	12	28	14	41	3	2	0	0
		Sevuparin/DF02+ Malanil	D1H11	Pf	22333	15	26	19	33	5	2	0	0
		Sevuparin/DF02+ Malanil	D1H17	Pf	16382	0	15	13	55	17	0	0	0
		Sevuparin/DF02+ Malanil	D1H23	Pf	3454	6	6	11	45	23	9	0	0
		Sevuparin/DF02+ Malanil	D1H29	Pf	276	ND	ND	ND	ND	ND	ND	ND	ND
		Sevuparin/DF02+ Malanil	D2H35	Pf	184	ND	ND	ND	ND	ND	ND	ND	ND
		Sevuparin/DF02+ Malanil	D2H41	Pf	61	ND	ND	ND	ND	ND	ND	ND	ND
		Sevuparin/DF02+ Malanil	D3H47	Pf	31	ND	ND	ND	ND	ND	ND	ND	ND
		Sevuparin/DF02+ Malanil	D3H53	Pf	0	ND	ND	ND	ND	ND	ND	ND	ND
		Sevuparin/DF02+ Malanil	D3H59	NEGATIVE	0	ND	ND	ND	ND	ND	ND	ND	ND
		Sevuparin/DF02+ Malanil	D7	NEGATIVE	0	ND	ND	ND	ND	ND	ND	ND	ND
		Sevuparin/DF02+ Malanil	D14	NEGATIVE	0	ND	ND	ND	ND	ND	ND	ND	ND
		Sevuparin/DF02+ Malanil	SCREENING	Pf	30236	7	29	38	26	0	0	0	0
		Sevuparin/DF02+	D1H0	Pf	20801	11	29	34	23	3	0	0	0

SUBJID	INITIAL	ARM	TIMEPOINT	SPECIES	PARASITAEMIA (/µL)	Tiny Rings (%)	Small Rings (%)	Large Rings (%)	Early Troph (%)	Mid Troph (%)	Late Troph (%)	Gamet (%)	Schiz (%)
		Malanil											
		Sevuparin/DF02+ Malanil	D1H1	Pf	16937	0	13	20	42	23	2	0	0
		Sevuparin/DF02+ Malanil	D1H2	Pf	16873	1	11	32	44	7	5	0	0
		Sevuparin/DF02+ Malanil	D1H3	Pf	14876	0	13	19	46	18	4	0	0
		Sevuparin/DF02+ Malanil	D1H4	Pf	13556	0	16	18	41	17	8	0	0
		Sevuparin/DF02+ Malanil	D1H6	Pf	10465	0	18	22	39	15	6	0	0
		Sevuparin/DF02+ Malanil	D1H8	Pf	9596	0	10	14	47	25	4	0	0
		Sevuparin/DF02+ Malanil	D1H10	Pf	4508	2	10	10	49	19	10	0	0
		Sevuparin/DF02+ Malanil	D1H11	Pf	3671	9	9	17	50	9	6	0	0
		Sevuparin/DF02+ Malanil	D1H17	Pf	1385	18	16	27	35	4	0	0	0
		Sevuparin/DF02+ Malanil	D1H23	Pf	708	9	4	18	41	23	5	0	0
		Sevuparin/DF02+ Malanil	D1H29	Pf	171	ND	ND	ND	ND	ND	ND	ND	ND
		Sevuparin/DF02+ Malanil	D2H35	Pf	95	ND	ND	ND	ND	ND	ND	ND	ND
		Sevuparin/DF02+ Malanil	D2H41	Pf	38	ND	ND	ND	ND	ND	ND	ND	ND

SUBJID	INITIAL	ARM	TIMEPOINT	SPECIES	PARASITAEMIA (/µL)	Tiny Rings (%)	Small Rings (%)	Large Rings (%)	Early Troph (%)	Mid Troph (%)	Late Troph (%)	Gamet (%)	Schiz (%)
		Sevuparin/DF02+ Malanil	D3H47	NEGATIVE	0	ND	ND	ND	ND	ND	ND	ND	ND
		Sevuparin/DF02+ Malanil	D3H53	NEGATIVE	0	ND	ND	ND	ND	ND	ND	ND	ND
		Sevuparin/DF02+ Malanil	D7	NEGATIVE	0	ND	ND	ND	ND	ND	ND	ND	ND
		Sevuparin/DF02+ Malanil	D14	NEGATIVE	0	ND	ND	ND	ND	ND	ND	ND	ND
		Sevuparin/DF02+ Malanil	SCREENING	Pf	24613	44	44	9	3	0	0	0	0
		Sevuparin/DF02+ Malanil	D1H0	Pf	20288	33	39	20	8	0	0	0	0
		Sevuparin/DF02+ Malanil	D1H1	Pf	19336	26	38	28	8	0	0	0	0
		Sevuparin/DF02+ Malanil	D1H2	Pf	16974	24	31	21	23	0	1	0	0
		Sevuparin/DF02+ Malanil	D1H3	Pf	16707	14	28	24	32	2	0	0	0
		Sevuparin/DF02+ Malanil	D1H4	Pf	16231	8	22	29	40	1	0	0	0
		Sevuparin/DF02+ Malanil	D1H6	Pf	14249	2	11	18	52	16	1	0	0
		Sevuparin/DF02+ Malanil	D1H8	Pf	14192	1	9	13	42	26	9	0	0
		Sevuparin/DF02+ Malanil	D1H10	Pf	12268	5	27	18	37	8	5	0	0
		Sevuparin/DF02+	D1H11	Pf	11773	0	8	8	34	31	19	0	0

SUBJID	INITIAL	ARM	TIMEPOINT	SPECIES	PARASITAEMIA (/µL)	Tiny Rings (%)	Small Rings (%)	Large Rings (%)	Early Troph (%)	Mid Troph (%)	Late Troph (%)	Gamet (%)	Schiz (%)
		Malanil											
		Sevuparin/DF02+ Malanil	D1H17	Pf	11087	4	2	6	51	21	16	0	0
		Sevuparin/DF02+ Malanil	D1H23	Pf	5639	0	4	10	43	23	20	0	0
		Sevuparin/DF02+ Malanil	D1H29	Pf	527	0	10	10	40	20	20	0	0
		Sevuparin/DF02+ Malanil	D2H35	Pf	229	ND	ND	ND	ND	ND	ND	ND	ND
		Sevuparin/DF02+ Malanil	D2H41	Pf	69	ND	ND	ND	ND	ND	ND	ND	ND
		Sevuparin/DF02+ Malanil	D3H47	NEGATIVE	0	ND	ND	ND	ND	ND	ND	ND	ND
		Sevuparin/DF02+ Malanil	D3H53	NEGATIVE	0	ND	ND	ND	ND	ND	ND	ND	ND
		Sevuparin/DF02+ Malanil	D7	NEGATIVE	0	ND	ND	ND	ND	ND	ND	ND	ND
		Sevuparin/DF02+ Malanil	D14	NEGATIVE	0	ND	ND	ND	ND	ND	ND	ND	ND
		Malanil	SCREENING	Pf	96300	77	22	1	0	0	0	0	0
		Malanil	D1H0	Pf	107000	64	31	5	0	0	0	0	0
		Malanil	D1H1	Pf	101650	70	29	1	0	0	0	0	0
		Malanil	D1H2	Pf	96300	56	37	7	0	0	0	0	0
		Malanil	D1H3	Pf	96300	38	38	16	8	0	0	0	0
		Malanil	D1H4	Pf	90950	49	34	9	7	1	0	0	0

SUBJID	INITIAL	ARM	TIMEPOINT	SPECIES	PARASITAEMIA (/µL)	Tiny Rings (%)	Small Rings (%)	Large Rings (%)	Early Troph (%)	Mid Troph (%)	Late Troph (%)	Gamet (%)	Schiz (%)
		Malanil	D1H6	Pf	96300	61	27	7	5	0	0	0	0
		Malanil	D1H8	Pf	90950	45	30	13	9	3	0	0	0
		Malanil	D1H10	Pf	85600	43	28	14	15	0	0	0	0
		Malanil	D1H11	Pf	80250	37	32	7	19	4	1	0	0
		Malanil	D1H17	Pf	74900	15	24	10	32	11	8	0	0
		Malanil	D1H23	Pf	69240	9	10	8	35	17	21	0	0
		Malanil	D1H29	Pf	20467	ND	ND	ND	ND	ND	ND	ND	ND
		Malanil	D2H35	Pf	6068	ND	ND	ND	ND	ND	ND	ND	ND
		Malanil	D2H41	Pf	2371	ND	ND	ND	ND	ND	ND	ND	ND
		Malanil	D3H47	Pf	1037	ND	ND	ND	ND	ND	ND	ND	ND
		Malanil	D3H53	Pf	718	ND	ND	ND	ND	ND	ND	ND	ND
		Malanil	D3H59	Pf	399	ND	ND	ND	ND	ND	ND	ND	ND
		Malanil	D3H65	Pf	106	ND	ND	ND	ND	ND	ND	ND	ND
		Malanil	D3H71	NEGATIVE	0	ND	ND	ND	ND	ND	ND	ND	ND
		Malanil	D4	NEGATIVE	0	ND	ND	ND	ND	ND	ND	ND	ND
		Malanil	D7	NEGATIVE	0	ND	ND	ND	ND	ND	ND	ND	ND
		Malanil	D14	NEGATIVE	0	ND	ND	ND	ND	ND	ND	ND	ND
		Malanil	SCREENING	Pf	49824	71	21	7	1	0	0	0	0
		Malanil	D1H0	Pf	104310	28	39	15	14	4	0	0	0
		Malanil	D1H1	Pf	109800	23	33	20	22	2	0	0	0
		Malanil	D1H2	Pf	109800	22	41	13	24	0	0	0	0
		Malanil	D1H3	Pf	115290	12	35	29	23	1	0	0	0

SUBJID	INITIAL	ARM	TIMEPOINT	SPECIES	PARASITAEMIA (/µL)	Tiny Rings (%)	Small Rings (%)	Large Rings (%)	Early Troph (%)	Mid Troph (%)	Late Troph (%)	Gamet (%)	Schiz (%)
		Malanil	D1H4	Pf	115290	6	32	31	30	1	0	0	0
		Malanil	D1H6	Pf	109800	14	30	28	28	0	0	0	0
		Malanil	D1H8	Pf	104310	17	25	22	36	0	0	0	0
		Malanil	D1H10	Pf	98820	14	26	21	34	3	2	0	0
		Malanil	D1H11	Pf	93330	0	19	27	51	3	0	0	0
		Malanil	D1H17	Pf	82350	1	7	11	63	15	3	0	0
		Malanil	D1H23	Pf	69020	0	12	5	50	18	15	0	0
		Malanil	D1H29	Pf	41272	ND	ND	ND	ND	ND	ND	ND	ND
		Malanil	D2H35	Pf	10134	ND	ND	ND	ND	ND	ND	ND	ND
		Malanil	D2H41	Pf	2432	ND	ND	ND	ND	ND	ND	ND	ND
		Malanil	D3H47	Pf	415	ND	ND	ND	ND	ND	ND	ND	ND
		Malanil	D3H53	Pf	104	ND	ND	ND	ND	ND	ND	ND	ND
		Malanil	D3H59	NEGATIVE	0	ND	ND	ND	ND	ND	ND	ND	ND
		Malanil	D3H65	NEGATIVE	0	ND	ND	ND	ND	ND	ND	ND	ND
		Malanil	D7	Pf	0	ND	ND	ND	ND	ND	ND	ND	ND
		Malanil	D14	Pf	0	ND	ND	ND	ND	ND	ND	ND	ND
		Malanil	SCREENING	Pf	99590	62	29	7	2	0	0	0	0
		Malanil	D1H0	Pf	121240	50	40	9	1	0	0	0	0
		Malanil	D1H1	Pf	129900	58	39	3	0	0	0	0	0
		Malanil	D1H2	Pf	138560	46	46	7	1	0	0	0	0
		Malanil	D1H3	Pf	151550	50	33	15	2	0	0	0	0
		Malanil	D1H4	Pf	155880	46	40	12	2	0	0	0	0

SUBJID	INITIAL	ARM	TIMEPOINT	SPECIES	PARASITAEMIA (/µL)	Tiny Rings (%)	Small Rings (%)	Large Rings (%)	Early Troph (%)	Mid Troph (%)	Late Troph (%)	Gamet (%)	Schiz (%)
		Malanil	D1H6	Pf	151550	33	44	19	4	0	0	0	0
		Malanil	D1H8	Pf	147220	36	42	17	5	0	0	0	0
		Malanil	D1H10	Pf	138560	18	39	24	14	4	1	0	0
		Malanil	D1H11	Pf	129900	17	43	22	14	4	0	0	0
		Malanil	D1H17	Pf	121240	5	34	24	24	8	5	0	0
		Malanil	D1H23	Pf	99320	0	18	11	48	13	10	0	0
		Malanil	D1H29	Pf	61120	1	16	2	28	39	14	0	0
		Malanil	D2H35	Pf	14365	ND	ND	ND	ND	ND	ND	ND	ND
		Malanil	D2H41	Pf	2515	ND	ND	ND	ND	ND	ND	ND	ND
		Malanil	D3H47	Pf	1058	ND	ND	ND	ND	ND	ND	ND	ND
		Malanil	D3H53	Pf	554	ND	ND	ND	ND	ND	ND	ND	ND
		Malanil	D3H59	Pf	126	ND	ND	ND	ND	ND	ND	ND	ND
		Malanil	D3H65	Pf	50	ND	ND	ND	ND	ND	ND	ND	ND
		Malanil	D3H71	Pf	0	ND	ND	ND	ND	ND	ND	ND	ND
		Malanil	D4	Pf	0	ND	ND	ND	ND	ND	ND	ND	ND
		Malanil	D7	Pf	0	ND	ND	ND	ND	ND	ND	ND	ND
		Malanil	D14	Pf	0	ND	ND	ND	ND	ND	ND	ND	ND

16.2.6.2 Cytoadherance, rosette assay

To be reported later.

16.2.7 Individual safety data

16.2.7.1 Adverse event listings

SUBJ ID	INITIAL	ARM	AE SEQ	AETERM	AEDECOD	AESOC	DESCRIPTION	ONSET DATE TIME *99:99=UNK*	END DATE	OUTCOME	INTENS	RELAT. DF02	RELAT. MALANIL	ALT RELAT	ALT REL OTHER	ACTION
		Sevuparin/ DF02+ Malanil	1	Eosinophilia	Eosinophilia	Blood and lymphatic system disorders	RESULT EO = 25% DATE 25-AUG-2012 EO = 10% DATE 10-SEP-2012			Improving	Mild	Not related	Not related	Other	PARASITE	Treatment Medicatio
		Sevuparin/ DF02+ Malanil	2	PARASITE STOOL TEST POSITIVE	Parasite stool test positive	Investigations	HIGH LEVEL OF CREATININE KINASE BUT NON CLINICALLY SIGNIFICANT; RESULT STOOL EXAM FOR PARASITE = HOOKWORM EGG DATE 26AUG2012; RESULT STOOL EXAM FOR PARASITE = NONE SEEN DATE 29AUG2012			Recovered	Mild	Not related	Not related	Other	PARASITE	Treatment Medicatio
		Malanil	1	ERYTHEMATOUS RASH	Rash erythematous	Skin and subcutaneous tissue	ERYTHEMA RASH AT ABDOMINAL			Recovered	Mild	Not applicable	Possibly related	Other	UNKNOWN	Treatment Medicatio

SUBJ ID	INITIAL	ARM	AE SEQ	AETERM	AEDECOD	AESOC	DESCRIPTION	ONSET DATE TIME *99:99=UNK*	END DATE	OUTCOME	INTENS	RELAT. DF02	RELAT. MALANIL	ALT RELAT	ALT REL OTHER	ACTION
						disorders	SURFACE									
		Malanil	1	FEVER	Pyrexia	General disorders and administration site conditions	SUBJECT TOLD THAT HAD FEVER FOR TWO DAYS BUT AS PHYSICAL EXAM ON THIS VISIT NO FEVER FOUND.			Recovered	Mild	Not applicable	Not related	Other	UNKNOWN	Treatment Medicatio
		Sevuparin/ DF02+Malanil	1	AST increased	Aspartate aminotransferase increased	Investigations	RESULTS AST INCREASED DAY 8 = 176 U/L			Recovered	Moderate	Possibly related	Not related	Concurrent illness	MALARIA	None
		Sevuparin/ DF02+Malanil	2	ALT INCREASED	Alanine aminotransferase increased	Investigations	RESULTS AST AND ALT INCREASED			Recovered	Moderate	Possibly related	Not related	Concurrent illness	MALARIA	None
		Sevuparin/ DF02+Malanil	1	PREMATURE VENTRICULAR COMPLEXES	Ventricular extrasystoles	Cardiac disorders	ECG SINUS TACHYCARDIA / PREMATURE VENTRICULAR COMPLEXES			Recovered	Mild	Not related	Not related	Concurrent illness	MALARIA	None
		Sevuparin/ DF02+Malanil	2	ANOREXIA	Decreased appetite	Metabolism and nutrition disorders	ANOREXIA			Recovered	Mild	Not related	Not related	Concurrent illness	MALARIA	Treatment Medicatio
		Sevuparin/ DF02+Malanil	3	PHARYNGITIS	Pharyngitis	Infections and infestations	FEVER, COUGH, SORE THROAT,			Recovered	Moderate	Not related	Not related	Other	VIRUS	Treatment Medicatio

SUBJ ID	INITIAL	ARM	AE SEQ	AETERM	AEDECOD	AESOC	DESCRIPTION	ONSET DATE TIME *99:99=UNK*	END DATE	OUTCOME	INTENS	RELAT. DF02	RELAT. MALANIL	ALT RELAT	ALT REL OTHER	ACTION
							MUSCLE PAIN									
		Sevuparin/ DF02+Malanil	4	SINUS TACHYCARDIA	Sinus tachycardia	Cardiac disorders	ECG SINUS TACHYCARDIA			Recovered	Mild	Not related	Not related	Concurrent illness	MALARIA	None
		Malanil	1	Platelets decrease	Platelet count decreased	Investigations	PLATELETS DAY SCREENING RESULT 109,000 THEN PLATELETS (DAY2) TIME 48 RESULT 66,000			Recovered	Mild	Not applicable	Not related	Concurrent illness	MALARIA	None
		Malanil	2	NAUSEA	Nausea	Gastrointestinal disorders	NAUSEA			Recovered	Mild	Not applicable	Possibly related	Concurrent illness	MALARIA	Treatment Medicatic
		Malanil	3	ANOREXIA	Decreased appetite	Metabolism and nutrition disorders	ANOREXIA			Recovered	Mild	Not applicable	Not related	Concurrent illness	MALARIA	None
		Malanil	1	HYPOKALEMIA	Hypokalaemia	Metabolism and nutrition disorders	RESULT POTASSIUM =3.3 MMOL/L (DAY 1)			Recovered	Mild	Not applicable	Not related	Concurrent illness	MALARIA	Treatment Medicatic
		Sevuparin/ DF02+Malanil	1	URINARY TRACT INFECTION	Urinary tract infection	Infections and infestations	RESULT URINE WBC OVER 100 DATE 5 FEB 2013			Recovered	Moderate	Not related	Not related	Other	UNKNOWN	Treatment Medicatic
		Sevuparin/ DF02+Mala	1	RESULT AST INCREASE	Aspartate aminotransfer	Investigations	RESULT AST = 173 U/L			Improving	Moderate	Possibly related	Not related	Other	UNKNOWN	None

SUBJ ID	INITIAL	ARM	AE SEQ	AETERM	AEDECOD	AESOC	DESCRIPTION	ONSET DATE TIME *99:99=UNK*	END DATE	OUTCOME	INTENS	RELAT. DF02	RELAT. MALANIL	ALT RELAT	ALT REL OTHER	ACTION
		nil			ase increased											
		Sevuparin/ DF02+Malanil	2	RESULT ALT INCREASE	Alanine aminotransferase increased	Investigations	RESULT ALT= 200 U/L			Improving	Moderate	Possibly related	Not related	Other	UNKNOWN	None
		Malanil	1	Platelets decrease	Platelet count decreased	Investigations	RESULT PLATELETS DATE 12-FEB-2013 = 91 THEN DATE 13-FEB-2013 = 65 AND DATE 14-FEB-2013 = 64 REPEAT DATE 15-FEB-2013 RESULT = 93 10(3)/UL			Recovered	Moderate	Not applicable	Not related	Concurrent illness	MALARIA	None
		Sevuparin/ DF02+Malanil	1	LONG QTC INTERVAL	Electrocardiogram QT prolonged	Investigations	QTC INTERVAL 456 MS			Recovered	Mild	Not related	Possibly related	Other	UNKNOWN	None
		Malanil	1	PVC	Ventricular extrasystoles	Cardiac disorders	PREMATURE VENTRICULAR CONTRACTION			Recovered	Mild	Not applicable	Not related	Other	UNKNOWN	None
		Sevuparin/ DF02+Malanil	1	PLATELETS DECREASED	Platelet count decreased	Investigations	RESULT PLATELETS DAY 1 = 28,000 /UL, DAY 2 = 29,000 /UL			Recovered	Severe	Possibly related	Possibly related	Concurrent illness	MALARIA	None
		Sevuparin/ DF02+Mala	2	ALKALINE PHOSPHATASE	Blood alkaline phosphatase	Investigations	RESULT ALP DAY 7 = 138 U/L, DAY 14 =			Not recovered	Mild	Possibly related	Possibly related	Concurrent illness	MALARIA	None

SUBJ ID	INITIAL	ARM	AE SEQ	AETERM	AEDECOD	AESOC	DESCRIPTION	ONSET DATE TIME *99:99=UNK*	END DATE	OUTCOME	INTENS	RELAT. DF02	RELAT. MALANIL	ALT RELAT	ALT REL OTHER	ACTION
		nil		INCREASED	increased		208 U/L									
		Sevuparin/ DF02+Malanil	3	ALANINE AMINOTRANSFERASE INCREASED	Alanine aminotransferase increased	Investigations	RESULT ALT DAY 7 = 391 U/L, DAY 14 = 380 U/L			Not recovered	Severe	Possibly related	Possibly related	Concurrent illness	MALARIA	None
		Sevuparin/ DF02+Malanil	4	ASPARTATE AMINOTRANSFERASE INCREASED	Aspartate aminotransferase increased	Investigations	RESULT AST DAY 7 = 451 U/L			Improving	Severe	Possibly related	Possibly related	Concurrent illness	MALARIA	None
		Sevuparin/ DF02+Malanil	5	BLOOD BILIRUBIN INCREASED	Blood bilirubin increased	Investigations	RESULT TOTAL BILIRUBIN =1.99 MG/DL			Not recovered	Moderate	Possibly related	Possibly related	Concurrent illness	MALARIA	None
		Malanil	1	ELECTROCARDIOGRAM QTc INTERVAL PROLONGED	Electrocardiogram QT prolonged	Investigations	QTC INTERVAL 11-APR-2013 = 475 MS, 14-APR-2013 = 538 MS, 17-APR-2013 = 444 MS			Recovered	Mild	Not applicable	Not related	Other	UNKNOWN	None
		Malanil	2	PLATELETS DECREASED	Platelet count decreased	Investigations	RESULT PLATELETS 12-APR-2013 = 87,000 /UL, 17-APR-2013 = 273,000 /UL			Recovered	Mild	Not applicable	Not related	Concurrent illness	MALARIA	None
		Malanil	1	Aspartate Aminotransferase Increased	Aspartate aminotransferase increased	Investigations	RESULT AST DAY7 = 143 U/L			Not recovered	Moderate	Not applicable	Not related	Other	PATIENT HAVE HISTORY DRINK ALCOHOL	None

SUBJ ID	INITIAL	ARM	AE SEQ	AETERM	AEDECOD	AESOC	DESCRIPTION	ONSET DATE TIME *99:99=UNK*	END DATE	OUTCOME	INTENS	RELAT. DF02	RELAT. MALANIL	ALT RELAT	ALT REL OTHER	ACTION
															EVERY DAY.	
		Malanil	1	BRADYCARDIA	Bradycardia	Cardiac disorders	BRADYCARDIA HEART RATE=47 BPM			Recovered	Mild	Not applicable	Not related	Other	UNKNOWN	None

16.2.7.2 Serious adverse event listing

Not applicable.

16.2.7.3 Physical examination (including weight and height)

SUBJID	INITIAL	ARM	DAY	HGT (cm)	WGT (kg)	GCSEYE	GCSVERBAL	GCSMOTOR	GCSTOTAL
		Malanil	0	157	50	4	5	6	15
		Malanil	4	NA	50	NA	NA	NA	NA
		Sevuparin/DF02+Malanil	0	163	55	4	5	6	15
		Sevuparin/DF02+Malanil	4	NA	55.7	NA	NA	NA	NA
		Sevuparin/DF02+Malanil	7	NA	54.5	4	5	6	15
		Sevuparin/DF02+Malanil	14	NA	54.1	4	5	6	15
		Sevuparin/DF02+Malanil	0	160	48	4	5	6	15
		Sevuparin/DF02+Malanil	4	NA	47.7	NA	NA	NA	NA
		Sevuparin/DF02+Malanil	7	NA	48.1	4	5	6	15
		Sevuparin/DF02+Malanil	14	NA	48.6	4	5	6	15
		Malanil	0	173	62	4	5	6	15
		Malanil	4	NA	62.5	NA	NA	NA	NA
		Malanil	7	NA	61.7	4	5	6	15
		Malanil	14	NA	62.9	4	5	6	15
		Malanil	0	160	55	4	5	6	15
		Malanil	4	NA	56.5	NA	NA	NA	NA
		Malanil	7	NA	55.8	4	5	6	15
		Malanil	14	NA	55.6	4	5	6	15
		Sevuparin/DF02+Malanil	0	168	64.2	4	5	6	15
		Sevuparin/DF02+Malanil	4	NA	65.5	NA	NA	NA	NA
		Sevuparin/DF02+Malanil	7	NA	63.4	4	5	6	15

SUBJID	INITIAL	ARM	DAY	HGT (cm)	WGT (kg)	GCSEYE	GCSVERBAL	GCSMOTOR	GCSTOTAL
		Sevuparin/DF02+Malanil	14	NA	66.1	4	5	6	15
		Malanil	0	160	56.5	4	5	6	15
		Malanil	4	NA	56.6	NA	NA	NA	NA
		Malanil	7	NA	55.6	4	5	6	15
		Sevuparin/DF02+Malanil	0	167	57.6	4	5	6	15
		Sevuparin/DF02+Malanil	4	NA	57.8	NA	NA	NA	NA
		Sevuparin/DF02+Malanil	7	NA	56.5	4	5	6	15
		Sevuparin/DF02+Malanil	14	NA	55.7	4	5	6	15
		Sevuparin/DF02+Malanil	0	163	49.3	4	5	6	15
		Sevuparin/DF02+Malanil	4	NA	49	NA	NA	NA	NA
		Sevuparin/DF02+Malanil	7	NA	50.2	4	5	6	15
		Sevuparin/DF02+Malanil	14	NA	51.3	4	5	6	15
		Malanil	0	165	51	4	5	6	15
		Malanil	4	NA	50	NA	NA	NA	NA
		Malanil	7	NA	53.1	4	5	6	15
		Malanil	14	NA	52	4	5	6	15
		Malanil	0	158	50.2	4	5	6	15
		Malanil	4	NA	48	NA	NA	NA	NA
		Malanil	7	NA	48.4	4	5	6	15
		Malanil	14	NA	48.1	4	5	6	15
		Sevuparin/DF02+Malanil	0	161	54.7	4	5	6	15
		Sevuparin/DF02+Malanil	4	NA	55.1	NA	NA	NA	NA
		Sevuparin/DF02+Malanil	7	NA	54.9	4	5	6	15

SUBJID	INITIAL	ARM	DAY	HGT (cm)	WGT (kg)	GCSEYE	GCSVERBAL	GCSMOTOR	GCSTOTAL
		Sevuparin/DF02+Malanil	0	157	54.2	4	5	6	15
		Sevuparin/DF02+Malanil	4	NA	54.2	NA	NA	NA	NA
		Sevuparin/DF02+Malanil	7	NA	53	4	5	6	15
		Sevuparin/DF02+Malanil	14	NA	52.8	4	5	6	15
		Malanil	0	161	55.4	4	5	6	15
		Malanil	4	NA	53.3	NA	NA	NA	NA
		Malanil	7	NA	53.8	4	5	6	15
		Malanil	14	NA	54.2	4	5	6	15
		Malanil	0	171	55.9	4	5	6	15
		Malanil	4	NA	56.3	NA	NA	NA	NA
		Malanil	7	NA	57.1	4	5	6	15
		Malanil	14	NA	56.8	4	5	6	15
		Malanil	0	150	49.4	4	5	6	15
		Malanil	4	NA	48.5	NA	NA	NA	NA
		Malanil	7	NA	48.7	4	5	6	15
		Malanil	14	NA	48.8	4	5	6	15
		Sevuparin/DF02+Malanil	0	159	56.7	4	5	6	15
		Sevuparin/DF02+Malanil	4	NA	56.6	NA	NA	NA	NA
		Sevuparin/DF02+Malanil	7	NA	54.9	4	5	6	15
		Sevuparin/DF02+Malanil	14	NA	54	4	5	6	15
		Sevuparin/DF02+Malanil	0	164	67.3	4	5	6	15
		Sevuparin/DF02+Malanil	4	NA	66.9	NA	NA	NA	NA
		Sevuparin/DF02+Malanil	7	NA	68.6	4	5	6	15

SUBJID	INITIAL	ARM	DAY	HGT (cm)	WGT (kg)	GCSEYE	GCSVERBAL	GCSMOTOR	GCSTOTAL
		Sevuparin/DF02+Malanil	14	NA	68.5	4	5	6	15
		Sevuparin/DF02+Malanil	0	155	50.1	4	5	6	15
		Sevuparin/DF02+Malanil	4	NA	49.7	NA	NA	NA	NA
		Sevuparin/DF02+Malanil	7	NA	49.7	4	5	6	15
		Sevuparin/DF02+Malanil	14	NA	48.6	4	5	6	15
		Malanil	0	168	54.4	4	5	6	15
		Malanil	4	NA	55.5	NA	NA	NA	NA
		Malanil	7	NA	57	4	5	6	15
		Malanil	14	NA	58	4	5	6	15
		Sevuparin/DF02+Malanil	0	165	56.1	4	5	6	15
		Sevuparin/DF02+Malanil	4	NA	54.1	NA	NA	NA	NA
		Sevuparin/DF02+Malanil	7	NA	54	4	5	6	15
		Sevuparin/DF02+Malanil	14	NA	56	4	5	6	15
		Sevuparin/DF02+Malanil	0	163	49	4	5	6	15
		Sevuparin/DF02+Malanil	4	NA	50.8	NA	NA	NA	NA
		Sevuparin/DF02+Malanil	7	NA	50.6	4	5	6	15
		Sevuparin/DF02+Malanil	14	NA	48	4	5	6	15
		Sevuparin/DF02+Malanil	0	152	66.9	4	5	6	15
		Sevuparin/DF02+Malanil	4	NA	66.1	NA	NA	NA	NA
		Sevuparin/DF02+Malanil	7	NA	66.7	4	5	6	15
		Sevuparin/DF02+Malanil	14	NA	66.4	4	5	6	15
		Sevuparin/DF02+Malanil	0	154	44.7	4	5	6	15
		Sevuparin/DF02+Malanil	4	NA	42.6	NA	NA	NA	NA

SUBJID	INITIAL	ARM	DAY	HGT (cm)	WGT (kg)	GCSEYE	GCSVERBAL	GCSMOTOR	GCSTOTAL
		Sevuparin/DF02+Malanil	7	NA	44.4	4	5	6	15
		Sevuparin/DF02+Malanil	14	NA	43.8	4	5	6	15
		Malanil	0	142	48.1	4	5	6	15
		Malanil	4	NA	47.5	NA	NA	NA	NA
		Malanil	7	NA	45.3	4	5	6	15
		Malanil	14	NA	47.9	4	5	6	15
		Malanil	0	161	59.7	4	5	6	15
		Malanil	4	NA	58.2	NA	NA	NA	NA
		Malanil	7	NA	59.5	4	5	6	15
		Malanil	14	NA	60.6	4	5	6	15
		Malanil	0	157	54.5	4	5	6	15
		Malanil	4	NA	55.8	NA	NA	NA	NA
		Malanil	7	NA	53.9	4	5	6	15
		Malanil	14	NA	52.3	4	5	6	15
		Sevuparin/DF02+Malanil	0	163	54.8	4	5	6	15
		Sevuparin/DF02+Malanil	4	NA	53.8	NA	NA	NA	NA
		Sevuparin/DF02+Malanil	7	NA	54.7	4	5	6	15
		Sevuparin/DF02+Malanil	14	NA	50.6	4	5	6	15
		Malanil	0	169	54.4	4	5	6	15
		Malanil	4	NA	52.3	NA	NA	NA	NA
		Malanil	7	NA	52.3	4	5	6	15
		Malanil	14	NA	52.6	4	5	6	15
		Malanil	0	155	51.7	4	5	6	15

SUBJID	INITIAL	ARM	DAY	HGT (cm)	WGT (kg)	GCSEYE	GCSVERBAL	GCSMOTOR	GCSTOTAL
		Malanil	4	NA	51	NA	NA	NA	NA
		Malanil	7	NA	52.2	4	5	6	15
		Malanil	14	NA	53.1	4	5	6	15
		Malanil	0	156	53.5	4	5	6	15
		Malanil	4	NA	53.3	NA	NA	NA	NA
		Malanil	7	NA	53.3	4	5	6	15
		Malanil	14	NA	54.9	4	5	6	15
		Malanil	0	161	48.3	4	5	6	15
		Malanil	4	NA	48.5	NA	NA	NA	NA
		Malanil	7	NA	49.1	4	5	6	15
		Malanil	14	NA	49.9	4	5	6	15
		Sevuparin/DF02+Malanil	0	159	44.8	4	5	6	15
		Sevuparin/DF02+Malanil	4	NA	44.1	NA	NA	NA	NA
		Sevuparin/DF02+Malanil	7	NA	43.6	4	5	6	15
		Sevuparin/DF02+Malanil	14	NA	45.4	4	5	6	15
		Malanil	0	157	51.8	4	5	6	15
		Malanil	4	NA	50.9	NA	NA	NA	NA
		Malanil	7	NA	51.5	4	5	6	15
		Malanil	14	NA	51.4	4	5	6	15
		Malanil	0	161	47	4	5	6	15
		Malanil	4	NA	46.3	NA	NA	NA	NA
		Malanil	7	NA	44.3	4	5	6	15
		Malanil	14	NA	45.9	4	5	6	15

SUBJID	INITIAL	ARM	DAY	HGT (cm)	WGT (kg)	GCSEYE	GCSVERBAL	GCSMOTOR	GCSTOTAL
		Malanil	0	161	53.5	4	5	6	15
		Malanil	4	NA	53.4	NA	NA	NA	NA
		Malanil	7	NA	54.5	4	5	6	15
		Malanil	14	NA	52.6	4	5	6	15
		Sevuparin/DF02+Malanil	0	164	57.2	4	5	6	15
		Sevuparin/DF02+Malanil	4	NA	56.9	NA	NA	NA	NA
		Sevuparin/DF02+Malanil	7	NA	57.4	4	5	6	15
		Sevuparin/DF02+Malanil	14	NA	57.4	4	5	6	15
		Sevuparin/DF02+Malanil	0	159	51.3	4	5	6	15
		Sevuparin/DF02+Malanil	4	NA	51.9	NA	NA	NA	NA
		Sevuparin/DF02+Malanil	7	NA	50.8	4	5	6	15
		Sevuparin/DF02+Malanil	14	NA	52	4	5	6	15
		Sevuparin/DF02+Malanil	0	162	55.6	4	5	6	15
		Sevuparin/DF02+Malanil	4	NA	53.8	NA	NA	NA	NA
		Sevuparin/DF02+Malanil	7	NA	52.1	4	5	6	15
		Sevuparin/DF02+Malanil	14	NA	55.8	4	5	6	15
		Sevuparin/DF02+Malanil	0	161	44.3	4	5	6	15
		Sevuparin/DF02+Malanil	4	NA	46.3	NA	NA	NA	NA
		Sevuparin/DF02+Malanil	7	NA	47	4	5	6	15
		Sevuparin/DF02+Malanil	14	NA	47	4	5	6	15
		Sevuparin/DF02+Malanil	0	159	43.6	4	5	6	15
		Sevuparin/DF02+Malanil	4	NA	43.7	NA	NA	NA	NA
		Sevuparin/DF02+Malanil	7	NA	44.3	4	5	6	15

SUBJID	INITIAL	ARM	DAY	HGT (cm)	WGT (kg)	GCSEYE	GCSVERBAL	GCSMOTOR	GCSTOTAL
		Sevuparin/DF02+Malanil	14	NA	44.5	4	5	6	15
		Malanil	0	159	45.8	4	5	6	15
		Malanil	4	NA	44.7	NA	NA	NA	NA
		Malanil	7	NA	45.8	4	5	6	15
		Malanil	14	NA	46.9	4	5	6	15
		Malanil	0	165	54.5	4	5	6	15
		Malanil	4	NA	53.9	NA	NA	NA	NA
		Malanil	7	NA	55.5	4	5	6	15
		Malanil	14	NA	56.6	4	5	6	15
		Malanil	0	156	50	4	5	6	15
		Malanil	4	NA	51.2	NA	NA	NA	NA
		Malanil	5	NA	51.2	NA	NA	NA	NA
		Malanil	7	NA	50.7	4	5	6	15
		Malanil	14	NA	50.3	4	5	6	15

16.2.7.3 Physical examination (including weight and height) (-continued)

SUBJID	INITIAL	ARM	DAY	SKIN	HEAD	EYES	EYES DETAIL	EARS	NOSE	THROAT	NECK	NECK DETAIL
		Malanil	0	Normal	Normal	Normal		Normal	Normal	Normal	Normal	
		Malanil	2	Normal	Normal	Normal		Normal	Normal	Normal	Normal	
		Malanil	3	Normal	Normal	Normal		Normal	Normal	Normal	Normal	
		Malanil	4	Normal	Normal	Normal		Normal	Normal	Normal	Normal	
		Sevuparin/DF02+Malanil	0	Normal	Normal	Normal		Normal	Normal	Normal	Normal	
		Sevuparin/DF02+Malanil	2	Normal	Normal	Normal		Normal	Normal	Normal	Normal	
		Sevuparin/DF02+Malanil	3	Normal	Normal	Normal		Normal	Normal	Normal	Normal	
		Sevuparin/DF02+Malanil	4	Normal	Normal	Normal		Normal	Normal	Normal	Normal	
		Sevuparin/DF02+Malanil	7	Normal	Normal	Normal		Normal	Normal	Normal	Normal	
		Sevuparin/DF02+Malanil	14	Normal	Normal	Normal		Normal	Normal	Normal	Normal	
		Sevuparin/DF02+Malanil	0	Normal	Normal	Normal		Normal	Normal	Normal	Normal	
		Sevuparin/DF02+Malanil	2	Normal	Normal	Normal		Normal	Normal	Normal	Normal	
		Sevuparin/DF02+Malanil	3	Normal	Normal	Normal		Normal	Normal	Normal	Normal	
		Sevuparin/DF02+Malanil	4	Normal	Normal	Normal		Normal	Normal	Normal	Normal	
		Sevuparin/DF02+Malanil	7	Normal	Normal	Normal		Normal	Normal	Normal	Normal	
		Sevuparin/DF02+Malanil	14	Normal	Normal	Normal		Normal	Normal	Normal	Normal	
		Malanil	0	Normal	Normal	Normal		Normal	Normal	Normal	Normal	
		Malanil	2	Normal	Normal	Normal		Normal	Normal	Normal	Normal	
		Malanil	3	Normal	Normal	Normal		Normal	Normal	Normal	Normal	
		Malanil	4	Normal	Normal	Normal		Normal	Normal	Normal	Normal	
		Malanil	7	Normal	Normal	Normal		Normal	Normal	Normal	Normal	
		Malanil	14	Normal	Normal	Normal		Normal	Normal	Normal	Normal	

SUBJID	INITIAL	ARM	DAY	SKIN	HEAD	EYES	EYES DETAIL	EARS	NOSE	THROAT	NECK	NECK DETAIL
		Malanil	0	Normal	Normal	Normal		Normal	Normal	Normal	Abnormal	CYSTIC MASS AT ADAM APPLE AREA DIAMETER 3CM-NSC
		Malanil	2	Normal	Normal	Normal		Normal	Normal	Normal	Abnormal	CYSTIC MASS AT ADAM APPLE AREA DIAMETER 3CM-NSC
		Malanil	3	Normal	Normal	Normal		Normal	Normal	Normal	Abnormal	CYSTIC MASS AT ADAM APPLE AREA DIAMETER 3CM-NSC
		Malanil	4	Normal	Normal	Normal		Normal	Normal	Normal	Abnormal	CYSTIC MASS AT ADAM APPLE AREA DIAMETER 3CM
		Malanil	7	Normal	Normal	Normal		Normal	Normal	Normal	Abnormal	CYSTIC MASS AT ADAM APPLE AREA DIAMETER 3CM
		Malanil	14	Normal	Normal	Normal		Normal	Normal	Normal	Abnormal	CYSTIC MASS AT ADAM

SUBJID	INITIAL	ARM	DAY	SKIN	HEAD	EYES	EYES DETAIL	EARS	NOSE	THROAT	NECK	NECK DETAIL
												APPLE AREA DIAMETER 3CM
		Sevuparin/DF02+Malanil	0	Normal	Normal	Normal		Normal	Normal	Normal	Normal	
		Sevuparin/DF02+Malanil	2	Normal	Normal	Normal		Normal	Normal	Normal	Normal	
		Sevuparin/DF02+Malanil	3	Normal	Normal	Normal		Normal	Normal	Normal	Normal	
		Sevuparin/DF02+Malanil	4	Normal	Normal	Normal		Normal	Normal	Normal	Normal	
		Sevuparin/DF02+Malanil	7	Normal	Normal	Normal		Normal	Normal	Normal	Normal	
		Sevuparin/DF02+Malanil	14	Normal	Normal	Normal		Normal	Normal	Normal	Normal	
		Malanil	0	Normal	Normal	Normal		Normal	Normal	Normal	Normal	
		Malanil	2	Normal	Normal	Normal		Normal	Normal	Normal	Normal	
		Malanil	3	Normal	Normal	Normal		Normal	Normal	Normal	Normal	
		Malanil	4	Normal	Normal	Normal		Normal	Normal	Normal	Normal	
		Malanil	7	Normal	Normal	Normal		Normal	Normal	Normal	Normal	
		Sevuparin/DF02+Malanil	0	Normal	Normal	Normal		Normal	Normal	Normal	Normal	
		Sevuparin/DF02+Malanil	2	Normal	Normal	Normal		Normal	Normal	Normal	Normal	
		Sevuparin/DF02+Malanil	3	Normal	Normal	Normal		Normal	Normal	Normal	Normal	
		Sevuparin/DF02+Malanil	4	Normal	Normal	Normal		Normal	Normal	Normal	Normal	
		Sevuparin/DF02+Malanil	7	Normal	Normal	Normal		Normal	Normal	Normal	Normal	
		Sevuparin/DF02+Malanil	14	Normal	Normal	Normal		Normal	Normal	Normal	Normal	
		Sevuparin/DF02+Malanil	0	Normal	Normal	Normal		Normal	Normal	Normal	Normal	
		Sevuparin/DF02+Malanil	2	Normal	Normal	Normal		Normal	Normal	Normal	Normal	
		Sevuparin/DF02+Malanil	3	Normal	Normal	Normal		Normal	Normal	Normal	Normal	
		Sevuparin/DF02+Malanil	4	Normal	Normal	Normal		Normal	Normal	Normal	Normal	

SUBJID	INITIAL	ARM	DAY	SKIN	HEAD	EYES	EYES DETAIL	EARS	NOSE	THROAT	NECK	NECK DETAIL
		Sevuparin/DF02+Malanil	7	Normal	Normal	Normal		Normal	Normal	Normal	Normal	
		Sevuparin/DF02+Malanil	14	Normal	Normal	Normal		Normal	Normal	Normal	Normal	
		Malanil	0	Normal	Normal	Normal		Normal	Normal	Normal	Normal	
		Malanil	2	Normal	Normal	Normal		Normal	Normal	Normal	Normal	
		Malanil	3	Normal	Normal	Normal		Normal	Normal	Normal	Normal	
		Malanil	4	Normal	Normal	Normal		Normal	Normal	Normal	Normal	
		Malanil	7	Normal	Normal	Normal		Normal	Normal	Normal	Normal	
		Malanil	14	Normal	Normal	Normal		Normal	Normal	Normal	Normal	
		Malanil	0	Normal	Normal	Normal		Normal	Normal	Normal	Normal	
		Malanil	2	Normal	Normal	Normal		Normal	Normal	Normal	Normal	
		Malanil	3	Normal	Normal	Normal		Normal	Normal	Normal	Normal	
		Malanil	4	Normal	Normal	Normal		Normal	Normal	Normal	Normal	
		Malanil	7	Normal	Normal	Normal		Normal	Normal	Normal	Normal	
		Malanil	14	Normal	Normal	Normal		Normal	Normal	Normal	Normal	
		Sevuparin/DF02+Malanil	0	Normal	Normal	Normal		Normal	Normal	Normal	Normal	
		Sevuparin/DF02+Malanil	2	Normal	Normal	Normal		Normal	Normal	Normal	Normal	
		Sevuparin/DF02+Malanil	3	Normal	Normal	Normal		Normal	Normal	Normal	Normal	
		Sevuparin/DF02+Malanil	4	Normal	Normal	Normal		Normal	Normal	Normal	Normal	
		Sevuparin/DF02+Malanil	7	Normal	Normal	Normal		Normal	Normal	Normal	Normal	
		Sevuparin/DF02+Malanil	0	Normal	Normal	Normal		Normal	Normal	Normal	Normal	
		Sevuparin/DF02+Malanil	2	Normal	Normal	Normal		Normal	Normal	Normal	Normal	
		Sevuparin/DF02+Malanil	3	Normal	Normal	Normal		Normal	Normal	Normal	Normal	
		Sevuparin/DF02+Malanil	4	Normal	Normal	Normal		Normal	Normal	Normal	Normal	

SUBJID	INITIAL	ARM	DAY	SKIN	HEAD	EYES	EYES DETAIL	EARS	NOSE	THROAT	NECK	NECK DETAIL
		Sevuparin/DF02+Malanil	7	Normal	Normal	Normal		Normal	Normal	Normal	Normal	
		Sevuparin/DF02+Malanil	14	Normal	Normal	Normal		Normal	Normal	Normal	Normal	
		Malanil	0	Normal	Normal	Normal		Normal	Normal	Normal	Normal	
		Malanil	2	Normal	Normal	Normal		Normal	Normal	Normal	Normal	
		Malanil	3	Normal	Normal	Normal		Normal	Normal	Normal	Normal	
		Malanil	4	Normal	Normal	Normal		Normal	Normal	Normal	Normal	
		Malanil	7	Normal	Normal	Normal		Normal	Normal	Normal	Normal	
		Malanil	14	Normal	Normal	Normal		Normal	Normal	Normal	Normal	
		Malanil	0	Normal	Normal	Normal		Normal	Normal	Normal	Normal	
		Malanil	2	Normal	Normal	Normal		Normal	Normal	Normal	Normal	
		Malanil	3	Normal	Normal	Normal		Normal	Normal	Normal	Normal	
		Malanil	4	Normal	Normal	Normal		Normal	Normal	Normal	Normal	
		Malanil	7	Normal	Normal	Normal		Normal	Normal	Normal	Normal	
		Malanil	14	Normal	Normal	Normal		Normal	Normal	Normal	Normal	
		Malanil	0	Normal	Normal	Normal		Normal	Normal	Normal	Normal	
		Malanil	2	Normal	Normal	Normal		Normal	Normal	Normal	Normal	
		Malanil	3	Normal	Normal	Normal		Normal	Normal	Normal	Normal	
		Malanil	4	Normal	Normal	Normal		Normal	Normal	Normal	Normal	
		Malanil	7	Normal	Normal	Normal		Normal	Normal	Normal	Normal	
		Malanil	14	Normal	Normal	Normal		Normal	Normal	Normal	Normal	
		Sevuparin/DF02+Malanil	0	Normal	Normal	Normal		Normal	Normal	Normal	Normal	
		Sevuparin/DF02+Malanil	2	Normal	Normal	Normal		Normal	Normal	Normal	Normal	
		Sevuparin/DF02+Malanil	3	Normal	Normal	Normal		Normal	Normal	Normal	Normal	

SUBJID	INITIAL	ARM	DAY	SKIN	HEAD	EYES	EYES DETAIL	EARS	NOSE	THROAT	NECK	NECK DETAIL
		Sevuparin/DF02+Malanil	4	Normal	Normal	Normal		Normal	Normal	Normal	Normal	
		Sevuparin/DF02+Malanil	7	Normal	Normal	Normal		Normal	Normal	Normal	Normal	
		Sevuparin/DF02+Malanil	14	Normal	Normal	Normal		Normal	Normal	Normal	Normal	
		Sevuparin/DF02+Malanil	0	Normal	Normal	Normal		Normal	Normal	Normal	Normal	
		Sevuparin/DF02+Malanil	2	Normal	Normal	Normal		Normal	Normal	Normal	Normal	
		Sevuparin/DF02+Malanil	3	Normal	Normal	Normal		Normal	Normal	Normal	Normal	
		Sevuparin/DF02+Malanil	4	Normal	Normal	Normal		Normal	Normal	Normal	Normal	
		Sevuparin/DF02+Malanil	7	Normal	Normal	Normal		Normal	Normal	Normal	Normal	
		Sevuparin/DF02+Malanil	14	Normal	Normal	Normal		Normal	Normal	Normal	Normal	
		Sevuparin/DF02+Malanil	0	Normal	Normal	Normal		Normal	Normal	Normal	Normal	
		Sevuparin/DF02+Malanil	2	Normal	Normal	Normal		Normal	Normal	Normal	Normal	
		Sevuparin/DF02+Malanil	3	Normal	Normal	Normal		Normal	Normal	Normal	Normal	
		Sevuparin/DF02+Malanil	4	Normal	Normal	Normal		Normal	Normal	Normal	Normal	
		Sevuparin/DF02+Malanil	7	Normal	Normal	Normal		Normal	Normal	Normal	Normal	
		Sevuparin/DF02+Malanil	14	Normal	Normal	Normal		Normal	Normal	Normal	Normal	
		Malanil	0	Normal	Normal	Normal		Normal	Normal	Normal	Normal	
		Malanil	2	Normal	Normal	Normal		Normal	Normal	Normal	Normal	
		Malanil	3	Normal	Normal	Normal		Normal	Normal	Normal	Normal	
		Malanil	4	Normal	Normal	Normal		Normal	Normal	Normal	Normal	
		Malanil	7	Normal	Normal	Normal		Normal	Normal	Normal	Normal	
		Malanil	14	Normal	Normal	Normal		Normal	Normal	Normal	Normal	
		Sevuparin/DF02+Malanil	0	Normal	Normal	Normal		Normal	Normal	Normal	Normal	
		Sevuparin/DF02+Malanil	2	Normal	Normal	Normal		Normal	Normal	Normal	Normal	

SUBJID	INITIAL	ARM	DAY	SKIN	HEAD	EYES	EYES DETAIL	EARS	NOSE	THROAT	NECK	NECK DETAIL
		Sevuparin/DF02+Malanil	3	Normal	Normal	Normal		Normal	Normal	Normal	Normal	
		Sevuparin/DF02+Malanil	4	Normal	Normal	Normal		Normal	Normal	Normal	Normal	
		Sevuparin/DF02+Malanil	7	Normal	Normal	Normal		Normal	Normal	Normal	Normal	
		Sevuparin/DF02+Malanil	14	Normal	Normal	Normal		Normal	Normal	Normal	Normal	
		Sevuparin/DF02+Malanil	0	Normal	Normal	Abnormal	MODERATE JAUNDICE	Normal	Normal	Normal	Normal	
		Sevuparin/DF02+Malanil	2	Normal	Normal	Abnormal	MILD JAUNDICE	Normal	Normal	Normal	Normal	
		Sevuparin/DF02+Malanil	3	Normal	Normal	Abnormal	MILD JAUNDICE	Normal	Normal	Normal	Normal	
		Sevuparin/DF02+Malanil	4	Normal	Normal	Normal		Normal	Normal	Normal	Normal	
		Sevuparin/DF02+Malanil	7	Normal	Normal	Normal		Normal	Normal	Normal	Normal	
		Sevuparin/DF02+Malanil	14	Normal	Normal	Normal		Normal	Normal	Normal	Normal	
		Sevuparin/DF02+Malanil	0	Normal	Normal	Normal		Normal	Normal	Normal	Normal	
		Sevuparin/DF02+Malanil	2	Normal	Normal	Normal		Normal	Normal	Normal	Normal	
		Sevuparin/DF02+Malanil	3	Normal	Normal	Normal		Normal	Normal	Normal	Normal	
		Sevuparin/DF02+Malanil	4	Normal	Normal	Normal		Normal	Normal	Normal	Normal	
		Sevuparin/DF02+Malanil	7	Normal	Normal	Normal		Normal	Normal	Normal	Normal	
		Sevuparin/DF02+Malanil	14	Normal	Normal	Normal		Normal	Normal	Normal	Normal	
		Sevuparin/DF02+Malanil	0	Normal	Normal	Abnormal	BLINDNESS LEFT EYE	Normal	Normal	Normal	Normal	
		Sevuparin/DF02+Malanil	2	Normal	Normal	Abnormal	BLINDNESS LEFT EYE	Normal	Normal	Normal	Normal	
		Sevuparin/DF02+Malanil	3	Normal	Normal	Abnormal	BLINDNESS LEFT EYE	Normal	Normal	Normal	Normal	

SUBJID	INITIAL	ARM	DAY	SKIN	HEAD	EYES	EYES DETAIL	EARS	NOSE	THROAT	NECK	NECK DETAIL
		Sevuparin/DF02+Malanil	4	Normal	Normal	Abnormal	BLINDNESS LEFT EYE	Normal	Normal	Normal	Normal	
		Sevuparin/DF02+Malanil	7	Normal	Normal	Abnormal	BLINDNESS LEFT EYE	Normal	Normal	Normal	Normal	
		Sevuparin/DF02+Malanil	14	Normal	Normal	Abnormal	BLINDNESS LEFT EYE	Normal	Normal	Normal	Normal	
		Malanil	0	Normal	Normal	Normal		Normal	Normal	Normal	Normal	
		Malanil	2	Normal	Normal	Normal		Normal	Normal	Normal	Normal	
		Malanil	3	Normal	Normal	Normal		Normal	Normal	Normal	Normal	
		Malanil	4	Normal	Normal	Normal		Normal	Normal	Normal	Normal	
		Malanil	7	Normal	Normal	Normal		Normal	Normal	Normal	Normal	
		Malanil	14	Normal	Normal	Normal		Normal	Normal	Normal	Normal	
		Malanil	0	Normal	Normal	Normal		Normal	Normal	Normal	Normal	
		Malanil	2	Normal	Normal	Normal		Normal	Normal	Normal	Normal	
		Malanil	3	Normal	Normal	Normal		Normal	Normal	Normal	Normal	
		Malanil	4	Normal	Normal	Normal		Normal	Normal	Normal	Normal	
		Malanil	7	Normal	Normal	Normal		Normal	Normal	Normal	Normal	
		Malanil	14	Normal	Normal	Normal		Normal	Normal	Normal	Normal	
		Malanil	0	Normal	Normal	Normal		Normal	Normal	Normal	Normal	
		Malanil	2	Normal	Normal	Normal		Normal	Normal	Normal	Normal	
		Malanil	3	Normal	Normal	Normal		Normal	Normal	Normal	Normal	
		Malanil	4	Normal	Normal	Normal		Normal	Normal	Normal	Normal	
		Malanil	7	Normal	Normal	Normal		Normal	Normal	Normal	Normal	
		Malanil	14	Normal	Normal	Normal		Normal	Normal	Normal	Normal	

SUBJID	INITIAL	ARM	DAY	SKIN	HEAD	EYES	EYES DETAIL	EARS	NOSE	THROAT	NECK	NECK DETAIL
		Sevuparin/DF02+Malanil	0	Normal	Normal	Normal		Normal	Normal	Normal	Normal	
		Sevuparin/DF02+Malanil	2	Normal	Normal	Normal		Normal	Normal	Normal	Normal	
		Sevuparin/DF02+Malanil	3	Normal	Normal	Normal		Normal	Normal	Normal	Normal	
		Sevuparin/DF02+Malanil	4	Normal	Normal	Normal		Normal	Normal	Normal	Normal	
		Sevuparin/DF02+Malanil	7	Normal	Normal	Normal		Normal	Normal	Normal	Normal	
		Sevuparin/DF02+Malanil	14	Normal	Normal	Normal		Normal	Normal	Normal	Normal	
		Malanil	0	Normal	Normal	Normal		Normal	Normal	Normal	Normal	
		Malanil	2	Normal	Normal	Normal		Normal	Normal	Normal	Normal	
		Malanil	3	Normal	Normal	Normal		Normal	Normal	Normal	Normal	
		Malanil	4	Normal	Normal	Normal		Normal	Normal	Normal	Normal	
		Malanil	7	Normal	Normal	Normal		Normal	Normal	Normal	Normal	
		Malanil	14	Normal	Normal	Normal		Normal	Normal	Normal	Normal	
		Malanil	0	Normal	Normal	Normal		Normal	Normal	Normal	Normal	
		Malanil	2	Normal	Normal	Normal		Normal	Normal	Normal	Normal	
		Malanil	3	Normal	Normal	Normal		Normal	Normal	Normal	Normal	
		Malanil	4	Normal	Normal	Normal		Normal	Normal	Normal	Normal	
		Malanil	7	Normal	Normal	Normal		Normal	Normal	Normal	Normal	
		Malanil	14	Normal	Normal	Normal		Normal	Normal	Normal	Normal	
		Malanil	0	Normal	Normal	Normal		Normal	Normal	Normal	Normal	
		Malanil	2	Normal	Normal	Normal		Normal	Normal	Normal	Normal	
		Malanil	3	Normal	Normal	Normal		Normal	Normal	Normal	Normal	
		Malanil	4	Normal	Normal	Normal		Normal	Normal	Normal	Normal	
		Malanil	7	Normal	Normal	Normal		Normal	Normal	Normal	Normal	

SUBJID	INITIAL	ARM	DAY	SKIN	HEAD	EYES	EYES DETAIL	EARS	NOSE	THROAT	NECK	NECK DETAIL
		Malanil	14	Normal	Normal	Normal		Normal	Normal	Normal	Normal	
		Malanil	0	Normal	Normal	Normal		Normal	Normal	Normal	Normal	
		Malanil	2	Normal	Normal	Normal		Normal	Normal	Normal	Normal	
		Malanil	3	Normal	Normal	Normal		Normal	Normal	Normal	Normal	
		Malanil	4	Normal	Normal	Normal		Normal	Normal	Normal	Normal	
		Malanil	7	Normal	Normal	Normal		Normal	Normal	Normal	Normal	
		Malanil	14	Normal	Normal	Normal		Normal	Normal	Normal	Normal	
		Sevuparin/DF02+Malanil	0	Normal	Normal	Normal		Normal	Normal	Normal	Normal	
		Sevuparin/DF02+Malanil	2	Normal	Normal	Normal		Normal	Normal	Normal	Normal	
		Sevuparin/DF02+Malanil	3	Normal	Normal	Normal		Normal	Normal	Normal	Normal	
		Sevuparin/DF02+Malanil	4	Normal	Normal	Normal		Normal	Normal	Normal	Normal	
		Sevuparin/DF02+Malanil	7	Normal	Normal	Normal		Normal	Normal	Normal	Normal	
		Sevuparin/DF02+Malanil	14	Normal	Normal	Normal		Normal	Normal	Normal	Normal	
		Malanil	0	Normal	Normal	Normal		Normal	Normal	Normal	Normal	
		Malanil	2	Normal	Normal	Normal		Normal	Normal	Normal	Normal	
		Malanil	3	Normal	Normal	Normal		Normal	Normal	Normal	Normal	
		Malanil	4	Normal	Normal	Normal		Normal	Normal	Normal	Normal	
		Malanil	7	Normal	Normal	Normal		Normal	Normal	Normal	Normal	
		Malanil	14	Normal	Normal	Normal		Normal	Normal	Normal	Normal	
		Malanil	0	Normal	Normal	Normal		Normal	Normal	Normal	Normal	
		Malanil	2	Normal	Normal	Normal		Normal	Normal	Normal	Normal	
		Malanil	3	Normal	Normal	Normal		Normal	Normal	Normal	Normal	
		Malanil	4	Normal	Normal	Normal		Normal	Normal	Normal	Normal	

SUBJID	INITIAL	ARM	DAY	SKIN	HEAD	EYES	EYES DETAIL	EARS	NOSE	THROAT	NECK	NECK DETAIL
		Malanil	7	Normal	Normal	Normal		Normal	Normal	Normal	Normal	
		Malanil	14	Normal	Normal	Normal		Normal	Normal	Normal	Normal	
		Malanil	0	Normal	Normal	Normal		Normal	Normal	Normal	Normal	
		Malanil	2	Normal	Normal	Normal		Normal	Normal	Normal	Normal	
		Malanil	3	Normal	Normal	Normal		Normal	Normal	Normal	Normal	
		Malanil	4	Normal	Normal	Normal		Normal	Normal	Normal	Normal	
		Malanil	7	Normal	Normal	Normal		Normal	Normal	Normal	Normal	
		Malanil	14	Normal	Normal	Normal		Normal	Normal	Normal	Normal	
		Sevuparin/DF02+Malanil	0	Normal	Normal	Abnormal	JAUNDICE	Normal	Normal	Normal	Normal	
		Sevuparin/DF02+Malanil	2	Normal	Normal	Abnormal	JAUNDICE	Normal	Normal	Normal	Normal	
		Sevuparin/DF02+Malanil	3	Normal	Normal	Abnormal	JAUNDICE	Normal	Normal	Normal	Normal	
		Sevuparin/DF02+Malanil	4	Normal	Normal	Abnormal	JAUNDICE	Normal	Normal	Normal	Normal	
		Sevuparin/DF02+Malanil	7	Normal	Normal	Normal		Normal	Normal	Normal	Normal	
		Sevuparin/DF02+Malanil	14	Normal	Normal	Normal		Normal	Normal	Normal	Normal	
		Sevuparin/DF02+Malanil	0	Normal	Normal	Normal		Normal	Normal	Normal	Normal	
		Sevuparin/DF02+Malanil	2	Normal	Normal	Normal		Normal	Normal	Normal	Normal	
		Sevuparin/DF02+Malanil	3	Normal	Normal	Normal		Normal	Normal	Normal	Normal	
		Sevuparin/DF02+Malanil	4	Normal	Normal	Normal		Normal	Normal	Normal	Normal	
		Sevuparin/DF02+Malanil	7	Normal	Normal	Normal		Normal	Normal	Normal	Normal	
		Sevuparin/DF02+Malanil	14	Normal	Normal	Normal		Normal	Normal	Normal	Normal	
		Sevuparin/DF02+Malanil	0	Normal	Normal	Normal		Normal	Normal	Normal	Normal	
		Sevuparin/DF02+Malanil	1	Normal	Normal	Normal		Normal	Normal	Normal	Normal	
		Sevuparin/DF02+Malanil	2	Normal	Normal	Normal		Normal	Normal	Normal	Normal	

SUBJID	INITIAL	ARM	DAY	SKIN	HEAD	EYES	EYES DETAIL	EARS	NOSE	THROAT	NECK	NECK DETAIL
		Sevuparin/DF02+Malanil	3	Normal	Normal	Normal		Normal	Normal	Normal	Normal	
		Sevuparin/DF02+Malanil	4	Normal	Normal	Normal		Normal	Normal	Normal	Normal	
		Sevuparin/DF02+Malanil	7	Normal	Normal	Normal		Normal	Normal	Normal	Normal	
		Sevuparin/DF02+Malanil	14	Normal	Normal	Normal		Normal	Normal	Normal	Normal	
		Sevuparin/DF02+Malanil	0	Normal	Normal	Normal		Normal	Normal	Normal	Normal	
		Sevuparin/DF02+Malanil	2	Normal	Normal	Normal		Normal	Normal	Normal	Normal	
		Sevuparin/DF02+Malanil	3	Normal	Normal	Normal		Normal	Normal	Normal	Normal	
		Sevuparin/DF02+Malanil	4	Normal	Normal	Normal		Normal	Normal	Normal	Normal	
		Sevuparin/DF02+Malanil	7	Normal	Normal	Normal		Normal	Normal	Normal	Normal	
		Sevuparin/DF02+Malanil	14	Normal	Normal	Normal		Normal	Normal	Normal	Normal	
		Sevuparin/DF02+Malanil	0	Normal	Normal	Normal		Normal	Normal	Normal	Normal	
		Sevuparin/DF02+Malanil	2	Normal	Normal	Normal		Normal	Normal	Normal	Normal	
		Sevuparin/DF02+Malanil	3	Normal	Normal	Normal		Normal	Normal	Normal	Normal	
		Sevuparin/DF02+Malanil	4	Normal	Normal	Normal		Normal	Normal	Normal	Normal	
		Sevuparin/DF02+Malanil	7	Normal	Normal	Normal		Normal	Normal	Normal	Normal	
		Sevuparin/DF02+Malanil	14	Normal	Normal	Normal		Normal	Normal	Normal	Normal	
		Malanil	0	Normal	Normal	Normal		Normal	Normal	Normal	Normal	
		Malanil	2	Normal	Normal	Normal		Normal	Normal	Normal	Normal	
		Malanil	3	Normal	Normal	Normal		Normal	Normal	Normal	Normal	
		Malanil	4	Normal	Normal	Normal		Normal	Normal	Normal	Normal	
		Malanil	7	Normal	Normal	Normal		Normal	Normal	Normal	Normal	
		Malanil	14	Normal	Normal	Normal		Normal	Normal	Normal	Normal	
		Malanil	0	Normal	Normal	Normal		Normal	Normal	Normal	Normal	

SUBJID	INITIAL	ARM	DAY	SKIN	HEAD	EYES	EYES DETAIL	EARS	NOSE	THROAT	NECK	NECK DETAIL
		Malanil	2	Normal	Normal	Normal		Normal	Normal	Normal	Normal	
		Malanil	3	Normal	Normal	Normal		Normal	Normal	Normal	Normal	
		Malanil	4	Normal	Normal	Normal		Normal	Normal	Normal	Normal	
		Malanil	7	Normal	Normal	Normal		Normal	Normal	Normal	Normal	
		Malanil	14	Normal	Normal	Normal		Normal	Normal	Normal	Normal	
		Malanil	0	Normal	Normal	Normal		Normal	Normal	Normal	Normal	
		Malanil	2	Normal	Normal	Normal		Normal	Normal	Normal	Normal	
		Malanil	3	Normal	Normal	Normal		Normal	Normal	Normal	Normal	
		Malanil	4	Normal	Normal	Normal		Normal	Normal	Normal	Normal	
		Malanil	5	Normal	Normal	Normal		Normal	Normal	Normal	Normal	
		Malanil	7	Normal	Normal	Normal		Normal	Normal	Normal	Normal	
		Malanil	14	Normal	Normal	Normal		Normal	Normal	Normal	Normal	

16.2.7.3 Physical examination (including weight and height) (-continued)

SUBJID	INITIAL	ARM	DAY	THYROID	THY DETAIL	LUNG	HEART	ABDOMEN	ABD DETAIL	LIVER (cm)	SPLEEN (cm)	LYMPH	EXTREMITIES	CNS
		Malanil	0	Normal		Normal	Normal	Abnormal		1	4	Normal	Normal	Normal
		Malanil	2	Normal		Normal	Normal	Abnormal	NO SIGNIFICANT	1	4	Normal	Normal	Normal
		Malanil	3	Normal		Normal	Normal	Abnormal	NO SIGNIFICANT	1	4	Normal	Normal	Normal
		Malanil	4	Normal		Normal	Normal	Abnormal	NO SIGNIFICANT	1	4	Normal	Normal	Normal
		Sevuparin/DF02 +Malanil	0	Normal		Normal	Normal	Normal				Normal	Normal	Normal
		Sevuparin/DF02 +Malanil	2	Normal		Normal	Normal	Normal				Normal	Normal	Normal
		Sevuparin/DF02 +Malanil	3	Normal		Normal	Normal	Normal				Normal	Normal	Normal
		Sevuparin/DF02 +Malanil	4	Normal		Normal	Normal	Normal				Normal	Normal	Normal
		Sevuparin/DF02 +Malanil	7	Normal		Normal	Normal	Normal				Normal	Normal	Normal
		Sevuparin/DF02 +Malanil	14	Normal		Normal	Normal	Normal				Normal	Normal	Normal
		Sevuparin/DF02 +Malanil	0	Normal		Normal	Normal	Normal				Normal	Normal	Normal
		Sevuparin/DF02 +Malanil	2	Normal		Normal	Normal	Normal				Normal	Normal	Normal
		Sevuparin/DF02 +Malanil	3	Normal		Normal	Normal	Normal				Normal	Normal	Normal
		Sevuparin/DF02	4	Normal		Normal	Normal	Normal				Normal	Normal	Normal

SUBJID	INITIAL	ARM	DAY	THYROID	THY DETAIL	LUNG	HEART	ABDOMEN	ABD DETAIL	LIVER (cm)	SPLEEN (cm)	LYMPH	EXTREMITIES	CNS
		+Malanil												
		Sevuparin/DF02 +Malanil	7	Normal		Normal	Normal	Normal				Normal	Normal	Normal
		Sevuparin/DF02 +Malanil	14	Normal		Normal	Normal	Normal				Normal	Normal	Normal
		Malanil	0	Abnormal	THYROID GRAD II DIAMETER 6CM	Normal	Normal	Normal				Normal	Normal	Normal
		Malanil	2	Abnormal	THYROID GRAD II DIAMETER 6CM-NSC	Normal	Normal	Normal				Normal	Normal	Normal
		Malanil	3	Abnormal	THYROID GRAD II DIAMETER 6CM-NSC	Normal	Normal	Normal				Normal	Normal	Normal
		Malanil	4	Abnormal	THYROID GRAD II DIAMETER 6CM	Normal	Normal	Normal				Normal	Normal	Normal
		Malanil	7	Abnormal	THYROID GRAD II DIAMETER 6CM-NSC	Normal	Normal	Normal				Normal	Normal	Normal
		Malanil	14	Abnormal	THYROID GRAD II DIAMETER 6CM-NSC	Normal	Normal	Normal				Normal	Normal	Normal
		Malanil	0	Normal		Normal	Normal	Normal				Normal	Normal	Normal

SUBJID	INITIAL	ARM	DAY	THYROID	THY DETAIL	LUNG	HEART	ABDOMEN	ABD DETAIL	LIVER (cm)	SPLEEN (cm)	LYMPH	EXTREMITIES	CNS
		Malanil	2	Normal		Normal	Normal	Normal				Normal	Normal	Normal
		Malanil	3	Normal		Normal	Normal	Normal				Normal	Normal	Normal
		Malanil	4	Normal		Normal	Normal	Normal				Normal	Normal	Normal
		Malanil	7	Normal		Normal	Normal	Normal				Normal	Normal	Normal
		Malanil	14	Normal		Normal	Normal	Normal				Normal	Normal	Normal
		Sevuparin/DF02 +Malanil	0	Normal		Normal	Normal	Normal				Normal	Normal	Normal
		Sevuparin/DF02 +Malanil	2	Normal		Normal	Normal	Normal				Normal	Normal	Normal
		Sevuparin/DF02 +Malanil	3	Normal		Normal	Normal	Normal				Normal	Normal	Normal
		Sevuparin/DF02 +Malanil	4	Normal		Normal	Normal	Normal				Normal	Normal	Normal
		Sevuparin/DF02 +Malanil	7	Normal		Normal	Normal	Normal				Normal	Normal	Normal
		Sevuparin/DF02 +Malanil	14	Normal		Normal	Normal	Normal				Normal	Normal	Normal
		Malanil	0	Normal		Normal	Normal	Normal				Normal	Normal	Normal
		Malanil	2	Normal		Normal	Normal	Normal				Normal	Normal	Normal
		Malanil	3	Normal		Normal	Normal	Normal				Normal	Normal	Normal
		Malanil	4	Normal		Normal	Normal	Normal				Normal	Normal	Normal
		Malanil	7	Normal		Normal	Normal	Normal				Normal	Normal	Normal
		Sevuparin/DF02 +Malanil	0	Normal		Normal	Normal	Normal				Normal	Normal	Normal
		Sevuparin/DF02 +Malanil	2	Normal		Normal	Normal	Normal				Normal	Normal	Normal

SUBJID	INITIAL	ARM	DAY	THYROID	THY DETAIL	LUNG	HEART	ABDOMEN	ABD DETAIL	LIVER (cm)	SPLEEN (cm)	LYMPH	EXTREMITIES	CNS
		Sevuparin/DF02 +Malanil	3	Normal		Normal	Normal	Normal				Normal	Normal	Normal
		Sevuparin/DF02 +Malanil	4	Normal		Normal	Normal	Normal				Normal	Normal	Normal
		Sevuparin/DF02 +Malanil	7	Normal		Normal	Normal	Normal				Normal	Normal	Normal
		Sevuparin/DF02 +Malanil	14	Normal		Normal	Normal	Normal				Normal	Normal	Normal
		Sevuparin/DF02 +Malanil	0	Normal		Normal	Normal	Normal				Normal	Normal	Normal
		Sevuparin/DF02 +Malanil	2	Normal		Normal	Normal	Normal				Normal	Normal	Normal
		Sevuparin/DF02 +Malanil	3	Normal		Normal	Normal	Normal				Normal	Normal	Normal
		Sevuparin/DF02 +Malanil	4	Normal		Normal	Normal	Normal				Normal	Normal	Normal
		Sevuparin/DF02 +Malanil	7	Normal		Normal	Normal	Normal				Normal	Normal	Normal
		Sevuparin/DF02 +Malanil	14	Normal		Normal	Normal	Normal				Normal	Normal	Normal
		Malanil	0	Normal		Normal	Normal	Normal				Normal	Normal	Normal
		Malanil	2	Normal		Normal	Normal	Normal				Normal	Normal	Normal
		Malanil	3	Normal		Normal	Normal	Normal				Normal	Normal	Normal
		Malanil	4	Normal		Normal	Normal	Normal				Normal	Normal	Normal
		Malanil	7	Normal		Normal	Normal	Normal				Normal	Normal	Normal
		Malanil	14	Normal		Normal	Normal	Normal				Normal	Normal	Normal

SUBJID	INITIAL	ARM	DAY	THYROID	THY DETAIL	LUNG	HEART	ABDOMEN	ABD DETAIL	LIVER (cm)	SPLEEN (cm)	LYMPH	EXTREMITIES	CNS
		Malanil	0	Normal		Normal	Normal	Abnormal			2	Normal	Normal	Normal
		Malanil	2	Normal		Normal	Normal	Abnormal			2	Normal	Normal	Normal
		Malanil	3	Normal		Normal	Normal	Abnormal			2	Normal	Normal	Normal
		Malanil	4	Normal		Normal	Normal	Abnormal			2	Normal	Normal	Normal
		Malanil	7	Normal		Normal	Normal	Abnormal			2	Normal	Normal	Normal
		Malanil	14	Normal		Normal	Normal	Abnormal			2	Normal	Normal	Normal
		Sevuparin/DF02 +Malanil	0	Normal		Normal	Normal	Normal				Normal	Normal	Normal
		Sevuparin/DF02 +Malanil	2	Normal		Normal	Normal	Normal				Normal	Normal	Normal
		Sevuparin/DF02 +Malanil	3	Normal		Normal	Normal	Normal				Normal	Normal	Normal
		Sevuparin/DF02 +Malanil	4	Normal		Normal	Normal	Normal				Normal	Normal	Normal
		Sevuparin/DF02 +Malanil	7	Normal		Normal	Normal	Normal				Normal	Normal	Normal
		Sevuparin/DF02 +Malanil	0	Normal		Normal	Normal	Abnormal			4	Normal	Normal	Normal
		Sevuparin/DF02 +Malanil	2	Normal		Normal	Normal	Abnormal			4	Normal	Normal	Normal
		Sevuparin/DF02 +Malanil	3	Normal		Normal	Normal	Abnormal			4	Normal	Normal	Normal
		Sevuparin/DF02 +Malanil	4	Normal		Normal	Normal	Abnormal			4	Normal	Normal	Normal
		Sevuparin/DF02 +Malanil	7	Normal		Normal	Normal	Abnormal			4	Normal	Normal	Normal

SUBJID	INITIAL	ARM	DAY	THYROID	THY DETAIL	LUNG	HEART	ABDOMEN	ABD DETAIL	LIVER (cm)	SPLEEN (cm)	LYMPH	EXTREMITIES	CNS
		Sevuparin/DF02 +Malanil	14	Normal		Normal	Normal	Abnormal			4	Normal	Normal	Normal
		Malanil	0	Normal		Normal	Normal	Normal				Normal	Normal	Normal
		Malanil	2	Normal		Normal	Normal	Normal				Normal	Normal	Normal
		Malanil	3	Normal		Normal	Normal	Normal				Normal	Normal	Normal
		Malanil	4	Normal		Normal	Normal	Normal				Normal	Normal	Normal
		Malanil	7	Normal		Normal	Normal	Normal				Normal	Normal	Normal
		Malanil	14	Normal		Normal	Normal	Normal				Normal	Normal	Normal
		Malanil	0	Normal		Normal	Normal	Normal				Normal	Normal	Normal
		Malanil	2	Normal		Normal	Normal	Normal				Normal	Normal	Normal
		Malanil	3	Normal		Normal	Normal	Normal				Normal	Normal	Normal
		Malanil	4	Normal		Normal	Normal	Normal				Normal	Normal	Normal
		Malanil	7	Normal		Normal	Normal	Normal				Normal	Normal	Normal
		Malanil	14	Normal		Normal	Normal	Normal				Normal	Normal	Normal
		Malanil	0	Normal		Normal	Normal	Normal				Normal	Normal	Normal
		Malanil	2	Normal		Normal	Normal	Normal				Normal	Normal	Normal
		Malanil	3	Normal		Normal	Normal	Normal				Normal	Normal	Normal
		Malanil	4	Normal		Normal	Normal	Normal				Normal	Normal	Normal
		Malanil	7	Normal		Normal	Normal	Normal				Normal	Normal	Normal
		Malanil	14	Normal		Normal	Normal	Normal				Normal	Normal	Normal
		Sevuparin/DF02 +Malanil	0	Normal		Normal	Normal	Normal				Normal	Normal	Normal
		Sevuparin/DF02 +Malanil	2	Normal		Normal	Normal	Normal				Normal	Normal	Normal

SUBJID	INITIAL	ARM	DAY	THYROID	THY DETAIL	LUNG	HEART	ABDOMEN	ABD DETAIL	LIVER (cm)	SPLEEN (cm)	LYMPH	EXTREMITIES	CNS
		Sevuparin/DF02 +Malanil	3	Normal		Normal	Normal	Normal				Normal	Normal	Normal
		Sevuparin/DF02 +Malanil	4	Normal		Normal	Normal	Normal				Normal	Normal	Normal
		Sevuparin/DF02 +Malanil	7	Normal		Normal	Normal	Normal				Normal	Normal	Normal
		Sevuparin/DF02 +Malanil	14	Normal		Normal	Normal	Normal				Normal	Normal	Normal
		Sevuparin/DF02 +Malanil	0	Normal		Normal	Normal	Normal				Normal	Normal	Normal
		Sevuparin/DF02 +Malanil	2	Normal		Normal	Normal	Normal				Normal	Normal	Normal
		Sevuparin/DF02 +Malanil	3	Normal		Normal	Normal	Normal				Normal	Normal	Normal
		Sevuparin/DF02 +Malanil	4	Normal		Normal	Normal	Normal				Normal	Normal	Normal
		Sevuparin/DF02 +Malanil	7	Normal		Normal	Normal	Normal				Normal	Normal	Normal
		Sevuparin/DF02 +Malanil	14	Normal		Normal	Normal	Normal				Normal	Normal	Normal
		Sevuparin/DF02 +Malanil	0	Normal		Normal	Normal	Abnormal	SOFT MILD TENDERNES S			Normal	Normal	Normal
		Sevuparin/DF02 +Malanil	2	Normal		Normal	Normal	Normal				Normal	Normal	Normal
		Sevuparin/DF02 +Malanil	3	Normal		Normal	Normal	Normal				Normal	Normal	Normal
		Sevuparin/DF02	4	Normal		Normal	Normal	Normal				Normal	Normal	Normal

SUBJID	INITIAL	ARM	DAY	THYROID	THY DETAIL	LUNG	HEART	ABDOMEN	ABD DETAIL	LIVER (cm)	SPLEEN (cm)	LYMPH	EXTREMITIES	CNS
		+Malanil												
		Sevuparin/DF02 +Malanil	7	Normal		Normal	Normal	Normal				Normal	Normal	Normal
		Sevuparin/DF02 +Malanil	14	Normal		Normal	Normal	Normal				Normal	Normal	Normal
		Malanil	0	Normal		Normal	Normal	Normal				Normal	Normal	Normal
		Malanil	2	Normal		Normal	Normal	Normal				Normal	Normal	Normal
		Malanil	3	Normal		Normal	Normal	Normal				Normal	Normal	Normal
		Malanil	4	Normal		Normal	Normal	Normal				Normal	Normal	Normal
		Malanil	7	Normal		Normal	Normal	Normal				Normal	Normal	Normal
		Malanil	14	Normal		Normal	Normal	Normal				Normal	Normal	Normal
		Sevuparin/DF02 +Malanil	0	Normal		Normal	Normal	Normal				Normal	Normal	Normal
		Sevuparin/DF02 +Malanil	2	Normal		Normal	Normal	Normal				Normal	Normal	Normal
		Sevuparin/DF02 +Malanil	3	Normal		Normal	Normal	Normal				Normal	Normal	Normal
		Sevuparin/DF02 +Malanil	4	Normal		Normal	Normal	Normal				Normal	Normal	Normal
		Sevuparin/DF02 +Malanil	7	Normal		Normal	Normal	Normal				Normal	Normal	Normal
		Sevuparin/DF02 +Malanil	14	Normal		Normal	Normal	Normal				Normal	Normal	Normal
		Sevuparin/DF02 +Malanil	0	Normal		Normal	Normal	Normal				Normal	Normal	Normal
		Sevuparin/DF02	2	Normal		Normal	Normal	Normal				Normal	Normal	Normal

SUBJID	INITIAL	ARM	DAY	THYROID	THY DETAIL	LUNG	HEART	ABDOMEN	ABD DETAIL	LIVER (cm)	SPLEEN (cm)	LYMPH	EXTREMITIES	CNS
		+Malanil												
		Sevuparin/DF02 +Malanil	3	Normal		Normal	Normal	Normal				Normal	Normal	Normal
		Sevuparin/DF02 +Malanil	4	Normal		Normal	Normal	Normal				Normal	Normal	Normal
		Sevuparin/DF02 +Malanil	7	Normal		Normal	Normal	Normal				Normal	Normal	Normal
		Sevuparin/DF02 +Malanil	14	Normal		Normal	Normal	Normal				Normal	Normal	Normal
		Sevuparin/DF02 +Malanil	0	Normal		Normal	Normal	Abnormal		2	2	Normal	Normal	Normal
		Sevuparin/DF02 +Malanil	2	Normal		Normal	Normal	Abnormal		2	2	Normal	Normal	Normal
		Sevuparin/DF02 +Malanil	3	Normal		Normal	Normal	Abnormal		2	2	Normal	Normal	Normal
		Sevuparin/DF02 +Malanil	4	Normal		Normal	Normal	Abnormal		2	2	Normal	Normal	Normal
		Sevuparin/DF02 +Malanil	7	Normal		Normal	Normal	Abnormal		2	2	Normal	Normal	Normal
		Sevuparin/DF02 +Malanil	14	Normal		Normal	Normal	Abnormal		2	2	Normal	Normal	Normal
		Sevuparin/DF02 +Malanil	0	Normal		Normal	Normal	Normal				Normal	Normal	Normal
		Sevuparin/DF02 +Malanil	2	Normal		Normal	Normal	Normal				Normal	Normal	Normal
		Sevuparin/DF02 +Malanil	3	Normal		Normal	Normal	Normal				Normal	Normal	Normal

SUBJID	INITIAL	ARM	DAY	THYROID	THY DETAIL	LUNG	HEART	ABDOMEN	ABD DETAIL	LIVER (cm)	SPLEEN (cm)	LYMPH	EXTREMITIES	CNS
		Sevuparin/DF02 +Malanil	4	Normal		Normal	Normal	Normal				Normal	Normal	Normal
		Sevuparin/DF02 +Malanil	7	Normal		Normal	Normal	Normal				Normal	Normal	Normal
		Sevuparin/DF02 +Malanil	14	Normal		Normal	Normal	Normal				Normal	Normal	Normal
		Malanil	0	Normal		Normal	Normal	Normal				Normal	Normal	Normal
		Malanil	2	Normal		Normal	Normal	Normal				Normal	Normal	Normal
		Malanil	3	Normal		Normal	Normal	Normal				Normal	Normal	Normal
		Malanil	4	Normal		Normal	Normal	Normal				Normal	Normal	Normal
		Malanil	7	Normal		Normal	Normal	Normal				Normal	Normal	Normal
		Malanil	14	Normal		Normal	Normal	Normal				Normal	Normal	Normal
		Malanil	0	Normal		Normal	Normal	Normal				Normal	Normal	Normal
		Malanil	2	Normal		Normal	Normal	Normal				Normal	Normal	Normal
		Malanil	3	Normal		Normal	Normal	Normal				Normal	Normal	Normal
		Malanil	4	Normal		Normal	Normal	Normal				Normal	Normal	Normal
		Malanil	7	Normal		Normal	Normal	Normal				Normal	Normal	Normal
		Malanil	14	Normal		Normal	Normal	Normal				Normal	Normal	Normal
		Malanil	0	Normal		Normal	Normal	Normal				Normal	Normal	Normal
		Malanil	2	Normal		Normal	Normal	Normal				Normal	Normal	Normal
		Malanil	3	Normal		Normal	Normal	Normal				Normal	Normal	Normal
		Malanil	4	Normal		Normal	Normal	Normal				Normal	Normal	Normal
		Malanil	7	Normal		Normal	Normal	Normal				Normal	Normal	Normal
		Malanil	14	Normal		Normal	Normal	Normal				Normal	Normal	Normal

SUBJID	INITIAL	ARM	DAY	THYROID	THY DETAIL	LUNG	HEART	ABDOMEN	ABD DETAIL	LIVER (cm)	SPLEEN (cm)	LYMPH	EXTREMITIES	CNS
		Sevuparin/DF02 +Malanil	0	Normal		Normal	Normal	Normal				Normal	Normal	Normal
		Sevuparin/DF02 +Malanil	2	Normal		Normal	Normal	Normal				Normal	Normal	Normal
		Sevuparin/DF02 +Malanil	3	Normal		Normal	Normal	Normal				Normal	Normal	Normal
		Sevuparin/DF02 +Malanil	4	Normal		Normal	Normal	Normal				Normal	Normal	Normal
		Sevuparin/DF02 +Malanil	7	Normal		Normal	Normal	Normal				Normal	Normal	Normal
		Sevuparin/DF02 +Malanil	14	Normal		Normal	Normal	Normal				Normal	Normal	Normal
		Malanil	0	Normal		Normal	Normal	Normal				Normal	Normal	Normal
		Malanil	2	Normal		Normal	Normal	Normal				Normal	Normal	Normal
		Malanil	3	Normal		Normal	Normal	Normal				Normal	Normal	Normal
		Malanil	4	Normal		Normal	Normal	Normal				Normal	Normal	Normal
		Malanil	7	Normal		Normal	Normal	Normal				Normal	Normal	Normal
		Malanil	14	Normal		Normal	Normal	Normal				Normal	Normal	Normal
		Malanil	0	Normal		Normal	Normal	Normal				Normal	Normal	Normal
		Malanil	2	Normal		Normal	Normal	Normal				Normal	Normal	Normal
		Malanil	3	Normal		Normal	Normal	Normal				Normal	Normal	Normal
		Malanil	4	Normal		Normal	Normal	Normal				Normal	Normal	Normal
		Malanil	7	Normal		Normal	Normal	Normal				Normal	Normal	Normal
		Malanil	14	Normal		Normal	Normal	Normal				Normal	Normal	Normal
		Malanil	0	Normal		Normal	Normal	Normal				Normal	Normal	Normal

SUBJID	INITIAL	ARM	DAY	THYROID	THY DETAIL	LUNG	HEART	ABDOMEN	ABD DETAIL	LIVER (cm)	SPLEEN (cm)	LYMPH	EXTREMITIES	CNS
		Malanil	2	Normal		Normal	Normal	Normal				Normal	Normal	Normal
		Malanil	3	Normal		Normal	Normal	Normal				Normal	Normal	Normal
		Malanil	4	Normal		Normal	Normal	Normal				Normal	Normal	Normal
		Malanil	7	Normal		Normal	Normal	Normal				Normal	Normal	Normal
		Malanil	14	Normal		Normal	Normal	Normal				Normal	Normal	Normal
		Malanil	0	Normal		Normal	Normal	Normal				Normal	Normal	Normal
		Malanil	2	Normal		Normal	Normal	Normal				Normal	Normal	Normal
		Malanil	3	Normal		Normal	Normal	Normal				Normal	Normal	Normal
		Malanil	4	Normal		Normal	Normal	Normal				Normal	Normal	Normal
		Malanil	7	Normal		Normal	Normal	Normal				Normal	Normal	Normal
		Malanil	14	Normal		Normal	Normal	Normal				Normal	Normal	Normal
		Sevuparin/DF02 +Malanil	0	Normal		Normal	Normal	Normal				Normal	Normal	Normal
		Sevuparin/DF02 +Malanil	2	Normal		Normal	Normal	Normal				Normal	Normal	Normal
		Sevuparin/DF02 +Malanil	3	Normal		Normal	Normal	Normal				Normal	Normal	Normal
		Sevuparin/DF02 +Malanil	4	Normal		Normal	Normal	Normal				Normal	Normal	Normal
		Sevuparin/DF02 +Malanil	7	Normal		Normal	Normal	Normal				Normal	Normal	Normal
		Sevuparin/DF02 +Malanil	14	Normal		Normal	Normal	Normal				Normal	Normal	Normal
		Malanil	0	Normal		Normal	Normal	Normal				Normal	Normal	Normal
		Malanil	2	Normal		Normal	Normal	Normal				Normal	Normal	Normal

SUBJID	INITIAL	ARM	DAY	THYROID	THY DETAIL	LUNG	HEART	ABDOMEN	ABD DETAIL	LIVER (cm)	SPLEEN (cm)	LYMPH	EXTREMITIES	CNS
		Malanil	3	Normal		Normal	Normal	Normal				Normal	Normal	Normal
		Malanil	4	Normal		Normal	Normal	Normal				Normal	Normal	Normal
		Malanil	7	Normal		Normal	Normal	Normal				Normal	Normal	Normal
		Malanil	14	Normal		Normal	Normal	Normal				Normal	Normal	Normal
		Malanil	0	Normal		Normal	Normal	Normal				Normal	Normal	Normal
		Malanil	2	Normal		Normal	Normal	Normal				Normal	Normal	Normal
		Malanil	3	Normal		Normal	Normal	Normal				Normal	Normal	Normal
		Malanil	4	Normal		Normal	Normal	Normal				Normal	Normal	Normal
		Malanil	7	Normal		Normal	Normal	Normal				Normal	Normal	Normal
		Malanil	14	Normal		Normal	Normal	Normal				Normal	Normal	Normal
		Malanil	0	Normal		Normal	Normal	Normal				Normal	Normal	Normal
		Malanil	2	Normal		Normal	Normal	Normal				Normal	Normal	Normal
		Malanil	3	Normal		Normal	Normal	Normal				Normal	Normal	Normal
		Malanil	4	Normal		Normal	Normal	Normal				Normal	Normal	Normal
		Malanil	7	Normal		Normal	Normal	Normal				Normal	Normal	Normal
		Malanil	14	Normal		Normal	Normal	Normal				Normal	Normal	Normal
		Sevuparin/DF02 +Malanil	0	Normal		Normal	Normal	Normal				Normal	Normal	Normal
		Sevuparin/DF02 +Malanil	2	Normal		Normal	Normal	Normal				Normal	Normal	Normal
		Sevuparin/DF02 +Malanil	3	Normal		Normal	Normal	Normal				Normal	Normal	Normal
		Sevuparin/DF02 +Malanil	4	Normal		Normal	Normal	Normal				Normal	Normal	Normal

SUBJID	INITIAL	ARM	DAY	THYROID	THY DETAIL	LUNG	HEART	ABDOMEN	ABD DETAIL	LIVER (cm)	SPLEEN (cm)	LYMPH	EXTREMITIES	CNS
		Sevuparin/DF02 +Malanil	7	Normal		Normal	Normal	Normal				Normal	Normal	Normal
		Sevuparin/DF02 +Malanil	14	Normal		Normal	Normal	Normal				Normal	Normal	Normal
		Sevuparin/DF02 +Malanil	0	Normal		Normal	Normal	Normal				Normal	Normal	Normal
		Sevuparin/DF02 +Malanil	2	Normal		Normal	Normal	Normal				Normal	Normal	Normal
		Sevuparin/DF02 +Malanil	3	Normal		Normal	Normal	Normal				Normal	Normal	Normal
		Sevuparin/DF02 +Malanil	4	Normal		Normal	Normal	Normal				Normal	Normal	Normal
		Sevuparin/DF02 +Malanil	7	Normal		Normal	Normal	Normal				Normal	Normal	Normal
		Sevuparin/DF02 +Malanil	14	Normal		Normal	Normal	Normal				Normal	Normal	Normal
		Sevuparin/DF02 +Malanil	0	Normal		Normal	Normal	Normal				Normal	Normal	Normal
		Sevuparin/DF02 +Malanil	1	Normal		Normal	Normal	Normal				Normal	Normal	Normal
		Sevuparin/DF02 +Malanil	2	Normal		Normal	Normal	Normal				Normal	Normal	Normal
		Sevuparin/DF02 +Malanil	3	Normal		Normal	Normal	Normal				Normal	Normal	Normal
		Sevuparin/DF02 +Malanil	4	Normal		Normal	Normal	Normal				Normal	Normal	Normal
		Sevuparin/DF02 +Malanil	7	Normal		Normal	Normal	Normal				Normal	Normal	Normal

SUBJID	INITIAL	ARM	DAY	THYROID	THY DETAIL	LUNG	HEART	ABDOMEN	ABD DETAIL	LIVER (cm)	SPLEEN (cm)	LYMPH	EXTREMITIES	CNS
		Sevuparin/DF02 +Malanil	14	Normal		Normal	Normal	Normal				Normal	Normal	Normal
		Sevuparin/DF02 +Malanil	0	Normal		Normal	Normal	Normal				Normal	Normal	Normal
		Sevuparin/DF02 +Malanil	2	Normal		Normal	Normal	Normal				Normal	Normal	Normal
		Sevuparin/DF02 +Malanil	3	Normal		Normal	Normal	Normal				Normal	Normal	Normal
		Sevuparin/DF02 +Malanil	4	Normal		Normal	Normal	Normal				Normal	Normal	Normal
		Sevuparin/DF02 +Malanil	7	Normal		Normal	Normal	Normal				Normal	Normal	Normal
		Sevuparin/DF02 +Malanil	14	Normal		Normal	Normal	Normal				Normal	Normal	Normal
		Sevuparin/DF02 +Malanil	0	Normal		Normal	Normal	Normal				Normal	Normal	Normal
		Sevuparin/DF02 +Malanil	2	Normal		Normal	Normal	Normal				Normal	Normal	Normal
		Sevuparin/DF02 +Malanil	3	Normal		Normal	Normal	Normal				Normal	Normal	Normal
		Sevuparin/DF02 +Malanil	4	Normal		Normal	Normal	Normal				Normal	Normal	Normal
		Sevuparin/DF02 +Malanil	7	Normal		Normal	Normal	Normal				Normal	Normal	Normal
		Sevuparin/DF02 +Malanil	14	Normal		Normal	Normal	Normal				Normal	Normal	Normal
		Malanil	0	Normal		Normal	Normal	Normal				Normal	Normal	Normal

SUBJID	INITIAL	ARM	DAY	THYROID	THY DETAIL	LUNG	HEART	ABDOMEN	ABD DETAIL	LIVER (cm)	SPLEEN (cm)	LYMPH	EXTREMITIES	CNS
		Malanil	2	Normal		Normal	Normal	Normal				Normal	Normal	Normal
		Malanil	3	Normal		Normal	Normal	Normal				Normal	Normal	Normal
		Malanil	4	Normal		Normal	Normal	Normal				Normal	Normal	Normal
		Malanil	7	Normal		Normal	Normal	Normal				Normal	Normal	Normal
		Malanil	14	Normal		Normal	Normal	Normal				Normal	Normal	Normal
		Malanil	0	Normal		Normal	Normal	Normal				Normal	Normal	Normal
		Malanil	2	Normal		Normal	Normal	Normal				Normal	Normal	Normal
		Malanil	3	Normal		Normal	Normal	Normal				Normal	Normal	Normal
		Malanil	4	Normal		Normal	Normal	Normal				Normal	Normal	Normal
		Malanil	7	Normal		Normal	Normal	Normal				Normal	Normal	Normal
		Malanil	14	Normal		Normal	Normal	Normal				Normal	Normal	Normal
		Malanil	0	Normal		Normal	Normal	Normal				Normal	Normal	Normal
		Malanil	2	Normal		Normal	Normal	Normal				Normal	Normal	Normal
		Malanil	3	Normal		Normal	Normal	Normal				Normal	Normal	Normal
		Malanil	4	Normal		Normal	Normal	Normal				Normal	Normal	Normal
		Malanil	5	Normal		Normal	Normal	Normal				Normal	Normal	Normal
		Malanil	7	Normal		Normal	Normal	Normal				Normal	Normal	Normal
		Malanil	14	Normal		Normal	Normal	Normal				Normal	Normal	Normal

16.2.7.4 Vital signs

SUBJID	INITIAL	ARM	TIMEPOINT	TEMP (˚C)	HR (bpm)	RR (bpm)	BP (mmHg)
		Malanil	screening	37	92	20	127/076
		Malanil	D1H0	37.2	80	20	108/062
		Malanil	D1H6	37.5	78	20	111/065
		Malanil	D1H12	36.6	72	20	107/060
		Malanil	D1H18	36.8	78	20	115/064
		Malanil	D2H24	36.4	80	20	108/058
		Malanil	D2H30	37.3	70	20	097/056
		Malanil	D2H36	36.4	74	20	105/059
		Malanil	D2H42	36.2	62	20	106/061
		Malanil	D3H48	36.2	62	20	109/053
		Malanil	D3H54	36.9	70	20	122/071
		Malanil	D3H60	36.4	66	20	104/065
		Malanil	D3H66	36.2	76	20	110/066
		Malanil	D4H72	36.1	70	20	107/062
		Sevuparin/DF02+Malanil	screening	36.8	68	20	125/073
		Sevuparin/DF02+Malanil	D1H0	37.2	70	20	104/053
		Sevuparin/DF02+Malanil	D1H6	37.2	70	20	117/063
		Sevuparin/DF02+Malanil	D1H12	37.2	62	20	107/065
		Sevuparin/DF02+Malanil	D1H18	35.8	62	20	102/057
		Sevuparin/DF02+Malanil	D2H24	36.5	64	20	111/059
		Sevuparin/DF02+Malanil	D2H30	36.5	64	20	106/055
		Sevuparin/DF02+Malanil	D2H36	35.9	58	20	106/062
		Sevuparin/DF02+Malanil	D2H42	36.4	62	20	103/058
		Sevuparin/DF02+Malanil	D3H48	36.9	64	20	106/055
		Sevuparin/DF02+Malanil	D3H54	36.8	62	20	117/063
		Sevuparin/DF02+Malanil	D3H60	36	58	20	099/054
		Sevuparin/DF02+Malanil	D3H66	36.8	66	20	105/062
		Sevuparin/DF02+Malanil	D4H72	36.8	66	20	105/062
		Sevuparin/DF02+Malanil	D7	37.3	92	20	128/078
		Sevuparin/DF02+Malanil	D14	36.9	84	20	119/065
		Sevuparin/DF02+Malanil	screening	38.8	104	20	118/067
		Sevuparin/DF02+Malanil	D1H0	37.6	76	20	110/066
		Sevuparin/DF02+Malanil	D1H6	37.6	72	20	125/080
		Sevuparin/DF02+Malanil	D1H12	35.9	64	20	107/061
		Sevuparin/DF02+Malanil	D1H18	37.5	70	20	101/062

SUBJID	INITIAL	ARM	TIMEPOINT	TEMP (˚C)	HR (bpm)	RR (bpm)	BP (mmHg)
		Sevuparin/DF02+Malanil	D2H24	38.1	78	20	105/047
		Sevuparin/DF02+Malanil	D2H30	38.8	86	20	108/059
		Sevuparin/DF02+Malanil	D2H36	38.8	80	20	100/060
		Sevuparin/DF02+Malanil	D2H42	37.5	76	20	091/053
		Sevuparin/DF02+Malanil	D3H48	37.4	70	20	107/063
		Sevuparin/DF02+Malanil	D3H54	36.7	72	20	116/078
		Sevuparin/DF02+Malanil	D3H60	35.2	60	20	110/076
		Sevuparin/DF02+Malanil	D3H66	36	60	20	100/050
		Sevuparin/DF02+Malanil	D4H72	36.4	70	20	117/076
		Sevuparin/DF02+Malanil	D5	35.9	68	20	117/067
		Sevuparin/DF02+Malanil	D7	37	80	20	120/068
		Sevuparin/DF02+Malanil	D14	37	82	20	123/065
		Malanil	screening	36.8	72	20	106/061
		Malanil	D1H0	36.9	78	20	100/053
		Malanil	D1H6	37.6	64	20	117/058
		Malanil	D1H12	38.3	72	20	130/055
		Malanil	D1H18	36.3	60	20	099/052
		Malanil	D2H24	36.4	58	20	115/058
		Malanil	D2H30	36.8	68	20	112/057
		Malanil	D2H36	36	56	20	102/057
		Malanil	D2H42	35.4	58	20	106/078
		Malanil	D3H48	36.4	90	20	100/060
		Malanil	D3H54	36.5	58	20	121/056
		Malanil	D3H60	36.7	56	20	117/061
		Malanil	D3H66	36.6	64	20	109/056
		Malanil	D4H72	36.6	64	20	109/056
		Malanil	D7	36.6	70	20	115/066
		Malanil	D14	35.5	62	20	108/052
		Malanil	screening	37	106	20	126/066
		Malanil	D1H0	37	90	20	102/054
		Malanil	D1H6	38.2	92	22	118/061
		Malanil	D1H12	36.9	70	20	106/059
		Malanil	D1H18	36.9	72	20	108/059
		Malanil	D2H24	37.2	90	20	117/065
		Malanil	D2H30	37.1	84	20	122/063
		Malanil	D2H36	37	70	20	095/055
		Malanil	D2H42	36	68	20	102/054

SUBJID	INITIAL	ARM	TIMEPOINT	TEMP (˚C)	HR (bpm)	RR (bpm)	BP (mmHg)
		Malanil	D3H48	36	70	20	116/066
		Malanil	D3H54	36	80	20	119/085
		Malanil	D3H60	36.6	72	20	099/054
		Malanil	D3H66	36.3	80	20	121/065
		Malanil	D4H72	36.3	80	20	121/065
		Malanil	D7	36.2	96	20	119/072
		Malanil	D14	36.9	100	20	112/066
		Sevuparin/DF02+Malanil	screening	37.7	90	20	131/054
		Sevuparin/DF02+Malanil	D1H0	37.3	82	20	119/061
		Sevuparin/DF02+Malanil	D1H6	36.9	78	20	120/062
		Sevuparin/DF02+Malanil	D1H12	36.6	70	20	127/066
		Sevuparin/DF02+Malanil	D1H18	36.6	64	20	122/064
		Sevuparin/DF02+Malanil	D2H24	36.7	62	20	103/055
		Sevuparin/DF02+Malanil	D2H30	36.9	60	20	130/055
		Sevuparin/DF02+Malanil	D2H36	36.9	60	20	122/058
		Sevuparin/DF02+Malanil	D2H42	36.1	56	20	119/053
		Sevuparin/DF02+Malanil	D3H48	36.7	58	20	133/053
		Sevuparin/DF02+Malanil	D3H54	36.7	60	20	122/057
		Sevuparin/DF02+Malanil	D3H60	35.3	62	20	109/063
		Sevuparin/DF02+Malanil	D3H66	36	60	20	111/065
		Sevuparin/DF02+Malanil	D4H72	36.2	64	20	108/064
		Sevuparin/DF02+Malanil	D7	35.6	72	20	124/066
		Sevuparin/DF02+Malanil	D14	35.3	70	20	126/060
		Malanil	screening	36.9	108	20	105/063
		Malanil	D1H0	37.3	98	20	097/066
		Malanil	D1H6	38.9	98	20	100/062
		Malanil	D1H12	36.7	88	20	099/063
		Malanil	D1H18	35.5	72	20	104/063
		Malanil	D2H24	36	72	20	100/064
		Malanil	D2H30	35.5	72	20	097/061
		Malanil	D2H36	35.2	70	20	098/058
		Malanil	D2H42	35.9	74	20	110/073
		Malanil	D3H48	37.5	74	20	100/061
		Malanil	D3H54	36.5	72	20	103/061
		Malanil	D3H60	36.3	66	20	108/056
		Malanil	D4H72	35.7	66	20	102/064
		Malanil	D7	36.2	90	20	115/065

SUBJID	INITIAL	ARM	TIMEPOINT	TEMP (˚C)	HR (bpm)	RR (bpm)	BP (mmHg)
		Sevuparin/DF02+Malanil	screening	36.3	82	20	117/065
		Sevuparin/DF02+Malanil	D1H0	37	94	20	121/071
		Sevuparin/DF02+Malanil	D1H6	37.7	86	20	133/063
		Sevuparin/DF02+Malanil	D1H12	38.7	90	20	129/066
		Sevuparin/DF02+Malanil	D1H18	36.8	62	20	115/084
		Sevuparin/DF02+Malanil	D2H24	37.9	82	20	119/066
		Sevuparin/DF02+Malanil	D2H30	39.1	92	20	134/072
		Sevuparin/DF02+Malanil	D2H36	36.9	72	20	108/072
		Sevuparin/DF02+Malanil	D2H42	36.9	78	20	124/066
		Sevuparin/DF02+Malanil	D3H48	37	80	20	108/074
		Sevuparin/DF02+Malanil	D3H54	37.6	70	20	117/073
		Sevuparin/DF02+Malanil	D3H60	37.2	68	20	128/070
		Sevuparin/DF02+Malanil	D4H72	36.5	64	20	122/075
		Sevuparin/DF02+Malanil	D7	37.4	86	20	128/067
		Sevuparin/DF02+Malanil	D14	35.3	92	20	105/076
		Sevuparin/DF02+Malanil	screening	36.3	94	20	093/053
		Sevuparin/DF02+Malanil	D1H0	36.2	84	20	116/061
		Sevuparin/DF02+Malanil	D1H6	38	100	20	113/050
		Sevuparin/DF02+Malanil	D1H12	35.8	76	20	099/052
		Sevuparin/DF02+Malanil	D1H18	36.6	96	20	109/050
		Sevuparin/DF02+Malanil	D2H24	37	82	20	123/064
		Sevuparin/DF02+Malanil	D2H30	37.6	84	20	115/058
		Sevuparin/DF02+Malanil	D2H36	38.7	84	20	113/055
		Sevuparin/DF02+Malanil	D2H42	37.6	62	20	133/053
		Sevuparin/DF02+Malanil	D3H48	36.9	76	20	105/069
		Sevuparin/DF02+Malanil	D3H54	38.2	86	20	117/061
		Sevuparin/DF02+Malanil	D3H60	35.2	62	20	109/055
		Sevuparin/DF02+Malanil	D3H66	35.7	72	20	120/060
		Sevuparin/DF02+Malanil	D4H72	35.7	72	20	120/060
		Sevuparin/DF02+Malanil	D7	36.5	84	20	105/055
		Sevuparin/DF02+Malanil	D14	36.6	82	20	094/052
		Malanil	screening	35.8	88	20	115/067
		Malanil	D1H0	36.1	82	20	104/058
		Malanil	D1H6	37.7	104	20	110/064
		Malanil	D1H12	36.7	82	20	124/087
		Malanil	D1H18	35.6	74	20	110/065
		Malanil	D2H24	36.9	76	20	105/068

SUBJID	INITIAL	ARM	TIMEPOINT	TEMP (˚C)	HR (bpm)	RR (bpm)	BP (mmHg)
		Malanil	D2H30	35.6	74	20	108/060
		Malanil	D2H36	36	68	20	106/058
		Malanil	D2H42	36.1	76	20	110/063
		Malanil	D3H48	36	76	20	106/066
		Malanil	D3H54	35.1	66	20	106/064
		Malanil	D3H60	36.1	62	20	108/073
		Malanil	D3H66	36.7	70	20	117/065
		Malanil	D4H72	36.7	70	20	117/065
		Malanil	D7	35.6	70	20	118/061
		Malanil	D14	36	80	20	110/076
		Malanil	screening	37.5	102	20	126/064
		Malanil	D1H0	37.3	92	20	120/067
		Malanil	D1H6	36.4	90	20	118/075
		Malanil	D1H12	36.2	74	20	110/060
		Malanil	D1H18	35.3	74	20	112/063
		Malanil	D2H24	36	72	20	110/062
		Malanil	D2H30	36	72	20	111/062
		Malanil	D2H36	36	84	20	113/064
		Malanil	D2H42	36	84	20	119/067
		Malanil	D3H48	35.7	74	20	102/062
		Malanil	D3H54	35.5	84	20	093/061
		Malanil	D3H60	35.2	68	20	109/060
		Malanil	D4H72	35.4	82	20	112/063
		Malanil	D7	37	118	20	126/068
		Malanil	D14	36.8	100	20	123/087
		Sevuparin/DF02+Malanil	screening	36	76	20	111/071
		Sevuparin/DF02+Malanil	D1H0	36.9	82	20	110/070
		Sevuparin/DF02+Malanil	D1H6	38.6	90	20	116/075
		Sevuparin/DF02+Malanil	D1H12	36.2	66	20	104/068
		Sevuparin/DF02+Malanil	D1H18	37.3	70	20	111/068
		Sevuparin/DF02+Malanil	D2H24	36	78	20	107/064
		Sevuparin/DF02+Malanil	D2H30	36.1	72	20	120/074
		Sevuparin/DF02+Malanil	D2H36	36.5	64	20	112/068
		Sevuparin/DF02+Malanil	D2H42	36.1	60	20	108/066
		Sevuparin/DF02+Malanil	D3H48	36.5	66	20	123/072
		Sevuparin/DF02+Malanil	D3H54	37	68	20	116/072
		Sevuparin/DF02+Malanil	D3H60	36.6	62	20	122/075

SUBJID	INITIAL	ARM	TIMEPOINT	TEMP (˚C)	HR (bpm)	RR (bpm)	BP (mmHg)
		Sevuparin/DF02+Malanil	D4H72	36	74	20	114/075
		Sevuparin/DF02+Malanil	D7	36.7	82	20	102/066
		Sevuparin/DF02+Malanil	screening	37.7	86	20	130/068
		Sevuparin/DF02+Malanil	D1H0	37.4	76	20	125/062
		Sevuparin/DF02+Malanil	D1H6	39	78	20	098/054
		Sevuparin/DF02+Malanil	D1H12	39.4	76	20	108/070
		Sevuparin/DF02+Malanil	D1H18	37.5	78	20	100/070
		Sevuparin/DF02+Malanil	D2H24	38	70	20	119/059
		Sevuparin/DF02+Malanil	D2H30	36.9	76	20	103/056
		Sevuparin/DF02+Malanil	D2H36	37.5	66	20	100/056
		Sevuparin/DF02+Malanil	D2H42	37.4	72	20	100/060
		Sevuparin/DF02+Malanil	D3H48	36.9	74	20	114/058
		Sevuparin/DF02+Malanil	D3H54	37.3	80	20	118/063
		Sevuparin/DF02+Malanil	D3H60	36.8	68	20	098/068
		Sevuparin/DF02+Malanil	D4H72	36.7	62	20	098/047
		Sevuparin/DF02+Malanil	D7	37.5	74	20	132/067
		Sevuparin/DF02+Malanil	D14	36.8	80	20	116/066
		Malanil	screening	36.5	104	20	120/071
		Malanil	D1H0	36.8	94	20	125/077
		Malanil	D1H6	37	82	20	110/068
		Malanil	D1H12	37.3	78	20	116/070
		Malanil	D1H18	38.9	84	20	115/068
		Malanil	D2H24	38.6	92	20	119/063
		Malanil	D2H30	37.6	86	20	123/075
		Malanil	D2H36	38.3	84	20	111/068
		Malanil	D2H42	37.2	76	20	117/072
		Malanil	D3H48	36.8	78	20	110/070
		Malanil	D3H54	36.9	70	20	110/070
		Malanil	D3H60	36.9	62	20	106/076
		Malanil	D3H66	36.3	62	20	116/074
		Malanil	D4H72	36.3	62	20	116/074
		Malanil	D7	36.5	100	20	121/086
		Malanil	D14	36.4	88	20	120/080
		Malanil	screening	39.9	128	20	115/066
		Malanil	D1H0	39.1	116	20	096/057
		Malanil	D1H6	38.9	100	20	100/060
		Malanil	D1H12	36.4	84	20	104/058

SUBJID	INITIAL	ARM	TIMEPOINT	TEMP (˚C)	HR (bpm)	RR (bpm)	BP (mmHg)
		Malanil	D1H18	36.8	94	20	104/058
		Malanil	D2H24	37.8	92	20	110/060
		Malanil	D2H30	37.4	86	20	109/053
		Malanil	D2H36	38.8	78	20	100/049
		Malanil	D2H42	38.9	94	20	110/060
		Malanil	D3H48	39.5	98	22	115/055
		Malanil	D3H54	39.8	100	20	120/070
		Malanil	D3H60	36	80	20	099/053
		Malanil	D3H66	37.4	88	20	100/060
		Malanil	D4H72	37.4	88	20	100/060
		Malanil	D7	36.7	70	20	115/060
		Malanil	D14	36.2	72	20	120/060
		Malanil	screening	37.9	64	20	098/062
		Malanil	D1H0	37.3	82	20	102/063
		Malanil	D1H6	37.1	62	20	105/065
		Malanil	D1H12	36.7	64	20	100/055
		Malanil	D1H18	36.5	62	20	110/070
		Malanil	D2H24	36.3	60	20	108/067
		Malanil	D2H30	36.4	60	20	108/066
		Malanil	D2H36	37.2	60	20	107/068
		Malanil	D2H42	36	62	20	118/072
		Malanil	D3H48	37.2	64	20	112/064
		Malanil	D3H54	36.2	60	20	111/069
		Malanil	D3H60	36.1	56	20	121/067
		Malanil	D3H66	36	56	20	117/068
		Malanil	D4H72	36	56	20	117/068
		Malanil	D7	36.4	80	20	118/068
		Malanil	D14	36	78	20	104/070
		Sevuparin/DF02+Malanil	screening	36.9	90	20	116/068
		Sevuparin/DF02+Malanil	D1H0	37.7	78	20	090/062
		Sevuparin/DF02+Malanil	D1H6	38.8	82	20	095/052
		Sevuparin/DF02+Malanil	D1H12	37.5	72	20	100/052
		Sevuparin/DF02+Malanil	D1H18	36.4	74	20	098/049
		Sevuparin/DF02+Malanil	D2H24	36.7	74	20	109/074
		Sevuparin/DF02+Malanil	D2H30	37.4	70	20	103/053
		Sevuparin/DF02+Malanil	D2H36	36.7	68	20	095/056
		Sevuparin/DF02+Malanil	D2H42	36.5	64	20	093/058

SUBJID	INITIAL	ARM	TIMEPOINT	TEMP (˚C)	HR (bpm)	RR (bpm)	BP (mmHg)
		Sevuparin/DF02+Malanil	D3H48	36.8	70	20	100/060
		Sevuparin/DF02+Malanil	D3H54	36.8	78	20	103/067
		Sevuparin/DF02+Malanil	D3H60	36.6	62	20	096/054
		Sevuparin/DF02+Malanil	D3H66	36.7	72	20	104/060
		Sevuparin/DF02+Malanil	D4H72	36.7	72	20	104/060
		Sevuparin/DF02+Malanil	D7	35.1	86	20	105/064
		Sevuparin/DF02+Malanil	D14	36	70	20	109/068
		Sevuparin/DF02+Malanil	screening	38.5	112	20	112/074
		Sevuparin/DF02+Malanil	D1H0	38.6	96	20	108/068
		Sevuparin/DF02+Malanil	D1H6	38.8	88	20	104/064
		Sevuparin/DF02+Malanil	D1H12	36.5	78	20	098/064
		Sevuparin/DF02+Malanil	D1H18	36.3	80	20	118/077
		Sevuparin/DF02+Malanil	D2H24	36.6	84	20	117/063
		Sevuparin/DF02+Malanil	D2H30	38.2	76	20	123/085
		Sevuparin/DF02+Malanil	D2H36	38	86	20	095/053
		Sevuparin/DF02+Malanil	D2H42	37.6	76	18	126/074
		Sevuparin/DF02+Malanil	D3H48	37.9	64	20	127/077
		Sevuparin/DF02+Malanil	D3H54	37.6	68	20	100/057
		Sevuparin/DF02+Malanil	D3H60	36.7	64	20	105/065
		Sevuparin/DF02+Malanil	D3H66	36.3	68	20	111/063
		Sevuparin/DF02+Malanil	D4H72	36.3	68	20	111/063
		Sevuparin/DF02+Malanil	D7	36.9	86	20	126/082
		Sevuparin/DF02+Malanil	D14	36.7	80	20	130/080
		Sevuparin/DF02+Malanil	screening	37.3	104	20	118/052
		Sevuparin/DF02+Malanil	D1H0	39.1	110	20	090/050
		Sevuparin/DF02+Malanil	D1H6	37.1	108	20	109/053
		Sevuparin/DF02+Malanil	D1H12	37.2	100	20	096/060
		Sevuparin/DF02+Malanil	D1H18	37.5	92	20	105/098
		Sevuparin/DF02+Malanil	D2H24	38.2	98	20	115/060
		Sevuparin/DF02+Malanil	D2H30	38.2	102	22	093/057
		Sevuparin/DF02+Malanil	D2H36	36.8	94	20	098/051
		Sevuparin/DF02+Malanil	D2H42	37.8	98	20	107/059
		Sevuparin/DF02+Malanil	D3H48	37.3	98	20	102/059
		Sevuparin/DF02+Malanil	D3H54	38.2	96	20	093/054
		Sevuparin/DF02+Malanil	D3H60	36.8	84	20	095/054
		Sevuparin/DF02+Malanil	D3H66	36	80	20	101/047
		Sevuparin/DF02+Malanil	D4H72	36.4	82	20	100/062

SUBJID	INITIAL	ARM	TIMEPOINT	TEMP (˚C)	HR (bpm)	RR (bpm)	BP (mmHg)
		Sevuparin/DF02+Malanil	D7	36	94	20	113/066
		Sevuparin/DF02+Malanil	D14	36.3	96	20	100/060
		Malanil	screening	36.8	74	20	117/062
		Malanil	D1H0	36.6	76	20	110/070
		Malanil	D1H6	37	76	20	109/076
		Malanil	D1H12	36	62	20	104/052
		Malanil	D1H18	36.7	60	20	115/051
		Malanil	D2H24	36.8	60	20	125/087
		Malanil	D2H30	36.7	62	20	099/051
		Malanil	D2H36	36.1	56	20	101/056
		Malanil	D2H42	36.3	56	20	103/058
		Malanil	D3H48	36.6	56	20	113/064
		Malanil	D3H54	37	52	20	121/063
		Malanil	D3H60	36.5	80	20	111/055
		Malanil	D3H66	36	60	20	117/059
		Malanil	D4H72	36	60	20	117/059
		Malanil	D7	36.5	60	20	108/052
		Malanil	D14	36.7	62	20	098/055
		Sevuparin/DF02+Malanil	screening	37.6	96	20	118/061
		Sevuparin/DF02+Malanil	D1H0	39.7	98	20	094/051
		Sevuparin/DF02+Malanil	D1H6	37.7	90	20	123/070
		Sevuparin/DF02+Malanil	D1H12	38.6	90	20	116/060
		Sevuparin/DF02+Malanil	D1H18	36.5	70	20	113/062
		Sevuparin/DF02+Malanil	D2H24	37.6	78	20	113/057
		Sevuparin/DF02+Malanil	D2H30	37.5	92	20	103/054
		Sevuparin/DF02+Malanil	D2H36	36.7	78	20	100/060
		Sevuparin/DF02+Malanil	D2H42	37.2	78	20	120/070
		Sevuparin/DF02+Malanil	D3H48	38.4	80	20	115/062
		Sevuparin/DF02+Malanil	D3H54	37.4	82	20	116/070
		Sevuparin/DF02+Malanil	D3H60	37.2	66	20	125/078
		Sevuparin/DF02+Malanil	D3H66	36.8	64	20	119/063
		Sevuparin/DF02+Malanil	D4H72	36.8	64	20	119/063
		Sevuparin/DF02+Malanil	D7	36.3	82	20	131/068
		Sevuparin/DF02+Malanil	D14	36.8	74	20	121/065
		Sevuparin/DF02+Malanil	screening	38.1	118	20	114/069
		Sevuparin/DF02+Malanil	D1H0	36.8	94	20	099/058
		Sevuparin/DF02+Malanil	D1H6	38.9	102	20	099/060

SUBJID	INITIAL	ARM	TIMEPOINT	TEMP (˚C)	HR (bpm)	RR (bpm)	BP (mmHg)
		Sevuparin/DF02+Malanil	D1H12	38	88	22	113/063
		Sevuparin/DF02+Malanil	D1H18	37.3	78	20	108/059
		Sevuparin/DF02+Malanil	D2H24	38.6	92	20	117/063
		Sevuparin/DF02+Malanil	D2H30	38	82	20	113/067
		Sevuparin/DF02+Malanil	D2H36	36.3	76	20	109/061
		Sevuparin/DF02+Malanil	D2H42	36.5	72	20	126/061
		Sevuparin/DF02+Malanil	D3H48	36.6	74	20	116/062
		Sevuparin/DF02+Malanil	D3H54	36.6	74	20	116/070
		Sevuparin/DF02+Malanil	D3H60	36.2	60	20	106/057
		Sevuparin/DF02+Malanil	D3H66	36.3	58	20	108/060
		Sevuparin/DF02+Malanil	D4H72	36.3	58	20	108/060
		Sevuparin/DF02+Malanil	D7	36.5	110	20	124/065
		Sevuparin/DF02+Malanil	D14	36.7	100	20	108/062
		Sevuparin/DF02+Malanil	screening	37.2	128	20	113/079
		Sevuparin/DF02+Malanil	D1H0	37.9	128	20	110/071
		Sevuparin/DF02+Malanil	D1H6	36.5	98	20	103/073
		Sevuparin/DF02+Malanil	D1H12	36.3	94	20	096/058
		Sevuparin/DF02+Malanil	D1H18	36.8	94	20	106/074
		Sevuparin/DF02+Malanil	D2H24	36.5	86	20	100/060
		Sevuparin/DF02+Malanil	D2H30	38.6	118	22	093/053
		Sevuparin/DF02+Malanil	D2H36	36.8	88	20	096/068
		Sevuparin/DF02+Malanil	D2H42	36.6	88	20	093/061
		Sevuparin/DF02+Malanil	D3H48	36.7	92	20	090/066
		Sevuparin/DF02+Malanil	D3H54	37.1	98	20	091/060
		Sevuparin/DF02+Malanil	D3H60	36.2	70	20	105/063
		Sevuparin/DF02+Malanil	D3H66	36	78	20	098/062
		Sevuparin/DF02+Malanil	D4H72	36	78	20	098/062
		Sevuparin/DF02+Malanil	D7	37.6	100	20	119/079
		Sevuparin/DF02+Malanil	D14	36.7	94	20	123/079
		Sevuparin/DF02+Malanil	screening	37.3	110	20	109/055
		Sevuparin/DF02+Malanil	D1H0	38.1	98	20	110/065
		Sevuparin/DF02+Malanil	D1H6	38	100	20	093/065
		Sevuparin/DF02+Malanil	D1H12	38.2	86	20	096/056
		Sevuparin/DF02+Malanil	D1H18	37	78	20	120/060
		Sevuparin/DF02+Malanil	D2H24	39.6	98	20	116/066
		Sevuparin/DF02+Malanil	D2H30	40.3	98	20	108/052
		Sevuparin/DF02+Malanil	D2H36	38.2	86	20	097/057

SUBJID	INITIAL	ARM	TIMEPOINT	TEMP (˚C)	HR (bpm)	RR (bpm)	BP (mmHg)
		Sevuparin/DF02+Malanil	D2H42	36.8	74	20	107/056
		Sevuparin/DF02+Malanil	D3H48	37.1	76	20	105/067
		Sevuparin/DF02+Malanil	D3H54	37.5	76	20	090/053
		Sevuparin/DF02+Malanil	D3H60	37.6	68	20	094/056
		Sevuparin/DF02+Malanil	D3H66	36.7	66	20	097/057
		Sevuparin/DF02+Malanil	D4H72	36.7	66	20	097/057
		Sevuparin/DF02+Malanil	D7	36.9	86	20	109/057
		Sevuparin/DF02+Malanil	D14	36.5	76	20	110/068
		Malanil	screening	37.2	80	20	114/076
		Malanil	D1H0	36.6	74	20	103/067
		Malanil	D1H6	37.3	88	20	105/075
		Malanil	D1H12	37.4	68	20	108/078
		Malanil	D1H18	36.7	82	20	116/078
		Malanil	D2H24	38.4	80	20	109/073
		Malanil	D2H30	37.7	88	20	113/063
		Malanil	D2H36	37.6	88	20	115/080
		Malanil	D2H42	38.2	76	24	108/072
		Malanil	D3H48	36.7	88	20	100/050
		Malanil	D3H54	37.3	88	20	105/075
		Malanil	D3H60	36.4	82	20	102/068
		Malanil	D3H66	36.5	88	20	108/062
		Malanil	D7	36.6	86	24	112/072
		Malanil	D14	36.1	76	20	129/080
		Malanil	screening	36.4	80	20	112/071
		Malanil	D1H0	37.4	82	20	108/067
		Malanil	D1H6	37.2	98	20	106/061
		Malanil	D1H12	38.1	90	20	090/053
		Malanil	D1H18	38.3	90	20	109/058
		Malanil	D2H24	38.3	92	20	095/055
		Malanil	D2H30	37	80	20	113/078
		Malanil	D2H36	38.8	80	24	102/067
		Malanil	D2H42	36.4	70	20	100/060
		Malanil	D3H48	36.7	88	20	099/059
		Malanil	D3H54	36.6	76	20	100/063
		Malanil	D3H60	36.5	74	20	102/058
		Malanil	D3H66	36.3	64	20	103/058
		Malanil	D7	36.8	78	20	106/064

SUBJID	INITIAL	ARM	TIMEPOINT	TEMP (˚C)	HR (bpm)	RR (bpm)	BP (mmHg)
		Malanil	D14	36.7	80	20	112/064
		Malanil	screening	38.5	82	20	118/058
		Malanil	D1H0	37.4	62	20	104/051
		Malanil	D1H6	37.3	68	20	097/050
		Malanil	D1H12	37.1	66	20	088/046
		Malanil	D1H18	36.7	74	20	103/051
		Malanil	D2H24	37	66	20	113/063
		Malanil	D2H30	36.8	74	20	105/074
		Malanil	D2H36	36.6	54	20	097/056
		Malanil	D2H42	36.4	64	20	090/050
		Malanil	D3H48	36.6	76	20	101/068
		Malanil	D3H54	36.9	70	20	109/064
		Malanil	D3H60	36.5	78	20	105/062
		Malanil	D3H66	37.8	68	20	096/057
		Malanil	D7	37.2	76	20	110/067
		Malanil	D14	36.8	90	20	128/074
		Sevuparin/DF02+Malanil	screening	36.7	84	20	107/066
		Sevuparin/DF02+Malanil	D1H0	36.4	66	20	092/051
		Sevuparin/DF02+Malanil	D1H6	37.2	76	20	096/047
		Sevuparin/DF02+Malanil	D1H12	36.6	74	20	099/067
		Sevuparin/DF02+Malanil	D1H18	36.5	70	20	099/058
		Sevuparin/DF02+Malanil	D2H24	36.4	72	20	114/068
		Sevuparin/DF02+Malanil	D2H30	36.6	66	20	094/060
		Sevuparin/DF02+Malanil	D2H36	36	52	20	094/054
		Sevuparin/DF02+Malanil	D2H42	36.7	72	20	104/061
		Sevuparin/DF02+Malanil	D3H48	36	64	20	116/067
		Sevuparin/DF02+Malanil	D3H54	36	66	20	111/066
		Sevuparin/DF02+Malanil	D3H60	36	68	20	094/056
		Sevuparin/DF02+Malanil	D3H66	37.2	56	20	100/057
		Sevuparin/DF02+Malanil	D7	36.9	64	20	106/070
		Sevuparin/DF02+Malanil	D14	36.7	76	20	106/062
		Malanil	screening	36.7	100	20	106/066
		Malanil	D1H0	36.7	82	20	095/056
		Malanil	D1H6	37.2	86	20	115/068
		Malanil	D1H12	36	78	20	110/073
		Malanil	D1H18	36.8	80	20	109/065
		Malanil	D2H24	38.7	84	20	110/062

SUBJID	INITIAL	ARM	TIMEPOINT	TEMP (˚C)	HR (bpm)	RR (bpm)	BP (mmHg)
		Malanil	D2H30	38.1	86	22	118/067
		Malanil	D2H36	36.3	72	20	094/063
		Malanil	D2H42	36.4	70	20	104/065
		Malanil	D3H48	36.7	66	20	106/064
		Malanil	D3H54	36.7	74	20	106/062
		Malanil	D3H60	36	68	20	104/076
		Malanil	D3H66	36.8	72	20	100/048
		Malanil	D4H72	36	60	20	103/060
		Malanil	D5	36.8	70	20	103/062
		Malanil	D6	36.2	68	20	106/052
		Malanil	D7	36.5	68	20	110/066
		Malanil	D14	36.8	80	20	110/064
		Malanil	screening	36.6	84	20	113/067
		Malanil	D1H0	38.7	102	22	120/069
		Malanil	D1H6	37.7	106	22	123/064
		Malanil	D1H12	36.6	86	20	113/068
		Malanil	D1H18	36.5	76	20	110/070
		Malanil	D2H24	38.3	84	20	114/051
		Malanil	D2H30	36.9	74	20	112/073
		Malanil	D2H36	36.2	64	20	128/066
		Malanil	D2H42	36.3	58	20	102/062
		Malanil	D3H48	36.5	60	20	116/066
		Malanil	D3H54	36.6	72	20	121/061
		Malanil	D3H60	36.3	54	20	101/058
		Malanil	D3H66	36.2	52	20	113/067
		Malanil	D7	36.2	80	20	119/064
		Malanil	D14	36	70	20	110/071
		Malanil	screening	37	88	20	123/076
		Malanil	D1H0	37	88	20	118/068
		Malanil	D1H6	38	86	20	116/069
		Malanil	D1H12	38.4	82	20	121/077
		Malanil	D1H18	36.7	78	20	128/083
		Malanil	D2H24	37.5	88	20	142/088
		Malanil	D2H30	36.7	78	20	105/058
		Malanil	D2H36	37.3	70	20	127/084
		Malanil	D2H42	36.4	70	20	127/078
		Malanil	D3H48	36.2	86	20	140/076

SUBJID	INITIAL	ARM	TIMEPOINT	TEMP (˚C)	HR (bpm)	RR (bpm)	BP (mmHg)
		Malanil	D3H54	36.8	76	20	113/076
		Malanil	D3H60	36.4	76	20	114/077
		Malanil	D3H66	36.3	72	20	108/073
		Malanil	D7	36.3	72	20	114/068
		Malanil	D14	36.5	76	20	114/068
		Malanil	screening	38.8	102	20	123/058
		Malanil	D1H0	38.4	98	20	128/071
		Malanil	D1H6	37.8	78	20	116/068
		Malanil	D1H12	38.6	80	20	102/056
		Malanil	D1H18	36	76	20	109/056
		Malanil	D2H24	39.5	86	20	124/058
		Malanil	D2H30	36.4	60	20	090/050
		Malanil	D2H36	37.1	78	20	107/055
		Malanil	D2H42	36.3	68	20	109/057
		Malanil	D3H48	36.4	84	20	126/064
		Malanil	D3H54	36.3	76	20	111/061
		Malanil	D3H60	36.7	78	20	108/064
		Malanil	D3H66	36.5	70	20	115/062
		Malanil	D7	36.2	92	20	104/077
		Malanil	D14	36.4	78	20	104/060
		Sevuparin/DF02+Malanil	screening	39.9	102	20	107/057
		Sevuparin/DF02+Malanil	D1H0	39.8	92	20	111/052
		Sevuparin/DF02+Malanil	D1H6	38.7	86	20	116/069
		Sevuparin/DF02+Malanil	D1H12	38	92	20	101/057
		Sevuparin/DF02+Malanil	D1H18	36.5	86	20	113/062
		Sevuparin/DF02+Malanil	D2H24	39.5	102	20	113/066
		Sevuparin/DF02+Malanil	D2H30	36.9	68	20	128/088
		Sevuparin/DF02+Malanil	D2H36	38.2	100	20	102/056
		Sevuparin/DF02+Malanil	D2H42	36.5	72	20	103/055
		Sevuparin/DF02+Malanil	D3H48	36.4	76	20	121/058
		Sevuparin/DF02+Malanil	D3H54	36	72	20	092/052
		Sevuparin/DF02+Malanil	D3H60	36.5	58	20	092/053
		Sevuparin/DF02+Malanil	D3H66	36.7	68	20	090/060
		Sevuparin/DF02+Malanil	D7	36	88	20	104/054
		Sevuparin/DF02+Malanil	D14	36.4	74	20	100/060
		Malanil	screening	38	108	20	116/063
		Malanil	D1H0	38.1	110	20	112/061

SUBJID	INITIAL	ARM	TIMEPOINT	TEMP (˚C)	HR (bpm)	RR (bpm)	BP (mmHg)
		Malanil	D1H6	36.6	74	20	109/067
		Malanil	D1H12	36.6	84	20	117/073
		Malanil	D1H18	36.3	86	20	123/075
		Malanil	D2H24	36.4	82	20	117/068
		Malanil	D2H30	38.1	94	20	120/066
		Malanil	D2H36	36.4	70	20	100/053
		Malanil	D2H42	37.3	80	20	106/070
		Malanil	D3H48	37	82	20	110/070
		Malanil	D3H54	37.4	78	20	119/064
		Malanil	D3H60	36.7	76	20	123/074
		Malanil	D3H66	36.3	68	20	119/058
		Malanil	D7	36.9	64	20	106/052
		Malanil	D14	36.9	68	20	116/062
		Malanil	screening	36.9	60	20	115/060
		Malanil	D1H0	36.9	66	20	122/061
		Malanil	D1H6	37.6	64	20	121/055
		Malanil	D1H12	36.6	70	20	121/063
		Malanil	D1H18	36.4	74	20	116/062
		Malanil	D2H24	37	68	20	124/060
		Malanil	D2H30	36.6	66	20	130/090
		Malanil	D2H36	36.7	54	20	113/064
		Malanil	D2H42	36.7	68	20	112/062
		Malanil	D3H48	37.5	58	20	122/066
		Malanil	D3H54	36.8	50	20	131/067
		Malanil	D3H60	36.1	58	20	110/052
		Malanil	D3H66	36	60	20	108/058
		Malanil	D7	37.4	82	20	118/060
		Malanil	D14	36.9	92	20	118/062
		Malanil	screening	37.2	90	20	101/054
		Malanil	D1H0	37.3	82	20	108/061
		Malanil	D1H6	36.8	88	20	101/068
		Malanil	D1H12	36.9	70	20	100/060
		Malanil	D1H18	36.5	68	20	103/061
		Malanil	D2H24	38.4	90	20	124/066
		Malanil	D2H30	39.2	88	20	118/062
		Malanil	D2H36	37.2	82	20	100/056
		Malanil	D2H42	36.6	68	20	106/064

SUBJID	INITIAL	ARM	TIMEPOINT	TEMP (˚C)	HR (bpm)	RR (bpm)	BP (mmHg)
		Malanil	D3H48	36.4	72	20	116/054
		Malanil	D3H54	36.6	68	20	111/061
		Malanil	D3H60	36.2	74	20	102/056
		Malanil	D3H66	36.7	64	20	121/072
		Malanil	D7	36.7	76	20	116/066
		Malanil	D14	36.9	72	20	120/078
		Sevuparin/DF02+Malanil	screening	37.8	104	20	109/060
		Sevuparin/DF02+Malanil	D1H0	37	80	20	100/059
		Sevuparin/DF02+Malanil	D1H6	36.8	68	20	110/070
		Sevuparin/DF02+Malanil	D1H12	36.4	66	20	108/059
		Sevuparin/DF02+Malanil	D1H18	36.6	64	20	106/061
		Sevuparin/DF02+Malanil	D2H24	37.3	84	20	106/055
		Sevuparin/DF02+Malanil	D2H30	38	82	20	113/058
		Sevuparin/DF02+Malanil	D2H36	36.2	80	20	110/060
		Sevuparin/DF02+Malanil	D2H42	36.8	60	20	104/064
		Sevuparin/DF02+Malanil	D3H48	37.4	64	20	103/065
		Sevuparin/DF02+Malanil	D3H54	37.2	60	20	097/058
		Sevuparin/DF02+Malanil	D3H60	36.6	62	20	101/053
		Sevuparin/DF02+Malanil	D3H66	36	66	20	100/060
		Sevuparin/DF02+Malanil	D7	36.4	68	20	115/060
		Sevuparin/DF02+Malanil	D14	36.8	66	20	112/056
		Sevuparin/DF02+Malanil	screening	36.5	82	20	130/064
		Sevuparin/DF02+Malanil	D1H0	37	82	20	112/061
		Sevuparin/DF02+Malanil	D1H6	36.7	72	20	113/065
		Sevuparin/DF02+Malanil	D1H12	36.7	66	20	106/063
		Sevuparin/DF02+Malanil	D1H18	36.8	66	20	114/065
		Sevuparin/DF02+Malanil	D2H24	37.2	70	20	117/077
		Sevuparin/DF02+Malanil	D2H30	36.7	72	20	110/064
		Sevuparin/DF02+Malanil	D2H36	36.4	64	20	117/067
		Sevuparin/DF02+Malanil	D2H42	36.8	68	20	118/066
		Sevuparin/DF02+Malanil	D3H48	36.8	58	20	109/061
		Sevuparin/DF02+Malanil	D3H54	36.9	70	20	108/066
		Sevuparin/DF02+Malanil	D3H60	36.6	78	20	110/070
		Sevuparin/DF02+Malanil	D3H66	36.3	74	20	116/063
		Sevuparin/DF02+Malanil	D7	36.7	80	20	102/060
		Sevuparin/DF02+Malanil	D14	36.9	88	20	117/058
		Sevuparin/DF02+Malanil	screening	39	110	20	115/052

SUBJID	INITIAL	ARM	TIMEPOINT	TEMP (˚C)	HR (bpm)	RR (bpm)	BP (mmHg)
		Sevuparin/DF02+Malanil	D1H0	37.7	88	20	104/052
		Sevuparin/DF02+Malanil	D1H6	36.7	76	20	107/061
		Sevuparin/DF02+Malanil	D1H12	36.9	78	20	104/058
		Sevuparin/DF02+Malanil	D1H18	38.3	86	20	102/052
		Sevuparin/DF02+Malanil	D2H24	37.7	74	20	112/054
		Sevuparin/DF02+Malanil	D2H30	37.5	76	20	114/061
		Sevuparin/DF02+Malanil	D2H36	37.2	78	20	117/058
		Sevuparin/DF02+Malanil	D2H42	37	76	20	112/074
		Sevuparin/DF02+Malanil	D3H48	36.2	76	20	102/072
		Sevuparin/DF02+Malanil	D3H54	36.5	58	20	103/051
		Sevuparin/DF02+Malanil	D3H60	36.6	74	20	114/067
		Sevuparin/DF02+Malanil	D3H66	36	58	20	118/059
		Sevuparin/DF02+Malanil	D7	36.8	86	20	116/060
		Sevuparin/DF02+Malanil	D14	36.7	88	20	127/066
		Sevuparin/DF02+Malanil	screening	38	106	20	101/059
		Sevuparin/DF02+Malanil	D1H0	37.7	84	20	103/053
		Sevuparin/DF02+Malanil	D1H6	39.6	84	22	130/089
		Sevuparin/DF02+Malanil	D1H12	37.2	60	22	110/070
		Sevuparin/DF02+Malanil	D1H18	38.6	84	22	117/056
		Sevuparin/DF02+Malanil	D2H24	38.5	84	20	106/056
		Sevuparin/DF02+Malanil	D2H30	37.5	78	20	101/054
		Sevuparin/DF02+Malanil	D2H36	38.5	78	22	105/055
		Sevuparin/DF02+Malanil	D2H42	36.6	74	20	110/070
		Sevuparin/DF02+Malanil	D3H48	37	72	20	094/062
		Sevuparin/DF02+Malanil	D3H54	36.7	72	20	095/057
		Sevuparin/DF02+Malanil	D3H60	36.8	62	20	090/060
		Sevuparin/DF02+Malanil	D3H66	36	74	20	090/060
		Sevuparin/DF02+Malanil	D7	36.9	96	20	115/063
		Sevuparin/DF02+Malanil	D14	37	106	20	116/060
		Sevuparin/DF02+Malanil	screening	37.8	86	20	121/056
		Sevuparin/DF02+Malanil	D1H0	37.4	70	20	098/054
		Sevuparin/DF02+Malanil	D1H6	36.3	70	20	100/060
		Sevuparin/DF02+Malanil	D1H12	37.3	86	20	108/063
		Sevuparin/DF02+Malanil	D1H18	36.5	78	20	109/059
		Sevuparin/DF02+Malanil	D2H24	38.2	90	20	110/060
		Sevuparin/DF02+Malanil	D2H30	37.7	82	20	112/054
		Sevuparin/DF02+Malanil	D2H36	37	90	20	090/060

SUBJID	INITIAL	ARM	TIMEPOINT	TEMP (˚C)	HR (bpm)	RR (bpm)	BP (mmHg)
		Sevuparin/DF02+Malanil	D2H42	36.3	64	20	110/057
		Sevuparin/DF02+Malanil	D3H48	36.7	74	20	098/068
		Sevuparin/DF02+Malanil	D3H54	36.6	76	20	108/064
		Sevuparin/DF02+Malanil	D3H60	37.2	62	20	090/060
		Sevuparin/DF02+Malanil	D3H66	36.2	56	20	090/060
		Sevuparin/DF02+Malanil	D7	36.6	62	20	108/056
		Sevuparin/DF02+Malanil	D14	36.3	78	20	100/060
		Malanil	screening	38.6	98	20	114/061
		Malanil	D1H0	39.3	106	20	117/068
		Malanil	D1H6	37	90	20	117/071
		Malanil	D1H12	36.7	98	20	128/073
		Malanil	D1H18	36.8	96	20	110/074
		Malanil	D2H24	38.2	98	20	120/072
		Malanil	D2H30	36.7	82	20	114/069
		Malanil	D2H36	38.2	86	22	096/052
		Malanil	D2H42	37.2	100	22	100/060
		Malanil	D3H48	38.2	100	20	110/080
		Malanil	D3H54	37.4	88	20	090/060
		Malanil	D3H60	37.1	84	20	112/084
		Malanil	D3H66	36.1	84	20	109/067
		Malanil	D4H72	36.2	88	20	100/060
		Malanil	D5	36.5	84	20	110/076
		Malanil	D7	36.9	88	20	102/064
		Malanil	D14	37	100	20	098/060
		Malanil	screening	38	104	20	100/060
		Malanil	D1H0	38.1	110	22	104/058
		Malanil	D1H6	38.4	102	20	108/065
		Malanil	D1H12	38	100	20	106/062
		Malanil	D1H18	36.7	88	20	100/061
		Malanil	D2H24	37	100	20	108/060
		Malanil	D2H30	38.2	106	20	100/066
		Malanil	D2H36	36.8	96	20	100/067
		Malanil	D2H42	38.6	92	20	116/071
		Malanil	D3H48	37.2	92	20	112/063
		Malanil	D3H54	38.8	96	20	122/068
		Malanil	D3H60	37	74	20	113/064
		Malanil	D3H66	36.5	82	20	117/062

SUBJID	INITIAL	ARM	TIMEPOINT	TEMP (˚C)	HR (bpm)	RR (bpm)	BP (mmHg)
		Malanil	D7	36.7	86	20	119/064
		Malanil	D14	36	96	20	132/074
		Malanil	screening	38.9	100	20	112/060
		Malanil	D1H0	39.3	98	20	134/060
		Malanil	D1H6	37.9	96	22	111/062
		Malanil	D1H12	36.6	86	20	107/065
		Malanil	D1H18	36.7	84	20	107/062
		Malanil	D2H24	36.1	80	20	106/063
		Malanil	D2H30	38.1	88	20	107/055
		Malanil	D2H36	37.6	82	20	100/054
		Malanil	D2H42	39	92	22	098/056
		Malanil	D3H48	37.8	78	20	087/053
		Malanil	D3H54	37.1	80	20	101/059
		Malanil	D3H60	36.7	74	20	115/052
		Malanil	D3H66	36.2	66	20	097/057
		Malanil	D4H72	36.7	82	20	107/065
		Malanil	D5	36.5	68	20	110/062
		Malanil	D7	36.6	86	20	110/060
		Malanil	D14	36.8	96	20	100/060

16.2.7.5 ECG

USUBJID	INITIAL	ARM	TIMEPOINT	QTCmsec	RESULT	RESOTHER
		Malanil	screening	397	Normal	
		Malanil	D1	399	Normal	
		Malanil	D3	406	Normal	
		Sevuparin/DF02+Malanil	screening	428	Normal	
		Sevuparin/DF02+Malanil	D1	417	Normal	
		Sevuparin/DF02+Malanil	D3	409	Normal	
		Sevuparin/DF02+Malanil	screening	412	Normal	
		Sevuparin/DF02+Malanil	D1	435	Normal	
		Sevuparin/DF02+Malanil	D3	420	Normal	
		Malanil	screening	438	Normal	
		Malanil	D1	416	Normal	
		Malanil	D3	439	Normal	
		Malanil	screening	394	Normal	
		Malanil	D1	398	Normal	
		Malanil	D3	399	Normal	
		Sevuparin/DF02+Malanil	screening	399	Normal	
		Sevuparin/DF02+Malanil	D1	399	Normal	
		Sevuparin/DF02+Malanil	D3	428	Normal	
		Malanil	screening	398	Normal	
		Malanil	D1	394	Normal	
		Malanil	D3	427	Normal	
		Sevuparin/DF02+Malanil	screening	408	Normal	
		Sevuparin/DF02+Malanil	D1	395	Normal	
		Sevuparin/DF02+Malanil	D3	397	Normal	
		Sevuparin/DF02+Malanil	screening	419	Normal	
		Sevuparin/DF02+Malanil	D1	407	Normal	
		Sevuparin/DF02+Malanil	D3	419	Normal	
		Malanil	screening	411	Normal	
		Malanil	D1	407	Normal	
		Malanil	D3	417	Normal	
		Malanil	screening	379	Normal	
		Malanil	D1	365	Normal	
		Malanil	D3	400	Normal	
		Sevuparin/DF02+Malanil	screening	425	Normal	
		Sevuparin/DF02+Malanil	D1	427	Normal	

USUBJID	INITIAL	ARM	TIMEPOINT	QTCmsec	RESULT	RESOTHER
		Sevuparin/DF02+Malanil	D3	400	Normal	
		Sevuparin/DF02+Malanil	screening	402	Normal	
		Sevuparin/DF02+Malanil	D1	392	Normal	
		Sevuparin/DF02+Malanil	D3	404	Normal	
		Malanil	screening	394	Normal	
		Malanil	D1	388	Normal	
		Malanil	D3	380	Normal	
		Malanil	screening	446	Normal	
		Malanil	D1	446	Normal	
		Malanil	D3	389	Normal	
		Malanil	screening	409	Normal	
		Malanil	D1	425	Normal	
		Malanil	D3	447	Normal	
		Sevuparin/DF02+Malanil	screening	386	Normal	
		Sevuparin/DF02+Malanil	D1	394	Normal	
		Sevuparin/DF02+Malanil	D3	400	Normal	
		Sevuparin/DF02+Malanil	screening	374	Normal	
		Sevuparin/DF02+Malanil	D1	412	Normal	
		Sevuparin/DF02+Malanil	D3	420	Normal	
		Sevuparin/DF02+Malanil	screening	387	Normal	
		Sevuparin/DF02+Malanil	D1	387	Normal	
		Sevuparin/DF02+Malanil	D3	432	Normal	
		Malanil	screening	436	Normal	
		Malanil	D1	445	Normal	
		Malanil	D3	448	Normal	
		Sevuparin/DF02+Malanil	screening	363	Normal	
		Sevuparin/DF02+Malanil	D1	362	Normal	
		Sevuparin/DF02+Malanil	D3	400	Normal	
		Sevuparin/DF02+Malanil	screening	404	Normal	
		Sevuparin/DF02+Malanil	D1	438	Normal	
		Sevuparin/DF02+Malanil	D3	456	Abnormal	QTC PROLONG
		Sevuparin/DF02+Malanil	screening	412	Normal	
		Sevuparin/DF02+Malanil	D1	408	Normal	
		Sevuparin/DF02+Malanil	D3	398	Normal	
		Sevuparin/DF02+Malanil	screening	392	Normal	
		Sevuparin/DF02+Malanil	D1	392	Normal	
		Sevuparin/DF02+Malanil	D3	424	Normal	

USUBJID	INITIAL	ARM	TIMEPOINT	QTCmsec	RESULT	RESOTHER
		Malanil	screening	438	Normal	
		Malanil	D1	436	Normal	
		Malanil	D3	445	Abnormal	PVC
		Malanil	screening	429	Normal	
		Malanil	D1	420	Normal	
		Malanil	D3	431	Normal	
		Malanil	screening	426	Normal	
		Malanil	D1	430	Normal	
		Malanil	D3	427	Normal	
		Sevuparin/DF02+Malanil	screening	409	Normal	
		Sevuparin/DF02+Malanil	D1	395	Normal	
		Sevuparin/DF02+Malanil	D3	425	Normal	
		Malanil	screening	415	Normal	
		Malanil	D1	475	Abnormal	QTC PROLONG
		Malanil	D3	538	Abnormal	QTC PROLONG
		Malanil	screening	407	Normal	
		Malanil	D1	383	Normal	
		Malanil	D3	447	Normal	
		Malanil	screening	448	Normal	
		Malanil	D1	428	Normal	
		Malanil	D3	442	Normal	
		Malanil	screening	391	Normal	
		Malanil	D1	389	Normal	
		Malanil	D3	408	Normal	
		Sevuparin/DF02+Malanil	screening	376	Normal	
		Sevuparin/DF02+Malanil	D1	373	Normal	
		Sevuparin/DF02+Malanil	D3	400	Normal	
		Malanil	screening	389	Normal	
		Malanil	D1	389	Normal	
		Malanil	D3	413	Normal	
		Malanil	screening	437	Normal	
		Malanil	D1	403	Normal	
		Malanil	D3	437	Abnormal	SINUS BRADYCARDIA
		Malanil	screening	402	Normal	
		Malanil	D1	400	Normal	
		Malanil	D3	420	Normal	
		Sevuparin/DF02+Malanil	screening	446	Normal	

USUBJID	INITIAL	ARM	TIMEPOINT	QTCmsec	RESULT	RESOTHER
		Sevuparin/DF02+Malanil	D1	445	Normal	
		Sevuparin/DF02+Malanil	D3	457	Abnormal	QTC PROLONG BUT NOT SIGNIFINICANT
		Sevuparin/DF02+Malanil	screening	401	Normal	
		Sevuparin/DF02+Malanil	D1	385	Normal	
		Sevuparin/DF02+Malanil	D3	396	Normal	
		Sevuparin/DF02+Malanil	screening	381	Normal	
		Sevuparin/DF02+Malanil	D1	409	Normal	
		Sevuparin/DF02+Malanil	D3	431	Normal	
		Sevuparin/DF02+Malanil	screening	395	Normal	
		Sevuparin/DF02+Malanil	D1	409	Normal	
		Sevuparin/DF02+Malanil	D3	408	Normal	
		Sevuparin/DF02+Malanil	screening	390	Normal	
		Sevuparin/DF02+Malanil	D1	390	Normal	
		Sevuparin/DF02+Malanil	D3	407	Normal	
		Malanil	screening	395	Normal	
		Malanil	D1	389	Normal	
		Malanil	D3	391	Normal	
		Malanil	screening	366	Normal	
		Malanil	D1	370	Normal	
		Malanil	D3	392	Normal	
		Malanil	screening	373	Normal	
		Malanil	D1	401	Normal	
		Malanil	D3	422	Normal	

16.2.8 Listing of individual laboratory measurements by subject

16.2.8.1 Haematology

SUBJID	INITIAL	ARM	DAY	RBC ($10^6/\mu L$) *99=Not avalible*	HCT (%) *99=Not avalible*	HB (g/dL) *99=Not avalible*	MCV (fL) *999=Not avalible*	MCH (pg/cell) *999=Not avalible*	MCHC (pg/cell) *999=Not avalible*	WBC ($10^3/ \mu L$) *99=Not avalible*	NEU (%) *999=Not avalible*	LYM (%) *999=Not avalible*	MON (%) *999=Not avalible*	EOS (%) *999=Not avalible*	PLT ($10^3/ \mu L$) *999=Not avalible*
		Malanil	1	4.2	36.2	12.7	85.9	30.1	35	7.5	62	26	6	6	132
		Malanil	2	3.7	32.5	11.4	87.1	30.5	35	5.3	64	25	5	4	101
		Malanil	3	3.8	33.2	11.8	86.6	30.9	35.7	5.2	58	32	5	5	121
		Sevuparin/DF02+ Malanil	1	4.9	31.5	10.1	65	20.9	32.2	4.8	49	32	2	15	184
		Sevuparin/DF02+ Malanil	2	4.7	30.4	9.9	65.4	21.2	32.4	4.5	45	38	4	11	125
		Sevuparin/DF02+ Malanil	3	4.5	29.9	9.5	66.1	21	31.7	5.2	32	38	3	25	168
		Sevuparin/DF02+ Malanil	7	4.6	29.8	9.6	64.7	21	31.7	6.9	34	50	2	14	199
		Sevuparin/DF02+ Malanil	14	4.5	28.9	9.6	63.9	21.2	33.1	5.3	32	51	5	10	150
		Sevuparin/DF02+ Malanil	1	4.1	34.1	11.7	74.7	25.7	34.4	5.6	65	26	3	3	76
		Sevuparin/DF02+ Malanil	2	3.9	30.7	10.6	75	25.7	34.3	4.7	61	30	4	4	81
		Sevuparin/DF02+ Malanil	3	3.9	29.1	10.3	73.7	26	35.2	4.5	44	45	3	4	78
		Sevuparin/DF02+ Malanil	7	4.3	32.6	11	75.6	25.5	33.7	7.6	54	36	0	10	248

SUBJID	INITIAL	ARM	DAY	RBC ($10^6/\mu L$) *99=Not avalible*	HCT (%) *99=Not avalible*	HB (g/dL) *99=Not avalible*	MCV (fL) *999=Not avalible*	MCH (pg/cell) *999=Not avalible*	MCHC (pg/cell) *999=Not avalible*	WBC ($10^3/\mu L$) *99=Not avalible*	NEU (%) *999=Not avalible*	LYM (%) *999=Not avalible*	MON (%) *999=Not avalible*	EOS (%) *999=Not avalible*	PLT ($10^3/\mu L$) *999=Not avalible*
		Sevuparin/DF02+ Malanil	14	4.4	33.1	11.1	76.2	25.6	33.6	7.4	46	49	1	4	193
		Malanil	1	5.3	39.6	13.2	74.9	25	33.4	5.4	60	25	6	4	122
		Malanil	2	4.8	35.6	11.8	74.3	24.6	33.2	3.4	50	35	5	8	104
		Malanil	3	4.7	34.5	11.4	74.2	24.6	33.1	3.8	37	45	8	10	159
		Malanil	7	4.9	36.5	12.4	74.4	25.2	33.8	8.4	57	36	2	3	321
		Malanil	14	5	36.6	12	73.8	24.2	32.8	5.5	45	45	3	5	337
		Malanil	1	3.5	33	12.4	94.6	35.6	37.6	7.7	76	17	4	1	180
		Malanil	2	3.1	29.2	11	94.7	35.6	37.5	5.3	52	35	4	6	111
		Malanil	3	2.9	27.1	10.3	94.9	36.1	38.1	4.8	50	45	2	3	98
		Malanil	7	3.4	32.5	11.8	96.1	35	36.4	7.4	58	33	2	4	202
		Malanil	14	3.7	34.9	12.3	86.4	32.2	34.8	7	60	32	2	6	236
		Sevuparin/DF02+ Malanil	1	5.2	42.3	14.5	80.9	27.8	34.4	3.7	82	12	2	2	66
		Sevuparin/DF02+ Malanil	2	4.9	38.9	13.4	79.8	27.4	34.3	3.6	45	42	3	7	79
		Sevuparin/DF02+ Malanil	3	4.6	36.6	12.2	80.2	26.8	33.4	3.5	45	43	3	7	74
		Sevuparin/DF02+ Malanil	7	4.2	34	13	80.5	30.7	38.2	5.7	55	37	3	5	171
		Sevuparin/DF02+ Malanil	14	4.5	36.9	12.6	81.3	27.8	34.2	7.2	51	40	3	6	167
		Malanil	1	4.6	33.2	11.4	71.5	24.5	34.3	4.3	62	30	3	2	56
		Malanil	2	4.2	29.6	10.5	69.9	24.8	35.4	3.6	36	51	7	2	64

SUBJID	INITIAL	ARM	DAY	RBC ($10^6/\mu L$) *99=Not avalible*	HCT (%) *99=Not avalible*	HB (g/dL) *99=Not avalible*	MCV (fL) *999=Not avalible*	MCH (pg/cell) *999=Not avalible*	MCHC (pg/cell) *999=Not avalible*	WBC ($10^3/\mu L$) *99=Not avalible*	NEU (%) *999=Not avalible*	LYM (%) *999=Not avalible*	MON (%) *999=Not avalible*	EOS (%) *999=Not avalible*	PLT ($10^3/\mu L$) *999=Not avalible*
		Malanil	3	4.4	31.2	11.1	70.8	25.2	35.6	4.4	46	43	5	2	68
		Malanil	7	4.6	33.1	11	72.3	24.1	33.3	6.3	49	43	4	3	197
		Sevuparin/DF02+ Malanil	1	5.2	41.3	14	78.7	26.7	33.9	6.5	74	17	3	5	154
		Sevuparin/DF02+ Malanil	2	4.6	35.3	12.4	77.5	27.2	35.1	5.2	70	20	6	4	86
		Sevuparin/DF02+ Malanil	3	4.8	37.2	12.8	76.9	26.4	34.3	5.2	59	27	7	5	98
		Sevuparin/DF02+ Malanil	7	4.8	37.3	13.2	77.9	27.5	35.3	11.5	75	19	4	2	239
		Sevuparin/DF02+ Malanil	14	5.4	41.6	13.9	77.5	25.8	33.4	7.7	66	27	4	3	318
		Sevuparin/DF02+ Malanil	1	4.6	36.4	12.6	72.7	25.1	34.5	8.3	74	20	3	1	114
		Sevuparin/DF02+ Malanil	2	4	28.5	9.9	71.6	24.9	34.8	5	52	40	6	1	81
		Sevuparin/DF02+ Malanil	3	4.1	29.6	10.1	72.4	24.6	34	4.4	61	32	4	3	71
		Sevuparin/DF02+ Malanil	7	4	28.7	9.6	72.4	24.3	33.6	5.8	54	40	4	2	228
		Sevuparin/DF02+ Malanil	14	4.2	32	10.5	76.2	24.9	32.7	6.2	48	45	4	3	311
		Malanil	1	5.4	35.6	11.6	65.4	21.2	32.5	7.7	64	28	4	4	207
		Malanil	2	5.2	33.8	11.1	65	21.4	32.9	5	54	37	4	5	158
		Malanil	3	5.4	34.6	11.4	64.4	21.1	32.9	5	55	40	3	2	154

SUBJID	INITIAL	ARM	DAY	RBC (10^6/µL) *99=Not avalible*	HCT (%) *99=Not avalible*	HB (g/dL) *99=Not avalible*	MCV (fL) *999=Not avalible*	MCH (pg/cell) *999=Not avalible*	MCHC (pg/cell) *999=Not avalible*	WBC (10^3/ µL) *99=Not avalible*	NEU (%) *999=Not avalible*	LYM (%) *999=Not avalible*	MON (%) *999=Not avalible*	EOS (%) *999=Not avalible*	PLT (10^3/ µL) *999=Not avalible*
		Malanil	7	5.1	33.6	11.2	65.6	21.8	33.3	8.2	54	37	4	5	294
		Malanil	14	5.4	35.7	11.5	66.3	21.4	32.3	8.6	64	31	3	2	271
		Malanil	1	4.3	35.5	12.3	82.5	28.5	34.6	4.9	70	24	4	2	93
		Malanil	2	4	32.6	11.4	81.7	28.6	35	4	53	40	5	2	98
		Malanil	3	4	33.2	11.4	82.3	28.2	34.2	5.2	61	33	4	2	122
		Malanil	7	4.2	35.1	12.1	82.8	28.5	34.4	8.1	68	25	5	2	255
		Malanil	14	4.8	34.7	12	72.7	25.2	34.6	4.6	60	35	3	2	180
		Sevuparin/DF02+ Malanil	1	4.3	35.4	12.2	81.7	28.1	34.4	3.2	42	50	5	3	58
		Sevuparin/DF02+ Malanil	2	4.2	34	11.8	81	28.1	34.7	3	44	40	8	4	65
		Sevuparin/DF02+ Malanil	3	4.2	33.8	11.7	81	28.1	34.6	3.4	43	44	7	2	70
		Sevuparin/DF02+ Malanil	7	4	32.5	11.1	82.1	27.9	34	3	45	48	5	2	170
		Sevuparin/DF02+ Malanil	1	5.3	36	11.6	68	21.9	32.2	4.7	79	18	1	2	77
		Sevuparin/DF02+ Malanil	2	4.7	31.1	10.2	66.6	21.8	32.8	2.2	66	27	5	2	35
		Sevuparin/DF02+ Malanil	3	4.5	29.6	9.8	66.6	22	33.1	2.2	50	38	8	4	36
		Sevuparin/DF02+ Malanil	7	4.5	30.6	9.8	67.7	21.7	32.1	5	57	30	7	6	160
		Sevuparin/DF02+ Malanil	14	4.9	33.2	10.5	68.4	21.7	31.8	6.4	62	30	4	4	237

SUBJID	INITIAL	ARM	DAY	RBC ($10^6/\mu L$) *99=Not avalible*	HCT (%) *99=Not avalible*	HB (g/dL) *99=Not avalible*	MCV (fL) *999=Not avalible*	MCH (pg/cell) *999=Not avalible*	MCHC (pg/cell) *999=Not avalible*	WBC ($10^3/\mu L$) *99=Not avalible*	NEU (%) *999=Not avalible*	LYM (%) *999=Not avalible*	MON (%) *999=Not avalible*	EOS (%) *999=Not avalible*	PLT ($10^3/\mu L$) *999=Not avalible*
		Malanil	1	5.6	45.6	15.9	80.9	28.2	34.9	5.9	70	20	6	2	109
		Malanil	2	5.1	41.5	14.6	80.8	28.3	35.1	3.9	72	20	5	3	81
		Malanil	3	5.2	41.4	14.7	79.8	28.2	35.4	4.8	62	30	5	3	66
		Malanil	7	5.5	44.5	15.5	80.6	28.1	34.8	4.8	58	30	6	6	192
		Malanil	14	5.8	47.3	16.3	81	27.9	34.4	5.5	56	32	6	6	188
		Malanil	1	4.9	40.4	14.1	82.5	28.8	35	5.1	78	17	2	3	53
		Malanil	2	4.4	35.8	12.6	82.3	28.9	35.1	5.6	75	15	5	3	24
		Malanil	3	4.1	33.5	12	81.5	29.3	36	4.7	66	24	5	5	41
		Malanil	7	3.8	31.3	11.1	83.4	29.7	35.6	4.5	52	40	6	2	188
		Malanil	14	4	33.4	11.7	84.8	29.7	35	5.5	64	30	4	2	348
		Malanil	1	4.9	37.6	12.7	76.6	25.9	33.8	4.7	70	20	5	5	204
		Malanil	2	4.8	36.5	12.5	76.3	26.1	34.2	4.8	59	31	4	6	139
		Malanil	3	5.1	39.3	13.3	76.6	25.9	33.9	4.8	45	42	8	3	154
		Malanil	7	4.8	37.8	12.5	78.2	25.8	32.9	6.2	47	45	3	5	219
		Malanil	14	5.3	41.4	13.8	77.8	26	33.5	7.8	48	40	5	7	267
		Sevuparin/DF02+ Malanil	1	3.5	30.5	10.4	87.7	30	34.3	5	66	31	2	1	302
		Sevuparin/DF02+ Malanil	2	2.9	25.5	9	87.7	30.9	35.2	4.1	50	40	6	3	225
		Sevuparin/DF02+ Malanil	3	3.1	26.8	9.4	87.1	30.4	34.9	4.8	53	36	7	4	243
		Sevuparin/DF02+ Malanil	7	3.3	28.8	10.4	87.8	31.6	36	5.7	65	27	5	3	397

SUBJID	INITIAL	ARM	DAY	RBC (10^6/µL) *99=Not avalible*	HCT (%) *99=Not avalible*	HB (g/dL) *99=Not avalible*	MCV (fL) *999=Not avalible*	MCH (pg/cell) *999=Not avalible*	MCHC (pg/cell) *999=Not avalible*	WBC (10^3/ µL) *99=Not avalible*	NEU (%) *999=Not avalible*	LYM (%) *999=Not avalible*	MON (%) *999=Not avalible*	EOS (%) *999=Not avalible*	PLT (10^3/ µL) *999=Not avalible*
		Sevuparin/DF02+ Malanil	14	3.8	32.9	11.2	86.7	29.5	34	5.8	65	30	3	2	383
		Sevuparin/DF02+ Malanil	1	4.8	38.4	13.4	80.3	28	34.9	4.2	78	14	6	2	98
		Sevuparin/DF02+ Malanil	2	4.3	34.2	11.9	79.4	27.6	34.7	3.3	61	30	7	2	90
		Sevuparin/DF02+ Malanil	3	4.1	32.5	11.8	78.6	28.5	36.2	3.8	58	35	6	1	96
		Sevuparin/DF02+ Malanil	7	3.8	30.3	10.8	80.2	28.5	35.6	5	68	28	2	2	161
		Sevuparin/DF02+ Malanil	14	3.7	30.2	10.6	80.9	28.3	35	5.6	75	20	2	3	233
		Sevuparin/DF02+ Malanil	1	5.3	38.8	12.8	73.9	24.4	33	5.7	80	18	1	1	112
		Sevuparin/DF02+ Malanil	2	4.5	32.2	11.1	72	24.8	34.4	4.3	50	43	6	1	66
		Sevuparin/DF02+ Malanil	3	4.2	30.1	10.4	71.8	24.8	34.5	4.4	38	55	5	2	84
		Sevuparin/DF02+ Malanil	7	4.1	29.5	10	72.1	24.5	34	5.1	39	50	5	6	222
		Sevuparin/DF02+ Malanil	14	4.5	33.1	11.1	73.8	24.8	33.6	5.4	49	42	4	5	285
		Malanil	1	5.6	43.5	15	78.1	26.9	34.5	6.3	66	25	6	3	91
		Malanil	2	5.3	41.4	14.1	78.3	26.7	34.1	4.2	51	35	8	4	65
		Malanil	3	4.9	38.3	13.5	77.5	27.3	35.2	4.3	52	33	10	5	64
		Malanil	7	4.6	36.4	12.4	79.8	27.1	34	6.1	52	38	7	3	168

SUBJID	INITIAL	ARM	DAY	RBC (10^6/µL) *99=Not avalible*	HCT (%) *99=Not avalible*	HB (g/dL) *99=Not avalible*	MCV (fL) *999=Not avalible*	MCH (pg/cell) *999=Not avalible*	MCHC (pg/cell) *999=Not avalible*	WBC (10^3/ µL) *99=Not avalible*	NEU (%) *999=Not avalible*	LYM (%) *999=Not avalible*	MON (%) *999=Not avalible*	EOS (%) *999=Not avalible*	PLT (10^3/ µL) *999=Not avalible*
		Malanil	14	4.9	39	12.9	79.6	26.4	33.1	5.2	60	30	6	2	172
		Sevuparin/DF02+ Malanil	1	5.1	46.3	16.4	90.7	32.1	35.4	4.1	69	24	5	2	154
		Sevuparin/DF02+ Malanil	2	4.6	41.4	14.8	89.7	32	35.8	5.3	64	30	5	1	94
		Sevuparin/DF02+ Malanil	3	4.6	40.6	14.9	88.6	32.5	36.7	5.6	58	32	8	2	108
		Sevuparin/DF02+ Malanil	7	4.7	41.6	14.7	88.4	31.2	35.3	5.9	44	45	4	7	175
		Sevuparin/DF02+ Malanil	14	4.3	38.3	13.1	88.3	30.2	34.2	9	38	52	4	6	336
		Sevuparin/DF02+ Malanil	1	6.1	37.7	12.9	62.2	21.4	34.4	9.6	82	12	3	3	63
		Sevuparin/DF02+ Malanil	2	5.1	32	10.8	62.8	21.2	33.7	5.7	61	30	7	2	75
		Sevuparin/DF02+ Malanil	3	4.9	30.2	10.6	62	21.7	35	5.2	50	40	8	2	120
		Sevuparin/DF02+ Malanil	7	5.1	32.4	11.2	63.4	21.8	34.4	10.9	60	32	6	2	576
		Sevuparin/DF02+ Malanil	14	6.3	40.3	13	63.6	20.5	32.2	13.6	36	57	5	2	219
		Sevuparin/DF02+ Malanil	1	6.1	45.6	14.9	74.4	24.4	32.8	8.3	85	11	2	2	56
		Sevuparin/DF02+ Malanil	2	5.3	38.8	12.9	73	24.3	33.3	7	56	35	6	3	67
		Sevuparin/DF02+	3	4.9	35.7	12.2	72.3	24.7	34.2	6.6	45	45	8	2	76

SUBJID	INITIAL	ARM	DAY	RBC ($10^6/\mu L$) *99=Not avalible*	HCT (%) *99=Not avalible*	HB (g/dL) *99=Not avalible*	MCV (fL) *999=Not avalible*	MCH (pg/cell) *999=Not avalible*	MCHC (pg/cell) *999=Not avalible*	WBC ($10^3/\mu L$) *99=Not avalible*	NEU (%) *999=Not avalible*	LYM (%) *999=Not avalible*	MON (%) *999=Not avalible*	EOS (%) *999=Not avalible*	PLT ($10^3/\mu L$) *999=Not avalible*
		Malanil													
		Sevuparin/DF02+ Malanil	7	4.4	31.8	10.7	73.2	24.7	33.7	9.5	51	40	5	4	235
		Sevuparin/DF02+ Malanil	14	3.4	27.3	9.6	81	28.3	35	10.8	55	31	4	10	271
		Sevuparin/DF02+ Malanil	1	5.5	42	13.9	76.2	25.2	33.1	7.4	78	15	5	2	87
		Sevuparin/DF02+ Malanil	2	5.3	39.3	13	74.6	24.6	33	4.7	66	25	5	4	53
		Sevuparin/DF02+ Malanil	3	5	36.9	12.5	73.7	25	33.9	6.1	51	35	9	5	72
		Sevuparin/DF02+ Malanil	7	5	36.8	12.3	74.1	24.8	33.4	7.7	62	28	7	3	178
		Sevuparin/DF02+ Malanil	14	4.9	36.6	12.2	75.2	24.9	33.2	10.5	44	49	3	4	222
		Malanil	1	4.6	33.7	11.4	73.1	24.7	33.8	4.8	75	20	3	2	63
		Malanil	2	4.7	33.9	11.4	72.4	24.3	33.7	6	66	30	3	1	47
		Malanil	3	4.7	33.9	11.4	72	24.1	33.5	6	54	37	7	2	30
		Malanil	7	4.2	30.7	10.2	72.6	24.2	33.3	5	56	38	4	2	266
		Malanil	14	4.2	30.9	10.4	73.1	24.6	33.6	4.7	42	50	5	3	284
		Malanil	1	4.8	37.7	12.8	78.4	26.7	34.1	4.9	50	40	4	6	153
		Malanil	2	4.9	38.3	12.8	77.7	26	33.5	5	75	18	6	1	109
		Malanil	3	5	38.1	12.6	77	25.4	32.9	5	45	50	3	2	106
		Malanil	7	4.6	35.4	11.8	77.1	25.6	33.2	6.5	54	38	3	5	281

SUBJID	INITIAL	ARM	DAY	RBC ($10^6/\mu L$) *99=Not avalible*	HCT (%) *99=Not avalible*	HB (g/dL) *99=Not avalible*	MCV (fL) *999=Not avalible*	MCH (pg/cell) *999=Not avalible*	MCHC (pg/cell) *999=Not avalible*	WBC ($10^3/\mu L$) *99=Not avalible*	NEU (%) *999=Not avalible*	LYM (%) *999=Not avalible*	MON (%) *999=Not avalible*	EOS (%) *999=Not avalible*	PLT ($10^3/\mu L$) *999=Not avalible*
		Malanil	14	4.5	35.7	11.6	79.1	25.6	32.4	6.2	49	40	3	8	315
		Malanil	1	3.8	34.5	12.1	90.3	31.8	35.2	6.6	81	15	2	2	107
		Malanil	2	3.7	32.3	11.6	88.6	31.8	35.9	4.4	68	24	5	3	122
		Malanil	3	3.7	33.5	12	89.6	32	35.7	4.7	59	30	6	5	109
		Malanil	7	3.6	31.7	11.4	88	31.6	35.9	6.7	70	25	3	2	156
		Malanil	14	3.7	32.5	11.5	87.1	30.8	35.3	8	65	27	3	4	110
		Sevuparin/DF02+ Malanil	1	5.4	43	14.2	80.5	26.5	33	4.1	64	30	3	3	71
		Sevuparin/DF02+ Malanil	2	4.7	37.2	12.6	79.7	27.1	33.9	3.3	42	50	4	4	28
		Sevuparin/DF02+ Malanil	3	4.5	36.2	12.3	80	27.2	34.1	3.4	54	40	1	5	29
		Sevuparin/DF02+ Malanil	7	4.6	35.8	12	78.6	26.3	33.4	4.9	49	41	3	7	257
		Sevuparin/DF02+ Malanil	14	4.6	37.3	12.6	80.9	27.3	33.7	5.2	64	30	3	3	130
		Malanil	1	4.8	43.5	14.5	83.5	29.2	35	6.8	80	13	2	5	143
		Malanil	2	5	41.3	14.3	82.6	28.6	34.6	6.2	66	28	4	2	87
		Malanil	3	5.1	41.8	14.4	81.5	28	34.4	6	63	30	5	2	101
		Malanil	7	5	41.6	14	83.2	28	33.6	7.7	41	50	4	5	273
		Malanil	14	4.5	39.1	12.9	86.2	28.5	33.1	9	54	40	3	3	402
		Malanil	1	4.6	41	13.8	88.6	30	33.8	6.1	76	16	5	3	138
		Malanil	2	4.2	36.7	13	87.2	30.8	35.3	3.4	63	30	5	2	86
		Malanil	3	4.4	37.8	13.2	86.8	30.3	35	3.4	55	40	4	1	123

SUBJID	INITIAL	ARM	DAY	RBC ($10^6/\mu L$) *99=Not avalible*	HCT (%) *99=Not avalible*	HB (g/dL) *99=Not avalible*	MCV (fL) *999=Not avalible*	MCH (pg/cell) *999=Not avalible*	MCHC (pg/cell) *999=Not avalible*	WBC ($10^3/ \mu L$) *99=Not avalible*	NEU (%) *999=Not avalible*	LYM (%) *999=Not avalible*	MON (%) *999=Not avalible*	EOS (%) *999=Not avalible*	PLT ($10^3/ \mu L$) *999=Not avalible*
		Malanil	7	3.8	33.2	12.1	86.4	31.4	36.4	6.6	55	36	4	5	254
		Malanil	14	4.2	37.1	13	88.9	31.2	35.1	6	55	40	3	2	252
		Malanil	1	4.2	36	13	86.2	31.2	36.2	6.7	50	41	6	3	59
		Malanil	2	4	34.8	12.5	86.1	30.9	35.8	3.1	59	35	5	1	23
		Malanil	3	4.1	35	12.5	85.4	30.5	35.7	3.7	50	46	3	1	39
		Malanil	7	3.8	33.2	7.7	87.2	20.2	23.1	4	40	54	4	2	156
		Malanil	14	3.7	32.7	11.5	88.8	31.1	35.1	5.6	50	45	3	2	154
		Malanil	1	4.8	40.8	14.3	85.6	30	35	9.3	75	20	3	2	153
		Malanil	2	4.3	36.5	13	84.6	30.1	35.5	5.1	63	28	6	3	77
		Malanil	3	4.6	38.6	13.5	84	29.4	35	5.4	46	50	3	1	115
		Malanil	7	4.3	36.2	13.1	84.8	30.6	36	8.5	59	36	3	2	305
		Malanil	14	4.5	38.9	13.3	86.1	29.4	34.1	8.3	60	31	3	6	333
		Sevuparin/DF02+ Malanil	1	5.2	40.8	14.1	78.3	27.2	34.7	5.9	78	18	2	2	71
		Sevuparin/DF02+ Malanil	2	4.9	38.1	13.2	78	27	34.6	3.8	63	30	5	2	26
		Sevuparin/DF02+ Malanil	3	5	39	13.5	77.8	27	34.7	4.1	51	40	8	1	63
		Sevuparin/DF02+ Malanil	7	5.1	39.2	13.3	77.6	26.3	33.9	5.2	53	38	3	6	155
		Sevuparin/DF02+ Malanil	14	4.9	38.8	13.1	79.7	27	33.9	5.4	57	35	2	6	196
		Malanil	1	5.7	46.5	16.4	81.9	28.9	35.3	3	71	22	5	2	65
		Malanil	2	5.6	45.6	16.2	81.4	29	35.6	4.6	49	45	3	3	65

SUBJID	INITIAL	ARM	DAY	RBC ($10^6/\mu L$) 99=Not avalible	HCT (%) 99=Not avalible	HB (g/dL) 99=Not avalible	MCV (fL) 999=Not avalible	MCH (pg/cell) 999=Not avalible	MCHC (pg/cell) 999=Not avalible	WBC ($10^3/\mu L$) 99=Not avalible	NEU (%) 999=Not avalible	LYM (%) 999=Not avalible	MON (%) 999=Not avalible	EOS (%) 999=Not avalible	PLT ($10^3/\mu L$) 999=Not avalible
		Malanil	3	5	40.4	14.1	81.3	28.3	34.9	3.3	49	45	4	2	53
		Malanil	7	4.6	38	13.1	83	28.6	34.5	4	52	42	4	2	157
		Malanil	14	4.7	38.4	13.2	82	28.2	34.4	7.4	50	44	3	3	300
		Malanil	1	5.5	31.6	9.7	57.6	17.8	30.8	6.5	61	30	5	4	105
		Malanil	2	5.3	29.9	9.2	56.9	17.4	30.6	4.2	61	30	4	5	79
		Malanil	3	5	28.6	9	56.8	17.9	31.5	4.2	49	40	4	7	84
		Malanil	7	5.1	29.9	9.2	58.7	18	30.6	7.6	56	36	3	5	156
		Malanil	14	5.2	31.3	9.7	60.3	18.7	31	6.4	50	42	4	4	172
		Malanil	1	4	33.6	11.7	85.4	29.8	34.9	4	69	25	4	2	51
		Malanil	2	3.6	30.6	10.8	84.1	29.7	35.3	3.1	68	25	4	3	45
		Malanil	3	4.1	34.7	11.9	85	29.2	34.4	4.4	62	30	5	3	52
		Malanil	7	3.6	30.4	10.6	85.2	29.8	35	6.4	58	30	5	7	118
		Malanil	14	3.8	33.7	11.6	87.8	30.3	34.5	5.1	58	30	3	9	214
		Sevuparin/DF02+ Malanil	1	4	36.1	13	89.7	32.2	36	6.1	85	10	3	2	59
		Sevuparin/DF02+ Malanil	2	3.7	32.6	12	88	32.5	36.9	5.8	63	25	9	3	38
		Sevuparin/DF02+ Malanil	3	3.7	32.6	11.9	88.8	32.5	36.6	4.3	46	48	4	2	57
		Sevuparin/DF02+ Malanil	7	3.1	28	10.1	91	32.7	36	3.7	60	30	3	7	186
		Sevuparin/DF02+ Malanil	14	3.5	31.5	10.9	91.3	31.5	34.5	4.2	59	30	5	6	220
		Sevuparin/DF02+	1	5.4	42.6	14.5	79.7	27.2	34.1	5	55	38	2	5	192

SUBJID	INITIAL	ARM	DAY	RBC (10^6/µL) 99=Not avalible	HCT (%) 99=Not avalible	HB (g/dL) 99=Not avalible	MCV (fL) 999=Not avalible	MCH (pg/cell) 999=Not avalible	MCHC (pg/cell) 999=Not avalible	WBC (10^3/ µL) 99=Not avalible	NEU (%) 999=Not avalible	LYM (%) 999=Not avalible	MON (%) 999=Not avalible	EOS (%) 999=Not avalible	PLT (10^3/ µL) 999=Not avalible
		Malanil													
		Sevuparin/DF02+ Malanil	2	5.3	43.6	14.2	83	27	32.6	4.9	62	25	4	9	184
		Sevuparin/DF02+ Malanil	3	5.1	41.8	14.2	82.2	28	34.1	5.2	53	36	4	7	223
		Sevuparin/DF02+ Malanil	7	5.1	42.3	14.6	82.5	28.5	34.6	4.8	50	38	2	10	246
		Sevuparin/DF02+ Malanil	14	5.2	42	15	80.4	28.8	35.8	6.6	57	30	4	9	276
		Sevuparin/DF02+ Malanil	1	4.3	32.3	11.1	75.5	26	34.4	6.8	72	20	5	3	103
		Sevuparin/DF02+ Malanil	2	4.1	30.5	10.7	74.8	26.2	35	6.1	58	33	8	1	85
		Sevuparin/DF02+ Malanil	3	4.1	30	10.6	73.9	25.9	35.1	5.9	51	37	5	7	90
		Sevuparin/DF02+ Malanil	7	4.1	30.8	10.4	74.7	25.1	33.6	9.2	57	36	4	3	237
		Sevuparin/DF02+ Malanil	14	4.3	33.1	11.1	77.7	26.1	33.6	8.8	66	30	2	2	282
		Sevuparin/DF02+ Malanil	1	4.7	39.2	13.7	83.4	29	34.8	6.4	76	17	4	3	79
		Sevuparin/DF02+ Malanil	2	4.2	35	12	82.6	28.3	34.3	3.8	65	26	8	1	79
		Sevuparin/DF02+ Malanil	3	4.6	37.7	13	82.4	28.5	34.6	3.9	46	48	4	2	83
		Sevuparin/DF02+	7	4.1	33.8	12	81.7	28.9	35.4	4.3	66	30	3	1	280

SUBJID	INITIAL	ARM	DAY	RBC ($10^6/\mu L$) 99=Not avalible	HCT (%) 99=Not avalible	HB (g/dL) 99=Not avalible	MCV (fL) 999=Not avalible	MCH (pg/cell) 999=Not avalible	MCHC (pg/cell) 999=Not avalible	WBC ($10^3/\mu L$) 99=Not avalible	NEU (%) 999=Not avalible	LYM (%) 999=Not avalible	MON (%) 999=Not avalible	EOS (%) 999=Not avalible	PLT ($10^3/\mu L$) 999=Not avalible
		Malanil													
		Sevuparin/DF02+ Malanil	14	4.9	40.6	13.3	83	27.2	32.8	6.4	57	35	6	2	372
		Sevuparin/DF02+ Malanil	1	4.5	38.8	13.1	85.4	28.8	33.8	3.8	58	38	3	1	144
		Sevuparin/DF02+ Malanil	2	4	32.7	11.7	82.1	29.2	35.6	2.3	48	45	4	3	121
		Sevuparin/DF02+ Malanil	3	4	33.5	11.4	83.2	28.4	34.1	2.9	40	53	5	2	121
		Sevuparin/DF02+ Malanil	7	4.1	34.1	11.7	83.8	28.7	34.2	3.2	51	40	3	6	198
		Sevuparin/DF02+ Malanil	14	4.2	35.2	11.6	84.7	27.9	32.9	6.3	58	34	5	3	248
		Malanil	1	5.4	42	14	78.6	26.1	33.2	6.4	79	16	4	1	74
		Malanil	2	5.8	44.5	14.9	77.1	25.9	33.5	4.4	60	35	4	1	98
		Malanil	3	5.2	39.9	13.4	76.5	25.8	33.7	5.3	66	30	4	0	104
		Malanil	7	4.8	36.6	11.9	76.9	25	32.5	5.6	65	30	4	1	310
		Malanil	14	4.7	36.6	11.9	77.5	25.3	32.6	6.9	67	30	2	1	327
		Malanil	1	5.5	46.2	16.1	84.2	29.4	34.9	5.2	85	12	2	1	56
		Malanil	2	4.9	40.7	14.5	82.4	29.4	35.7	7.4	62	34	3	1	77
		Malanil	3	4.7	38.5	14.2	82	30.2	36.8	6.9	59	34	6	1	82
		Malanil	7	4.3	35.8	12.1	83.7	28.4	33.9	7	63	32	4	1	289
		Malanil	14	4.5	37.7	12.7	84.1	28.3	33.7	8.5	61	35	3	1	297
		Malanil	1	4.3	38.3	13.6	88.5	31.4	35.5	5.9	83	12	3	2	55

SUBJID	INITIAL	ARM	DAY	RBC (10^6/µL) *99=Not avalible*	HCT (%) *99=Not avalible*	HB (g/dL) *99=Not avalible*	MCV (fL) *999=Not avalible*	MCH (pg/cell) *999=Not avalible*	MCHC (pg/cell) *999=Not avalible*	WBC (10^3/ µL) *99=Not avalible*	NEU (%) *999=Not avalible*	LYM (%) *999=Not avalible*	MON (%) *999=Not avalible*	EOS (%) *999=Not avalible*	PLT (10^3/ µL) *999=Not avalible*
		Malanil	2	3.8	33.4	11.7	87.4	30.5	34.9	5.4	59	30	6	5	42
		Malanil	3	3.7	32.3	11.4	86.7	30.6	35.4	5	67	25	5	3	56
		Malanil	7	3.2	29.3	9.9	91.4	30.9	33.8	9.6	59	30	5	6	194
		Malanil	14	3.4	31.2	10.8	92.1	31.9	34.6	8	63	29	2	6	213

16.2.8.1 Biochemistry

SUBJID	INITIAL	ARM	DAY	GLUC (mg/dL) *999= Not available*	BUN (mg/dL) *999= Not available*	CREAT (mg/dL) *99= Not available*	BILI (mg/dL) *99= Not available*	AST (U/L) *9999= Not available*	ALT (U/L) *9999= Not available*	ALP (U/L) *9999= Not available*	SODIUM (mmol/L) *999= Not available*	K (mmol/L) *99= Not available*	CL (mmol/L) *999= Not available*	CO2 (mmol/L) *999= Not available*	CA (mmol/ L) *99= Not available*	CK (U/L) *9999= Not available*	LDH (U/L) *9999= Not available*
		Malanil	1	103	12.2	0.8	1.4	42	46	91	130.4	3.5	101	25.4	11	87	226
		Malanil	2	121	10.5	0.9	1.9	35	39	71	130.1	3.4	105.4	25.4	10	44	202
		Malanil	3	125	11.9	0.6	1.5	33	39	73	130.5	4.5	100.9	26.2	10.9	40	216
		Sevuparin/DF02+ Malanil	1	157	5	0.9	0.7	35	21	80	134.2	4	96.2	23.3	11.6	134	138
		Sevuparin/DF02+ Malanil	2	99	5.8	1.1	0.6	30	13	70	136.8	3.6	100.7	23.2	11	70	147
		Sevuparin/DF02+ Malanil	3	80	10.3	0.7	0.4	22	12	63	138.3	3.6	104.1	23.1	10.8	50	150
		Sevuparin/DF02+ Malanil	7	90	12.7	1.1	0.4	28	12	75	143.4	4	105.9	22.1	10.7	222	135
		Sevuparin/DF02+ Malanil	14	136	8.4	1.1	0.4	47	20	63	141.2	4.1	104	23.5	10.8	577	119
		Sevuparin/DF02+ Malanil	1	195	21.8	1.3	1.4	31	21	80	130.3	3.3	92.6	22.6	10.6	132	109
		Sevuparin/DF02+ Malanil	2	165	19	0.9	1	29	21	69	133.7	3.6	102.8	22.4	10.4	130	121
		Sevuparin/DF02+ Malanil	3	121	15.9	1	0.9	33	24	76	134.8	3.8	101.4	22.3	10.6	228	146
		Sevuparin/DF02+ Malanil	7	121	16.5	0.8	0.7	144	171	113	138.2	3.9	101.8	23.2	11.5	71	138
		Sevuparin/DF02+	14	105	13.7	1	0.8	40	68	79	140.2	3.6	101.6	21.4	9	86	92

SUBJID	INITIAL	ARM	DAY	GLUC (mg/dL) *999= Not available*	BUN (mg/dL) *999= Not available*	CREAT (mg/dL) *99= Not available*	BILI (mg/dL) *99= Not available*	AST (U/L) *9999= Not available*	ALT (U/L) *9999= Not available*	ALP (U/L) *9999= Not available*	SODIUM (mmol/L) *999= Not available*	K (mmol/L) *99= Not available*	CL (mmol/L) *999= Not available*	CO2 (mmol/L) *999= Not available*	CA (mmol/ L) *99= Not available*	CK (U/L) *9999= Not available*	LDH (U/L) *9999= Not available*
		Malanil															
		Malanil	1	75	14.6	1	1.9	22	20	73	137.1	3.8	98.7	24.8	10.6	46	180
		Malanil	2	116	10.8	0.9	1.5	29	18	62	135.8	4	102.5	24.5	10.3	45	231
		Malanil	3	142	9	0.7	1.2	37	23	55	135.3	4.1	101	24.8	7.7	48	222
		Malanil	7	85	13.8	1	0.8	32	36	87	137.9	3.9	98.5	22	8.7	53	149
		Malanil	14	93	11.4	0.9	0.8	20	25	59	137.1	4	104.6	22.8	8.1	110	97
		Malanil	1	102	9.6	0.9	2.8	31	58	80	137.9	4.6	101	24.7	11.8	67	283
		Malanil	2	115	13.8	1	1.8	35	50	67	137	3.8	104.1	23.2	8.4	158	284
		Malanil	3	140	16	0.7	1.3	25	48	63	135.9	3.8	103.5	23.6	8.3	97	245
		Malanil	7	94	11.8	0.8	0.8	46	48	85	137.8	3.8	101	22.7	9.2	72	196
		Malanil	14	93	8.1	0.8	1.1	18	23	58	137.3	4	104.9	21.8	9.8	99	139
		Sevuparin/DF02+ Malanil	1	134	21.9	1	1.1	36	21	121	133.8	3.5	102.6	22.1	8.2	148	90
		Sevuparin/DF02+ Malanil	2	112	20.1	0.7	0.9	39	21	125	132.2	3.9	105	23.5	7.7	74	73
		Sevuparin/DF02+ Malanil	3	96	13	0.8	0.9	35	20	146	133.6	3.5	105.2	23.7	7.6	275	74
		Sevuparin/DF02+ Malanil	7	171	12.7	0.9	0.4	176	159	222	136.6	4.4	105.5	22.5	8.6	140	208
		Sevuparin/DF02+ Malanil	14	103	10.5	0.7	0.8	43	39	151	135.8	4.3	105.1	22.8	8.5	124	133
		Malanil	1	97	16.3	1.1	1.8	35	27	62	132	4.2	101.7	24.5	9	47	217
		Malanil	2	94	12.7	0.8	1	31	25	69	133.6	3.9	97.3	27.2	7.9	46	186

**SUBJID**	**INITIAL**	**ARM**	**DAY**	**GLUC** (mg/dL) *999= Not available*	**BUN** (mg/dL) *999= Not available*	**CREAT** (mg/dL) *99= Not available*	**BILI** (mg/dL) *99= Not available*	**AST** (U/L) *9999= Not available*	**ALT** (U/L) *9999= Not available*	**ALP** (U/L) *9999= Not available*	**SODIUM** (mmol/L) *999= Not available*	**K** (mmol/L) *99= Not available*	**CL** (mmol/L) *999= Not available*	**CO2** (mmol/L) *999= Not available*	**CA** (mmol/ L) *99= Not available*	**CK** (U/L) *9999= Not available*	**LDH** (U/L) *9999= Not available*
		Malanil	3	85	10.7	0.9	1.1	32	30	68	138.5	4.2	103.2	24.5	7.7	46	184
		Malanil	7	124	5.8	0.9	0.7	30	36	85	136	4.5	100.5	25.2	8.5	55	130
		Sevuparin/DF02+ Malanil	1	76	25.7	2	0.6	22	21	68	138.1	3.3	107.1	23.6	8.7	68	117
		Sevuparin/DF02+ Malanil	2	123	18.9	2	0.6	16	15	61	138.8	3.5	107.8	21.1	7.5	2	122
		Sevuparin/DF02+ Malanil	3	123	22.7	1.9	0.7	13	12	76	138.7	3.7	103.8	21.1	7.8	38	147
		Sevuparin/DF02+ Malanil	7	86	25.6	2.3	0.8	26	38	97	135.8	4	101.6	22.9	9.2	52	100
		Sevuparin/DF02+ Malanil	14	112	15.4	1.9	0.8	34	34	105	136.1	4.1	102	26.8	9	39	76
		Sevuparin/DF02+ Malanil	1	95	16.7	1	1.2	31	24	147	134.7	3.5	99.4	21.7	8.2	34	310
		Sevuparin/DF02+ Malanil	2	133	8.1	0.7	1	22	17	96	133.3	3.6	105	21.7	7	23	300
		Sevuparin/DF02+ Malanil	3	94	10.8	0.6	1.1	20	16	94	134.1	3.2	102.7	23.4	7.7	15	343
		Sevuparin/DF02+ Malanil	7	93	4.6	0.8	0.6	59	53	94	141.2	3.7	108.6	24.6	8	143	314
		Sevuparin/DF02+ Malanil	14	82	7.4	0.8	0.7	20	17	125	140.8	4.1	101.6	29.1	8.6	90	193
		Malanil	1	109	11.3	0.9	1.2	18	10	122	136.3	3.9	100.8	26.1	8.2	47	132
		Malanil	2	114	11.6	0.9	1.3	21	11	104	134.9	4	103	26.9	7.8	44	168
		Malanil	3	125	13.2	1	1	15	8	102	136.7	4	101.9	23.9	8.1	25	136

SUBJID	INITIAL	ARM	DAY	GLUC (mg/dL) *999= Not available*	BUN (mg/dL) *999= Not available*	CREAT (mg/dL) *99= Not available*	BILI (mg/dL) *99= Not available*	AST (U/L) *9999= Not available*	ALT (U/L) *9999= Not available*	ALP (U/L) *9999= Not available*	SODIUM (mmol/L) *999= Not available*	K (mmol/L) *99= Not available*	CL (mmol/L) *999= Not available*	CO2 (mmol/L) *999= Not available*	CA (mmol/ L) *99= Not available*	CK (U/L) *9999= Not available*	LDH (U/L) *9999= Not available*
		Malanil	7	93	7.8	0.9	0.5	20	13	121	139.7	5.1	103.9	30.7	9	45	109
		Malanil	14	80	9.6	1	1	21	17	120	138.3	4.1	98.8	24.8	8.8	94	123
		Malanil	1	109	11.1	0.9	1.3	28	21	206	137.7	4	100.9	27.4	8.4	129	219
		Malanil	2	102	17.9	0.7	1	21	17	188	136.7	4	99.6	26.5	8.3	77	212
		Malanil	3	96	16.3	0.9	1.1	22	17	190	136.9	4.4	98.8	27	8.2	65	220
		Malanil	7	96	12.5	1	1.1	23	17	159	136.4	4.6	97.4	25.3	9	115	180
		Malanil	14	87	11.5	0.9	0.8	29	21	184	141.6	4	106.1	24.4	8.5	310	309
		Sevuparin/DF02+ Malanil	1	147	12.5	0.9	0.7	29	19	140	138.9	3.9	100.7	26.5	8.6	37	197
		Sevuparin/DF02+ Malanil	2	107	8.3	0.9	0.9	28	13	100	132.7	4.3	101.4	23.3	8	34	296
		Sevuparin/DF02+ Malanil	3	129	11.2	1.1	0.9	33	16	106	137.8	4.1	104.9	25	8.4	32	309
		Sevuparin/DF02+ Malanil	7	94	8.6	0.7	0.7	41	33	106	145.5	3.9	109.4	27	8.3	48	254
		Sevuparin/DF02+ Malanil	1	115	11.8	0.8	2.5	65	41	73	135.4	3.9	90.8	26.1	8.4	62	162
		Sevuparin/DF02+ Malanil	2	153	4.9	0.4	2.5	83	47	60	129.9	3.7	90.2	26.5	7.2	34	202
		Sevuparin/DF02+ Malanil	3	114	12	0.7	1.4	52	44	61	134	3.7	90.4	26	7	26	165

SUBJID	INITIAL	ARM	DAY	GLUC (mg/dL) *999= Not available*	BUN (mg/dL) *999= Not available*	CREAT (mg/dL) *99= Not available*	BILI (mg/dL) *99= Not available*	AST (U/L) *9999= Not available*	ALT (U/L) *9999= Not available*	ALP (U/L) *9999= Not available*	SODIUM (mmol/L) *999= Not available*	K (mmol/L) *99= Not available*	CL (mmol/L) *999= Not available*	CO2 (mmol/L) *999= Not available*	CA (mmol/ L) *99= Not available*	CK (U/L) *9999= Not available*	LDH (U/L) *9999= Not available*
		Sevuparin/DF02+ Malanil	7	95	9.5	0.9	1.2	36	49	101	136.2	3.9	99.5	26.1	8.5	34	145
		Sevuparin/DF02+ Malanil	14	83	11.1	0.9	0.8	36	35	92	140.5	4.3	101.5	25	8.3	111	158
		Malanil	1	149	12.5	1.1	1.2	25	17	84	140.5	4.3	101.8	27.7	8.6	83	116
		Malanil	2	119	10.2	1.2	2.4	43	23	77	135.5	3.6	99.9	24.5	7.8	43	172
		Malanil	3	152	13.5	1	1.4	28	22	76	133.3	3.6	98.2	23.8	7.7	26	183
		Malanil	7	95	11.9	1	0.5	54	55	89	139.5	4.9	97.7	27.2	8.8	52	180
		Malanil	14	77	16.2	1	0.7	21	24	93	140.4	4.4	98.5	27.6	8.9	64	123
		Malanil	1	170	24.5	1.3	2.4	33	27	114	136.6	3.5	98.1	24.4	7.4	33	150
		Malanil	2	138	19.9	0.7	1.9	30	25	85	133.5	3.3	96.9	25.2	7.1	21	185
		Malanil	3	103	15.8	0.9	2.5	33	25	92	137.1	3.6	97.8	26.5	7	16	171
		Malanil	7	90	12.4	0.8	0.6	60	69	111	138.7	3.7	100	28.4	7.1	28	155
		Malanil	14	84	11.9	1	1.5	63	105	131	138.4	4	95.2	27.8	8.3	43	217
		Malanil	1	173	12	0.9	1.2	32	12	269	136.5	3.8	97.2	26.8	8.2	100	91
		Malanil	2	108	11.2	0.9	0.6	33	13	282	139.2	4	100.4	27.1	8.6	66	93
		Malanil	3	131	13.4	0.8	1	33	16	313	138.7	3.8	95.4	27.3	9	70	152
		Malanil	7	83	15.1	1	0.3	32	10	255	145	3.7	104.6	25.9	8.4	99	143
		Malanil	14	112	11.5	0.9	0.8	29	11	226	141.7	5.3	103.2	25.9	9.6	117	127
		Sevuparin/DF02+ Malanil	1	191	6.4	0.8	1.1	37	22	54	135	4.2	104.8	25.8	9	58	227
		Sevuparin/DF02+ Malanil	2	103	7.6	0.7	0.7	20	16	65	137	3.2	106.6	24.8	7.6	31	212

**SUBJID**	**INITIAL**	**ARM**	**DAY**	**GLUC** (mg/dL) *999= Not available*	**BUN** (mg/dL) *999= Not available*	**CREAT** (mg/dL) *99= Not available*	**BILI** (mg/dL) *99= Not available*	**AST** (U/L) *9999= Not available*	**ALT** (U/L) *9999= Not available*	**ALP** (U/L) *9999= Not available*	**SODIUM** (mmol/L) *999= Not available*	**K** (mmol/L) *99= Not available*	**CL** (mmol/L) *999= Not available*	**CO2** (mmol/L) *999= Not available*	**CA** (mmol/ L) *99= Not available*	**CK** (U/L) *9999= Not available*	**LDH** (U/L) *9999= Not available*
		Sevuparin/DF02+ Malanil	3	105	7.7	0.8	0.8	21	16	68	136.8	3.7	106.9	23.3	8.1	27	229
		Sevuparin/DF02+ Malanil	7	104	8.3	0.9	0.6	49	59	60	138.5	4.5	102.6	27.5	9.3	75	200
		Sevuparin/DF02+ Malanil	14	72	11.7	1.2	1.4	42	32	65	135.3	4.5	99.2	21.5	8.7	335	265
		Sevuparin/DF02+ Malanil	1	154	19.4	1.3	1.8	31	42	105	136.3	4.1	98.2	26.4	8.3	43	157
		Sevuparin/DF02+ Malanil	2	158	21.2	1.1	1.1	22	28	85	134.6	3.9	93.5	28.3	7.8	34	148
		Sevuparin/DF02+ Malanil	3	142	14.6	1.2	0.9	20	23	79	134.7	3.8	98.8	25	7.7	24	159
		Sevuparin/DF02+ Malanil	7	115	8.6	1.1	0.9	173	200	99	137.4	4.3	99.5	26.1	7.9	39	198
		Sevuparin/DF02+ Malanil	14	194	7.5	1.1	0.6	41	100	75	139.8	4	104.8	26.7	7.8	49	152
		Sevuparin/DF02+ Malanil	1	143	12.4	0.9	1.4	19	12	45	130.5	3.8	98.1	24	8.4	82	134
		Sevuparin/DF02+ Malanil	2	82	4.8	0.7	0.7	19	12	52	131.7	4.1	102.7	25	7.2	30	136
		Sevuparin/DF02+ Malanil	3	105	7.8	0.7	0.7	13	9	54	129.9	3.1	101.8	24.5	7.5	18	137
		Sevuparin/DF02+ Malanil	7	80	7.1	0.6	0.5	86	105	45	136	3.9	104.9	26	7.5	43	122
		Sevuparin/DF02+ Malanil	14	81	7.4	0.7	0.9	22	35	46	138.5	4.5	102.9	24.4	8.1	62	203

SUBJID	INITIAL	ARM	DAY	GLUC (mg/dL) *999= Not available*	BUN (mg/dL) *999= Not available*	CREAT (mg/dL) *99= Not available*	BILI (mg/dL) *99= Not available*	AST (U/L) *9999= Not available*	ALT (U/L) *9999= Not available*	ALP (U/L) *9999= Not available*	SODIUM (mmol/L) *999= Not available*	K (mmol/L) *99= Not available*	CL (mmol/L) *999= Not available*	CO2 (mmol/L) *999= Not available*	CA (mmol/ L) *99= Not available*	CK (U/L) *9999= Not available*	LDH (U/L) *9999= Not available*
		Malanil	1	85	14.3	1	1	34	25	79	134	4	99	25.5	8.3	192	119
		Malanil	2	87	16	1	1	22	18	70	132.9	3.9	101.9	27.5	8	87	103
		Malanil	3	101	13.5	0.9	0.8	20	15	61	132.3	3.9	100.5	23.6	7.8	57	95
		Malanil	7	85	10	0.9	0.5	27	19	50	136.2	3.7	102.5	23.9	7.4	163	95
		Malanil	14	70	10.4	0.9	0.5	25	22	63	137.9	4.6	103.7	25.9	7.4	179	145
		Sevuparin/DF02+ Malanil	1	89	9.5	1.1	1.7	31	25	74	135.9	4.5	97	23.4	8.3	68	145
		Sevuparin/DF02+ Malanil	2	113	11.6	0.9	1.2	38	28	133	130.8	3.8	98	25.5	7.5	37	193
		Sevuparin/DF02+ Malanil	3	108	10.2	0.8	1.1	22	19	137	132	3.5	95.7	15.5	7.7	22	199
		Sevuparin/DF02+ Malanil	7	92	12.7	0.8	0.7	39	43	145	138.2	4.4	102.7	23.1	8.1	31	147
		Sevuparin/DF02+ Malanil	14	80	10.9	1.1	0.9	21	18	109	139.3	4.3	106.4	25	8	118	176
		Sevuparin/DF02+ Malanil	1	107	12	0.7	1.6	30	35	79	129	3.3	88	24.6	8.3	52	186
		Sevuparin/DF02+ Malanil	2	122	7.5	0.7	1.2	57	33	82	129.6	3.6	95.4	22.3	7.1	27	172
		Sevuparin/DF02+ Malanil	3	93	4.6	0.7	0.8	108	52	123	133.9	3.6	102.1	24.5	7.1	29	193
		Sevuparin/DF02+ Malanil	7	90	4.4	0.8	0.9	52	89	117	144.7	4.4	109.2	21.9	8.1	59	151
		Sevuparin/DF02+ Malanil	14	86	13.4	0.9	0.6	44	29	80	142.1	5.4	105.7	20.6	9	352	262

SUBJID	INITIAL	ARM	DAY	GLUC (mg/dL) *999= Not available*	BUN (mg/dL) *999= Not available*	CREAT (mg/dL) *99= Not available*	BILI (mg/dL) *99= Not available*	AST (U/L) *9999= Not available*	ALT (U/L) *9999= Not available*	ALP (U/L) *9999= Not available*	SODIUM (mmol/L) *999= Not available*	K (mmol/L) *99= Not available*	CL (mmol/L) *999= Not available*	CO2 (mmol/L) *999= Not available*	CA (mmol/ L) *99= Not available*	CK (U/L) *9999= Not available*	LDH (U/L) *9999= Not available*
		Sevuparin/DF02+ Malanil	1	127	12.8	0.9	2	45	52	124	137.2	3.1	100.4	22.4	7.8	118	173
		Sevuparin/DF02+ Malanil	2	127	11.8	0.7	1.1	41	51	103	136.4	3.9	97	24.7	7	46	169
		Sevuparin/DF02+ Malanil	3	108	12.9	0.7	0.8	34	43	95	136.1	4	100.2	23	7.4	30	142
		Sevuparin/DF02+ Malanil	7	88	12.3	0.7	3.4	32	35	70	137.7	4.1	105.9	22.2	7.5	67	261
		Sevuparin/DF02+ Malanil	14	100	9.3	0.8	1.5	27	24	80	139.6	4.3	107.1	18.5	8.6	81	329
		Sevuparin/DF02+ Malanil	1	123	18.1	1	1.4	21	19	85	136.2	3.3	102.8	26.2	7.9	85	144
		Sevuparin/DF02+ Malanil	2	108	14	0.8	1.1	21	17	70	132.6	4	100.3	21.3	6.6	41	194
		Sevuparin/DF02+ Malanil	3	114	14.5	0.8	0.8	17	15	68	132.6	3.3	100.8	21.9	7	27	220
		Sevuparin/DF02+ Malanil	7	101	8.5	1	0.6	28	24	67	136.1	4.5	100.7	26.4	8.5	44	216
		Sevuparin/DF02+ Malanil	14	143	5.8	0.6	0.6	33	32	81	137.2	4	103.8	20	8.3	96	232
		Malanil	1	139	6	1	1	50	36	57	139.3	2.7	105.4	22.7	8.1	121	212
		Malanil	2	153	6.6	0.8	1.3	29	31	55	137.5	2.6	103.4	20.8	8	59	308
		Malanil	3	139	10.5	0.9	1	17	23	54	138.4	3.7	105.2	26.3	8.6	40	182
		Malanil	7	122	7.7	0.7	0.8	13	15	45	141.4	3.1	104.7	25.9	8.4	54	149
		Malanil	14	114	4.3	0.6	0.6	14	9	46	142.5	3.3	105.7	26.1	7.5	88	144

**SUBJID**	**INITIAL**	**ARM**	**DAY**	**GLUC** (mg/dL) *999= Not available*	**BUN** (mg/dL) *999= Not available*	**CREAT** (mg/dL) *99= Not available*	**BILI** (mg/dL) *99= Not available*	**AST** (U/L) *9999= Not available*	**ALT** (U/L) *9999= Not available*	**ALP** (U/L) *9999= Not available*	**SODIUM** (mmol/L) *999= Not available*	**K** (mmol/L) *99= Not available*	**CL** (mmol/L) *999= Not available*	**CO2** (mmol/L) *999= Not available*	**CA** (mmol/ L) *99= Not available*	**CK** (U/L) *9999= Not available*	**LDH** (U/L) *9999= Not available*
		Malanil	1	119	14.5	1.1	1.5	21	17	53	138.7	4	104.8	22.3	8.4	94	143
		Malanil	2	188	10.5	1.1	1.6	25	17	47	135.7	3.8	100.9	25.5	8.2	61	248
		Malanil	3	124	14.9	1.1	1.1	16	17	47	134.5	4	103.4	21.6	8.4	31	173
		Malanil	7	91	11.6	0.7	0.5	20	23	47	139.1	4.4	105.6	22	8.8	112	158
		Malanil	14	100	7.8	1	0.5	20	16	49	140.8	4	104.6	27	8.1	191	130
		Malanil	1	121	10.8	1.1	3.1	30	19	88	134.7	3.4	102.5	21.5	8.3	70	195
		Malanil	2	162	17	1	1.9	56	58	96	135.8	3.6	104.2	21.5	8.2	41	204
		Malanil	3	149	13.5	1.1	1.9	49	65	102	134.8	3.9	103.2	23.8	8.1	30	164
		Malanil	7	107	6.8	0.7	1.5	44	84	127	135.7	3.6	103.3	21.2	8.4	83	167
		Malanil	14	109	9.2	0.7	0.9	37	33	124	140.5	3.4	102.2	21.8	7.9	110	204
		Sevuparin/DF02+ Malanil	1	131	11	1.1	2.2	33	32	44	130.7	3.4	97.9	28.9	8	44	308
		Sevuparin/DF02+ Malanil	2	153	10.7	0.9	1.3	39	35	36	134.4	3.3	97.7	30.8	7.3	27	281
		Sevuparin/DF02+ Malanil	3	127	7.7	0.9	1.1	78	62	57	134.5	3.5	101.3	27.8	7.3	31	358
		Sevuparin/DF02+ Malanil	7	104	7.3	0.9	0.8	451	391	138	138.8	4	103.5	24.2	7.9	83	431
		Sevuparin/DF02+ Malanil	14	100	9.5	0.9	2	45	380	208	137.2	4.5	105.9	22	7.7	92	521
		Malanil	1	156	17.2	1.5	3	24	18	52	129.5	3.2	90.1	23.6	8.4	154	196
		Malanil	2	148	16.6	1.2	2.2	14	17	68	128.1	3.1	92.4	24.8	8.2	66	245
		Malanil	3	147	20.3	1.1	2.2	16	16	63	132.8	3.1	90.7	25.2	8.1	33	216

**SUBJID**	**INITIAL**	**ARM**	**DAY**	**GLUC** (mg/dL) *999= Not available*	**BUN** (mg/dL) *999= Not available*	**CREAT** (mg/dL) *99= Not available*	**BILI** (mg/dL) *99= Not available*	**AST** (U/L) *9999= Not available*	**ALT** (U/L) *9999= Not available*	**ALP** (U/L) *9999= Not available*	**SODIUM** (mmol/L) *999= Not available*	**K** (mmol/L) *99= Not available*	**CL** (mmol/L) *999= Not available*	**CO2** (mmol/L) *999= Not available*	**CA** (mmol/ L) *99= Not available*	**CK** (U/L) *9999= Not available*	**LDH** (U/L) *9999= Not available*
		Malanil	7	119	15.1	1	0.9	20	25	56	135.7	4	97.6	29.6	8	52	212
		Malanil	14	106	8.6	1.1	1.1	19	29	61	139.1	4.4	105.6	29	8.1	114	228
		Malanil	1	215	13.2	1.1	1.2	19	14	55	135.4	4.3	100.8	25.9	8.2	63	112
		Malanil	2	175	14.6	0.9	1.3	17	11	46	131.6	3.7	99.6	22.2	7.7	40	117
		Malanil	3	149	6.8	1	1	18	12	45	135.7	3.8	100.4	23.2	8.1	39	146
		Malanil	7	103	15.5	0.8	0.6	14	12	56	138.9	4	103.1	27.3	8	81	131
		Malanil	14	84	10.4	0.9	0.8	18	9	57	140.6	3.8	105	24.1	8.2	106	152
		Malanil	1	119	8.5	0.8	1.6	79	82	64	138.4	3.5	100	26.3	7.7	298	196
		Malanil	2	180	6.5	0.9	1.6	78	65	58	131.1	3.1	98.6	27.6	7.8	117	273
		Malanil	3	116	8.8	0.7	1.4	71	59	57	135.2	3.5	102.6	21.8	7.7	50	258
		Malanil	7	107	4.8	0.8	1.1	143	78	66	145.3	3.6	108	20.2	7.5	112	287
		Malanil	14	100	13	0.8	1.1	160	73	45	143.6	3.8	106.1	26.8	8	125	245
		Malanil	1	123	12	1.1	1.6	22	22	83	137.9	3.7	102.4	23.2	8.1	93	131
		Malanil	2	166	10.1	1.2	1.5	17	19	73	133	3.6	103.1	20.5	7.9	46	188
		Malanil	3	130	11.8	1	1.3	18	22	73	136	3.9	102.7	21.2	8.3	35	177
		Malanil	7	99	10.4	0.9	1.5	27	42	77	142.4	3.9	105.4	19.9	8.4	106	165
		Malanil	14	79	8.6	1.1	0.8	20	28	54	138	4.4	103.3	26.4	8.6	114	120
		Sevuparin/DF02+	1	161	17	0.9	2	11	10	132	132.9	4.1	100.6	21.5	7.9	50	151

SUBJID	INITIAL	ARM	DAY	GLUC (mg/dL) *999= Not available*	BUN (mg/dL) *999= Not available*	CREAT (mg/dL) *99= Not available*	BILI (mg/dL) *99= Not available*	AST (U/L) *9999= Not available*	ALT (U/L) *9999= Not available*	ALP (U/L) *9999= Not available*	SODIUM (mmol/L) *999= Not available*	K (mmol/L) *99= Not available*	CL (mmol/L) *999= Not available*	CO2 (mmol/L) *999= Not available*	CA (mmol/ L) *99= Not available*	CK (U/L) *9999= Not available*	LDH (U/L) *9999= Not available*
		Malanil															
		Sevuparin/DF02+ Malanil	2	143	15.3	1	1.6	18	11	100	126.3	3.6	98.2	19.8	7.4	23	246
		Sevuparin/DF02+ Malanil	3	97	16.7	0.8	1	14	10	91	132.8	4	101	18.8	7.8	15	202
		Sevuparin/DF02+ Malanil	7	76	10	0.8	0.8	25	29	97	141.4	4	104.4	19.7	8.3	42	163
		Sevuparin/DF02+ Malanil	14	82	9.3	0.9	0.5	17	15	78	138.1	4.2	103	26.9	8.3	79	234
		Malanil	1	187	15.3	1.1	2.7	19	20	84	136.7	3.8	100.6	25.5	8.4	82	188
		Malanil	2	81	21.2	0.9	1.7	19	18	75	136.5	3.9	102	18.4	8.3	45	174
		Malanil	3	130	18	0.9	1.6	14	13	56	132.8	3.8	104.2	26.2	7.5	36	192
		Malanil	7	84	6.9	0.8	1.2	22	23	66	141	3.6	105.9	20.2	7.8	62	170
		Malanil	14	89	14.8	1	1.5	25	29	54	139.3	4.2	103.3	25.4	7.9	116	248
		Malanil	1	98	15	0.8	1.4	45	39	79	135.5	3.5	104.5	27.4	7.4	180	194
		Malanil	2	141	6	0.9	1.3	36	38	100	135.3	3.5	105	22	7.7	85	193
		Malanil	3	99	17.7	0.8	1.1	29	34	97	137.3	3.7	106.1	20.1	7.6	63	168
		Malanil	7	91	10.5	0.8	1.2	27	26	82	139.9	3.9	106.4	23	7.6	111	188
		Malanil	14	149	17.5	0.8	1.7	29	20	89	137.3	3.6	103.1	23.4	7.7	114	198
		Malanil	1	120	11.7	0.9	0.9	45	29	56	135	2.7	104.3	24.7	7.4	138	220
		Malanil	2	129	11.2	0.9	0.7	35	28	62	135.1	3.1	104.2	26.2	6.7	93	208
		Malanil	3	127	7.5	1	0.7	49	44	87	140.1	3	105.8	25.3	7.5	83	298
		Malanil	7	107	8.9	1	0.6	41	40	65	139.2	3.6	103.8	28.7	7	178	306

SUBJID	INITIAL	ARM	DAY	GLUC (mg/dL) *999= Not available*	BUN (mg/dL) *999= Not available*	CREAT (mg/dL) *99= Not available*	BILI (mg/dL) *99= Not available*	AST (U/L) *9999= Not available*	ALT (U/L) *9999= Not available*	ALP (U/L) *9999= Not available*	SODIUM (mmol/L) *999= Not available*	K (mmol/L) *99= Not available*	CL (mmol/L) *999= Not available*	CO2 (mmol/L) *999= Not available*	CA (mmol/ L) *99= Not available*	CK (U/L) *9999= Not available*	LDH (U/L) *9999= Not available*
		Malanil	14	102	4.1	1.1	0.9	29	25	81	138.5	4.4	101.2	24.1	8	157	298
		Sevuparin/DF02+ Malanil	1	144	18.5	1.2	5.7	27	24	60	125.9	3.3	93.9	24.1	7.9	122	161
		Sevuparin/DF02+ Malanil	2	90	15.2	0.8	2.4	24	22	48	136	3.1	98.9	20.4	6.9	55	160
		Sevuparin/DF02+ Malanil	3	98	11.9	1	2.9	22	22	59	137.4	3.2	96.8	22.9	7.2	34	186
		Sevuparin/DF02+ Malanil	7	106	7.2	0.5	1.1	23	26	56	140.9	3.7	105.1	24	7.4	47	140
		Sevuparin/DF02+ Malanil	14	113	9.2	0.9	1	22	21	52	140.5	3.8	108.4	24.3	8.3	61	150
		Sevuparin/DF02+ Malanil	1	107	9.2	0.9	0.6	18	10	128	138.5	3.7	103.8	23.8	9	117	133
		Sevuparin/DF02+ Malanil	2	107	8.5	0.7	0.5	16	11	126	138	3.4	100.5	25.4	9	54	138
		Sevuparin/DF02+ Malanil	3	86	8.6	0.9	0.7	17	18	119	137.8	3.7	102.5	23.5	9.1	43	132
		Sevuparin/DF02+ Malanil	7	103	11.7	0.9	0.7	69	82	87	137.8	4	100.3	23.1	9.5	118	175
		Sevuparin/DF02+ Malanil	14	130	8.9	0.9	0.7	24	20	90	141.7	3.8	103.1	26.2	9.2	189	112
		Sevuparin/DF02+ Malanil	1	128	16.7	1	1.2	26	41	123	138	3.9	104.4	25.9	8	66	166
		Sevuparin/DF02+ Malanil	2	124	13.3	0.9	1.4	17	31	122	135.5	3.8	103.2	28.1	8.1	35	197
		Sevuparin/DF02+	3	108	15	0.9	1.2	17	26	120	134.3	3.7	106.2	26.3	8.6	21	210

SUBJID	INITIAL	ARM	DAY	GLUC (mg/dL) *999= Not available*	BUN (mg/dL) *999= Not available*	CREAT (mg/dL) *99= Not available*	BILI (mg/dL) *99= Not available*	AST (U/L) *9999= Not available*	ALT (U/L) *9999= Not available*	ALP (U/L) *9999= Not available*	SODIUM (mmol/L) *999= Not available*	K (mmol/L) *99= Not available*	CL (mmol/L) *999= Not available*	CO2 (mmol/L) *999= Not available*	CA (mmol/ L) *99= Not available*	CK (U/L) *9999= Not available*	LDH (U/L) *9999= Not available*
		Malanil															
		Sevuparin/DF02+ Malanil	7	97	13.6	1	1.5	109	276	147	140.5	4.9	105.4	29.7	9.5	65	197
		Sevuparin/DF02+ Malanil	14	100	9	0.9	1	32	95	101	138.4	4.1	106.7	26.3	8.9	66	245
		Sevuparin/DF02+ Malanil	1	94	13.2	1.3	2.3	33	27	77	129.8	3.6	95.7	28.1	9.1	89	213
		Sevuparin/DF02+ Malanil	2	138	14.9	1.1	1.4	24	24	79	129.3	3.3	101.3	26.6	8.5	59	221
		Sevuparin/DF02+ Malanil	3	115	11.7	0.9	1.1	22	26	87	133.4	3.9	101.1	31.9	8.3	30	222
		Sevuparin/DF02+ Malanil	7	101	6.4	1	0.9	51	65	65	136.7	3.4	107.9	27	8.9	71	180
		Sevuparin/DF02+ Malanil	14	97	12.8	1.1	1.4	30	26	71	141.2	4.2	100.1	28.3	10.1	48	209
		Sevuparin/DF02+ Malanil	1	109	14.9	1.1	0.8	25	25	66	132.3	3.5	102.4	26.7	9.2	85	159
		Sevuparin/DF02+ Malanil	2	131	12.7	1	0.8	21	21	61	129.6	3.5	101.2	25.7	8.7	45	157
		Sevuparin/DF02+ Malanil	3	93	12.3	0.9	0.9	16	19	59	132.3	3.5	102.6	26.3	8.8	36	161
		Sevuparin/DF02+ Malanil	7	116	8	1	0.6	99	75	69	135	4.4	103	28.1	8.4	71	194
		Sevuparin/DF02+ Malanil	14	92	10	0.9	0.6	29	39	57	139.8	4.1	103.2	26.9	9.1	100	129
		Malanil	1	118	22	1.3	1.3	34	26	108	133.6	3.8	96.6	25.4	9.2	144	149

SUBJID	INITIAL	ARM	DAY	GLUC (mg/dL) *999= Not available*	BUN (mg/dL) *999= Not available*	CREAT (mg/dL) *99= Not available*	BILI (mg/dL) *99= Not available*	AST (U/L) *9999= Not available*	ALT (U/L) *9999= Not available*	ALP (U/L) *9999= Not available*	SODIUM (mmol/L) *999= Not available*	K (mmol/L) *99= Not available*	CL (mmol/L) *999= Not available*	CO2 (mmol/L) *999= Not available*	CA (mmol/ L) *99= Not available*	CK (U/L) *9999= Not available*	LDH (U/L) *9999= Not available*
		Malanil	2	99	11.9	0.9	0.9	36	32	88	132	3.8	101.9	26.8	8.4	13	210
		Malanil	3	78	16.3	0.9	1.3	25	26	93	131.2	4.1	99.9	24.9	8.7	53	192
		Malanil	7	96	12.2	0.9	0.5	28	30	73	139	4.4	100.3	30.7	8.5	53	143
		Malanil	14	90	9.8	0.9	0.5	18	19	92	137.6	4.3	99.4	27.2	9.1	85	146
		Malanil	1	99	22.1	1.2	3.4	45	50	152	133.4	3.7	100.6	22.4	8.7	48	268
		Malanil	2	101	20	0.9	2	44	52	109	128.4	3.5	98	22.9	8.4	36	325
		Malanil	3	115	18.8	0.3	2.2	44	45	151	130.1	4	100.1	25.9	8.4	26	382
		Malanil	7	81	11	1	0.7	112	214	331	141.2	4.3	101.6	28.2	8.4	41	264
		Malanil	14	98	10.8	0.9	1.1	38	80	224	138.3	4.2	99.1	28.3	8.5	80	286
		Malanil	1	96	14.9	0.8	5.1	29	18	80	132.9	3.8	99	25.7	8.5	50	105
		Malanil	2	125	20.1	0.9	4.5	23	15	66	135.5	3.2	95.2	25.5	7.9	36	97
		Malanil	3	90	18.3	0.8	4.1	27	14	61	133.3	3.9	89.1	26.8	8.4	36	436
		Malanil	7	77	9.5	0.9	1.6	24	18	85	139.2	3.5	103.4	30.4	8.1	52	256
		Malanil	14	105	10.5	1	1.4	21	16	107	137	3.8	102.5	25.5	8.3	95	209

16.2.8.2 Coagulation tests

SUBJID	INITIAL	ARM	TIMEPOINT	APTT	PT	INR
		Sevuparin/DF02+ Malanil	screening	30.6	11.3	0.98
		Sevuparin/DF02+ Malanil	pre-dose	30.6	11.3	0.98
		Sevuparin/DF02+ Malanil	D1H2	40.2	11.7	1.02
		Sevuparin/DF02+ Malanil	D1H5	30.6	11.7	1.02
		Sevuparin/DF02+ Malanil	D1H11	31.8	11.2	0.97
		Sevuparin/DF02+ Malanil	D1H17	32.8	11	0.96
		Sevuparin/DF02+ Malanil	D1H23	30.3	10.6	0.92
		Sevuparin/DF02+ Malanil	D2H29	33	11.4	0.99
		Sevuparin/DF02+ Malanil	D2H32	45.7	11.2	0.97
		Sevuparin/DF02+ Malanil	D2H35	31.6	10.3	0.9
		Sevuparin/DF02+ Malanil	D2H41	36.3	10.1	0.88
		Sevuparin/DF02+ Malanil	D2H47	34.3	11.6	1.01
		Sevuparin/DF02+ Malanil	D3H53	33.2	11.8	1.03
		Sevuparin/DF02+ Malanil	D3H59	37.4	13.5	1.17
		Sevuparin/DF02+ Malanil	D3H65	33.6	11.7	1.02
		Sevuparin/DF02+ Malanil	D3H68	40.9	11.6	1.01
		Sevuparin/DF02+ Malanil	D3H71	33.5	11.3	0.98
		Sevuparin/DF02+ Malanil	D7	25.8	11.6	1.01
		Sevuparin/DF02+ Malanil	D14	27.5	11.6	1.01
		Sevuparin/DF02+ Malanil	screening	31.2	14.9	1.3
		Sevuparin/DF02+ Malanil	pre-dose	31.2	14.9	1.3

SUBJID	INITIAL	ARM	TIMEPOINT	APTT	PT	INR
		Sevuparin/DF02+ Malanil	D1H2	48	16.3	1.42
		Sevuparin/DF02+ Malanil	D1H5	34.1	13.9	1.21
		Sevuparin/DF02+ Malanil	D1H11	38.4	15.2	1.32
		Sevuparin/DF02+ Malanil	D1H17	38.2	13.9	1.21
		Sevuparin/DF02+ Malanil	D1H23	40.6	13.5	1.17
		Sevuparin/DF02+ Malanil	D2H29	39.6	13.6	1.18
		Sevuparin/DF02+ Malanil	D2H32	48.7	14.4	1.25
		Sevuparin/DF02+ Malanil	D2H35	41.1	14.2	1.23
		Sevuparin/DF02+ Malanil	D2H41	41.4	14.2	1.23
		Sevuparin/DF02+ Malanil	D2H47	35.7	13.8	1.2
		Sevuparin/DF02+ Malanil	D3H53	39.5	14.3	1.24
		Sevuparin/DF02+ Malanil	D3H59	39.5	13.9	1.21
		Sevuparin/DF02+ Malanil	D3H65	35.2	14.4	1.25
		Sevuparin/DF02+ Malanil	D3H68	45.8	14.1	1.23
		Sevuparin/DF02+ Malanil	D3H71	39.3	13.7	1.19
		Sevuparin/DF02+ Malanil	D7	30.4	15	1.3
		Sevuparin/DF02+ Malanil	D14	29.8	13.1	1.14
		Sevuparin/DF02+ Malanil	screening	28.5	13.2	1.15
		Sevuparin/DF02+ Malanil	pre-dose	28.5	13.2	1.15
		Sevuparin/DF02+ Malanil	D1H2	37.9	13.6	1.18
		Sevuparin/DF02+ Malanil	D1H5	30.3	14.7	1.28
		Sevuparin/DF02+ Malanil	D1H11	31.8	13.5	1.17
		Sevuparin/DF02+ Malanil	D1H17	28.6	12.1	1.05

SUBJID	INITIAL	ARM	TIMEPOINT	APTT	PT	INR
		Sevuparin/DF02+ Malanil	D1H23	35	11.4	0.99
		Sevuparin/DF02+ Malanil	D2H29	29.5	13.3	1.16
		Sevuparin/DF02+ Malanil	D2H32	43.5	11.9	1.03
		Sevuparin/DF02+ Malanil	D2H35	29.6	12.7	1.1
		Sevuparin/DF02+ Malanil	D2H41	29.6	12	1.04
		Sevuparin/DF02+ Malanil	D2H47	30.2	12.7	1.1
		Sevuparin/DF02+ Malanil	D3H53	27.3	13.6	1.18
		Sevuparin/DF02+ Malanil	D3H59	32.9	13.4	1.17
		Sevuparin/DF02+ Malanil	D3H65	34.4	13.1	1.14
		Sevuparin/DF02+ Malanil	D3H68	42.9	14.3	1.24
		Sevuparin/DF02+ Malanil	D3H71	29.1	11.8	1.03
		Sevuparin/DF02+ Malanil	D7	28.7	12.9	1.12
		Sevuparin/DF02+ Malanil	D14	25.9	10.9	0.91
		Sevuparin/DF02+ Malanil	screening	28.8	12.6	1.05
		Sevuparin/DF02+ Malanil	pre-dose	28.8	12.6	1.05
		Sevuparin/DF02+ Malanil	D1H2	42.5	13.5	1.13
		Sevuparin/DF02+ Malanil	D1H5	33.5	13.9	1.16
		Sevuparin/DF02+ Malanil	D1H11	38.6	15.3	1.26
		Sevuparin/DF02+ Malanil	D1H17	41.4	14.8	1.22
		Sevuparin/DF02+ Malanil	D1H23	49.4	14.8	1.22
		Sevuparin/DF02+ Malanil	D2H29	48	14.6	1.22
		Sevuparin/DF02+ Malanil	D2H32	70.7	16.2	1.34
		Sevuparin/DF02+ Malanil	D2H35	53.4	13.4	1.12

TSM02 Individual subject listings part 2

CSR Appendix 16.2

SUBJID	INITIAL	ARM	TIMEPOINT	APTT	PT	INR
		Sevuparin/DF02+ Malanil	D2H41	50.3	15.3	1.26
		Sevuparin/DF02+ Malanil	D2H47	48.3	13.8	1.15
		Sevuparin/DF02+ Malanil	D3H53	41.5	14.5	1.21
		Sevuparin/DF02+ Malanil	D3H59	45.1	13.4	1.12
		Sevuparin/DF02+ Malanil	D3H65	45.6	14	1.17
		Sevuparin/DF02+ Malanil	D3H68	68.5	14.2	1.18
		Sevuparin/DF02+ Malanil	D3H71	43.4	13.7	1.14
		Sevuparin/DF02+ Malanil	D7	26.9	12.5	1.04
		Sevuparin/DF02+ Malanil	D14	27	13.2	1.1
		Sevuparin/DF02+ Malanil	screening	30.7	12.9	1.08
		Sevuparin/DF02+ Malanil	pre-dose	30.7	12.9	1.08
		Sevuparin/DF02+ Malanil	D1H2	35	13.6	1.13
		Sevuparin/DF02+ Malanil	D1H5	30.2	14.8	1.22
		Sevuparin/DF02+ Malanil	D1H11	36.2	15	1.24
		Sevuparin/DF02+ Malanil	D1H17	32.4	12.6	1.05
		Sevuparin/DF02+ Malanil	D1H23	33.7	14.3	1.19
		Sevuparin/DF02+ Malanil	D2H29	33.1	12.7	1.06
		Sevuparin/DF02+ Malanil	D2H32	52.2	13.7	1.14
		Sevuparin/DF02+ Malanil	D2H35	36.4	13.4	1.12
		Sevuparin/DF02+ Malanil	D2H41	34.9	12.7	1.06
		Sevuparin/DF02+ Malanil	D2H47	33.8	12.9	1.08
		Sevuparin/DF02+ Malanil	D3H53	34	12.3	1.02
		Sevuparin/DF02+ Malanil	D3H59	34.2	13	1.08

SUBJID	INITIAL	ARM	TIMEPOINT	APTT	PT	INR
		Sevuparin/DF02+ Malanil	D3H65	32.6	12.1	1.01
		Sevuparin/DF02+ Malanil	D3H68	42	13.2	1.1
		Sevuparin/DF02+ Malanil	D3H71	31	12.4	1.03
		Sevuparin/DF02+ Malanil	D7	27.8	13.3	1.11
		Sevuparin/DF02+ Malanil	D14	27.6	12.9	1.08
		Sevuparin/DF02+ Malanil	screening	29.9	13.1	1.09
		Sevuparin/DF02+ Malanil	pre-dose	29.9	13.1	1.09
		Sevuparin/DF02+ Malanil	D1H2	40.6	11.7	0.98
		Sevuparin/DF02+ Malanil	D1H5	33.9	13.5	1.13
		Sevuparin/DF02+ Malanil	D1H11	41.6	14.1	1.18
		Sevuparin/DF02+ Malanil	D1H17	33.2	12.2	1.02
		Sevuparin/DF02+ Malanil	D1H23	38.8	12.8	1.07
		Sevuparin/DF02+ Malanil	D2H29	35	12.8	1.07
		Sevuparin/DF02+ Malanil	D2H32	65.7	12.3	1.02
		Sevuparin/DF02+ Malanil	D2H35	38.5	13.2	1.1
		Sevuparin/DF02+ Malanil	D2H41	32.2	12.3	1.02
		Sevuparin/DF02+ Malanil	D2H47	34	11.9	0.99
		Sevuparin/DF02+ Malanil	D3H53	38.2	14	1.17
		Sevuparin/DF02+ Malanil	D3H59	33.8	13	1.08
		Sevuparin/DF02+ Malanil	D3H65	32.1	11.6	0.97
		Sevuparin/DF02+ Malanil	D3H68	44.2	11.9	0.99
		Sevuparin/DF02+ Malanil	D3H71	39.2	13.8	1.15
		Sevuparin/DF02+ Malanil	D7	25.8	14.3	1.19

SUBJID	INITIAL	ARM	TIMEPOINT	APTT	PT	INR
		Sevuparin/DF02+ Malanil	screening	28	13.2	1.1
		Sevuparin/DF02+ Malanil	pre-dose	28	13.2	1.1
		Sevuparin/DF02+ Malanil	D1H2	38.9	13.3	1.11
		Sevuparin/DF02+ Malanil	D1H5	30.1	13.6	1.13
		Sevuparin/DF02+ Malanil	D1H11	35.2	16.1	1.33
		Sevuparin/DF02+ Malanil	D1H17	42.4	17.4	1.44
		Sevuparin/DF02+ Malanil	D1H23	44.4	17.5	1.45
		Sevuparin/DF02+ Malanil	D2H29	41.9	17	1.41
		Sevuparin/DF02+ Malanil	D2H32	78.5	21.4	1.76
		Sevuparin/DF02+ Malanil	D2H35	44.1	17.1	1.42
		Sevuparin/DF02+ Malanil	D2H41	43	17.2	1.42
		Sevuparin/DF02+ Malanil	D2H47	40.8	14.6	1.22
		Sevuparin/DF02+ Malanil	D3H53	42.3	16.6	1.37
		Sevuparin/DF02+ Malanil	D3H59	41.2	16.3	1.35
		Sevuparin/DF02+ Malanil	D3H65	39.3	14	1.17
		Sevuparin/DF02+ Malanil	D3H68	54	14.8	1.22
		Sevuparin/DF02+ Malanil	D3H71	39.4	15.3	1.26
		Sevuparin/DF02+ Malanil	D7	28.3	13.4	1.12
		Sevuparin/DF02+ Malanil	D14	28.8	14.2	1.18
		Sevuparin/DF02+ Malanil	screening	26.3	12.1	1.01
		Sevuparin/DF02+ Malanil	pre-dose	26.3	12.1	1.01
		Sevuparin/DF02+ Malanil	D1H2	49.1	13.9	1.16
		Sevuparin/DF02+ Malanil	D1H5	31.2	13.3	1.11

SUBJID	INITIAL	ARM	TIMEPOINT	APTT	PT	INR
		Sevuparin/DF02+ Malanil	D1H11	30.1	14.4	1.2
		Sevuparin/DF02+ Malanil	D1H17	36.5	13.5	1.13
		Sevuparin/DF02+ Malanil	D1H23	26.2	13	1.08
		Sevuparin/DF02+ Malanil	D2H29	38.5	13.2	1.1
		Sevuparin/DF02+ Malanil	D2H32	52	13.6	1.13
		Sevuparin/DF02+ Malanil	D2H35	36.7	13.9	1.16
		Sevuparin/DF02+ Malanil	D2H41	36.8	13.4	1.12
		Sevuparin/DF02+ Malanil	D2H47	39	12.6	1.05
		Sevuparin/DF02+ Malanil	D3H53	38.3	13.2	1.1
		Sevuparin/DF02+ Malanil	D3H59	36.1	13.2	1.1
		Sevuparin/DF02+ Malanil	D3H65	37.2	12.9	1.08
		Sevuparin/DF02+ Malanil	D3H68	53.2	13.5	1.13
		Sevuparin/DF02+ Malanil	D3H71	35.4	13.1	1.09
		Sevuparin/DF02+ Malanil	D7	25.9	11.8	0.98
		Sevuparin/DF02+ Malanil	D14	25.5	10.8	0.9
		Sevuparin/DF02+ Malanil	screening	24.1	11.8	0.98
		Sevuparin/DF02+ Malanil	pre-dose	24.1	11.8	0.98
		Sevuparin/DF02+ Malanil	D1H2	68.9	16.3	1.35
		Sevuparin/DF02+ Malanil	D1H5	38.5	14	1.17
		Sevuparin/DF02+ Malanil	D1H11	45.5	15.9	1.32
		Sevuparin/DF02+ Malanil	D1H17	47.1	16.1	1.33
		Sevuparin/DF02+ Malanil	D1H23	46.3	14.2	1.18
		Sevuparin/DF02+ Malanil	D2H29	45.4	14	1.17

SUBJID	INITIAL	ARM	TIMEPOINT	APTT	PT	INR
		Sevuparin/DF02+ Malanil	D2H32	73.7	16.9	1.4
		Sevuparin/DF02+ Malanil	D2H35	53.9	14.8	1.22
		Sevuparin/DF02+ Malanil	D2H41	48.3	15	1.24
		Sevuparin/DF02+ Malanil	D2H47	48.1	13.9	1.16
		Sevuparin/DF02+ Malanil	D3H53	47.1	14.6	1.22
		Sevuparin/DF02+ Malanil	D3H59	53.7	14.6	1.22
		Sevuparin/DF02+ Malanil	D3H65	57.5	14	1.17
		Sevuparin/DF02+ Malanil	D3H68	71.6	14.9	1.23
		Sevuparin/DF02+ Malanil	D3H71	51.9	13.4	1.12
		Sevuparin/DF02+ Malanil	D7	27.5	10.8	0.9
		Sevuparin/DF02+ Malanil	D14	25.6	13.9	1.16
		Sevuparin/DF02+ Malanil	screening	27.8	14.6	1.22
		Sevuparin/DF02+ Malanil	pre-dose	27.8	14.6	1.22
		Sevuparin/DF02+ Malanil	D1H2	50.4	17.7	1.47
		Sevuparin/DF02+ Malanil	D1H5	33.8	16.4	1.36
		Sevuparin/DF02+ Malanil	D1H11	37	17.6	1.46
		Sevuparin/DF02+ Malanil	D1H17	37.1	16	1.32
		Sevuparin/DF02+ Malanil	D1H23	37.9	18	1.49
		Sevuparin/DF02+ Malanil	D2H29	36.6	14.6	1.22
		Sevuparin/DF02+ Malanil	D2H32	52.2	15.4	1.27
		Sevuparin/DF02+ Malanil	D2H35	38.2	16.8	1.39
		Sevuparin/DF02+ Malanil	D2H41	34	14.9	1.23
		Sevuparin/DF02+ Malanil	D2H47	34.5	14.1	1.18

SUBJID	INITIAL	ARM	TIMEPOINT	APTT	PT	INR
		Sevuparin/DF02+ Malanil	D3H53	28.2	13.1	1.09
		Sevuparin/DF02+ Malanil	D3H59	35.2	14.6	1.22
		Sevuparin/DF02+ Malanil	D3H65	34.3	14.9	1.23
		Sevuparin/DF02+ Malanil	D3H68	48.7	14.2	1.18
		Sevuparin/DF02+ Malanil	D3H71	32.7	14.6	1.22
		Sevuparin/DF02+ Malanil	D7	25.7	13.8	1.15
		Sevuparin/DF02+ Malanil	D14	24.5	13.6	1.13
		Sevuparin/DF02+ Malanil	screening	29.5	12.4	1.03
		Sevuparin/DF02+ Malanil	pre-dose	29.5	12.4	1.03
		Sevuparin/DF02+ Malanil	D1H2	43.8	14.5	1.21
		Sevuparin/DF02+ Malanil	D1H5	34.4	13.2	1.1
		Sevuparin/DF02+ Malanil	D1H11	38.3	17.6	1.42
		Sevuparin/DF02+ Malanil	D1H17	51.4	17.4	1.44
		Sevuparin/DF02+ Malanil	D1H23	35.2	12.7	1.06
		Sevuparin/DF02+ Malanil	D2H29	33.3	12.3	1.02
		Sevuparin/DF02+ Malanil	D2H32	40.7	15.3	1.26
		Sevuparin/DF02+ Malanil	D2H35	35.4	12.9	1.08
		Sevuparin/DF02+ Malanil	D2H41	37	12.9	1.08
		Sevuparin/DF02+ Malanil	D2H47	35	11.7	0.98
		Sevuparin/DF02+ Malanil	D3H53	44.9	16.7	1.38
		Sevuparin/DF02+ Malanil	D3H59	38.2	13.6	1.13
		Sevuparin/DF02+ Malanil	D3H65	37.1	13.6	1.13
		Sevuparin/DF02+ Malanil	D3H68	45.6	14.8	1.22

SUBJID	INITIAL	ARM	TIMEPOINT	APTT	PT	INR
		Sevuparin/DF02+ Malanil	D3H71	33.7	13.3	1.11
		Sevuparin/DF02+ Malanil	D7	26.8	12.5	1.04
		Sevuparin/DF02+ Malanil	D14	26.4	11.2	0.93
		Sevuparin/DF02+ Malanil	screening	30	11.5	0.96
		Sevuparin/DF02+ Malanil	pre-dose	30	11.5	0.96
		Sevuparin/DF02+ Malanil	D1H2	41.3	12.4	1.03
		Sevuparin/DF02+ Malanil	D1H5	29.9	11.9	0.99
		Sevuparin/DF02+ Malanil	D1H11	33.8	12.1	1.01
		Sevuparin/DF02+ Malanil	D1H17	32.1	12	1
		Sevuparin/DF02+ Malanil	D1H23	29.7	11.5	0.96
		Sevuparin/DF02+ Malanil	D2H29	29.3	12.4	1.03
		Sevuparin/DF02+ Malanil	D2H32	46.1	12.3	1.02
		Sevuparin/DF02+ Malanil	D2H35	33.3	11.6	0.97
		Sevuparin/DF02+ Malanil	D2H41	31.6	12.1	1.01
		Sevuparin/DF02+ Malanil	D2H47	30.3	11.7	0.98
		Sevuparin/DF02+ Malanil	D3H53	34.7	12.7	1.06
		Sevuparin/DF02+ Malanil	D3H59	29.5	13.2	1.1
		Sevuparin/DF02+ Malanil	D3H65	30.1	12.2	1.02
		Sevuparin/DF02+ Malanil	D3H68	26	11.1	0.93
		Sevuparin/DF02+ Malanil	D3H71	27.1	10.8	0.9
		Sevuparin/DF02+ Malanil	D7	27.4	11.5	0.96
		Sevuparin/DF02+ Malanil	D14	27.6	11	0.92
		Sevuparin/DF02+ Malanil	screening	22.4	11.7	0.98

SUBJID	INITIAL	ARM	TIMEPOINT	APTT	PT	INR
		Sevuparin/DF02+ Malanil	pre-dose	22.4	11.7	0.98
		Sevuparin/DF02+ Malanil	D1H2	39.4	16	1.32
		Sevuparin/DF02+ Malanil	D1H5	32.7	15.5	1.28
		Sevuparin/DF02+ Malanil	D1H11	42.1	17.6	1.46
		Sevuparin/DF02+ Malanil	D1H17	30.5	13.6	1.13
		Sevuparin/DF02+ Malanil	D1H23	30.3	12.6	1.05
		Sevuparin/DF02+ Malanil	D2H29	31.1	13.4	1.12
		Sevuparin/DF02+ Malanil	D2H32	49.6	15.8	1.31
		Sevuparin/DF02+ Malanil	D2H35	42.9	17.1	1.42
		Sevuparin/DF02+ Malanil	D2H41	34.8	14.6	1.22
		Sevuparin/DF02+ Malanil	D2H47	38.1	13	1.08
		Sevuparin/DF02+ Malanil	D3H53	39.4	15.5	1.28
		Sevuparin/DF02+ Malanil	D3H59	36.8	13.9	1.16
		Sevuparin/DF02+ Malanil	D3H65	41.7	15.2	1.26
		Sevuparin/DF02+ Malanil	D3H68	50.3	14.2	1.18
		Sevuparin/DF02+ Malanil	D3H71	37.8	13.1	1.09
		Sevuparin/DF02+ Malanil	D7	27.6	11.9	0.99
		Sevuparin/DF02+ Malanil	D14	26	12.2	1.02
		Sevuparin/DF02+ Malanil	screening	30.3	12.8	1.07
		Sevuparin/DF02+ Malanil	pre-dose	30.3	12.8	1.07
		Sevuparin/DF02+ Malanil	D1H2	37	14.6	1.22
		Sevuparin/DF02+ Malanil	D1H5	32.8	14.6	1.22
		Sevuparin/DF02+ Malanil	D1H11	34.1	13.8	1.15

SUBJID	INITIAL	ARM	TIMEPOINT	APTT	PT	INR
		Sevuparin/DF02+ Malanil	D1H17	36.8	15.1	1.25
		Sevuparin/DF02+ Malanil	D1H23	34.5	15.5	1.28
		Sevuparin/DF02+ Malanil	D2H29	38.9	15.8	1.31
		Sevuparin/DF02+ Malanil	D2H32	56.9	17.2	1.42
		Sevuparin/DF02+ Malanil	D2H35	44.1	16.2	1.34
		Sevuparin/DF02+ Malanil	D2H41	40.9	16.2	1.34
		Sevuparin/DF02+ Malanil	D2H47	36.2	14.3	1.19
		Sevuparin/DF02+ Malanil	D3H53	35.9	14.5	1.21
		Sevuparin/DF02+ Malanil	D3H59	40	14.7	1.22
		Sevuparin/DF02+ Malanil	D3H65	41.2	15.9	1.32
		Sevuparin/DF02+ Malanil	D3H68	44.6	13.2	1.1
		Sevuparin/DF02+ Malanil	D3H71	35.7	13.6	1.13
		Sevuparin/DF02+ Malanil	D7	27	13.2	1.1
		Sevuparin/DF02+ Malanil	D14	25.8	12.4	1.03
		Sevuparin/DF02+ Malanil	screening	28.5	13.6	1.13
		Sevuparin/DF02+ Malanil	pre-dose	28.5	13.6	1.13
		Sevuparin/DF02+ Malanil	D1H2	39.6	14.1	1.18
		Sevuparin/DF02+ Malanil	D1H5	29.3	13.5	1.13
		Sevuparin/DF02+ Malanil	D1H11	30.6	13.9	1.16
		Sevuparin/DF02+ Malanil	D1H17	32.2	13.9	1.16
		Sevuparin/DF02+ Malanil	D1H23	30.6	14.3	1.19
		Sevuparin/DF02+ Malanil	D2H29	33.3	13.8	1.15
		Sevuparin/DF02+ Malanil	D2H32	41.1	13	1.08

SUBJID	INITIAL	ARM	TIMEPOINT	APTT	PT	INR
		Sevuparin/DF02+ Malanil	D2H35	33.1	14.1	1.18
		Sevuparin/DF02+ Malanil	D2H41	33.6	13.5	1.13
		Sevuparin/DF02+ Malanil	D2H47	32.8	13.8	1.15
		Sevuparin/DF02+ Malanil	D3H53	36.4	15.6	1.29
		Sevuparin/DF02+ Malanil	D3H59	34.3	14.1	1.18
		Sevuparin/DF02+ Malanil	D3H65	35.6	14	1.17
		Sevuparin/DF02+ Malanil	D3H68	46.7	14	1.17
		Sevuparin/DF02+ Malanil	D3H71	36.9	13.9	1.16
		Sevuparin/DF02+ Malanil	D7	30.1	13.7	1.14
		Sevuparin/DF02+ Malanil	D14	28.8	12.8	1.07
		Sevuparin/DF02+ Malanil	screening	29.3	15.5	1.28
		Sevuparin/DF02+ Malanil	pre-dose	29.3	15.5	1.28
		Sevuparin/DF02+ Malanil	D1H2	48	16.5	1.37
		Sevuparin/DF02+ Malanil	D1H5	35.2	16.9	1.4
		Sevuparin/DF02+ Malanil	D1H11	37.1	18.7	1.55
		Sevuparin/DF02+ Malanil	D1H17	43	18.2	1.51
		Sevuparin/DF02+ Malanil	D1H23	47.1	17.2	1.42
		Sevuparin/DF02+ Malanil	D2H29	44.1	14.1	1.18
		Sevuparin/DF02+ Malanil	D2H32	72.4	16.1	1.33
		Sevuparin/DF02+ Malanil	D2H35	52.2	16.1	1.33
		Sevuparin/DF02+ Malanil	D2H41	46.5	14.1	1.18
		Sevuparin/DF02+ Malanil	D2H47	44.8	13.2	1.1
		Sevuparin/DF02+ Malanil	D3H53	40	13.4	1.12

SUBJID	INITIAL	ARM	TIMEPOINT	APTT	PT	INR
		Sevuparin/DF02+ Malanil	D3H59	47	14.5	1.21
		Sevuparin/DF02+ Malanil	D3H65	43	14.1	1.18
		Sevuparin/DF02+ Malanil	D3H68	55.8	13.2	1.1
		Sevuparin/DF02+ Malanil	D3H71	39.5	13	1.08
		Sevuparin/DF02+ Malanil	D7	29.6	12.7	1.06
		Sevuparin/DF02+ Malanil	D14	29.4	13.6	1.13
		Sevuparin/DF02+ Malanil	screening	38.8	14.7	1.22
		Sevuparin/DF02+ Malanil	pre-dose	38.8	14.7	1.22
		Sevuparin/DF02+ Malanil	D1H2	46.1	15.5	1.28
		Sevuparin/DF02+ Malanil	D1H5	35.1	15.1	1.25
		Sevuparin/DF02+ Malanil	D1H11	34.7	14.9	1.23
		Sevuparin/DF02+ Malanil	D1H17	35.7	14.5	1.21
		Sevuparin/DF02+ Malanil	D1H23	36.5	14.1	1.18
		Sevuparin/DF02+ Malanil	D2H29	39.1	14.9	1.23
		Sevuparin/DF02+ Malanil	D2H32	54.5	16.2	1.34
		Sevuparin/DF02+ Malanil	D2H35	49.4	16.1	1.33
		Sevuparin/DF02+ Malanil	D2H41	40.2	15.6	1.29
		Sevuparin/DF02+ Malanil	D2H47	44.6	17.2	1.42
		Sevuparin/DF02+ Malanil	D3H53	45.6	17.9	1.48
		Sevuparin/DF02+ Malanil	D3H59	42.5	16.2	1.34
		Sevuparin/DF02+ Malanil	D3H65	33.5	13.7	1.14
		Sevuparin/DF02+ Malanil	D3H68	49.1	15.2	1.26
		Sevuparin/DF02+ Malanil	D3H71	42	15.1	1.25

SUBJID	INITIAL	ARM	TIMEPOINT	APTT	PT	INR
		Sevuparin/DF02+ Malanil	D7	32.1	13.6	1.13
		Sevuparin/DF02+ Malanil	D14	31.9	14.6	1.22
		Sevuparin/DF02+ Malanil	screening	25.7	12.5	1.04
		Sevuparin/DF02+ Malanil	pre-dose	25.7	12.5	1.04
		Sevuparin/DF02+ Malanil	D1H2	32.1	12.3	1.02
		Sevuparin/DF02+ Malanil	D1H5	25.5	12.2	1.02
		Sevuparin/DF02+ Malanil	D1H11	28.7	12.8	1.07
		Sevuparin/DF02+ Malanil	D1H17	28.1	13.1	1.09
		Sevuparin/DF02+ Malanil	D1H23	30.9	13.9	1.16
		Sevuparin/DF02+ Malanil	D2H29	28.5	13.4	1.12
		Sevuparin/DF02+ Malanil	D2H32	41.5	12.8	1.07
		Sevuparin/DF02+ Malanil	D2H35	29.2	13.1	1.09
		Sevuparin/DF02+ Malanil	D2H41	28.7	13.3	1.11
		Sevuparin/DF02+ Malanil	D2H47	32.6	13.1	1.09
		Sevuparin/DF02+ Malanil	D3H53	32.3	14.5	1.21
		Sevuparin/DF02+ Malanil	D3H59	29.3	13.2	1.1
		Sevuparin/DF02+ Malanil	D3H65	28.6	13.8	1.15
		Sevuparin/DF02+ Malanil	D3H68	37.3	14.3	1.19
		Sevuparin/DF02+ Malanil	D3H71	28.8	13.6	1.13
		Sevuparin/DF02+ Malanil	D7	24.9	11.5	0.96
		Sevuparin/DF02+ Malanil	D14	24.6	11.7	0.98
		Sevuparin/DF02+ Malanil	screening	28.1	13.7	1.14
		Sevuparin/DF02+ Malanil	pre-dose	28.1	13.7	1.14

SUBJID	INITIAL	ARM	TIMEPOINT	APTT	PT	INR
		Sevuparin/DF02+ Malanil	D1H2	38.7	14.1	1.18
		Sevuparin/DF02+ Malanil	D1H5	30.5	13.8	1.15
		Sevuparin/DF02+ Malanil	D1H11	36.6	15.1	1.25
		Sevuparin/DF02+ Malanil	D1H17	28.5	12.6	1.05
		Sevuparin/DF02+ Malanil	D1H23	35.2	16.3	1.35
		Sevuparin/DF02+ Malanil	D2H29	32.5	14.3	1.19
		Sevuparin/DF02+ Malanil	D2H32	42.8	13.6	1.13
		Sevuparin/DF02+ Malanil	D2H35	31.5	13.7	1.14
		Sevuparin/DF02+ Malanil	D2H41	37.2	16.7	1.38
		Sevuparin/DF02+ Malanil	D2H47	35.9	14.1	1.18
		Sevuparin/DF02+ Malanil	D3H53	31.4	12.6	1.05
		Sevuparin/DF02+ Malanil	D3H59	34.2	13.9	1.16
		Sevuparin/DF02+ Malanil	D3H65	31.4	13.1	1.09
		Sevuparin/DF02+ Malanil	D3H68	52.6	13.6	1.13
		Sevuparin/DF02+ Malanil	D3H71	31.4	11.6	0.97
		Sevuparin/DF02+ Malanil	D7	24.7	11.3	0.94
		Sevuparin/DF02+ Malanil	D14	23.3	10.5	0.88
		Sevuparin/DF02+ Malanil	screening	33.8	15.3	1.26
		Sevuparin/DF02+ Malanil	pre-dose	33.8	15.3	1.26
		Sevuparin/DF02+ Malanil	D1H2	49.1	15.1	1.25
		Sevuparin/DF02+ Malanil	D1H5	35.6	15.3	1.26
		Sevuparin/DF02+ Malanil	D1H11	39	15.6	1.29
		Sevuparin/DF02+ Malanil	D1H17	38.8	14.4	1.2

SUBJID	INITIAL	ARM	TIMEPOINT	APTT	PT	INR
		Sevuparin/DF02+ Malanil	D1H23	36.4	15.9	1.32
		Sevuparin/DF02+ Malanil	D2H29	38	14.9	1.23
		Sevuparin/DF02+ Malanil	D2H32	55.7	15.6	1.29
		Sevuparin/DF02+ Malanil	D2H35	40.4	14.2	1.18
		Sevuparin/DF02+ Malanil	D2H41	39.7	15.1	1.25
		Sevuparin/DF02+ Malanil	D2H47	33.5	13.8	1.15
		Sevuparin/DF02+ Malanil	D3H53	38.2	15.4	1.27
		Sevuparin/DF02+ Malanil	D3H59	34.6	13.6	1.13
		Sevuparin/DF02+ Malanil	D3H65	38.1	14.4	1.2
		Sevuparin/DF02+ Malanil	D3H68	35.5	14.7	1.22
		Sevuparin/DF02+ Malanil	D3H71	33.1	14.3	1.19
		Sevuparin/DF02+ Malanil	D7	29	15.5	1.28
		Sevuparin/DF02+ Malanil	D14	26.9	12.5	1.04
		Sevuparin/DF02+ Malanil	screening	29.7	15.5	1.28
		Sevuparin/DF02+ Malanil	pre-dose	29.7	15.5	1.28
		Sevuparin/DF02+ Malanil	D1H2	37.5	13.9	1.16
		Sevuparin/DF02+ Malanil	D1H5	27.7	13.4	1.12
		Sevuparin/DF02+ Malanil	D1H11	28	13	1.08
		Sevuparin/DF02+ Malanil	D1H17	29.3	13	1.08
		Sevuparin/DF02+ Malanil	D1H23	28.7	13.5	1.13
		Sevuparin/DF02+ Malanil	D2H29	30.7	14.1	1.18
		Sevuparin/DF02+ Malanil	D2H32	43.5	14	1.17
		Sevuparin/DF02+ Malanil	D2H35	36.1	14.6	1.22

SUBJID	INITIAL	ARM	TIMEPOINT	APTT	PT	INR
		Sevuparin/DF02+ Malanil	D2H41	40	11.8	0.98
		Sevuparin/DF02+ Malanil	D2H47	29.1	12.4	1.03
		Sevuparin/DF02+ Malanil	D3H53	31.6	13.1	1.09
		Sevuparin/DF02+ Malanil	D3H59	31.9	12.6	1.05
		Sevuparin/DF02+ Malanil	D3H65	42	12.9	1.08
		Sevuparin/DF02+ Malanil	D3H68	47.5	13.6	1.13
		Sevuparin/DF02+ Malanil	D3H71	36.3	14.2	1.18
		Sevuparin/DF02+ Malanil	D7	28.2	12.9	1.08
		Sevuparin/DF02+ Malanil	D14	28.5	12.8	1.07

16.2.9 Concomitant medications

SUBJID	INITIAL	ARM	CM SEQ	DRUGNAME	ATC TERM	ATC CODE	START DATE TIME *99:99=UNK*	DUR (days)	DOSE	UNIT	ROUTE	FREQ	INDICATION
		Sevuparin/DF02+ Malanil	1	ALB	ALBENDAZOLE	P02CA03		3	2	TABS	PO	OD	EOSINOPHILIA,PARSITE STOOL TEST POSITIVE
		Sevuparin/DF02+ Malanil	1	Potassium Chloride	POTASSIUM CHLORIDE	B05XA01		2	10	ML	IV	OD	HYPOKALEMIA
		Sevuparin/DF02+ Malanil	2	PARA	PARACETAMOL	N02BE01		1	300	MG	IM	OD	FEVER
		Sevuparin/DF02+ Malanil	3	FS	FERROUS SULFATE	B03AA07		20	1	TABS	PO	TDS	ANEMIA
		Sevuparin/DF02+ Malanil	4	Dextrose 5% in normal saline	ELECTROLYTES WITH CARBOHYDRATES	B05BB02		2	1000	ML	IV	OD	HYPOKALEMIA
		Malanil	1	Chlorphenira mine	CHLORPHENAMINE	R06AB04		1	2	TABS	PO	OD	ERYTHEMATOUS RASH
		Malanil	2	Calamine lotion	OTHER ANTIPRURITICS	D04AX		1	60	ML	TOP	PRN	ERYTHEMATOUS RASH
		Malanil	1	PARA	PARACETAMOL	N02BE01		NK	2	TABS	PO	PRN	FEVER
		Sevuparin/DF02+ Malanil	1	PARA	PARACETAMOL	N02BE01		2	2	TABS	PO	PRN	FEVER
		Sevuparin/DF02+ Malanil	2	GLUCOSE	GLUCOSE	V06DC01		1	50	ML	IV	PRN	ANOREXIA

SUBJID	INIT IAL	ARM	CM SEQ	DRUGNAME	ATC TERM	ATC CODE	START DATE TIME *99:99=UNK*	DUR (days)	DOSE	UNIT	ROUTE	FREQ	INDICATION
		Sevuparin/DF02+ Malanil	3	PARA	PARACETAMOL	N02BE01		1	2	ML	IM	PRN	FEVER
		Sevuparin/DF02+ Malanil	4	5% DNSS Dextrose 5% in Saline	ELECTROLYTES WITH CARBOHYDRATES	B05BB02		1	1000	ML	IV	OD	ANOREXIA
		Sevuparin/DF02+ Malanil	5	PLA	METOCLOPRAMIDE	A03FA01		1	2	ML	IV	PRN	ANOREXIA
		Sevuparin/DF02+ Malanil	6	AMOX	AMOXICILLIN	J01CA04		8	2	TABS	PO	BD	PHARYNGITIS
		Sevuparin/DF02+ Malanil	7	FLUIMUCIL	ACETYLCYSTEINE	R05CB01		4	100	MG	PO	TDS	PHARYNGITIS
		Sevuparin/DF02+ Malanil	8	PARA	PARACETAMOL	N02BE01		NK	2	TABS	PO	PRN	PHARYNGITIS
		Sevuparin/DF02+ Malanil	1	5% DNSS Dextrose 5% in Saline	ELECTROLYTES WITH CARBOHYDRATES	B05BB02		1	2000	ML	IV	OD	SUPPLEMENT
		Sevuparin/DF02+ Malanil	2	PARA	PARACETAMOL	N02BE01		2	2	TABS	PO	PRN	FEVER
		Sevuparin/DF02+ Malanil	3	ALUMMILK	ANTACIDS	A02A		1	30	ML	PO	PRN	ABDOMINAL PAIN
		Sevuparin/DF02+ Malanil	4	ORS	ORAL REHYDRATION SALT FORMULATIONS	A07CA		1	1	UNITS	PO	PRN	SUPPLEMENT
		Malanil	1	PARA	PARACETAMOL	N02BE01		1	2	TABS	PO	PRN	FEVER

SUBJID	INITIAL	ARM	CM SEQ	DRUGNAME	ATC TERM	ATC CODE	START DATE TIME *99:99=UNK*	DUR (days)	DOSE	UNIT	ROUTE	FREQ	INDICATION
		Sevuparin/DF02+ Malanil	1	PARA	PARACETAMOL	N02BE01		1	2	TABS	PO	PRN	FEVER
		Sevuparin/DF02+ Malanil	1	PARA	PARACETAMOL	N02BE01		2	2	TABS	PO	PRN	FEVER
		Malanil	1	PARA	PARACETAMOL	N02BE01		3	2	TABS	PO	PRN	FEVER
		Malanil	2	PLA	METOCLOPRAMIDE	A03FA01		1	10	MG	IV	PRN	NAUSEA
		Malanil	3	DOMPERIDONE	DOMPERIDONE	A03FA03		3	1	TABS	PO	QDS	ANTIEMETIC
		Malanil	1	PARA	PARACETAMOL	N02BE01		1	2	TABS	PO	PRN	FEVER
		Malanil	2	POTASSIUM CHLORIDE ELIXIR	POTASSIUM CHLORIDE	A12BA01		1	30	ML	PO	PRN	HYPOKALEMIA
		Sevuparin/DF02+ Malanil	1	PARA	PARACETAMOL	N02BE01		1	2	TABS	PO	PRN	FEVER
		Sevuparin/DF02+ Malanil	2	OFLOXACIN	OFLOXACIN	J01MA01		5	400	MG	PO	BD	URINARY TRACT INFECTION
		Sevuparin/DF02+ Malanil	3	Levonorgestrel (0.15mg) Ethinyl estradiol (0.03mg)	LEVONORGESTREL AND ETHINYLESTRADIOL	G03AA07		ONGOING	1	TABS	PO	OD	PREVENT PREGNANCY
		Sevuparin/DF02+	1	PLA	METOCLOPRAMIDE	A03FA01		1	10	MG	IV	PRN	VOMITING

SUBJID	INIT IAL	ARM	CM SEQ	DRUGNAME	ATC TERM	ATC CODE	START DATE TIME *99:99=UNK*	DUR (days)	DOSE	UNIT	ROUTE	FREQ	INDICATION
		Malanil											
		Sevuparin/DF02+ Malanil	2	PARA	PARACETAMOL	N02BE01		3	2	TABS	PO	PRN	FEVER
		Sevuparin/DF02+ Malanil	3	Levonorgestre l (0.15 mg) Ethinyl estradiol (0.03 mg)	LEVONORGESTREL AND ETHINYLESTRADIOL	G03AA07		ONGO ING	1	TABS	PO	OD	PREVENT PREGNANCY
		Sevuparin/DF02+ Malanil	1	PARA	PARACETAMOL	N02BE01		3	2	TABS	PO	PRN	FEVER
		Sevuparin/DF02+ Malanil	2	NORMAL SALINE SOLUTION	SODIUM CHLORIDE	B05XA03		2	1000	ML	IV	OD	ANOREXIA
		Sevuparin/DF02+ Malanil	3	RANITIDINE	RANITIDINE	A02BA02		4	1	TABS	PO	BD	ABDOMINAL PAIN
		Sevuparin/DF02+ Malanil	4	ANTACID SUSPENSIO N	ANTACIDS	A02A		4	30	ML	PO	TDS	ABDOMINAL PAIN
		Sevuparin/DF02+ Malanil	5	Levonorgestre l (0.15 mg) Ethinyl estradiol (0.03 mg)	LEVONORGESTREL AND ETHINYLESTRADIOL	G03AA07		ONGO ING	1	TABS	PO	OD	PREVENT PREGNANCY
		Sevuparin/DF02+ Malanil	1	PARA	PARACETAMOL	N02BE01		3	2	TABS	PO	PRN	FEVER
		Sevuparin/DF02+ Malanil	1	PARA	PARACETAMOL	N02BE01		2	2	TABS	PO	PRN	FEVER

SUBJID	INITIAL	ARM	CM SEQ	DRUGNAME	ATC TERM	ATC CODE	START DATE TIME *99:99=UNK*	DUR (days)	DOSE	UNIT	ROUTE	FREQ	INDICATION
		Sevuparin/DF02+ Malanil	2	NORMAL SALINE SOLUTION	SODIUM CHLORIDE	B05XA03		4	1920	ML	IV	OD	ANOREXIA
		Sevuparin/DF02+ Malanil	3	POTASSIUM CHLORIDE ELIXIR	POTASSIUM CHLORIDE	A12BA01		1	30	ML	PO	PRN	ANOREXIA
		Sevuparin/DF02+ Malanil	1	PARA	PARACETAMOL	N02BE01		2	2	TABS	PO	PRN	FEVER
		Sevuparin/DF02+ Malanil	2	POTASSIUM CHLORIDE ELIXIR	POTASSIUM CHLORIDE	A12BA01		1	60	ML	PO	OD	ANOREXIA
		Sevuparin/DF02+ Malanil	1	POTASSIUM CHLORIDE ELIXIR	POTASSIUM CHLORIDE	A12BA01		1	30	ML	PO	PRN	HYPOKALEMIA
		Sevuparin/DF02+ Malanil	2	PARA	PARACETAMOL	N02BE01		3	2	TABS	PO	PRN	FEVER
		Malanil	1	PARA	PARACETAMOL	N02BE01		2	2	TABS	PO	PRN	FEVER
		Malanil	1	PARA	PARACETAMOL	N02BE01		2	2	TABS	PO	PRN	FEVER
		Malanil	1	PARA	PARACETAMOL	N02BE01		1	2	TABS	PO	PRN	FEVER
		Malanil	1	PARA	PARACETAMOL	N02BE01		1	2	TABS	PO	PRN	FEVER
		Malanil	1	PARA	PARACETAMOL	N02BE01		2	2	TABS	PO	PRN	FEVER

SUBJID	INIT IAL	ARM	CM SEQ	DRUGNAME	ATC TERM	ATC CODE	START DATE TIME *99:99=UNK*	DUR (days)	DOSE	UNIT	ROUTE	FREQ	INDICATION
		Malanil	1	PARA	PARACETAMOL	N02BE01		2	2	TABS	PO	PRN	FEVER
		Malanil	1	PARA	PARACETAMOL	N02BE01		2	2	TABS	PO	PRN	FEVER
		Sevuparin/DF02+ Malanil	1	PARA	PARACETAMOL	N02BE01		3	2	TABS	PO	PRN	FEVER
		Malanil	1	PARA	PARACETAMOL	N02BE01		2	2	TABS	PO	PRN	FEVER
		Malanil	1	PARA	PARACETAMOL	N02BE01		1	2	TABS	PO	PRN	FEVER
		Sevuparin/DF02+ Malanil	1	PARA	PARACETAMOL	N02BE01		1	2	TABS	PO	PRN	FEVER
		Sevuparin/DF02+ Malanil	2	FERROUS SULFATE	FERROUS SULFATE	B03AA07		20	1	TABS	PO	TDS	PROPHYLAXIS ANEMIA
		Sevuparin/DF02+ Malanil	1	PARA	PARACETAMOL	N02BE01		1	2	TABS	PO	PRN	FEVER
		Sevuparin/DF02+ Malanil	1	PARA	PARACETAMOL	N02BE01		3	2	TABS	PO	PRN	FEVER
		Sevuparin/DF02+ Malanil	2	Dextrose 5% in sterile saline	ELECTROLYTES WITH CARBOHYDRATES	B05BB02		2	1000	ML	IV	OD	ANOREXIA
		Sevuparin/DF02+ Malanil	1	PARA	PARACETAMOL	N02BE01		1	2	TABS	PO	PRN	FEVER

SUBJID	INIT IAL	ARM	CM SEQ	DRUGNAME	ATC TERM	ATC CODE	START DATE TIME *99:99=UNK*	DUR (days)	DOSE	UNIT	ROUTE	FREQ	INDICATION
		Malanil	1	PARA	PARACETAMOL	N02BE01		3	2	TABS	PO	PRN	FEVER
		Malanil	2	Dextrose 5% in 0.5 sterile saline	ELECTROLYTES WITH CARBOHYDRATES	B05BB02		2	2000	ML	IV	OD	ANOREXIA
		Malanil	1	PARA	PARACETAMOL	N02BE01		3	2	TABS	PO	PRN	FEVER
		Malanil	1	PARA	PARACETAMOL	N02BE01		3	2	TABS	PO	PRN	FEVER
