# Supplementary figures and images for: Inhibition of merozoite invasion and transient de-sequestration by sevuparin in humans with Plasmodium falciparum malaria
Source: PLoS One. 2017 Dec 15;12(12):e0188754. doi: 10.1371/journal.pone.0188754 (PMC5731734; doi:10.1371/journal.pone.0188754)

S1 Fig

Malanil

Sevuparin for anti-adhesion and anti-invasion

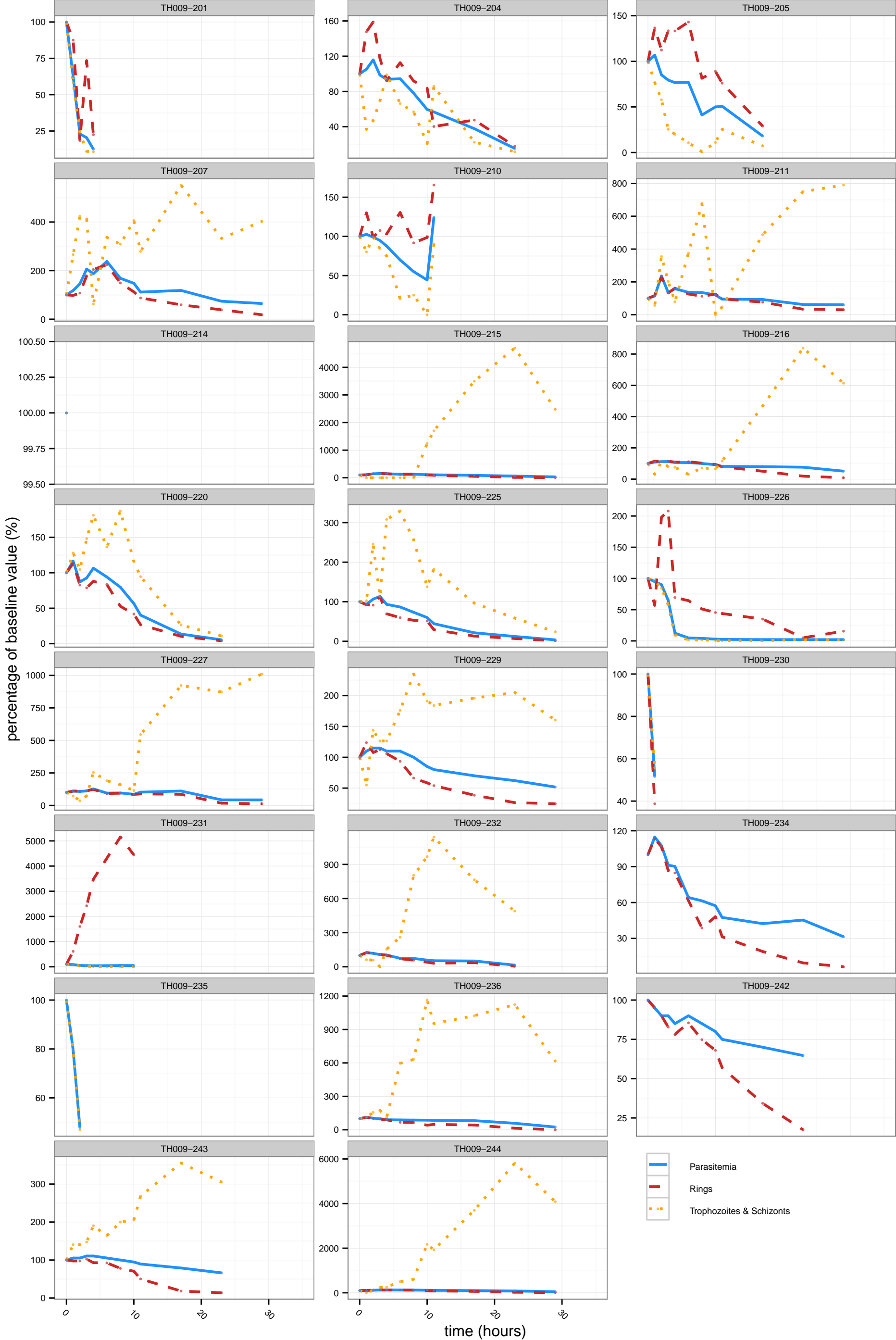

FS2 Fig cont.

Sevuparin/Malanil

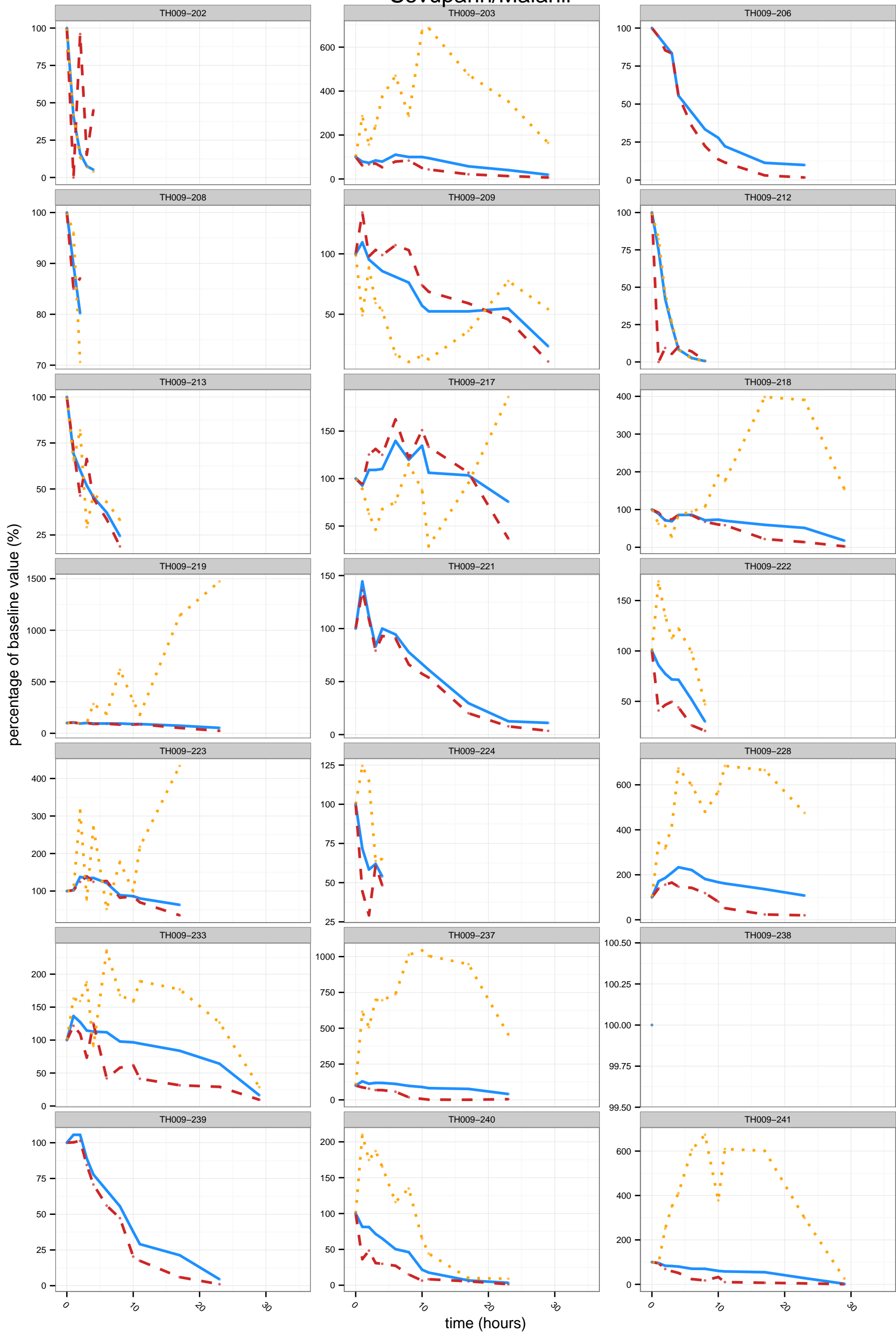

Supplement: S1 Fig — (PDF) [file pone.0188754.s006.pdf]
