# Supplementary material for: Inhibition of merozoite invasion and transient de-sequestration by sevuparin in humans with Plasmodium falciparum malaria
Source: PLoS One. 2017 Dec 15;12(12):e0188754. doi: 10.1371/journal.pone.0188754 (PMC5731734; doi:10.1371/journal.pone.0188754)
Supplement: S1 Table — (DOCX) [file pone.0188754.s007.docx]

**S1 Table Shift table of Anti Xa in patients with uncomplicated malaria treated with sevuparin.**

|  | **Day 01 H -1** | |  | **Highest recorded value** | | |
| --- | --- | --- | --- | --- | --- | --- |
| **Part 1** | **1.5 mg/kg sevuparin** | | | | | |
|  |  | (0-0.1] | | (0.1-0.2] | (0.2-0.5] | (0.5 - |
|  | (0-0.1] | 3 | | 0 | 0 | 0 |
|  | (0.1-0.2] | 0 | | 0 | 0 | 0 |
|  | (0.2-0.5] | 0 | | 0 | 0 | 0 |
|  | (0.5 - | 0 | | 0 | 0 | 0 |
|  |  | **3.0 mg/kg sevuparin** | | | | |
|  |  | (0-0.1] | | (0.1-0.2] | (0.2-0.5] | (0.5 - |
|  | (0-0.1] | 0 | | 0 | 2 | 1 |
|  | (0.1-0.2] | 0 | | 0 | 0 | 0 |
|  | (0.2-0.5] | 0 | | 0 | 0 | 0 |
|  | (0.5 - | 0 | | 0 | 0 | 0 |
|  |  | **1.5 mg/kg sevuparin** | | | | |
|  |  | (0-0.1] | | (0.1-0.2] | (0.2-0.5] | (0.5 - |
|  | (0-0.1] | 0 | | 3 | 0 | 0 |
|  | (0.1-0.2] | 0 | | 0 | 0 | 0 |
|  | (0.2-0.5] | 0 | | 0 | 0 | 0 |
|  | (0.5 - | 0 | | 0 | 0 | 0 |
| **Part 2** | **3 mg/kg sevuparin** | | | | | |
|  |  | (0-0.1] | | (0.1-0.2] | (0.2-0.5] | (0.5 - |
|  | (0-0.1] | 18 | | 2 | 1 | 0 |
|  | (0.1-0.2] | 0 | | 0 | 0 | 0 |
|  | (0.2-0.5] | 0 | | 0 | 0 | 0 |
|  | (0.5 - | 0 | | 0 | 0 | 0 |
