# Supplementary material for: Inhibition of merozoite invasion and transient de-sequestration by sevuparin in humans with Plasmodium falciparum malaria
Source: PLoS One. 2017 Dec 15;12(12):e0188754. doi: 10.1371/journal.pone.0188754 (PMC5731734; doi:10.1371/journal.pone.0188754)
Supplement: S2 Table — (DOCX) [file pone.0188754.s008.docx]

**S2 Table Summary of PT (seconds) and INR in patient with uncomplicated malaria receiving 1.5, 3, and 6 mg/kg sevuparin in part 1 and 3 mg/kg sevuparin in part 2.**

|  | **Dose** | **Day 1**  **Mean (SD)** | **Day 3***  **Mean (SD)** | **Day 7**  **Mean (SD)** | **Day 14**  **Mean (SD)** | **Abs. change Day 3-Day 1**  **Mean (SD)** | **Rel. change (%) Day 3-Day1**  **Mean (SD)** |
| --- | --- | --- | --- | --- | --- | --- | --- |
|  | **Part 1** |  |  |  |  |  |  |
| PT (sec) | Sevuparin 1.5 mg/kg | 12.53 (0.32) | 13.23 (1.62) | 11.93 (0.64) | 11.20 (1.40) | 0.70 (1.30) | 5.41 (10.09) |
|  | Sevuparin 3.0 mg/kg | 13.20 (1.65) | 13.17 (1.51) | 12.50 (1.68) | 11.50 (0.36) | -0.03 (1.11) | 0.12 (8.87) |
|  | Sevuparin 6.0 mg/kg | 12.57 (0.72) | 13.23 (0.35) | 12.50 (0.17) | 11.37 (0.55) | 0.67 (0.42) | 5.43 (3.50) |
| INR | Sevuparin 1.5 mg/kg | 1.04 (0.03) | 1.10 (0.13) | 0.99 (0.05) | 0.93 (0.12) | 0.06 (0.10) | 5.25 (9.09) |
|  | Sevuparin 3.0 mg/kg | 1.10 (0.13) | 1.09 (0.12) | 1.04 (0.14) | 1.07 (0.11) | 0.00 (0.09) | 0.05 (8.70) |
|  | Sevuparin 6.0 mg/kg | 1.05 (0.06) | 1.10 (0.03) | 1.04 (0.02) | 1.00 (0.06) | 0.05 (0.04) | 5.22 (3.78) |
|  | **Part 2** |  |  |  |  |  |  |
| PT (sec) | Sevuparin 3.0 mg/kg | 13.28 (1.34) | 13.31 (1.19) | 12.84 (1.24) | 12.47 (1.21) | 0.03 (1.27) | 0.76 (9.38) |
| INR | Sevuparin 3.0 mg/kg | 1.11 (0.11) | 1.11 (0.09) | 1.08 (0.11) | 1.04 (0.10) | 0.00 (0.10) | 0.74 (9.15) |

*The Day 3 timepoint used is H71, 5 hours following the last sevuparin injection
