# Supplementary material for: Inhibition of merozoite invasion and transient de-sequestration by sevuparin in humans with Plasmodium falciparum malaria
Source: PLoS One. 2017 Dec 15;12(12):e0188754. doi: 10.1371/journal.pone.0188754 (PMC5731734; doi:10.1371/journal.pone.0188754)
Supplement: S3 Table — (DOCX) [file pone.0188754.s009.docx]

**S3 Table APTT (seconds) at 1, 2 and 5 hours following sevuparin dose number 1 and 12, part 1.**

|  |  | **n** | **Mi-**  **ssing** | **Mean** | **SD** | **Min** | **Q1** | **Median** | **Q3** | **Max** | **Lower**  **95CI** | **Upper**  **95CI** |
| --- | --- | --- | --- | --- | --- | --- | --- | --- | --- | --- | --- | --- |
| APTT (sec) | | | | | | | | | | | | |
| **Sevuparin 1.5 mg/kg** | | | | | | | | | | | | |
| Day 01 Hour 00 screening | obs | 3 | 0 | 28.60 | 0.95 | 27.50 | 28.30 | 29.10 | 29.15 | 29.20 | 26.23 | 30.97 |
| **Dose 1** |  |  |  |  |  |  |  |  |  |  |  |  |
| Day 01 Hour 01 | obs | 3 | 0 | 42.47 | 3.98 | 39.20 | 40.25 | 41.30 | 44.10 | 46.90 | 32.58 | 52.35 |
| Day 01 Hour 01 | cha | 3 | 0 | 13.87 | 3.41 | 11.70 | 11.90 | 12.10 | 14.95 | 17.80 | 5.39 | 22.34 |
| Day 01 Hour 01 | %cha | 3 | 0 | 48.38 | 11.09 | 41.44 | 41.99 | 42.55 | 51.86 | 61.17 | 20.85 | 75.92 |
| Day 01 Hour 02 | obs | 3 | 0 | 34.97 | 1.46 | 33.80 | 34.15 | 34.50 | 35.55 | 36.60 | 31.35 | 38.59 |
| Day 01 Hour 02 | cha | 3 | 0 | 6.37 | 1.00 | 5.40 | 5.85 | 6.30 | 6.85 | 7.40 | 3.88 | 8.85 |
| Day 01 Hour 02 | %cha | 3 | 0 | 22.27 | 3.44 | 18.56 | 20.73 | 22.91 | 24.13 | 25.34 | 13.73 | 30.81 |
| Day 01 Hour 05 | obs | 3 | 0 | 30.40 | 2.29 | 27.80 | 29.55 | 31.30 | 31.70 | 32.10 | 24.72 | 36.08 |
| Day 01 Hour 05 | cha | 3 | 0 | 1.80 | 1.35 | 0.30 | 1.25 | 2.20 | 2.55 | 2.90 | -1.54 | 5.14 |
| Day 01 Hour 05 | %cha | 3 | 0 | 6.19 | 4.58 | 1.09 | 4.33 | 7.56 | 8.75 | 9.93 | -5.17 | 17.56 |
| **Sevuparin 3.0 mg/kg** | | | | | | | | | | | | |
| Day 01 Hour 00 screening | obs | 3 | 0 | 28.97 | 2.42 | 26.20 | 28.10 | 30.00 | 30.35 | 30.70 | 22.95 | 34.98 |
| **Dose 1** |  |  |  |  |  |  |  |  |  |  |  |  |
| Day 01 Hour 01 | obs | 3 | 0 | 51.73 | 3.27 | 48.00 | 50.55 | 53.10 | 53.60 | 54.10 | 43.61 | 59.86 |
| Day 01 Hour 01 | cha | 3 | 0 | 22.77 | 4.94 | 17.30 | 20.70 | 24.10 | 25.50 | 26.90 | 10.50 | 35.03 |
| Day 01 Hour 01 | %cha | 3 | 0 | 79.79 | 23.16 | 56.35 | 68.34 | 80.33 | 91.50 | 102.67 | 22.24 | 137.33 |
| Day 01 Hour 02 | obs | 3 | 0 | 40.73 | 3.48 | 36.90 | 39.25 | 41.60 | 42.65 | 43.70 | 32.08 | 49.38 |
| Day 01 Hour 02 | cha | 3 | 0 | 11.77 | 4.90 | 6.20 | 9.95 | 13.70 | 14.55 | 15.40 | -0.39 | 23.93 |
| Day 01 Hour 02 | %cha | 3 | 0 | 41.55 | 19.62 | 20.20 | 32.93 | 45.67 | 52.22 | 58.78 | -7.19 | 90.28 |
| Day 01 Hour 05 | obs | 3 | 0 | 36.80 | 4.94 | 33.90 | 33.95 | 34.00 | 38.25 | 42.50 | 24.54 | 49.06 |
| Day 01 Hour 05 | cha | 3 | 0 | 7.83 | 7.34 | 3.20 | 3.60 | 4.00 | 10.15 | 16.30 | -10.41 | 26.07 |
| Day 01 Hour 05 | %cha | 3 | 0 | 28.66 | 29.10 | 10.42 | 11.88 | 13.33 | 37.77 | 62.21 | -43.63 | 100.94 |
| **Sevuparin 6.0 mg/kg** | | | | | | | | | | | | |
| Day 01 Hour 00 screening | obs | 3 | 0 | 26.40 | 4.01 | 22.60 | 24.30 | 26.00 | 28.30 | 30.60 | 16.43 | 36.37 |
| **Dose 1** |  |  |  |  |  |  |  |  |  |  |  |  |
| Day 01 Hour 01 | obs | 3 | 0 | 77.57 | 34.58 | 55.30 | 57.65 | 60.00 | 88.70 | 117.40 | -8.33 | 163.46 |
| Day 01 Hour 01 | cha | 3 | 0 | 51.17 | 31.12 | 29.30 | 33.35 | 37.40 | 62.10 | 86.80 | -26.15 | 128.48 |
| Day 01 Hour 01 | %cha | 3 | 0 | 187.28 | 87.54 | 112.69 | 139.09 | 165.49 | 224.57 | 283.66 | -30.19 | 404.75 |
| Day 01 Hour 02 | obs | 3 | 0 | 47.57 | 16.81 | 35.70 | 37.95 | 40.20 | 53.50 | 66.80 | 5.81 | 89.32 |
| Day 01 Hour 02 | cha | 3 | 0 | 21.17 | 13.61 | 9.70 | 13.65 | 17.60 | 26.90 | 36.20 | -12.63 | 54.96 |
| Day 01 Hour 02 | %cha | 3 | 0 | 77.83 | 40.50 | 37.31 | 57.59 | 77.88 | 98.09 | 118.30 | -22.77 | 178.43 |
| Day 01 Hour 05 | obs | 3 | 0 | 33.57 | 7.20 | 28.00 | 29.50 | 31.00 | 36.35 | 41.70 | 15.68 | 51.46 |
| Day 01 Hour 05 | cha | 3 | 0 | 7.17 | 4.67 | 2.00 | 5.20 | 8.40 | 9.75 | 11.10 | -4.44 | 18.78 |
| Day 01 Hour 05 | %cha | 3 | 0 | 27.04 | 16.77 | 7.69 | 21.98 | 36.27 | 36.72 | 37.17 | -14.60 | 68.69 |
