# Supplementary material for: Inhibition of merozoite invasion and transient de-sequestration by sevuparin in humans with Plasmodium falciparum malaria
Source: PLoS One. 2017 Dec 15;12(12):e0188754. doi: 10.1371/journal.pone.0188754 (PMC5731734; doi:10.1371/journal.pone.0188754)
Supplement: S4 Table — (DOCX) [file pone.0188754.s010.docx]

**S4 Table APTT (seconds) over time following sevuparin treatment in patients with uncomplicated malaria, part 2.**

|  |  | **n** | **Mi- ssing** | **Mean** | **SD** | **Min** | **Q1** | **Me- dian** | **Q3** | **Max** | **Lower**  **95CI** | **Upper**  **95CI** |
| --- | --- | --- | --- | --- | --- | --- | --- | --- | --- | --- | --- | --- |
| APTT (sec) | | | | | | | | | | | | |
| **Sevuparin 3.0 mg/kg** | | | | | | | | | | | | |
| Day 01 Hour screening | obs | 21 | 0 | 29.14 | 3.34 | 22.40 | 28.00 | 29.30 | 30.30 | 38.80 | 27.62 | 30.66 |
| Day 01 Hour predose | obs | 21 | 0 | 29.14 | 3.34 | 22.40 | 28.00 | 29.30 | 30.30 | 38.80 | 27.62 | 30.66 |
| Day 01 Hour predose | cha | 21 | 0 | 0.00 | 0.00 | 0.00 | 0.00 | 0.00 | 0.00 | 0.00 | - | - |
| Day 01 Hour predose | %cha | 21 | 0 | 0.00 | 0.00 | 0.00 | 0.00 | 0.00 | 0.00 | 0.00 | - | - |
| Day 01 Hour 02 | obs | 21 | 0 | 43.05 | 7.78 | 32.10 | 38.70 | 40.60 | 48.00 | 68.90 | 39.51 | 46.60 |
| Day 01 Hour 02 | cha | 21 | 0 | 13.91 | 8.71 | 4.30 | 9.40 | 11.10 | 16.80 | 44.80 | 9.94 | 17.87 |
| Day 01 Hour 02 | %cha | 21 | 0 | 49.92 | 36.96 | 14.01 | 31.37 | 38.93 | 53.85 | 185.89 | 33.09 | 66.74 |
| Day 01 Hour 05 | obs | 21 | 0 | 32.14 | 3.02 | 25.50 | 30.20 | 32.70 | 34.10 | 38.50 | 30.76 | 33.51 |
| Day 01 Hour 05 | cha | 21 | 0 | 3.00 | 4.07 | -3.70 | 0.00 | 2.40 | 4.90 | 14.40 | 1.14 | 4.85 |
| Day 01 Hour 05 | %cha | 21 | 0 | 11.50 | 16.35 | -9.54 | 0.00 | 8.25 | 16.61 | 59.75 | 4.05 | 18.94 |
| Day 01 Hour 11 | obs | 21 | 0 | 35.68 | 4.55 | 28.00 | 31.80 | 36.20 | 38.40 | 45.50 | 33.61 | 37.75 |
| Day 01 Hour 11 | cha | 21 | 0 | 6.53 | 6.03 | -4.10 | 3.30 | 5.50 | 8.80 | 21.40 | 3.79 | 9.28 |
| Day 01 Hour 11 | %cha | 21 | 0 | 24.27 | 24.81 | -10.57 | 11.67 | 17.92 | 30.25 | 88.80 | 12.98 | 35.56 |
| Day 01 Hour 17 | obs | 21 | 0 | 36.00 | 6.34 | 28.10 | 32.10 | 35.70 | 38.80 | 51.40 | 33.12 | 38.89 |
| Day 01 Hour 17 | cha | 21 | 0 | 6.86 | 7.07 | -3.10 | 2.10 | 5.00 | 10.20 | 23.00 | 3.65 | 10.08 |
| Day 01 Hour 17 | %cha | 21 | 0 | 24.96 | 26.43 | -7.99 | 7.00 | 14.79 | 38.78 | 95.44 | 12.93 | 36.99 |
| Day 01 Hour 23 | obs | 21 | 0 | 36.08 | 6.45 | 26.20 | 30.60 | 35.20 | 38.80 | 49.40 | 33.15 | 39.02 |
| Day 01 Hour 23 | cha | 21 | 0 | 6.94 | 7.14 | -2.30 | 2.10 | 5.70 | 9.40 | 22.20 | 3.69 | 10.19 |
| Day 01 Hour 23 | %cha | 21 | 0 | 25.20 | 26.59 | -5.93 | 7.37 | 20.23 | 35.27 | 92.12 | 13.09 | 37.30 |
| Day 02 Hour 29 | obs | 21 | 0 | 36.16 | 5.54 | 28.50 | 32.50 | 35.00 | 39.10 | 48.00 | 33.64 | 38.69 |
| Day 02 Hour 29 | cha | 21 | 0 | 7.02 | 6.20 | -0.70 | 2.40 | 4.80 | 8.80 | 21.30 | 4.20 | 9.84 |
| Day 02 Hour 29 | %cha | 21 | 0 | 25.43 | 23.76 | -2.33 | 7.84 | 16.84 | 38.84 | 88.38 | 14.62 | 36.25 |
| Day 02 Hour 32 | obs | 21 | 0 | 53.70 | 11.80 | 40.70 | 43.50 | 52.00 | 56.90 | 78.50 | 48.33 | 59.07 |
| Day 02 Hour 32 | cha | 21 | 0 | 24.56 | 12.43 | 11.20 | 15.10 | 21.50 | 27.20 | 50.50 | 18.90 | 30.22 |
| Day 02 Hour 32 | %cha | 21 | 0 | 86.79 | 48.78 | 37.97 | 52.31 | 64.79 | 119.73 | 205.81 | 64.59 | 108.99 |
| Day 02 Hour 35 | obs | 21 | 0 | 39.58 | 7.67 | 29.20 | 33.30 | 38.20 | 44.10 | 53.90 | 36.09 | 43.07 |
| Day 02 Hour 35 | cha | 21 | 0 | 10.43 | 8.10 | 1.00 | 4.60 | 8.60 | 13.80 | 29.80 | 6.75 | 14.12 |
| Day 02 Hour 35 | %cha | 21 | 0 | 37.44 | 32.32 | 3.27 | 16.14 | 27.32 | 45.54 | 123.65 | 22.73 | 52.15 |
| Day 02 Hour 41 | obs | 21 | 0 | 37.95 | 5.83 | 28.70 | 34.00 | 37.00 | 40.90 | 50.30 | 35.30 | 40.60 |
| Day 02 Hour 41 | cha | 21 | 0 | 8.81 | 6.46 | 1.10 | 4.20 | 7.50 | 10.60 | 24.20 | 5.87 | 11.75 |
| Day 02 Hour 41 | %cha | 21 | 0 | 31.66 | 25.18 | 3.61 | 13.68 | 25.42 | 39.92 | 100.41 | 20.20 | 43.12 |
| Day 02 Hour 47 | obs | 21 | 0 | 36.74 | 5.62 | 29.10 | 33.50 | 35.00 | 39.00 | 48.30 | 34.19 | 39.30 |
| Day 02 Hour 47 | cha | 21 | 0 | 7.60 | 6.67 | -0.60 | 3.70 | 5.80 | 12.70 | 24.00 | 4.56 | 10.64 |
| Day 02 Hour 47 | %cha | 21 | 0 | 27.88 | 26.69 | -2.02 | 12.09 | 18.64 | 45.71 | 99.59 | 15.73 | 40.03 |
| Day 03 Hour 53 | obs | 21 | 0 | 37.14 | 5.46 | 27.30 | 33.20 | 38.20 | 40.00 | 47.10 | 34.66 | 39.63 |
| Day 03 Hour 53 | cha | 21 | 0 | 8.00 | 6.05 | -1.20 | 3.30 | 6.80 | 12.00 | 23.00 | 5.25 | 10.75 |
| Day 03 Hour 53 | %cha | 21 | 0 | 28.95 | 24.82 | -4.21 | 11.74 | 25.68 | 44.10 | 95.44 | 17.65 | 40.25 |
| Day 03 Hour 59 | obs | 21 | 0 | 37.50 | 5.94 | 29.30 | 34.20 | 36.10 | 40.00 | 53.70 | 34.79 | 40.20 |
| Day 03 Hour 59 | cha | 21 | 0 | 8.35 | 6.92 | -0.50 | 3.70 | 6.80 | 9.80 | 29.60 | 5.20 | 11.50 |
| Day 03 Hour 59 | %cha | 21 | 0 | 30.43 | 28.12 | -1.67 | 13.04 | 22.22 | 37.26 | 122.82 | 17.63 | 43.23 |
| Day 03 Hour 65 | obs | 21 | 0 | 37.34 | 6.46 | 28.60 | 33.50 | 35.60 | 41.20 | 57.50 | 34.40 | 40.28 |
| Day 03 Hour 65 | cha | 21 | 0 | 8.20 | 8.21 | -5.30 | 3.00 | 6.50 | 11.30 | 33.40 | 4.46 | 11.93 |
| Day 03 Hour 65 | %cha | 21 | 0 | 30.59 | 33.28 | -13.66 | 11.28 | 23.38 | 41.41 | 138.59 | 15.44 | 45.74 |
| Day 03 Hour 68 | obs | 21 | 0 | 47.75 | 10.08 | 26.00 | 42.90 | 46.70 | 52.60 | 71.60 | 43.16 | 52.34 |
| Day 03 Hour 68 | cha | 21 | 0 | 18.61 | 11.63 | -4.00 | 11.60 | 16.10 | 26.00 | 47.50 | 13.31 | 23.91 |
| Day 03 Hour 68 | %cha | 21 | 0 | 67.24 | 47.05 | -13.33 | 45.14 | 54.58 | 90.44 | 197.10 | 45.82 | 88.66 |
| Day 03 Hour 71 | obs | 21 | 0 | 36.06 | 5.70 | 27.10 | 32.70 | 35.70 | 39.30 | 51.90 | 33.46 | 38.65 |
| Day 03 Hour 71 | cha | 21 | 0 | 6.91 | 6.79 | -2.90 | 3.10 | 5.40 | 9.30 | 27.80 | 3.82 | 10.00 |
| Day 03 Hour 71 | %cha | 21 | 0 | 25.54 | 27.71 | -9.67 | 9.48 | 17.82 | 34.60 | 115.35 | 12.92 | 38.15 |
| Day 07 | obs | 21 | 0 | 27.63 | 1.91 | 24.70 | 25.90 | 27.50 | 28.70 | 32.10 | 26.76 | 28.50 |
| Day 07 | cha | 21 | 0 | -1.51 | 2.81 | -6.70 | -3.30 | -1.90 | 0.20 | 5.20 | -2.79 | -0.24 |
| Day 07 | %cha | 21 | 0 | -4.37 | 9.91 | -17.27 | -10.89 | -6.60 | 0.70 | 23.21 | -8.88 | 0.14 |
| Day 14 | obs | 20 | 1 | 27.07 | 2.07 | 23.30 | 25.75 | 26.95 | 28.57 | 31.90 | 26.10 | 28.04 |
| Day 14 | cha | 20 | 1 | -2.03 | 2.64 | -6.90 | -3.15 | -2.10 | -0.58 | 3.60 | -3.27 | -0.80 |
| Day 14 | %cha | 20 | 1 | -6.27 | 8.80 | -20.41 | -10.85 | -7.12 | -2.20 | 16.07 | -10.39 | -2.15 |
