# Supplementary material for: Inhibition of merozoite invasion and transient de-sequestration by sevuparin in humans with Plasmodium falciparum malaria
Source: PLoS One. 2017 Dec 15;12(12):e0188754. doi: 10.1371/journal.pone.0188754 (PMC5731734; doi:10.1371/journal.pone.0188754)
Supplement: S5 Table — (DOCX) [file pone.0188754.s011.docx]

**S5 Table Summary of AEs reported in part 1 of study in patients with uncomplicated malaria treated with multiple doses sevuparin.**

|  | **Sevuparin**  **1,5 mg/kg, n=3** | **Sevuparin**  **3.0 mg/kg, n=3** | **Sevuparin**  **6.0 mg/kg, n=3** | **Total n=9** |
| --- | --- | --- | --- | --- |
| **Total number of AEs reported, n** | **5** | **10** | **9** | **24** |
| **Number of patients with at least 1 AE, n (%)** | **2 (66.7%)** | **3 (100%)** | **3 (100%)** | **8 (88.9%)** |
| **MedDRA SOC preferred term** |  |  |  |  |
| **Blood and lymphatic system disorders** |  |  |  |  |
| Thrombocytopenia |  | 1a |  | 1 |
| Anaemia |  |  | 1 | 1 |
| **Congenital, familial and genetic disorders** |  |  |  |  |
| Haemoglobinopathy |  | 1 |  | 1 |
| **Gastrointestinal disorders** |  |  |  |  |
| Nausea | 1 |  |  | 1 |
| Vomiting | 2 | 1 |  | 3 |
| **Investigations** |  |  |  |  |
| AST increased |  |  | 3 | 3 |
| ALT increased |  | 2 | 3 | 5 |
| Prolonged APTT |  | 1 |  | 1 |
| **Metabolism and nutrition disorders** |  |  |  |  |
| Hypocalcemia | 1 | 2 |  | 3 |
| Hyperkalemia |  |  | 1 | 1 |
| Hypokalemia | 1 |  |  | 1 |
| **Nervous system disorders** |  |  |  |  |
| Dizziness |  |  | 1 | 1 |

**^a^**Thrombocytopenia was present before start of sevuparin treatment
