# Supplementary material for: Inhibition of merozoite invasion and transient de-sequestration by sevuparin in humans with Plasmodium falciparum malaria
Source: PLoS One. 2017 Dec 15;12(12):e0188754. doi: 10.1371/journal.pone.0188754 (PMC5731734; doi:10.1371/journal.pone.0188754)
Supplement: S6 Table — (DOCX) [file pone.0188754.s012.docx]

**S6 Table Summary of AEs reported in part 2 of study in patients with uncomplicated malaria, treated with multiple doses of sevuparin.**

|  | **Sevuparin**  **3.0 mg/kg n=21** | **Control**  **n=23** |
| --- | --- | --- |
| **Total number of AEs reported, n** | **17** | **12** |
| **Number of patients with at least 1 AE, n (%)** | **7 (33.3%)** | **9 (39.1%)** |
| **MedDRA SOC** Preferred term | **n** | **n** |
| **Blood and lymphatic system disorders** |  |  |
| Eosinophilia | 1 |  |
| **Cardiac disorders** |  |  |
| Bradycardia |  | 1 |
| Ventricular extrasystoles | 1 | 1 |
| Sinus tachycardia | 1 |  |
| **Gastrointestinal disorders** |  |  |
| Nausea |  | 1 |
| **General disorders and administration site conditions** | | |
| Pyrexia |  | 1 |
| **Infections and infestations** |  |  |
| Urinary tract infection | 1 |  |
| Pharyngitis | 1 |  |
| **Investigations** |  |  |
| AST increased | 3 | 1 |
| ALT increased | 3 |  |
| Electrocardiogram QT prolonged | 1 | 1 |
| Platelets decreased | 1 | 3 |
| Bilirubin increased | 1 |  |
| ALP increased | 1 |  |
| Parasite stool test positive | 1 |  |
| **Metabolism and nutrition disorders** |  |  |
| Hypokalaemia |  | 1 |
| Decreased appetite | 1 | 1 |
| **Skin and subcutaneous tissue disorders** |  |  |
| Rash erythematous |  | 1 |
