# Supplementary material for: Inhibition of merozoite invasion and transient de-sequestration by sevuparin in humans with Plasmodium falciparum malaria
Source: PLoS One. 2017 Dec 15;12(12):e0188754. doi: 10.1371/journal.pone.0188754 (PMC5731734; doi:10.1371/journal.pone.0188754)
Supplement: S7 Table — Part 2. (DOCX) [file pone.0188754.s013.docx]

**S7 Table Cumulative AUC of late stage peripheral blood parasitemia (trophozoites and schizonts only) at 11 hours (primary endpoint) and at 1, 2, 3, 4 and 17 hours (secondary endpoints) after first dose of sevuparin. Part 2.**

| **Primary endpoint AUC**  (hours x parasites/µL) | **n** | **Geometric mean** | | **95%** | | **CI** | | |
| --- | --- | --- | --- | --- | --- | --- | --- | --- |
| **At 11 hours**  Control (N=23) | 21 | 41303 | | 23398 | | 72907 | | |
| Sevuparin 3.0 mg/kg (N=21) | 17 | 30350 | | 7306 | | 126083 | | |
| **Secondary endpoint AUC**  (hours x parasites/µL) | **n** | **Geometric mean** | **95%** | | **CI** | | |  |
| **At 1 hour** |  |  |  | |  | | |  |
| Control (N=23) | 21 | 1955 | 679 | | 5628 | | |  |
| Sevuparin 3.0 mg/kg (N=21) | 17 | 1616 | 366 | | 7125 | | |  |
| **At 2 hours** |  |  |  | |  | | |  |
| Control (N=23) | 21 | 3728 | 1225 | | 11345 | | |  |
| Sevuparin 3.0 mg/kg (N=21) | 17 | 4930 | 1479 | | 16431 | | |  |
| **At 3 hours** |  |  |  | |  | | |  |
| Control (N=23) | 21 | 8163 | 4037 | | 16507 | | |  |
| Sevuparin 3.0 mg/kg (N=21) | 17 | 7280 | 2094 | | 25306 | | |  |
| **At 4 hours** |  |  |  | |  | | |  |
| Control (N=23) | 21 | 11236 | 5663 | | 22291 | | |  |
| Sevuparin 3.0 mg/kg (N=21) | 17 | 9608 | 2657 | | 34741 | | |  |
| **At 17 hours** |  |  |  | |  | | |  |
| Control (N=23) | 21 | 74925 | 40603 | | 138259 | | |  |
| Sevuparin 3.0 mg/kg (N=21) | 17 | 47967 | 10714 | | 214746 | | |  |
|  | **p-value*** |  |  | |  | | |  |
| H1, mean [log] AUCs | 0.823 |  |  | |  | | |  |
| H2, mean [log] AUCs | 0.841 |  |  | |  | | |  |
| H3, mean [log] AUCs | 0.861 |  |  |  |  |  |  |  |
| H4, mean [log] AUCs | 0.813 |  |  |  |  |  |  |  |
| H11, mean [log] AUCs | 0.651 |  |  |  |  |  |  |  |
| H17, mean [log] AUCs | 0.536 | | | | | |  |  |
