# Supplementary material for: Inhibition of merozoite invasion and transient de-sequestration by sevuparin in humans with Plasmodium falciparum malaria
Source: PLoS One. 2017 Dec 15;12(12):e0188754. doi: 10.1371/journal.pone.0188754 (PMC5731734; doi:10.1371/journal.pone.0188754)
Supplement: S8 Table — (DOCX) [file pone.0188754.s014.docx]

**S8 Table Median (range) parasite clearance outcomes in the two groups in part 2 of study in patients with uncomplicated malaria.**

| **Parameter** | **n** | **Median** | **Min** | **Max** | **p-value*** |
| --- | --- | --- | --- | --- | --- |
| **PC50** (hours) |  |  |  |  |  |
| Control | 23 | 12.87 | 1.06 | 31.45 | 0.404 |
| Sevuparin 3.0 mg/kg | 21 | 12.66 | 0.77 | 27.39 | 0.404 |
| **PC90** (hours) |  |  |  |  |  |
| Control | 23 | 30.71 | 4.39 | 44.47 | 0.148 |
| Sevuparin 3.0 mg/kg | 21 | 25.59 | 2.63 | 37.02 | 0.148 |
| **PRR24** (%) |  |  |  |  |  |
| Control | 23 | 84.64 | 18.08 | 100 | 0.391 |
| Sevuparin 3.0 mg/kg | 21 | 87.44 | -7.69 | 100 | 0.391 |
| **PRR48** (%) |  |  |  |  |  |
| Control | 23 | 99.78 | 94.18 | 100 | 0.054 |
| Sevuparin 3.0 mg/kg | 21 | 99.96 | 98.61 | 100 | 0.054 |
| **Time to clearance**** (hours) | | | | | |
| Control | 23 | 59 | 53 | 65 | 0.720 |
| Sevuparin 3.0 mg/kg | 21 | 53 | 41 | 65 | 0.720 |

PC50 = median time to 50% of parasite clearance; PC90 = median time to 90% of parasite clearance; PRR24 = parasite reduction ratio at 24 hours and PRR48 = parasite reduction ratio at 48 hours, *Mann Whitney-U test, except time to clearance (log rank test); **Median time to clearance calculated using the Kaplan-Meier method and compared using the log rank test with 25th and 75th centiles reported in lieu of minimum and maximum values.
